# Molecular Mechanism of the *Saposhnikovia divaricata*–*Angelica dahurica* Herb Pair in Migraine Therapy Based on Network Pharmacology and Molecular Docking

**DOI:** 10.1155/2022/1994575

**Published:** 2022-11-26

**Authors:** Fengzhen Wu, Jing Liu, Zhengtong Cao, Tianqi Wang, Liang Ye, Minmin Zhu, Zhenguo Wang

**Affiliations:** ^1^Institute of Chinese Medical Literature and Culture, Shandong University of Traditional Chinese Medicine, Jinan 250355, Shandong, China; ^2^Shandong College of Traditional Chinese Medicine, Yantai 264199, Shandong, China; ^3^Medical College of Shaoguan University, Shaoguan 512005, Guangdong, China; ^4^Journal Editorial Board of Science and Technology Department, Nanjing University of Chinese Medicine, Nanjing 210023, Jiangsu, China; ^5^The First Clinical Medical College, Shandong University of Traditional Chinese Medicine, Jinan 250014, Shandong, China

## Abstract

**Objective:**

This work studied the molecular mechanism of the *Saposhnikovia divaricata*–*Angelica dahurica* herb pair (SAHP) in migraine treatment.

**Methods:**

The active ingredients of drugs were screened, and potential targets were predicted by the Traditional Chinese Medicine Systems Pharmacology (TCMSP), TCMID, ETCM, and other databases. Migraine-related targets were obtained by harnessing the GeneCards, DrugBank, OMIM, TTD, and other databases. The protein-protein interaction (PPI) network was constructed with STRING software by performing a Venn analysis with bioinformatics. Gene Ontology (GO) functional enrichment and the Kyoto Encyclopedia of Genes and Genomes (KEGG) pathway analysis were performed with the Metascape platform. The component-target-pathway (C-T-P) network was constructed with Cytoscape 3.7.2 software, and molecular docking was assessed with AutoDockVina software.

**Results:**

A total of 183 relevant targets and 39 active ingredients in migraine therapy were obtained from SAHP. The active ingredients and targets were screened according to topological parameters: wogonin, anomalin, imperatorin, prangenin, 2-linoleoylglycerol, and methylenetanshinquinone were identified as key active ingredients. PTGS2, PIK3CA, PIK3CB, PIK3CD, F2, and AR were identified as key targets. The molecular docking results demonstrated high binding activity between the key active ingredients and key targets. A total of 20 important signaling pathways, including neural signaling pathways, calcium signaling pathways, pathways in cancer, cAMP signaling pathways, and PI3K-Akt signaling pathways, were obtained through enrichment analysis.

**Conclusion:**

Migraine with SAHP is mainly treated through anti-inflammatory and analgesic effects. The herb pair can be used for migraine using “multicomponent, multitarget, and multipathway” approaches.

## 1. Introduction

Migraine is a universal chronic paroxysmal neurovascular illness, with recurrent or periodic attacks of throbbing headaches on one or both sides, often accompanied by autonomic symptoms such as nausea, vomiting, and photophobia, which can easily diminish a patient's ability to learn and work, as well as lower the quality of life. The current methods of treating migraine are mainly drug therapy, surgical therapy, and pressure therapy. Although these methods are effective, they have side effects [1]. Faced with these problems, traditional Chinese medicine (TCM) has significant merits in migraine therapy with its characteristics of being multifaceted, multitargeting, multimechanistic, and having slight toxicity and few side effects [2]. The *Saposhnikovia divaricata*–*Angelica dahurica* herb pair (SAHP) is frequently used in treating migraine and is a commonly used herb pair for migraine treatment [3]. TCM classifies migraine as “headaches” and “intractable headaches,” whose causes include external and internal injuries. It is only the wind that can climb the top of high mountains. Successive dynasties' physicians comprehensively applied the wind-expelling drug to treat migraine. The wind-expelling drug can not only treat external injury but also has a certain effect on internal injury [4]. Wind-expelling drugs are mild in taste and light in weight, have wind and wood properties, and are mainly used to treat diseases caused by pathogenic wind. As the representative wind-expelling drug pair, SAHP is capable of effectively treating the migraine and alleviating migraine, as well as of high safety and shaving side effects free.

**Figure 1:**
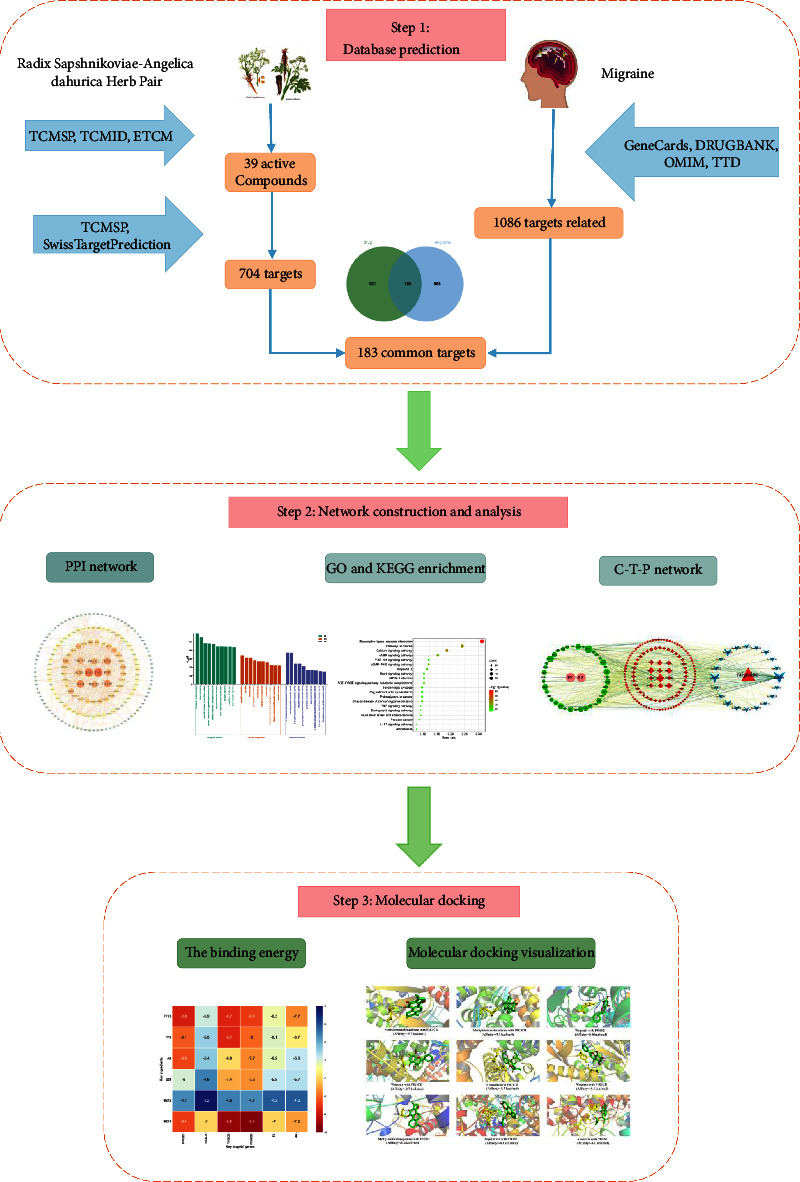
Flow diagram of the research.

Network pharmacology can predict the mechanism of drug functions in disease therapy as a whole, which is similar to the mechanism of multiconstituents and multitargeting function of traditional Chinese medicine compounds and conforms to the principle of a holistic view of traditional Chinese medicine [5]. Molecular docking can illustrate the mechanism of action between ligands and receptors at the molecular level and further analyze and identify the consequences of network pharmacology. Established on the mathematical model and complicated network model, network pharmacology research is capable of abstractly expressing the interaction relationship of TCM prescriptions in various systems of organisms in the form of networks. Based on combined network pharmacology and molecular docking technology, network pharmacology research has certain accuracy in predicting the function mechanism of drug therapy for diseases. Meanwhile, through regulating different target proteins, different active components act on different pathways, which serve therapy purposes and reflect the characteristics of multiple components, multiple target points, and multiple pathways of TCM [6]. Herein, the network pharmacology and molecular docking method were used to analyze the molecular mechanism of SAHP in migraine therapy to provide a basis for the clinical application of the pair. The working flowchart is shown in Figure 1.

## 2. Materials and Methods

### 2.1. Active Ingredient Screening and Target Prediction

The chemical components of SAHP were collected through the Traditional Chinese Medicine Systems Pharmacology (TCMSP, https://tcmspw.com/tcmsp.php) database [7], the Traditional Chinese Medicine Integrated Database [8] (TCMID, https://47.100.169.139:8000/tcmid/), and the Encyclopedia of Traditional Chinese Medicine [9] (ETCM, https://www.tcmip.cn/ETCM/) database, and the relevant literature for those components was excluded from the databases. Initial chemical components were obtained by integrating and deduplicating chemical components.

To screen initial chemical components and to screen the conditions for the chemical components included in the TCMSP, oral bioavailability (OB) ≥30% and drug-likeness (DL) ≥0.18 [10] were used. The prediction of the drug-likeness property is based on the Lipinski principle, which means that small molecular compounds have the drug-likeness property if they exceed three or more indicators, including molecular weight ≤500, lipid water partition coefficient ≤5, number of hydrogen bond donors ≤5, number of hydrogen bond receptors ≤10, and number of rotatable bonds ≤10 [11]. The screened chemical components were used as active components of SAHP. To predict the target of active ingredients, SwissTargetPrediction [12] (https://www.swisstargetprediction.ch/) and the TCMSP were used.

### 2.2. Migraine-Related Target Screening

GeneCards [13] (https://www.genecards.org/), DrugBank [14] (https://go.drugbank.com/), Online Mendelian Inheritance in Man [15] (OMIM, https://www.omim.org/), and the Therapeutic Target Database [16] (TTD, https://db.idrblab.org/ttd/) were searched using “migraine” as a keyword, and search results were integrated and deduplicated. The obtained targets were normalized on the UniProt database [17] (https://www.uniprot.org/), and the relevant targets of migraine treatment were acquired.

### 2.3. Potential Target Acquisition of SAHP for Migraine Therapy

By drawing a Venn diagram for the migraine-related targets of SAHP with the jvenn module of the bioinformatics platform [18] (https://www.bioinformatics.com.cn/), intersecting targets that are underlying targets for herb pairing for migraine therapy were obtained.

### 2.4. Protein-Protein Interaction (PPI) Network Establishment and Core Target Screening

PPI analysis is essential for the analysis of biological processes and for understanding complex mechanisms in cells. The PPI network was constructed using STRING [19] (https://cn.string-db.org/). The abovementioned intersecting targets were introduced into STRING, the species was selected as “homo sapiens,” and the interaction score was set to 0.4 to obtain the PPI network relationship. The PPI network chart was drawn with CytoScape3.7.2 software [20]. The degree of the value was calculated with the network analyzer function. The top 10 targets with degree scores could be called central targets.

### 2.5. Gene Ontology (GO) Functional Enrichment and the Kyoto Encyclopedia of Genes and Genomes (KEGG) Pathway Analysis

The potential targets of SAHP for migraine therapy were imported into the Metascape platform [21] (https://metascape.org/) for bioinformatic enrichment analysis, including a GO analysis of biological processes, molecular functions, and cellular components, and a pathway enrichment analysis of the KEGG. The species was set to “Homo sapiens,” and the significance level was *P* < 0.01. After the analysis, the top 10 GO functions and top 20 KEGG pathways were chosen, and the results were saved and visualized through the bioinformatics platform.

### 2.6. Network Construction of the Component-Target-Pathway (C-T-P)

The active ingredient information, potential target information, and top 20 KEGG pathway information of SAHP for the treatment of migraine were input to Cytoscape 3.7.2 software to establish SAHP for migraine treatment. The network of topological parameters containing degree, betweenness, and closeness was calculated. Then, the core target and primary effective active ingredients of SAHP for the treatment of migraine were determined using the NetworkAnalyzer analysis tool.

### 2.7. Molecular Docking Assessment

Molecular docking of the core targets chosen from SAHP for migraine treatment and their corresponding active ingredients was performed. Compound structures were downloaded from the PubChem database (https://pubchem.ncbi.nlm.nih.gov/), as were core proteins from the PDB database (https://www.rcsb.org/). A 3D structure of the main effective ingredients with the Chem 3D component was saved in ChemOffice in the mol2 format, the minimized force field applicable to ligand energy was MMFF94s, and its energy was minimized. Proteins were dewatered and hydrogenated using PyMOL software (https://www.Pymol.org), and compounds and target protein formats were converted to the pdbqt format with AutoDockTools software [22] (https://autodock.scripps.edu/resources/tools). Molecular docking was performed using AutoDockVina [23] (https://vina.scripps.edu/), and the corresponding calculation results were obtained. Active ingredients and targets were screened using better integration activity through the docking value affinity, in which the value <−4.25 kcal·mol^−1^ can be considered to have binding activity between ligands and targets, the value <−5.0 kcal·mol^−1^ can be considered to have better binding activity, and the value <−7.0 kcal·mol^−1^ has yet greater docking activity [24]. Finally, the molecular docking results were visualized using PyMOL software.

## 3. Results

### 3.1. Active Ingredient Screening and Target Prediction of SAHP

Through multiple database conjoint analyses, 39 active ingredients were screened, of which eight were standard components of *Saposhnikovia divaricata* and *Angelica dahurica*, 13 were derived from *Saposhnikovia divaricata*, and 18 were derived from *Angelica dahurica* (Table 1). After the target prediction results were merged and deduplicated, 704 targets were obtained.

### 3.2. Migraine-Related Target Screening Results

A total of 901, 224, 40, and 40 migraine-associated targets were obtained by searching the GeneCards, DrugBank, OMIM, and TTD disease gene databases, respectively, and 1086 targets were left after merging and removing any duplicates.

### 3.3. Potential Targets of SAHP for Migraine Therapy

**Figure 2:**
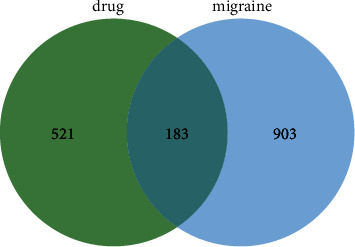
The Venn diagram of SAHP for migraine treatment.

A total of 1086 migraine-related targets were intersected with the 704 corresponding targets of SAHP obtained by screening, and 183 common targets were identified (Figure 2).

### 3.4. PPI Network Construction

**Figure 3:**
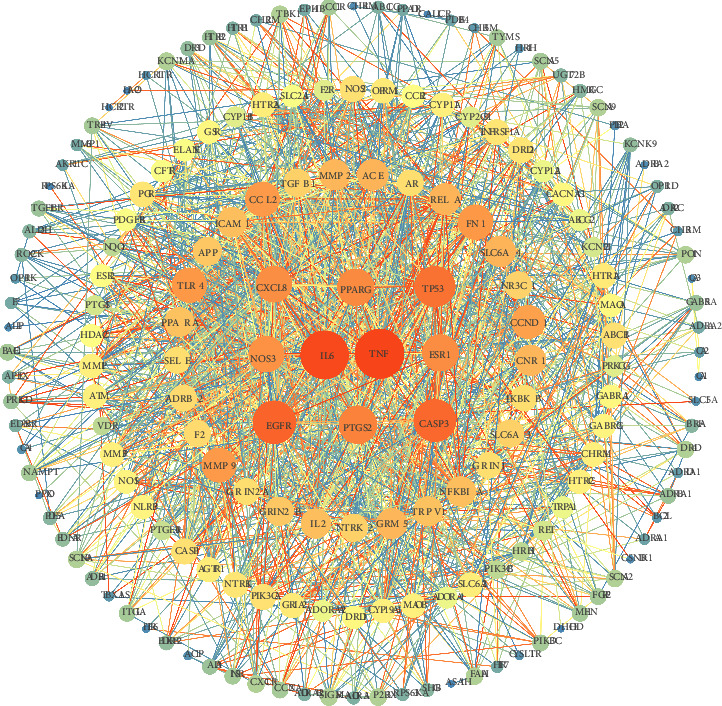
The PPI network of target proteins.

A total of 183 intersection targets were imported into the STRING database to gain PPI network information. The PPI network contains 183 nodes and 1744 edges, where nodes stand for proteins and edges stand for PPI. The imported protein interaction information in the PPI network was imported into Cytoscape 3.7.2 for visual analysis (Figure 3). The degree value was calculated with the network analyzer function after obtaining the PPI network. The top 10 targets by the degree value were TNF, IL6, EGFR, CASP3, TP53, PTGS2, PPARG, ESR1, CXCL8, and NOS3, and the degree values of these targets were 84, 81, 69, 67, 63, 57, 54, 53, 52, and 51, respectively. These targets are central targets at the center of the PPI network and act as links connecting other targets.

### 3.5. GO Functional Enrichment and KEGG Pathway Analysis

**Figure 4:**
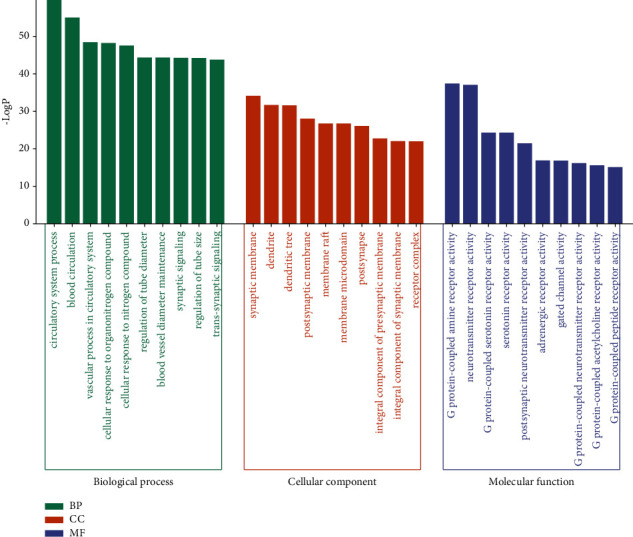
GO enrichment analysis.

GO and KEGG pathway enrichment analyses were performed on 183 potential targets of SAHP for migraine therapy using the Metascape platform. A total of 2267 GO enrichment analysis consequences were obtained through analysis, comprising 1921 biological process analysis results, 127 cellular component analysis results, and 219 molecular function analysis results. The top ten GO features were selected, and their results were saved and visualized by using the bioinformatics platform (Figure 4). Visualization shows that the main biological processes involved were circulatory system processes, blood circulation, vascular processes in the circulatory system, cell responses to organonitrogen compounds, and cell responses to nitrogen compounds. The main relevant cellular constituents comprised synaptic membranes, dendrites, dendritic trees, postsynaptic membranes, and membrane rafts. The main molecular functions involved were G protein-coupled amine receptor activities, neurotransmitter receptor activities, G protein-coupled serotonin receptor activities, serotonin receptor activities, and postsynaptic neurotransmitter receptor activities.

**Figure 5:**
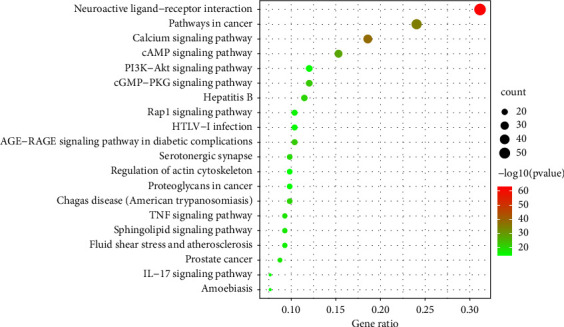
KEGG pathway enrichment analysis.

A total of 321 results were acquired from KEGG analysis, and the top 20 items of KEGG pathway information were chosen for input and visualized through the bioinformatics platform. KEGG pathways were mainly related to neuroactive ligand-receptor interactions, calcium signaling pathways, pathways in cancer, cAMP signaling pathways, and PI3K-Akt signaling pathways (Figure 5).

### 3.6. Network Analysis Results of C-T-P

**Figure 6:**
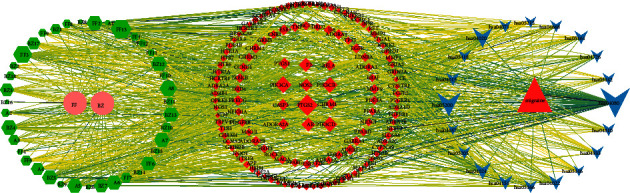
The component-target-pathway network.

CytoScape3.7.2 was used to establish the C-T-P network diagram of SAHP for migraine therapy (Figure 6). Network topological parameters were studied using its implanted tools to obtain constituents and core action targets. The network consists of 191 nodes and 1130 edges.

The top six active ingredients in network topology parameters were wogonin, anomalin, imperatorin, prangenin, 2-linoleoylglycerol, and methylenetanshinquinone. These active ingredients may play a vital role in migraine therapy using SAHP (Table 2). The top six targets for network topology parameters were PTGS2, PIK3CA, PIK3CB, PIK3CD, F2, and AR. These targets may be the key targets for SAHP in migraine therapy (Table 3).

### 3.7. Molecular Docking Assessment

**Figure 7:**
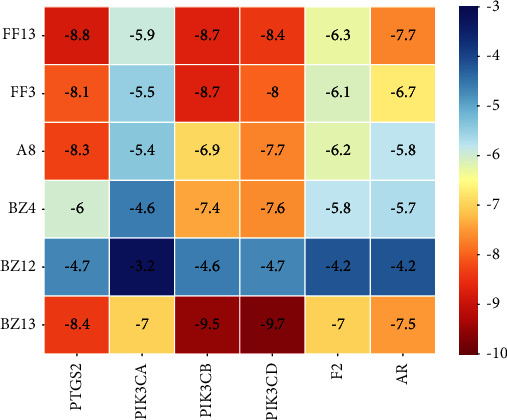
The binding energy of key components and key targets.

**Figure 8:**
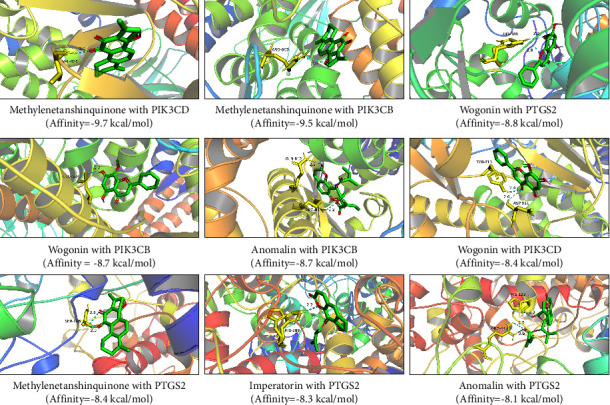
Molecular docking visualization of key components and key targets.

To clarify the binding activity among target proteins and corresponding components, the top six targets (i.e., PTGS2, PIK3CA, PIK3CB, PIK3CD, F2, and AR) in the C-T-P network diagram of the SAHP for the treatment of migraine and the top six active ingredients (i.e., wogonin, anomalin, imperatorin, prangenin, 2-linoleoylglycerol, and methylenetanshinquinone) underwent molecular docking separately Based on the same method, subsequently, 36 groups of receptor-ligand docking consequences were obtained. During the process of molecular docking, intermolecular forces shall be considered. The intermolecular forces in the study are mainly hydrogen bonds.  Figure 7 shows molecular docking scores, Among the 36 groups of receptor-ligand results, there were 29 groups with affinity <−5 kcal·mol^−1^, accounting for 80.56%, and 15 groups with affinity <−7 kcal·mol^−1^, accounting for 41.67%. It indicates that the screened potential key effective constituents have a good binding activity using key targets.  Figure 8 shows the docking modes of some core compounds. The methylenetanshinquinone compound formed a hydrogen bond with amino acid residues VAL-828 in PIK3CD, ARG-663 in PIK3CB, and SER-126 in PTGS2. The wogonin compound formed a hydrogen bond with amino acid residues HIS-388 in PTGS2, LEU-634 in target point PIK3CB, and TYR-813 and ASP-911 in target point PIK3CD. The anomalin compound formed a hydrogen bond with amino acid residues GLN-812 and ARG-815 in PIK3CB and HIS-122 and ARG-44 in target point PTGS2. The imperatorin compound formed a hydrogen bond with the amino acid residue HIS-388 in PTGS2. There was a minimum of one hydrogen bond formed between each target ligand and active compound residues, which demonstrates the scientific nature and reliability of the prediction of research.

## 4. Discussion

Migraine is a common clinical primary headache characterized by high morbidity and a high recurrence rate. The disease has a long course and is hard to recover. It seriously impacts the patient's life, work, and study and significantly reduces their quality of life [25]. Regarding migraine pathogenesis, modern medicine generally accepts trigeminovascular theory. The central sensitization in the trigeminal nerve pathway is closely related to inflammatory mediators released by microglia in the caudate nucleus of the trigeminal nerve, and neurogenic inflammation is considered the basic pathological feature of migraine [26, 27]. There remains an unmet need for effective and safe drugs to treat migraine. SAHP is widely used in migraine treatment. *Saposhnikovia divaricata* is considered sweet and spicy, warm in nature, attributed to the bladder meridian, liver meridian, and spleen meridian. It relieves rheumatism and exterior syndrome, overcomes dampness, and relieves pain. Modern pharmacological studies have confirmed that *Saposhnikovia divaricata* has the functions of antipyretic, analgesic, anti-inflammatory, and antioxidant effects and promotes blood circulation [28]. *Angelica dahurica* is sweet and warm in nature, attributed to the stomach meridian, large intestine meridian, and lung meridian, and it relieves exterior syndrome and rheumatism and dissipates cold and pain. *Angelica dahurica* can reduce the behavioral manifestations of migraine, reduce the secretion of ENK, increase the content of *β*-EP, and decrease the expression of C-FOS [29]. The compatibility of *Saposhnikovia divaricata* and *Angelica dahurica* is in line with migraine pathogenesis, and it has a significant curative effect on migraine. It can effectively and safely relieve headache symptoms.

This study has six key active ingredients (i.e., wogonin, anomalin, imperatorin, prangenin, 2-linoleoylglycerol, and methylenetanshinquinone) that were preliminarily screened out using the network pharmacology method of SAHP for migraine treatment. Wogonin can effectively prevent and reduce inflammatory responses by regulating the COX-2 pathway and inflammatory mediators, and it exercises a therapeutic effect on chronic neuroinflammation [30]. Anomalin can inhibit NF-*κ*B signaling transduction, regulate iNOS, COX-2, TNF*α*, MAPKs, and CREB pathways, and has a therapeutic effect on inflammatory pain [31]. Imperatorin is the main active component of *Angelica dahurica*, having anti-inflammatory [32], analgesic [33], and vasodilating [34] effects. It can regulate the contents of PGE2, 5-HT, and CGRP in the brain tissue of a migraine rat model and reduce NO content in peripheral circulation [35]. Prangenin is a coumarin component in *Angelica dahurica* [36]. Animal experiments have demonstrated that the total amount of coumarin of *Angelica dahurica* has anti-inflammatory and analgesic effects, significantly inhibiting the serum level of PGE_2_ and reducing the content of TNF*α* in blood [37]. 2-Linoleoylglycerol is a local exhilarant of human cannabinoid type 1 receptors that inhibit the activity of 2-arachidonic acid glyceride and cannabinoid amides, with the potential to treat painful diseases [38]. Methylenetanshinquinone shows anti-inflammatory activity and can act on the NF-*κ*B pathway to inhibit the expression of inflammatory factors in macrophage THP-1 induced by LPS [39].

It was found that PTGS2, PIK3CA, PIK3CB, PIK3CD, F2, and AR were key targets combined with the PPI network and the C-T-P network. PTGS2 is a core enzyme in the biosynthesis of prostaglandins, also called cyclooxygenase-2 (COX-2). It overexpresses under mechanical, chemical, and physical stimuli, and it plays a vital role in the formation of inflammation [40]. Research has indicated that COX-2 is closely associated with migraine pathogenesis [41]. PIK3CA, PIK3CB, and PIK3CD are important genes involved in cell proliferation, G protein-coupled receptor signal transduction, and cancer gene expression, and they may be related to migraine caused by glial neuron tumors [42]. To date, it is generally accepted that migraine is associated with primary and secondary coagulation abnormalities [43, 44], and F2, as prothrombin, plays a significant role in migraine pathogenesis [45, 46]. Abnormal sex hormone receptors can lead to migraine [47]. AR is an androgen receptor gene; animal experiments have demonstrated that androgen levels are actively related to migraine risk [48].

The results of the KEGG study have indicated that the main pathways of SAHP in migraine therapy are related to neuroactive ligand-receptor interaction, calcium signaling pathways, pathways in cancer, cAMP signaling pathways, and PI3K-Akt signaling pathways. The nerve signal transmission pathway is closely associated with nervous functions, and it is a collection of receptors and ligands associated with the plasma membrane and intracellular and extracellular signaling pathways [49]. These pathways involve the synthesis and release of transmitters at synapses and their interaction with receptors so that pain signals are transmitted to the central nervous system [50]. The calcium signaling pathway plays a significant role in neurodegenerative and neuropsychiatric diseases, and gene mutations in this pathway are significantly correlated with the pathogenesis of migraine [51]. Pathways in cancers are very complex metabolic pathways involving the cAMP signaling pathway, P13K-Akt, NF-*κ*B, and other pathways [52]. cAMP (cyclic adenosine monophosphate) response element-binding protein (CREB) is widely expressed in bodies, and all cells in the brain have a certain expression. Many target genes of CREB are linked to central sensitization, which correlates with depression and pain [53]. Other research has found that the PI3K/Akt signaling pathway is activated in the brain tissue of migraine rat models [54]. The activation of this pathway can protect against cerebral ischemia-reperfusion injury, and nerve protection function and suppresses neuronal autophagy are associated with oxidative stress [55]. NF-*κ*B is an important pathway in the process of migraine; it is associated with upregulating the release of downstream inflammatory mediators and mediating neurogenic inflammation [56].

## 5. Conclusion

To conclude, SAHP mainly treats migraine through its anti-inflammatory and analgesic effects. Based on the network pharmacology method and molecular docking technology, this research initially revealed the molecular mechanism of SAHP to treat migraine through multicomponent, multitarget, and multibiological pathway regulation.

Herein, we believe that the key active ingredients are wogonin, anomalin, imperatorin, prangenin, 2-linoleoylglycerol, and methylenetanshinquinone components of SAHP for migraine treatment. Such active ingredients may influence neuroactive ligand-receptor interaction, calcium signaling pathways, signaling pathways in cancer, cAMP signaling pathways, and PI3K pathways by acting on multiple targets such as PTGS2, PIK3CA, PIK3CB, PIK3CD, F2, and AR to treat migraine. Through network pharmacology and molecular docking technology, this study introduces the potential mechanism of SAHP in treating migraine, which not only provides a reference for the clinical treatment of migraine but also provides some theoretical support and data support for the development of new drugs to treat migraine.

Meanwhile, this study had certain limitations. According to multiple databases, the study mainly predicted the pharmacology mechanism of SAHP in migraine therapy. However, to ensure reliability and rationality of forecast results, it is necessary to conduct further in vivo pharmacological research development of SAHP and conduct further animal or cell experiments.

## Figures and Tables

**Table 1:** Active ingredients of SAHP.

MolID	MolName	OB (%)	DL	Herb
FF1	Divaricatacid	87.00	0.32	*Saposhnikovia divaricata* (Fangfeng, FF)
FF2	Cleomiscosin A	68.83	0.66	*Saposhnikovia divaricata* (Fangfeng, FF)
FF3	Anomalin	59.65	0.66	*Saposhnikovia divaricata* (Fangfeng, FF)
FF4	11-Hydroxy-sec-o-beta-d-glucosylhamaudol_qt	50.24	0.27	*Saposhnikovia divaricata* (Fangfeng, FF)
FF5	Phellopterin	43.39	0.28	*Saposhnikovia divaricata* (Fangfeng, FF)
FF6	Decursin	39.27	0.38	*Saposhnikovia divaricata* (Fangfeng, FF)
FF7	5-O-Methylvisamminol	37.99	0.25	*Saposhnikovia divaricata* (Fangfeng, FF)
FF8	Glyceryl monolinoleate	37.18	0.30	*Saposhnikovia divaricata* (Fangfeng, FF)
FF9	Beta-sitosterol	36.91	0.75	*Saposhnikovia divaricata* (Fangfeng, FF)
FF10	Glyceryl monooleate	34.13	0.30	*Saposhnikovia divaricata* (Fangfeng, FF)
FF11	Ledebouriellol	32.05	0.51	*Saposhnikovia divaricata* (Fangfeng, FF)
FF12	Divaricatol	31.65	0.38	*Saposhnikovia divaricata* (Fangfeng, FF)
FF13	Wogonin	30.68	0.23	*Saposhnikovia divaricata* (Fangfeng, FF)
BZ1	Sen-byakangelicol	58.00	0.61	*Angelica dahurica* (Baizhi, BZ)
BZ2	Marmesin	50.28	0.18	*Angelica dahurica* (Baizhi, BZ)
BZ3	Stigmasterol	43.83	0.76	*Angelica dahurica* (Baizhi, BZ)
BZ4	Prangenin	43.60	0.29	*Angelica dahurica* (Baizhi, BZ)
BZ5	ZINC03860434	43.59	0.35	*Angelica dahurica* (Baizhi, BZ)
BZ6	Pabulenol	42.85	0.26	*Angelica dahurica* (Baizhi, BZ)
BZ7	Byakangelicol	41.42	0.36	*Angelica dahurica* (Baizhi, BZ)
BZ8	Oxypeucedanin hydrate	39.99	0.29	*Angelica dahurica* (Baizhi, BZ)
BZ9	Skimmin	38.35	0.32	*Angelica dahurica* (Baizhi, BZ)
BZ10	Cholesterol	37.87	0.68	*Angelica dahurica* (Baizhi, BZ)
BZ11	Propylene glycol monoleate	37.60	0.26	*Angelica dahurica* (Baizhi, BZ)
BZ12	2-Linoleoylglycerol	37.28	0.30	*Angelica dahurica* (Baizhi, BZ)
BZ13	Methylenetanshinquinone	37.07	0.36	*Angelica dahurica* (Baizhi, BZ)
BZ14	Neobyakangelicol	36.18	0.31	*Angelica dahurica* (Baizhi, BZ)
BZ15	Alloisoimperatorin	34.80	0.22	*Angelica dahurica* (Baizhi, BZ)
BZ16	Squalene	33.55	0.42	*Angelica dahurica* (Baizhi, BZ)
BZ17	Cnidilin	32.69	0.28	*Angelica dahurica* (Baizhi, BZ)
BZ18	Ethyl oleate	32.40	0.19	*Angelica dahurica* (Baizhi, BZ)
A1	Nodakenin	57.12	0.69	*Saposhnikovia divaricata* (Fangfeng, FF) *Angelica dahurica* (Baizhi, BZ)
A2	Isoimperatorin	45.46	0.23	*Saposhnikovia divaricata* (Fangfeng, FF) *Angelica dahurica* (Baizhi, BZ)
A3	Ethyl linoleate	42.00	0.19	*Saposhnikovia divaricata* (Fangfeng, FF) *Angelica dahurica* (Baizhi, BZ)
A4	Phellopterin	40.19	0.28	*Saposhnikovia divaricata* (Fangfeng, FF) *Angelica dahurica* (Baizhi, BZ)
A5	11,14-Eicosadienoic acid, methyl ester	39.67	0.23	*Saposhnikovia divaricata* (Fangfeng, FF) *Angelica dahurica* (Baizhi, BZ)
A6	Beta-sitosterol	36.91	0.75	*Saposhnikovia divaricata* (Fangfeng, FF) *Angelica dahurica* (Baizhi, BZ)
A7	Alloimperatorin	36.31	0.22	*Saposhnikovia divaricata* (Fangfeng, FF) *Angelica dahurica* (Baizhi, BZ)
A8	Imperatorin	34.55	0.22	*Saposhnikovia divaricata* (Fangfeng, FF) *Angelica dahurica* (Baizhi, BZ)

**Table 2:** Network topology parameters of key components.

MolID	Name	Degree	Betweenness centrality	Closeness centrality
FF13	Wogonin	38	0.0585	0.4338
FF3	Anomalin	34	0.0392	0.4260
A8	Imperatorin	30	0.0311	0.4204
BZ4	Prangenin	28	0.0261	0.4167
BZ12	2-Linoleoylglycerol	28	0.0391	0.4167
BZ13	Methylenetanshinquinone	28	0.0235	0.4077

**Table 3:** Network topological parameters of key targets.

Name	Degree	Betweenness centrality	Closeness centrality
PTGS2	39	0.0769	0.5205
PIK3CA	29	0.0361	0.4738
PIK3CB	26	0.0262	0.4600
PIK3CD	25	0.0232	0.4556
F2	23	0.0231	0.4600
AR	22	0.0206	0.4368

## Data Availability

The relevant data are available from the corresponding author upon reasonable request.

## Supplementary Materials

Supplementary Materials Table S1: 704 targets of SAHP. Table S2: 1086 targets of migraine. Table S3: 183 common targets of SAHP and migraine. Table S4: the result of GO functional enrichment analysis. Table S5: the result of KEGG pathway enrichment analysis.
