# Supplementary material for: Molecular Mechanism of the Saposhnikovia divaricata–Angelica dahurica Herb Pair in Migraine Therapy Based on Network Pharmacology and Molecular Docking
Source: Evid Based Complement Alternat Med. 2022 Nov 26;2022:1994575. doi: 10.1155/2022/1994575 (PMC9722292; doi:10.1155/2022/1994575)
Supplement: Supplementary Materials — Table S1: 704 targets of SAHP. Table S2: 1086 targets of migraine. Table S3: 183 common targets of SAHP and migraine. Table S4: the result of GO functional enrichment analysis. Table S5: the result of KEGG pathway enrichment analysis. [file 1994575.f1.zip › Table S1.704 targets of SAHP.pdf]

Target
MME
MKNK2
KDM4A
PDE5A
CYSLTR1
KDM4C
GRK6
CSNK2A1
MAPK8
ACE
MPI
IMPDH2
IMPDH1
FABP4
EGLN1
PYGL
FLT1
KIT
KDR
ANPEP
LTA4H
ECE1
KDM4D
HAO2
HMGCR
AMPD3
MMP16
MMP9
MMP1
ACLY
MMP8
CASP3
TYMS
IGFBP3
ERN1
CXCR2
PARP1
MAPK1
GPR35
ERBB2
CASP6
CASP7
CASP8
FFAR1
CASP1

CASP2
PTGDR2
ITGB1
PYGM
EGLN3
FOLH1
GSK3B
GSK3A
TOP1
KDM3A
GSR
KDM5B
NEU2
PTGIR
MARS
FDFT1
IDE
AR
DPP4
NOS2
PTGS2
F2
PIK3CA
CDK2
CDK1
PRKCD
CTSV
SYK
CHEK1
WEE1
AKR1B10
RET
LNPEP
MTOR
MAP2K1
AKT1
KCNH2
BRAF
ADORA2A
ADORA3
PIK3C2A
PIK3C2G
PIP4K2C
RAF1
FLT3
FKBP1A
MYLK

DAPK3
DAPK1
JAK1
CDK7
DAPK2
PIK3CD
PRKDC
PIK3CB
PIK3CG
PI4KB
CHUK
TYK2
CSNK2A2
CLK1
CLK2
CLK3
DYRK2
CDC42BPA
HIPK2
HIPK3
HIPK1
DYRK1B
PIK3C2B
LIMK2
RIOK2
MAP3K19
CXCR1
MMP13
HSP90AA1
HSP90AB1
MAP3K14
EIF4A1
PAK3
PAK2
PAK1
ABL1
EGFR
HCK
EPHB4
ALPL
PLAA
ADAM17
CFTR
GBA
ADCY1
PNP
F9

ADORA1
MMP3
JUN
ABCC1
NTRK1
DRD1
DRD2
MMP7
ADAM10
MAP3K7
EDNRB
EDNRA
AKT2
SLC5A2
HDAC6
HDAC2
P2RY12
HDAC1
KCNMA1
CAMKK2
F7
F10
TOP2A
ESR1
NOS3
NCOA1
SCN5A
PRSS1
CNR1
ELANE
CNR2
CSF1R
KCNJ6
RBP4
SCN2A
HCRTR2
SCN10A
KCNJ5
P2RX3
JAK3
JAK2
SCN9A
CTSS
CTSL
PDE4B
ELOVL6
PPIA

GRM5
TSPO
P2RX7
KCNK3
KCNK9
VCP
IDH1
CHRM2
CHRM1
CHRM3
BDKRB1
NPY5R
HTR1A
HTR7
HTR6
MAPK11
HCRTR1
ITGAV
SRC
CTSK
TACR3
TRPA1
CFD
TGM2
TGM1
CMA1
F13A1
PABPC1
CCND3
CPT1A
CCND1
IKBKB
CCNE2
CCNB3
DRD4
FAAH
GRM4
PDE7A
NOS1
PDE10A
TRPV1
CYP11B1
CYP19A1
PSEN2
CYP11B2
CHRM4
PRKCG

PREP
PSMB5
SAE1
PFKFB3
GCK
CTSB
PTK2
CCKBR
GRM2
DBF4
CCNA2
PTGS1
PIM1
BACE1
MAOA
GABRB3
GABRA2
MAPK14
NUDT1
MCHR1
DUT
MAOB
PDE8B
GYS1
SRD5A1
KCNA3
ADORA2B
TAAR1
CA9
HCAR2
HSF1
CYP1A2
SLC9A1
CA2
CA1
PDPK1
NAAA
DNMT3A
RPS6KB1
PTGES
KCNA5
PLA2G7
CD38
APP
CDC7
HRH3
PTGER3

HRH4
CRHR1
PDE2A
DYRK1A
CLK4
ACHE
RXRA
PTPN1
GCGR
ASAH1
OGT
MAP4K4
SCD
HPGD
AURKA
AURKB
ROCK2
PDE4D
PDE3A
PDE3B
HSD11B1
PKM
EPHX2
ADAMTS5
MTNR1A
MTNR1B
GRM1
TBXAS1
FNTA
SLC5A1
BRD4
TDP2
IDO1
PARP2
SRD5A2
CHEK2
PDE4A
PDE4C
PLK1
ALOX5AP
PLK3
PLK2
PDGFRB
FLT4
CHRNA4
EPHX1
PPARG

CHRNA7
SLC10A2
HSD17B2
HSD17B1
CCNC
CDK8
RORC
GRIN1
PLAU
SIRT2
ICAM1
SELE
PIM2
PTAFR
TTK
ADRB2
ADRA1A
ADRA1B
ESR2
GABRA1
OPRM1
PRKCA
PRKCQ
ALOX5
PTPN2
AVPR1A
LPAR6
LPAR5
S1PR3
S1PR1
LPAR3
LPAR2
LPAR1
LPAR4
ENPP2
PRKCH
CES2
CYP17A1
ITGAL ICAM1
AURKAIP1
GPR119
MAP2
CDK6
CDK4
CSNK1D
CDK9
IKBKE

PLK4
STK16
CDK5
SLC8A1
CDK3
CDK16
MAPK15
CDK18
MAPK7
TBK1
CDK17
S1PR5
S1PR4
MDM2
CYP51A1
NPC1L1
NR1H3
SHBG
SREBF2
CYP2C19
SLC6A2
BCHE
RORA
SERPINA6
SLC6A4
G6PD
NR1I3
VDR
NR1H2
CDC25A
PTGER1
PTGER2
DHCR7
GLRA1
PPARD
SQLE
PTPN6
NR3C1
CDC25B
SHH
UGT2B7
HSD11B2
POLB
NR3C2
NCOA2
PGR
PRKCE

BCL2L1
ACACB
PPARA
ALOX15
HIF1A
CCR4
AKR1B1
OPRD1
ABCB1
NTRK2
KDM4E
XDH
ALOX12
CYP1A1
ABCG2
SLC22A12
CYP1B1
CA4
MCL1
CA7
CA12
CDK5R1
CBR1
TERT
CA6
PTPRS
MPG
MMP2
LCK
MMP12
ARG1
MAPT
INSR
MPO
PIK3R1
CA3
CA14
MET
CA13
CAMK2B
PLA2G1B
CA5A
APEX1
AKR1C2
AKR1C1
AKR1C3
AKR1C4

AKR1A1
ODC1
PLA2G2A
HSP90B1
TNKS
TTR
NOX4
AVPR2
NAE1
AHSA1
BAX
BCL2
BBC3
PRKACA
CASP9
TP53
CDKN1A
EIF6
FN1
IL6
CXCL8
CCL2
TEP1
RELA
TNF
DDAH1
WNT3A
TNKS2
LIPG
LPL
GHSR
TNNC1
PLEC
XIAP
ERBB4
SIGMAR1
LRRK2
P4HTM
FBP1
GABRA5
KMT5A
CDC25C
DUSP3
FGFR1
HIPK4
SBK1
BLK

PHKG2
MAPK10
MYLK2
RPS6KA4
IRAK1
PI4KA
IRAK4
PRKD1
STK17B
STK10
EPHA5
PHKG1
ABL2
EPHA8
MAPK9
SLK
FRK
STK36
GAK
TXK
STK17A
EPHA6
TNIK
MKNK1
DPEP1
HTR2A
ADRA2A
ADRB1
CTRB1
IGHG1
SLC6A3
TLR4
CCR1
CCR2
ITGB7
BRD2
BRD3
AOC3
OXTR
NQO1
METAP2
PFKFB4
HTR1B
NQO2
PTK2B
CTSD
STK3

CCNE1
GPBAR1
GABRG2
PGGT1B
TNFRSF1A
CPB1
MEN1
TRPC6
TRPC3
TRPV4
MAP3K8
SLC2A1
SLC2A3
SLC2A2
CAPN1
PTGFR
AGTR1
KCNN4
SLC6A9
BAD
BCL2L2
BCL2L10
BCL2A1
SCARB1
TDO2
NMBR
NFKBIA
MALT1
PLCG2
ENPP1
LDHA
TMIGD3
LIMK1
KIF11
ADK
ALDH2
IL2
MANBA
HSPA8
HSPA5
HK2
HK1
GRK1
GAA
ADA
GAPDH
AHCY

HTR2B
ADRA2C
PRKCB
DNM1
BACE2
NLRP3
FABP3
FABP5
FABP1
TGFBR1
SMO
ADRA1D
PDGFRA
FGFR2
ZAP70
GRIN2A
GRIA2
PRF1
SMG1
AGPAT2
BTK
ITGAL
PTPN11
EED
CES1
RBBP4
PTPRC
TLR9
BDKRB2
NR4A1
CACNA1C
IGF1R
CHRM5
XPO1
DHODH
CCR9
DHFR
CSNK1A1
TK2
TLR7
STS
EIF2AK3
CTRC
EPAS1
TAS2R31
PTPN13
PTPRF

ACP1
MGLL
ROCK1
CA5B
OPRK1
HPGDS
ATM
C5AR1
F2R
GRIN2B
RARG
RARB
RARA
RXRG
NPY2R
QPCT
ACACA
MGAT2
TBXA2R
PDE6D
ITK
SLC6A15
CETP
MAP3K5
YES1
CALCRL
SOAT1
SSTR4
HTR2C
NAMPT
PPID
SGPL1
MERTK
SNCA
camC
CHRNA2
PON1
TGFB1
DRD3
HDAC8
RPS6KA3
HDAC3
ADRA2B
BRS3
MAPKAPK2
CSNK1G1
PPOX

PTGER4
