# Supplementary material for: Molecular Mechanism of the Saposhnikovia divaricata–Angelica dahurica Herb Pair in Migraine Therapy Based on Network Pharmacology and Molecular Docking
Source: Evid Based Complement Alternat Med. 2022 Nov 26;2022:1994575. doi: 10.1155/2022/1994575 (PMC9722292; doi:10.1155/2022/1994575)
Supplement: Supplementary Materials — Table S1: 704 targets of SAHP. Table S2: 1086 targets of migraine. Table S3: 183 common targets of SAHP and migraine. Table S4: the result of GO functional enrichment analysis. Table S5: the result of KEGG pathway enrichment analysis. [file 1994575.f1.zip › Table S2.1086 targets of migraine.pdf]

Target
mdlB
DECR1
OGDH
HMGCR
Prkab1
HTR1A
HTR1B
HTR2C
HTR1D
HTR1E
HTR1F
HTR2A
HTR2B
HTR3A
HTR4
HTR6
HTR7
HEL-S-89n
ADORA1
ADORA2A
ADORA2B
ADORA3
AKR1C1
ALPL
ADRA1A
ORM1
ADRA1B
ADRA1D
ADRA2A
ADRA2B
ADRA2C
AMACR
MAOA
ACE
BCL2
NAT2
ABCG2
NTRK2
ADRB1
ADRB2
ADRB3
ABCB11
CALCA
CALCB
CALCRL

PDE1A
PDE1B
CALM1
PDE4B
CA1
CA2
CA3
CA4
CASP1
CASP3
TP53
mbtI
CXCR1
cGIPDE1
CCNA2
CFTR
MT-CO1
CYP1A1
CYP1A2
CYP1B1
CYP2A6
CYP2B6
CYP2C19
CYP2C8
CYP2C9
CYP2D6
CYP2E1
CYP3A4
CYP3A5
CYP3A7
DRD3
OPRD1
DHODH
pyrDA
DPYD
FMO3
PRKDC
DRD2
EDNRA
epiD
SULT1E1
MAPK15
FABP2
GLU2
BLVRB
fpr
NDOR1

POR
azoR1
azr
CCND1
GABRA1
HBA1
HBA2
HBB
KCNH2
NTRK1
HRH1
HRH2
HRH3
HRH4
HNMT
HDAC2
HDAC9
HAO1
HAO2
IKBKB
ITPR1
fni
GRIK2
OPRK1
LDLR
LYZD2
MTHFR
SLC16A1
SLC47A1
SLC47A2
ABCC1
CHRM2
CHRM1
CHRM3
CHRM4
CHRM5
OPRM1
MYC
NQO1
NDUFAF6
NDUFV3
NOX1
NFKBIA
NISCH
CYB5R3
PPARA
PPARD

PPARG
ABCB1
PIK3CA
PIK3CB
PIK3CD
PLCH2
PPCDC
PNPO
GP1BA
KCNA1
KCNQ2
KCNQ3
POU2F1
POU2F2
PCNA
PTGS1
PTGS2
S100A7
RUSC1-AS1
PDXK
PDXP
REN
RFK
FLAD1
RPS6KA3
RPS6KA4
RYR1
SGK1
ATM
ALB
ACADSB
SIGMAR1
SCN3A
SCN2A
SLC6A3
SLC6A2
SLC6A4
SLC15A1
SLC15A2
SLC22A1
SLC22A11
SLC22A2
SLC22A3
SLC22A5
SLC22A6
SLC22A7
SLC22A8

SLCO1A2
SLCO1B1
SLCO2B1
SDHA
ALDH5A1
SLC18A2
THBD
PP35
TNFAIP6
AGTR1
UGT1A1
UGT1A10
UGT1A3
UGT1A4
UGT1A6
UGT1A7
UGT1A8
UGT1A9
UGT2B10
UGT2B15
UGT2B4
UGT2B7
VLDLR
CACNA1C
CACNA2D1
CACNA1D
CACNB2
CACNA1E
ATP1A2
CACNA1A
SCN1A
NOTCH3
MGR6
MGR5
MGR2
MGR1
MGR3
MGR12
MGR10
MGR11
TNF
MGR8
ESR1
KCNK18
LOC102724058
BRCA2
CAPN3

PPOX
PRRT2
MGR4
MGR7
MGR9
MT-TL1
ETL4
SLC1A3
POLG
COL4A1
SLC2A1
CSNK1D
ATP1A3
IL10
NOS3
SCN9A
FHL5
DBH
PRDM16
MTDH
F2
TRPM8
ALPK1
PRL
CLCN1
CPQ
ASTN2
COMT
DRD5
BDNF
IL1B
EDN1
SCN8A
EDNRB
DRD4
IL6
TSPAN2
VIP
FLNA
MEF2D
DRD1
P2RX3
PHACTR1
CRP
CNR1
CACNA1B
MAOB

RAMP1
SCN5A
HCRTR2
NOS1
TAC1
NCS1
TSPAN16
ADCYAP1
GRIA1
TPH1
GABRQ
APOH
NGF
SLC1A2
TPM3
LEP
PDE5A
KNG1
TRPV1
TWNK
POMC
ADM
TREX1
SLC20A2
CACNA1H
GSTM1
GRIN2B
PNOC
S100B
CACNB4
PPBP
SCN4A
HCRT
KCNK2
CACNA1S
KCNK5
CAV2
PEX11B
NPY
TAB2
SLC4A4
STIM1
CRH
TACR1
TRPV3
FMR1
CCK

SCN11A
PPIG
ENG
F5
SNAP25
SV2A
ARHGEF16
ASIC3
RAMP2
IAPP
OPN4
ADM2
MT-TQ
OXT
INS
H2AC18
FOS
LOC110806262
CALCR
RAMP3
SCN10A
GRIK1
TRPA1
ATP12A
ATP4A
SCT
TRPV4
GRM5
FAAH
PLG
KCNK9
VIPR1
ADCYAP1R1
P2RX2
PDYN
VIPR2
ANK1
PANX1
P2RX4
KCNK4
KCNK10
KCNS1
LAPTM4A
LIPN
MTRNR2L5
MT-ND4
ATRIP

ATRIP-TREX1
PDGFRB
MT-ND5
MPEG1
TGFBR2
MT-ND1
MT-TF
MT-TS1
PGK1
PRKCSH
MT-TK
LOC102723566
RASA1
MT-ND6
MT-TV
MT-TW
LOC108663985
PDGFB
MT-TH
TLR4
AFG3L2
SUGCT
APP
NAXE
LGI1
SLC6A1
ATP1B1
DGUOK
MT-ATP6
STARD7
MYORG
SPTAN1
GABRG2
RRM2B
LTA
ATP1B2
ACVRL1
INSR
GNAI2
GNB1
GNG2
MMP2
PRICKLE2
APOE
CXCL12
HLA-DQB1
C1QTNF3-AMACR

MTR
MT-CO3
TNFRSF1A
SMARCB1
OPA1
SPAST
STXBP1
SLC25A4
POLG2
SOD2
RELN
HLA-B
NPFF
PMM2
FGFR3
COL4A2
MYD88
GABRA5
SCN3B
ATP1A4
AQP1
NF1
RNASEH1
SLC1A7
MT-TT
MT-TI
MT-TP
SMAD3
MT-CO2
MT-TS2
RAB3GAP1
PRICKLE2-AS1
EA8
EA3
EA7
ORAI1
TGFB1
HLA-DRB1
MT-TL2
MT-TN
MT-TE
MT-TA
COL3A1
IL23R
DEPDC5
ADA2
SQOR

LRP1
SLC5A1
SLC34A1
SPTB
CTU2
SMAD4
MVK
MEFV
SPOP
FBN1
NAV1
BMPR1A
NLRP3
FAS
MSH6
MLH1
MSH2
PMS2
EPCAM
ADAMTSL1
STAT4
NOS2
EPHB4
ATP13A1
NOP53
IL1A
DNM1L
MLH3
SH2B1
FAN1
ZFTA
MYH7
ITGB3
NRP1
GNB3
MTRR
PGR
NFIX
TWIST1
CTLA4
CCNH
PLOD1
FGFR2
GRIN2A
KRAS
RELA
GATA2

GP9
CCR1
SEMA4A
AIP
C4A
ERAP1
MLX
SLC6A19
RPS20
ACSF3
IL12B
NOP56
IL12A
GP1BB
GDF2
NDP
PMS1
SRPX2
GPR101
KLRC4
UBAC2
IL12A-AS1
GABRE
IL13
ICAM1
SELE
STX1A
NPC1
FKRP
IL4
CD40LG
MMP3
KCNN3
IL1RN
RNR2
AGT
NOTCH4
MT-CYB
HTRA1
GRIA3
SERPINE1
SELP
PON1
SERPINC1
FH
HHT4
VCAM1

F8
CYP19A1
MTHFD1
ADIPOQ
PDE2A
F7
TPH2
MMP9
AQP4
BRAF
CSF2
C3
HLA-DQA1
GNAQ
TANGO2
PF4
SMARCAL1
CNTNAP2
TYMS
KCNE2
SERPINA3
VDR
NOD2
YARS2
ITGA2
IL9
CASZ1
CCL2
CXCL8
B9D2
STAT6
HP
ENO2
ATRX
MEN1
PITRM1
PITRM1-AS1
LOC101927870
PNKD2
ESR2
TH
GFRA1
STIN2-VNTR
PLAUR
VEGFA
POLR1C
RBFOX3

GSTP1
RYR2
GAL
CCL11
FANCI
PACS1
MYH6
IGLL1
AGBL1
CCL5
SCN1B
NBEA
PSTPIP1
NPPA
ADH1B
ADH1C
CCR3
CCR5
LPA
CCR2
SMARCA4
GC
AHNAK2
SOD1
HFE
PNKD
NNMT
LTC4S
KCNB2
KCNS3
TBXAS1
TSC1
F2R
XPR1
FBN2
COL1A1
TLR5
CSTB
EIF2B2
ARID1B
PCDH19
IER3IP1
OPA1-AS1
BMIQ16
DEL17Q11.2
SHBG
MT-TC

ATP2C2
KCNK12
FSHR
FASLG
NRIP1
KCNN1
MMP1
JAG1
DLL3
HEY2
DDC
ABCC9
RHAG
EFHC1
SST
VWC2
CACNA1F
GNAS
RLBP1
OFD1
CBY1
EDAR
AHI1
INPP5E
JAM2
CEP290
MKS1
C2CD3
IFT172
CC2D2A
TCTN2
RPGRIP1L
TCTN1
TMEM67
B9D1
TMEM216
TMEM237
KIAA0586
KIAA0753
C12orf29
TMEM218
CPLANE1
TOGARAM1
COL4A2-AS2
COL4A2-AS1
MMVP1
ECT

CELIAC2
CELIAC10
CELIAC11
CELIAC12
BFIS1
CELIAC13
CELIAC5
CELIAC6
CELIAC7
CELIAC8
CELIAC9
UTS2
ITGAL
ADA
IL1RAPL2
HPSE2
IRAG1
GSTT1
EIF2B3
CBS
IL4R
SCNN1A
C5
FGB
IL5RA
TCF7
CD14
ADD1
SCGB1A1
CDC42EP2
METTL5
SCPEP1
RAB40B
ROPN1L
SYTL1
AHDC1
RILPL1
ARMC3
ARHGAP36
PLEKHA2
OTUD1
IL5
TYMP
IFIH1
PRKG1
CD40
IGHE

GPT
MMP17
DLG2
INA
MARCHF4
IGF1
PLCE1
RET
FZD4
GRIN1
ASS1
PLCB1
RB1
SLC25A13
WWOX
ASAH1
MAF
STRADA
DKK1
EIF2B4
EFEMP1
CHD2
TSPAN12
SCARB2
AARS2
COL11A1
CPA6
EPM2A
EIF2B1
EIF2B5
KCNT1
PRICKLE1
FKBP14
ANKLE2
PRSS23
TBC1D24
RBFOX1
SNIP1
KLHDC8B
SZT2
CLTRN
NDP-AS1
SCN1A-AS1
SEPT5-GP1BB
SIK1B
ARFGEF1-DT
FKBP14-AS1

LOC109504727
EKD2
HHT3
WM2
PTX3
BACE1
CLIC5
IRX4
MEPE
RNF214
SV2C
TFAM
KCNJ10
HTR5A
KCNJ9
SRR
KCNMA1
SHMT1
GSR
IL18
AR
TNFRSF21
MEP1A
SLC25A27
NR3C1
ACTN4
NEDD4L
FOXP1
EYA1
MMP16
PTPRD
INHBA
STK10
CDH13
GFRA2
NSD1
WASL
SUV39H2
CDH4
CNNM2
SMYD3
KANK1
SORBS1
TBC1D7
ATF7
ADARB2
KSR2

CTNNA3
RSU1
FARP1
PHF20
ZDHHC6
LIMCH1
FRMD4A
SLC35D2
ARL15
LEPROTL1
MRPL37
RABGAP1L
SNX24
HAUS1
AJAP1
COX7B2
CTIF
VSTM4
WAPL
BPIFC
TRMT9B
CDKN2B-AS1
INHBA-AS1
NR2F2-AS1
OTUD6B-AS1
LOC349160
LOC102723445
SPTBN2
ATXN7
S100A12
DIDO1
OGG1
XRCC3
GATA4
CACNA1G
CD79A
TNFSF10
LEPQTL1
DDIT3
CD70
CFLAR
GABRA3
CYP20A1
SAMHD1
HLA-DPB1
TNFRSF1B
AHSG

MAP2K2
DSP
MAPK3
HLA-A
F3
MIR30A
NOTCH1
EGF
NOTCH2
RBPJ
DLL4
DLL1
TIMP3
KCNC3
HEY1
DARS2
JAG2
HEYL
SRRT
HTR3D
DDX58
IRF3
EXO1
CTNNB1
PRKCG
ATXN1
MIR9-1
PAFAH1B1
CACNA1I
EGFR
PSMC4
TBK1
ISG15
ADAR
DNASE1L3
RNASEH2A
MAVS
RNASEH2C
IFIT1
RNASEH2B
STING1
ARMC8
ZNF621
KLHDC7A
CGAS
IL2RA
VWF

HLA-C
HLA-G
HLA-E
HCRTR1
LOC110973015
SLC1A1
MS4A2
DNA2
GABRR1
CAT
ALDH2
DAO
GABRA4
ANK3
AGTR2
COQ8A
ERCC2
APEX1
MEIS1
RNF138
CLDN5
ESM1
CD160
VWA8
NAMPT
HEPH
MDFI
FLJ16779
SUCLA2
SUCLG1
SURF1
MPV17
SSBP1
MGME1
ROCK2
PRDX2
ACSL5
AOC1
NRXN2
TXNDC9
LOC111365141
SLC1A4
GRIA2
GRIA4
ZEB2
PRNP
ANKK1

IFNG
TF
SLC5A6
SYNE1
FRAXA
LOC101927066
IL2
SPG7
PGM1
GNAI1
BDKRB2
ACP1
CDKN3
NTS
NF2
ACADVL
PPP2R2B
PLP1
MSX2
HSD17B4
SCP2
ACOX1
AKR1D1
SLC1A6
ACOX2
PEX6
ACOX3
HACL1
SLC45A2
ACOT8
PEX16
PXMP4
TMEM176A
CDKL5
PSAT1
DBT
RETN
EPN1
LOC101927558
IRF4
ELANE
TGFBR1
TGFB2
ACTA2
FN1
TLR2
CD4

CCR6
COL5A1
APTX
SYP
ATXN2
COQ2
SIL1
ICOSLG
MRRF
NFKB1
ITGB2
ITGA4
TPT1
LOC111258525
SLC5A3
NFKBIB
CACNL1A4
ESR
FHM2
FHM3
GEFSP2
MA
MFDA
MGOA
TNFA
TRESK
ESTRR
MHP2
SCA6
SMEI
TRIK
DEE42
FEB3A
MGR13
DRVT
DEE6A
DEE6B
CGRPR
COX
ALOX
PAC1R
NaC
MAO-B
GlyR
mGluR2
CYSLTR1
CALC

PTGER4
MOP
5HT2R
5HT1R
D2R
SERT
NET
