# Supplementary material for: Molecular Mechanism of the Saposhnikovia divaricata–Angelica dahurica Herb Pair in Migraine Therapy Based on Network Pharmacology and Molecular Docking
Source: Evid Based Complement Alternat Med. 2022 Nov 26;2022:1994575. doi: 10.1155/2022/1994575 (PMC9722292; doi:10.1155/2022/1994575)
Supplement: Supplementary Materials — Table S1: 704 targets of SAHP. Table S2: 1086 targets of migraine. Table S3: 183 common targets of SAHP and migraine. Table S4: the result of GO functional enrichment analysis. Table S5: the result of KEGG pathway enrichment analysis. [file 1994575.f1.zip › Table S3.183 common targets of SAHP and migraine.pdf]

Target
PDE5A
CYSLTR1
ACE
HAO2
HMGCR
MMP16
MMP9
MMP1
CASP3
TYMS
CASP1
GSR
AR
NOS2
PTGS2
F2
PIK3CA
RET
KCNH2
BRAF
ADORA2A
ADORA3
PIK3CD
PRKDC
PIK3CB
CXCR1
EGFR
EPHB4
ALPL
CFTR
ADORA1
MMP3
ABCC1
NTRK1
DRD1
DRD2
EDNRB
EDNRA
HDAC2
KCNMA1
F7
ESR1
NOS3
SCN5A
CNR1

ELANE
SCN2A
HCRTR2
SCN10A
P2RX3
SCN9A
PDE4B
GRM5
KCNK9
CHRM2
CHRM1
CHRM3
HTR1A
HTR7
HTR6
HCRTR1
TRPA1
CCND1
IKBKB
DRD4
FAAH
NOS1
TRPV1
CYP19A1
CHRM4
PRKCG
CCNA2
PTGS1
BACE1
MAOA
MAOB
ADORA2B
CYP1A2
CA2
CA1
APP
HRH3
HRH4
PDE2A
ASAH1
ROCK2
TBXAS1
SLC5A1
PDGFRB
PPARG
GRIN1
ICAM1

SELE
ADRB2
ADRA1A
ADRA1B
ESR2
GABRA1
OPRM1
CSNK1D
MAPK15
TBK1
SHBG
CYP2C19
SLC6A2
SLC6A4
VDR
PPARD
NR3C1
UGT2B7
PGR
PPARA
OPRD1
ABCB1
NTRK2
CYP1A1
ABCG2
CYP1B1
CA4
MMP2
INSR
CA3
APEX1
AKR1C1
BCL2
TP53
FN1
IL6
CXCL8
CCL2
RELA
TNF
SIGMAR1
GABRA5
RPS6KA4
STK10
HTR2A
ADRA2A
ADRB1

SLC6A3
TLR4
CCR1
CCR2
NQO1
HTR1B
GABRG2
TNFRSF1A
MEN1
TRPV4
SLC2A1
AGTR1
NFKBIA
ALDH2
IL2
ADA
HTR2B
ADRA2C
NLRP3
TGFBR1
ADRA1D
FGFR2
GRIN2A
GRIA2
ITGAL
BDKRB2
CACNA1C
CHRM5
DHODH
ACP1
OPRK1
ATM
F2R
GRIN2B
CALCRL
HTR2C
NAMPT
PON1
TGFB1
DRD3
RPS6KA3
ADRA2B
PPOX
PTGER4
