# Supplementary material for: Molecular Mechanism of the Saposhnikovia divaricata–Angelica dahurica Herb Pair in Migraine Therapy Based on Network Pharmacology and Molecular Docking
Source: Evid Based Complement Alternat Med. 2022 Nov 26;2022:1994575. doi: 10.1155/2022/1994575 (PMC9722292; doi:10.1155/2022/1994575)
Supplement: Supplementary Materials — Table S1: 704 targets of SAHP. Table S2: 1086 targets of migraine. Table S3: 183 common targets of SAHP and migraine. Table S4: the result of GO functional enrichment analysis. Table S5: the result of KEGG pathway enrichment analysis. [file 1994575.f1.zip › Table S4.the result of GO functional enrichment analysis.pdf]

_MEMBER_MyList	_LogP_MyList	GO	_PATTERN_	_RANK_	GiniIndex	Category	CategoryID	Description	LogP	Enrichment	Z-score	#TotalGeneInLibrary	#GeneInGO	#GeneInHitList	#GeneInGOAndHitList	%InGO	STDV %InGO	GeneID	Hits	Log(q-value)	EvidenceCutoff	GROUP_ID	FirstInGroupByEnrichment	FirstInGroupByLogP	BestLogPInGroup	BestEnrichmentInGroup
1	-59.7472	GO:0003013	M1	1	0	GO Biological Processes	19	circulatory system process	-59.7472	17.41	31.62475	30242	598	183	63	34.42623	3.512241	100|134|135|140|146|147|148|150|151|152|153|154|185|367|624|775|1129|1131|1268|1636|1812|1813|1814|1909|1910|1956|2149|3156|3350|3351|3356|3357|3363|3383|3558|3643|3757|3778|4363|4842|4843|4846|5138|5142|5243|5290|5465|5467|5468|5742|5743|6331|6336|6513|6523|6532|6916|7040|7442|9429|9475|10135|59341	ADA|ADORA1|ADORA2A|ADORA3|ADRA1D|ADRA1B|ADRA1A|ADRA2A|ADRA2B|ADRA2C|ADRB1|ADRB2|AGTR1|AR|BDKRB2|CACNA1C|CHRM2|CHRM3|CNR1|ACE|DRD1|DRD2|DRD3|EDNRA|EDNRB|EGFR|F2R|HMGCR|HTR1A|HTR1B|HTR2A|HTR2B|HTR7|ICAM1|IL2|INSR|KCNH2|KCNMA1|ABCC1|NOS1|NOS2|NOS3|PDE2A|PDE4B|ABCB1|PIK3CA|PPARA|PPARD|PPARG|PTGS1|PTGS2|SCN5A|SCN10A|SLC2A1|SLC5A1|SLC6A4|TBXAS1|TGFB1|TRPV1|ABCG2|ROCK2|NAMPT|TRPV4	-55.5512	0	1	1	1	-59.7472	51.93786105
1	-55.0185	GO:0008015	M1	1	0	GO Biological Processes	19	blood circulation	-55.0185	18.36187	30.94578	30242	513	183	57	31.14754	3.423309	100|134|135|140|146|147|148|150|151|152|153|154|185|367|624|775|1129|1131|1268|1636|1812|1813|1814|1909|1910|1956|2149|3156|3350|3351|3356|3357|3363|3383|3558|3757|3778|4842|4843|4846|5138|5142|5290|5465|5467|5468|5742|5743|6331|6336|6532|6916|7040|7442|9475|10135|59341	ADA|ADORA1|ADORA2A|ADORA3|ADRA1D|ADRA1B|ADRA1A|ADRA2A|ADRA2B|ADRA2C|ADRB1|ADRB2|AGTR1|AR|BDKRB2|CACNA1C|CHRM2|CHRM3|CNR1|ACE|DRD1|DRD2|DRD3|EDNRA|EDNRB|EGFR|F2R|HMGCR|HTR1A|HTR1B|HTR2A|HTR2B|HTR7|ICAM1|IL2|KCNH2|KCNMA1|NOS1|NOS2|NOS3|PDE2A|PDE4B|PIK3CA|PPARA|PPARD|PPARG|PTGS1|PTGS2|SCN5A|SCN10A|SLC6A4|TBXAS1|TGFB1|TRPV1|ROCK2|NAMPT|TRPV4	-51.1236	0	1	0	0	-59.7472	51.93786105
1	-48.4362	GO:0003018	M1	1	0	GO Biological Processes	19	vascular process in circulatory system	-48.4362	27.01918	33.06734	30242	263	183	43	23.49727	3.134163	134|135|146|147|148|150|151|152|153|154|185|	ADORA1|ADORA2A|ADRA1D|ADRA1B|A	-44.7173	0	1	0	0	-59.7472	51.93786105

																		624|1131|1636|1812|1909|1910|1956|2149|3156|3350|3351|3356|3357|3363|3383|3643|3778|4363|4842|4846|5138|5243|5467|5743|6513|6523|6532|6916|7040|9429|9475|59341	DRA1A|ADRA2A|ADRA2B|ADRA2C|ADRB1|ADRB2|AGTR1|BDKRB2|CHRM3|ACE|DRD1|EDNRA|EDNRB|EGFR|F2R|HMGCR|HTR1A|HTR1B|HTR2A|HTR2B|HTR7|ICAM1|INSR|KCNMA1|ABCC1|NOS1|NOS3|PDE2A|ABCB1|PPARD|PTGS2|SLC2A1|SLC5A1|SLC6A4|TBXAS1|TGFB1|ABCG2|ROCK2|TRPV4							
1	-44.3344	GO:0035296	M1	1	0	GO Biological Processes	19	regulation of tube diameter	-44.3344	39.84916	36.07756	30242	141	183	34	18.57923	2.87512	134|135|146|147|148|150|151|152|153|154|185|624|1131|1636|1812|1909|1910|1956|2149|3156|3350|3351|3356|3357|3363|3383|3778|4842|4846|5467|5743|6532|6916|9475	ADORA1|ADORA2A|ADRA1D|ADRA1B|ADRA1A|ADRA2A|ADRA2B|ADRA2C|ADRB1|ADRB2|AGTR1|BDKRB2|CHRM3|ACE|DRD1|EDNRA|EDNRB|EGFR|F2R|HMGCR|HTR1A|HTR1B|HTR2A|HTR2B|HTR7|ICAM1|KCNMA1|NOS1|NOS3|PPARD|PTGS2|SLC6A4|TBXAS1|ROCK2	-40.9835	0	1	0	0	-59.7472	51.93786105
1	-44.3344	GO:0097746	M1	1	0	GO Biological Processes	19	blood vessel diameter maintenance	-44.3344	39.84916	36.07756	30242	141	183	34	18.57923	2.87512	134|135|146|147|148|150|151|152|153|154|185|624|1131|1636|1812|1909|1910|1956|2149|3156|3350|3351|3356|3357|3363|3383|3778|4842|4846|5467|5743|6532|6916|9475	ADORA1|ADORA2A|ADRA1D|ADRA1B|ADRA1A|ADRA2A|ADRA2B|ADRA2C|ADRB1|ADRB2|AGTR1|BDKRB2|CHRM3|ACE|DRD1|EDNRA|EDNRB|EGFR|F2R|HMGCR|HTR1A|HTR1B|HTR2A|HTR2B|HTR7|ICAM1|KCNMA1|NOS1|NOS3|PPARD|PTGS2|SLC6A4|TBXAS1|ROCK2	-40.9835	0	1	0	0	-59.7472	51.93786105
1	-44.2176	GO:0035150	M1	1	0	GO Biological Processes	19	regulation of tube size	-44.2176	39.56854	35.94433	30242	142	183	34	18.57923	2.87512	134|135|146|147|148|150|151|152|153|154|185|624|1131|1636|1812|1909|1910|1956|2149|3156|3350|3351|3356|3357|3363|3383|3778|4842|4846|5467|5743|6532|6916|9475	ADORA1|ADORA2A|ADRA1D|ADRA1B|ADRA1A|ADRA2A|ADRA2B|ADRA2C|ADRB1|ADRB2|AGTR1|BDKRB2|CHRM3|ACE|DRD1|EDNRA|EDNRB|EGFR|F2R|HMGCR|	-40.9759	0	1	0	0	-59.7472	51.93786105

																			HTR1A|HTR1B|HTR2A|HTR2B|HTR7|ICAM1|KCNMA1|NOS1|NOS3|PPARD|PTGS2|SLC6A4|TBXAS1|ROCK2							
1	-42.3256	GO:0044057	M1	1	0	GO Biological Processes	19	regulation of system process	-42.3256	14.22176	25.1085	30242	581	183	50	27.3224	3.29408	100|134|135|140|146|147|148|150|151|152|153|154|185|351|624|775|1129|1131|1636|1813|1814|1910|1956|2149|2902|3156|3350|3356|3358|3383|3558|3757|3778|4842|4846|4985|4986|4988|5024|5142|5465|5743|6331|6336|6916|7132|7442|9475|10203|59341	ADA|ADORA1|ADORA2A|ADORA3|ADRA1D|ADRA1B|ADRA1A|ADRA2A|ADRA2B|ADRA2C|ADRB1|ADRB2|AGTR1|APP|BDKRB2|CACNA1C|CHRM2|CHRM3|ACE|DRD2|DRD3|EDNRB|EGFR|F2R|GRIN1|HMGCR|HTR1A|HTR2A|HTR2C|ICAM1|IL2|KCNH2|KCNMA1|NOS1|NOS3|OPRD1|OPRK1|OPRM1|P2RX3|PDE4B|PPARA|PTGS2|SCN5A|SCN10A|TBXAS1|TNFRSF1A|TRPV1|ROCK2|CALCRL|TRPV4	-39.2436	0	1	0	0	-59.7472	51.93786105
1	-33.2414	GO:0042310	M1	1	0	GO Biological Processes	19	vasoconstriction	-33.2414	47.21624	33.09553	30242	84	183	24	13.11475	2.495327	146|147|148|150|151|152|185|624|1131|1636|1909|1910|1956|2149|3156|3350|3351|3356|3357|3363|3383|5743|6532|6916	ADRA1D|ADRA1B|ADRA1A|ADRA2A|ADRA2B|ADRA2C|AGTR1|BDKRB2|CHRM3|ACE|EDNRA|EDNRB|EGFR|F2R|HMGCR|HTR1A|HTR1B|HTR2A|HTR2B|HTR7|ICAM1|PTGS2|SLC6A4|TBXAS1	-30.5368	0	1	0	0	-59.7472	51.93786105
1	-31.8783	GO:1903522	M1	1	0	GO Biological Processes	19	regulation of blood circulation	-31.8783	20.57673	24.59184	30242	257	183	32	17.48634	2.807934	100|134|140|146|147|148|150|151|152|153|185|624|775|1129|1131|1636|1813|1956|2149|3156|3350|3356|3383|3558|3757|4842|5142|5743|6331|6336|6916|7442	ADA|ADORA1|ADORA3|ADRA1D|ADRA1B|ADRA1A|ADRA2A|ADRA2B|ADRA2C|ADRB1|AGTR1|BDKRB2|CACNA1C|CHRM2|CHRM3|ACE|DRD2|EGFR|F2R|HMGCR|HTR1A|HTR2A|ICAM1|IL2|KCNH2|NOS1|PDE4B|PTGS2|SCN5A|SCN10A|TBXAS1|TRPV1	-29.2138	0	1	0	0	-59.7472	51.93786105
1	-29.2071	GO:0090066	M1	1	0	GO Biological Processes	19	regulation of anatomical structure size	-29.2071	12.09973	19.90151	30242	519	183	38	20.76503	2.998466	134|135|146|147|148|150|151|152|153|154|185|624|1131|1636|1812|19	ADORA1|ADORA2A|ADRA1D|ADRA1B|ADRA1A|ADRA2A|AD	-26.6021	0	1	0	0	-59.7472	51.93786105

																		09|1910|1956|2149|2335|3156|3350|3351|3356|3357|3363|3383|3778|4842|4846|5290|5467|5743|5979|6532|6916|9475|59341	RA2B|ADRA2C|ADRB1|ADRB2|AGTR1|BDKRB2|CHRM3|ACE|DRD1|EDNRA|EDNRB|EGFR|F2R|FN1|HMGCR|HTR1A|HTR1B|HTR2A|HTR2B|HTR7|ICAM1|KCNMA1|NOS1|NOS3|PIK3CA|PPARD|PTGS2|RET|SLC6A4|TBXAS1|ROCK2|TRPV4							
1	-28.9171	GO:0003012	M1	1	0	GO Biological Processes	19	muscle system process	-28.9171	13.16205	20.32718	30242	452	183	36	19.67213	2.938551	100|134|147|148|150|151|152|154|624|775|1129|1131|1813|1909|1910|2149|3066|3356|3357|3363|3757|3778|4842|4846|5024|5142|5290|5465|5743|6331|6336|7132|7442|9475|10203|59341	ADA|ADORA1|ADRA1B|ADRA1A|ADRA2A|ADRA2B|ADRA2C|ADRB2|BDKRB2|CACNA1C|CHRM2|CHRM3|DRD2|EDNRA|EDNRB|F2R|HDAC2|HTR2A|HTR2B|HTR7|KCNH2|KCNMA1|NOS1|NOS3|P2RX3|PDE4B|PIK3CA|PPARA|PTGS2|SCN5A|SCN10A|TNFRSF1A|TRPV1|ROCK2|CALCRL|TRPV4	-26.3339	0	1	0	0	-59.7472	51.93786105
1	-28.3685	GO:0006939	M1	1	0	GO Biological Processes	19	smooth muscle contraction	-28.3685	34.5537	27.50801	30242	110	183	23	12.56831	2.450458	100|134|148|150|151|152|154|624|1129|1131|1813|1909|1910|2149|3356|3357|3363|3778|5024|5743|7442|9475|10203	ADA|ADORA1|ADRA1A|ADRA2A|ADRA2B|ADRA2C|ADRB2|BDKRB2|CHRM2|CHRM3|DRD2|EDNRA|EDNRB|F2R|HTR2A|HTR2B|HTR7|KCNMA1|P2RX3|PTGS2|TRPV1|ROCK2|CALCRL	-25.7957	0	1	0	0	-59.7472	51.93786105
1	-27.6628	GO:0006936	M1	1	0	GO Biological Processes	19	muscle contraction	-27.6628	15.23982	20.81644	30242	347	183	32	17.48634	2.807934	100|134|147|148|150|151|152|154|624|775|1129|1131|1813|1909|1910|2149|3356|3357|3363|3757|3778|4842|5024|5142|5290|5743|6331|6336|7442|9475|10203|59341	ADA|ADORA1|ADRA1B|ADRA1A|ADRA2A|ADRA2B|ADRA2C|ADRB2|BDKRB2|CACNA1C|CHRM2|CHRM3|DRD2|EDNRA|EDNRB|F2R|HTR2A|HTR2B|HTR7|KCNH2|KCNMA1|NOS1|P2RX3|PDE4B|PIK3CA|PTGS2|SCN5A|SCN10A|TRPV1|ROCK2|CALCRL|TRPV4	-25.1295	0	1	0	0	-59.7472	51.93786105
1	-24.5724	GO:0019229	M1	1	0	GO Biological Processes	19	regulation of vasoconstriction	-24.5724	45.07004	27.9654	30242	66	183	18	9.836066	2.201413	146|147|148|150|151|152|185|624|1131|1636|1956|2149|3156|3350|335	ADRA1D|ADRA1B|ADRA1A|ADRA2A|ADRA2B|ADRA2C|AGT	-22.1758	0	1	0	0	-59.7472	51.93786105

																		6|3383|5743|6916	R1|BDKRB2|CHRM3|ACE|EGFR|F2R|HMGCR|HTR1A|HTR2A|ICAM1|PTGS2|TBXAS1							
1	-21.0699	GO:0090257	M1	1	0	GO Biological Processes	19	regulation of muscle system process	-21.0699	15.73875	18.33196	30242	252	183	24	13.11475	2.495327	100|134|147|148|150|151|152|154|775|1129|1131|2149|3778|4842|4846|5142|5465|5743|6331|6336|7132|9475|10203|59341	ADA|ADORA1|ADRA1B|ADRA1A|ADRA2A|ADRA2B|ADRA2C|ADRB2|CACNA1C|CHRM2|CHRM3|F2R|KCNMA1|NOS1|NOS3|PDE4B|PPARA|PTGS2|SCN5A|SCN10A|TNFRSF1A|ROCK2|CALCRL|TRPV4	-18.8134	0	1	0	0	-59.7472	51.93786105
1	-19.4986	GO:0006937	M1	1	0	GO Biological Processes	19	regulation of muscle contraction	-19.4986	19.55702	18.87555	30242	169	183	20	10.92896	2.306387	100|134|147|148|150|151|152|154|775|1129|1131|2149|3778|4842|5142|5743|6331|6336|10203|59341	ADA|ADORA1|ADRA1B|ADRA1A|ADRA2A|ADRA2B|ADRA2C|ADRB2|CACNA1C|CHRM2|CHRM3|F2R|KCNMA1|NOS1|PDE4B|PTGS2|SCN5A|SCN10A|CALCRL|TRPV4	-17.2711	0	1	0	0	-59.7472	51.93786105
1	-16.0219	GO:0006940	M1	1	0	GO Biological Processes	19	regulation of smooth muscle contraction	-16.0219	33.56779	20.35024	30242	64	183	13	7.103825	1.898976	100|134|148|150|151|152|154|1129|1131|2149|3778|5743|10203	ADA|ADORA1|ADRA1A|ADRA2A|ADRA2B|ADRA2C|ADRB2|CHRM2|CHRM3|F2R|KCNMA1|PTGS2|CALCRL	-13.9813	0	1	0	0	-59.7472	51.93786105
1	-15.9662	GO:0045907	M1	1	0	GO Biological Processes	19	positive regulation of vasoconstriction	-15.9662	51.93786	23.5265	30242	35	183	11	6.010929	1.757049	146|147|148|152|1956|2149|3156|3356|3383|5743|6916	ADRA1D|ADRA1B|ADRA1A|ADRA2C|EGFR|F2R|HMGCR|HTR2A|ICAM1|PTGS2|TBXAS1	-13.9286	0	1	0	0	-59.7472	51.93786105
1	-48.2064	GO:0071417	M1	1	0	GO Biological Processes	19	cellular response to organonitrogen compound	-48.2064	14.5053	26.90146	30242	638	183	56	30.60109	3.406585	154|185|328|351|760|836|890|1080|1128|1129|1131|1132|1133|1812|1813|1814|1815|2554|2566|2902|2903|2915|3066|3350|3351|3356|3357|3358|3362|3363|3383|3643|4221|4313|4363|4914|4915|4988|5024|5138|5142|5290|5468|5591|5743|5970|6532|7099|7124|7157|7442|9475|10135|11255|23621|59340	ADRB2|AGTR1|APEX1|APP|CA2|CASP3|CCNA2|CFTR|CHRM1|CHRM2|CHRM3|CHRM4|CHRM5|DRD1|DRD2|DRD3|DRD4|GABRA1|GABRG2|GRIN1|GRIN2A|GRM5|HDAC2|HTR1A|HTR1B|HTR2A|HTR2B|HTR2C|HTR6|HTR7|ICAM1|INSR|MEN1|MMP2|ABCC1|NTRK1|NTRK2|OPRM1|P2RX3|PDE2A|PDE4B|PIK3CA|PPARG|PRKDC|PTGS2|RELA|SLC6A4|TLR4|TNF|TP53|TRPV1|ROCK2|NA	-44.6125	0	2	1	1	-48.2064	165.2568306

<table>
<tr><td></td><td></td><td></td><td></td><td></td><td></td><td></td><td></td><td></td><td></td><td></td><td></td><td></td><td></td><td></td><td></td><td></td><td></td><td></td><td>MPT|HRH3|BACE1|HRH4</td><td></td><td></td><td></td><td></td><td></td><td></td><td></td></tr>
<tr><td>1</td><td>-47.5252</td><td>GO:1901699</td><td>M1</td><td>1</td><td>0</td><td>GO Biological Processes</td><td>19</td><td>cellular response to nitrogen compound</td><td>-47.5252</td><td>13.59255</td><td>26.16648</td><td>30242</td><td>693</td><td>183</td><td>57</td><td>31.14754</td><td>3.423309</td><td>154|185|328|351|760|836|890|1080|1128|1129|1131|1132|1133|1812|1813|1814|1815|2554|2566|2902|2903|2915|3066|3350|3351|3356|3357|3358|3362|3363|3383|3643|4221|4313|4314|4363|4914|4915|4988|5024|5138|5142|5290|5468|5591|5743|5970|6532|7099|7124|7157|7442|9475|10135|11255|23621|59340</td><td>ADRB2|AGTR1|APEX1|APP|CA2|CASP3|CCNA2|CFTR|CHRM1|CHRM2|CHRM3|CHRM4|CHRM5|DRD1|DRD2|DRD3|DRD4|GABRA1|GABRG2|GRIN1|GRIN2A|GRM5|HDAC2|HTR1A|HTR1B|HTR2A|HTR2B|HTR2C|HTR6|HTR7|ICAM1|INSR|MEN1|MMP2|MMP3|ABCC1|NTRK1|NTRK2|OPRM1|P2RX3|PDE2A|PDE4B|PIK3CA|PPARG|PRKDC|PTGS2|RELA|SLC6A4|TLR4|TNF|TP53|TRPV1|ROCK2|NAMPT|HRH3|BACE1|HRH4</td><td>-44.0282</td><td>0</td><td>2</td><td>0</td><td>0</td><td>-48.2064</td><td>165.2568306</td></tr>
<tr><td>1</td><td>-42.1408</td><td>GO:0071407</td><td>M1</td><td>1</td><td>0</td><td>GO Biological Processes</td><td>19</td><td>cellular response to organic cyclic compound</td><td>-42.1408</td><td>14.10041</td><td>24.98698</td><td>30242</td><td>586</td><td>183</td><td>50</td><td>27.3224</td><td>3.29408</td><td>249|328|351|367|836|890|1080|1128|1129|1131|1132|1133|1543|1545|1812|1813|1814|1815|1956|2099|2100|2554|2566|2908|3066|3350|3351|3356|3357|3358|3362|3363|3383|4988|5024|5138|5142|5241|5465|5467|5743|6347|6532|7040|7124|7421|7442|9475|11255|59340</td><td>ALPL|APEX1|APP|AR|CASP3|CCNA2|CFTR|CHRM1|CHRM2|CHRM3|CHRM4|CHRM5|CYP1A1|CYP1B1|DRD1|DRD2|DRD3|DRD4|EGFR|ESR1|ESR2|GABRA1|GABRG2|NR3C1|HDAC2|HTR1A|HTR1B|HTR2A|HTR2B|HTR2C|HTR6|HTR7|ICAM1|OPRM1|P2RX3|PDE2A|PDE4B|PGR|PPARA|PPARD|PTGS2|CCL2|SLC6A4|TGFB1|TNF|VDR|TRPV1|ROCK2|HRH3|HRH4</td><td>-39.091</td><td>0</td><td>2</td><td>0</td><td>0</td><td>-48.2064</td><td>165.2568306</td></tr>
<tr><td>1</td><td>-34.7964</td><td>GO:0007188</td><td>M1</td><td>1</td><td>0</td><td>GO Biological Processes</td><td>19</td><td>adenylate cyclase-modulating G protein-coupled receptor signaling pathway</td><td>-34.7964</td><td>23.40547</td><td>26.78815</td><td>30242</td><td>233</td><td>183</td><td>33</td><td>18.03279</td><td>2.842012</td><td>134|135|146|147|148|150|151|152|153|154|1128|1129|1131|1132|1133|1268|1812|1813|1814|1815|1909|2915|3350|3351|4985|4986|4988|5138|5142|5734|10203|11255|59340</td><td>ADORA1|ADORA2A|ADRA1D|ADRA1B|ADRA1A|ADRA2A|ADRA2B|ADRA2C|ADRB1|ADRB2|CHRM1|CHRM2|CHRM3|CHRM4|CHRM5|CNR1|DRD1|DRD2|DRD3|DRD4|EDNRA|GRM5|HTR1A|HTR1B|OPRD1|OPRK1|OPRM1|PDE2A|PDE4B|PTGER4|CALC</td><td>-32.0318</td><td>0</td><td>2</td><td>0</td><td>0</td><td>-48.2064</td><td>165.2568306</td></tr>
</table>

<table>
<tr><td></td><td></td><td></td><td></td><td></td><td></td><td></td><td></td><td></td><td></td><td></td><td></td><td></td><td></td><td></td><td></td><td></td><td></td><td></td><td>RL|HRH3|HRH4</td><td></td><td></td><td></td><td></td><td></td><td></td><td></td></tr>
<tr><td>1</td><td>-32.0337</td><td>GO:0007187</td><td>M1</td><td>1</td><td>0</td><td>GO Biological Processes</td><td>19</td><td>G protein-coupled receptor signaling pathway, coupled to cyclic nucleotide second messenger</td><td>-32.0337</td><td>54.26344</td><td>34.05488</td><td>30242</td><td>67</td><td>183</td><td>22</td><td>12.02186</td><td>2.404073</td><td>1128|1129|1131|1132|1133|1230|1268|1812|1815|3350|3351|3356|3357|3358|3362|3363|4985|4988|6347|10203|11255|59340</td><td>CHRM1|CHRM2|CHRM3|CHRM4|CHRM5|CCR1|CNR1|DRD1|DRD4|HTR1A|HTR1B|HTR2A|HTR2B|HTR2C|HTR6|HTR7|OPRD1|OPRM1|CCL2|CALCRL|HRH3|HRH4</td><td>-29.3563</td><td>0</td><td>2</td><td>0</td><td>0</td><td>-48.2064</td><td>165.2568306</td></tr>
<tr><td>1</td><td>-26.6614</td><td>GO:0007193</td><td>M1</td><td>1</td><td>0</td><td>GO Biological Processes</td><td>19</td><td>adenylate cyclase-inhibiting G protein-coupled receptor signaling pathway</td><td>-26.6614</td><td>42.37355</td><td>28.5471</td><td>30242</td><td>78</td><td>183</td><td>20</td><td>10.92896</td><td>2.306387</td><td>134|150|1128|1129|1131|1132|1133|1813|1814|1815|1909|2915|3350|3351|4985|4986|4988|5138|11255|59340</td><td>ADORA1|ADRA2A|CHRM1|CHRM2|CHRM3|CHRM4|CHRM5|DRD2|DRD3|DRD4|EDNRA|GRM5|HTR1A|HTR1B|OPRD1|OPRK1|OPRM1|PDE2A|HRH3|HRH4</td><td>-24.1644</td><td>0</td><td>2</td><td>0</td><td>0</td><td>-48.2064</td><td>165.2568306</td></tr>
<tr><td>1</td><td>-25.2922</td><td>GO:0071868</td><td>M1</td><td>1</td><td>0</td><td>GO Biological Processes</td><td>19</td><td>cellular response to monoamine stimulus</td><td>-25.2922</td><td>32.43358</td><td>25.41479</td><td>30242</td><td>107</td><td>183</td><td>21</td><td>11.47541</td><td>2.356083</td><td>351|1128|1129|1131|1132|1133|1812|1813|1814|1815|3066|3350|3351|3356|3357|3358|3362|3363|5142|11255|59340</td><td>APP|CHRM1|CHRM2|CHRM3|CHRM4|CHRM5|DRD1|DRD2|DRD3|DRD4|HDAC2|HTR1A|HTR1B|HTR2A|HTR2B|HTR2C|HTR6|HTR7|PDE4B|HRH3|HRH4</td><td>-22.867</td><td>0</td><td>2</td><td>0</td><td>0</td><td>-48.2064</td><td>165.2568306</td></tr>
<tr><td>1</td><td>-25.2922</td><td>GO:0071870</td><td>M1</td><td>1</td><td>0</td><td>GO Biological Processes</td><td>19</td><td>cellular response to catecholamine stimulus</td><td>-25.2922</td><td>32.43358</td><td>25.41479</td><td>30242</td><td>107</td><td>183</td><td>21</td><td>11.47541</td><td>2.356083</td><td>351|1128|1129|1131|1132|1133|1812|1813|1814|1815|3066|3350|3351|3356|3357|3358|3362|3363|5142|11255|59340</td><td>APP|CHRM1|CHRM2|CHRM3|CHRM4|CHRM5|DRD1|DRD2|DRD3|DRD4|HDAC2|HTR1A|HTR1B|HTR2A|HTR2B|HTR2C|HTR6|HTR7|PDE4B|HRH3|HRH4</td><td>-22.867</td><td>0</td><td>2</td><td>0</td><td>0</td><td>-48.2064</td><td>165.2568306</td></tr>
<tr><td>1</td><td>-24.9312</td><td>GO:0071867</td><td>M1</td><td>1</td><td>0</td><td>GO Biological Processes</td><td>19</td><td>response to monoamine</td><td>-24.9312</td><td>31.26481</td><td>24.92464</td><td>30242</td><td>111</td><td>183</td><td>21</td><td>11.47541</td><td>2.356083</td><td>351|1128|1129|1131|1132|1133|1812|1813|1814|1815|3066|3350|3351|3356|3357|3358|3362|3363|5142|11255|59340</td><td>APP|CHRM1|CHRM2|CHRM3|CHRM4|CHRM5|DRD1|DRD2|DRD3|DRD4|HDAC2|HTR1A|HTR1B|HTR2A|HTR2B|HTR2C|HTR6|HTR7|PDE4B|HRH3|HRH4</td><td>-22.5205</td><td>0</td><td>2</td><td>0</td><td>0</td><td>-48.2064</td><td>165.2568306</td></tr>
<tr><td>1</td><td>-24.9312</td><td>GO:0071869</td><td>M1</td><td>1</td><td>0</td><td>GO Biological Processes</td><td>19</td><td>response to catecholamine</td><td>-24.9312</td><td>31.26481</td><td>24.92464</td><td>30242</td><td>111</td><td>183</td><td>21</td><td>11.47541</td><td>2.356083</td><td>351|1128|1129|1131|1132|1133|1812|1813|1814|1815|3066|3350|3351|3356|3357|3358|3362|3363|5142|11255|59340</td><td>APP|CHRM1|CHRM2|CHRM3|CHRM4|CHRM5|DRD1|DRD2|DRD3|DRD4|HDAC2|HTR1A|HTR1B|HTR2A|HTR2B|HTR2C|HTR6|HTR7|PDE4B|HRH3|HRH4</td><td>-22.5205</td><td>0</td><td>2</td><td>0</td><td>0</td><td>-48.2064</td><td>165.2568306</td></tr>
<tr><td>1</td><td>-24.3015</td><td>GO:0098664</td><td>M1</td><td>1</td><td>0</td><td>GO Biological Processes</td><td>19</td><td>G protein-coupled serotonin receptor</td><td>-24.3015</td><td>72.90743</td><td>32.73321</td><td>30242</td><td>34</td><td>183</td><td>15</td><td>8.196721</td><td>2.027793</td><td>1128|1129|1131|1132|1133|1815|3350|3351|33</td><td>CHRM1|CHRM2|CHRM3|CHRM4|CHRM5|</td><td>-21.925</td><td>0</td><td>2</td><td>0</td><td>0</td><td>-48.2064</td><td>165.2568306</td></tr>
</table>

<table>
<tr><td></td><td></td><td></td><td></td><td></td><td></td><td></td><td></td><td>signaling pathway</td><td></td><td></td><td></td><td></td><td></td><td></td><td></td><td></td><td></td><td>56|3357|3358|3362|3363|11255|59340</td><td>DRD4|HTR1A|HTR1B|HTR2A|HTR2B|HTR2C|HTR6|HTR7|HRH3|HRH4</td><td></td><td></td><td></td><td></td><td></td><td></td><td></td></tr>
<tr><td>1</td><td>-23.3674</td><td>GO:1903351</td><td>M1</td><td>1</td><td>0</td><td>GO Biological Processes</td><td>19</td><td>cellular response to dopamine</td><td>-23.3674</td><td>34.12913</td><td>24.83114</td><td>30242</td><td>92</td><td>183</td><td>19</td><td>10.38251</td><td>2.254873</td><td>1128|1129|1131|1132|1133|1812|1813|1814|1815|3066|3350|3351|3356|3357|3358|3362|3363|11255|59340</td><td>CHRM1|CHRM2|CHRM3|CHRM4|CHRM5|DRD1|DRD2|DRD3|DRD4|HDAC2|HTR1A|HTR1B|HTR2A|HTR2B|HTR2C|HTR6|HTR7|HRH3|HRH4</td><td>-21.0227</td><td>0</td><td>2</td><td>0</td><td>0</td><td>-48.2064</td><td>165.2568306</td></tr>
<tr><td>1</td><td>-23.2704</td><td>GO:1903350</td><td>M1</td><td>1</td><td>0</td><td>GO Biological Processes</td><td>19</td><td>response to dopamine</td><td>-23.2704</td><td>33.76215</td><td>24.68958</td><td>30242</td><td>93</td><td>183</td><td>19</td><td>10.38251</td><td>2.254873</td><td>1128|1129|1131|1132|1133|1812|1813|1814|1815|3066|3350|3351|3356|3357|3358|3362|3363|11255|59340</td><td>CHRM1|CHRM2|CHRM3|CHRM4|CHRM5|DRD1|DRD2|DRD3|DRD4|HDAC2|HTR1A|HTR1B|HTR2A|HTR2B|HTR2C|HTR6|HTR7|HRH3|HRH4</td><td>-20.9318</td><td>0</td><td>2</td><td>0</td><td>0</td><td>-48.2064</td><td>165.2568306</td></tr>
<tr><td>1</td><td>-23.181</td><td>GO:0007210</td><td>M1</td><td>1</td><td>0</td><td>GO Biological Processes</td><td>19</td><td>serotonin receptor signaling pathway</td><td>-23.181</td><td>63.56032</td><td>30.503</td><td>30242</td><td>39</td><td>183</td><td>15</td><td>8.196721</td><td>2.027793</td><td>1128|1129|1131|1132|1133|1815|3350|3351|3356|3357|3358|3362|3363|11255|59340</td><td>CHRM1|CHRM2|CHRM3|CHRM4|CHRM5|DRD4|HTR1A|HTR1B|HTR2A|HTR2B|HTR2C|HTR6|HTR7|HRH3|HRH4</td><td>-20.8483</td><td>0</td><td>2</td><td>0</td><td>0</td><td>-48.2064</td><td>165.2568306</td></tr>
<tr><td>1</td><td>-17.8122</td><td>GO:0007197</td><td>M1</td><td>1</td><td>0</td><td>GO Biological Processes</td><td>19</td><td>adenylate cyclase-inhibiting G protein-coupled acetylcholine receptor signaling pathway</td><td>-17.8122</td><td>165.2568</td><td>36.25409</td><td>30242</td><td>8</td><td>183</td><td>8</td><td>4.371585</td><td>1.511428</td><td>1128|1129|1131|1132|1133|4988|11255|59340</td><td>CHRM1|CHRM2|CHRM3|CHRM4|CHRM5|OPRM1|HRH3|HRH4</td><td>-15.677</td><td>0</td><td>2</td><td>0</td><td>0</td><td>-48.2064</td><td>165.2568306</td></tr>
<tr><td>1</td><td>-12.9585</td><td>GO:0007213</td><td>M1</td><td>1</td><td>0</td><td>GO Biological Processes</td><td>19</td><td>G protein-coupled acetylcholine receptor signaling pathway</td><td>-12.9585</td><td>69.58182</td><td>23.33208</td><td>30242</td><td>19</td><td>183</td><td>8</td><td>4.371585</td><td>1.511428</td><td>1128|1129|1131|1132|1133|4988|11255|59340</td><td>CHRM1|CHRM2|CHRM3|CHRM4|CHRM5|OPRM1|HRH3|HRH4</td><td>-11.07</td><td>0</td><td>2</td><td>0</td><td>0</td><td>-48.2064</td><td>165.2568306</td></tr>
<tr><td>1</td><td>-12.7948</td><td>GO:1905144</td><td>M1</td><td>1</td><td>0</td><td>GO Biological Processes</td><td>19</td><td>response to acetylcholine</td><td>-12.7948</td><td>47.97779</td><td>20.41866</td><td>30242</td><td>31</td><td>183</td><td>9</td><td>4.918033</td><td>1.598524</td><td>1128|1129|1131|1132|1133|4988|9475|11255|59340</td><td>CHRM1|CHRM2|CHRM3|CHRM4|CHRM5|OPRM1|ROCK2|HRH3|HRH4</td><td>-10.9169</td><td>0</td><td>2</td><td>0</td><td>0</td><td>-48.2064</td><td>165.2568306</td></tr>
<tr><td>1</td><td>-12.7948</td><td>GO:1905145</td><td>M1</td><td>1</td><td>0</td><td>GO Biological Processes</td><td>19</td><td>cellular response to acetylcholine</td><td>-12.7948</td><td>47.97779</td><td>20.41866</td><td>30242</td><td>31</td><td>183</td><td>9</td><td>4.918033</td><td>1.598524</td><td>1128|1129|1131|1132|1133|4988|9475|11255|59340</td><td>CHRM1|CHRM2|CHRM3|CHRM4|CHRM5|OPRM1|ROCK2|HRH3|HRH4</td><td>-10.9169</td><td>0</td><td>2</td><td>0</td><td>0</td><td>-48.2064</td><td>165.2568306</td></tr>
<tr><td>1</td><td>-11.9029</td><td>GO:0098926</td><td>M1</td><td>1</td><td>0</td><td>GO Biological Processes</td><td>19</td><td>postsynaptic signal transduction</td><td>-11.9029</td><td>39.13978</td><td>18.35583</td><td>30242</td><td>38</td><td>183</td><td>9</td><td>4.918033</td><td>1.598524</td><td>1128|1129|1131|1132|1133|4988|5970|11255|59340</td><td>CHRM1|CHRM2|CHRM3|CHRM4|CHRM5|OPRM1|RELA|HRH3|HRH4</td><td>-10.0843</td><td>0</td><td>2</td><td>0</td><td>0</td><td>-48.2064</td><td>165.2568306</td></tr>
<tr><td>1</td><td>-11.2266</td><td>GO:0095500</td><td>M1</td><td>1</td><td>0</td><td>GO Biological Processes</td><td>19</td><td>acetylcholine receptor signaling pathway</td><td>-11.2266</td><td>45.58809</td><td>18.74379</td><td>30242</td><td>29</td><td>183</td><td>8</td><td>4.371585</td><td>1.511428</td><td>1128|1129|1131|1132|1133|4988|11255|59340</td><td>CHRM1|CHRM2|CHRM3|CHRM4|CHRM5|OPRM1|HRH3|HRH4</td><td>-9.45058</td><td>0</td><td>2</td><td>0</td><td>0</td><td>-48.2064</td><td>165.2568306</td></tr>
<tr><td>1</td><td>-44.2494</td><td>GO:0099536</td><td>M1</td><td>1</td><td>0</td><td>GO Biological Processes</td><td>19</td><td>synaptic signaling</td><td>-44.2494</td><td>12.30636</td><td>24.498</td><td>30242</td><td>752</td><td>183</td><td>56</td><td>30.60109</td><td>3.406585</td><td>134|135|148|150|154|351|1128|1129|1131|1132|1133|1268|1728|1812|1813|1814|1815|1956|21</td><td>ADORA1|ADORA2A|ADRA1A|ADRA2A|ADRB2|APP|CHRM1|CHRM2|CHRM3|CHRM</td><td>-40.9759</td><td>0</td><td>3</td><td>1</td><td>1</td><td>-44.2494</td><td>38.55992714</td></tr>
</table>

																		49|2554|2558|2566|2891|2902|2903|2904|2915|3061|3062|3350|3351|3356|3357|3358|3362|3363|4842|4914|4915|4986|4988|5024|5582|5743|5970|6197|6347|6530|6531|6532|7124|7442|11255|23621|59340|729230	4|CHRM5|CNR1|NQO1|DRD1|DRD2|DRD3|DRD4|EGFR|F2R|GABRA1|GABRA5|GABRG2|GRIA2|GRIN1|GRIN2A|GRIN2B|GRM5|HCRTR1|HCRTR2|HTR1A|HTR1B|HTR2A|HTR2B|HTR2C|HTR6|HTR7|NOS1|NTRK1|NTRK2|OPRK1|OPRM1|P2RX3|PRKCG|PTGS2|RELA|RPS6KA3|CCL2|SLC6A2|SLC6A3|SLC6A4|TNF|TRPV1|HRH3|BACE1|HRH4|CCR2							
1	-43.7433	GO:0099537	M1	1	0	GO Biological Processes	19	trans-synaptic signaling	-43.7433	12.48506	24.47493	30242	728	183	55	30.05464	3.389298	134|135|148|150|154|351|1128|1129|1131|1132|1133|1268|1728|1812|1813|1814|1815|1956|2149|2554|2558|2566|2891|2902|2903|2904|2915|3061|3062|3350|3351|3356|3357|3358|3362|3363|4842|4914|4915|4986|4988|5024|5582|5743|6197|6347|6530|6531|6532|7124|7442|11255|23621|59340|729230	ADORA1|ADORA2A|ADRA1A|ADRA2A|ADRB2|APP|CHRM1|CHRM2|CHRM3|CHRM4|CHRM5|CNR1|NQO1|DRD1|DRD2|DRD3|DRD4|EGFR|F2R|GABRA1|GABRA5|GABRG2|GRIA2|GRIN1|GRIN2A|GRIN2B|GRM5|HCRTR1|HCRTR2|HTR1A|HTR1B|HTR2A|HTR2B|HTR2C|HTR6|HTR7|NOS1|NTRK1|NTRK2|OPRK1|OPRM1|P2RX3|PRKCG|PTGS2|RPS6KA3|CCL2|SLC6A2|SLC6A3|SLC6A4|TNF|TRPV1|HRH3|BACE1|HRH4|CCR2	-40.5473	0	3	0	0	-44.2494	38.55992714
1	-42.7276	GO:0007268	M1	1	0	GO Biological Processes	19	chemical synaptic transmission	-42.7276	12.39426	24.14437	30242	720	183	54	29.5082	3.371437	134|135|148|150|154|351|1128|1129|1131|1132|1133|1268|1728|1812|1813|1814|1815|1956|2149|2554|2558|2566|2891|2902|2903|2904|2915|3061|3062|3350|3351|3356|3357|3358|3362|3363|4914|4915|4986|4988|5024|5582|5743|6197|6347|6530|6531|6532|7124|7442|11255|23621|59340|729230	ADORA1|ADORA2A|ADRA1A|ADRA2A|ADRB2|APP|CHRM1|CHRM2|CHRM3|CHRM4|CHRM5|CNR1|NQO1|DRD1|DRD2|DRD3|DRD4|EGFR|F2R|GABRA1|GABRA5|GABRG2|GRIA2|GRIN1|GRIN2A|GRIN2B|GRM5|HCRTR1|HCRTR2|HTR1A|HTR1B|HTR2A|HTR2B|HTR2C|HTR6|HTR7|NTRK1|NTRK2|	-39.6107	0	3	0	0	-44.2494	38.55992714

<table>
<tr><td></td><td></td><td></td><td></td><td></td><td></td><td></td><td></td><td></td><td></td><td></td><td></td><td></td><td></td><td></td><td></td><td></td><td></td><td></td><td>OPRK1|OPRM1|P2RX3|PRKCG|PTGS2|RPS6KA3|CCL2|SLC6A2|SLC6A3|SLC6A4|TNF|TRPV1|HRH3|BACE1|HRH4|CCR2</td><td></td><td></td><td></td><td></td><td></td><td></td><td></td></tr>
<tr><td>1</td><td>-42.7276</td><td>GO:0098916</td><td>M1</td><td>1</td><td>0</td><td>GO Biological Processes</td><td>19</td><td>anterograde trans-synaptic signaling</td><td>-42.7276</td><td>12.39426</td><td>24.14437</td><td>30242</td><td>720</td><td>183</td><td>54</td><td>29.5082</td><td>3.371437</td><td>134|135|148|150|154|351|1128|1129|1131|1132|1133|1268|1728|1812|1813|1814|1815|1956|2149|2554|2558|2566|2891|2902|2903|2904|2915|3061|3062|3350|3351|3356|3357|3358|3362|3363|4914|4915|4986|4988|5024|5582|5743|6197|6347|6530|6531|6532|7124|7442|11255|23621|59340|729230</td><td>ADORA1|ADORA2A|ADRA1A|ADRA2A|ADRB2|APP|CHRM1|CHRM2|CHRM3|CHRM4|CHRM5|CNR1|NQO1|DRD1|DRD2|DRD3|DRD4|EGFR|F2R|GABRA1|GABRA5|GABRG2|GRIA2|GRIN1|GRIN2A|GRIN2B|GRM5|HCRTR1|HCRTR2|HTR1A|HTR1B|HTR2A|HTR2B|HTR2C|HTR6|HTR7|NTRK1|NTRK2|OPRK1|OPRM1|P2RX3|PRKCG|PTGS2|RPS6KA3|CCL2|SLC6A2|SLC6A3|SLC6A4|TNF|TRPV1|HRH3|BACE1|HRH4|CCR2</td><td>-39.6107</td><td>0</td><td>3</td><td>0</td><td>0</td><td>-44.2494</td><td>38.55992714</td></tr>
<tr><td>1</td><td>-34.62</td><td>GO:0007610</td><td>M1</td><td>1</td><td>0</td><td>GO Biological Processes</td><td>19</td><td>behavior</td><td>-34.62</td><td>12.51515</td><td>21.8676</td><td>30242</td><td>581</td><td>183</td><td>44</td><td>24.04372</td><td>3.159054</td><td>100|134|135|153|351|596|836|1268|1812|1813|1814|1815|1956|2558|2566|2902|2903|2904|2915|3061|3062|3066|3156|3350|3351|3356|3357|3358|3643|4914|4915|4985|4986|4988|5024|5465|5582|5743|6326|6335|6531|6532|7157|7442</td><td>ADA|ADORA1|ADORA2A|ADRB1|APP|BCL2|CASP3|CNR1|DRD1|DRD2|DRD3|DRD4|EGFR|GABRA5|GABRG2|GRIN1|GRIN2A|GRIN2B|GRM5|HCRTR1|HCRTR2|HDAC2|HMGCR|HTR1A|HTR1B|HTR2A|HTR2B|HTR2C|INSR|NTRK1|NTRK2|OPRD1|OPRK1|OPRM1|P2RX3|PPARA|PRKCG|PTGS2|SCN2A|SCN9A|SLC6A3|SLC6A4|TP53|TRPV1</td><td>-31.8864</td><td>0</td><td>3</td><td>0</td><td>0</td><td>-44.2494</td><td>38.55992714</td></tr>
<tr><td>1</td><td>-21.9107</td><td>GO:0050890</td><td>M1</td><td>1</td><td>0</td><td>GO Biological Processes</td><td>19</td><td>cognition</td><td>-21.9107</td><td>14.5158</td><td>18.23285</td><td>30242</td><td>296</td><td>183</td><td>26</td><td>14.20765</td><td>2.580833</td><td>134|351|836|1128|1268|1812|1813|1814|1956|2558|2902|2903|2904|2915|3156|3356|3643|4914|4915|4986|5582|5743|6326|6532|7124|11255</td><td>ADORA1|APP|CASP3|CHRM1|CNR1|DRD1|DRD2|DRD3|EGFR|GABRA5|GRIN1|GRIN2A|GRIN2B|GRM5|HMGCR|HTR2A|INSR|NTRK1|NTRK2|OPRK1|PRKCG|PTGS2|SCN2A|SLC6A4|TNF|HRH3</td><td>-19.6285</td><td>0</td><td>3</td><td>0</td><td>0</td><td>-44.2494</td><td>38.55992714</td></tr>
</table>

<table>
<tr><td>1</td><td>-18.896</td><td>GO:0051966</td><td>M1</td><td>1</td><td>0</td><td>GO Biological Processes</td><td>19</td><td>regulation of synaptic transmission, glutamatergic</td><td>-18.896</td><td>35.9254</td><td>22.66173</td><td>30242</td><td>69</td><td>183</td><td>15</td><td>8.196721</td><td>2.027793</td><td>134|135|1268|1812|1813|1814|1956|2915|3351|3356|4914|5743|6347|7124|729230</td><td>ADORA1|ADORA2A|CNR1|DRD1|DRD2|DRD3|EGFR|GRM5|HTR1B|HTR2A|NTRK1|PTGS2|CCL2|TNF|CCR2</td><td>-16.6956</td><td>0</td><td>3</td><td>0</td><td>0</td><td>-44.2494</td><td>38.55992714</td></tr>
<tr><td>1</td><td>-18.7042</td><td>GO:0050804</td><td>M1</td><td>1</td><td>0</td><td>GO Biological Processes</td><td>19</td><td>modulation of chemical synaptic transmission</td><td>-18.7042</td><td>10.16386</td><td>15.09097</td><td>30242</td><td>439</td><td>183</td><td>27</td><td>14.7541</td><td>2.621607</td><td>134|135|148|150|154|351|1268|1812|1813|1814|1956|2902|2903|2904|2915|3351|3356|4914|4915|5024|5582|5743|6347|6532|7124|23621|729230</td><td>ADORA1|ADORA2A|ADRA1A|ADRA2A|ADRB2|APP|CNR1|DRD1|DRD2|DRD3|EGFR|GRIN1|GRIN2A|GRIN2B|GRM5|HTR1B|HTR2A|NTRK1|NTRK2|P2RX3|PRKCG|PTGS2|CCL2|SLC6A4|TNF|BACE1|CCR2</td><td>-16.5169</td><td>0</td><td>3</td><td>0</td><td>0</td><td>-44.2494</td><td>38.55992714</td></tr>
<tr><td>1</td><td>-18.6789</td><td>GO:0099177</td><td>M1</td><td>1</td><td>0</td><td>GO Biological Processes</td><td>19</td><td>regulation of trans-synaptic signaling</td><td>-18.6789</td><td>10.14076</td><td>15.07031</td><td>30242</td><td>440</td><td>183</td><td>27</td><td>14.7541</td><td>2.621607</td><td>134|135|148|150|154|351|1268|1812|1813|1814|1956|2902|2903|2904|2915|3351|3356|4914|4915|5024|5582|5743|6347|6532|7124|23621|729230</td><td>ADORA1|ADORA2A|ADRA1A|ADRA2A|ADRB2|APP|CNR1|DRD1|DRD2|DRD3|EGFR|GRIN1|GRIN2A|GRIN2B|GRM5|HTR1B|HTR2A|NTRK1|NTRK2|P2RX3|PRKCG|PTGS2|CCL2|SLC6A4|TNF|BACE1|CCR2</td><td>-16.4958</td><td>0</td><td>3</td><td>0</td><td>0</td><td>-44.2494</td><td>38.55992714</td></tr>
<tr><td>1</td><td>-18.4555</td><td>GO:0035249</td><td>M1</td><td>1</td><td>0</td><td>GO Biological Processes</td><td>19</td><td>synaptic transmission, glutamatergic</td><td>-18.4555</td><td>28.74032</td><td>20.79209</td><td>30242</td><td>92</td><td>183</td><td>16</td><td>8.743169</td><td>2.088053</td><td>134|135|1268|1812|1813|1814|1956|2891|2915|3351|3356|4914|5743|6347|7124|729230</td><td>ADORA1|ADORA2A|CNR1|DRD1|DRD2|DRD3|EGFR|GRIA2|GRM5|HTR1B|HTR2A|NTRK1|PTGS2|CCL2|TNF|CCR2</td><td>-16.2807</td><td>0</td><td>3</td><td>0</td><td>0</td><td>-44.2494</td><td>38.55992714</td></tr>
<tr><td>1</td><td>-18.3974</td><td>GO:0007611</td><td>M1</td><td>1</td><td>0</td><td>GO Biological Processes</td><td>19</td><td>learning or memory</td><td>-18.3974</td><td>14.25745</td><td>16.58823</td><td>30242</td><td>255</td><td>183</td><td>22</td><td>12.02186</td><td>2.404073</td><td>351|836|1268|1812|1813|1814|1956|2558|2902|2903|2904|2915|3156|3356|3643|4914|4915|4986|5582|5743|6326|6532</td><td>APP|CASP3|CNR1|DRD1|DRD2|DRD3|EGFR|GABRA5|GRIN1|GRIN2A|GRIN2B|GRM5|HMGCR|HTR2A|INSR|NTRK1|NTRK2|OPRK1|PRKCG|PTGS2|SCN2A|SLC6A4</td><td>-16.2348</td><td>0</td><td>3</td><td>0</td><td>0</td><td>-44.2494</td><td>38.55992714</td></tr>
<tr><td>1</td><td>-15.6436</td><td>GO:0050806</td><td>M1</td><td>1</td><td>0</td><td>GO Biological Processes</td><td>19</td><td>positive regulation of synaptic transmission</td><td>-15.6436</td><td>17.13028</td><td>16.16128</td><td>30242</td><td>164</td><td>183</td><td>17</td><td>9.289617</td><td>2.145862</td><td>134|135|148|351|1268|1812|1813|1956|2903|2904|4914|4915|5582|5743|6347|7124|729230</td><td>ADORA1|ADORA2A|ADRA1A|APP|CNR1|DRD1|DRD2|EGFR|GRIN2A|GRIN2B|NTRK1|NTRK2|PRKCG|PTGS2|CCL2|TNF|CCR2</td><td>-13.6295</td><td>0</td><td>3</td><td>0</td><td>0</td><td>-44.2494</td><td>38.55992714</td></tr>
<tr><td>1</td><td>-9.31949</td><td>GO:0051968</td><td>M1</td><td>1</td><td>0</td><td>GO Biological Processes</td><td>19</td><td>positive regulation of synaptic transmission, glutamatergic</td><td>-9.31949</td><td>38.55993</td><td>16.0595</td><td>30242</td><td>30</td><td>183</td><td>7</td><td>3.825137</td><td>1.417845</td><td>135|1812|1956|4914|5743|6347|729230</td><td>ADORA2A|DRD1|EGFR|NTRK1|PTGS2|CCL2|CCR2</td><td>-7.68102</td><td>0</td><td>3</td><td>0</td><td>0</td><td>-44.2494</td><td>38.55992714</td></tr>
<tr><td>1</td><td>-38.1485</td><td>GO:0050801</td><td>M1</td><td>1</td><td>0</td><td>GO Biological Processes</td><td>19</td><td>ion homeostasis</td><td>-38.1485</td><td>10.86391</td><td>21.9343</td><td>30242</td><td>791</td><td>183</td><td>52</td><td>28.4153</td><td>3.333962</td><td>134|135|146|147|148|185|351|596|624|760|775|</td><td>ADORA1|ADORA2A|ADRA1D|ADRA1B|A</td><td>-35.1498</td><td>0</td><td>4</td><td>1</td><td>1</td><td>-38.1485</td><td>34.9581757</td></tr>
</table>

																		1080|1230|1268|1812|1813|1814|1815|1909|1910|1956|1991|2099|2147|2149|2902|2903|2904|2915|3061|3062|3351|3356|3357|3358|3558|3577|3757|3778|4842|5024|5734|6331|6336|6916|7421|7442|8989|10800|59340|59341|729230	DRA1A|AGTR1|APP|BCL2|BDKRB2|CA2|CACNA1C|CFTR|CCR1|CNR1|DRD1|DRD2|DRD3|DRD4|EDNRA|EDNRB|EGFR|ELANE|ESR1|F2|F2R|GRIN1|GRIN2A|GRIN2B|GRM5|HCRTR1|HCRTR2|HTR1B|HTR2A|HTR2B|HTR2C|IL2|CXCR1|KCNH2|KCNMA1|NOS1|P2RX3|PTGER4|SCN5A|SCN10A|TBXAS1|VDR|TRPV1|TRPA1|CYSLTR1|HRH4|TRPV4|CCR2								
1	-38.1417	GO:0006874	M1	1	0	GO Biological Processes	19	cellular calcium ion homeostasis	-38.1417	15.82638	24.69662	30242	449	183	43	23.49727	3.134163	134|146|147|148|185|351|596|624|775|1230|1268|1812|1813|1814|1815|1909|1910|1991|2099|2147|2149|2902|2903|2904|2915|3061|3062|3351|3356|3357|3358|3558|3577|4842|5024|5734|7421|7442|8989|10800|59340|59341|729230	ADORA1|ADRA1D|ADRA1B|ADRA1A|AGTR1|APP|BCL2|BDKRB2|CACNA1C|CCR1|CNR1|DRD1|DRD2|DRD3|DRD4|EDNRA|EDNRB|ELANE|ESR1|F2|F2R|GRIN1|GRIN2A|GRIN2B|GRM5|HCRTR1|HCRTR2|HTR1B|HTR2A|HTR2B|HTR2C|IL2|CXCR1|NOS1|P2RX3|PTGER4|VDR|TRPV1|TRPA1|CYSLTR1|HRH4|TRPV4|CCR2	-35.1498	0	4	0	0	-38.1485	34.9581757
1	-37.6489	GO:0055074	M1	1	0	GO Biological Processes	19	calcium ion homeostasis	-37.6489	15.41441	24.33403	30242	461	183	43	23.49727	3.134163	134|146|147|148|185|351|596|624|775|1230|1268|1812|1813|1814|1815|1909|1910|1991|2099|2147|2149|2902|2903|2904|2915|3061|3062|3351|3356|3357|3358|3558|3577|4842|5024|5734|7421|7442|8989|10800|59340|59341|729230	ADORA1|ADRA1D|ADRA1B|ADRA1A|AGTR1|APP|BCL2|BDKRB2|CACNA1C|CCR1|CNR1|DRD1|DRD2|DRD3|DRD4|EDNRA|EDNRB|ELANE|ESR1|F2|F2R|GRIN1|GRIN2A|GRIN2B|GRM5|HCRTR1|HCRTR2|HTR1B|HTR2A|HTR2B|HTR2C|IL2|CXCR1|NOS1|P2RX3|PTGER4|VDR|TRPV1|TRPA1|CYSLTR1|HRH4|TRPV4|CCR2	-34.6833	0	4	0	0	-38.1485	34.9581757
1	-37.1479	GO:0072507	M1	1	0	GO Biological Processes	19	divalent inorganic cation homeostasis	-37.1479	14.31358	23.6122	30242	508	183	44	24.04372	3.159054	134|146|147|148|185|351|596|624|775|1230|1268|1812|1813|1814|1815|1909|1910|1956|1991|2	ADORA1|ADRA1D|ADRA1B|ADRA1A|AGTR1|APP|BCL2|BDKRB2|CACNA1C|CCR1|	-34.2072	0	4	0	0	-38.1485	34.9581757

																		099|2147|2149|2902|2903|2904|2915|3061|3062|3351|3356|3357|3358|3558|3577|4842|5024|5734|7421|7442|8989|10800|59340|59341|729230	CNR1|DRD1|DRD2|DRD3|DRD4|EDNRA|EDNRB|EGFR|ELANE|ESR1|F2|F2R|GRIN1|GRIN2A|GRIN2B|GRM5|HCRTR1|HCRTR2|HTR1B|HTR2A|HTR2B|HTR2C|IL2|CXCR1|NOS1|P2RX3|PTGER4|VDR|TRPV1|TRPA1|CYSLTR1|HRH4|TRPV4|CCR2							
1	-37.0567	GO:0098771	M1	1	0	GO Biological Processes	19	inorganic ion homeostasis	-37.0567	11.10597	21.77733	30242	744	183	50	27.3224	3.29408	134|135|146|147|148|185|351|596|624|760|775|1080|1230|1268|1812|1813|1814|1815|1909|1910|1956|1991|2099|2147|2149|2902|2903|2904|2915|3061|3062|3351|3356|3357|3358|3558|3577|3757|3778|4842|5024|5734|6916|7421|7442|8989|10800|59340|59341|729230	ADORA1|ADORA2A|ADRA1D|ADRA1B|ADRA1A|AGTR1|APP|BCL2|BDKRB2|CA2|CACNA1C|CFTR|CCR1|CNR1|DRD1|DRD2|DRD3|DRD4|EDNRA|EDNRB|EGFR|ELANE|ESR1|F2|F2R|GRIN1|GRIN2A|GRIN2B|GRM5|HCRTR1|HCRTR2|HTR1B|HTR2A|HTR2B|HTR2C|IL2|CXCR1|KCNH2|KCNMA1|NOS1|P2RX3|PTGER4|TBXAS1|VDR|TRPV1|TRPA1|CYSLTR1|HRH4|TRPV4|CCR2	-34.1394	0	4	0	0	-38.1485	34.9581757
1	-36.8771	GO:0051480	M1	1	0	GO Biological Processes	19	regulation of cytosolic calcium ion concentration	-36.8771	18.20626	25.40833	30242	354	183	39	21.31148	3.027171	134|146|147|148|185|596|624|775|1230|1268|1812|1813|1814|1909|1910|2099|2147|2149|2902|2903|2904|2915|3061|3062|3351|3356|3357|3358|3558|3577|4842|5024|5734|7442|8989|10800|59340|59341|729230	ADORA1|ADRA1D|ADRA1B|ADRA1A|AGTR1|BCL2|BDKRB2|CACNA1C|CCR1|CNR1|DRD1|DRD2|DRD3|EDNRA|EDNRB|ESR1|F2|F2R|GRIN1|GRIN2A|GRIN2B|GRM5|HCRTR1|HCRTR2|HTR1B|HTR2A|HTR2B|HTR2C|IL2|CXCR1|NOS1|P2RX3|PTGER4|TRPV1|TRPA1|CYSLTR1|HRH4|TRPV4|CCR2	-33.9821	0	4	0	0	-38.1485	34.9581757
1	-36.6279	GO:0072503	M1	1	0	GO Biological Processes	19	cellular divalent inorganic cation homeostasis	-36.6279	14.59147	23.59322	30242	487	183	43	23.49727	3.134163	134|146|147|148|185|351|596|624|775|1230|1268|1812|1813|1814|1815|1909|1910|1991|2099|2147|2149|2902|2903|2904|2915|3061|3062|3351|3356|3357|3358|3558|	ADORA1|ADRA1D|ADRA1B|ADRA1A|AGTR1|APP|BCL2|BDKRB2|CACNA1C|CCR1|CNR1|DRD1|DRD2|DRD3|DRD4|EDNRA|EDNRB|ELANE|ESR1|F	-33.7541	0	4	0	0	-38.1485	34.9581757

<table>
<tr><td></td><td></td><td></td><td></td><td></td><td></td><td></td><td></td><td></td><td></td><td></td><td></td><td></td><td></td><td></td><td></td><td></td><td></td><td>3577|4842|5024|5734|7421|7442|8989|10800|59340|59341|729230</td><td>2|F2R|GRIN1|GRIN2A|GRIN2B|GRM5|HCRTR1|HCRTR2|HTR1B|HTR2A|HTR2B|HTR2C|IL2|CXCR1|NOS1|P2RX3|PTGER4|VDR|TRPV1|TRPA1|CYSLTR1|HRH4|TRPV4|CCR2</td><td></td><td></td><td></td><td></td><td></td><td></td><td></td></tr>
<tr><td>1</td><td>-36.2491</td><td>GO:0055080</td><td>M1</td><td>1</td><td>0</td><td>GO Biological Processes</td><td>19</td><td>cation homeostasis</td><td>-36.2491</td><td>11.09258</td><td>21.53777</td><td>30242</td><td>730</td><td>183</td><td>49</td><td>26.77596</td><td>3.27321</td><td>134|135|146|147|148|185|351|596|624|760|775|1080|1230|1268|1812|1813|1814|1815|1909|1910|1956|1991|2099|2147|2149|2902|2903|2904|2915|3061|3062|3351|3356|3357|3358|3558|3577|3757|3778|4842|5024|5734|7421|7442|8989|10800|59340|59341|729230</td><td>ADORA1|ADORA2A|ADRA1D|ADRA1B|ADRA1A|AGTR1|APP|BCL2|BDKRB2|CA2|CACNA1C|CFTR|CCR1|CNR1|DRD1|DRD2|DRD3|DRD4|EDNRA|EDNRB|EGFR|ELANE|ESR1|F2|F2R|GRIN1|GRIN2A|GRIN2B|GRM5|HCRTR1|HCRTR2|HTR1B|HTR2A|HTR2B|HTR2C|IL2|CXCR1|KCNH2|KCNMA1|NOS1|P2RX3|PTGER4|VDR|TRPV1|TRPA1|CYSLTR1|HRH4|TRPV4|CCR2</td><td>-33.4148</td><td>0</td><td>4</td><td>0</td><td>0</td><td>-38.1485</td><td>34.9581757</td></tr>
<tr><td>1</td><td>-35.9242</td><td>GO:0055065</td><td>M1</td><td>1</td><td>0</td><td>GO Biological Processes</td><td>19</td><td>metal ion homeostasis</td><td>-35.9242</td><td>11.80406</td><td>21.8629</td><td>30242</td><td>658</td><td>183</td><td>47</td><td>25.68306</td><td>3.229548</td><td>134|135|146|147|148|185|351|596|624|775|1230|1268|1812|1813|1814|1815|1909|1910|1956|1991|2099|2147|2149|2902|2903|2904|2915|3061|3062|3351|3356|3357|3358|3558|3577|3757|3778|4842|5024|5734|7421|7442|8989|10800|59340|59341|729230</td><td>ADORA1|ADORA2A|ADRA1D|ADRA1B|ADRA1A|AGTR1|APP|BCL2|BDKRB2|CACNA1C|CCR1|CNR1|DRD1|DRD2|DRD3|DRD4|EDNRA|EDNRB|EGFR|ELANE|ESR1|F2|F2R|GRIN1|GRIN2A|GRIN2B|GRM5|HCRTR1|HCRTR2|HTR1B|HTR2A|HTR2B|HTR2C|IL2|CXCR1|KCNH2|KCNMA1|NOS1|P2RX3|PTGER4|VDR|TRPV1|TRPA1|CYSLTR1|HRH4|TRPV4|CCR2</td><td>-33.1084</td><td>0</td><td>4</td><td>0</td><td>0</td><td>-38.1485</td><td>34.9581757</td></tr>
<tr><td>1</td><td>-35.6842</td><td>GO:0006873</td><td>M1</td><td>1</td><td>0</td><td>GO Biological Processes</td><td>19</td><td>cellular ion homeostasis</td><td>-35.6842</td><td>11.66227</td><td>21.70968</td><td>30242</td><td>666</td><td>183</td><td>47</td><td>25.68306</td><td>3.229548</td><td>134|146|147|148|185|351|596|624|760|775|1080|1230|1268|1812|1813|1814|1815|1909|1910|1991|2099|2147|2149|2902|2903|2904|2915|3061|3062|3351|3356|3357|3358|3558|3577|3778|48</td><td>ADORA1|ADRA1D|ADRA1B|ADRA1A|AGTR1|APP|BCL2|BDKRB2|CA2|CACNA1C|CFTR|CCR1|CNR1|DRD1|DRD2|DRD3|DRD4|EDNRA|EDNRB|ELANE|ESR1|F2|F2R|GRIN1</td><td>-32.8862</td><td>0</td><td>4</td><td>0</td><td>0</td><td>-38.1485</td><td>34.9581757</td></tr>
</table>

																		42|5024|5734|6916|7421|7442|8989|10800|59340|59341|729230	|GRIN2A|GRIN2B|GRM5|HCRTR1|HCRTR2|HTR1B|HTR2A|HTR2B|HTR2C|IL2|CXCR1|KCNMA1|NOS1|P2RX3|PTGER4|TBXAS1|VDR|TRPV1|TRPA1|CYSLTR1|HRH4|TRPV4|CCR2							
1	-34.9731	GO:0030003	M1	1	0	GO Biological Processes	19	cellular cation homeostasis	-34.9731	11.71312	21.52683	30242	649	183	46	25.13661	3.206731	134|146|147|148|185|351|596|624|760|775|1080|1230|1268|1812|1813|1814|1815|1909|1910|1991|2099|2147|2149|2902|2903|2904|2915|3061|3062|3351|3356|3357|3358|3558|3577|3778|4842|5024|5734|7421|7442|8989|10800|59340|59341|729230	ADORA1|ADRA1D|ADRA1B|ADRA1A|AGTR1|APP|BCL2|BDKRB2|CA2|CACNA1C|CFTR|CCR1|CNR1|DRD1|DRD2|DRD3|DRD4|EDNRA|EDNRB|ELANE|ESR1|F2|F2R|GRIN1|GRIN2A|GRIN2B|GRM5|HCRTR1|HCRTR2|HTR1B|HTR2A|HTR2B|HTR2C|IL2|CXCR1|KCNMA1|NOS1|P2RX3|PTGER4|VDR|TRPV1|TRPA1|CYSLTR1|HRH4|TRPV4|CCR2	-32.1921	0	4	0	0	-38.1485	34.9581757
1	-34.62	GO:0006875	M1	1	0	GO Biological Processes	19	cellular metal ion homeostasis	-34.62	12.51515	21.8676	30242	581	183	44	24.04372	3.159054	134|146|147|148|185|351|596|624|775|1230|1268|1812|1813|1814|1815|1909|1910|1991|2099|2147|2149|2902|2903|2904|2915|3061|3062|3351|3356|3357|3358|3558|3577|3778|4842|5024|5734|7421|7442|8989|10800|59340|59341|729230	ADORA1|ADRA1D|ADRA1B|ADRA1A|AGTR1|APP|BCL2|BDKRB2|CACNA1C|CCR1|CNR1|DRD1|DRD2|DRD3|DRD4|EDNRA|EDNRB|ELANE|ESR1|F2|F2R|GRIN1|GRIN2A|GRIN2B|GRM5|HCRTR1|HCRTR2|HTR1B|HTR2A|HTR2B|HTR2C|IL2|CXCR1|KCNMA1|NOS1|P2RX3|PTGER4|VDR|TRPV1|TRPA1|CYSLTR1|HRH4|TRPV4|CCR2	-31.8864	0	4	0	0	-38.1485	34.9581757
1	-31.5605	GO:0007204	M1	1	0	GO Biological Processes	19	positive regulation of cytosolic calcium ion concentration	-31.5605	17.61358	23.27514	30242	319	183	34	18.57923	2.87512	146|147|148|185|596|624|775|1230|1268|1812|1813|1814|1909|1910|2099|2147|2149|2902|2903|2904|3356|3357|3358|3558|3577|4842|5024|5734|7442|8989|10800|59340|59341|729230	ADRA1D|ADRA1B|ADRA1A|AGTR1|BCL2|BDKRB2|CACNA1C|CCR1|CNR1|DRD1|DRD2|DRD3|EDNRA|EDNRB|ESR1|F2|F2R|GRIN1|GRIN2A|GRIN2B|HTR2A|HTR2B|HTR2C|IL2|CXCR1|NOS1|P2RX3|PTGER4|TRP	-28.9208	0	4	0	0	-38.1485	34.9581757

																			V1|TRPA1|CYSLTR1|HRH4|TRPV4|CCR2							
1	-27.2643	GO:0007200	M1	1	0	GO Biological Processes	19	phospholipase C-activating G protein-coupled receptor signaling pathway	-27.2643	34.95818	27.067	30242	104	183	22	12.02186	2.404073	146|147|148|150|185|1128|1129|1812|1813|1814|1910|2099|2147|2149|2915|3062|3356|3357|3358|4985|4986|4988	ADRA1D|ADRA1B|ADRA1A|ADRA2A|AGTR1|CHRM1|CHRM2|DRD1|DRD2|DRD3|EDNRB|ESR1|F2|F2R|GRM5|HCRTR2|HTR2A|HTR2B|HTR2C|OPRD1|OPRK1|OPRM1	-24.7496	0	4	0	0	-38.1485	34.9581757
1	-17.5963	GO:0098662	M1	1	0	GO Biological Processes	19	inorganic cation transmembrane transport	-17.5963	6.825376	13.02095	30242	799	183	33	18.03279	2.842012	150|775|1812|1813|1814|1815|2147|2149|2902|2903|2904|3356|3357|3358|3757|3778|4842|4986|4988|5024|5142|6326|6331|6335|6336|6523|6530|6531|6532|7442|8989|51305|59341	ADRA2A|CACNA1C|DRD1|DRD2|DRD3|DRD4|F2|F2R|GRIN1|GRIN2A|GRIN2B|HTR2A|HTR2B|HTR2C|KCNH2|KCNMA1|NOS1|OPRK1|OPRM1|P2RX3|PDE4B|SCN2A|SCN5A|SCN9A|SCN10A|SLC5A1|SLC6A2|SLC6A3|SLC6A4|TRPV1|TRPA1|KCNK9|TRPV4	-15.4685	0	4	0	0	-38.1485	34.9581757
1	-17.1836	GO:0060402	M1	1	0	GO Biological Processes	19	calcium ion transport into cytosol	-17.1836	18.59139	17.40777	30242	160	183	18	9.836066	2.201413	148|596|775|1812|1813|2147|2149|2902|2903|2904|3356|3357|3358|4842|5024|7442|8989|59341	ADRA1A|BCL2|CACNA1C|DRD1|DRD2|F2|F2R|GRIN1|GRIN2A|GRIN2B|HTR2A|HTR2B|HTR2C|NOS1|P2RX3|TRPV1|TRPA1|TRPV4	-15.0668	0	4	0	0	-38.1485	34.9581757
1	-16.1722	GO:0060401	M1	1	0	GO Biological Processes	19	cytosolic calcium ion transport	-16.1722	16.34408	16.20013	30242	182	183	18	9.836066	2.201413	148|596|775|1812|1813|2147|2149|2902|2903|2904|3356|3357|3358|4842|5024|7442|8989|59341	ADRA1A|BCL2|CACNA1C|DRD1|DRD2|F2|F2R|GRIN1|GRIN2A|GRIN2B|HTR2A|HTR2B|HTR2C|NOS1|P2RX3|TRPV1|TRPA1|TRPV4	-14.1129	0	4	0	0	-38.1485	34.9581757
1	-14.2564	GO:0070588	M1	1	0	GO Biological Processes	19	calcium ion transmembrane transport	-14.2564	10.59339	13.29022	30242	312	183	20	10.92896	2.306387	150|775|1812|1813|1814|1815|2147|2149|2902|2903|2904|3356|3357|3358|4842|4988|5142|7442|8989|59341	ADRA2A|CACNA1C|DRD1|DRD2|DRD3|DRD4|F2|F2R|GRIN1|GRIN2A|GRIN2B|HTR2A|HTR2B|HTR2C|NOS1|OPRM1|PDE4B|TRPV1|TRPA1|TRPV4	-12.301	0	4	0	0	-38.1485	34.9581757
1	-13.9801	GO:0097553	M1	1	0	GO Biological Processes	19	calcium ion transmembrane import into cytosol	-13.9801	17.45671	15.33699	30242	142	183	15	8.196721	2.027793	775|1812|1813|2147|2149|2902|2903|2904|3356|3357|3358|4842|7442|8989|59341	CACNA1C|DRD1|DRD2|F2|F2R|GRIN1|GRIN2A|GRIN2B|HTR2A|HTR2B|HTR2C|NOS1|TRPV1|TRPA1|TRPV4	-12.0394	0	4	0	0	-38.1485	34.9581757
1	-10.7289	GO:0051235	M1	1	0	GO Biological Processes	19	maintenance of location	-10.7289	8.591334	10.76921	30242	327	183	17	9.289617	2.145862	775|1812|1813|2147|2149|3356|3357|3358|3569|4792|4842|5465|5467|	CACNA1C|DRD1|DRD2|F2|F2R|HTR2A|HTR2B|HTR2C|IL6|NFK	-8.99228	0	4	0	0	-38.1485	34.9581757

<table>
<tr><td></td><td></td><td></td><td></td><td></td><td></td><td></td><td></td><td></td><td></td><td></td><td></td><td></td><td></td><td></td><td></td><td></td><td></td><td>5468|7124|7442|8989</td><td>BIA|NOS1|PPARA|PPARD|PPARG|TNF|TRPV1|TRPA1</td><td></td><td></td><td></td><td></td><td></td><td></td><td></td></tr>
<tr><td>1</td><td>-9.91596</td><td>GO:0051209</td><td>M1</td><td>1</td><td>0</td><td>GO Biological Processes</td><td>19</td><td>release of sequestered calcium ion into cytosol</td><td>-9.91596</td><td>15.80718</td><td>12.41308</td><td>30242</td><td>115</td><td>183</td><td>11</td><td>6.010929</td><td>1.757049</td><td>775|1812|1813|2147|2149|3356|3357|3358|4842|7442|8989</td><td>CACNA1C|DRD1|DRD2|F2|F2R|HTR2A|HTR2B|HTR2C|NOS1|TRPV1|TRPA1</td><td>-8.24112</td><td>0</td><td>4</td><td>0</td><td>0</td><td>-38.1485</td><td>34.9581757</td></tr>
<tr><td>1</td><td>-9.87496</td><td>GO:0051283</td><td>M1</td><td>1</td><td>0</td><td>GO Biological Processes</td><td>19</td><td>negative regulation of sequestering of calcium ion</td><td>-9.87496</td><td>15.67091</td><td>12.35241</td><td>30242</td><td>116</td><td>183</td><td>11</td><td>6.010929</td><td>1.757049</td><td>775|1812|1813|2147|2149|3356|3357|3358|4842|7442|8989</td><td>CACNA1C|DRD1|DRD2|F2|F2R|HTR2A|HTR2B|HTR2C|NOS1|TRPV1|TRPA1</td><td>-8.20497</td><td>0</td><td>4</td><td>0</td><td>0</td><td>-38.1485</td><td>34.9581757</td></tr>
<tr><td>1</td><td>-9.79412</td><td>GO:0051282</td><td>M1</td><td>1</td><td>0</td><td>GO Biological Processes</td><td>19</td><td>regulation of sequestering of calcium ion</td><td>-9.79412</td><td>15.4053</td><td>12.23329</td><td>30242</td><td>118</td><td>183</td><td>11</td><td>6.010929</td><td>1.757049</td><td>775|1812|1813|2147|2149|3356|3357|3358|4842|7442|8989</td><td>CACNA1C|DRD1|DRD2|F2|F2R|HTR2A|HTR2B|HTR2C|NOS1|TRPV1|TRPA1</td><td>-8.13089</td><td>0</td><td>4</td><td>0</td><td>0</td><td>-38.1485</td><td>34.9581757</td></tr>
<tr><td>1</td><td>-9.63689</td><td>GO:0051208</td><td>M1</td><td>1</td><td>0</td><td>GO Biological Processes</td><td>19</td><td>sequestering of calcium ion</td><td>-9.63689</td><td>14.90021</td><td>12.00356</td><td>30242</td><td>122</td><td>183</td><td>11</td><td>6.010929</td><td>1.757049</td><td>775|1812|1813|2147|2149|3356|3357|3358|4842|7442|8989</td><td>CACNA1C|DRD1|DRD2|F2|F2R|HTR2A|HTR2B|HTR2C|NOS1|TRPV1|TRPA1</td><td>-7.98249</td><td>0</td><td>4</td><td>0</td><td>0</td><td>-38.1485</td><td>34.9581757</td></tr>
<tr><td>1</td><td>-7.07277</td><td>GO:0051651</td><td>M1</td><td>1</td><td>0</td><td>GO Biological Processes</td><td>19</td><td>maintenance of location in cell</td><td>-7.07277</td><td>8.49451</td><td>8.584664</td><td>30242</td><td>214</td><td>183</td><td>11</td><td>6.010929</td><td>1.757049</td><td>775|1812|1813|2147|2149|3356|3357|3358|4842|7442|8989</td><td>CACNA1C|DRD1|DRD2|F2|F2R|HTR2A|HTR2B|HTR2C|NOS1|TRPV1|TRPA1</td><td>-5.62843</td><td>0</td><td>4</td><td>0</td><td>0</td><td>-38.1485</td><td>34.9581757</td></tr>
<tr><td>1</td><td>-36.2749</td><td>GO:0009410</td><td>M1</td><td>1</td><td>0</td><td>GO Biological Processes</td><td>19</td><td>response to xenobiotic stimulus</td><td>-36.2749</td><td>15.02335</td><td>23.69991</td><td>30242</td><td>462</td><td>183</td><td>42</td><td>22.95082</td><td>3.108548</td><td>100|135|148|328|596|836|1543|1544|1545|1557|1645|1723|1728|1812|1813|1814|1956|2902|2903|3066|3351|3356|3357|3358|3383|3757|4129|4363|4842|4843|4914|5142|5243|5498|5743|5970|5979|6530|6531|6532|7298|8989</td><td>ADA|ADORA2A|ADRA1A|APEX1|BCL2|CASP3|CYP1A1|CYP1A2|CYP1B1|CYP2C19|AKR1C1|DHODH|NQO1|DRD1|DRD2|DRD3|EGFR|GRIN1|GRIN2A|HDAC2|HTR1B|HTR2A|HTR2B|HTR2C|ICAM1|KCNH2|MAOB|ABCC1|NOS1|NOS2|NTRK1|PDE4B|ABCB1|PPOX|PTGS2|RELA|RET|SLC6A2|SLC6A3|SLC6A4|TYMS|TRPA1</td><td>-33.4213</td><td>0</td><td>5</td><td>1</td><td>1</td><td>-36.2749</td><td>15.02334824</td></tr>
<tr><td>1</td><td>-33.6476</td><td>GO:0043269</td><td>M1</td><td>1</td><td>0</td><td>GO Biological Processes</td><td>19</td><td>regulation of ion transport</td><td>-33.6476</td><td>10.93786</td><td>20.68069</td><td>30242</td><td>695</td><td>183</td><td>46</td><td>25.13661</td><td>3.206731</td><td>134|135|150|154|351|596|760|775|1080|1128|1131|1230|1268|1812|1813|1814|1815|2147|2149|2902|2915|3350|3351|3356|3383|3757|3778|4129|4318|4842|4985|4986|4988|5024|5142|5159|5243|5743|6326|6331|6335|6336|6347|6532|11255|729230</td><td>ADORA1|ADORA2A|ADRA2A|ADRB2|APP|BCL2|CA2|CACNA1C|CFTR|CHRM1|CHRM3|CCR1|CNR1|DRD1|DRD2|DRD3|DRD4|F2|F2R|GRIN1|GRM5|HTR1A|HTR1B|HTR2A|ICAM1|KCNH2|KCNMA1|MAOB|MMP9|NOS1|OPRD1|OPRK1|OPRM1|P2RX3|PDE4B|PDGFRB|ABCB1|PT</td><td>-30.9288</td><td>0</td><td>6</td><td>1</td><td>1</td><td>-33.6476</td><td>13.82148038</td></tr>
</table>

<table>
<tr><td></td><td></td><td></td><td></td><td></td><td></td><td></td><td></td><td></td><td></td><td></td><td></td><td></td><td></td><td></td><td></td><td></td><td></td><td></td><td>GS2|SCN2A|SCN5A|SCN9A|SCN10A|CCL2|SLC6A4|HRH3|CCR2</td><td></td><td></td><td></td><td></td><td></td><td></td><td></td></tr>
<tr><td>1</td><td>-26.2081</td><td>GO:0006816</td><td>M1</td><td>1</td><td>0</td><td>GO Biological Processes</td><td>19</td><td>calcium ion transport</td><td>-26.2081</td><td>12.92293</td><td>19.24517</td><td>30242</td><td>422</td><td>183</td><td>33</td><td>18.03279</td><td>2.842012</td><td>135|148|150|596|775|1230|1812|1813|1814|1815|2147|2149|2902|2903|2904|3356|3357|3358|3383|4842|4985|4988|5024|5142|5159|5743|6347|7421|7442|8989|10203|10800|59341</td><td>ADORA2A|ADRA1A|ADRA2A|BCL2|CACNA1C|CCR1|DRD1|DRD2|DRD3|DRD4|F2|F2R|GRIN1|GRIN2A|GRIN2B|HTR2A|HTR2B|HTR2C|ICAM1|NOS1|OPRD1|OPRM1|P2RX3|PDE4B|PDGFRB|PTGS2|CCL2|VDR|TRPV1|TRPA1|CALCRL|CYSLTR1|TRPV4</td><td>-23.7364</td><td>0</td><td>6</td><td>0</td><td>0</td><td>-33.6476</td><td>13.82148038</td></tr>
<tr><td>1</td><td>-21.6006</td><td>GO:0034762</td><td>M1</td><td>1</td><td>0</td><td>GO Biological Processes</td><td>19</td><td>regulation of transmembrane transport</td><td>-21.6006</td><td>9.243179</td><td>15.77721</td><td>30242</td><td>590</td><td>183</td><td>33</td><td>18.03279</td><td>2.842012</td><td>150|154|351|596|673|760|775|1080|1131|1812|1813|1814|1815|1909|2147|2149|2915|3643|3757|3778|4318|4842|4986|4988|5142|5243|6326|6331|6335|6336|6347|7124|729230</td><td>ADRA2A|ADRB2|APP|BCL2|BRAF|CA2|CACNA1C|CFTR|CHRM3|DRD1|DRD2|DRD3|DRD4|EDNRA|F2|F2R|GRM5|INSR|KCNH2|KCNMA1|MMP9|NOS1|OPRK1|OPRM1|PDE4B|ABCB1|SCN2A|SCN5A|SCN9A|SCN10A|CCL2|TNF|CCR2</td><td>-19.3237</td><td>0</td><td>6</td><td>0</td><td>0</td><td>-33.6476</td><td>13.82148038</td></tr>
<tr><td>1</td><td>-18.9178</td><td>GO:0043270</td><td>M1</td><td>1</td><td>0</td><td>GO Biological Processes</td><td>19</td><td>positive regulation of ion transport</td><td>-18.9178</td><td>13.82148</td><td>16.66553</td><td>30242</td><td>275</td><td>183</td><td>23</td><td>12.56831</td><td>2.450458</td><td>134|135|150|154|1080|1128|1230|1812|1813|1815|2147|2149|2902|3757|4842|4986|5024|5159|5243|6331|6347|6532|729230</td><td>ADORA1|ADORA2A|ADRA2A|ADRB2|CFTR|CHRM1|CCR1|DRD1|DRD2|DRD4|F2|F2R|GRIN1|KCNH2|NOS1|OPRK1|P2RX3|PDGFRB|ABCB1|SCN5A|CCL2|SLC6A4|CCR2</td><td>-16.713</td><td>0</td><td>6</td><td>0</td><td>0</td><td>-33.6476</td><td>13.82148038</td></tr>
<tr><td>1</td><td>-18.4481</td><td>GO:0010959</td><td>M1</td><td>1</td><td>0</td><td>GO Biological Processes</td><td>19</td><td>regulation of metal ion transport</td><td>-18.4481</td><td>10.58295</td><td>15.168</td><td>30242</td><td>406</td><td>183</td><td>26</td><td>14.20765</td><td>2.580833</td><td>134|135|150|154|596|775|1230|1812|1813|1814|1815|2147|2149|2902|3356|3383|3757|4842|4985|4986|5024|5142|5159|5743|6331|6347</td><td>ADORA1|ADORA2A|ADRA2A|ADRB2|BCL2|CACNA1C|CCR1|DRD1|DRD2|DRD3|DRD4|F2|F2R|GRIN1|HTR2A|ICAM1|KCNH2|NOS1|OPRD1|OPRK1|P2RX3|PDE4B|PDGFRB|PTGS2|SCN5A|CCL2</td><td>-16.2774</td><td>0</td><td>6</td><td>0</td><td>0</td><td>-33.6476</td><td>13.82148038</td></tr>
<tr><td>1</td><td>-17.4673</td><td>GO:0034765</td><td>M1</td><td>1</td><td>0</td><td>GO Biological Processes</td><td>19</td><td>regulation of ion transmembrane transport</td><td>-17.4673</td><td>9.087443</td><td>14.09735</td><td>30242</td><td>491</td><td>183</td><td>27</td><td>14.7541</td><td>2.621607</td><td>150|154|351|775|1080|1131|1812|1813|1814|1815|2147|2149|2915|3757|3778|4318|4842|4986|4988|5142|5243|6326|6331|6335|6336|6347|729230</td><td>ADRA2A|ADRB2|APP|CACNA1C|CFTR|CHRM3|DRD1|DRD2|DRD3|DRD4|F2|F2R|GRM5|KCNH2|KCNMA1|MMP9|NOS1|OPRK1|OPRM1|PDE4B|ABCB</td><td>-15.3432</td><td>0</td><td>6</td><td>0</td><td>0</td><td>-33.6476</td><td>13.82148038</td></tr>
</table>

																			1|SCN2A|SCN5A|SCN9A|SCN10A|CCL2|CCR2							
1	-15.9426	GO:0051924	M1	1	0	GO Biological Processes	19	regulation of calcium ion transport	-15.9426	12.96132	14.96646	30242	255	183	20	10.92896	2.306387	135|150|596|775|1230|1812|1813|1814|1815|2147|2149|2902|3383|4842|4985|5024|5142|5159|5743|6347	ADORA2A|ADRA2A|BCL2|CACNA1C|CCR1|DRD1|DRD2|DRD3|DRD4|F2|F2R|GRIN1|ICAM1|NOS1|OPRD1|P2RX3|PDE4B|PDGFRB|PTGS2|CCL2	-13.908	0	6	0	0	-33.6476	13.82148038
1	-13.2114	GO:0032409	M1	1	0	GO Biological Processes	19	regulation of transporter activity	-13.2114	10.12864	12.60537	30242	310	183	19	10.38251	2.254873	150|154|351|596|1080|1131|1813|1814|1815|2915|4318|4842|4988|5142|5243|5444|5468|6347|729230	ADRA2A|ADRB2|APP|BCL2|CFTR|CHRM3|DRD2|DRD3|DRD4|GRM5|MMP9|NOS1|OPRM1|PDE4B|ABCB1|PON1|PPARG|CCL2|CCR2	-11.3142	0	6	0	0	-33.6476	13.82148038
1	-12.1199	GO:1904062	M1	1	0	GO Biological Processes	19	regulation of cation transmembrane transport	-12.1199	8.795181	11.56033	30242	357	183	19	10.38251	2.254873	150|154|351|775|1812|1813|1814|1815|2147|2149|3757|4318|4842|4986|4988|5142|6331|6347|729230	ADRA2A|ADRB2|APP|CACNA1C|DRD1|DRD2|DRD3|DRD4|F2|F2R|KCNH2|MMP9|NOS1|OPRK1|OPRM1|PDE4B|SCN5A|CCL2|CCR2	-10.2875	0	6	0	0	-33.6476	13.82148038
1	-11.8494	GO:0022898	M1	1	0	GO Biological Processes	19	regulation of transmembrane transporter activity	-11.8494	10.10563	11.9006	30242	278	183	17	9.289617	2.145862	150|154|351|596|1080|1131|1813|1814|1815|2915|4318|4842|4988|5142|5243|6347|729230	ADRA2A|ADRB2|APP|BCL2|CFTR|CHRM3|DRD2|DRD3|DRD4|GRM5|MMP9|NOS1|OPRM1|PDE4B|ABCB1|CCL2|CCR2	-10.0336	0	6	0	0	-33.6476	13.82148038
1	-11.213	GO:0034764	M1	1	0	GO Biological Processes	19	positive regulation of transmembrane transport	-11.213	11.31896	11.95824	30242	219	183	15	8.196721	2.027793	154|673|760|1080|1812|1815|2147|2149|3643|3757|4842|4986|5243|6347|729230	ADRB2|BRAF|CA2|CFTR|DRD1|DRD4|F2|F2R|INSR|KCNH2|NOS1|OPRK1|ABCB1|CCL2|CCR2	-9.43866	0	6	0	0	-33.6476	13.82148038
1	-11.0467	GO:0032412	M1	1	0	GO Biological Processes	19	regulation of ion transmembrane transporter activity	-11.0467	9.903031	11.40119	30242	267	183	16	8.743169	2.088053	150|154|351|1080|1131|1813|1814|1815|2915|4318|4842|4988|5142|5243|6347|729230	ADRA2A|ADRB2|APP|CFTR|CHRM3|DRD2|DRD3|DRD4|GRM5|MMP9|NOS1|OPRM1|PDE4B|ABCB1|CCL2|CCR2	-9.29007	0	6	0	0	-33.6476	13.82148038
1	-9.3128	GO:0034767	M1	1	0	GO Biological Processes	19	positive regulation of ion transmembrane transport	-9.3128	11.87474	10.99539	30242	167	183	12	6.557377	1.829835	154|1080|1812|1815|2147|2149|3757|4842|4986|5243|6347|729230	ADRB2|CFTR|DRD1|DRD4|F2|F2R|KCNH2|NOS1|OPRK1|ABCB1|CCL2|CCR2	-7.67673	0	6	0	0	-33.6476	13.82148038
1	-7.74518	GO:2001257	M1	1	0	GO Biological Processes	19	regulation of cation channel activity	-7.74518	9.879484	9.426554	30242	184	183	11	6.010929	1.757049	154|351|1813|1814|1815|4318|4842|4988|5142|6347|729230	ADRB2|APP|DRD2|DRD3|DRD4|MMP9|NOS1|OPRM1|PDE4B|CCL2|CCR2	-6.23405	0	6	0	0	-33.6476	13.82148038
1	-7.51091	GO:1904064	M1	1	0	GO Biological Processes	19	positive regulation of cation transmembrane	-7.51091	10.94416	9.558168	30242	151	183	10	5.464481	1.680144	154|1812|1815|2147|2149|3757|4842|4986|634	ADRB2|DRD1|DRD4|F2|F2R|KCNH2|NOS1|	-6.0225	0	6	0	0	-33.6476	13.82148038

								transport										7|729230	OPRK1|CCL2|CCR2							
1	-5.05265	GO:0032411	M1	1	0	GO Biological Processes	19	positive regulation of transporter activity	-5.05265	9.639982	7.399424	30242	120	183	7	3.825137	1.417845	154|1080|1815|5243|5444|6347|729230	ADRB2|CFTR|DRD4|ABCB1|PON1|CCL2|CCR2	-3.81091	0	6	0	0	-33.6476	13.82148038
1	-4.26237	GO:0032414	M1	1	0	GO Biological Processes	19	positive regulation of ion transmembrane transporter activity	-4.26237	9.096706	6.607494	30242	109	183	6	3.278689	1.316393	154|1080|1815|5243|6347|729230	ADRB2|CFTR|DRD4|ABCB1|CCL2|CCR2	-3.12258	0	6	0	0	-33.6476	13.82148038
1	-32.5748	GO:0000165	M1	1	0	GO Biological Processes	19	MAPK cascade	-32.5748	9.957783	19.77667	30242	780	183	47	25.68306	3.229548	136|146|147|148|150|151|152|154|351|367|673|1230|1813|1815|1956|1991|2149|2263|2335|3061|3156|3356|3357|3358|3383|3551|3569|3643|4221|4914|4915|4986|4988|5159|5291|5468|5734|5979|6347|7040|7046|7099|7124|9475|59340|59341|225689	ADORA2B|ADRA1D|ADRA1B|ADRA1A|ADRA2A|ADRA2B|ADRA2C|ADRB2|APP|AR|BRAF|CCR1|DRD2|DRD4|EGFR|ELANE|F2R|FGFR2|FN1|HCRTR1|HMGCR|HTR2A|HTR2B|HTR2C|ICAM1|IKBKB|IL6|INSR|MEN1|NTRK1|NTRK2|OPRK1|OPRM1|PDGFRB|PIK3CB|PPARG|PTGER4|RET|CCL2|TGFB1|TGFBR1|TLR4|TNF|ROCK2|HRH4|TRPV4|MAPK15	-29.8839	0	7	1	1	-32.5748	17.19867468
1	-31.5979	GO:0001934	M1	1	0	GO Biological Processes	19	positive regulation of protein phosphorylation	-31.5979	10.21505	19.63748	30242	728	183	45	24.59016	3.183238	134|148|150|151|152|154|351|595|596|673|1453|1636|1813|1815|1910|1956|1991|2147|2915|3066|3356|3357|3383|3558|3569|3643|4318|4842|4914|4915|4985|5159|5290|5468|5743|5979|7040|7046|7099|7124|7132|7157|8986|9475|29110	ADORA1|ADRA1A|ADRA2A|ADRA2B|ADRA2C|ADRB2|APP|CCND1|BCL2|BRAF|CSNK1D|ACE|DRD2|DRD4|EDNRB|EGFR|ELANE|F2|GRM5|HDAC2|HTR2A|HTR2B|ICAM1|IL2|IL6|INSR|MMP9|NOS1|NTRK1|NTRK2|OPRD1|PDGFRB|PIK3CA|PPARG|PTGS2|RET|TGFB1|TGFBR1|TLR4|TNF|TNFRSF1A|TP53|RPS6KA4|ROCK2|TBK1	-28.946	0	7	0	0	-32.5748	17.19867468
1	-30.5399	GO:0043408	M1	1	0	GO Biological Processes	19	regulation of MAPK cascade	-30.5399	10.45006	19.44727	30242	680	183	43	23.49727	3.134163	146|147|148|150|151|152|154|351|367|673|1230|1813|1815|1956|1991|2149|2263|2335|3061|3156|3356|3357|3358|3383|3569|3643|4221|4914|4915|4986|4988|5159|5291|5468|5979|6347|7040|7046|7099|7124|9475|59340|59341	ADRA1D|ADRA1B|ADRA1A|ADRA2A|ADRA2B|ADRA2C|ADRB2|APP|AR|BRAF|CCR1|DRD2|DRD4|EGFR|ELANE|F2R|FGFR2|FN1|HCRTR1|HMGCR|HTR2A|HTR2B|HTR2C|ICAM1|IL6|INSR|MEN1|NTRK1|NTRK2|OPRK1|OPRM1|PDGF	-27.9121	0	7	0	0	-32.5748	17.19867468

																			RB|PIK3CB|PPARG|RET|CCL2|TGFB1|TGFBR1|TLR4|TNF|ROCK2|HRH4|TRPV4							
1	-30.3988	GO:0043410	M1	1	0	GO Biological Processes	19	positive regulation of MAPK cascade	-30.3988	13.02855	20.77096	30242	482	183	38	20.76503	2.998466	146|147|148|150|151|152|154|351|367|673|1230|1813|1815|1956|1991|2149|2263|3061|3156|3356|3357|3358|3383|3569|3643|4914|4915|4986|4988|5159|5979|6347|7040|7046|7099|7124|9475|59341	ADRA1D|ADRA1B|ADRA1A|ADRA2A|ADRA2B|ADRA2C|ADRB2|APP|AR|BRAF|CCR1|DRD2|DRD4|EGFR|ELANE|F2R|FGFR2|HCRTR1|HMGCR|HTR2A|HTR2B|HTR2C|ICAM1|IL6|INSR|NTRK1|NTRK2|OPRK1|OPRM1|PDGFRB|RET|CCL2|TGFB1|TGFBR1|TLR4|TNF|ROCK2|TRPV4	-27.7826	0	7	0	0	-32.5748	17.19867468
1	-21.2201	GO:0043549	M1	1	0	GO Biological Processes	19	regulation of kinase activity	-21.2201	7.848609	14.89979	30242	758	183	36	19.67213	2.938551	134|135|148|150|151|152|154|351|595|836|890|1636|1813|1815|1956|1991|2050|2147|2263|2915|3156|3356|3357|3558|3643|4221|4914|4915|5159|5290|5291|5468|5979|7040|7099|7124	ADORA1|ADORA2A|ADRA1A|ADRA2A|ADRA2B|ADRA2C|ADRB2|APP|CCND1|CASP3|CCNA2|ACE|DRD2|DRD4|EGFR|ELANE|EPHB4|F2|FGFR2|GRM5|HMGCR|HTR2A|HTR2B|IL2|INSR|MEN1|NTRK1|NTRK2|PDGFRB|PIK3CA|PIK3CB|PPARG|RET|TGFB1|TLR4|TNF	-18.9535	0	7	0	0	-32.5748	17.19867468
1	-21.0855	GO:0070374	M1	1	0	GO Biological Processes	19	positive regulation of ERK1 and ERK2 cascade	-21.0855	17.19867	18.85816	30242	221	183	23	12.56831	2.450458	148|351|673|1230|1813|1956|2149|2263|3061|3156|3356|3357|3358|3383|4914|4915|4988|5159|6347|7040|7099|7124|59341	ADRA1A|APP|BRAF|CCR1|DRD2|EGFR|F2R|FGFR2|HCRTR1|HMGCR|HTR2A|HTR2B|HTR2C|ICAM1|NTRK1|NTRK2|OPRM1|PDGFRB|CCL2|TGFB1|TLR4|TNF|TRPV4	-18.8241	0	7	0	0	-32.5748	17.19867468
1	-19.2775	GO:0070371	M1	1	0	GO Biological Processes	19	ERK1 and ERK2 cascade	-19.2775	12.25941	16.21791	30242	337	183	25	13.6612	2.538762	148|351|673|1230|1813|1956|2149|2263|2335|3061|3156|3356|3357|3358|3383|4914|4915|4988|5159|5734|6347|7040|7099|7124|59341	ADRA1A|APP|BRAF|CCR1|DRD2|EGFR|F2R|FGFR2|FN1|HCRTR1|HMGCR|HTR2A|HTR2B|HTR2C|ICAM1|NTRK1|NTRK2|OPRM1|PDGFRB|PTGER4|CCL2|TGFB1|TLR4|TNF|TRPV4	-17.0592	0	7	0	0	-32.5748	17.19867468
1	-19.0536	GO:0033674	M1	1	0	GO Biological Processes	19	positive regulation of kinase activity	-19.0536	9.866079	15.09882	30242	469	183	28	15.30055	2.661143	134|148|150|151|152|154|595|1636|1813|1815|1956|1991|2050|2147|22	ADORA1|ADRA1A|ADRA2A|ADRA2B|ADRA2C|ADRB2|CCND1	-16.8444	0	7	0	0	-32.5748	17.19867468

																		63|2915|3356|3357|3558|3643|4914|4915|5159|5290|5979|7040|7099|7124	|ACE|DRD2|DRD4|EGFR|ELANE|EPHB4|F2|FGFR2|GRM5|HTR2A|HTR2B|IL2|INSR|NTRK1|NTRK2|PDGFRB|PIK3CA|RET|TGFB1|TLR4|TNF							
1	-18.7826	GO:0070372	M1	1	0	GO Biological Processes	19	regulation of ERK1 and ERK2 cascade	-18.7826	12.591	16.13544	30242	315	183	24	13.11475	2.495327	148|351|673|1230|1813|1956|2149|2263|2335|3061|3156|3356|3357|3358|3383|4914|4915|4988|5159|6347|7040|7099|7124|59341	ADRA1A|APP|BRAF|CCR1|DRD2|EGFR|F2R|FGFR2|FN1|HCRTR1|HMGCR|HTR2A|HTR2B|HTR2C|ICAM1|NTRK1|NTRK2|OPRM1|PDGFRB|CCL2|TGFB1|TLR4|TNF|TRPV4	-16.591	0	7	0	0	-32.5748	17.19867468
1	-18.2237	GO:0051347	M1	1	0	GO Biological Processes	19	positive regulation of transferase activity	-18.2237	8.681971	14.2126	30242	552	183	29	15.84699	2.699496	134|148|150|151|152|154|595|1636|1813|1815|1956|1991|2050|2147|2263|2915|3356|3357|3558|3643|4914|4915|5159|5290|5979|7040|7099|7124|225689	ADORA1|ADRA1A|ADRA2A|ADRA2B|ADRA2C|ADRB2|CCND1|ACE|DRD2|DRD4|EGFR|ELANE|EPHB4|F2|FGFR2|GRM5|HTR2A|HTR2B|IL2|INSR|NTRK1|NTRK2|PDGFRB|PIK3CA|RET|TGFB1|TLR4|TNF|MAPK15	-16.0691	0	7	0	0	-32.5748	17.19867468
1	-16.5609	GO:0045859	M1	1	0	GO Biological Processes	19	regulation of protein kinase activity	-16.5609	7.523466	12.9838	30242	637	183	29	15.84699	2.699496	134|135|148|150|151|152|154|351|595|836|890|1636|1813|1815|1956|1991|2915|3156|3356|3357|3643|4221|5159|5290|5291|5468|7040|7099|7124	ADORA1|ADORA2A|ADRA1A|ADRA2A|ADRA2B|ADRA2C|ADRB2|APP|CCND1|CASP3|CCNA2|ACE|DRD2|DRD4|EGFR|ELANE|GRM5|HMGCR|HTR2A|HTR2B|INSR|MEN1|PDGFRB|PIK3CA|PIK3CB|PPARG|TGFB1|TLR4|TNF	-14.4687	0	7	0	0	-32.5748	17.19867468
1	-15.9051	GO:0018108	M1	1	0	GO Biological Processes	19	peptidyl-tyrosine phosphorylation	-15.9051	10.1088	13.86764	30242	376	183	23	12.56831	2.450458	134|148|150|351|1636|1956|2050|2263|2915|3066|3356|3383|3558|3569|3643|4914|4915|5159|5979|7040|7124|7132|7157	ADORA1|ADRA1A|ADRA2A|APP|ACE|EGFR|EPHB4|FGFR2|GRM5|HDAC2|HTR2A|ICAM1|IL2|IL6|INSR|NTRK1|NTRK2|PDGFRB|RET|TGFB1|TNF|TNFRSF1A|TP53	-13.8734	0	7	0	0	-32.5748	17.19867468
1	-15.83	GO:0018212	M1	1	0	GO Biological Processes	19	peptidyl-tyrosine modification	-15.83	10.02878	13.80124	30242	379	183	23	12.56831	2.450458	134|148|150|351|1636|1956|2050|2263|2915|3066|3356|3383|3558|3569|3643|4914|4915|5159|5979|7040|7124|7132|7157	ADORA1|ADRA1A|ADRA2A|APP|ACE|EGFR|EPHB4|FGFR2|GRM5|HDAC2|HTR2A|ICAM1|IL2|IL6|INSR|NTRK1|NTRK2|PDGFRB|RET|TGFB1|TNF|TNF	-13.8042	0	7	0	0	-32.5748	17.19867468

																			RSF1A|TP53							
1	-13.4993	GO:0045860	M1	1	0	GO Biological Processes	19	positive regulation of protein kinase activity	-13.4993	8.944313	12.28874	30242	388	183	21	11.47541	2.356083	134|148|150|151|152|154|595|1636|1813|1815|1956|1991|2915|3356|3357|3643|5159|5290|7040|7099|7124	ADORA1|ADRA1A|ADRA2A|ADRA2B|ADRA2C|ADRB2|CCND1|ACE|DRD2|DRD4|EGFR|ELANE|GRM5|HTR2A|HTR2B|INSR|PDGFRB|PIK3CA|TGFB1|TLR4|TNF	-11.5866	0	7	0	0	-32.5748	17.19867468
1	-11.9923	GO:0071900	M1	1	0	GO Biological Processes	19	regulation of protein serine/threonine kinase activity	-11.9923	8.649807	11.44082	30242	363	183	19	10.38251	2.254873	150|154|595|836|890|1815|1956|1991|3156|3356|3357|3643|4221|5159|5291|5468|7040|7099|7124	ADRA2A|ADRB2|CCND1|CASP3|CCNA2|DRD4|EGFR|ELANE|HMGCR|HTR2A|HTR2B|INSR|MEN1|PDGFRB|PIK3CB|PPARG|TGFB1|TLR4|TNF	-10.1692	0	7	0	0	-32.5748	17.19867468
1	-11.211	GO:0043405	M1	1	0	GO Biological Processes	19	regulation of MAP kinase activity	-11.211	12.7823	12.4052	30242	181	183	14	7.650273	1.964856	150|1815|1956|1991|3156|3356|3357|3643|5159|5291|5468|7040|7099|7124	ADRA2A|DRD4|EGFR|ELANE|HMGCR|HTR2A|HTR2B|INSR|PDGFRB|PIK3CB|PPARG|TGFB1|TLR4|TNF	-9.43822	0	7	0	0	-32.5748	17.19867468
1	-11.1207	GO:0050730	M1	1	0	GO Biological Processes	19	regulation of peptidyl-tyrosine phosphorylation	-11.1207	10.01557	11.47968	30242	264	183	16	8.743169	2.088053	134|148|150|351|1636|1956|2915|3066|3356|3383|3558|3569|7040|7124|7132|7157	ADORA1|ADRA1A|ADRA2A|APP|ACE|EGFR|GRM5|HDAC2|HTR2A|ICAM1|IL2|IL6|TGFB1|TNF|TNFRSF1A|TP53	-9.35288	0	7	0	0	-32.5748	17.19867468
1	-10.8337	GO:0050731	M1	1	0	GO Biological Processes	19	positive regulation of peptidyl-tyrosine phosphorylation	-10.8337	11.98754	11.94815	30242	193	183	14	7.650273	1.964856	134|148|150|1636|2915|3066|3356|3383|3558|3569|7040|7124|7132|7157	ADORA1|ADRA1A|ADRA2A|ACE|GRM5|HDAC2|HTR2A|ICAM1|IL2|IL6|TGFB1|TNF|TNFRSF1A|TP53	-9.09254	0	7	0	0	-32.5748	17.19867468
1	-9.95736	GO:0043406	M1	1	0	GO Biological Processes	19	positive regulation of MAP kinase activity	-9.95736	15.94583	12.47452	30242	114	183	11	6.010929	1.757049	150|1815|1956|1991|3356|3357|3643|5159|7040|7099|7124	ADRA2A|DRD4|EGFR|ELANE|HTR2A|HTR2B|INSR|PDGFRB|TGFB1|TLR4|TNF	-8.27459	0	7	0	0	-32.5748	17.19867468
1	-9.45027	GO:0071902	M1	1	0	GO Biological Processes	19	positive regulation of protein serine/threonine kinase activity	-9.45027	10.63534	10.72085	30242	202	183	13	7.103825	1.898976	150|154|595|1815|1956|1991|3356|3357|3643|5159|7040|7099|7124	ADRA2A|ADRB2|CCND1|DRD4|EGFR|ELANE|HTR2A|HTR2B|INSR|PDGFRB|TGFB1|TLR4|TNF	-7.80817	0	7	0	0	-32.5748	17.19867468
1	-29.1442	GO:0032496	M1	1	0	GO Biological Processes	19	response to lipopolysaccharide	-29.1442	15.89935	21.65321	30242	343	183	33	18.03279	2.842012	249|834|836|1268|1543|1544|1910|1991|2149|2263|3066|3383|3569|3576|4129|4792|4842|4843|4846|4986|4988|5142|5467|5734|5743|5970|6197|6347|6401|7040|7099|7124|114548	ALPL|CASP1|CASP3|CNR1|CYP1A1|CYP1A2|EDNRB|ELANE|F2R|FGFR2|HDAC2|ICAM1|IL6|CXCL8|MAOB|NFKBIA|NOS1|NOS2|NOS3|OPRK1|OPRM1|PDE4B|PPARD|PTGER4|PTGS2|RELA|RPS6KA3|CCL2|SELE|	-26.5503	0	8	1	1	-29.1442	15.89934522

<table>
<tr><th></th><th></th><th></th><th></th><th></th><th></th><th></th><th></th><th></th><th></th><th></th><th></th><th></th><th></th><th></th><th></th><th></th><th></th><th></th><th></th><th></th><th></th><th></th><th></th><th></th><th></th><th></th></tr>
<tr><td></td><td></td><td></td><td></td><td></td><td></td><td></td><td></td><td></td><td></td><td></td><td></td><td></td><td></td><td></td><td></td><td></td><td></td><td></td><td>TGFB1|TLR4|TNF|NLRP3</td><td></td><td></td><td></td><td></td><td></td><td></td><td></td></tr>
<tr><td>1</td><td>-28.3351</td><td>GO:0002237</td><td>M1</td><td>1</td><td>0</td><td>GO Biological Processes</td><td>19</td><td>response to molecule of bacterial origin</td><td>-28.3351</td><td>15.02335</td><td>20.97289</td><td>30242</td><td>363</td><td>183</td><td>33</td><td>18.03279</td><td>2.842012</td><td>249|834|836|1268|1543|1544|1910|1991|2149|2263|3066|3383|3569|3576|4129|4792|4842|4843|4846|4986|4988|5142|5467|5734|5743|5970|6197|6347|6401|7040|7099|7124|114548</td><td>ALPL|CASP1|CASP3|CNR1|CYP1A1|CYP1A2|EDNRB|ELANE|F2R|FGFR2|HDAC2|ICAM1|IL6|CXCL8|MAOB|NFKBIA|NOS1|NOS2|NOS3|OPRK1|OPRM1|PDE4B|PPARD|PTGER4|PTGS2|RELA|RPS6KA3|CCL2|SELE|TGFB1|TLR4|TNF|NLRP3</td><td>-25.7725</td><td>0</td><td>8</td><td>0</td><td>0</td><td>-29.1442</td><td>15.89934522</td></tr>
<tr><td>1</td><td>-22.4093</td><td>GO:0009617</td><td>M1</td><td>1</td><td>0</td><td>GO Biological Processes</td><td>19</td><td>response to bacterium</td><td>-22.4093</td><td>8.163555</td><td>15.48982</td><td>30242</td><td>749</td><td>183</td><td>37</td><td>20.21858</td><td>2.968935</td><td>249|761|834|836|1268|1543|1544|1910|1991|2147|2149|2263|3066|3383|3569|3576|4129|4792|4842|4843|4846|4986|4988|5142|5467|5734|5743|5970|6197|6347|6401|7040|7099|7124|7132|29110|114548</td><td>ALPL|CA3|CASP1|CASP3|CNR1|CYP1A1|CYP1A2|EDNRB|ELANE|F2|F2R|FGFR2|HDAC2|ICAM1|IL6|CXCL8|MAOB|NFKBIA|NOS1|NOS2|NOS3|OPRK1|OPRM1|PDE4B|PPARD|PTGER4|PTGS2|RELA|RPS6KA3|CCL2|SELE|TGFB1|TLR4|TNF|TNFRSF1A|TBK1|NLRP3</td><td>-20.1054</td><td>0</td><td>8</td><td>0</td><td>0</td><td>-29.1442</td><td>15.89934522</td></tr>
<tr><td>1</td><td>-21.9248</td><td>GO:0071396</td><td>M1</td><td>1</td><td>0</td><td>GO Biological Processes</td><td>19</td><td>cellular response to lipid</td><td>-21.9248</td><td>9.467839</td><td>16.00998</td><td>30242</td><td>576</td><td>183</td><td>33</td><td>18.03279</td><td>2.842012</td><td>367|472|834|890|1080|1645|1910|1956|2099|2100|2263|2908|3066|3383|3569|3576|4792|4843|4986|5142|5241|5465|5467|5970|5979|6347|6532|7040|7099|7124|7421|9475|114548</td><td>AR|ATM|CASP1|CCNA2|CFTR|AKR1C1|EDNRB|EGFR|ESR1|ESR2|FGFR2|NR3C1|HDAC2|ICAM1|IL6|CXCL8|NFKBIA|NOS2|OPRK1|PDE4B|PGR|PPARA|PPARD|RELA|RET|CCL2|SLC6A4|TGFB1|TLR4|TNF|VDR|ROCK2|NLRP3</td><td>-19.6319</td><td>0</td><td>8</td><td>0</td><td>0</td><td>-29.1442</td><td>15.89934522</td></tr>
<tr><td>1</td><td>-12.7086</td><td>GO:0071216</td><td>M1</td><td>1</td><td>0</td><td>GO Biological Processes</td><td>19</td><td>cellular response to biotic stimulus</td><td>-12.7086</td><td>11.42019</td><td>12.80406</td><td>30242</td><td>246</td><td>183</td><td>17</td><td>9.289617</td><td>2.145862</td><td>834|1910|3383|3569|3576|4792|4843|4986|5142|5467|5970|6347|7040|7099|7124|7157|114548</td><td>CASP1|EDNRB|ICAM1|IL6|CXCL8|NFKBIA|NOS2|OPRK1|PDE4B|PPARD|RELA|CCL2|TGFB1|TLR4|TNF|TP53|NLRP3</td><td>-10.8389</td><td>0</td><td>8</td><td>0</td><td>0</td><td>-29.1442</td><td>15.89934522</td></tr>
<tr><td>1</td><td>-12.6736</td><td>GO:0071222</td><td>M1</td><td>1</td><td>0</td><td>GO Biological Processes</td><td>19</td><td>cellular response to lipopolysaccharide</td><td>-12.6736</td><td>12.65124</td><td>13.1881</td><td>30242</td><td>209</td><td>183</td><td>16</td><td>8.743169</td><td>2.088053</td><td>834|1910|3383|3569|3576|4792|4843|4986|5142|5467|5970|6347|7040|7099|7124|114548</td><td>CASP1|EDNRB|ICAM1|IL6|CXCL8|NFKBIA|NOS2|OPRK1|PDE4B|PPARD|RELA|CCL2|TGFB1|TLR4|TNF|NLRP3</td><td>-10.81</td><td>0</td><td>8</td><td>0</td><td>0</td><td>-29.1442</td><td>15.89934522</td></tr>
<tr><td>1</td><td>-12.2986</td><td>GO:0071219</td><td>M1</td><td>1</td><td>0</td><td>GO Biological</td><td>19</td><td>cellular response to</td><td>-12.2986</td><td>11.9643</td><td>12.76441</td><td>30242</td><td>221</td><td>183</td><td>16</td><td>8.743169</td><td>2.088053</td><td>834|1910|3383|3569|35</td><td>CASP1|EDNRB|ICAM</td><td>-10.4529</td><td>0</td><td>8</td><td>0</td><td>0</td><td>-29.1442</td><td>15.89934522</td></tr>
</table>

						Processes		molecule of bacterial origin										76|4792|4843|4986|5142|5467|5970|6347|7040|7099|7124|114548	1|IL6|CXCL8|NFKBIA|NOS2|OPRK1|PDE4B|PPARD|RELA|CCL2|TGFB1|TLR4|TNF|NLRP3							
1	-27.7508	GO:0097305	M1	1	0	GO Biological Processes	19	response to alcohol	-27.7508	18.86791	22.31275	30242	254	183	29	15.84699	2.699496	595|761|1080|1268|1728|1813|1814|1815|2155|2263|2902|2903|2904|3066|3156|3351|3383|3558|4129|4986|4988|5465|5734|6531|6916|7040|7046|7298|114548	CCND1|CA3|CFTR|CNR1|NQO1|DRD2|DRD3|DRD4|F7|FGFR2|GRIN1|GRIN2A|GRIN2B|HDAC2|HMGCR|HTR1B|ICAM1|IL2|MAOB|OPRK1|OPRM1|PPARA|PTGER4|SLC6A3|TBXAS1|TGFB1|TGFBR1|TYMS|NLRP3	-25.1983	0	9	1	1	-27.7508	28.74031837
1	-27.5261	GO:0045471	M1	1	0	GO Biological Processes	19	response to ethanol	-27.5261	28.74032	25.48445	30242	138	183	24	13.11475	2.495327	595|761|1268|1728|1813|1814|1815|2263|2902|2903|2904|3066|3156|3351|3383|3558|4129|4986|4988|5465|6531|6916|7298|114548	CCND1|CA3|CNR1|NQO1|DRD2|DRD3|DRD4|FGFR2|GRIN1|GRIN2A|GRIN2B|HDAC2|HMGCR|HTR1B|ICAM1|IL2|MAOB|OPRK1|OPRM1|PPARA|SLC6A3|TBXAS1|TYMS|NLRP3	-25.0022	0	9	0	0	-27.7508	28.74031837
1	-27.7221	GO:0015844	M1	1	0	GO Biological Processes	19	monoamine transport	-27.7221	41.31421	28.86907	30242	84	183	21	11.47541	2.356083	135|150|151|152|1133|1268|1812|1813|1814|1815|3350|3351|3356|4129|4842|4986|6530|6531|6532|11255|225689	ADORA2A|ADRA2A|ADRA2B|ADRA2C|CHRM5|CNR1|DRD1|DRD2|DRD3|DRD4|HTR1A|HTR1B|HTR2A|MAOB|NOS1|OPRK1|SLC6A2|SLC6A3|SLC6A4|HRH3|MAPK15	-25.1793	0	10	1	1	-27.7221	91.80935033
1	-26.3305	GO:0015850	M1	1	0	GO Biological Processes	19	organic hydroxy compound transport	-26.3305	16.87482	20.97239	30242	284	183	29	15.84699	2.699496	135|150|151|152|185|1080|1133|1268|1588|1645|1812|1813|1814|1815|3350|3351|3356|4129|4792|4842|4986|5444|5468|6523|6530|6531|6532|11255|225689	ADORA2A|ADRA2A|ADRA2B|ADRA2C|AGTR1|CFTR|CHRM5|CNR1|CYP19A1|AKR1C1|DRD1|DRD2|DRD3|DRD4|HTR1A|HTR1B|HTR2A|MAOB|NFKBIA|NOS1|OPRK1|PON1|PPARG|SLC5A1|SLC6A2|SLC6A3|SLC6A4|HRH3|MAPK15	-23.8421	0	10	0	0	-27.7221	91.80935033
1	-22.975	GO:0043279	M1	1	0	GO Biological Processes	19	response to alkaloid	-22.975	28.74032	23.25513	30242	115	183	20	10.92896	2.306387	100|135|836|890|1268|1723|1728|1813|1814|1815|3066|3351|3356|3383|4986|4988|5582|5970|6531|7442	ADA|ADORA2A|CASP3|CCNA2|CNR1|DHODH|NQO1|DRD2|DRD3|DRD4|HDAC2|HTR1B|HTR2A|ICAM1|OPRK1|OPRM1|PRKCG|RELA|SLC6A3|TRPV1	-20.6483	0	10	0	0	-27.7221	91.80935033

<table>
<tr><td>1</td><td>-21.9188</td><td>GO:0051937</td><td>M1</td><td>1</td><td>0</td><td>GO Biological Processes</td><td>19</td><td>catecholamine transport</td><td>-21.9188</td><td>38.48447</td><td>25.01893</td><td>30242</td><td>73</td><td>183</td><td>17</td><td>9.289617</td><td>2.145862</td><td>135|150|151|152|1133|1268|1812|1813|1814|1815|3351|3356|4986|6530|6531|11255|225689</td><td>ADORA2A|ADRA2A|ADRA2B|ADRA2C|CHRM5|CNR1|DRD1|DRD2|DRD3|DRD4|HTR1B|HTR2A|OPRK1|SLC6A2|SLC6A3|HRH3|MAPK15</td><td>-19.6313</td><td>0</td><td>10</td><td>0</td><td>0</td><td>-27.7221</td><td>91.80935033</td></tr>
<tr><td>1</td><td>-16.166</td><td>GO:0001505</td><td>M1</td><td>1</td><td>0</td><td>GO Biological Processes</td><td>19</td><td>regulation of neurotransmitter levels</td><td>-16.166</td><td>14.67234</td><td>15.66112</td><td>30242</td><td>214</td><td>183</td><td>19</td><td>10.38251</td><td>2.254873</td><td>135|148|150|1268|1812|1813|1814|1815|3351|3356|4128|4129|4842|5582|6530|6531|6532|11255|23621</td><td>ADORA2A|ADRA1A|ADRA2A|CNR1|DRD1|DRD2|DRD3|DRD4|HTR1B|HTR2A|MAOA|MAOB|NOS1|PRKCG|SLC6A2|SLC6A3|SLC6A4|HRH3|BACE1</td><td>-14.1099</td><td>0</td><td>10</td><td>0</td><td>0</td><td>-27.7221</td><td>91.80935033</td></tr>
<tr><td>1</td><td>-15.7792</td><td>GO:0015872</td><td>M1</td><td>1</td><td>0</td><td>GO Biological Processes</td><td>19</td><td>dopamine transport</td><td>-15.7792</td><td>39.66164</td><td>21.34793</td><td>30242</td><td>50</td><td>183</td><td>12</td><td>6.557377</td><td>1.829835</td><td>1133|1268|1812|1813|1814|1815|3351|3356|4986|6530|6531|225689</td><td>CHRM5|CNR1|DRD1|DRD2|DRD3|DRD4|HTR1B|HTR2A|OPRK1|SLC6A2|SLC6A3|MAPK15</td><td>-13.7593</td><td>0</td><td>10</td><td>0</td><td>0</td><td>-27.7221</td><td>91.80935033</td></tr>
<tr><td>1</td><td>-15.4208</td><td>GO:0051952</td><td>M1</td><td>1</td><td>0</td><td>GO Biological Processes</td><td>19</td><td>regulation of amine transport</td><td>-15.4208</td><td>25.42413</td><td>18.20645</td><td>30242</td><td>91</td><td>183</td><td>14</td><td>7.650273</td><td>1.964856</td><td>134|135|150|151|152|1268|1812|1813|1814|1815|3351|3356|4986|11255</td><td>ADORA1|ADORA2A|ADRA2A|ADRA2B|ADRA2C|CNR1|DRD1|DRD2|DRD3|DRD4|HTR1B|HTR2A|OPRK1|HRH3</td><td>-13.4095</td><td>0</td><td>10</td><td>0</td><td>0</td><td>-27.7221</td><td>91.80935033</td></tr>
<tr><td>1</td><td>-14.8878</td><td>GO:0015837</td><td>M1</td><td>1</td><td>0</td><td>GO Biological Processes</td><td>19</td><td>amine transport</td><td>-14.8878</td><td>23.36965</td><td>17.39482</td><td>30242</td><td>99</td><td>183</td><td>14</td><td>7.650273</td><td>1.964856</td><td>134|135|150|151|152|1268|1812|1813|1814|1815|3351|3356|4986|11255</td><td>ADORA1|ADORA2A|ADRA2A|ADRA2B|ADRA2C|CNR1|DRD1|DRD2|DRD3|DRD4|HTR1B|HTR2A|OPRK1|HRH3</td><td>-12.904</td><td>0</td><td>10</td><td>0</td><td>0</td><td>-27.7221</td><td>91.80935033</td></tr>
<tr><td>1</td><td>-13.8729</td><td>GO:0006836</td><td>M1</td><td>1</td><td>0</td><td>GO Biological Processes</td><td>19</td><td>neurotransmitter transport</td><td>-13.8729</td><td>13.44194</td><td>14.0831</td><td>30242</td><td>209</td><td>183</td><td>17</td><td>9.289617</td><td>2.145862</td><td>135|148|150|1268|1812|1813|1814|1815|3351|3356|4842|5582|6530|6531|6532|11255|23621</td><td>ADORA2A|ADRA1A|ADRA2A|CNR1|DRD1|DRD2|DRD3|DRD4|HTR1B|HTR2A|NOS1|PRKCG|SLC6A2|SLC6A3|SLC6A4|HRH3|BACE1</td><td>-11.944</td><td>0</td><td>10</td><td>0</td><td>0</td><td>-27.7221</td><td>91.80935033</td></tr>
<tr><td>1</td><td>-13.554</td><td>GO:0042220</td><td>M1</td><td>1</td><td>0</td><td>GO Biological Processes</td><td>19</td><td>response to cocaine</td><td>-13.554</td><td>33.05137</td><td>18.56326</td><td>30242</td><td>55</td><td>183</td><td>11</td><td>6.010929</td><td>1.757049</td><td>890|1268|1813|1814|1815|3066|3351|3356|4986|4988|6531</td><td>CCNA2|CNR1|DRD2|DRD3|DRD4|HDAC2|HTR1B|HTR2A|OPRK1|OPRM1|SLC6A3</td><td>-11.6345</td><td>0</td><td>10</td><td>0</td><td>0</td><td>-27.7221</td><td>91.80935033</td></tr>
<tr><td>1</td><td>-13.4613</td><td>GO:0050433</td><td>M1</td><td>1</td><td>0</td><td>GO Biological Processes</td><td>19</td><td>regulation of catecholamine secretion</td><td>-13.4613</td><td>32.46116</td><td>18.38664</td><td>30242</td><td>56</td><td>183</td><td>11</td><td>6.010929</td><td>1.757049</td><td>135|150|151|152|1268|1813|1814|3351|3356|4986|11255</td><td>ADORA2A|ADRA2A|ADRA2B|ADRA2C|CNR1|DRD2|DRD3|HTR1B|HTR2A|OPRK1|HRH3</td><td>-11.5509</td><td>0</td><td>10</td><td>0</td><td>0</td><td>-27.7221</td><td>91.80935033</td></tr>
<tr><td>1</td><td>-13.3704</td><td>GO:0051588</td><td>M1</td><td>1</td><td>0</td><td>GO Biological Processes</td><td>19</td><td>regulation of neurotransmitter transport</td><td>-13.3704</td><td>21.48339</td><td>16.00855</td><td>30242</td><td>100</td><td>183</td><td>13</td><td>7.103825</td><td>1.898976</td><td>135|148|150|1268|1812|1813|1814|1815|3351|3356|4842|5582|23621</td><td>ADORA2A|ADRA1A|ADRA2A|CNR1|DRD1|DRD2|DRD3|DRD4|HTR1B|HTR2A|NOS1|</td><td>-11.4644</td><td>0</td><td>10</td><td>0</td><td>0</td><td>-27.7221</td><td>91.80935033</td></tr>
</table>

																			PRKCG|BACE1							
1	-13.2814	GO:0050432	M1	1	0	GO Biological Processes	19	catecholamine secretion	-13.2814	31.34181	18.04694	30242	58	183	11	6.010929	1.757049	135|150|151|152|1268|1813|1814|3351|3356|4986|11255	ADORA2A|ADRA2A|ADRA2B|ADRA2C|CNR1|DRD2|DRD3|HTR1B|HTR2A|OPRK1|HRH3	-11.3799	0	10	0	0	-27.7221	91.80935033
1	-10.8272	GO:0051932	M1	1	0	GO Biological Processes	19	synaptic transmission, GABAergic	-10.8272	30.3533	16.04494	30242	49	183	9	4.918033	1.598524	134|135|148|1268|1813|2554|2558|2566|3351	ADORA1|ADORA2A|ADRA1A|CNR1|DRD2|GABRA1|GABRA5|GABRG2|HTR1B	-9.08757	0	10	0	0	-27.7221	91.80935033
1	-10.6114	GO:0014072	M1	1	0	GO Biological Processes	19	response to isoquinoline alkaloid	-10.6114	38.88396	17.24528	30242	34	183	8	4.371585	1.511428	100|1268|1813|1814|4986|4988|5582|5970	ADA|CNR1|DRD2|DRD3|OPRK1|OPRM1|PRKCG|RELA	-8.88381	0	10	0	0	-27.7221	91.80935033
1	-10.6114	GO:0043278	M1	1	0	GO Biological Processes	19	response to morphine	-10.6114	38.88396	17.24528	30242	34	183	8	4.371585	1.511428	100|1268|1813|1814|4986|4988|5582|5970	ADA|CNR1|DRD2|DRD3|OPRK1|OPRM1|PRKCG|RELA	-8.88381	0	10	0	0	-27.7221	91.80935033
1	-10.4254	GO:2000300	M1	1	0	GO Biological Processes	19	regulation of synaptic vesicle exocytosis	-10.4254	27.54281	15.23221	30242	54	183	9	4.918033	1.598524	135|148|150|1268|1813|3351|3356|5582|23621	ADORA2A|ADRA1A|ADRA2A|CNR1|DRD2|HTR1B|HTR2A|PRKCG|BACE1	-8.71086	0	10	0	0	-27.7221	91.80935033
1	-10.0327	GO:0060627	M1	1	0	GO Biological Processes	19	regulation of vesicle-mediated transport	-10.0327	6.236107	9.488921	30242	530	183	20	10.92896	2.306387	135|148|150|351|1080|1268|1813|1814|1815|3351|3356|3643|5291|5582|6347|6401|7124|23621|225689|729230	ADORA2A|ADRA1A|ADRA2A|APP|CFTR|CNR1|DRD2|DRD3|DRD4|HTR1B|HTR2A|INSR|PIK3CB|PRKCG|CCL2|SELE|TNF|BACE1|MAPK15|CCR2	-8.34183	0	10	0	0	-27.7221	91.80935033
1	-9.02275	GO:0051967	M1	1	0	GO Biological Processes	19	negative regulation of synaptic transmission, glutamatergic	-9.02275	91.80935	21.25923	30242	9	183	5	2.73224	1.205087	134|1813|1814|3351|3356	ADORA1|DRD2|DRD3|HTR1B|HTR2A	-7.4077	0	10	0	0	-27.7221	91.80935033
1	-8.56059	GO:0046928	M1	1	0	GO Biological Processes	19	regulation of neurotransmitter secretion	-8.56059	17.29432	11.80687	30242	86	183	9	4.918033	1.598524	135|148|150|1268|1813|3351|3356|5582|23621	ADORA2A|ADRA1A|ADRA2A|CNR1|DRD2|HTR1B|HTR2A|PRKCG|BACE1	-6.97527	0	10	0	0	-27.7221	91.80935033
1	-7.94633	GO:1903305	M1	1	0	GO Biological Processes	19	regulation of regulated secretory pathway	-7.94633	12.15124	10.16956	30242	136	183	10	5.464481	1.680144	135|148|150|1268|1813|3351|3356|5582|23621|729230	ADORA2A|ADRA1A|ADRA2A|CNR1|DRD2|HTR1B|HTR2A|PRKCG|BACE1|CCR2	-6.41499	0	10	0	0	-27.7221	91.80935033
1	-7.83233	GO:0048148	M1	1	0	GO Biological Processes	19	behavioral response to cocaine	-7.83233	59.0203	16.94243	30242	14	183	5	2.73224	1.205087	1813|1814|1815|3356|4986	DRD2|DRD3|DRD4|HTR2A|OPRK1	-6.31304	0	10	0	0	-27.7221	91.80935033
1	-7.75184	GO:0016079	M1	1	0	GO Biological Processes	19	synaptic vesicle exocytosis	-7.75184	14.03124	10.48654	30242	106	183	9	4.918033	1.598524	135|148|150|1268|1813|3351|3356|5582|23621	ADORA2A|ADRA1A|ADRA2A|CNR1|DRD2|HTR1B|HTR2A|PRKCG|BACE1	-6.23981	0	10	0	0	-27.7221	91.80935033
1	-7.65048	GO:0007269	M1	1	0	GO Biological Processes	19	neurotransmitter secretion	-7.65048	11.31896	9.752015	30242	146	183	10	5.464481	1.680144	135|148|150|1268|1813|3351|3356|5582|11255|23621	ADORA2A|ADRA1A|ADRA2A|CNR1|DRD2|HTR1B|HTR2A|PRKCG|HRH3|BACE1	-6.14998	0	10	0	0	-27.7221	91.80935033
1	-7.65048	GO:0099643	M1	1	0	GO Biological Processes	19	signal release from synapse	-7.65048	11.31896	9.752015	30242	146	183	10	5.464481	1.680144	135|148|150|1268|1813|3351|3356|5582|11255|23621	ADORA2A|ADRA1A|ADRA2A|CNR1|DRD2|HTR1B|HTR2A|PRK	-6.14998	0	10	0	0	-27.7221	91.80935033

																			CG|HRH3|BACE1							
1	-7.35926	GO:0032228	M1	1	0	GO Biological Processes	19	regulation of synaptic transmission, GABAergic	-7.35926	30.0467	13.02632	30242	33	183	6	3.278689	1.316393	134|135|148|1268|1813|3351	ADORA1|ADORA2A|ADRA1A|CNR1|DRD2|HTR1B	-5.88178	0	10	0	0	-27.7221	91.80935033
1	-7.35017	GO:0099003	M1	1	0	GO Biological Processes	19	vesicle-mediated transport in synapse	-7.35017	9.043906	8.927795	30242	201	183	11	6.010929	1.757049	135|148|150|1268|1813|1814|1815|3351|3356|5582|23621	ADORA2A|ADRA1A|ADRA2A|CNR1|DRD2|DRD3|DRD4|HTR1B|HTR2A|PRKCG|BACE1	-5.87776	0	10	0	0	-27.7221	91.80935033
1	-7.28439	GO:0017157	M1	1	0	GO Biological Processes	19	regulation of exocytosis	-7.28439	8.910908	8.845904	30242	204	183	11	6.010929	1.757049	135|148|150|1080|1268|1813|3351|3356|5582|23621|729230	ADORA2A|ADRA1A|ADRA2A|CFTR|CNR1|DRD2|HTR1B|HTR2A|PRKCG|BACE1|CCR2	-5.8224	0	10	0	0	-27.7221	91.80935033
1	-7.12062	GO:0014046	M1	1	0	GO Biological Processes	19	dopamine secretion	-7.12062	27.54281	12.43334	30242	36	183	6	3.278689	1.316393	1268|1813|1814|3351|3356|4986	CNR1|DRD2|DRD3|HTR1B|HTR2A|OPRK1	-5.67283	0	10	0	0	-27.7221	91.80935033
1	-7.12062	GO:0014059	M1	1	0	GO Biological Processes	19	regulation of dopamine secretion	-7.12062	27.54281	12.43334	30242	36	183	6	3.278689	1.316393	1268|1813|1814|3351|3356|4986	CNR1|DRD2|DRD3|HTR1B|HTR2A|OPRK1	-5.67283	0	10	0	0	-27.7221	91.80935033
1	-6.73764	GO:0045055	M1	1	0	GO Biological Processes	19	regulated exocytosis	-6.73764	7.869373	8.177475	30242	231	183	11	6.010929	1.757049	135|148|150|1268|1813|3351|3356|5293|5582|23621|729230	ADORA2A|ADRA1A|ADRA2A|CNR1|DRD2|HTR1B|HTR2A|PIK3CD|PRKCG|BACE1|CCR2	-5.32627	0	10	0	0	-27.7221	91.80935033
1	-6.68154	GO:0050805	M1	1	0	GO Biological Processes	19	negative regulation of synaptic transmission	-6.68154	16.76519	10.22941	30242	69	183	7	3.825137	1.417845	134|1813|1814|3351|3356|5743|6532	ADORA1|DRD2|DRD3|HTR1B|HTR2A|PTGS2|SLC6A4	-5.27433	0	10	0	0	-27.7221	91.80935033
1	-6.54017	GO:0006887	M1	1	0	GO Biological Processes	19	exocytosis	-6.54017	6.051659	7.470391	30242	355	183	13	7.103825	1.898976	135|148|150|1080|1230|1268|1813|3351|3356|5293|5582|23621|729230	ADORA2A|ADRA1A|ADRA2A|CFTR|CCR1|CNR1|DRD2|HTR1B|HTR2A|PIK3CD|PRKCG|BACE1|CCR2	-5.14832	0	10	0	0	-27.7221	91.80935033
1	-5.74543	GO:0099504	M1	1	0	GO Biological Processes	19	synaptic vesicle cycle	-5.74543	8.172041	7.57217	30242	182	183	9	4.918033	1.598524	135|148|150|1268|1813|3351|3356|5582|23621	ADORA2A|ADRA1A|ADRA2A|CNR1|DRD2|HTR1B|HTR2A|PRKCG|BACE1	-4.4216	0	10	0	0	-27.7221	91.80935033
1	-27.0145	GO:0048511	M1	1	0	GO Biological Processes	19	rhythmic process	-27.0145	16.58095	21.12605	30242	299	183	30	16.39344	2.736716	100|134|135|153|836|1453|1813|1814|1815|1956|2099|2155|3062|3066|3363|4843|4914|4915|4986|4988|5241|5465|5468|5582|5591|6532|7157|7298|9475|10135	ADA|ADORA1|ADORA2A|ADRB1|CASP3|CSNK1D|DRD2|DRD3|DRD4|EGFR|ESR1|F7|HCRTR2|HDAC2|HTR7|NOS2|NTRK1|NTRK2|OPRK1|OPRM1|PGR|PPARA|PPARG|PRKCG|PRKDC|SLC6A4|TP53|TYMS|ROCK2|NAMPT	-24.5088	0	11	1	1	-27.0145	20.48638396
1	-24.3828	GO:0007623	M1	1	0	GO Biological Processes	19	circadian rhythm	-24.3828	19.67343	21.18742	30242	210	183	25	13.6612	2.538762	100|134|135|153|1453|1813|1814|1815|1956|2155|3062|3066|3363|4843|4914|4915|5465|5468|5582|5591|6532|7157|7	ADA|ADORA1|ADORA2A|ADRB1|CSNK1D|DRD2|DRD3|DRD4|EGFR|F7|HCRTR2|HDAC2|HTR7|NOS2|NTRK	-21.9998	0	11	0	0	-27.0145	20.48638396

<table>
<tr><td></td><td></td><td></td><td></td><td></td><td></td><td></td><td></td><td></td><td></td><td></td><td></td><td></td><td></td><td></td><td></td><td></td><td></td><td>298|9475|10135</td><td>1|NTRK2|PPARA|PPARG|PRKCG|PRKDC|SLC6A4|TP53|TYMS|ROCK2|NAMPT</td><td></td><td></td><td></td><td></td><td></td><td></td><td></td></tr>
<tr><td>1</td><td>-15.0352</td><td>GO:0042752</td><td>M1</td><td>1</td><td>0</td><td>GO Biological Processes</td><td>19</td><td>regulation of circadian rhythm</td><td>-15.0352</td><td>20.48638</td><td>16.75813</td><td>30242</td><td>121</td><td>183</td><td>15</td><td>8.196721</td><td>2.027793</td><td>100|134|135|153|1453|1813|1814|1815|3062|5465|5468|5582|5591|7157|9475</td><td>ADA|ADORA1|ADORA2A|ADRB1|CSNK1D|DRD2|DRD3|DRD4|HCRTR2|PPARA|PPARG|PRKCG|PRKDC|TP53|ROCK2</td><td>-13.0433</td><td>0</td><td>11</td><td>0</td><td>0</td><td>-27.0145</td><td>20.48638396</td></tr>
<tr><td>1</td><td>-26.3052</td><td>GO:0051046</td><td>M1</td><td>1</td><td>0</td><td>GO Biological Processes</td><td>19</td><td>regulation of secretion</td><td>-26.3052</td><td>10.07987</td><td>17.86796</td><td>30242</td><td>623</td><td>183</td><td>38</td><td>20.76503</td><td>2.998466</td><td>100|134|135|148|150|151|152|185|1080|1268|1588|1813|1814|1815|1910|1956|2149|3156|3350|3351|3356|3358|3569|4129|4843|4986|4988|5467|5582|6532|7040|7099|7124|7132|7442|11255|23621|729230</td><td>ADA|ADORA1|ADORA2A|ADRA1A|ADRA2A|ADRA2B|ADRA2C|AGTR1|CFTR|CNR1|CYP19A1|DRD2|DRD3|DRD4|EDNRB|EGFR|F2R|HMGCR|HTR1A|HTR1B|HTR2A|HTR2C|IL6|MAOB|NOS2|OPRK1|OPRM1|PPARD|PRKCG|SLC6A4|TGFB1|TLR4|TNF|TNFRSF1A|TRPV1|HRH3|BACE1|CCR2</td><td>-23.8252</td><td>0</td><td>12</td><td>1</td><td>1</td><td>-26.3052</td><td>19.11133415</td></tr>
<tr><td>1</td><td>-24.5815</td><td>GO:0032940</td><td>M1</td><td>1</td><td>0</td><td>GO Biological Processes</td><td>19</td><td>secretion by cell</td><td>-24.5815</td><td>8.314809</td><td>16.30806</td><td>30242</td><td>795</td><td>183</td><td>40</td><td>21.85792</td><td>3.055072</td><td>134|135|148|150|151|152|185|1080|1230|1268|1588|1813|1814|1815|1956|2149|3156|3350|3351|3356|3358|3569|4129|4843|4915|4986|4988|5293|5467|5582|6532|7040|7099|7124|7132|7442|11255|23621|59341|729230</td><td>ADORA1|ADORA2A|ADRA1A|ADRA2A|ADRA2B|ADRA2C|AGTR1|CFTR|CCR1|CNR1|CYP19A1|DRD2|DRD3|DRD4|EGFR|F2R|HMGCR|HTR1A|HTR1B|HTR2A|HTR2C|IL6|MAOB|NOS2|NTRK2|OPRK1|OPRM1|PIK3CD|PPARD|PRKCG|SLC6A4|TGFB1|TLR4|TNF|TNFRSF1A|TRPV1|HRH3|BACE1|TRPV4|CCR2</td><td>-22.1779</td><td>0</td><td>12</td><td>0</td><td>0</td><td>-26.3052</td><td>19.11133415</td></tr>
<tr><td>1</td><td>-24.4041</td><td>GO:1903530</td><td>M1</td><td>1</td><td>0</td><td>GO Biological Processes</td><td>19</td><td>regulation of secretion by cell</td><td>-24.4041</td><td>10.23715</td><td>17.29372</td><td>30242</td><td>565</td><td>183</td><td>35</td><td>19.12568</td><td>2.907289</td><td>134|135|148|150|151|152|185|1080|1268|1588|1813|1814|1815|1956|2149|3156|3350|3351|3356|3358|3569|4129|4843|4986|4988|5467|5582|6532|7040|7099|7124|7132|11255|23621|729230</td><td>ADORA1|ADORA2A|ADRA1A|ADRA2A|ADRA2B|ADRA2C|AGTR1|CFTR|CNR1|CYP19A1|DRD2|DRD3|DRD4|EGFR|F2R|HMGCR|HTR1A|HTR1B|HTR2A|HTR2C|IL6|MAOB|NOS2|OPRK1|OPRM1|PPARD|PRKCG|SLC6A4|TGFB1|TLR4|TNF|TNFRSF1A|HRH</td><td>-22.0143</td><td>0</td><td>12</td><td>0</td><td>0</td><td>-26.3052</td><td>19.11133415</td></tr>
</table>

																			3|BACE1|CCR2							
1	-22.5213	GO:0023061	M1	1	0	GO Biological Processes	19	signal release	-22.5213	11.01712	16.98499	30242	465	183	31	16.93989	2.772848	134|135|148|150|151|152|185|1080|1268|1588|1813|1814|1956|2149|3156|3350|3351|3356|3358|3569|4129|4843|4986|4988|5467|5582|6532|7124|11255|23621|59341	ADORA1|ADORA2A|ADRA1A|ADRA2A|ADRA2B|ADRA2C|AGTR1|CFTR|CNR1|CYP19A1|DRD2|DRD3|EGFR|F2R|HMGCR|HTR1A|HTR1B|HTR2A|HTR2C|IL6|MAOB|NOS2|OPRK1|OPRM1|PPARD|PRKCG|SLC6A4|TNF|HRH3|BACE1|TRPV4	-20.2118	0	12	0	0	-26.3052	19.11133415
1	-17.8781	GO:0051051	M1	1	0	GO Biological Processes	19	negative regulation of transport	-17.8781	9.433265	14.42383	30242	473	183	27	14.7541	2.621607	100|134|150|151|152|351|596|1268|1813|1814|1815|2149|3156|3351|3356|3383|3757|4129|4318|4842|4986|4988|5743|7124|7132|11255|729230	ADA|ADORA1|ADRA2A|ADRA2B|ADRA2C|APP|BCL2|CNR1|DRD2|DRD3|DRD4|F2R|HMGCR|HTR1B|HTR2A|ICAM1|KCNH2|MAOB|MMP9|NOS1|OPRK1|OPRM1|PTGS2|TNF|TNFRSF1A|HRH3|CCR2	-15.739	0	12	0	0	-26.3052	19.11133415
1	-16.6599	GO:0051048	M1	1	0	GO Biological Processes	19	negative regulation of secretion	-16.6599	17.39546	16.77583	30242	171	183	18	9.836066	2.201413	100|134|150|151|152|1268|1813|1814|1815|2149|3156|3351|4129|4986|4988|7132|11255|729230	ADA|ADORA1|ADRA2A|ADRA2B|ADRA2C|CNR1|DRD2|DRD3|DRD4|F2R|HMGCR|HTR1B|MAOB|OPRK1|OPRM1|TNFRSF1A|HRH3|CCR2	-14.5608	0	12	0	0	-26.3052	19.11133415
1	-16.458	GO:1903531	M1	1	0	GO Biological Processes	19	negative regulation of secretion by cell	-16.458	19.11133	17.17506	30242	147	183	17	9.289617	2.145862	134|150|151|152|1268|1813|1814|1815|2149|3156|3351|4129|4986|4988|7132|11255|729230	ADORA1|ADRA2A|ADRA2B|ADRA2C|CNR1|DRD2|DRD3|DRD4|F2R|HMGCR|HTR1B|MAOB|OPRK1|OPRM1|TNFRSF1A|HRH3|CCR2	-14.3759	0	12	0	0	-26.3052	19.11133415
1	-14.1166	GO:0010817	M1	1	0	GO Biological Processes	19	regulation of hormone levels	-14.1166	7.838269	12.10365	30242	506	183	24	13.11475	2.495327	134|150|185|1080|1268|1543|1544|1545|1588|1636|1645|1813|1956|2099|3156|3350|3358|3569|4843|4986|5467|7124|7364|59341	ADORA1|ADRA2A|AGTR1|CFTR|CNR1|CYP1A1|CYP1A2|CYP1B1|CYP19A1|ACE|AKR1C1|DRD2|EGFR|ESR1|HMGCR|HTR1A|HTR2C|IL6|NOS2|OPRK1|PPARD|TNF|UGT2B7|TRPV4	-12.1636	0	12	0	0	-26.3052	19.11133415
1	-12.283	GO:0042886	M1	1	0	GO Biological Processes	19	amide transport	-12.283	9.784944	12.0116	30242	304	183	18	9.836066	2.201413	134|150|760|1080|1268|1813|1956|3156|3358|3569|4363|4843|5243|5467|7124|7442|9429|59341	ADORA1|ADRA2A|CA2|CFTR|CNR1|DRD2|EGFR|HMGCR|HTR2C|IL6|ABCC1|NOS2|ABCB1|PPARD|TNF|TR	-10.443	0	12	0	0	-26.3052	19.11133415

<table>
<tr><td></td><td></td><td></td><td></td><td></td><td></td><td></td><td></td><td></td><td></td><td></td><td></td><td></td><td></td><td></td><td></td><td></td><td></td><td></td><td>PV1|ABCG2|TRPV4</td><td></td><td></td><td></td><td></td><td></td><td></td><td></td></tr>
<tr><td>1</td><td>-12.1054</td><td>GO:0050708</td><td>M1</td><td>1</td><td>0</td><td>GO Biological Processes</td><td>19</td><td>regulation of protein secretion</td><td>-12.1054</td><td>10.48271</td><td>12.16645</td><td>30242</td><td>268</td><td>183</td><td>17</td><td>9.289617</td><td>2.145862</td><td>135|150|1080|1268|1813|1814|1815|1956|2149|3156|3569|4843|4988|5467|7040|7099|7124</td><td>ADORA2A|ADRA2A|CFTR|CNR1|DRD2|DRD3|DRD4|EGFR|F2R|HMGCR|IL6|NOS2|OPRM1|PPARD|TGFB1|TLR4|TNF</td><td>-10.2749</td><td>0</td><td>12</td><td>0</td><td>0</td><td>-26.3052</td><td>19.11133415</td></tr>
<tr><td>1</td><td>-11.4265</td><td>GO:0046883</td><td>M1</td><td>1</td><td>0</td><td>GO Biological Processes</td><td>19</td><td>regulation of hormone secretion</td><td>-11.4265</td><td>10.4925</td><td>11.80671</td><td>30242</td><td>252</td><td>183</td><td>16</td><td>8.743169</td><td>2.088053</td><td>134|150|185|1080|1268|1588|1813|1956|3156|3350|3358|3569|4843|4986|5467|7124</td><td>ADORA1|ADRA2A|AGTR1|CFTR|CNR1|CYP19A1|DRD2|EGFR|HMGCR|HTR1A|HTR2C|IL6|NOS2|OPRK1|PPARD|TNF</td><td>-9.63538</td><td>0</td><td>12</td><td>0</td><td>0</td><td>-26.3052</td><td>19.11133415</td></tr>
<tr><td>1</td><td>-11.367</td><td>GO:0046879</td><td>M1</td><td>1</td><td>0</td><td>GO Biological Processes</td><td>19</td><td>hormone secretion</td><td>-11.367</td><td>9.427403</td><td>11.40731</td><td>30242</td><td>298</td><td>183</td><td>17</td><td>9.289617</td><td>2.145862</td><td>134|150|185|1080|1268|1588|1813|1956|3156|3350|3358|3569|4843|4986|5467|7124|59341</td><td>ADORA1|ADRA2A|AGTR1|CFTR|CNR1|CYP19A1|DRD2|EGFR|HMGCR|HTR1A|HTR2C|IL6|NOS2|OPRK1|PPARD|TNF|TRPV4</td><td>-9.58091</td><td>0</td><td>12</td><td>0</td><td>0</td><td>-26.3052</td><td>19.11133415</td></tr>
<tr><td>1</td><td>-11.1207</td><td>GO:0015833</td><td>M1</td><td>1</td><td>0</td><td>GO Biological Processes</td><td>19</td><td>peptide transport</td><td>-11.1207</td><td>10.01557</td><td>11.47968</td><td>30242</td><td>264</td><td>183</td><td>16</td><td>8.743169</td><td>2.088053</td><td>134|150|760|1080|1268|1813|1956|3156|3358|3569|4363|4843|5467|7124|7442|59341</td><td>ADORA1|ADRA2A|CA2|CFTR|CNR1|DRD2|EGFR|HMGCR|HTR2C|IL6|ABCC1|NOS2|PPARD|TNF|TRPV1|TRPV4</td><td>-9.35288</td><td>0</td><td>12</td><td>0</td><td>0</td><td>-26.3052</td><td>19.11133415</td></tr>
<tr><td>1</td><td>-11.117</td><td>GO:0009914</td><td>M1</td><td>1</td><td>0</td><td>GO Biological Processes</td><td>19</td><td>hormone transport</td><td>-11.117</td><td>9.0918</td><td>11.15541</td><td>30242</td><td>309</td><td>183</td><td>17</td><td>9.289617</td><td>2.145862</td><td>134|150|185|1080|1268|1588|1813|1956|3156|3350|3358|3569|4843|4986|5467|7124|59341</td><td>ADORA1|ADRA2A|AGTR1|CFTR|CNR1|CYP19A1|DRD2|EGFR|HMGCR|HTR1A|HTR2C|IL6|NOS2|OPRK1|PPARD|TNF|TRPV4</td><td>-9.35073</td><td>0</td><td>12</td><td>0</td><td>0</td><td>-26.3052</td><td>19.11133415</td></tr>
<tr><td>1</td><td>-10.0955</td><td>GO:0009306</td><td>M1</td><td>1</td><td>0</td><td>GO Biological Processes</td><td>19</td><td>protein secretion</td><td>-10.0955</td><td>7.825532</td><td>10.15098</td><td>30242</td><td>359</td><td>183</td><td>17</td><td>9.289617</td><td>2.145862</td><td>135|150|1080|1268|1813|1814|1815|1956|2149|3156|3569|4843|4988|5467|7040|7099|7124</td><td>ADORA2A|ADRA2A|CFTR|CNR1|DRD2|DRD3|DRD4|EGFR|F2R|HMGCR|IL6|NOS2|OPRM1|PPARD|TGFB1|TLR4|TNF</td><td>-8.39923</td><td>0</td><td>12</td><td>0</td><td>0</td><td>-26.3052</td><td>19.11133415</td></tr>
<tr><td>1</td><td>-10.0768</td><td>GO:0035592</td><td>M1</td><td>1</td><td>0</td><td>GO Biological Processes</td><td>19</td><td>establishment of protein localization to extracellular region</td><td>-10.0768</td><td>7.803795</td><td>10.13291</td><td>30242</td><td>360</td><td>183</td><td>17</td><td>9.289617</td><td>2.145862</td><td>135|150|1080|1268|1813|1814|1815|1956|2149|3156|3569|4843|4988|5467|7040|7099|7124</td><td>ADORA2A|ADRA2A|CFTR|CNR1|DRD2|DRD3|DRD4|EGFR|F2R|HMGCR|IL6|NOS2|OPRM1|PPARD|TGFB1|TLR4|TNF</td><td>-8.38354</td><td>0</td><td>12</td><td>0</td><td>0</td><td>-26.3052</td><td>19.11133415</td></tr>
<tr><td>1</td><td>-9.92899</td><td>GO:0071692</td><td>M1</td><td>1</td><td>0</td><td>GO Biological Processes</td><td>19</td><td>protein localization to extracellular region</td><td>-9.92899</td><td>7.634147</td><td>9.990763</td><td>30242</td><td>368</td><td>183</td><td>17</td><td>9.289617</td><td>2.145862</td><td>135|150|1080|1268|1813|1814|1815|1956|2149|3156|3569|4843|4988|5467|7040|7099|7124</td><td>ADORA2A|ADRA2A|CFTR|CNR1|DRD2|DRD3|DRD4|EGFR|F2R|HMGCR|IL6|NOS2|OPRM1|PPARD|TGFB1|TLR4|TNF</td><td>-8.25153</td><td>0</td><td>12</td><td>0</td><td>0</td><td>-26.3052</td><td>19.11133415</td></tr>
<tr><td>1</td><td>-9.52625</td><td>GO:0002790</td><td>M1</td><td>1</td><td>0</td><td>GO Biological Processes</td><td>19</td><td>peptide secretion</td><td>-9.52625</td><td>9.560313</td><td>10.43213</td><td>30242</td><td>242</td><td>183</td><td>14</td><td>7.650273</td><td>1.964856</td><td>134|150|1080|1268|1813|1956|3156|3358|3569|</td><td>ADORA1|ADRA2A|CFTR|CNR1|DRD2|EGF</td><td>-7.88049</td><td>0</td><td>12</td><td>0</td><td>0</td><td>-26.3052</td><td>19.11133415</td></tr>
</table>

																		4843|5467|7124|7442|59341	R|HMGCR|HTR2C|IL6|NOS2|PPARD|TNF|TRPV1|TRPV4							
1	-9.45027	GO:0090087	M1	1	0	GO Biological Processes	19	regulation of peptide transport	-9.45027	10.63534	10.72085	30242	202	183	13	7.103825	1.898976	134|150|760|1080|1268|1813|1956|3156|3358|3569|4843|5467|7124	ADORA1|ADRA2A|CA2|CFTR|CNR1|DRD2|EGFR|HMGCR|HTR2C|IL6|NOS2|PPARD|TNF	-7.80817	0	12	0	0	-26.3052	19.11133415
1	-9.40169	GO:0051223	M1	1	0	GO Biological Processes	19	regulation of protein transport	-9.40169	6.108716	9.114793	30242	514	183	19	10.38251	2.254873	135|150|1080|1128|1268|1813|1814|1815|1956|2149|3156|3569|4843|4988|5467|5743|7040|7099|7124	ADORA2A|ADRA2A|CFTR|CHRM1|CNR1|DRD2|DRD3|DRD4|EGFR|F2R|HMGCR|IL6|NOS2|OPRM1|PPARD|PTGS2|TGFB1|TLR4|TNF	-7.76201	0	12	0	0	-26.3052	19.11133415
1	-9.09267	GO:0051047	M1	1	0	GO Biological Processes	19	positive regulation of secretion	-9.09267	7.996298	9.660901	30242	310	183	15	8.196721	2.027793	134|135|1080|1268|1588|1813|1814|1910|1956|4986|5467|6532|7040|7099|7442	ADORA1|ADORA2A|CFTR|CNR1|CYP19A1|DRD2|DRD3|EDNRB|EGFR|OPRK1|PPARD|SLC6A4|TGFB1|TLR4|TRPV1	-7.46955	0	12	0	0	-26.3052	19.11133415
1	-9.05954	GO:0070201	M1	1	0	GO Biological Processes	19	regulation of establishment of protein localization	-9.05954	5.82538	8.819868	30242	539	183	19	10.38251	2.254873	135|150|1080|1128|1268|1813|1814|1815|1956|2149|3156|3569|4843|4988|5467|5743|7040|7099|7124	ADORA2A|ADRA2A|CFTR|CHRM1|CNR1|DRD2|DRD3|DRD4|EGFR|F2R|HMGCR|IL6|NOS2|OPRM1|PPARD|PTGS2|TGFB1|TLR4|TNF	-7.43758	0	12	0	0	-26.3052	19.11133415
1	-8.41846	GO:0002791	M1	1	0	GO Biological Processes	19	regulation of peptide secretion	-8.41846	9.91541	9.870252	30242	200	183	12	6.557377	1.829835	134|150|1080|1268|1813|1956|3156|3358|3569|4843|5467|7124	ADORA1|ADRA2A|CFTR|CNR1|DRD2|EGFR|HMGCR|HTR2C|IL6|NOS2|PPARD|TNF	-6.84676	0	12	0	0	-26.3052	19.11133415
1	-7.61501	GO:0030072	M1	1	0	GO Biological Processes	19	peptide hormone secretion	-7.61501	8.40289	8.908192	30242	236	183	12	6.557377	1.829835	150|1080|1268|1813|1956|3156|3358|3569|4843|5467|7124|59341	ADRA2A|CFTR|CNR1|DRD2|EGFR|HMGCR|HTR2C|IL6|NOS2|PPARD|TNF|TRPV4	-6.118	0	12	0	0	-26.3052	19.11133415
1	-7.46232	GO:0090276	M1	1	0	GO Biological Processes	19	regulation of peptide hormone secretion	-7.46232	9.274618	9.068157	30242	196	183	11	6.010929	1.757049	150|1080|1268|1813|1956|3156|3358|3569|4843|5467|7124	ADRA2A|CFTR|CNR1|DRD2|EGFR|HMGCR|HTR2C|IL6|NOS2|PPARD|TNF	-5.97983	0	12	0	0	-26.3052	19.11133415
1	-6.0997	GO:0050796	M1	1	0	GO Biological Processes	19	regulation of insulin secretion	-6.0997	9.014009	8.053986	30242	165	183	9	4.918033	1.598524	150|1080|1268|1813|3156|3569|4843|5467|7124	ADRA2A|CFTR|CNR1|DRD2|HMGCR|IL6|NOS2|PPARD|TNF	-4.74319	0	12	0	0	-26.3052	19.11133415
1	-5.88093	GO:1903532	M1	1	0	GO Biological Processes	19	positive regulation of secretion by cell	-5.88093	6.446188	7.169398	30242	282	183	11	6.010929	1.757049	135|1080|1268|1588|1813|1956|4986|5467|6532|7040|7099	ADORA2A|CFTR|CNR1|CYP19A1|DRD2|EGFR|OPRK1|PPARD|SLC6A4|TGFB1|TLR4	-4.54708	0	12	0	0	-26.3052	19.11133415
1	-5.49903	GO:0030073	M1	1	0	GO Biological Processes	19	insulin secretion	-5.49903	7.627238	7.244121	30242	195	183	9	4.918033	1.598524	150|1080|1268|1813|3156|3569|4843|5467|7124	ADRA2A|CFTR|CNR1|DRD2|HMGCR|IL6|NOS2|PPARD|TNF	-4.19903	0	12	0	0	-26.3052	19.11133415
1	-3.98898	GO:0051222	M1	1	0	GO Biological	19	positive regulation of	-3.98898	4.908619	5.335343	30242	303	183	9	4.918033	1.598524	135|1080|1128|1956|54	ADORA2A|CFTR|CH	-2.87721	0	12	0	0	-26.3052	19.11133415

						Processes		protein transport										67|5743|7040|7099|7124	RM1|EGFR|PPARD|PTGS2|TGFB1|TLR4|TNF							
1	-3.82096	GO:1904951	M1	1	0	GO Biological Processes	19	positive regulation of establishment of protein localization	-3.82096	4.662418	5.130942	30242	319	183	9	4.918033	1.598524	135|1080|1128|1956|5467|5743|7040|7099|7124	ADORA2A|CFTR|CHRM1|EGFR|PPARD|PTGS2|TGFB1|TLR4|TNF	-2.72947	0	12	0	0	-26.3052	19.11133415
1	-3.71458	GO:0050714	M1	1	0	GO Biological Processes	19	positive regulation of protein secretion	-3.71458	7.237525	5.709389	30242	137	183	6	3.278689	1.316393	135|1080|1956|5467|7040|7099	ADORA2A|CFTR|EGFR|PPARD|TGFB1|TLR4	-2.6372	0	12	0	0	-26.3052	19.11133415
1	-3.32323	GO:0002793	M1	1	0	GO Biological Processes	19	positive regulation of peptide secretion	-3.32323	7.795134	5.468201	30242	106	183	5	2.73224	1.205087	134|1080|1813|1956|5467	ADORA1|CFTR|DRD2|EGFR|PPARD	-2.28652	0	12	0	0	-26.3052	19.11133415
1	-2.87883	GO:0046887	M1	1	0	GO Biological Processes	19	positive regulation of hormone secretion	-2.87883	6.212663	4.70082	30242	133	183	5	2.73224	1.205087	1080|1588|1813|1956|5467	CFTR|CYP19A1|DRD2|EGFR|PPARD	-1.88751	0	12	0	0	-26.3052	19.11133415
1	-2.44365	GO:0090277	M1	1	0	GO Biological Processes	19	positive regulation of peptide hormone secretion	-2.44365	6.417741	4.29743	30242	103	183	4	2.185792	1.080886	1080|1813|1956|5467	CFTR|DRD2|EGFR|PPARD	-1.49711	0	12	0	0	-26.3052	19.11133415
1	-26.0932	GO:0050727	M1	1	0	GO Biological Processes	19	regulation of inflammatory response	-26.0932	13.59439	19.50717	30242	389	183	32	17.48634	2.807934	100|134|135|185|351|834|1268|1910|1956|1991|2099|3558|3569|4314|4318|4363|4792|5138|5465|5467|5468|5734|5743|5970|6401|7099|7124|7132|10203|59341|114548|729230	ADA|ADORA1|ADORA2A|AGTR1|APP|CASP1|CNR1|EDNRB|EGFR|ELANE|ESR1|IL2|IL6|MMP3|MMP9|ABCC1|NFKBIA|PDE2A|PPARA|PPARD|PPARG|PTGER4|PTGS2|RELA|SELE|TLR4|TNF|TNFRSF1A|CALCRL|TRPV4|NLRP3|CCR2	-23.6297	0	13	1	1	-26.0932	18.62048796
1	-24.0367	GO:0031347	M1	1	0	GO Biological Processes	19	regulation of defense response	-24.0367	9.518793	16.79128	30242	625	183	36	19.67213	2.938551	100|134|135|185|351|834|1268|1813|1910|1956|1991|2099|3551|3558|3569|4314|4318|4363|4792|5138|5465|5467|5468|5591|5734|5743|5970|6401|7099|7124|7132|10203|29110|59341|114548|729230	ADA|ADORA1|ADORA2A|AGTR1|APP|CASP1|CNR1|DRD2|EDNRB|EGFR|ELANE|ESR1|IKBKB|IL2|IL6|MMP3|MMP9|ABCC1|NFKBIA|PDE2A|PPARA|PPARD|PPARG|PRKDC|PTGER4|PTGS2|RELA|SELE|TLR4|TNF|TNFRSF1A|CALCRL|TBK1|TRPV4|NLRP3|CCR2	-21.6732	0	13	0	0	-26.0932	18.62048796
1	-17.907	GO:0032103	M1	1	0	GO Biological Processes	19	positive regulation of response to external stimulus	-17.907	10.06248	14.71566	30242	427	183	26	14.20765	2.580833	185|351|1230|1268|1956|2147|2155|3551|3558|3569|3576|4363|4792|4988|5138|5159|5591|5734|5743|7040|7099|7124|7132|29110|59341|729230	AGTR1|APP|CCR1|CNR1|EGFR|F2|F7|IKBKB|IL2|IL6|CXCL8|ABCC1|NFKBIA|OPRM1|PDE2A|PDGFRB|PRKDC|PTGER4|PTGS2|TGFB1|TLR4|TNF|TNFRSF1A|TBK1|TRPV4|CCR2	-15.7641	0	13	0	0	-26.0932	18.62048796
1	-15.8885	GO:0001819	M1	1	0	GO Biological	19	positive regulation of	-15.8885	8.827822	13.31624	30242	468	183	25	13.6612	2.538762	150|351|834|1545|1813|	ADRA2A|APP|CASP1|	-13.8598	0	13	0	0	-26.0932	18.62048796

						Processes		cytokine production										1991|2149|3066|3357|3558|3569|4843|5142|5293|5734|5743|5970|7040|7099|7124|9475|29110|59341|114548|729230	CYP1B1|DRD2|ELANE|F2R|HDAC2|HTR2B|IL2|IL6|NOS2|PDE4B|PIK3CD|PTGER4|PTGS2|RELA|TGFB1|TLR4|TNF|ROCK2|TBK1|TRPV4|NLRP3|CCR2							
1	-15.3327	GO:0050729	M1	1	0	GO Biological Processes	19	positive regulation of inflammatory response	-15.3327	18.62049	16.42159	30242	142	183	16	8.743169	2.088053	185|351|1268|1956|3558|3569|4363|4792|5138|5734|5743|7099|7124|7132|59341|729230	AGTR1|APP|CNR1|EGFR|IL2|IL6|ABCC1|NFKBIA|PDE2A|PTGER4|PTGS2|TLR4|TNF|TNFRSF1A|TRPV4|CCR2	-13.327	0	13	0	0	-26.0932	18.62048796
1	-14.067	GO:0031349	M1	1	0	GO Biological Processes	19	positive regulation of defense response	-14.067	11.29453	13.45443	30242	278	183	19	10.38251	2.254873	185|351|1268|1956|3551|3558|3569|4363|4792|5138|5591|5734|5743|7099|7124|7132|29110|59341|729230	AGTR1|APP|CNR1|EGFR|IKBKB|IL2|IL6|ABCC1|NFKBIA|PDE2A|PRKDC|PTGER4|PTGS2|TLR4|TNF|TNFRSF1A|TBK1|TRPV4|CCR2	-12.119	0	13	0	0	-26.0932	18.62048796
1	-14.0032	GO:0001817	M1	1	0	GO Biological Processes	19	regulation of cytokine production	-14.0032	5.998058	11.17172	30242	799	183	29	15.84699	2.699496	150|351|834|1545|1813|1991|2147|2149|2335|3066|3357|3558|3569|4843|5142|5293|5465|5734|5743|5970|7040|7099|7124|8986|9475|29110|59341|114548|729230	ADRA2A|APP|CASP1|CYP1B1|DRD2|ELANE|F2|F2R|FN1|HDAC2|HTR2B|IL2|IL6|NOS2|PDE4B|PIK3CD|PPARA|PTGER4|PTGS2|RELA|TGFB1|TLR4|TNF|RPS6KA4|ROCK2|TBK1|TRPV4|NLRP3|CCR2	-12.06	0	13	0	0	-26.0932	18.62048796
1	-25.9543	GO:1901652	M1	1	0	GO Biological Processes	19	response to peptide	-25.9543	11.38581	18.41966	30242	508	183	35	19.12568	2.907289	154|185|328|351|760|890|1910|2155|2902|2903|2915|3383|3643|4221|4313|4314|4318|4363|4792|4914|4986|5290|5465|5468|5591|5743|5970|6513|7099|7157|7442|9475|10135|23621|59341	ADRB2|AGTR1|APEX1|APP|CA2|CCNA2|EDNRB|F7|GRIN1|GRIN2A|GRM5|ICAM1|INSR|MEN1|MMP2|MMP3|MMP9|ABCC1|NFKBIA|NTRK1|OPRK1|PIK3CA|PPARA|PPARG|PRKDC|PTGS2|RELA|SLC2A1|TLR4|TP53|TRPV1|ROCK2|NAMPT|BACE1|TRPV4	-23.4987	0	14	1	1	-25.9543	11.38580526
1	-16.2385	GO:1901653	M1	1	0	GO Biological Processes	19	cellular response to peptide	-16.2385	10.47082	14.16427	30242	363	183	23	12.56831	2.450458	154|185|328|351|760|890|2902|2903|2915|3383|3643|4221|4363|4914|5290|5468|5591|5970|7099|7157|9475|10135|23621	ADRB2|AGTR1|APEX1|APP|CA2|CCNA2|GRIN1|GRIN2A|GRM5|ICAM1|INSR|MEN1|ABCC1|NTRK1|PIK3CA|PPARG|PRKDC|RELA|TLR4|TP53|ROCK2|NAMPT|BACE1	-14.1761	0	14	0	0	-25.9543	11.38580526
1	-12.9103	GO:0043434	M1	1	0	GO Biological	19	response to peptide	-12.9103	8.342292	11.76568	30242	416	183	21	11.47541	2.356083	185|328|760|890|1910|2	AGTR1|APEX1|CA2|C	-11.0261	0	14	0	0	-25.9543	11.38580526

<table>
<tr><td></td><td></td><td></td><td></td><td></td><td></td><td></td><td></td><td></td><td></td><td></td><td></td><td></td><td></td><td></td><td></td><td></td><td></td><td></td><td></td><td></td><td></td><td></td><td></td><td></td><td></td><td></td></tr>
<tr><td></td><td></td><td></td><td></td><td></td><td></td><td>Processes</td><td></td><td>hormone</td><td></td><td></td><td></td><td></td><td></td><td></td><td></td><td></td><td></td><td>155|3383|3643|4221|4986|5290|5465|5468|5591|5743|5970|6513|7442|9475|10135|59341</td><td>CNA2|EDNRB|F7|ICAM1|INSR|MEN1|OPRK1|PIK3CA|PPARA|PPARG|PRKDC|PTGS2|RELA|SLC2A1|TRPV1|ROCK2|NAMPT|TRPV4</td><td></td><td></td><td></td><td></td><td></td><td></td><td></td></tr>
<tr><td>1</td><td>-12.6996</td><td>GO:0032870</td><td>M1</td><td>1</td><td>0</td><td>GO Biological Processes</td><td>19</td><td>cellular response to hormone stimulus</td><td>-12.6996</td><td>6.745177</td><td>10.97708</td><td>30242</td><td>588</td><td>183</td><td>24</td><td>13.11475</td><td>2.495327</td><td>150|185|328|367|760|890|1645|1956|2099|2100|2908|3383|3643|4221|5241|5290|5465|5467|5468|5591|5734|5970|9475|10135</td><td>ADRA2A|AGTR1|APEX1|AR|CA2|CCNA2|AKR1C1|EGFR|ESR1|ESR2|NR3C1|ICAM1|INSR|MEN1|PGR|PIK3CA|PPARA|PPARD|PPARG|PRKDC|PTGER4|RELA|ROCK2|NAMPT</td><td>-10.8341</td><td>0</td><td>14</td><td>0</td><td>0</td><td>-25.9543</td><td>11.38580526</td></tr>
<tr><td>1</td><td>-6.60856</td><td>GO:0071375</td><td>M1</td><td>1</td><td>0</td><td>GO Biological Processes</td><td>19</td><td>cellular response to peptide hormone stimulus</td><td>-6.60856</td><td>6.791377</td><td>7.759097</td><td>30242</td><td>292</td><td>183</td><td>12</td><td>6.557377</td><td>1.829835</td><td>185|328|760|890|3643|4221|5290|5468|5591|5970|9475|10135</td><td>AGTR1|APEX1|CA2|CCNA2|INSR|MEN1|PIK3CA|PPARG|PRKDC|RELA|ROCK2|NAMPT</td><td>-5.21123</td><td>0</td><td>14</td><td>0</td><td>0</td><td>-25.9543</td><td>11.38580526</td></tr>
<tr><td>1</td><td>-6.12896</td><td>GO:0032868</td><td>M1</td><td>1</td><td>0</td><td>GO Biological Processes</td><td>19</td><td>response to insulin</td><td>-6.12896</td><td>6.833929</td><td>7.456783</td><td>30242</td><td>266</td><td>183</td><td>11</td><td>6.010929</td><td>1.757049</td><td>3383|3643|4986|5290|5465|5468|5591|5970|6513|10135|59341</td><td>ICAM1|INSR|OPRK1|PIK3CA|PPARA|PPARG|PRKDC|RELA|SLC2A1|NAMPT|TRPV4</td><td>-4.77057</td><td>0</td><td>14</td><td>0</td><td>0</td><td>-25.9543</td><td>11.38580526</td></tr>
<tr><td>1</td><td>-2.7947</td><td>GO:0032869</td><td>M1</td><td>1</td><td>0</td><td>GO Biological Processes</td><td>19</td><td>cellular response to insulin stimulus</td><td>-2.7947</td><td>4.836785</td><td>4.300832</td><td>30242</td><td>205</td><td>183</td><td>6</td><td>3.278689</td><td>1.316393</td><td>3643|5290|5468|5591|5970|10135</td><td>INSR|PIK3CA|PPARG|PRKDC|RELA|NAMPT</td><td>-1.81276</td><td>0</td><td>14</td><td>0</td><td>0</td><td>-25.9543</td><td>11.38580526</td></tr>
<tr><td>1</td><td>-2.2345</td><td>GO:0008286</td><td>M1</td><td>1</td><td>0</td><td>GO Biological Processes</td><td>19</td><td>insulin receptor signaling pathway</td><td>-2.2345</td><td>5.601926</td><td>3.908055</td><td>30242</td><td>118</td><td>183</td><td>4</td><td>2.185792</td><td>1.080886</td><td>3643|5290|5970|10135</td><td>INSR|PIK3CA|RELA|NAMPT</td><td>-1.30616</td><td>0</td><td>14</td><td>0</td><td>0</td><td>-25.9543</td><td>11.38580526</td></tr>
<tr><td>1</td><td>-25.8159</td><td>GO:0042391</td><td>M1</td><td>1</td><td>0</td><td>GO Biological Processes</td><td>19</td><td>regulation of membrane potential</td><td>-25.8159</td><td>12.56561</td><td>18.93579</td><td>30242</td><td>434</td><td>183</td><td>33</td><td>18.03279</td><td>2.842012</td><td>134|135|148|153|154|351|596|775|834|1080|1128|1268|1813|1815|2554|2558|2566|2902|2903|2904|2915|3757|3778|4915|4985|4988|5024|6326|6331|6335|6336|7442|51305</td><td>ADORA1|ADORA2A|ADRA1A|ADRB1|ADRB2|APP|BCL2|CACNA1C|CASP1|CFTR|CHRM1|CNR1|DRD2|DRD4|GABRA1|GABRA5|GABRG2|GRIN1|GRIN2A|GRIN2B|GRM5|KCNH2|KCNMA1|NTRK2|OPRD1|OPRM1|P2RX3|SCN2A|SCN5A|SCN9A|SCN10A|TRPV1|KCNK9</td><td>-23.3681</td><td>0</td><td>15</td><td>1</td><td>1</td><td>-25.8159</td><td>55.0856102</td></tr>
<tr><td>1</td><td>-18.4206</td><td>GO:0060078</td><td>M1</td><td>1</td><td>0</td><td>GO Biological Processes</td><td>19</td><td>regulation of postsynaptic membrane potential</td><td>-18.4206</td><td>21.71258</td><td>18.95883</td><td>30242</td><td>137</td><td>183</td><td>18</td><td>9.836066</td><td>2.201413</td><td>134|135|153|154|351|1128|1813|1815|2554|2558|2566|2902|2903|2904|2915|4988|5024|7442</td><td>ADORA1|ADORA2A|ADRB1|ADRB2|APP|CHRM1|DRD2|DRD4|GABRA1|GABRA5|GABRG2|GRIN1|GRIN2A|GRIN2B|GRM5|OPRM1|P2RX3|TRPV1</td><td>-16.254</td><td>0</td><td>15</td><td>0</td><td>0</td><td>-25.8159</td><td>55.0856102</td></tr>
<tr><td>1</td><td>-11.6481</td><td>GO:0099565</td><td>M1</td><td>1</td><td>0</td><td>GO Biological</td><td>19</td><td>chemical synaptic</td><td>-11.6481</td><td>18.70832</td><td>14.2503</td><td>30242</td><td>106</td><td>183</td><td>12</td><td>6.557377</td><td>1.829835</td><td>134|135|154|351|1813|1</td><td>ADORA1|ADORA2A|</td><td>-9.84483</td><td>0</td><td>15</td><td>0</td><td>0</td><td>-25.8159</td><td>55.0856102</td></tr>
</table>

						Processes		transmission, postsynaptic										815|2902|2903|2904|4988|5024|7442	ADRB2|APP|DRD2|DRD4|GRIN1|GRIN2A|GRIN2B|OPRM1|P2RX3|TRPV1							
1	-10.583	GO:0060079	M1	1	0	GO Biological Processes	19	excitatory postsynaptic potential	-10.583	18.17825	13.42546	30242	100	183	11	6.010929	1.757049	134|135|154|351|1813|2902|2903|2904|4988|5024|7442	ADORA1|ADORA2A|ADRB2|APP|DRD2|GRIN1|GRIN2A|GRIN2B|OPRM1|P2RX3|TRPV1	-8.85827	0	15	0	0	-25.8159	55.0856102
1	-8.4678	GO:0048167	M1	1	0	GO Biological Processes	19	regulation of synaptic plasticity	-8.4678	10.01557	9.930773	30242	198	183	12	6.557377	1.829835	134|135|351|1813|2902|2903|2904|2915|4915|5024|5582|5743	ADORA1|ADORA2A|APP|DRD2|GRIN1|GRIN2A|GRIN2B|GRM5|NTRK2|P2RX3|PRKCG|PTGS2	-6.88987	0	15	0	0	-25.8159	55.0856102
1	-7.33254	GO:1905114	M1	1	0	GO Biological Processes	19	cell surface receptor signaling pathway involved in cell-cell signaling	-7.33254	5.117242	7.596297	30242	549	183	17	9.289617	2.145862	134|135|154|351|595|1453|1813|1815|1956|2263|2902|2903|2904|4988|5024|7040|7442	ADORA1|ADORA2A|ADRB2|APP|CCND1|CSNK1D|DRD2|DRD4|EGFR|FGFR2|GRIN1|GRIN2A|GRIN2B|OPRM1|P2RX3|TGFB1|TRPV1	-5.86491	0	15	0	0	-25.8159	55.0856102
1	-6.78227	GO:0060291	M1	1	0	GO Biological Processes	19	long-term synaptic potentiation	-6.78227	13.35409	9.606637	30242	99	183	8	4.371585	1.511428	134|135|351|1813|2903|2904|4915|5582	ADORA1|ADORA2A|APP|DRD2|GRIN2A|GRIN2B|NTRK2|PRKCG	-5.3666	0	15	0	0	-25.8159	55.0856102
1	-6.25448	GO:0035235	M1	1	0	GO Biological Processes	19	ionotropic glutamate receptor signaling pathway	-6.25448	30.60312	12.00727	30242	27	183	5	2.73224	1.205087	351|2891|2902|2903|2904	APP|GRIA2|GRIN1|GRIN2A|GRIN2B	-4.88197	0	15	0	0	-25.8159	55.0856102
1	-6.03729	GO:0007215	M1	1	0	GO Biological Processes	19	glutamate receptor signaling pathway	-6.03729	18.36187	9.963515	30242	54	183	6	3.278689	1.316393	351|2891|2902|2903|2904|2915	APP|GRIA2|GRIN1|GRIN2A|GRIN2B|GRM5	-4.68703	0	15	0	0	-25.8159	55.0856102
1	-4.74896	GO:0098976	M1	1	0	GO Biological Processes	19	excitatory chemical synaptic transmission	-4.74896	55.08561	12.6619	30242	9	183	3	1.639344	0.938686	2902|2903|2904	GRIN1|GRIN2A|GRIN2B	-3.54861	0	15	0	0	-25.8159	55.0856102
1	-2.64755	GO:0098815	M1	1	0	GO Biological Processes	19	modulation of excitatory postsynaptic potential	-2.64755	11.52955	5.391182	30242	43	183	3	1.639344	0.938686	154|351|2902	ADRB2|APP|GRIN1	-1.68278	0	15	0	0	-25.8159	55.0856102
1	-25.7351	GO:0008217	M1	1	0	GO Biological Processes	19	regulation of blood pressure	-25.7351	22.21194	22.64163	30242	186	183	25	13.6612	2.538762	134|146|147|148|151|153|154|185|367|1268|1636|1813|1814|1909|1910|2149|4842|4843|4846|5465|5468|5742|5743|7442|10135	ADORA1|ADRA1D|ADRA1B|ADRA1A|ADRA2B|ADRB1|ADRB2|AGTR1|AR|CNR1|ACE|DRD2|DRD3|EDNRA|EDNRB|F2R|NOS1|NOS2|NOS3|PPARA|PPARG|PTGS1|PTGS2|TRPV1|NAMPT	-23.295	0	16	1	1	-25.7351	46.11818528
1	-16.6617	GO:0045776	M1	1	0	GO Biological Processes	19	negative regulation of blood pressure	-16.6617	46.11819	23.10074	30242	43	183	12	6.557377	1.829835	134|148|153|154|1268|1813|1814|4842|4843|4846|5465|7442	ADORA1|ADRA1A|ADRB1|ADRB2|CNR1|DRD2|DRD3|NOS1|NOS2|NOS3|PPARA|TRPV1	-14.5608	0	16	0	0	-25.7351	46.11818528
1	-16.1385	GO:0042311	M1	1	0	GO Biological	19	vasodilation	-16.1385	42.19323	22.05178	30242	47	183	12	6.557377	1.829835	134|135|153|154|624|18	ADORA1|ADORA2A|	-14.0855	0	16	0	0	-25.7351	46.11818528

<table>
<tr><th></th><th></th><th></th><th></th><th></th><th></th><th></th><th></th><th></th><th></th><th></th><th></th><th></th><th></th><th></th><th></th><th></th><th></th><th></th><th></th><th></th><th></th><th></th><th></th><th></th><th></th><th></th></tr>
<tr><td></td><td></td><td></td><td></td><td></td><td></td><td>Processes</td><td></td><td></td><td></td><td></td><td></td><td></td><td></td><td></td><td></td><td></td><td></td><td>12|1910|1956|3778|4842|4846|5467</td><td>ADRB1|ADRB2|BDKRB2|DRD1|EDNRB|EGFR|KCNMA1|NOS1|NOS3|PPARD</td><td></td><td></td><td></td><td></td><td></td><td></td><td></td></tr>
<tr><td>1</td><td>-24.1754</td><td>GO:0010035</td><td>M1</td><td>1</td><td>0</td><td>GO Biological Processes</td><td>19</td><td>response to inorganic substance</td><td>-24.1754</td><td>10.07664</td><td>17.13062</td><td>30242</td><td>574</td><td>183</td><td>35</td><td>19.12568</td><td>2.907289</td><td>100|328|351|595|596|673|760|836|890|1543|1544|1545|1728|1813|1956|3066|3383|3569|3778|4129|4314|4318|4846|4914|5159|5290|5444|5743|5970|6331|6523|6531|8989|10280|23621</td><td>ADA|APEX1|APP|CCND1|BCL2|BRAF|CA2|CASP3|CCNA2|CYP1A1|CYP1A2|CYP1B1|NQO1|DRD2|EGFR|HDAC2|ICAM1|IL6|KCNMA1|MAOB|MMP3|MMP9|NOS3|NTRK1|PDGFRB|PIK3CA|PON1|PTGS2|RELA|SCN5A|SLC5A1|SLC6A3|TRPA1|SIGMAR1|BACE1</td><td>-21.8055</td><td>0</td><td>17</td><td>1</td><td>1</td><td>-24.1754</td><td>24.78852459</td></tr>
<tr><td>1</td><td>-11.785</td><td>GO:0010038</td><td>M1</td><td>1</td><td>0</td><td>GO Biological Processes</td><td>19</td><td>response to metal ion</td><td>-11.785</td><td>8.417908</td><td>11.24765</td><td>30242</td><td>373</td><td>183</td><td>19</td><td>10.38251</td><td>2.254873</td><td>351|595|596|673|760|836|1543|1544|1728|1813|1956|3383|3778|4129|4318|5743|6331|6531|23621</td><td>APP|CCND1|BCL2|BRAF|CA2|CASP3|CYP1A1|CYP1A2|NQO1|DRD2|EGFR|ICAM1|KCNMA1|MAOB|MMP9|PTGS2|SCN5A|SLC6A3|BACE1</td><td>-9.97639</td><td>0</td><td>17</td><td>0</td><td>0</td><td>-24.1754</td><td>24.78852459</td></tr>
<tr><td>1</td><td>-7.82349</td><td>GO:0071241</td><td>M1</td><td>1</td><td>0</td><td>GO Biological Processes</td><td>19</td><td>cellular response to inorganic substance</td><td>-7.82349</td><td>8.774699</td><td>9.153723</td><td>30242</td><td>226</td><td>183</td><td>12</td><td>6.557377</td><td>1.829835</td><td>351|673|890|1543|1544|1728|1956|4314|4318|5743|6331|23621</td><td>APP|BRAF|CCNA2|CYP1A1|CYP1A2|NQO1|EGFR|MMP3|MMP9|PTGS2|SCN5A|BACE1</td><td>-6.30511</td><td>0</td><td>17</td><td>0</td><td>0</td><td>-24.1754</td><td>24.78852459</td></tr>
<tr><td>1</td><td>-6.42855</td><td>GO:0071248</td><td>M1</td><td>1</td><td>0</td><td>GO Biological Processes</td><td>19</td><td>cellular response to metal ion</td><td>-6.42855</td><td>8.388672</td><td>8.118013</td><td>30242</td><td>197</td><td>183</td><td>10</td><td>5.464481</td><td>1.680144</td><td>351|673|1543|1544|1728|1956|4318|5743|6331|23621</td><td>APP|BRAF|CYP1A1|CYP1A2|NQO1|EGFR|MMP9|PTGS2|SCN5A|BACE1</td><td>-5.04414</td><td>0</td><td>17</td><td>0</td><td>0</td><td>-24.1754</td><td>24.78852459</td></tr>
<tr><td>1</td><td>-5.26368</td><td>GO:0046688</td><td>M1</td><td>1</td><td>0</td><td>GO Biological Processes</td><td>19</td><td>response to copper ion</td><td>-5.26368</td><td>19.67343</td><td>9.448909</td><td>30242</td><td>42</td><td>183</td><td>5</td><td>2.73224</td><td>1.205087</td><td>351|1543|1544|3383|23621</td><td>APP|CYP1A1|CYP1A2|ICAM1|BACE1</td><td>-3.99967</td><td>0</td><td>17</td><td>0</td><td>0</td><td>-24.1754</td><td>24.78852459</td></tr>
<tr><td>1</td><td>-4.62504</td><td>GO:0071280</td><td>M1</td><td>1</td><td>0</td><td>GO Biological Processes</td><td>19</td><td>cellular response to copper ion</td><td>-4.62504</td><td>23.60812</td><td>9.338469</td><td>30242</td><td>28</td><td>183</td><td>4</td><td>2.185792</td><td>1.080886</td><td>351|1543|1544|23621</td><td>APP|CYP1A1|CYP1A2|BACE1</td><td>-3.43978</td><td>0</td><td>17</td><td>0</td><td>0</td><td>-24.1754</td><td>24.78852459</td></tr>
<tr><td>1</td><td>-3.63764</td><td>GO:0010042</td><td>M1</td><td>1</td><td>0</td><td>GO Biological Processes</td><td>19</td><td>response to manganese ion</td><td>-3.63764</td><td>24.78852</td><td>8.303425</td><td>30242</td><td>20</td><td>183</td><td>3</td><td>1.639344</td><td>0.938686</td><td>351|5743|23621</td><td>APP|PTGS2|BACE1</td><td>-2.56974</td><td>0</td><td>17</td><td>0</td><td>0</td><td>-24.1754</td><td>24.78852459</td></tr>
<tr><td>1</td><td>-3.39607</td><td>GO:0010288</td><td>M1</td><td>1</td><td>0</td><td>GO Biological Processes</td><td>19</td><td>response to lead ion</td><td>-3.39607</td><td>20.6571</td><td>7.516725</td><td>30242</td><td>24</td><td>183</td><td>3</td><td>1.639344</td><td>0.938686</td><td>351|5743|23621</td><td>APP|PTGS2|BACE1</td><td>-2.35299</td><td>0</td><td>17</td><td>0</td><td>0</td><td>-24.1754</td><td>24.78852459</td></tr>
<tr><td>1</td><td>-24.0005</td><td>GO:0019233</td><td>M1</td><td>1</td><td>0</td><td>GO Biological Processes</td><td>19</td><td>sensory perception of pain</td><td>-24.0005</td><td>32.0887</td><td>24.66001</td><td>30242</td><td>103</td><td>183</td><td>20</td><td>10.92896</td><td>2.306387</td><td>134|152|1268|1910|2149|2903|3356|4914|4985|4986|4988|5024|5743|6335|6336|6347|7442|8989|23621|729230</td><td>ADORA1|ADRA2C|CNR1|EDNRB|F2R|GRIN2A|HTR2A|NTRK1|OPRD1|OPRK1|OPRM1|P2RX3|PTGS2|SCN9A|SCN10A|CCL2|TRPV1|TRPA1|BACE1|CCR2</td><td>-21.6434</td><td>0</td><td>18</td><td>1</td><td>1</td><td>-24.0005</td><td>66.10273224</td></tr>
<tr><td>1</td><td>-12.5767</td><td>GO:0031644</td><td>M1</td><td>1</td><td>0</td><td>GO Biological Processes</td><td>19</td><td>regulation of nervous system process</td><td>-12.5767</td><td>16.06664</td><td>14.14051</td><td>30242</td><td>144</td><td>183</td><td>14</td><td>7.650273</td><td>1.964856</td><td>134|152|154|351|1910|2149|2902|3358|4846|49</td><td>ADORA1|ADRA2C|ADRB2|APP|EDNRB|F2</td><td>-10.7212</td><td>0</td><td>18</td><td>0</td><td>0</td><td>-24.0005</td><td>66.10273224</td></tr>
</table>

																		85|4986|4988|5024|9475	R|GRIN1|HTR2C|NOS3|OPRD1|OPRK1|OPRM1|P2RX3|ROCK2							
1	-11.9557	GO:0007631	M1	1	0	GO Biological Processes	19	feeding behavior	-11.9557	19.83082	14.71701	30242	100	183	12	6.557377	1.829835	135|351|1268|1813|3061|3062|3351|3358|4915|4985|4986|4988	ADORA2A|APP|CNR1|DRD2|HCRTR1|HCRTR2|HTR1B|HTR2C|NTRK2|OPRD1|OPRK1|OPRM1	-10.1345	0	18	0	0	-24.0005	66.10273224
1	-10.1901	GO:0051930	M1	1	0	GO Biological Processes	19	regulation of sensory perception of pain	-10.1901	34.79091	16.26282	30242	38	183	8	4.371585	1.511428	134|152|1910|2149|4985|4986|4988|5024	ADORA1|ADRA2C|EDNRB|F2R|OPRD1|OPRK1|OPRM1|P2RX3	-8.48686	0	18	0	0	-24.0005	66.10273224
1	-10.0926	GO:0051931	M1	1	0	GO Biological Processes	19	regulation of sensory perception	-10.0926	33.89884	16.04073	30242	39	183	8	4.371585	1.511428	134|152|1910|2149|4985|4986|4988|5024	ADORA1|ADRA2C|EDNRB|F2R|OPRD1|OPRK1|OPRM1|P2RX3	-8.39769	0	18	0	0	-24.0005	66.10273224
1	-6.57703	GO:0038003	M1	1	0	GO Biological Processes	19	G protein-coupled opioid receptor signaling pathway	-6.57703	66.10273	16.06579	30242	10	183	4	2.185792	1.080886	4985|4986|4988|10280	OPRD1|OPRK1|OPRM1|SIGMAR1	-5.18107	0	18	0	0	-24.0005	66.10273224
1	-4.33034	GO:0007218	M1	1	0	GO Biological Processes	19	neuropeptide signaling pathway	-4.33034	9.35416	6.722786	30242	106	183	6	3.278689	1.316393	3061|3062|4985|4986|4988|10800	HCRTR1|HCRTR2|OPRD1|OPRK1|OPRM1|CYSLTR1	-3.18628	0	18	0	0	-24.0005	66.10273224
1	-4.18252	GO:0042755	M1	1	0	GO Biological Processes	19	eating behavior	-4.18252	18.36187	8.132751	30242	36	183	4	2.185792	1.080886	135|4985|4986|4988	ADORA2A|OPRD1|OPRK1|OPRM1	-3.05249	0	18	0	0	-24.0005	66.10273224
1	-23.4236	GO:0031667	M1	1	0	GO Biological Processes	19	response to nutrient levels	-23.4236	11.15658	17.38994	30242	474	183	32	17.48634	2.807934	100|153|154|249|595|596|1268|1543|1723|1728|1956|2155|3156|3358|3383|4914|4988|5444|5465|5467|5468|5582|5743|5970|6513|6532|7157|7298|7421|7442|10135|59341	ADA|ADRB1|ADRB2|ALPL|CCND1|BCL2|CNR1|CYP1A1|DHODH|NQO1|EGFR|F7|HMGCR|HTR2C|ICAM1|NTRK1|OPRM1|PON1|PPARA|PPARD|PPARG|PRKCG|PTGS2|RELA|SLC2A1|SLC6A4|TP53|TYMS|VDR|TRPV1|NAMPT|TRPV4	-21.0727	0	19	1	1	-23.4236	19.54650685
1	-22.5803	GO:0009991	M1	1	0	GO Biological Processes	19	response to extracellular stimulus	-22.5803	10.47172	16.74798	30242	505	183	32	17.48634	2.807934	100|153|154|249|595|596|1268|1543|1723|1728|1956|2155|3156|3358|3383|4914|4988|5444|5465|5467|5468|5582|5743|5970|6513|6532|7157|7298|7421|7442|10135|59341	ADA|ADRB1|ADRB2|ALPL|CCND1|BCL2|CNR1|CYP1A1|DHODH|NQO1|EGFR|F7|HMGCR|HTR2C|ICAM1|NTRK1|OPRM1|PON1|PPARA|PPARD|PPARG|PRKCG|PTGS2|RELA|SLC2A1|SLC6A4|TP53|TYMS|VDR|TRPV1|NAMPT|TRPV4	-20.2651	0	19	0	0	-23.4236	19.54650685
1	-16.8907	GO:0007568	M1	1	0	GO Biological Processes	19	aging	-16.8907	11.21212	14.75348	30242	339	183	23	12.56831	2.450458	100|148|328|351|472|596|1268|1543|1728|1910|2915|3156|3356|3383|4914|5159|5591|5743|5970|6531|7157|7298|10135	ADA|ADRA1A|APEX1|APP|ATM|BCL2|CNR1|CYP1A1|NQO1|EDNRB|GRM5|HMGCR|HTR2A|ICAM1|NTRK1|PDGFRB|PRKDC|PTGS2|RELA|SLC6A3|T	-14.7847	0	19	0	0	-23.4236	19.54650685

<table>
<tr><th></th><th></th><th></th><th></th><th></th><th></th><th></th><th></th><th></th><th></th><th></th><th></th><th></th><th></th><th></th><th></th><th></th><th></th><th></th><th></th><th></th><th></th><th></th><th></th><th></th><th></th><th></th></tr>
<tr><td></td><td></td><td></td><td></td><td></td><td></td><td></td><td></td><td></td><td></td><td></td><td></td><td></td><td></td><td></td><td></td><td></td><td></td><td></td><td>P53|TYMS|NAMPT</td><td></td><td></td><td></td><td></td><td></td><td></td><td></td></tr>
<tr><td>1</td><td>-13.9208</td><td>GO:0007584</td><td>M1</td><td>1</td><td>0</td><td>GO Biological Processes</td><td>19</td><td>response to nutrient</td><td>-13.9208</td><td>15.19603</td><td>14.65297</td><td>30242</td><td>174</td><td>183</td><td>16</td><td>8.743169</td><td>2.088053</td><td>100|249|595|1268|1543|1728|1956|2155|3156|5467|5468|5743|5970|6532|7298|7421</td><td>ADA|ALPL|CCND1|CNR1|CYP1A1|NQO1|EGFR|F7|HMGCR|PPARD|PPARG|PTGS2|RELA|SLC6A4|TYMS|VDR</td><td>-11.9849</td><td>0</td><td>19</td><td>0</td><td>0</td><td>-23.4236</td><td>19.54650685</td></tr>
<tr><td>1</td><td>-10.9332</td><td>GO:0033273</td><td>M1</td><td>1</td><td>0</td><td>GO Biological Processes</td><td>19</td><td>response to vitamin</td><td>-10.9332</td><td>19.54651</td><td>13.97666</td><td>30242</td><td>93</td><td>183</td><td>11</td><td>6.010929</td><td>1.757049</td><td>100|249|595|1543|1956|2155|5467|5743|5970|7298|7421</td><td>ADA|ALPL|CCND1|CYP1A1|EGFR|F7|PPARD|PTGS2|RELA|TYMS|VDR</td><td>-9.18433</td><td>0</td><td>19</td><td>0</td><td>0</td><td>-23.4236</td><td>19.54650685</td></tr>
<tr><td>1</td><td>-22.5805</td><td>GO:0070482</td><td>M1</td><td>1</td><td>0</td><td>GO Biological Processes</td><td>19</td><td>response to oxygen levels</td><td>-22.5805</td><td>13.33485</td><td>18.03165</td><td>30242</td><td>347</td><td>183</td><td>28</td><td>15.30055</td><td>2.661143</td><td>100|134|596|834|836|890|1543|1813|1909|2155|3066|3383|3778|4313|4842|4843|4985|5024|5159|5465|5467|5743|6326|6513|6532|7157|9475|10135</td><td>ADA|ADORA1|BCL2|CASP1|CASP3|CCNA2|CYP1A1|DRD2|EDNRA|F7|HDAC2|ICAM1|KCNMA1|MMP2|NOS1|NOS2|OPRD1|P2RX3|PDGFRB|PPARA|PPARD|PTGS2|SCN2A|SLC2A1|SLC6A4|TP53|ROCK2|NAMPT</td><td>-20.2651</td><td>0</td><td>20</td><td>1</td><td>1</td><td>-22.5805</td><td>13.45739663</td></tr>
<tr><td>1</td><td>-20.9763</td><td>GO:0036293</td><td>M1</td><td>1</td><td>0</td><td>GO Biological Processes</td><td>19</td><td>response to decreased oxygen levels</td><td>-20.9763</td><td>13.34372</td><td>17.37519</td><td>30242</td><td>322</td><td>183</td><td>26</td><td>14.20765</td><td>2.580833</td><td>100|134|596|834|836|890|1543|1813|1909|2155|3383|3778|4313|4842|4843|4985|5024|5465|5467|5743|6326|6513|6532|7157|9475|10135</td><td>ADA|ADORA1|BCL2|CASP1|CASP3|CCNA2|CYP1A1|DRD2|EDNRA|F7|ICAM1|KCNMA1|MMP2|NOS1|NOS2|OPRD1|P2RX3|PPARA|PPARD|PTGS2|SCN2A|SLC2A1|SLC6A4|TP53|ROCK2|NAMPT</td><td>-18.7297</td><td>0</td><td>20</td><td>0</td><td>0</td><td>-22.5805</td><td>13.45739663</td></tr>
<tr><td>1</td><td>-20.2635</td><td>GO:0001666</td><td>M1</td><td>1</td><td>0</td><td>GO Biological Processes</td><td>19</td><td>response to hypoxia</td><td>-20.2635</td><td>13.4574</td><td>17.11761</td><td>30242</td><td>307</td><td>183</td><td>25</td><td>13.6612</td><td>2.538762</td><td>100|134|596|834|836|890|1543|1813|1909|2155|3383|3778|4313|4842|4843|4985|5024|5465|5467|5743|6326|6513|6532|7157|9475</td><td>ADA|ADORA1|BCL2|CASP1|CASP3|CCNA2|CYP1A1|DRD2|EDNRA|F7|ICAM1|KCNMA1|MMP2|NOS1|NOS2|OPRD1|P2RX3|PPARA|PPARD|PTGS2|SCN2A|SLC2A1|SLC6A4|TP53|ROCK2</td><td>-18.0265</td><td>0</td><td>20</td><td>0</td><td>0</td><td>-22.5805</td><td>13.45739663</td></tr>
<tr><td>1</td><td>-7.24664</td><td>GO:0036294</td><td>M1</td><td>1</td><td>0</td><td>GO Biological Processes</td><td>19</td><td>cellular response to decreased oxygen levels</td><td>-7.24664</td><td>10.2644</td><td>9.196455</td><td>30242</td><td>161</td><td>183</td><td>10</td><td>5.464481</td><td>1.680144</td><td>596|890|3383|4985|5467|5743|6326|7157|9475|10135</td><td>BCL2|CCNA2|ICAM1|OPRD1|PPARD|PTGS2|SCN2A|TP53|ROCK2|NAMPT</td><td>-5.79023</td><td>0</td><td>20</td><td>0</td><td>0</td><td>-22.5805</td><td>13.45739663</td></tr>
<tr><td>1</td><td>-6.85991</td><td>GO:0071453</td><td>M1</td><td>1</td><td>0</td><td>GO Biological Processes</td><td>19</td><td>cellular response to oxygen levels</td><td>-6.85991</td><td>9.336544</td><td>8.679175</td><td>30242</td><td>177</td><td>183</td><td>10</td><td>5.464481</td><td>1.680144</td><td>596|890|3383|4985|5467|5743|6326|7157|9475|10135</td><td>BCL2|CCNA2|ICAM1|OPRD1|PPARD|PTGS2|SCN2A|TP53|ROCK2|NAMPT</td><td>-5.43331</td><td>0</td><td>20</td><td>0</td><td>0</td><td>-22.5805</td><td>13.45739663</td></tr>
<tr><td>1</td><td>-6.42392</td><td>GO:0071456</td><td>M1</td><td>1</td><td>0</td><td>GO Biological Processes</td><td>19</td><td>cellular response to hypoxia</td><td>-6.42392</td><td>9.849745</td><td>8.506233</td><td>30242</td><td>151</td><td>183</td><td>9</td><td>4.918033</td><td>1.598524</td><td>596|890|3383|4985|5467|5743|6326|7157|9475</td><td>BCL2|CCNA2|ICAM1|OPRD1|PPARD|PTGS2|SCN2A|TP53|ROCK2</td><td>-5.04018</td><td>0</td><td>20</td><td>0</td><td>0</td><td>-22.5805</td><td>13.45739663</td></tr>
</table>

<table>
<tr><td>1</td><td>-22.0317</td><td>GO:0033555</td><td>M1</td><td>1</td><td>0</td><td>GO Biological Processes</td><td>19</td><td>multicellular organismal response to stress</td><td>-22.0317</td><td>39.01897</td><td>25.20086</td><td>30242</td><td>72</td><td>183</td><td>17</td><td>9.289617</td><td>2.145862</td><td>150|153|596|1815|1910|2558|3350|3358|4842|4914|5024|5582|5979|6335|6530|7442|8989</td><td>ADRA2A|ADRB1|BCL2|DRD4|EDNRB|GABRA5|HTR1A|HTR2C|NOS1|NTRK1|P2RX3|PRKCG|RET|SCN9A|SLC6A2|TRPV1|TRPA1</td><td>-19.7333</td><td>0</td><td>21</td><td>1</td><td>1</td><td>-22.0317</td><td>51.2866026</td></tr>
<tr><td>1</td><td>-13.0942</td><td>GO:0048265</td><td>M1</td><td>1</td><td>0</td><td>GO Biological Processes</td><td>19</td><td>response to pain</td><td>-13.0942</td><td>51.2866</td><td>21.13931</td><td>30242</td><td>29</td><td>183</td><td>9</td><td>4.918033</td><td>1.598524</td><td>1910|4914|5024|5582|5979|6335|6530|7442|8989</td><td>EDNRB|NTRK1|P2RX3|PRKCG|RET|SCN9A|SLC6A2|TRPV1|TRPA1</td><td>-11.1992</td><td>0</td><td>21</td><td>0</td><td>0</td><td>-22.0317</td><td>51.2866026</td></tr>
<tr><td>1</td><td>-4.9788</td><td>GO:0060384</td><td>M1</td><td>1</td><td>0</td><td>GO Biological Processes</td><td>19</td><td>innervation</td><td>-4.9788</td><td>28.74032</td><td>10.38417</td><td>30242</td><td>23</td><td>183</td><td>4</td><td>2.185792</td><td>1.080886</td><td>2558|4914|5582|5979</td><td>GABRA5|NTRK1|PRKCG|RET</td><td>-3.74566</td><td>0</td><td>21</td><td>0</td><td>0</td><td>-22.0317</td><td>51.2866026</td></tr>
<tr><td>1</td><td>-2.90504</td><td>GO:0021675</td><td>M1</td><td>1</td><td>0</td><td>GO Biological Processes</td><td>19</td><td>nerve development</td><td>-2.90504</td><td>8.58477</td><td>5.199631</td><td>30242</td><td>77</td><td>183</td><td>4</td><td>2.185792</td><td>1.080886</td><td>2558|4914|5582|5979</td><td>GABRA5|NTRK1|PRKCG|RET</td><td>-1.911</td><td>0</td><td>21</td><td>0</td><td>0</td><td>-22.0317</td><td>51.2866026</td></tr>
<tr><td>1</td><td>-21.4062</td><td>GO:0009611</td><td>M1</td><td>1</td><td>0</td><td>GO Biological Processes</td><td>19</td><td>response to wounding</td><td>-21.4062</td><td>9.580106</td><td>15.87426</td><td>30242</td><td>552</td><td>183</td><td>32</td><td>17.48634</td><td>2.807934</td><td>135|140|150|151|152|596|836|1230|1543|1813|1956|2147|2149|2155|2263|2335|2903|3156|3569|4846|4914|4988|5159|5290|5291|5465|5467|7040|7046|7099|7124|729230</td><td>ADORA2A|ADORA3|ADRA2A|ADRA2B|ADRA2C|BCL2|CASP3|CCR1|CYP1A1|DRD2|EGFR|F2|F2R|F7|FGFR2|FN1|GRIN2A|HMGCR|IL6|NOS3|NTRK1|OPRM1|PDGFRB|PIK3CA|PIK3CB|PPARA|PPARD|TGFB1|TGFBR1|TLR4|TNF|CCR2</td><td>-19.1345</td><td>0</td><td>22</td><td>1</td><td>1</td><td>-21.4062</td><td>14.65988013</td></tr>
<tr><td>1</td><td>-20.2645</td><td>GO:0050878</td><td>M1</td><td>1</td><td>0</td><td>GO Biological Processes</td><td>19</td><td>regulation of body fluid levels</td><td>-20.2645</td><td>11.68046</td><td>16.39128</td><td>30242</td><td>382</td><td>183</td><td>27</td><td>14.7541</td><td>2.621607</td><td>100|134|135|150|151|152|595|1080|1128|1131|1723|1813|1910|1956|2147|2149|2155|2335|3569|4846|4986|5290|5291|6531|7099|7421|59341</td><td>ADA|ADORA1|ADORA2A|ADRA2A|ADRA2B|ADRA2C|CCND1|CFTR|CHRM1|CHRM3|DHODH|DRD2|EDNRB|EGFR|F2|F2R|F7|FN1|IL6|NOS3|OPRK1|PIK3CA|PIK3CB|SLC6A3|TLR4|VDR|TRPV4</td><td>-18.0265</td><td>0</td><td>22</td><td>0</td><td>0</td><td>-21.4062</td><td>14.65988013</td></tr>
<tr><td>1</td><td>-17.3097</td><td>GO:0040017</td><td>M1</td><td>1</td><td>0</td><td>GO Biological Processes</td><td>19</td><td>positive regulation of locomotion</td><td>-17.3097</td><td>8.027551</td><td>13.53168</td><td>30242</td><td>597</td><td>183</td><td>29</td><td>15.84699</td><td>2.699496</td><td>150|351|472|596|1230|1812|1956|2149|2155|2335|3383|3569|3576|3643|4318|4846|4986|5159|5291|5293|5743|5979|7040|7046|7099|7124|9475|59341|729230</td><td>ADRA2A|APP|ATM|BCL2|CCR1|DRD1|EGFR|F2R|F7|FN1|ICAM1|IL6|CXCL8|INSR|MMP9|NOS3|OPRK1|PDGFRB|PIK3CB|PIK3CD|PTGS2|RET|TGFB1|TGFBR1|TLR4|TNF|ROCK2|TRPV4|CCR2</td><td>-15.1892</td><td>0</td><td>22</td><td>0</td><td>0</td><td>-21.4062</td><td>14.65988013</td></tr>
<tr><td>1</td><td>-17.0972</td><td>GO:0030335</td><td>M1</td><td>1</td><td>0</td><td>GO Biological Processes</td><td>19</td><td>positive regulation of cell migration</td><td>-17.0972</td><td>8.307345</td><td>13.58173</td><td>30242</td><td>557</td><td>183</td><td>28</td><td>15.30055</td><td>2.661143</td><td>150|351|472|596|1230|1812|1956|2149|2155|2335|3383|3569|3576|3643|4318|4846|5159|5291|5293|5743|5979|7040|7046|7099|7124|9475|59341|729230</td><td>ADRA2A|APP|ATM|BCL2|CCR1|DRD1|EGFR|F2R|F7|FN1|ICAM1|IL6|CXCL8|INSR|MMP9|NOS3|PDGFRB|PIK3CB|PIK3CD|PTGS2|RET|TGFB1|TGFBR1|</td><td>-14.9876</td><td>0</td><td>22</td><td>0</td><td>0</td><td>-21.4062</td><td>14.65988013</td></tr>
</table>

<table>
<tr><td></td><td></td><td></td><td></td><td></td><td></td><td></td><td></td><td></td><td></td><td></td><td></td><td></td><td></td><td></td><td></td><td></td><td></td><td></td><td>TLR4|TNF|ROCK2|TRPV4|CCR2</td><td></td><td></td><td></td><td></td><td></td><td></td><td></td></tr>
<tr><td>1</td><td>-16.5857</td><td>GO:2000147</td><td>M1</td><td>1</td><td>0</td><td>GO Biological Processes</td><td>19</td><td>positive regulation of cell motility</td><td>-16.5857</td><td>7.936863</td><td>13.1964</td><td>30242</td><td>583</td><td>183</td><td>28</td><td>15.30055</td><td>2.661143</td><td>150|351|472|596|1230|1812|1956|2149|2155|2335|3383|3569|3576|3643|4318|4846|5159|5291|5293|5743|5979|7040|7046|7099|7124|9475|59341|729230</td><td>ADRA2A|APP|ATM|BCL2|CCR1|DRD1|EGFR|F2R|F7|FN1|ICAM1|IL6|CXCL8|INSR|MMP9|NOS3|PDGFRB|PIK3CB|PIK3CD|PTGS2|RET|TGFB1|TGFBR1|TLR4|TNF|ROCK2|TRPV4|CCR2</td><td>-14.4901</td><td>0</td><td>22</td><td>0</td><td>0</td><td>-21.4062</td><td>14.65988013</td></tr>
<tr><td>1</td><td>-16.3584</td><td>GO:0051272</td><td>M1</td><td>1</td><td>0</td><td>GO Biological Processes</td><td>19</td><td>positive regulation of cellular component movement</td><td>-16.3584</td><td>7.776792</td><td>13.02653</td><td>30242</td><td>595</td><td>183</td><td>28</td><td>15.30055</td><td>2.661143</td><td>150|351|472|596|1230|1812|1956|2149|2155|2335|3383|3569|3576|3643|4318|4846|5159|5291|5293|5743|5979|7040|7046|7099|7124|9475|59341|729230</td><td>ADRA2A|APP|ATM|BCL2|CCR1|DRD1|EGFR|F2R|F7|FN1|ICAM1|IL6|CXCL8|INSR|MMP9|NOS3|PDGFRB|PIK3CB|PIK3CD|PTGS2|RET|TGFB1|TGFBR1|TLR4|TNF|ROCK2|TRPV4|CCR2</td><td>-14.283</td><td>0</td><td>22</td><td>0</td><td>0</td><td>-21.4062</td><td>14.65988013</td></tr>
<tr><td>1</td><td>-15.8247</td><td>GO:0042060</td><td>M1</td><td>1</td><td>0</td><td>GO Biological Processes</td><td>19</td><td>wound healing</td><td>-15.8247</td><td>9.35416</td><td>13.51708</td><td>30242</td><td>424</td><td>183</td><td>24</td><td>13.11475</td><td>2.495327</td><td>135|150|151|152|836|1956|2147|2149|2155|2263|2335|3156|3569|4846|4988|5159|5290|5291|5465|5467|7040|7046|7099|7124</td><td>ADORA2A|ADRA2A|ADRA2B|ADRA2C|CASP3|EGFR|F2|F2R|F7|FGFR2|FN1|HMGCR|IL6|NOS3|OPRM1|PDGFRB|PIK3CA|PIK3CB|PPARA|PPARD|TGFB1|TGFBR1|TLR4|TNF</td><td>-13.8019</td><td>0</td><td>22</td><td>0</td><td>0</td><td>-21.4062</td><td>14.65988013</td></tr>
<tr><td>1</td><td>-9.5604</td><td>GO:0030168</td><td>M1</td><td>1</td><td>0</td><td>GO Biological Processes</td><td>19</td><td>platelet activation</td><td>-9.5604</td><td>14.65988</td><td>11.89272</td><td>30242</td><td>124</td><td>183</td><td>11</td><td>6.010929</td><td>1.757049</td><td>150|151|152|2147|2149|2335|3569|4846|5290|5291|7099</td><td>ADRA2A|ADRA2B|ADRA2C|F2|F2R|FN1|IL6|NOS3|PIK3CA|PIK3CB|TLR4</td><td>-7.91342</td><td>0</td><td>22</td><td>0</td><td>0</td><td>-21.4062</td><td>14.65988013</td></tr>
<tr><td>1</td><td>-9.04348</td><td>GO:0007596</td><td>M1</td><td>1</td><td>0</td><td>GO Biological Processes</td><td>19</td><td>blood coagulation</td><td>-9.04348</td><td>9.854765</td><td>10.23782</td><td>30242</td><td>218</td><td>183</td><td>13</td><td>7.103825</td><td>1.898976</td><td>135|150|151|152|2147|2149|2155|2335|3569|4846|5290|5291|7099</td><td>ADORA2A|ADRA2A|ADRA2B|ADRA2C|F2|F2R|F7|FN1|IL6|NOS3|PIK3CA|PIK3CB|TLR4</td><td>-7.42614</td><td>0</td><td>22</td><td>0</td><td>0</td><td>-21.4062</td><td>14.65988013</td></tr>
<tr><td>1</td><td>-8.9232</td><td>GO:0007599</td><td>M1</td><td>1</td><td>0</td><td>GO Biological Processes</td><td>19</td><td>hemostasis</td><td>-8.9232</td><td>9.633806</td><td>10.09701</td><td>30242</td><td>223</td><td>183</td><td>13</td><td>7.103825</td><td>1.898976</td><td>135|150|151|152|2147|2149|2155|2335|3569|4846|5290|5291|7099</td><td>ADORA2A|ADRA2A|ADRA2B|ADRA2C|F2|F2R|F7|FN1|IL6|NOS3|PIK3CA|PIK3CB|TLR4</td><td>-7.31381</td><td>0</td><td>22</td><td>0</td><td>0</td><td>-21.4062</td><td>14.65988013</td></tr>
<tr><td>1</td><td>-8.9232</td><td>GO:0050817</td><td>M1</td><td>1</td><td>0</td><td>GO Biological Processes</td><td>19</td><td>coagulation</td><td>-8.9232</td><td>9.633806</td><td>10.09701</td><td>30242</td><td>223</td><td>183</td><td>13</td><td>7.103825</td><td>1.898976</td><td>135|150|151|152|2147|2149|2155|2335|3569|4846|5290|5291|7099</td><td>ADORA2A|ADRA2A|ADRA2B|ADRA2C|F2|F2R|F7|FN1|IL6|NOS3|PIK3CA|PIK3CB|TLR4</td><td>-7.31381</td><td>0</td><td>22</td><td>0</td><td>0</td><td>-21.4062</td><td>14.65988013</td></tr>
<tr><td>1</td><td>-20.9775</td><td>GO:0010942</td><td>M1</td><td>1</td><td>0</td><td>GO Biological Processes</td><td>19</td><td>positive regulation of cell death</td><td>-20.9775</td><td>8.824394</td><td>15.33414</td><td>30242</td><td>618</td><td>183</td><td>33</td><td>18.03279</td><td>2.842012</td><td>134|135|472|836|1268|1545|1723|1728|2903|2904|3156|3569|3778|431</td><td>ADORA1|ADORA2A|ATM|CASP3|CNR1|CYP1B1|DHODH|NQO</td><td>-18.7297</td><td>0</td><td>23</td><td>1</td><td>1</td><td>-20.9775</td><td>8.824393867</td></tr>
</table>

																		4|4318|4843|4914|5159|5291|5293|5468|5591|5743|5979|6347|7046|7099|7124|7132|7157|7421|7442|23621	1|GRIN2A|GRIN2B|HMGCR|IL6|KCNMA1|MMP3|MMP9|NOS2|NTRK1|PDGFRB|PIK3CB|PIK3CD|PPARG|PRKDC|PTGS2|RET|CCL2|TGFBR1|TLR4|TNF|TNFRSF1A|TP53|VDR|TRPV1|BACE1							
1	-18.1601	GO:0043068	M1	1	0	GO Biological Processes	19	positive regulation of programmed cell death	-18.1601	8.635042	14.16482	30242	555	183	29	15.84699	2.699496	135|472|836|1268|1545|1723|1728|2903|3156|3569|3778|4318|4843|4914|5159|5291|5293|5468|5591|5743|5979|6347|7046|7124|7132|7157|7421|7442|23621	ADORA2A|ATM|CASP3|CNR1|CYP1B1|DHODH|NQO1|GRIN2A|HMGCR|IL6|KCNMA1|MMP9|NOS2|NTRK1|PDGFRB|PIK3CB|PIK3CD|PPARG|PRKDC|PTGS2|RET|CCL2|TGFBR1|TNF|TNFRSF1A|TP53|VDR|TRPV1|BACE1	-16.0095	0	23	0	0	-20.9775	8.824393867
1	-16.4503	GO:0043065	M1	1	0	GO Biological Processes	19	positive regulation of apoptotic process	-16.4503	8.278171	13.3031	30242	539	183	27	14.7541	2.621607	135|472|836|1268|1545|1723|1728|2903|3156|3569|3778|4318|5159|5291|5293|5468|5591|5743|5979|6347|7046|7124|7132|7157|7421|7442|23621	ADORA2A|ATM|CASP3|CNR1|CYP1B1|DHODH|NQO1|GRIN2A|HMGCR|IL6|KCNMA1|MMP9|PDGFRB|PIK3CB|PIK3CD|PPARG|PRKDC|PTGS2|RET|CCL2|TGFBR1|TNF|TNFRSF1A|TP53|VDR|TRPV1|BACE1	-14.3716	0	23	0	0	-20.9775	8.824393867
1	-19.8239	GO:0071880	M1	1	0	GO Biological Processes	19	adenylate cyclase-activating adrenergic receptor signaling pathway	-19.8239	76.27238	29.95989	30242	26	183	12	6.557377	1.829835	146|147|148|150|151|152|153|154|1812|1813|1814|5142	ADRA1D|ADRA1B|ADRA1A|ADRA2A|ADRA2B|ADRA2C|ADRB1|ADRB2|DRD1|DRD2|DRD3|PDE4B	-17.5917	0	24	1	1	-19.8239	110.1712204
1	-18.4687	GO:0071875	M1	1	0	GO Biological Processes	19	adrenergic receptor signaling pathway	-18.4687	61.97131	26.92536	30242	32	183	12	6.557377	1.829835	146|147|148|150|151|152|153|154|1812|1813|1814|5142	ADRA1D|ADRA1B|ADRA1A|ADRA2A|ADRA2B|ADRA2C|ADRB1|ADRB2|DRD1|DRD2|DRD3|PDE4B	-16.2898	0	24	0	0	-19.8239	110.1712204
1	-16.5091	GO:0007189	M1	1	0	GO Biological Processes	19	adenylate cyclase-activating G protein-coupled receptor signaling pathway	-16.5091	19.24223	17.23997	30242	146	183	17	9.289617	2.145862	135|146|147|148|150|151|152|153|154|1268|1812|1813|1814|4988|5142|5734|10203	ADORA2A|ADRA1D|ADRA1B|ADRA1A|ADRA2A|ADRA2B|ADRA2C|ADRB1|ADRB2|CNR1|DRD1|DRD2|DRD3|OPRM1|PDE4B|PTGER4|CALCRL	-14.4203	0	24	0	0	-19.8239	110.1712204
1	-15.172	GO:0060047	M1	1	0	GO Biological Processes	19	heart contraction	-15.172	12.97471	14.59313	30242	242	183	19	10.38251	2.254873	100|134|140|146|147|148|153|775|1129|1636|1813|3558|3757|4842|5142|5290|6331|6336|7442	ADA|ADORA1|ADORA3|ADRA1D|ADRA1B|ADRA1A|ADRB1|CACNA1C|CHRM2|AC	-13.1719	0	24	0	0	-19.8239	110.1712204

																			E|DRD2|IL2|KCNH2|NOS1|PDE4B|PIK3CA|SCN5A|SCN10A|TRPV1							
1	-14.8476	GO:0003015	M1	1	0	GO Biological Processes	19	heart process	-14.8476	12.45984	14.25368	30242	252	183	19	10.38251	2.254873	100|134|140|146|147|148|153|775|1129|1636|1813|3558|3757|4842|5142|5290|6331|6336|7442	ADA|ADORA1|ADORA3|ADRA1D|ADRA1B|ADRA1A|ADRB1|CACNA1C|CHRM2|ACE|DRD2|IL2|KCNH2|NOS1|PDE4B|PIK3CA|SCN5A|SCN10A|TRPV1	-12.8691	0	24	0	0	-19.8239	110.1712204
1	-13.9422	GO:0008016	M1	1	0	GO Biological Processes	19	regulation of heart contraction	-13.9422	13.57182	14.16139	30242	207	183	17	9.289617	2.145862	100|134|140|146|147|148|153|775|1129|1813|3558|3757|4842|5142|6331|6336|7442	ADA|ADORA1|ADORA3|ADRA1D|ADRA1B|ADRA1A|ADRB1|CACNA1C|CHRM2|DRD2|IL2|KCNH2|NOS1|PDE4B|SCN5A|SCN10A|TRPV1	-12.0039	0	24	0	0	-19.8239	110.1712204
1	-13.6072	GO:0003073	M1	1	0	GO Biological Processes	19	regulation of systemic arterial blood pressure	-13.6072	22.37853	16.36948	30242	96	183	13	7.103825	1.898976	134|146|147|148|153|154|185|367|1636|1813|2149|4846|7442	ADORA1|ADRA1D|ADRA1B|ADRA1A|ADRB1|ADRB2|AGTR1|AR|ACE|DRD2|F2R|NOS3|TRPV1	-11.6831	0	24	0	0	-19.8239	110.1712204
1	-11.001	GO:0003044	M1	1	0	GO Biological Processes	19	regulation of systemic arterial blood pressure mediated by a chemical signal	-11.001	31.64493	16.405	30242	47	183	9	4.918033	1.598524	146|147|148|153|154|185|1636|2149|4846	ADRA1D|ADRA1B|ADRA1A|ADRB1|ADRB2|AGTR1|ACE|F2R|NOS3	-9.24749	0	24	0	0	-19.8239	110.1712204
1	-10.4411	GO:0002027	M1	1	0	GO Biological Processes	19	regulation of heart rate	-10.4411	17.64879	13.20606	30242	103	183	11	6.010929	1.757049	100|146|147|148|153|775|1813|3757|6331|6336|7442	ADA|ADRA1D|ADRA1B|ADRA1A|ADRB1|CACNA1C|DRD2|KCNH2|SCN5A|SCN10A|TRPV1	-8.72077	0	24	0	0	-19.8239	110.1712204
1	-9.99794	GO:0045777	M1	1	0	GO Biological Processes	19	positive regulation of blood pressure	-9.99794	33.05137	15.82687	30242	40	183	8	4.371585	1.511428	134|146|147|148|151|153|1268|1636	ADORA1|ADRA1D|ADRA1B|ADRA1A|ADRA2B|ADRB1|CNR1|ACE	-8.31116	0	24	0	0	-19.8239	110.1712204
1	-9.02275	GO:0001993	M1	1	0	GO Biological Processes	19	regulation of systemic arterial blood pressure by norepinephrine-epinephrine	-9.02275	91.80935	21.25923	30242	9	183	5	2.73224	1.205087	146|147|148|153|154	ADRA1D|ADRA1B|ADRA1A|ADRB1|ADRB2	-7.4077	0	24	0	0	-19.8239	110.1712204
1	-7.71491	GO:0001996	M1	1	0	GO Biological Processes	19	positive regulation of heart rate by epinephrine-norepinephrine	-7.71491	110.1712	20.8669	30242	6	183	4	2.185792	1.080886	146|147|148|153	ADRA1D|ADRA1B|ADRA1A|ADRB1	-6.20913	0	24	0	0	-19.8239	110.1712204
1	-7.349	GO:0003321	M1	1	0	GO Biological Processes	19	positive regulation of blood pressure by epinephrine-norepinephrine	-7.349	94.43247	19.28982	30242	7	183	4	2.185792	1.080886	146|147|148|153	ADRA1D|ADRA1B|ADRA1A|ADRB1	-5.87776	0	24	0	0	-19.8239	110.1712204
1	-6.17118	GO:0010460	M1	1	0	GO Biological Processes	19	positive regulation of heart rate	-6.17118	29.51015	11.77635	30242	28	183	5	2.73224	1.205087	100|146|147|148|153	ADA|ADRA1D|ADRA1B|ADRA1A|ADRB1	-4.80962	0	24	0	0	-19.8239	110.1712204

1	-5.66547	GO:0150099	M1	1	0	GO Biological Processes	19	neuron-glial cell signaling	-5.66547	99.1541	17.12618	30242	5	183	3	1.639344	0.938686	146|147|148	ADRA1D|ADRA1B|ADRA1A	-4.35145	0	24	0	0	-19.8239	110.1712204
1	-5.42682	GO:0045823	M1	1	0	GO Biological Processes	19	positive regulation of heart contraction	-5.42682	21.18677	9.842615	30242	39	183	5	2.73224	1.205087	100|146|147|148|153	ADA|ADRA1D|ADRA1B|ADRA1A|ADRB1	-4.13771	0	24	0	0	-19.8239	110.1712204
1	-5.3166	GO:1903524	M1	1	0	GO Biological Processes	19	positive regulation of blood circulation	-5.3166	20.15327	9.575481	30242	41	183	5	2.73224	1.205087	100|146|147|148|153	ADA|ADRA1D|ADRA1B|ADRA1A|ADRB1	-4.04593	0	24	0	0	-19.8239	110.1712204
1	-3.29117	GO:0002026	M1	1	0	GO Biological Processes	19	regulation of the force of heart contraction	-3.29117	19.0681	7.191458	30242	26	183	3	1.639344	0.938686	148|153|4842	ADRA1A|ADRB1|NOS1	-2.25835	0	24	0	0	-19.8239	110.1712204
1	-19.3522	GO:0009314	M1	1	0	GO Biological Processes	19	response to radiation	-19.3522	10.12514	15.33682	30242	457	183	28	15.30055	2.661143	351|472|595|596|836|1812|1813|1814|1956|1991|2902|2903|3156|3383|4221|4312|4313|4314|4318|4914|4986|4988|5591|5743|5970|7157|10135|23621	APP|ATM|CCND1|BCL2|CASP3|DRD1|DRD2|DRD3|EGFR|ELANE|GRIN1|GRIN2A|HMGCR|ICAM1|MEN1|MMP1|MMP2|MMP3|MMP9|NTRK1|OPRK1|OPRM1|PRKDC|PTGS2|RELA|TP53|NAMPT|BACE1	-17.1293	0	25	1	1	-19.3522	70.82435597
1	-14.0219	GO:0009416	M1	1	0	GO Biological Processes	19	response to light stimulus	-14.0219	10.29638	13.06515	30242	321	183	20	10.92896	2.306387	351|595|596|836|1812|1813|1814|1956|1991|2902|2903|3156|4221|4312|4313|4314|4318|5743|5970|7157	APP|CCND1|BCL2|CASP3|DRD1|DRD2|DRD3|EGFR|ELANE|GRIN1|GRIN2A|HMGCR|MEN1|MMP1|MMP2|MMP3|MMP9|PTGS2|RELA|TP53	-12.0763	0	25	0	0	-19.3522	70.82435597
1	-11.0735	GO:0009411	M1	1	0	GO Biological Processes	19	response to UV	-11.0735	14.32226	12.76246	30242	150	183	13	7.103825	1.898976	595|596|836|1956|1991|4221|4312|4313|4314|4318|5743|5970|7157	CCND1|BCL2|CASP3|EGFR|ELANE|MEN1|MMP1|MMP2|MMP3|MMP9|PTGS2|RELA|TP53	-9.31525	0	25	0	0	-19.3522	70.82435597
1	-9.88462	GO:0070141	M1	1	0	GO Biological Processes	19	response to UV-A	-9.88462	70.82436	20.38931	30242	14	183	6	3.278689	1.316393	595|1956|4312|4313|4314|4318	CCND1|EGFR|MMP1|MMP2|MMP3|MMP9	-8.21238	0	25	0	0	-19.3522	70.82435597
1	-7.31622	GO:0030198	M1	1	0	GO Biological Processes	19	extracellular matrix organization	-7.31622	7.066904	8.295238	30242	304	183	13	7.103825	1.898976	351|1545|1991|3569|4312|4313|4314|4318|4325|7040|7046|7124|7132	APP|CYP1B1|ELANE|IL6|MMP1|MMP2|MMP3|MMP9|MMP16|TGFB1|TGFBR1|TNF|TNFRSF1A	-5.8494	0	25	0	0	-19.3522	70.82435597
1	-7.29958	GO:0043062	M1	1	0	GO Biological Processes	19	extracellular structure organization	-7.29958	7.043734	8.277276	30242	305	183	13	7.103825	1.898976	351|1545|1991|3569|4312|4313|4314|4318|4325|7040|7046|7124|7132	APP|CYP1B1|ELANE|IL6|MMP1|MMP2|MMP3|MMP9|MMP16|TGFB1|TGFBR1|TNF|TNFRSF1A	-5.83438	0	25	0	0	-19.3522	70.82435597
1	-7.26648	GO:0045229	M1	1	0	GO Biological Processes	19	external encapsulating structure organization	-7.26648	6.997846	8.241594	30242	307	183	13	7.103825	1.898976	351|1545|1991|3569|4312|4313|4314|4318|4325|7040|7046|7124|7132	APP|CYP1B1|ELANE|IL6|MMP1|MMP2|MMP3|MMP9|MMP16|TGFB1|TGFBR1|TNF|TNFRSF1A	-5.80696	0	25	0	0	-19.3522	70.82435597
1	-6.86295	GO:0022617	M1	1	0	GO Biological Processes	19	extracellular matrix disassembly	-6.86295	17.79689	10.57752	30242	65	183	7	3.825137	1.417845	1991|3569|4312|4313|4314|4318|7040	ELANE|IL6|MMP1|MMP2|MMP3|MMP9|TGFB1	-5.43561	0	25	0	0	-19.3522	70.82435597
1	-6.38279	GO:0071492	M1	1	0	GO Biological	19	cellular response to UV-	-6.38279	60.09339	15.29486	30242	11	183	4	2.185792	1.080886	4312|4313|4314|4318	MMP1|MMP2|MMP3|	-5.00371	0	25	0	0	-19.3522	70.82435597

						Processes		A											MMP9							
1	-6.17323	GO:0022411	M1	1	0	GO Biological Processes	19	cellular component disassembly	-6.17323	5.164276	6.928679	30242	448	183	14	7.650273	1.964856	154|1991|3569|3643|4312|4313|4314|4318|5290|7040|7046|7124|7157|59341	ADRB2|ELANE|IL6|INSR|MMP1|MMP2|MMP3|MMP9|PIK3CA|TGFB1|TGFBR1|TNF|TP53|TRPV4	-4.80975	0	25	0	0	-19.3522	70.82435597
1	-5.26368	GO:0030574	M1	1	0	GO Biological Processes	19	collagen catabolic process	-5.26368	19.67343	9.448909	30242	42	183	5	2.73224	1.205087	4312|4313|4314|4318|4325	MMP1|MMP2|MMP3|MMP9|MMP16	-3.99967	0	25	0	0	-19.3522	70.82435597
1	-4.70857	GO:0071478	M1	1	0	GO Biological Processes	19	cellular response to radiation	-4.70857	7.069811	6.496418	30242	187	183	8	4.371585	1.511428	472|4312|4313|4314|4318|5743|7157|10135	ATM|MMP1|MMP2|MMP3|MMP9|PTGS2|TP53|NAMPT	-3.5123	0	25	0	0	-19.3522	70.82435597
1	-4.70593	GO:0034644	M1	1	0	GO Biological Processes	19	cellular response to UV	-4.70593	10.89605	7.376805	30242	91	183	6	3.278689	1.316393	4312|4313|4314|4318|5743|7157	MMP1|MMP2|MMP3|MMP9|PTGS2|TP53	-3.51082	0	25	0	0	-19.3522	70.82435597
1	-3.9514	GO:0071482	M1	1	0	GO Biological Processes	19	cellular response to light stimulus	-3.9514	7.996298	6.091194	30242	124	183	6	3.278689	1.316393	4312|4313|4314|4318|5743|7157	MMP1|MMP2|MMP3|MMP9|PTGS2|TP53	-2.84462	0	25	0	0	-19.3522	70.82435597
1	-19.2153	GO:0062197	M1	1	0	GO Biological Processes	19	cellular response to chemical stress	-19.2153	12.18708	16.16202	30242	339	183	25	13.6612	2.538762	328|472|596|624|836|1545|1728|1956|2936|3066|3569|4313|4314|4318|4363|4846|5290|5743|5970|6326|6513|7099|7157|10280|59341	APEX1|ATM|BCL2|BDKRB2|CASP3|CYP1B1|NQO1|EGFR|GSR|HDAC2|IL6|MMP2|MMP3|MMP9|ABCC1|NOS3|PIK3CA|PTGS2|RELA|SCN2A|SLC2A1|TLR4|TP53|SIGMAR1|TRPV4	-17.0016	0	26	1	1	-19.2153	13.5827532
1	-16.3087	GO:0006979	M1	1	0	GO Biological Processes	19	response to oxidative stress	-16.3087	9.201383	13.66122	30242	449	183	25	13.6612	2.538762	100|328|351|596|761|836|1545|1728|1956|2936|3066|3569|4313|4314|4318|4363|4846|5159|5742|5743|5970|7099|7157|8989|10280	ADA|APEX1|APP|BCL2|CA3|CASP3|CYP1B1|NQO1|EGFR|GSR|HDAC2|IL6|MMP2|MMP3|MMP9|ABCC1|NOS3|PDGFRB|PTGS1|PTGS2|RELA|TLR4|TP53|TRPA1|SIGMAR1	-14.2366	0	26	0	0	-19.2153	13.5827532
1	-12.2384	GO:0000302	M1	1	0	GO Biological Processes	19	response to reactive oxygen species	-12.2384	11.85699	12.69697	30242	223	183	16	8.743169	2.088053	100|328|596|836|1545|1728|1956|3066|3569|4313|4318|4846|5159|5970|8989|10280	ADA|APEX1|BCL2|CASP3|CYP1B1|NQO1|EGFR|HDAC2|IL6|MMP2|MMP9|NOS3|PDGFRB|RELA|TRPA1|SIGMAR1	-10.4022	0	26	0	0	-19.2153	13.5827532
1	-11.5554	GO:0034599	M1	1	0	GO Biological Processes	19	cellular response to oxidative stress	-11.5554	9.687469	11.59887	30242	290	183	17	9.289617	2.145862	328|596|1545|1728|1956|2936|3066|3569|4313|4314|4318|4363|4846|5970|7099|7157|10280	APEX1|BCL2|CYP1B1|NQO1|EGFR|GSR|HDAC2|IL6|MMP2|MMP3|MMP9|ABCC1|NOS3|RELA|TLR4|TP53|SIGMAR1	-9.75563	0	26	0	0	-19.2153	13.5827532
1	-9.99141	GO:0042542	M1	1	0	GO Biological Processes	19	response to hydrogen peroxide	-9.99141	13.58275	11.89143	30242	146	183	12	6.557377	1.829835	100|328|596|836|1545|1728|3066|3569|5159|5970|8989|10280	ADA|APEX1|BCL2|CASP3|CYP1B1|NQO1|HDAC2|IL6|PDGFRB|RELA|TRPA1|SIGMAR1	-8.30597	0	26	0	0	-19.2153	13.5827532
1	-8.52411	GO:0034614	M1	1	0	GO Biological Processes	19	cellular response to reactive oxygen species	-8.52411	11.7279	10.44787	30242	155	183	11	6.010929	1.757049	328|1545|1728|1956|3066|3569|4313|4318|484	APEX1|CYP1B1|NQO1|EGFR|HDAC2|IL6|M	-6.93985	0	26	0	0	-19.2153	13.5827532

																		6|5970|10280	MP2|MMP9|NOS3|RELA|SIGMAR1							
1	-5.63761	GO:0070301	M1	1	0	GO Biological Processes	19	cellular response to hydrogen peroxide	-5.63761	11.80406	8.358646	30242	98	183	7	3.825137	1.417845	328|1545|1728|3066|3569|5970|10280	APEX1|CYP1B1|NQO1|HDAC2|IL6|RELA|SIGMAR1	-4.32642	0	26	0	0	-19.2153	13.5827532
1	-18.7826	GO:0018105	M1	1	0	GO Biological Processes	19	peptidyl-serine phosphorylation	-18.7826	12.591	16.13544	30242	315	183	24	13.11475	2.495327	351|472|596|624|673|1453|1956|3551|3569|4842|4915|4985|5290|5582|5591|5743|5979|6197|7040|7046|7124|8986|9475|29110	APP|ATM|BCL2|BDKRB2|BRAF|CSNK1D|EGFR|IKBKB|IL6|NOS1|NTRK2|OPRD1|PIK3CA|PRKCG|PRKDC|PTGS2|RET|RPS6KA3|TGFB1|TGFBR1|TNF|RPS6KA4|ROCK2|TBK1	-16.591	0	27	1	1	-18.7826	21.42218174
1	-18.0718	GO:0018209	M1	1	0	GO Biological Processes	19	peptidyl-serine modification	-18.0718	11.73421	15.48461	30242	338	183	24	13.11475	2.495327	351|472|596|624|673|1453|1956|3551|3569|4842|4915|4985|5290|5582|5591|5743|5979|6197|7040|7046|7124|8986|9475|29110	APP|ATM|BCL2|BDKRB2|BRAF|CSNK1D|EGFR|IKBKB|IL6|NOS1|NTRK2|OPRD1|PIK3CA|PRKCG|PRKDC|PTGS2|RET|RPS6KA3|TGFB1|TGFBR1|TNF|RPS6KA4|ROCK2|TBK1	-15.9251	0	27	0	0	-18.7826	21.42218174
1	-14.3427	GO:0033138	M1	1	0	GO Biological Processes	19	positive regulation of peptidyl-serine phosphorylation	-14.3427	21.42218	16.58907	30242	108	183	14	7.650273	1.964856	351|596|673|1956|3569|4842|4915|4985|5290|5743|5979|7040|7124|29110	APP|BCL2|BRAF|EGFR|IL6|NOS1|NTRK2|OPRD1|PIK3CA|PTGS2|RET|TGFB1|TNF|TBK1	-12.3823	0	27	0	0	-18.7826	21.42218174
1	-13.8887	GO:0033135	M1	1	0	GO Biological Processes	19	regulation of peptidyl-serine phosphorylation	-13.8887	17.21425	15.21758	30242	144	183	15	8.196721	2.027793	351|596|624|673|1956|3569|4842|4915|4985|5290|5743|5979|7040|7124|29110	APP|BCL2|BDKRB2|BRAF|EGFR|IL6|NOS1|NTRK2|OPRD1|PIK3CA|PTGS2|RET|TGFB1|TNF|TBK1	-11.9576	0	27	0	0	-18.7826	21.42218174
1	-5.14962	GO:0018107	M1	1	0	GO Biological Processes	19	peptidyl-threonine phosphorylation	-5.14962	9.972395	7.554453	30242	116	183	7	3.825137	1.417845	351|596|5591|7040|7046|9475|29110	APP|BCL2|PRKDC|TGFB1|TGFBR1|ROCK2|TBK1	-3.8996	0	27	0	0	-18.7826	21.42218174
1	-4.93641	GO:0018210	M1	1	0	GO Biological Processes	19	peptidyl-threonine modification	-4.93641	9.254383	7.215561	30242	125	183	7	3.825137	1.417845	351|596|5591|7040|7046|9475|29110	APP|BCL2|PRKDC|TGFB1|TGFBR1|ROCK2|TBK1	-3.70798	0	27	0	0	-18.7826	21.42218174
1	-18.2529	GO:1904645	M1	1	0	GO Biological Processes	19	response to amyloid-beta	-18.2529	39.21349	22.92439	30242	59	183	14	7.650273	1.964856	154|351|2902|2903|2915|3383|4313|4314|4318|4363|4914|7099|10135|23621	ADRB2|APP|GRIN1|GRIN2A|GRM5|ICAM1|MMP2|MMP3|MMP9|ABCC1|NTRK1|TLR4|NAMPT|BACE1	-16.0943	0	28	1	1	-18.2529	39.21348523
1	-14.2621	GO:1904646	M1	1	0	GO Biological Processes	19	cellular response to amyloid-beta	-14.2621	37.87136	19.94738	30242	48	183	11	6.010929	1.757049	154|351|2902|2903|2915|3383|4363|4914|7099|10135|23621	ADRB2|APP|GRIN1|GRIN2A|GRM5|ICAM1|ABCC1|NTRK1|TLR4|NAMPT|BACE1	-12.3041	0	28	0	0	-18.2529	39.21348523
1	-17.6891	GO:0019932	M1	1	0	GO Biological Processes	19	second-messenger-mediated signaling	-17.6891	12.18239	15.49156	30242	312	183	23	12.56831	2.450458	100|185|775|1131|1230|1910|1956|2902|2903|2904|2915|3357|3358|35	ADA|AGTR1|CACNA1C|CHRM3|CCR1|EDNRB|EGFR|GRIN1|GR	-15.5576	0	29	1	1	-17.6891	14.72585619

																		76|3577|4842|4843|4846|5024|5138|6401|7124|729230	IN2A|GRIN2B|GRM5|HTR2B|HTR2C|CXCL8|CXCR1|NOS1|NOS2|NOS3|P2RX3|PDE2A|SELE|TNF|CCR2							
1	-15.3638	GO:0019722	M1	1	0	GO Biological Processes	19	calcium-mediated signaling	-15.3638	14.72586	15.2722	30242	202	183	18	9.836066	2.201413	100|185|775|1131|1230|1910|1956|2902|2903|2904|2915|3357|3576|3577|5024|6401|7124|729230	ADA|AGTR1|CACNA1C|CHRM3|CCR1|EDNRB|EGFR|GRIN1|GRIN2A|GRIN2B|GRM5|HTR2B|CXCL8|CXCR1|P2RX3|SELE|TNF|CCR2	-13.3554	0	29	0	0	-17.6891	14.72585619
1	-17.141	GO:0050900	M1	1	0	GO Biological Processes	19	leukocyte migration	-17.141	10.69047	14.65356	30242	371	183	24	13.11475	2.495327	100|134|351|1230|1910|1991|2155|3383|3569|3576|3577|3683|4318|5142|5293|5734|5979|6347|6401|6793|7124|59341|114548|729230	ADA|ADORA1|APP|CCR1|EDNRB|ELANE|F7|ICAM1|IL6|CXCL8|CXCR1|ITGAL|MMP9|PDE4B|PIK3CD|PTGER4|RET|CCL2|SELE|STK10|TNF|TRPV4|NLRP3|CCR2	-15.0278	0	30	1	1	-17.141	19.06809584
1	-12.6094	GO:0002685	M1	1	0	GO Biological Processes	19	regulation of leukocyte migration	-12.6094	12.53132	13.11511	30242	211	183	16	8.743169	2.088053	100|134|351|1230|1991|2155|3383|3569|3576|5734|6347|6401|6793|7124|59341|729230	ADA|ADORA1|APP|CCR1|ELANE|F7|ICAM1|IL6|CXCL8|PTGER4|CCL2|SELE|STK10|TNF|TRPV4|CCR2	-10.7499	0	30	0	0	-17.141	19.06809584
1	-10.6749	GO:0006935	M1	1	0	GO Biological Processes	19	chemotaxis	-10.6749	5.999423	9.700246	30242	606	183	22	12.02186	2.404073	185|351|1230|1910|1991|2050|2155|3569|3576|3577|4363|4914|5142|5159|5291|5293|5979|6347|7040|10800|59341|729230	AGTR1|APP|CCR1|EDNRB|ELANE|EPHB4|F7|IL6|CXCL8|CXCR1|ABCC1|NTRK1|PDE4B|PDGFRB|PIK3CB|PIK3CD|RET|CCL2|TGFB1|CYSLTR1|TRPV4|CCR2	-8.94136	0	30	0	0	-17.141	19.06809584
1	-10.6337	GO:0042330	M1	1	0	GO Biological Processes	19	taxis	-10.6337	5.969869	9.667232	30242	609	183	22	12.02186	2.404073	185|351|1230|1910|1991|2050|2155|3569|3576|3577|4363|4914|5142|5159|5291|5293|5979|6347|7040|10800|59341|729230	AGTR1|APP|CCR1|EDNRB|ELANE|EPHB4|F7|IL6|CXCL8|CXCR1|ABCC1|NTRK1|PDE4B|PDGFRB|PIK3CB|PIK3CD|RET|CCL2|TGFB1|CYSLTR1|TRPV4|CCR2	-8.90307	0	30	0	0	-17.141	19.06809584
1	-9.58485	GO:0071674	M1	1	0	GO Biological Processes	19	mononuclear cell migration	-9.58485	10.90527	10.88303	30242	197	183	13	7.103825	1.898976	351|1230|3383|3569|3577|3683|5293|5979|6347|6793|7124|59341|729230	APP|CCR1|ICAM1|IL6|CXCR1|ITGAL|PIK3CD|RET|CCL2|STK10|TNF|TRPV4|CCR2	-7.93417	0	30	0	0	-17.141	19.06809584
1	-8.12054	GO:0060326	M1	1	0	GO Biological Processes	19	cell chemotaxis	-8.12054	7.439214	8.906125	30242	311	183	14	7.650273	1.964856	185|1230|1910|2155|3569|3576|3577|4363|5142|5159|5293|6347|59341|729230	AGTR1|CCR1|EDNRB|F7|IL6|CXCL8|CXCR1|ABCC1|PDE4B|PDGFRB|PIK3CD|CCL2|TRPV4|CCR2	-6.57486	0	30	0	0	-17.141	19.06809584
1	-6.91142	GO:0097529	M1	1	0	GO Biological	19	myeloid leukocyte	-6.91142	8.188402	8.387641	30242	222	183	11	6.010929	1.757049	1230|1910|3569|3576|3	CCR1|EDNRB|IL6|CX	-5.48185	0	30	0	0	-17.141	19.06809584

						Processes		migration										577|5142|5293|5734|6347|59341|729230	CL8|CXCR1|PDE4B|PIK3CD|PTGER4|CCL2|TRPV4|CCR2							
1	-6.83853	GO:0002687	M1	1	0	GO Biological Processes	19	positive regulation of leukocyte migration	-6.83853	11.01712	9.101489	30242	135	183	9	4.918033	1.598524	351|1230|2155|3383|3569|3576|7124|59341|729230	APP|CCR1|F7|ICAM1|IL6|CXCL8|TNF|TRPV4|CCR2	-5.41487	0	30	0	0	-17.141	19.06809584
1	-6.73764	GO:0030595	M1	1	0	GO Biological Processes	19	leukocyte chemotaxis	-6.73764	7.869373	8.177475	30242	231	183	11	6.010929	1.757049	1230|1910|2155|3569|3576|3577|5142|5293|6347|59341|729230	CCR1|EDNRB|F7|IL6|CXCL8|CXCR1|PDE4B|PIK3CD|CCL2|TRPV4|CCR2	-5.32627	0	30	0	0	-17.141	19.06809584
1	-5.93692	GO:0050920	M1	1	0	GO Biological Processes	19	regulation of chemotaxis	-5.93692	7.41062	7.497055	30242	223	183	10	5.464481	1.680144	1230|1991|2155|3569|3576|5159|6347|7040|59341|729230	CCR1|ELANE|F7|IL6|CXCL8|PDGFRB|CCL2|TGFB1|TRPV4|CCR2	-4.59403	0	30	0	0	-17.141	19.06809584
1	-5.60904	GO:0050921	M1	1	0	GO Biological Processes	19	positive regulation of chemotaxis	-5.60904	9.376274	7.778688	30242	141	183	8	4.371585	1.511428	1230|2155|3569|3576|5159|7040|59341|729230	CCR1|F7|IL6|CXCL8|PDGFRB|TGFB1|TRPV4|CCR2	-4.29955	0	30	0	0	-17.141	19.06809584
1	-5.00552	GO:0002688	M1	1	0	GO Biological Processes	19	regulation of leukocyte chemotaxis	-5.00552	9.481949	7.324609	30242	122	183	7	3.825137	1.417845	1230|2155|3569|3576|6347|59341|729230	CCR1|F7|IL6|CXCL8|CCL2|TRPV4|CCR2	-3.76905	0	30	0	0	-17.141	19.06809584
1	-4.62554	GO:0002690	M1	1	0	GO Biological Processes	19	positive regulation of leukocyte chemotaxis	-4.62554	10.54831	7.234316	30242	94	183	6	3.278689	1.316393	1230|2155|3569|3576|59341|729230	CCR1|F7|IL6|CXCL8|TRPV4|CCR2	-3.43978	0	30	0	0	-17.141	19.06809584
1	-4.44317	GO:0097530	M1	1	0	GO Biological Processes	19	granulocyte migration	-4.44317	7.763744	6.45779	30242	149	183	7	3.825137	1.417845	3576|3577|5142|5293|5734|6347|59341	CXCL8|CXCR1|PDE4B|PIK3CD|PTGER4|CCL2|TRPV4	-3.28976	0	30	0	0	-17.141	19.06809584
1	-3.9322	GO:0071621	M1	1	0	GO Biological Processes	19	granulocyte chemotaxis	-3.9322	7.932328	6.059888	30242	125	183	6	3.278689	1.316393	3576|3577|5142|5293|6347|59341	CXCL8|CXCR1|PDE4B|PIK3CD|CCL2|TRPV4	-2.82961	0	30	0	0	-17.141	19.06809584
1	-3.69798	GO:0070098	M1	1	0	GO Biological Processes	19	chemokine-mediated signaling pathway	-3.69798	9.389593	6.149589	30242	88	183	5	2.73224	1.205087	1230|3576|3577|6347|729230	CCR1|CXCL8|CXCR1|CCL2|CCR2	-2.62334	0	30	0	0	-17.141	19.06809584
1	-3.50082	GO:1990868	M1	1	0	GO Biological Processes	19	response to chemokine	-3.50082	8.518393	5.786815	30242	97	183	5	2.73224	1.205087	1230|3576|3577|6347|729230	CCR1|CXCL8|CXCR1|CCL2|CCR2	-2.44723	0	30	0	0	-17.141	19.06809584
1	-3.50082	GO:1990869	M1	1	0	GO Biological Processes	19	cellular response to chemokine	-3.50082	8.518393	5.786815	30242	97	183	5	2.73224	1.205087	1230|3576|3577|6347|729230	CCR1|CXCL8|CXCR1|CCL2|CCR2	-2.44723	0	30	0	0	-17.141	19.06809584
1	-3.38048	GO:0030593	M1	1	0	GO Biological Processes	19	neutrophil chemotaxis	-3.38048	8.022176	5.570091	30242	103	183	5	2.73224	1.205087	3576|3577|5142|5293|6347	CXCL8|CXCR1|PDE4B|PIK3CD|CCL2	-2.338	0	30	0	0	-17.141	19.06809584
1	-3.29117	GO:0002407	M1	1	0	GO Biological Processes	19	dendritic cell chemotaxis	-3.29117	19.0681	7.191458	30242	26	183	3	1.639344	0.938686	1230|3577|729230	CCR1|CXCR1|CCR2	-2.25835	0	30	0	0	-17.141	19.06809584
1	-3.0367	GO:0002548	M1	1	0	GO Biological Processes	19	monocyte chemotaxis	-3.0367	9.310244	5.469964	30242	71	183	4	2.185792	1.080886	1230|3569|6347|729230	CCR1|IL6|CCL2|CCR2	-2.02937	0	30	0	0	-17.141	19.06809584
1	-3.03025	GO:1990266	M1	1	0	GO Biological Processes	19	neutrophil migration	-3.03025	6.717757	4.957861	30242	123	183	5	2.73224	1.205087	3576|3577|5142|5293|6347	CXCL8|CXCR1|PDE4B|PIK3CD|CCL2	-2.02376	0	30	0	0	-17.141	19.06809584
1	-2.98278	GO:0036336	M1	1	0	GO Biological Processes	19	dendritic cell migration	-2.98278	15.02335	6.288932	30242	33	183	3	1.639344	0.938686	1230|3577|729230	CCR1|CXCR1|CCR2	-1.98159	0	30	0	0	-17.141	19.06809584
1	-16.4924	GO:0072593	M1	1	0	GO Biological Processes	19	reactive oxygen species metabolic process	-16.4924	13.82902	15.53626	30242	239	183	20	10.92896	2.306387	185|596|1543|1544|1545|1645|1728|1956|2147|2902|4129|4314|4843|4846|5159|5465|7040|7099|7124|7157	AGTR1|BCL2|CYP1A1|CYP1A2|CYP1B1|AKR1C1|NQO1|EGFR|F2|GRIN1|MAOB|MMP3|NOS2|NOS3|PDGFRB|PPARA|TGFB1|TLR4|TNF|TP53	-14.407	0	31	1	1	-16.4924	19.56988783
1	-12.0566	GO:2000377	M1	1	0	GO Biological	19	regulation of reactive	-12.0566	14.73628	13.46419	30242	157	183	14	7.650273	1.964856	185|596|1545|1645|195	AGTR1|BCL2|CYP1B	-10.2316	0	31	0	0	-16.4924	19.56988783

						Processes		oxygen species metabolic process										6|2147|2902|4314|5159|5465|7040|7099|7124|7157	1|AKR1C1|EGFR|F2|GRIN1|MMP3|PDGFRB|PPARA|TGFB1|TLR4|TNF|TP53							
1	-9.04699	GO:2000379	M1	1	0	GO Biological Processes	19	positive regulation of reactive oxygen species metabolic process	-9.04699	19.56989	12.64718	30242	76	183	9	4.918033	1.598524	185|1645|1956|2147|2902|5159|7040|7099|7157	AGTR1|AKR1C1|EGFR|F2|GRIN1|PDGFRB|TGFB1|TLR4|TP53	-7.4285	0	31	0	0	-16.4924	19.56988783
1	-5.59621	GO:1903409	M1	1	0	GO Biological Processes	19	reactive oxygen species biosynthetic process	-5.59621	15.49283	9.055955	30242	64	183	6	3.278689	1.316393	1543|1544|2902|4129|5465|7099	CYP1A1|CYP1A2|GRIN1|MAOB|PPARA|TLR4	-4.28953	0	31	0	0	-16.4924	19.56988783
1	-2.41178	GO:1903426	M1	1	0	GO Biological Processes	19	regulation of reactive oxygen species biosynthetic process	-2.41178	9.534048	4.805754	30242	52	183	3	1.639344	0.938686	2902|5465|7099	GRIN1|PPARA|TLR4	-1.4684	0	31	0	0	-16.4924	19.56988783
1	-16.291	GO:0070997	M1	1	0	GO Biological Processes	19	neuron death	-16.291	10.52883	14.21124	30242	361	183	23	12.56831	2.450458	134|135|351|596|836|1728|2149|2558|2904|4914|4915|5290|5465|5582|6326|6347|7099|7124|7157|10135|10280|23621|29110	ADORA1|ADORA2A|APP|BCL2|CASP3|NQO1|F2R|GABRA5|GRIN2B|NTRK1|NTRK2|PIK3CA|PPARA|PRKCG|SCN2A|CCL2|TLR4|TNF|TP53|NAMPT|SIGMAR1|BACE1|TBK1	-14.2221	0	32	1	1	-16.291	11.69270028
1	-14.0734	GO:1901214	M1	1	0	GO Biological Processes	19	regulation of neuron death	-14.0734	10.36093	13.11439	30242	319	183	20	10.92896	2.306387	134|135|596|836|1728|2149|2558|2904|4914|4915|5290|5465|5582|6347|7099|7124|7157|10280|23621|29110	ADORA1|ADORA2A|BCL2|CASP3|NQO1|F2R|GABRA5|GRIN2B|NTRK1|NTRK2|PIK3CA|PPARA|PRKCG|CCL2|TLR4|TNF|TP53|SIGMAR1|BACE1|TBK1	-12.1229	0	32	0	0	-16.291	11.69270028
1	-12.7086	GO:0051402	M1	1	0	GO Biological Processes	19	neuron apoptotic process	-12.7086	11.42019	12.80406	30242	246	183	17	9.289617	2.145862	135|351|596|836|1728|2149|2558|4914|4915|5290|5582|6326|6347|7124|7157|10280|23621	ADORA2A|APP|BCL2|CASP3|NQO1|F2R|GABRA5|NTRK1|NTRK2|PIK3CA|PRKCG|SCN2A|CCL2|TNF|TP53|SIGMAR1|BACE1	-10.8389	0	32	0	0	-16.291	11.69270028
1	-11.416	GO:0043523	M1	1	0	GO Biological Processes	19	regulation of neuron apoptotic process	-11.416	11.6927	12.19029	30242	212	183	15	8.196721	2.027793	135|596|836|1728|2149|2558|4914|4915|5290|5582|6347|7124|7157|10280|23621	ADORA2A|BCL2|CASP3|NQO1|F2R|GABRA5|NTRK1|NTRK2|PIK3CA|PRKCG|CCL2|TNF|TP53|SIGMAR1|BACE1	-9.62651	0	32	0	0	-16.291	11.69270028
1	-6.57337	GO:0043524	M1	1	0	GO Biological Processes	19	negative regulation of neuron apoptotic process	-6.57337	10.25732	8.718546	30242	145	183	9	4.918033	1.598524	135|596|2149|2558|4914|4915|5290|5582|6347	ADORA2A|BCL2|F2R|GABRA5|NTRK1|NTRK2|PIK3CA|PRKCG|CCL2	-5.17811	0	32	0	0	-16.291	11.69270028
1	-6.212	GO:1901215	M1	1	0	GO Biological Processes	19	negative regulation of neuron death	-6.212	7.94504	7.842169	30242	208	183	10	5.464481	1.680144	135|596|2149|2558|4914|4915|5290|5465|5582|6347	ADORA2A|BCL2|F2R|GABRA5|NTRK1|NTRK2|PIK3CA|PPARA|PRKCG|CCL2	-4.84403	0	32	0	0	-16.291	11.69270028
1	-4.68927	GO:0006909	M1	1	0	GO Biological Processes	19	phagocytosis	-4.68927	5.348117	5.994345	30242	309	183	10	5.464481	1.680144	134|135|1991|3683|5290|5582|6347|7099|7124|	ADORA1|ADORA2A|ELANE|ITGAL|PIK3C	-3.49631	0	32	0	0	-16.291	11.69270028

																		729230	A|PRKCG|CCL2|TLR4 |TNF|CCR2							
1	-16.2584	GO:0048660	M1	1	0	GO Biological Processes	19	regulation of smooth muscle cell proliferation	-16.2584	16.52568	16.30101	30242	180	183	18	9.836066	2.201413	1956|1991|2263|3156|3351|3569|4313|4318|5159|5467|5468|5591|5743|7040|7099|7124|10135|10203	EGFR|ELANE|FGFR2|HMGCR|HTR1B|IL6|MMP2|MMP9|PDGFRB|PPARD|PPARG|PRKDC|PTGS2|TGFB1|TLR4|TNF|NAMPT|CALCRL	-14.1928	0	33	1	1	-16.2584	23.8351198
1	-16.087	GO:0048659	M1	1	0	GO Biological Processes	19	smooth muscle cell proliferation	-16.087	16.16643	16.10084	30242	184	183	18	9.836066	2.201413	1956|1991|2263|3156|3351|3569|4313|4318|5159|5467|5468|5591|5743|7040|7099|7124|10135|10203	EGFR|ELANE|FGFR2|HMGCR|HTR1B|IL6|MMP2|MMP9|PDGFRB|PPARD|PPARG|PRKDC|PTGS2|TGFB1|TLR4|TNF|NAMPT|CALCRL	-14.0372	0	33	0	0	-16.2584	23.8351198
1	-16.0503	GO:0048661	M1	1	0	GO Biological Processes	19	positive regulation of smooth muscle cell proliferation	-16.0503	23.83512	18.20116	30242	104	183	15	8.196721	2.027793	1956|1991|2263|3156|3351|3569|4313|4318|5159|5743|7040|7099|7124|10135|10203	EGFR|ELANE|FGFR2|HMGCR|HTR1B|IL6|MMP2|MMP9|PDGFRB|PTGS2|TGFB1|TLR4|TNF|NAMPT|CALCRL	-14.0066	0	33	0	0	-16.2584	23.8351198
1	-14.9756	GO:0033002	M1	1	0	GO Biological Processes	19	muscle cell proliferation	-14.9756	12.66081	14.38711	30242	248	183	19	10.38251	2.254873	1956|1991|2263|3156|3351|3569|4313|4318|5159|5467|5468|5591|5743|7040|7046|7099|7124|10135|10203	EGFR|ELANE|FGFR2|HMGCR|HTR1B|IL6|MMP2|MMP9|PDGFRB|PPARD|PPARG|PRKDC|PTGS2|TGFB1|TGFBR1|TLR4|TNF|NAMPT|CALCRL	-12.9891	0	33	0	0	-16.2584	23.8351198
1	-8.33599	GO:0001503	M1	1	0	GO Biological Processes	19	ossification	-8.33599	6.464815	8.682104	30242	409	183	16	8.743169	2.088053	154|249|596|1230|1956|2263|3569|4221|4313|4318|4325|5468|5734|5743|7040|7124	ADRB2|ALPL|BCL2|CCR1|EGFR|FGFR2|IL6|MEN1|MMP2|MMP9|MMP16|PPARG|PTGER4|PTGS2|TGFB1|TNF	-6.77145	0	33	0	0	-16.2584	23.8351198
1	-16.0543	GO:0070848	M1	1	0	GO Biological Processes	19	response to growth factor	-16.0543	6.866627	12.44902	30242	722	183	30	16.39344	2.736716	351|836|890|1453|1956|2263|2908|3066|3576|4221|4842|4914|4915|4985|4988|5138|5159|5290|5291|5293|5465|5468|5970|6347|7040|7046|7099|7157|7442|9475	APP|CASP3|CCNA2|CSNK1D|EGFR|FGFR2|NR3C1|HDAC2|CXCL8|MEN1|NOS1|NTRK1|NTRK2|OPRD1|OPRM1|PDE2A|PDGFRB|PIK3CA|PIK3CB|PIK3CD|PPARA|PPARG|RELA|CCL2|TGFB1|TGFBR1|TLR4|TP53|TRPV1|ROCK2	-14.0076	0	34	1	1	-16.0543	6.86662731
1	-14.7103	GO:0071363	M1	1	0	GO Biological Processes	19	cellular response to growth factor stimulus	-14.7103	6.696369	11.81918	30242	691	183	28	15.30055	2.661143	351|836|890|1453|1956|2263|2908|3066|3576|4221|4842|4914|4915|4985|5138|5159|5290|5291|5293|5465|5468|5970|	APP|CASP3|CCNA2|CSNK1D|EGFR|FGFR2|NR3C1|HDAC2|CXCL8|MEN1|NOS1|NTRK1|NTRK2|OPRD1|PDE2	-12.7406	0	34	0	0	-16.0543	6.86662731

																		6347|7040|7046|7099|7157|7442	A|PDGFRB|PIK3CA|PIK3CB|PIK3CD|PPARA|PPARG|RELA|CCL2|TGFB1|TGFBR1|TLR4|TP53|TRPV1							
1	-15.7618	GO:0048545	M1	1	0	GO Biological Processes	19	response to steroid hormone	-15.7618	10.72463	14.04921	30242	339	183	22	12.02186	2.404073	249|367|595|596|760|836|1956|2099|2100|2908|3351|3383|3569|4129|5241|5465|5467|5743|5970|7040|7124|7298	ALPL|AR|CCND1|BCL2|CA2|CASP3|EGFR|ESR1|ESR2|NR3C1|HTR1B|ICAM1|IL6|MAOB|PGR|PPARA|PPARD|PTGS2|RELA|TGFB1|TNF|TYMS	-13.7448	0	35	1	1	-15.7618	18.88649493
1	-12.4974	GO:0048732	M1	1	0	GO Biological Processes	19	gland development	-12.4974	7.941404	11.40459	30242	437	183	21	11.47541	2.356083	100|367|595|596|1543|1723|1813|1956|2099|2263|3569|5241|5290|5591|5970|6531|7040|7046|7124|7298|7421	ADA|AR|CCND1|BCL2|CYP1A1|DHODH|DRD2|EGFR|ESR1|FGFR2|IL6|PGR|PIK3CA|PRKDC|RELA|SLC6A3|TGFB1|TGFBR1|TNF|TYMS|VDR	-10.6459	0	35	0	0	-15.7618	18.88649493
1	-10.4817	GO:0031960	M1	1	0	GO Biological Processes	19	response to corticosteroid	-10.4817	12.8643	11.99593	30242	167	183	13	7.103825	1.898976	249|595|596|836|1956|2908|3351|3383|3569|4129|5743|7124|7298	ALPL|CCND1|BCL2|CASP3|EGFR|NR3C1|HTR1B|ICAM1|IL6|MAOB|PTGS2|TNF|TYMS	-8.75998	0	35	0	0	-15.7618	18.88649493
1	-9.92225	GO:0051384	M1	1	0	GO Biological Processes	19	response to glucocorticoid	-9.92225	13.3992	11.79834	30242	148	183	12	6.557377	1.829835	249|595|596|836|1956|2908|3383|3569|4129|5743|7124|7298	ALPL|CCND1|BCL2|CASP3|EGFR|NR3C1|ICAM1|IL6|MAOB|PTGS2|TNF|TYMS	-8.2461	0	35	0	0	-15.7618	18.88649493
1	-5.47398	GO:0001889	M1	1	0	GO Biological Processes	19	liver development	-5.47398	8.993569	7.580338	30242	147	183	8	4.371585	1.511428	100|595|1543|1956|3569|5290|5970|7298	ADA|CCND1|CYP1A1|EGFR|IL6|PIK3CA|RELA|TYMS	-4.17836	0	35	0	0	-15.7618	18.88649493
1	-5.40876	GO:0061008	M1	1	0	GO Biological Processes	19	hepaticobiliary system development	-5.40876	8.813698	7.485366	30242	150	183	8	4.371585	1.511428	100|595|1543|1956|3569|5290|5970|7298	ADA|CCND1|CYP1A1|EGFR|IL6|PIK3CA|RELA|TYMS	-4.12072	0	35	0	0	-15.7618	18.88649493
1	-5.15752	GO:0031100	M1	1	0	GO Biological Processes	19	animal organ regeneration	-5.15752	13.04659	8.204431	30242	76	183	6	3.278689	1.316393	595|890|1956|2155|3569|7298	CCND1|CCNA2|EGFR|F7|IL6|TYMS	-3.90602	0	35	0	0	-15.7618	18.88649493
1	-4.53073	GO:0031099	M1	1	0	GO Biological Processes	19	regeneration	-4.53073	6.677044	6.253344	30242	198	183	8	4.371585	1.511428	595|596|890|1956|2155|3569|5467|7298	CCND1|BCL2|CCNA2|EGFR|F7|IL6|PPARD|TYMS	-3.3625	0	35	0	0	-15.7618	18.88649493
1	-4.23163	GO:0097421	M1	1	0	GO Biological Processes	19	liver regeneration	-4.23163	18.88649	8.261175	30242	35	183	4	2.185792	1.080886	595|1956|3569|7298	CCND1|EGFR|IL6|TYMS	-3.09596	0	35	0	0	-15.7618	18.88649493
1	-15.3079	GO:0050795	M1	1	0	GO Biological Processes	19	regulation of behavior	-15.3079	29.83804	19.11528	30242	72	183	13	7.103825	1.898976	100|134|135|153|1268|1813|1814|3062|3066|3350|3351|3357|4986	ADA|ADORA1|ADORA2A|ADRB1|CNR1|DRD2|DRD3|HCRTR2|HDAC2|HTR1A|HTR1B|HTR2B|OPRK1	-13.305	0	36	1	1	-15.3079	55.0856102
1	-12.9418	GO:0007588	M1	1	0	GO Biological Processes	19	excretion	-12.9418	29.31976	17.41655	30242	62	183	11	6.010929	1.757049	134|135|136|148|185|1813|1814|1910|3778|7442|9429	ADORA1|ADORA2A|ADORA2B|ADRA1A|AGTR1|DRD2|DRD3|EDNRB|KCNMA1|TRPV1|ABCG2	-11.0554	0	36	0	0	-15.3079	55.0856102

1	-10.844	GO:0030431	M1	1	0	GO Biological Processes	19	sleep	-10.844	41.31421	17.80304	30242	32	183	8	4.371585	1.511428	100|134|135|153|1813|1814|2903|3356	ADA|ADORA1|ADORA2A|ADRB1|DRD2|DRD3|GRIN2A|HTR2A	-9.09985	0	36	0	0	-15.3079	55.0856102
1	-10.3787	GO:0042749	M1	1	0	GO Biological Processes	19	regulation of circadian sleep/wake cycle	-10.3787	52.58172	18.8841	30242	22	183	7	3.825137	1.417845	100|134|135|153|1813|1814|3062	ADA|ADORA1|ADORA2A|ADRB1|DRD2|DRD3|HCRTR2	-8.67264	0	36	0	0	-15.3079	55.0856102
1	-10.127	GO:0003014	M1	1	0	GO Biological Processes	19	renal system process	-10.127	16.52568	12.72827	30242	110	183	11	6.010929	1.757049	134|135|148|185|596|1813|1814|1910|2149|3778|7442	ADORA1|ADORA2A|ADRA1A|AGTR1|BCL2|DRD2|DRD3|EDNRB|F2R|KCNMA1|TRPV1	-8.42937	0	36	0	0	-15.3079	55.0856102
1	-10.0757	GO:0022410	M1	1	0	GO Biological Processes	19	circadian sleep/wake cycle process	-10.0757	48.19991	18.04888	30242	24	183	7	3.825137	1.417845	100|134|135|153|1813|1814|3062	ADA|ADORA1|ADORA2A|ADRB1|DRD2|DRD3|HCRTR2	-8.38354	0	36	0	0	-15.3079	55.0856102
1	-9.67316	GO:0042745	M1	1	0	GO Biological Processes	19	circadian sleep/wake cycle	-9.67316	42.84436	16.97243	30242	27	183	7	3.825137	1.417845	100|134|135|153|1813|1814|3062	ADA|ADORA1|ADORA2A|ADRB1|DRD2|DRD3|HCRTR2	-8.01625	0	36	0	0	-15.3079	55.0856102
1	-9.402	GO:0048512	M1	1	0	GO Biological Processes	19	circadian behavior	-9.402	28.12882	14.52274	30242	47	183	8	4.371585	1.511428	100|134|135|153|1813|1814|3062|7157	ADA|ADORA1|ADORA2A|ADRB1|DRD2|DRD3|HCRTR2|TP53	-7.76201	0	36	0	0	-15.3079	55.0856102
1	-9.24988	GO:0007622	M1	1	0	GO Biological Processes	19	rhythmic behavior	-9.24988	26.98071	14.20143	30242	49	183	8	4.371585	1.511428	100|134|135|153|1813|1814|3062|7157	ADA|ADORA1|ADORA2A|ADRB1|DRD2|DRD3|HCRTR2|TP53	-7.61856	0	36	0	0	-15.3079	55.0856102
1	-9.10453	GO:0051339	M1	1	0	GO Biological Processes	19	regulation of lyase activity	-9.10453	25.92264	13.89878	30242	51	183	8	4.371585	1.511428	775|1813|1814|1910|4842|4843|4846|729230	CACNA1C|DRD2|DRD3|EDNRB|NOS1|NOS2|NOS3|CCR2	-7.47792	0	36	0	0	-15.3079	55.0856102
1	-9.10223	GO:0045187	M1	1	0	GO Biological Processes	19	regulation of circadian sleep/wake cycle, sleep	-9.10223	55.08561	17.90929	30242	18	183	6	3.278689	1.316393	100|134|135|153|1813|1814	ADA|ADORA1|ADORA2A|ADRB1|DRD2|DRD3	-7.47679	0	36	0	0	-15.3079	55.0856102
1	-8.96539	GO:0031279	M1	1	0	GO Biological Processes	19	regulation of cyclase activity	-8.96539	24.94443	13.61302	30242	53	183	8	4.371585	1.511428	775|1813|1814|1910|4842|4843|4846|729230	CACNA1C|DRD2|DRD3|EDNRB|NOS1|NOS2|NOS3|CCR2	-7.3526	0	36	0	0	-15.3079	55.0856102
1	-8.81164	GO:0098801	M1	1	0	GO Biological Processes	19	regulation of renal system process	-8.81164	33.05137	14.80346	30242	35	183	7	3.825137	1.417845	134|135|185|1813|1814|1910|2149	ADORA1|ADORA2A|AGTR1|DRD2|DRD3|EDNRB|F2R	-7.20895	0	36	0	0	-15.3079	55.0856102
1	-8.64422	GO:0055067	M1	1	0	GO Biological Processes	19	monovalent inorganic cation homeostasis	-8.64422	12.03858	10.6101	30242	151	183	11	6.010929	1.757049	134|135|185|596|760|1080|1813|1814|1910|3757|3778	ADORA1|ADORA2A|AGTR1|BCL2|CA2|CFTR|DRD2|DRD3|EDNRB|KCNH2|KCNMA1	-7.04684	0	36	0	0	-15.3079	55.0856102
1	-8.64294	GO:0035813	M1	1	0	GO Biological Processes	19	regulation of renal sodium excretion	-8.64294	47.21624	16.53051	30242	21	183	6	3.278689	1.316393	134|135|185|1813|1814|1910	ADORA1|ADORA2A|AGTR1|DRD2|DRD3|EDNRB	-7.04684	0	36	0	0	-15.3079	55.0856102
1	-8.64294	GO:0050802	M1	1	0	GO Biological Processes	19	circadian sleep/wake cycle, sleep	-8.64294	47.21624	16.53051	30242	21	183	6	3.278689	1.316393	100|134|135|153|1813|1814	ADA|ADORA1|ADORA2A|ADRB1|DRD2|DRD3	-7.04684	0	36	0	0	-15.3079	55.0856102
1	-8.37772	GO:0035812	M1	1	0	GO Biological Processes	19	renal sodium excretion	-8.37772	43.11048	15.76342	30242	23	183	6	3.278689	1.316393	134|135|185|1813|1814|1910	ADORA1|ADORA2A|AGTR1|DRD2|DRD3|EDNRB	-6.81115	0	36	0	0	-15.3079	55.0856102
1	-8.25497	GO:0044062	M1	1	0	GO Biological Processes	19	regulation of excretion	-8.25497	41.31421	15.41584	30242	24	183	6	3.278689	1.316393	134|135|185|1813|1814|1910	ADORA1|ADORA2A|AGTR1|DRD2|DRD3|EDNRB	-6.69346	0	36	0	0	-15.3079	55.0856102

1	-5.94226	GO:0055078	M1	1	0	GO Biological Processes	19	sodium ion homeostasis	-5.94226	17.70609	9.763429	30242	56	183	6	3.278689	1.316393	134|135|185|1813|1814|1910	ADORA1|ADORA2A|AGTR1|DRD2|DRD3|EDNRB	-4.59693	0	36	0	0	-15.3079	55.0856102
1	-5.90705	GO:0035815	M1	1	0	GO Biological Processes	19	positive regulation of renal sodium excretion	-5.90705	47.21624	13.49554	30242	14	183	4	2.185792	1.080886	135|1813|1814|1910	ADORA2A|DRD2|DRD3|EDNRB	-4.56779	0	36	0	0	-15.3079	55.0856102
1	-5.86815	GO:0045761	M1	1	0	GO Biological Processes	19	regulation of adenylate cyclase activity	-5.86815	25.82138	10.9613	30242	32	183	5	2.73224	1.205087	775|1813|1814|1910|729230	CACNA1C|DRD2|DRD3|EDNRB|CCR2	-4.53728	0	36	0	0	-15.3079	55.0856102
1	-5.23453	GO:0007194	M1	1	0	GO Biological Processes	19	negative regulation of adenylate cyclase activity	-5.23453	33.05137	11.18758	30242	20	183	4	2.185792	1.080886	1813|1814|1910|729230	DRD2|DRD3|EDNRB|CCR2	-3.97355	0	36	0	0	-15.3079	55.0856102
1	-5.05972	GO:0014821	M1	1	0	GO Biological Processes	19	phasic smooth muscle contraction	-5.05972	30.0467	10.63401	30242	22	183	4	2.185792	1.080886	1813|1910|3357|5024	DRD2|EDNRB|HTR2B|P2RX3	-3.8175	0	36	0	0	-15.3079	55.0856102
1	-4.9788	GO:0031280	M1	1	0	GO Biological Processes	19	negative regulation of cyclase activity	-4.9788	28.74032	10.38417	30242	23	183	4	2.185792	1.080886	1813|1814|1910|729230	DRD2|DRD3|EDNRB|CCR2	-3.74566	0	36	0	0	-15.3079	55.0856102
1	-4.75751	GO:0051350	M1	1	0	GO Biological Processes	19	negative regulation of lyase activity	-4.75751	25.42413	9.721283	30242	26	183	4	2.185792	1.080886	1813|1814|1910|729230	DRD2|DRD3|EDNRB|CCR2	-3.55409	0	36	0	0	-15.3079	55.0856102
1	-4.12183	GO:0035810	M1	1	0	GO Biological Processes	19	positive regulation of urine volume	-4.12183	35.41218	10.04865	30242	14	183	3	1.639344	0.938686	135|1813|1910	ADORA2A|DRD2|EDNRB	-2.99883	0	36	0	0	-15.3079	55.0856102
1	-3.5109	GO:0035809	M1	1	0	GO Biological Processes	19	regulation of urine volume	-3.5109	22.53502	7.883985	30242	22	183	3	1.639344	0.938686	135|1813|1910	ADORA2A|DRD2|EDNRB	-2.45542	0	36	0	0	-15.3079	55.0856102
1	-15.1686	GO:0008285	M1	1	0	GO Biological Processes	19	negative regulation of cell population proliferation	-15.1686	6.364191	11.83507	30242	779	183	30	16.39344	2.736716	134|135|140|148|351|367|472|596|836|1545|1813|2149|2263|3558|3569|3576|4221|4846|4914|4988|5467|5468|5743|6347|6532|7040|7046|7124|7157|7421	ADORA1|ADORA2A|ADORA3|ADRA1A|APP|AR|ATM|BCL2|CASP3|CYP1B1|DRD2|F2R|FGFR2|IL2|IL6|CXCL8|MEN1|NOS3|NTRK1|OPRM1|PPARD|PPARG|PTGS2|CCL2|SLC6A4|TGFB1|TGFBR1|TNF|TP53|VDR	-13.1713	0	37	1	1	-15.1686	6.364191166
1	-9.68605	GO:0045596	M1	1	0	GO Biological Processes	19	negative regulation of cell differentiation	-9.68605	5.32306	8.916474	30242	683	183	22	12.02186	2.404073	351|595|1814|1910|1956|2147|3558|3569|4221|4318|4792|5465|5467|5468|6532|7040|7046|7099|7124|7157|7442|59341	APP|CCND1|DRD3|EDNRB|EGFR|F2|IL2|IL6|MEN1|MMP9|NFKBIA|PPARA|PPARD|PPARG|SLC6A4|TGFB1|TGFBR1|TLR4|TNF|TP53|TRPV1|TRPV4	-8.02662	0	37	0	0	-15.1686	6.364191166
1	-15.0442	GO:0006820	M1	1	0	GO Biological Processes	19	anion transport	-15.0442	8.11674	12.63447	30242	509	183	25	13.6612	2.538762	134|135|624|760|762|1080|1636|1645|1813|1814|1815|2554|2558|2566|2915|3351|4363|4843|4915|5243|6513|6523|7442|9429|11255	ADORA1|ADORA2A|BDKRB2|CA2|CA4|CFTR|ACE|AKR1C1|DRD2|DRD3|DRD4|GABRA1|GABRA5|GABRG2|GRM5|HTR1B|ABCC1|NOS2|NTRK2|ABCB1|SLC2A1|SLC5A1|TRPV1|ABCG2|HRH3	-13.0497	0	38	1	1	-15.0442	10.29468781
1	-13.3384	GO:0015849	M1	1	0	GO Biological Processes	19	organic acid transport	-13.3384	10.29469	12.72966	30242	305	183	19	10.38251	2.254873	134|135|624|1636|1645|1813|1814|1815|3351|4363|4915|5243|5465|5467|5468|6513|7442|9429|11255	ADORA1|ADORA2A|BDKRB2|ACE|AKR1C1|DRD2|DRD3|DRD4|HTR1B|ABCC1|NTRK2|ABCB1|PPARA|PP	-11.4346	0	38	0	0	-15.0442	10.29468781

<table>
<tr><td></td><td></td><td></td><td></td><td></td><td></td><td></td><td></td><td></td><td></td><td></td><td></td><td></td><td></td><td></td><td></td><td></td><td></td><td></td><td>ARD|PPARG|SLC2A1|TRPV1|ABCG2|HRH3</td><td></td><td></td><td></td><td></td><td></td><td></td><td></td></tr>
<tr><td>1</td><td>-12.2901</td><td>GO:0010876</td><td>M1</td><td>1</td><td>0</td><td>GO Biological Processes</td><td>19</td><td>lipid localization</td><td>-12.2901</td><td>7.746414</td><td>11.22494</td><td>30242</td><td>448</td><td>183</td><td>21</td><td>11.47541</td><td>2.356083</td><td>185|624|1080|1588|1636|1645|1813|1814|1815|3569|4363|4792|4843|5243|5444|5465|5467|5468|6513|7124|10280</td><td>AGTR1|BDKRB2|CFTR|CYP19A1|ACE|AKR1C1|DRD2|DRD3|DRD4|IL6|ABCC1|NFKBIA|NOS2|ABCB1|PON1|PPARA|PPARD|PPARG|SLC2A1|TNF|SIGMAR1</td><td>-10.4483</td><td>0</td><td>38</td><td>0</td><td>0</td><td>-15.0442</td><td>10.29468781</td></tr>
<tr><td>1</td><td>-11.2932</td><td>GO:0006869</td><td>M1</td><td>1</td><td>0</td><td>GO Biological Processes</td><td>19</td><td>lipid transport</td><td>-11.2932</td><td>7.889145</td><td>10.7948</td><td>30242</td><td>398</td><td>183</td><td>19</td><td>10.38251</td><td>2.254873</td><td>185|624|1080|1588|1636|1645|1813|1814|1815|4363|4792|4843|5243|5444|5465|5467|5468|6513|10280</td><td>AGTR1|BDKRB2|CFTR|CYP19A1|ACE|AKR1C1|DRD2|DRD3|DRD4|ABCC1|NFKBIA|NOS2|ABCB1|PON1|PPARA|PPARD|PPARG|SLC2A1|SIGMAR1</td><td>-9.51387</td><td>0</td><td>38</td><td>0</td><td>0</td><td>-15.0442</td><td>10.29468781</td></tr>
<tr><td>1</td><td>-11.1535</td><td>GO:0015711</td><td>M1</td><td>1</td><td>0</td><td>GO Biological Processes</td><td>19</td><td>organic anion transport</td><td>-11.1535</td><td>8.37922</td><td>10.91239</td><td>30242</td><td>355</td><td>183</td><td>18</td><td>9.836066</td><td>2.201413</td><td>134|135|624|762|1080|1636|1645|1813|1814|1815|3351|4363|4843|4915|6513|7442|9429|11255</td><td>ADORA1|ADORA2A|BDKRB2|CA4|CFTR|ACE|AKR1C1|DRD2|DRD3|DRD4|HTR1B|ABCC1|NOS2|NTRK2|SLC2A1|TRPV1|ABCG2|HRH3</td><td>-9.38236</td><td>0</td><td>38</td><td>0</td><td>0</td><td>-15.0442</td><td>10.29468781</td></tr>
<tr><td>1</td><td>-9.83495</td><td>GO:0046942</td><td>M1</td><td>1</td><td>0</td><td>GO Biological Processes</td><td>19</td><td>carboxylic acid transport</td><td>-9.83495</td><td>9.046907</td><td>10.44027</td><td>30242</td><td>274</td><td>183</td><td>15</td><td>8.196721</td><td>2.027793</td><td>134|135|624|1636|1645|1813|1814|1815|3351|4363|4843|4915|7442|9429|11255</td><td>ADORA1|ADORA2A|BDKRB2|ACE|AKR1C1|DRD2|DRD3|DRD4|HTR1B|ABCC1|NOS2|NTRK2|TRPV1|ABCG2|HRH3</td><td>-8.16917</td><td>0</td><td>38</td><td>0</td><td>0</td><td>-15.0442</td><td>10.29468781</td></tr>
<tr><td>1</td><td>-14.9992</td><td>GO:0009266</td><td>M1</td><td>1</td><td>0</td><td>GO Biological Processes</td><td>19</td><td>response to temperature stimulus</td><td>-14.9992</td><td>15.69478</td><td>15.38544</td><td>30242</td><td>179</td><td>183</td><td>17</td><td>9.289617</td><td>2.145862</td><td>134|153|154|472|3066|3351|3356|3357|4792|4842|4846|4914|5024|5743|7442|8989|59341</td><td>ADORA1|ADRB1|ADRB2|ATM|HDAC2|HTR1B|HTR2A|HTR2B|NFKBIA|NOS1|NOS3|NTRK1|P2RX3|PTGS2|TRPV1|TRPA1|TRPV4</td><td>-13.01</td><td>0</td><td>39</td><td>1</td><td>1</td><td>-14.9992</td><td>15.69478279</td></tr>
<tr><td>1</td><td>-6.3976</td><td>GO:0009408</td><td>M1</td><td>1</td><td>0</td><td>GO Biological Processes</td><td>19</td><td>response to heat</td><td>-6.3976</td><td>11.9104</td><td>8.985284</td><td>30242</td><td>111</td><td>183</td><td>8</td><td>4.371585</td><td>1.511428</td><td>472|3066|4842|4846|5024|5743|7442|59341</td><td>ATM|HDAC2|NOS1|NOS3|P2RX3|PTGS2|TRPV1|TRPV4</td><td>-5.01719</td><td>0</td><td>39</td><td>0</td><td>0</td><td>-14.9992</td><td>15.69478279</td></tr>
<tr><td>1</td><td>-4.17</td><td>GO:0034605</td><td>M1</td><td>1</td><td>0</td><td>GO Biological Processes</td><td>19</td><td>cellular response to heat</td><td>-4.17</td><td>11.80406</td><td>7.061066</td><td>30242</td><td>70</td><td>183</td><td>5</td><td>2.73224</td><td>1.205087</td><td>472|3066|5743|7442|59341</td><td>ATM|HDAC2|PTGS2|TRPV1|TRPV4</td><td>-3.04034</td><td>0</td><td>39</td><td>0</td><td>0</td><td>-14.9992</td><td>15.69478279</td></tr>
<tr><td>1</td><td>-14.849</td><td>GO:0009612</td><td>M1</td><td>1</td><td>0</td><td>GO Biological Processes</td><td>19</td><td>response to mechanical stimulus</td><td>-14.849</td><td>13.7714</td><td>14.69783</td><td>30242</td><td>216</td><td>183</td><td>18</td><td>9.836066</td><td>2.201413</td><td>834|1813|1956|3356|4792|4914|5024|5138|5290|5734|5743|5970|6513|7099|7132|8989|23621|59341</td><td>CASP1|DRD2|EGFR|HTR2A|NFKBIA|NTRK1|P2RX3|PDE2A|PIK3CA|PTGER4|PTGS2|RELA|SLC2A1|TLR4|TNFRSF1A|TRPA1|BACE1|TRPV4</td><td>-12.8691</td><td>0</td><td>40</td><td>1</td><td>1</td><td>-14.849</td><td>16.32166228</td></tr>
<tr><td>1</td><td>-14.8105</td><td>GO:0071214</td><td>M1</td><td>1</td><td>0</td><td>GO Biological Processes</td><td>19</td><td>cellular response to abiotic stimulus</td><td>-14.8105</td><td>10.4216</td><td>13.48908</td><td>30242</td><td>333</td><td>183</td><td>21</td><td>11.47541</td><td>2.356083</td><td>472|624|834|836|1956|4312|4313|4314|4318|5138|5290|5734|5743|632</td><td>ATM|BDKRB2|CASP1|CASP3|EGFR|MMP1|MMP2|MMP3|MMP9|</td><td>-12.8372</td><td>0</td><td>40</td><td>0</td><td>0</td><td>-14.849</td><td>16.32166228</td></tr>
</table>

																		6|6513|7099|7132|7157|7442|10135|59341	PDE2A|PIK3CA|PTGER4|PTGS2|SCN2A|SLC2A1|TLR4|TNFRSF1A|TP53|TRPV1|NAMPT|TRPV4							
1	-14.8105	GO:0104004	M1	1	0	GO Biological Processes	19	cellular response to environmental stimulus	-14.8105	10.4216	13.48908	30242	333	183	21	11.47541	2.356083	472|624|834|836|1956|4312|4313|4314|4318|5138|5290|5734|5743|6326|6513|7099|7132|7157|7442|10135|59341	ATM|BDKRB2|CASP1|CASP3|EGFR|MMP1|MMP2|MMP3|MMP9|PDE2A|PIK3CA|PTGER4|PTGS2|SCN2A|SLC2A1|TLR4|TNFRSF1A|TP53|TRPV1|NAMPT|TRPV4	-12.8372	0	40	0	0	-14.849	16.32166228
1	-7.96388	GO:0071496	M1	1	0	GO Biological Processes	19	cellular response to external stimulus	-7.96388	7.229986	8.741846	30242	320	183	14	7.650273	1.964856	596|834|1956|3383|5138|5465|5734|5743|6513|7099|7132|7157|7421|10135	BCL2|CASP1|EGFR|ICAM1|PDE2A|PPARA|PTGER4|PTGS2|SLC2A1|TLR4|TNFRSF1A|TP53|VDR|NAMPT	-6.4316	0	40	0	0	-14.849	16.32166228
1	-7.4682	GO:0071260	M1	1	0	GO Biological Processes	19	cellular response to mechanical stimulus	-7.4682	16.32166	10.77363	30242	81	183	8	4.371585	1.511428	834|1956|5138|5734|5743|6513|7099|7132	CASP1|EGFR|PDE2A|PTGER4|PTGS2|SLC2A1|TLR4|TNFRSF1A	-5.98402	0	40	0	0	-14.849	16.32166228
1	-14.7087	GO:0044706	M1	1	0	GO Biological Processes	19	multi-multicellular organism process	-14.7087	13.52101	14.54345	30242	220	183	18	9.836066	2.201413	151|152|367|596|1268|1543|1723|2099|4221|4313|4318|5241|5467|5743|6513|6532|7421|10135	ADRA2B|ADRA2C|AR|BCL2|CNR1|CYP1A1|DHODH|ESR1|MEN1|MMP2|MMP9|PGR|PPARD|PTGS2|SLC2A1|SLC6A4|VDR|NAMPT	-12.7406	0	41	1	1	-14.7087	25.42412778
1	-13.2125	GO:0007565	M1	1	0	GO Biological Processes	19	female pregnancy	-13.2125	13.70005	13.81037	30242	193	183	16	8.743169	2.088053	151|152|367|596|1268|1723|2099|4221|4313|4318|5241|5467|5743|6513|7421|10135	ADRA2B|ADRA2C|AR|BCL2|CNR1|DHODH|ESR1|MEN1|MMP2|MMP9|PGR|PPARD|PTGS2|SLC2A1|VDR|NAMPT	-11.3142	0	41	0	0	-14.7087	25.42412778
1	-8.40405	GO:0060135	M1	1	0	GO Biological Processes	19	maternal process involved in female pregnancy	-8.40405	21.32346	12.49886	30242	62	183	8	4.371585	1.511428	367|1268|2099|4221|5241|5467|5743|7421	AR|CNR1|ESR1|MEN1|PGR|PPARD|PTGS2|VDR	-6.83338	0	41	0	0	-14.7087	25.42412778
1	-4.75751	GO:0046697	M1	1	0	GO Biological Processes	19	decidualization	-4.75751	25.42413	9.721283	30242	26	183	4	2.185792	1.080886	4221|5467|5743|7421	MEN1|PPARD|PTGS2|VDR	-3.55409	0	41	0	0	-14.7087	25.42412778
1	-4.51884	GO:0001890	M1	1	0	GO Biological Processes	19	placenta development	-4.51884	7.977916	6.571802	30242	145	183	7	3.825137	1.417845	100|2263|4221|5467|5468|5743|7421	ADA|FGFR2|MEN1|PPARD|PPARG|PTGS2|VDR	-3.35305	0	41	0	0	-14.7087	25.42412778
1	-4.23163	GO:0001893	M1	1	0	GO Biological Processes	19	maternal placenta development	-4.23163	18.88649	8.261175	30242	35	183	4	2.185792	1.080886	4221|5467|5743|7421	MEN1|PPARD|PTGS2|VDR	-3.09596	0	41	0	0	-14.7087	25.42412778
1	-2.90759	GO:0033280	M1	1	0	GO Biological Processes	19	response to vitamin D	-2.90759	14.16487	6.080415	30242	35	183	3	1.639344	0.938686	249|5743|7421	ALPL|PTGS2|VDR	-1.91274	0	41	0	0	-14.7087	25.42412778
1	-2.53666	GO:0043616	M1	1	0	GO Biological Processes	19	keratinocyte proliferation	-2.53666	10.54831	5.111451	30242	47	183	3	1.639344	0.938686	2263|5467|7421	FGFR2|PPARD|VDR	-1.58173	0	41	0	0	-14.7087	25.42412778
1	-2.14076	GO:0045682	M1	1	0	GO Biological Processes	19	regulation of epidermis development	-2.14076	7.627238	4.173377	30242	65	183	3	1.639344	0.938686	5467|7421|9475	PPARD|VDR|ROCK2	-1.21893	0	41	0	0	-14.7087	25.42412778
1	-14.5372	GO:0009636	M1	1	0	GO Biological	19	response to toxic	-14.5372	11.98427	13.93293	30242	262	183	19	10.38251	2.254873	596|1543|1545|1728|18	BCL2|CYP1A1|CYP1	-12.5716	0	42	1	1	-14.5372	18.88649493

						Processes		substance										13|2936|4129|4846|4985|5159|5243|5444|5582|5742|5743|6335|6532|7298|9429	B1|NQO1|DRD2|GSR|MAOB|NOS3|OPRD1|PDGFRB|ABCB1|PON1|PRKCG|PTGS1|PTGS2|SCN9A|SLC6A4|TYMS|ABCG2							
1	-4.95924	GO:0097237	M1	1	0	GO Biological Processes	19	cellular response to toxic substance	-4.95924	9.329015	7.251498	30242	124	183	7	3.825137	1.417845	1728|2936|4846|4985|5742|5743|9429	NQO1|GSR|NOS3|OPRD1|PTGS1|PTGS2|ABCG2	-3.7294	0	42	0	0	-14.5372	18.88649493
1	-4.38792	GO:0098754	M1	1	0	GO Biological Processes	19	detoxification	-4.38792	7.610512	6.375029	30242	152	183	7	3.825137	1.417845	1728|2936|4846|5243|5742|5743|9429	NQO1|GSR|NOS3|ABCB1|PTGS1|PTGS2|ABCG2	-3.23843	0	42	0	0	-14.5372	18.88649493
1	-4.23163	GO:0034405	M1	1	0	GO Biological Processes	19	response to fluid shear stress	-4.23163	18.88649	8.261175	30242	35	183	4	2.185792	1.080886	760|4846|5159|5743	CA2|NOS3|PDGFRB|PTGS2	-3.09596	0	42	0	0	-14.5372	18.88649493
1	-4.11161	GO:1990748	M1	1	0	GO Biological Processes	19	cellular detoxification	-4.11161	8.547767	6.354964	30242	116	183	6	3.278689	1.316393	1728|2936|4846|5742|5743|9429	NQO1|GSR|NOS3|PTGS1|PTGS2|ABCG2	-2.98937	0	42	0	0	-14.5372	18.88649493
1	-3.41969	GO:0098869	M1	1	0	GO Biological Processes	19	cellular oxidant detoxification	-3.41969	8.181031	5.640337	30242	101	183	5	2.73224	1.205087	1728|2936|4846|5742|5743	NQO1|GSR|NOS3|PTGS1|PTGS2	-2.37416	0	42	0	0	-14.5372	18.88649493
1	-14.4566	GO:0043271	M1	1	0	GO Biological Processes	19	negative regulation of ion transport	-14.4566	16.42304	15.30993	30242	161	183	16	8.743169	2.088053	134|150|596|1268|1813|1814|1815|3351|3356|3383|3757|4129|4318|4842|5743|11255	ADORA1|ADRA2A|BCL2|CNR1|DRD2|DRD3|DRD4|HTR1B|HTR2A|ICAM1|KCNH2|MAOB|MMP9|NOS1|PTGS2|HRH3	-12.4937	0	43	1	1	-14.4566	82.6284153
1	-13.5933	GO:0001963	M1	1	0	GO Biological Processes	19	synaptic transmission, dopaminergic	-13.5933	57.20429	22.37041	30242	26	183	9	4.918033	1.598524	135|1812|1813|1814|1815|5743|6530|6531|6532	ADORA2A|DRD1|DRD2|DRD3|DRD4|PTGS2|SLC6A2|SLC6A3|SLC6A4	-11.6714	0	43	0	0	-14.4566	82.6284153
1	-11.7912	GO:0042417	M1	1	0	GO Biological Processes	19	dopamine metabolic process	-11.7912	38.13619	18.10677	30242	39	183	9	4.918033	1.598524	1812|1813|1814|1815|2903|3350|4128|4129|6531	DRD1|DRD2|DRD3|DRD4|GRIN2A|HTR1A|MAOA|MAOB|SLC6A3	-9.97902	0	43	0	0	-14.4566	82.6284153
1	-11.3105	GO:0090494	M1	1	0	GO Biological Processes	19	dopamine uptake	-11.3105	68.04693	21.57529	30242	17	183	7	3.825137	1.417845	1812|1813|1814|1815|6530|6531|225689	DRD1|DRD2|DRD3|DRD4|SLC6A2|SLC6A3|MAPK15	-9.52787	0	43	0	0	-14.4566	82.6284153
1	-11.0989	GO:0090493	M1	1	0	GO Biological Processes	19	catecholamine uptake	-11.0989	64.26655	20.94936	30242	18	183	7	3.825137	1.417845	1812|1813|1814|1815|6530|6531|225689	DRD1|DRD2|DRD3|DRD4|SLC6A2|SLC6A3|MAPK15	-9.33907	0	43	0	0	-14.4566	82.6284153
1	-10.9625	GO:0098657	M1	1	0	GO Biological Processes	19	import into cell	-10.9625	10.87216	11.67492	30242	228	183	15	8.196721	2.027793	1812|1813|1814|1815|3757|4842|6513|6523|6530|6531|6532|7442|51305|59341|225689	DRD1|DRD2|DRD3|DRD4|KCNH2|NOS1|SLC2A1|SLC5A1|SLC6A2|SLC6A3|SLC6A4|TRPV1|KCNK9|TRPV4|MAPK15	-9.2106	0	43	0	0	-14.4566	82.6284153
1	-10.501	GO:0098810	M1	1	0	GO Biological Processes	19	neurotransmitter reuptake	-10.501	37.77299	16.98422	30242	35	183	8	4.371585	1.511428	1812|1813|1814|1815|4842|6530|6531|6532	DRD1|DRD2|DRD3|DRD4|NOS1|SLC6A2|SLC6A3|SLC6A4	-8.77775	0	43	0	0	-14.4566	82.6284153
1	-10.4254	GO:0006584	M1	1	0	GO Biological Processes	19	catecholamine metabolic process	-10.4254	27.54281	15.23221	30242	54	183	9	4.918033	1.598524	1812|1813|1814|1815|2903|3350|4128|4129|6531	DRD1|DRD2|DRD3|DRD4|GRIN2A|HTR1A|MAOA|MAOB|SLC6A3	-8.71086	0	43	0	0	-14.4566	82.6284153

1	-10.4254	GO:0009712	M1	1	0	GO Biological Processes	19	catechol-containing compound metabolic process	-10.4254	27.54281	15.23221	30242	54	183	9	4.918033	1.598524	1812|1813|1814|1815|2903|3350|4128|4129|6531	DRD1|DRD2|DRD3|DRD4|GRIN2A|HTR1A|MAOA|MAOB|SLC6A3	-8.71086	0	43	0	0	-14.4566	82.6284153
1	-10.3921	GO:0051583	M1	1	0	GO Biological Processes	19	dopamine uptake involved in synaptic transmission	-10.3921	82.62842	22.0673	30242	12	183	6	3.278689	1.316393	1812|1813|1814|1815|6530|6531	DRD1|DRD2|DRD3|DRD4|SLC6A2|SLC6A3	-8.68413	0	43	0	0	-14.4566	82.6284153
1	-10.3921	GO:0051934	M1	1	0	GO Biological Processes	19	catecholamine uptake involved in synaptic transmission	-10.3921	82.62842	22.0673	30242	12	183	6	3.278689	1.316393	1812|1813|1814|1815|6530|6531	DRD1|DRD2|DRD3|DRD4|SLC6A2|SLC6A3	-8.68413	0	43	0	0	-14.4566	82.6284153
1	-10.3921	GO:0060134	M1	1	0	GO Biological Processes	19	prepulse inhibition	-10.3921	82.62842	22.0673	30242	12	183	6	3.278689	1.316393	135|1812|1813|1814|6531|23621	ADORA2A|DRD1|DRD2|DRD3|SLC6A3|BACE1	-8.68413	0	43	0	0	-14.4566	82.6284153
1	-9.93528	GO:0001964	M1	1	0	GO Biological Processes	19	startle response	-9.93528	46.27191	17.6689	30242	25	183	7	3.825137	1.417845	135|1812|1813|1814|2903|6531|23621	ADORA2A|DRD1|DRD2|DRD3|GRIN2A|SLC6A3|BACE1	-8.25649	0	43	0	0	-14.4566	82.6284153
1	-9.72922	GO:0001504	M1	1	0	GO Biological Processes	19	neurotransmitter uptake	-9.72922	30.74546	15.22983	30242	43	183	8	4.371585	1.511428	1812|1813|1814|1815|4842|6530|6531|6532	DRD1|DRD2|DRD3|DRD4|NOS1|SLC6A2|SLC6A3|SLC6A4	-8.06853	0	43	0	0	-14.4566	82.6284153
1	-8.84484	GO:0018958	M1	1	0	GO Biological Processes	19	phenol-containing compound metabolic process	-8.84484	15.02335	11.49663	30242	110	183	10	5.464481	1.680144	596|1812|1813|1814|1815|2903|3350|4128|4129|6531	BCL2|DRD1|DRD2|DRD3|DRD4|GRIN2A|HTR1A|MAOA|MAOB|SLC6A3	-7.23881	0	43	0	0	-14.4566	82.6284153
1	-8.56059	GO:0015908	M1	1	0	GO Biological Processes	19	fatty acid transport	-8.56059	17.29432	11.80687	30242	86	183	9	4.918033	1.598524	624|1636|1813|1814|1815|5465|5467|5468|6513	BDKRB2|ACE|DRD2|DRD3|DRD4|PPARA|PPARD|PPARG|SLC2A1	-6.97527	0	43	0	0	-14.4566	82.6284153
1	-8.50942	GO:0009308	M1	1	0	GO Biological Processes	19	amine metabolic process	-8.50942	13.88713	10.99049	30242	119	183	10	5.464481	1.680144	1543|1812|1813|1814|1815|2903|3350|4128|4129|6531	CYP1A1|DRD1|DRD2|DRD3|DRD4|GRIN2A|HTR1A|MAOA|MAOB|SLC6A3	-6.92728	0	43	0	0	-14.4566	82.6284153
1	-8.46275	GO:0034776	M1	1	0	GO Biological Processes	19	response to histamine	-8.46275	75.11674	19.18328	30242	11	183	5	2.73224	1.205087	1813|1814|1815|2554|2566	DRD2|DRD3|DRD4|GABRA1|GABRG2	-6.88741	0	43	0	0	-14.4566	82.6284153
1	-8.4279	GO:0006814	M1	1	0	GO Biological Processes	19	sodium ion transport	-8.4279	8.76873	9.526437	30242	245	183	13	7.103825	1.898976	154|1813|1814|1815|4842|6326|6331|6335|6336|6523|6530|6531|6532	ADRB2|DRD2|DRD3|DRD4|NOS1|SCN2A|SCN5A|SCN9A|SCN10A|SLC5A1|SLC6A2|SLC6A3|SLC6A4	-6.85517	0	43	0	0	-14.4566	82.6284153
1	-8.02647	GO:0051926	M1	1	0	GO Biological Processes	19	negative regulation of calcium ion transport	-8.02647	19.16021	11.78345	30242	69	183	8	4.371585	1.511428	150|596|1813|1814|1815|3383|4842|5743	ADRA2A|BCL2|DRD2|DRD3|DRD4|ICAM1|NOS1|PTGS2	-6.48727	0	43	0	0	-14.4566	82.6284153
1	-7.89931	GO:0006576	M1	1	0	GO Biological Processes	19	cellular biogenic amine metabolic process	-7.89931	14.58149	10.72043	30242	102	183	9	4.918033	1.598524	1812|1813|1814|1815|2903|3350|4128|4129|6531	DRD1|DRD2|DRD3|DRD4|GRIN2A|HTR1A|MAOA|MAOB|SLC6A3	-6.37172	0	43	0	0	-14.4566	82.6284153
1	-7.89451	GO:0035725	M1	1	0	GO Biological Processes	19	sodium ion transmembrane transport	-7.89451	10.2125	9.618349	30242	178	183	11	6.010929	1.757049	1814|1815|4842|6326|6331|6335|6336|6523|6530|6531|6532	DRD3|DRD4|NOS1|SCN2A|SCN5A|SCN9A|SCN10A|SLC5A1|SLC6A2|SLC6A3|SLC6A4	-6.36784	0	43	0	0	-14.4566	82.6284153
1	-7.49122	GO:0071715	M1	1	0	GO Biological Processes	19	icosanoid transport	-7.49122	21.82637	11.84032	30242	53	183	7	3.825137	1.417845	624|1636|1813|1814|1815|4363|4843	BDKRB2|ACE|DRD2|DRD3|DRD4|ABCC1|	-6.00535	0	43	0	0	-14.4566	82.6284153

<table>
<tr><td></td><td></td><td></td><td></td><td></td><td></td><td></td><td></td><td></td><td></td><td></td><td></td><td></td><td></td><td></td><td></td><td></td><td></td><td></td><td>NOS2</td><td></td><td></td><td></td><td></td><td></td><td></td><td></td></tr>
<tr><td>1</td><td>-7.47453</td><td>GO:0044106</td><td>M1</td><td>1</td><td>0</td><td>GO Biological Processes</td><td>19</td><td>cellular amine metabolic process</td><td>-7.47453</td><td>13.04659</td><td>10.05467</td><td>30242</td><td>114</td><td>183</td><td>9</td><td>4.918033</td><td>1.598524</td><td>1812|1813|1814|1815|2903|3350|4128|4129|6531</td><td>DRD1|DRD2|DRD3|DRD4|GRIN2A|HTR1A|MAOA|MAOB|SLC6A3</td><td>-5.98951</td><td>0</td><td>43</td><td>0</td><td>0</td><td>-14.4566</td><td>82.6284153</td></tr>
<tr><td>1</td><td>-7.34865</td><td>GO:0051580</td><td>M1</td><td>1</td><td>0</td><td>GO Biological Processes</td><td>19</td><td>regulation of neurotransmitter uptake</td><td>-7.34865</td><td>48.60495</td><td>15.31898</td><td>30242</td><td>17</td><td>183</td><td>5</td><td>2.73224</td><td>1.205087</td><td>1812|1813|1814|1815|4842</td><td>DRD1|DRD2|DRD3|DRD4|NOS1</td><td>-5.87776</td><td>0</td><td>43</td><td>0</td><td>0</td><td>-14.4566</td><td>82.6284153</td></tr>
<tr><td>1</td><td>-7.298</td><td>GO:1903169</td><td>M1</td><td>1</td><td>0</td><td>GO Biological Processes</td><td>19</td><td>regulation of calcium ion transmembrane transport</td><td>-7.298</td><td>10.39351</td><td>9.266214</td><td>30242</td><td>159</td><td>183</td><td>10</td><td>5.464481</td><td>1.680144</td><td>150|775|1812|1813|1814|1815|2147|2149|4842|5142</td><td>ADRA2A|CACNA1C|DRD1|DRD2|DRD3|DRD4|F2|F2R|NOS1|PDE4B</td><td>-5.83361</td><td>0</td><td>43</td><td>0</td><td>0</td><td>-14.4566</td><td>82.6284153</td></tr>
<tr><td>1</td><td>-7.05003</td><td>GO:0051584</td><td>M1</td><td>1</td><td>0</td><td>GO Biological Processes</td><td>19</td><td>regulation of dopamine uptake involved in synaptic transmission</td><td>-7.05003</td><td>82.62842</td><td>18.01669</td><td>30242</td><td>8</td><td>183</td><td>4</td><td>2.185792</td><td>1.080886</td><td>1812|1813|1814|1815</td><td>DRD1|DRD2|DRD3|DRD4</td><td>-5.61068</td><td>0</td><td>43</td><td>0</td><td>0</td><td>-14.4566</td><td>82.6284153</td></tr>
<tr><td>1</td><td>-7.05003</td><td>GO:0051940</td><td>M1</td><td>1</td><td>0</td><td>GO Biological Processes</td><td>19</td><td>regulation of catecholamine uptake involved in synaptic transmission</td><td>-7.05003</td><td>82.62842</td><td>18.01669</td><td>30242</td><td>8</td><td>183</td><td>4</td><td>2.185792</td><td>1.080886</td><td>1812|1813|1814|1815</td><td>DRD1|DRD2|DRD3|DRD4</td><td>-5.61068</td><td>0</td><td>43</td><td>0</td><td>0</td><td>-14.4566</td><td>82.6284153</td></tr>
<tr><td>1</td><td>-6.86295</td><td>GO:0015909</td><td>M1</td><td>1</td><td>0</td><td>GO Biological Processes</td><td>19</td><td>long-chain fatty acid transport</td><td>-6.86295</td><td>17.79689</td><td>10.57752</td><td>30242</td><td>65</td><td>183</td><td>7</td><td>3.825137</td><td>1.417845</td><td>624|1636|1813|1814|1815|5468|6513</td><td>BDKRB2|ACE|DRD2|DRD3|DRD4|PPARG|SLC2A1</td><td>-5.43561</td><td>0</td><td>43</td><td>0</td><td>0</td><td>-14.4566</td><td>82.6284153</td></tr>
<tr><td>1</td><td>-6.55292</td><td>GO:0050709</td><td>M1</td><td>1</td><td>0</td><td>GO Biological Processes</td><td>19</td><td>negative regulation of protein secretion</td><td>-6.55292</td><td>16.06664</td><td>9.986913</td><td>30242</td><td>72</td><td>183</td><td>7</td><td>3.825137</td><td>1.417845</td><td>150|1813|1814|1815|2149|3156|4988</td><td>ADRA2A|DRD2|DRD3|DRD4|F2R|HMGCR|OPRM1</td><td>-5.16039</td><td>0</td><td>43</td><td>0</td><td>0</td><td>-14.4566</td><td>82.6284153</td></tr>
<tr><td>1</td><td>-6.51888</td><td>GO:0032309</td><td>M1</td><td>1</td><td>0</td><td>GO Biological Processes</td><td>19</td><td>icosanoid secretion</td><td>-6.51888</td><td>22.03424</td><td>11.01762</td><td>30242</td><td>45</td><td>183</td><td>6</td><td>3.278689</td><td>1.316393</td><td>624|1636|1813|1814|1815|4843</td><td>BDKRB2|ACE|DRD2|DRD3|DRD4|NOS2</td><td>-5.12976</td><td>0</td><td>43</td><td>0</td><td>0</td><td>-14.4566</td><td>82.6284153</td></tr>
<tr><td>1</td><td>-6.3976</td><td>GO:0015718</td><td>M1</td><td>1</td><td>0</td><td>GO Biological Processes</td><td>19</td><td>monocarboxylic acid transport</td><td>-6.3976</td><td>11.9104</td><td>8.985284</td><td>30242</td><td>111</td><td>183</td><td>8</td><td>4.371585</td><td>1.511428</td><td>624|1636|1645|1813|1814|1815|4843|9429</td><td>BDKRB2|ACE|AKR1C1|DRD2|DRD3|DRD4|NOS2|ABCG2</td><td>-5.01719</td><td>0</td><td>43</td><td>0</td><td>0</td><td>-14.4566</td><td>82.6284153</td></tr>
<tr><td>1</td><td>-5.93981</td><td>GO:0050482</td><td>M1</td><td>1</td><td>0</td><td>GO Biological Processes</td><td>19</td><td>arachidonic acid secretion</td><td>-5.93981</td><td>26.65433</td><td>11.15052</td><td>30242</td><td>31</td><td>183</td><td>5</td><td>2.73224</td><td>1.205087</td><td>624|1636|1813|1814|1815</td><td>BDKRB2|ACE|DRD2|DRD3|DRD4</td><td>-4.59631</td><td>0</td><td>43</td><td>0</td><td>0</td><td>-14.4566</td><td>82.6284153</td></tr>
<tr><td>1</td><td>-5.93981</td><td>GO:1903963</td><td>M1</td><td>1</td><td>0</td><td>GO Biological Processes</td><td>19</td><td>arachidonate transport</td><td>-5.93981</td><td>26.65433</td><td>11.15052</td><td>30242</td><td>31</td><td>183</td><td>5</td><td>2.73224</td><td>1.205087</td><td>624|1636|1813|1814|1815</td><td>BDKRB2|ACE|DRD2|DRD3|DRD4</td><td>-4.59631</td><td>0</td><td>43</td><td>0</td><td>0</td><td>-14.4566</td><td>82.6284153</td></tr>
<tr><td>1</td><td>-5.53708</td><td>GO:0042053</td><td>M1</td><td>1</td><td>0</td><td>GO Biological Processes</td><td>19</td><td>regulation of dopamine metabolic process</td><td>-5.53708</td><td>38.88396</td><td>12.19082</td><td>30242</td><td>17</td><td>183</td><td>4</td><td>2.185792</td><td>1.080886</td><td>1815|3350|4129|6531</td><td>DRD4|HTR1A|MAOB|SLC6A3</td><td>-4.23375</td><td>0</td><td>43</td><td>0</td><td>0</td><td>-14.4566</td><td>82.6284153</td></tr>
<tr><td>1</td><td>-5.53708</td><td>GO:0042069</td><td>M1</td><td>1</td><td>0</td><td>GO Biological Processes</td><td>19</td><td>regulation of catecholamine metabolic process</td><td>-5.53708</td><td>38.88396</td><td>12.19082</td><td>30242</td><td>17</td><td>183</td><td>4</td><td>2.185792</td><td>1.080886</td><td>1815|3350|4129|6531</td><td>DRD4|HTR1A|MAOB|SLC6A3</td><td>-4.23375</td><td>0</td><td>43</td><td>0</td><td>0</td><td>-14.4566</td><td>82.6284153</td></tr>
<tr><td>1</td><td>-5.45207</td><td>GO:0050905</td><td>M1</td><td>1</td><td>0</td><td>GO Biological Processes</td><td>19</td><td>neuromuscular process</td><td>-5.45207</td><td>8.932802</td><td>7.548382</td><td>30242</td><td>148</td><td>183</td><td>8</td><td>4.371585</td><td>1.511428</td><td>135|351|1812|1813|1814|2903|6531|23621</td><td>ADORA2A|APP|DRD1|DRD2|DRD3|GRIN2A|SLC6A3|BACE1</td><td>-4.15863</td><td>0</td><td>43</td><td>0</td><td>0</td><td>-14.4566</td><td>82.6284153</td></tr>
<tr><td>1</td><td>-5.36639</td><td>GO:0007195</td><td>M1</td><td>1</td><td>0</td><td>GO Biological Processes</td><td>19</td><td>adenylate cyclase-inhibiting dopamine receptor signaling pathway</td><td>-5.36639</td><td>82.62842</td><td>15.60239</td><td>30242</td><td>6</td><td>183</td><td>3</td><td>1.639344</td><td>0.938686</td><td>1813|1814|1815</td><td>DRD2|DRD3|DRD4</td><td>-4.08918</td><td>0</td><td>43</td><td>0</td><td>0</td><td>-14.4566</td><td>82.6284153</td></tr>
<tr><td>1</td><td>-4.92311</td><td>GO:0060158</td><td>M1</td><td>1</td><td>0</td><td>GO Biological Processes</td><td>19</td><td>phospholipase C-activating dopamine receptor signaling pathway</td><td>-4.92311</td><td>61.97131</td><td>13.45734</td><td>30242</td><td>8</td><td>183</td><td>3</td><td>1.639344</td><td>0.938686</td><td>1812|1813|1814</td><td>DRD1|DRD2|DRD3</td><td>-3.69979</td><td>0</td><td>43</td><td>0</td><td>0</td><td>-14.4566</td><td>82.6284153</td></tr>
<tr><td>1</td><td>-4.89137</td><td>GO:0051224</td><td>M1</td><td>1</td><td>0</td><td>GO Biological Processes</td><td>19</td><td>negative regulation of protein transport</td><td>-4.89137</td><td>9.108644</td><td>7.144881</td><td>30242</td><td>127</td><td>183</td><td>7</td><td>3.825137</td><td>1.417845</td><td>150|1813|1814|1815|2149|3156|4988</td><td>ADRA2A|DRD2|DRD3|DRD4|F2R|HMGCR|</td><td>-3.67403</td><td>0</td><td>43</td><td>0</td><td>0</td><td>-14.4566</td><td>82.6284153</td></tr>
</table>

																			OPRM1							
1	-4.80364	GO:1904950	M1	1	0	GO Biological Processes	19	negative regulation of establishment of protein localization	-4.80364	8.830518	7.008093	30242	131	183	7	3.825137	1.417845	150|1813|1814|1815|2149|3156|4988	ADRA2A|DRD2|DRD3|DRD4|F2R|HMGCR|OPRM1	-3.59532	0	43	0	0	-14.4566	82.6284153
1	-4.73339	GO:0002028	M1	1	0	GO Biological Processes	19	regulation of sodium ion transport	-4.73339	11.01712	7.425787	30242	90	183	6	3.278689	1.316393	154|1813|1814|1815|4842|6331	ADRB2|DRD2|DRD3|DRD4|NOS1|SCN5A	-3.53566	0	43	0	0	-14.4566	82.6284153
1	-4.65202	GO:1901019	M1	1	0	GO Biological Processes	19	regulation of calcium ion transmembrane transporter activity	-4.65202	10.66173	7.281089	30242	93	183	6	3.278689	1.316393	150|1813|1814|1815|4842|5142	ADRA2A|DRD2|DRD3|DRD4|NOS1|PDE4B	-3.46165	0	43	0	0	-14.4566	82.6284153
1	-4.53813	GO:0034763	M1	1	0	GO Biological Processes	19	negative regulation of transmembrane transport	-4.53813	8.033318	6.60099	30242	144	183	7	3.825137	1.417845	150|1813|1814|1815|3757|4318|7124	ADRA2A|DRD2|DRD3|DRD4|KCNH2|MMP9|TNF	-3.36786	0	43	0	0	-14.4566	82.6284153
1	-4.44459	GO:0033238	M1	1	0	GO Biological Processes	19	regulation of cellular amine metabolic process	-4.44459	21.32346	8.83349	30242	31	183	4	2.185792	1.080886	1815|3350|4129|6531	DRD4|HTR1A|MAOB|SLC6A3	-3.29079	0	43	0	0	-14.4566	82.6284153
1	-4.40051	GO:1904063	M1	1	0	GO Biological Processes	19	negative regulation of cation transmembrane transport	-4.40051	9.626611	6.842752	30242	103	183	6	3.278689	1.316393	150|1813|1814|1815|3757|4318	ADRA2A|DRD2|DRD3|DRD4|KCNH2|MMP9	-3.24867	0	43	0	0	-14.4566	82.6284153
1	-4.24017	GO:0034766	M1	1	0	GO Biological Processes	19	negative regulation of ion transmembrane transport	-4.24017	9.014009	6.570048	30242	110	183	6	3.278689	1.316393	150|1813|1814|1815|3757|4318	ADRA2A|DRD2|DRD3|DRD4|KCNH2|MMP9	-3.10224	0	43	0	0	-14.4566	82.6284153
1	-3.95744	GO:1901020	M1	1	0	GO Biological Processes	19	negative regulation of calcium ion transmembrane transporter activity	-3.95744	16.12262	7.560397	30242	41	183	4	2.185792	1.080886	150|1813|1814|1815	ADRA2A|DRD2|DRD3|DRD4	-2.8503	0	43	0	0	-14.4566	82.6284153
1	-3.95744	GO:1901385	M1	1	0	GO Biological Processes	19	regulation of voltage-gated calcium channel activity	-3.95744	16.12262	7.560397	30242	41	183	4	2.185792	1.080886	1813|1814|1815|5142	DRD2|DRD3|DRD4|PDE4B	-2.8503	0	43	0	0	-14.4566	82.6284153
1	-3.91602	GO:0007212	M1	1	0	GO Biological Processes	19	dopamine receptor signaling pathway	-3.91602	15.73875	7.457926	30242	42	183	4	2.185792	1.080886	1812|1813|1814|1815	DRD1|DRD2|DRD3|DRD4	-2.81521	0	43	0	0	-14.4566	82.6284153
1	-3.72127	GO:0032413	M1	1	0	GO Biological Processes	19	negative regulation of ion transmembrane transporter activity	-3.72127	9.497519	6.193105	30242	87	183	5	2.73224	1.205087	150|1813|1814|1815|4318	ADRA2A|DRD2|DRD3|DRD4|MMP9	-2.64322	0	43	0	0	-14.4566	82.6284153
1	-3.68815	GO:1903170	M1	1	0	GO Biological Processes	19	negative regulation of calcium ion transmembrane transport	-3.68815	13.7714	6.909319	30242	48	183	4	2.185792	1.080886	150|1813|1814|1815	ADRA2A|DRD2|DRD3|DRD4	-2.6157	0	43	0	0	-14.4566	82.6284153
1	-3.63764	GO:1901386	M1	1	0	GO Biological Processes	19	negative regulation of voltage-gated calcium channel activity	-3.63764	24.78852	8.303425	30242	20	183	3	1.639344	0.938686	1813|1814|1815	DRD2|DRD3|DRD4	-2.56974	0	43	0	0	-14.4566	82.6284153
1	-3.31368	GO:2001258	M1	1	0	GO Biological Processes	19	negative regulation of cation channel activity	-3.31368	11.01712	6.060116	30242	60	183	4	2.185792	1.080886	1813|1814|1815|4318	DRD2|DRD3|DRD4|MMP9	-2.27727	0	43	0	0	-14.4566	82.6284153
1	-3.2497	GO:0032410	M1	1	0	GO Biological Processes	19	negative regulation of transporter activity	-3.2497	7.511674	5.338403	30242	110	183	5	2.73224	1.205087	150|1813|1814|1815|4318	ADRA2A|DRD2|DRD3|DRD4|MMP9	-2.21986	0	43	0	0	-14.4566	82.6284153
1	-3.10726	GO:1902305	M1	1	0	GO Biological Processes	19	regulation of sodium ion transmembrane transport	-3.10726	9.72099	5.617462	30242	68	183	4	2.185792	1.080886	1814|1815|4842|6331	DRD3|DRD4|NOS1|SCN5A	-2.09198	0	43	0	0	-14.4566	82.6284153
1	-2.90759	GO:0010765	M1	1	0	GO Biological Processes	19	positive regulation of sodium ion transport	-2.90759	14.16487	6.080415	30242	35	183	3	1.639344	0.938686	1815|4842|6331	DRD4|NOS1|SCN5A	-1.91274	0	43	0	0	-14.4566	82.6284153
1	-2.41178	GO:0035176	M1	1	0	GO Biological Processes	19	social behavior	-2.41178	9.534048	4.805754	30242	52	183	3	1.639344	0.938686	1814|1815|6532	DRD3|DRD4|SLC6A4	-1.4684	0	43	0	0	-14.4566	82.6284153

1	-2.36548	GO:0051703	M1	1	0	GO Biological Processes	19	biological process involved in intraspecies interaction between organisms	-2.36548	9.180935	4.694821	30242	54	183	3	1.639344	0.938686	1814|1815|6532	DRD3|DRD4|SLC6A4	-1.4267	0	43	0	0	-14.4566	82.6284153
1	-13.8887	GO:0014065	M1	1	0	GO Biological Processes	19	phosphatidylinositol 3-kinase signaling	-13.8887	17.21425	15.21758	30242	144	183	15	8.196721	2.027793	1956|2147|2149|2335|3356|3357|3643|4914|4915|5159|5290|5291|5293|5467|7124	EGFR|F2|F2R|FN1|HTR2A|HTR2B|INSR|NTRK1|NTRK2|PDGFRB|PIK3CA|PIK3CB|PIK3CD|PPARD|TNF	-11.9576	0	44	1	1	-13.8887	18.82672754
1	-12.6094	GO:0043491	M1	1	0	GO Biological Processes	19	protein kinase B signaling	-12.6094	12.53132	13.11511	30242	211	183	16	8.743169	2.088053	1813|1814|1956|2155|3643|4914|4915|5290|5291|5293|5465|5979|6347|7040|7046|7124	DRD2|DRD3|EGFR|F7|INSR|NTRK1|NTRK2|PIK3CA|PIK3CB|PIK3CD|PPARA|RET|CCL2|TGFB1|TGFBR1|TNF	-10.7499	0	44	0	0	-13.8887	18.82672754
1	-12.5207	GO:0048015	M1	1	0	GO Biological Processes	19	phosphatidylinositol-mediated signaling	-12.5207	13.92614	13.49559	30242	178	183	15	8.196721	2.027793	1956|2147|2149|2335|3356|3357|3643|4914|4915|5159|5290|5291|5293|5467|7124	EGFR|F2|F2R|FN1|HTR2A|HTR2B|INSR|NTRK1|NTRK2|PDGFRB|PIK3CA|PIK3CB|PIK3CD|PPARD|TNF	-10.6671	0	44	0	0	-13.8887	18.82672754
1	-12.379	GO:0048017	M1	1	0	GO Biological Processes	19	inositol lipid-mediated signaling	-12.379	13.62007	13.32414	30242	182	183	15	8.196721	2.027793	1956|2147|2149|2335|3356|3357|3643|4914|4915|5159|5290|5291|5293|5467|7124	EGFR|F2|F2R|FN1|HTR2A|HTR2B|INSR|NTRK1|NTRK2|PDGFRB|PIK3CA|PIK3CB|PIK3CD|PPARD|TNF	-10.5294	0	44	0	0	-13.8887	18.82672754
1	-12.2751	GO:0051896	M1	1	0	GO Biological Processes	19	regulation of protein kinase B signaling	-12.2751	13.3992	13.19906	30242	185	183	15	8.196721	2.027793	1813|1814|1956|2155|3643|4914|4915|5290|5291|5293|5465|5979|7040|7046|7124	DRD2|DRD3|EGFR|F7|INSR|NTRK1|NTRK2|PIK3CA|PIK3CB|PIK3CD|PPARA|RET|TGFB1|TGFBR1|TNF	-10.437	0	44	0	0	-13.8887	18.82672754
1	-12.0602	GO:0007169	M1	1	0	GO Biological Processes	19	transmembrane receptor protein tyrosine kinase signaling pathway	-12.0602	6.295498	10.48044	30242	630	183	24	13.11475	2.495327	134|150|154|367|624|673|836|1956|2050|2155|2263|3643|4313|4318|4914|4915|5159|5290|5291|5293|5970|5979|7040|10135	ADORA1|ADRA2A|ADRB2|AR|BDKRB2|BRAF|CASP3|EGFR|EPHB4|F7|FGFR2|INSR|MMP2|MMP9|NTRK1|NTRK2|PDGFRB|PIK3CA|PIK3CB|PIK3CD|RELA|RET|TGFB1|NAMPT	-10.2334	0	44	0	0	-13.8887	18.82672754
1	-8.89399	GO:0014068	M1	1	0	GO Biological Processes	19	positive regulation of phosphatidylinositol 3-kinase signaling	-8.89399	18.82673	12.37896	30242	79	183	9	4.918033	1.598524	2147|2149|2335|3643|4914|4915|5159|5467|7124	F2|F2R|FN1|INSR|NTRK1|NTRK2|PDGFRB|PPARD|TNF	-7.28683	0	44	0	0	-13.8887	18.82672754
1	-8.8061	GO:0014066	M1	1	0	GO Biological Processes	19	regulation of phosphatidylinositol 3-kinase signaling	-8.8061	14.888	11.43749	30242	111	183	10	5.464481	1.680144	1956|2147|2149|2335|3643|4914|4915|5159|5467|7124	EGFR|F2|F2R|FN1|INSR|NTRK1|NTRK2|PDGFRB|PPARD|TNF	-7.20451	0	44	0	0	-13.8887	18.82672754
1	-8.4739	GO:0051897	M1	1	0	GO Biological Processes	19	positive regulation of protein kinase B signaling	-8.4739	13.7714	10.93764	30242	120	183	10	5.464481	1.680144	1956|2155|3643|5290|5291|5293|5979|7040|7046|7124	EGFR|F7|INSR|PIK3CA|PIK3CB|PIK3CD|RET|TGFB1|TGFBR1|TNF	-6.89386	0	44	0	0	-13.8887	18.82672754
1	-13.8364	GO:0032102	M1	1	0	GO Biological Processes	19	negative regulation of response to external stimulus	-13.8364	8.65631	12.32855	30242	420	183	22	12.02186	2.404073	100|134|135|1812|1813|1814|1991|2147|3156|3558|4846|5465|5467|54	ADA|ADORA1|ADORA2A|DRD1|DRD2|DRD3|ELANE|F2|HMGC	-11.9099	0	45	1	1	-13.8364	11.26751118

<table>
<tr><td></td><td></td><td></td><td></td><td></td><td></td><td></td><td></td><td></td><td></td><td></td><td></td><td></td><td></td><td></td><td></td><td></td><td></td><td></td><td></td><td></td><td></td><td></td><td></td><td></td><td></td><td></td></tr>
<tr><td></td><td></td><td></td><td></td><td></td><td></td><td></td><td></td><td></td><td></td><td></td><td></td><td></td><td></td><td></td><td></td><td></td><td></td><td>68|5734|6347|6531|7124|7132|10203|23621|114548</td><td>R|IL2|NOS3|PPARA|PPARD|PPARG|PTGER4|CCL2|SLC6A3|TNF|TNFRSF1A|CALCRL|BACE1|NLRP3</td><td></td><td></td><td></td><td></td><td></td><td></td><td></td></tr>
<tr><td>1</td><td>-9.05051</td><td>GO:0050728</td><td>M1</td><td>1</td><td>0</td><td>GO Biological Processes</td><td>19</td><td>negative regulation of inflammatory response</td><td>-9.05051</td><td>11.26751</td><td>10.65909</td><td>30242</td><td>176</td><td>183</td><td>12</td><td>6.557377</td><td>1.829835</td><td>100|134|135|1991|3558|5465|5467|5468|5734|7132|10203|114548</td><td>ADA|ADORA1|ADORA2A|ELANE|IL2|PPARA|PPARD|PPARG|PTGER4|TNFRSF1A|CALCRL|NLRP3</td><td>-7.43087</td><td>0</td><td>45</td><td>0</td><td>0</td><td>-13.8364</td><td>11.26751118</td></tr>
<tr><td>1</td><td>-8.61383</td><td>GO:0040013</td><td>M1</td><td>1</td><td>0</td><td>GO Biological Processes</td><td>19</td><td>negative regulation of locomotion</td><td>-8.61383</td><td>6.762428</td><td>8.948511</td><td>30242</td><td>391</td><td>183</td><td>16</td><td>8.743169</td><td>2.088053</td><td>100|134|135|140|328|596|1545|1813|1991|5467|5468|5734|6347|7040|7124|225689</td><td>ADA|ADORA1|ADORA2A|ADORA3|APEX1|BCL2|CYP1B1|DRD2|ELANE|PPARD|PPARG|PTGER4|CCL2|TGFB1|TNF|MAPK15</td><td>-7.02315</td><td>0</td><td>45</td><td>0</td><td>0</td><td>-13.8364</td><td>11.26751118</td></tr>
<tr><td>1</td><td>-8.15837</td><td>GO:0031348</td><td>M1</td><td>1</td><td>0</td><td>GO Biological Processes</td><td>19</td><td>negative regulation of defense response</td><td>-8.15837</td><td>8.326895</td><td>9.22192</td><td>30242</td><td>258</td><td>183</td><td>13</td><td>7.103825</td><td>1.898976</td><td>100|134|135|1813|1991|3558|5465|5467|5468|5734|7132|10203|114548</td><td>ADA|ADORA1|ADORA2A|DRD2|ELANE|IL2|PPARA|PPARD|PPARG|PTGER4|TNFRSF1A|CALCRL|NLRP3</td><td>-6.60485</td><td>0</td><td>45</td><td>0</td><td>0</td><td>-13.8364</td><td>11.26751118</td></tr>
<tr><td>1</td><td>-7.97305</td><td>GO:0002683</td><td>M1</td><td>1</td><td>0</td><td>GO Biological Processes</td><td>19</td><td>negative regulation of immune system process</td><td>-7.97305</td><td>6.092418</td><td>8.337557</td><td>30242</td><td>434</td><td>183</td><td>16</td><td>8.743169</td><td>2.088053</td><td>100|134|135|472|836|1268|1813|3558|5468|5591|5734|6347|7040|7099|7124|729230</td><td>ADA|ADORA1|ADORA2A|ATM|CASP3|CNR1|DRD2|IL2|PPARG|PRKDC|PTGER4|CCL2|TGFB1|TLR4|TNF|CCR2</td><td>-6.43983</td><td>0</td><td>45</td><td>0</td><td>0</td><td>-13.8364</td><td>11.26751118</td></tr>
<tr><td>1</td><td>-7.56992</td><td>GO:0030336</td><td>M1</td><td>1</td><td>0</td><td>GO Biological Processes</td><td>19</td><td>negative regulation of cell migration</td><td>-7.56992</td><td>6.725569</td><td>8.333226</td><td>30242</td><td>344</td><td>183</td><td>14</td><td>7.650273</td><td>1.964856</td><td>100|134|140|328|596|1545|1813|5467|5468|5734|6347|7040|7124|225689</td><td>ADA|ADORA1|ADORA3|APEX1|BCL2|CYP1B1|DRD2|PPARD|PPARG|PTGER4|CCL2|TGFB1|TNF|MAPK15</td><td>-6.07723</td><td>0</td><td>45</td><td>0</td><td>0</td><td>-13.8364</td><td>11.26751118</td></tr>
<tr><td>1</td><td>-7.33963</td><td>GO:2000146</td><td>M1</td><td>1</td><td>0</td><td>GO Biological Processes</td><td>19</td><td>negative regulation of cell motility</td><td>-7.33963</td><td>6.444556</td><td>8.097183</td><td>30242</td><td>359</td><td>183</td><td>14</td><td>7.650273</td><td>1.964856</td><td>100|134|140|328|596|1545|1813|5467|5468|5734|6347|7040|7124|225689</td><td>ADA|ADORA1|ADORA3|APEX1|BCL2|CYP1B1|DRD2|PPARD|PPARG|PTGER4|CCL2|TGFB1|TNF|MAPK15</td><td>-5.87037</td><td>0</td><td>45</td><td>0</td><td>0</td><td>-13.8364</td><td>11.26751118</td></tr>
<tr><td>1</td><td>-7.22137</td><td>GO:0051271</td><td>M1</td><td>1</td><td>0</td><td>GO Biological Processes</td><td>19</td><td>negative regulation of cellular component movement</td><td>-7.22137</td><td>6.304075</td><td>7.976734</td><td>30242</td><td>367</td><td>183</td><td>14</td><td>7.650273</td><td>1.964856</td><td>100|134|140|328|596|1545|1813|5467|5468|5734|6347|7040|7124|225689</td><td>ADA|ADORA1|ADORA3|APEX1|BCL2|CYP1B1|DRD2|PPARD|PPARG|PTGER4|CCL2|TGFB1|TNF|MAPK15</td><td>-5.76811</td><td>0</td><td>45</td><td>0</td><td>0</td><td>-13.8364</td><td>11.26751118</td></tr>
<tr><td>1</td><td>-13.5517</td><td>GO:1901615</td><td>M1</td><td>1</td><td>0</td><td>GO Biological Processes</td><td>19</td><td>organic hydroxy compound metabolic process</td><td>-13.5517</td><td>7.38578</td><td>11.64991</td><td>30242</td><td>537</td><td>183</td><td>24</td><td>13.11475</td><td>2.495327</td><td>217|351|427|596|1080|1543|1544|1545|1588|1645|1812|1813|1814|1815|2903|3156|3350|4128|4129|5444|5467|6531|7124|7157</td><td>ALDH2|APP|ASAH1|BCL2|CFTR|CYP1A1|CYP1A2|CYP1B1|CYP19A1|AKR1C1|DRD1|DRD2|DRD3|DRD4|GRIN2A|HMGCR|HTR1A|MAOA|MAOB|PON1|PPARD|SLC6A3|TNF|TP53</td><td>-11.6345</td><td>0</td><td>46</td><td>1</td><td>1</td><td>-13.5517</td><td>8.640880031</td></tr>
</table>

<table>
<tr><td>1</td><td>-9.89537</td><td>GO:0008202</td><td>M1</td><td>1</td><td>0</td><td>GO Biological Processes</td><td>19</td><td>steroid metabolic process</td><td>-9.89537</td><td>8.288744</td><td>10.21132</td><td>30242</td><td>319</td><td>183</td><td>16</td><td>8.743169</td><td>2.088053</td><td>185|351|427|1080|1543|1544|1545|1557|1588|1645|2099|3156|5444|5467|7124|7364</td><td>AGTR1|APP|ASAH1|CFTR|CYP1A1|CYP1A2|CYP1B1|CYP2C19|CYP19A1|AKR1C1|ESR1|HMGCR|PON1|PPARD|TNF|UGT2B7</td><td>-8.22183</td><td>0</td><td>46</td><td>0</td><td>0</td><td>-13.5517</td><td>8.640880031</td></tr>
<tr><td>1</td><td>-7.29378</td><td>GO:0008610</td><td>M1</td><td>1</td><td>0</td><td>GO Biological Processes</td><td>19</td><td>lipid biosynthetic process</td><td>-7.29378</td><td>4.524322</td><td>7.328712</td><td>30242</td><td>694</td><td>183</td><td>19</td><td>10.38251</td><td>2.254873</td><td>427|472|1080|1543|1544|1588|3156|3356|3357|3358|5290|5291|5293|5465|5467|5742|5743|6916|7124</td><td>ASAH1|ATM|CFTR|CYP1A1|CYP1A2|CYP19A1|HMGCR|HTR2A|HTR2B|HTR2C|PIK3CA|PIK3CB|PIK3CD|PPARA|PPARD|PTGS1|PTGS2|TBXAS1|TNF</td><td>-5.83099</td><td>0</td><td>46</td><td>0</td><td>0</td><td>-13.5517</td><td>8.640880031</td></tr>
<tr><td>1</td><td>-5.34499</td><td>GO:0016125</td><td>M1</td><td>1</td><td>0</td><td>GO Biological Processes</td><td>19</td><td>sterol metabolic process</td><td>-5.34499</td><td>8.64088</td><td>7.393015</td><td>30242</td><td>153</td><td>183</td><td>8</td><td>4.371585</td><td>1.511428</td><td>351|1080|1544|1545|1588|3156|5444|5467</td><td>APP|CFTR|CYP1A2|CYP1B1|CYP19A1|HMGCR|PON1|PPARD</td><td>-4.06966</td><td>0</td><td>46</td><td>0</td><td>0</td><td>-13.5517</td><td>8.640880031</td></tr>
<tr><td>1</td><td>-4.95141</td><td>GO:0006066</td><td>M1</td><td>1</td><td>0</td><td>GO Biological Processes</td><td>19</td><td>alcohol metabolic process</td><td>-4.95141</td><td>5.149646</td><td>6.118981</td><td>30242</td><td>353</td><td>183</td><td>11</td><td>6.010929</td><td>1.757049</td><td>217|351|427|1080|1543|1544|1545|1645|3156|5444|5467</td><td>ALDH2|APP|ASAH1|CFTR|CYP1A1|CYP1A2|CYP1B1|AKR1C1|HMGCR|PON1|PPARD</td><td>-3.7225</td><td>0</td><td>46</td><td>0</td><td>0</td><td>-13.5517</td><td>8.640880031</td></tr>
<tr><td>1</td><td>-3.69744</td><td>GO:0008203</td><td>M1</td><td>1</td><td>0</td><td>GO Biological Processes</td><td>19</td><td>cholesterol metabolic process</td><td>-3.69744</td><td>7.18508</td><td>5.682103</td><td>30242</td><td>138</td><td>183</td><td>6</td><td>3.278689</td><td>1.316393</td><td>351|1080|1544|3156|5444|5467</td><td>APP|CFTR|CYP1A2|HMGCR|PON1|PPARD</td><td>-2.62334</td><td>0</td><td>46</td><td>0</td><td>0</td><td>-13.5517</td><td>8.640880031</td></tr>
<tr><td>1</td><td>-3.53346</td><td>GO:1902652</td><td>M1</td><td>1</td><td>0</td><td>GO Biological Processes</td><td>19</td><td>secondary alcohol metabolic process</td><td>-3.53346</td><td>6.699601</td><td>5.423401</td><td>30242</td><td>148</td><td>183</td><td>6</td><td>3.278689</td><td>1.316393</td><td>351|1080|1544|3156|5444|5467</td><td>APP|CFTR|CYP1A2|HMGCR|PON1|PPARD</td><td>-2.47452</td><td>0</td><td>46</td><td>0</td><td>0</td><td>-13.5517</td><td>8.640880031</td></tr>
<tr><td>1</td><td>-3.22058</td><td>GO:0044283</td><td>M1</td><td>1</td><td>0</td><td>GO Biological Processes</td><td>19</td><td>small molecule biosynthetic process</td><td>-3.22058</td><td>3.275361</td><td>4.221336</td><td>30242</td><td>555</td><td>183</td><td>11</td><td>6.010929</td><td>1.757049</td><td>100|427|1080|1543|1544|3156|5465|5742|5743|6916|7124</td><td>ADA|ASAH1|CFTR|CYP1A1|CYP1A2|HMGCR|PPARA|PTGS1|PTGS2|TBXAS1|TNF</td><td>-2.19339</td><td>0</td><td>46</td><td>0</td><td>0</td><td>-13.5517</td><td>8.640880031</td></tr>
<tr><td>1</td><td>-3.16106</td><td>GO:0006694</td><td>M1</td><td>1</td><td>0</td><td>GO Biological Processes</td><td>19</td><td>steroid biosynthetic process</td><td>-3.16106</td><td>5.698511</td><td>4.849746</td><td>30242</td><td>174</td><td>183</td><td>6</td><td>3.278689</td><td>1.316393</td><td>427|1080|1543|1588|3156|7124</td><td>ASAH1|CFTR|CYP1A1|CYP19A1|HMGCR|TNF</td><td>-2.14088</td><td>0</td><td>46</td><td>0</td><td>0</td><td>-13.5517</td><td>8.640880031</td></tr>
<tr><td>1</td><td>-13.5346</td><td>GO:0051345</td><td>M1</td><td>1</td><td>0</td><td>GO Biological Processes</td><td>19</td><td>positive regulation of hydrolase activity</td><td>-13.5346</td><td>6.966983</td><td>11.45021</td><td>30242</td><td>593</td><td>183</td><td>25</td><td>13.6612</td><td>2.538762</td><td>148|153|185|351|834|1909|1956|2099|2149|2263|2335|2902|2903|2904|3356|3357|3383|4914|5159|5468|6347|6401|7124|9475|114548</td><td>ADRA1A|ADRB1|AGTR1|APP|CASP1|EDNRA|EGFR|ESR1|F2R|FGFR2|FN1|GRIN1|GRIN2A|GRIN2B|HTR2A|HTR2B|ICAM1|NTRK1|PDGFRB|PPARG|CCL2|SELE|TNF|ROCK2|NLRP3</td><td>-11.6197</td><td>0</td><td>47</td><td>1</td><td>1</td><td>-13.5346</td><td>33.05136612</td></tr>
<tr><td>1</td><td>-11.8423</td><td>GO:0010518</td><td>M1</td><td>1</td><td>0</td><td>GO Biological Processes</td><td>19</td><td>positive regulation of phospholipase activity</td><td>-11.8423</td><td>29.51015</td><td>16.662</td><td>30242</td><td>56</td><td>183</td><td>10</td><td>5.464481</td><td>1.680144</td><td>148|185|1909|1956|2099|2263|3356|3357|5159|6401</td><td>ADRA1A|AGTR1|EDNRA|EGFR|ESR1|FGFR2|HTR2A|HTR2B|PDGFRB|SELE</td><td>-10.0283</td><td>0</td><td>47</td><td>0</td><td>0</td><td>-13.5346</td><td>33.05136612</td></tr>
<tr><td>1</td><td>-10.9574</td><td>GO:0010517</td><td>M1</td><td>1</td><td>0</td><td>GO Biological Processes</td><td>19</td><td>regulation of phospholipase activity</td><td>-10.9574</td><td>24.30248</td><td>15.00985</td><td>30242</td><td>68</td><td>183</td><td>10</td><td>5.464481</td><td>1.680144</td><td>148|185|1909|1956|2099|2263|3356|3357|5159|6401</td><td>ADRA1A|AGTR1|EDNRA|EGFR|ESR1|FGFR2|HTR2A|HTR2B|PDGFRB|SELE</td><td>-9.20707</td><td>0</td><td>47</td><td>0</td><td>0</td><td>-13.5346</td><td>33.05136612</td></tr>
<tr><td>1</td><td>-10.8917</td><td>GO:0060193</td><td>M1</td><td>1</td><td>0</td><td>GO Biological Processes</td><td>19</td><td>positive regulation of lipase activity</td><td>-10.8917</td><td>23.95027</td><td>14.89153</td><td>30242</td><td>69</td><td>183</td><td>10</td><td>5.464481</td><td>1.680144</td><td>148|185|1909|1956|2099|2263|3356|3357|5159|</td><td>ADRA1A|AGTR1|EDNRA|EGFR|ESR1|FGF</td><td>-9.14443</td><td>0</td><td>47</td><td>0</td><td>0</td><td>-13.5346</td><td>33.05136612</td></tr>
</table>

																		6401	R2|HTR2A|HTR2B|PDGFRB|SELE							
1	-9.99794	GO:0010863	M1	1	0	GO Biological Processes	19	positive regulation of phospholipase C activity	-9.99794	33.05137	15.82687	30242	40	183	8	4.371585	1.511428	148|1909|1956|2099|3356|3357|5159|6401	ADRA1A|EDNRA|EGFR|ESR1|HTR2A|HTR2B|PDGFRB|SELE	-8.31116	0	47	0	0	-13.5346	33.05136612
1	-9.72922	GO:1900274	M1	1	0	GO Biological Processes	19	regulation of phospholipase C activity	-9.72922	30.74546	15.22983	30242	43	183	8	4.371585	1.511428	148|1909|1956|2099|3356|3357|5159|6401	ADRA1A|EDNRA|EGFR|ESR1|HTR2A|HTR2B|PDGFRB|SELE	-8.06853	0	47	0	0	-13.5346	33.05136612
1	-9.6173	GO:0060191	M1	1	0	GO Biological Processes	19	regulation of lipase activity	-9.6173	17.9627	12.71398	30242	92	183	10	5.464481	1.680144	148|185|1909|1956|2099|2263|3356|3357|5159|6401	ADRA1A|AGTR1|EDNRA|EGFR|ESR1|FGFR2|HTR2A|HTR2B|PDGFRB|SELE	-7.96414	0	47	0	0	-13.5346	33.05136612
1	-7.62346	GO:0007202	M1	1	0	GO Biological Processes	19	activation of phospholipase C activity	-7.62346	33.05137	13.7042	30242	30	183	6	3.278689	1.316393	148|1909|1956|3356|3357|6401	ADRA1A|EDNRA|EGFR|HTR2A|HTR2B|SELE	-6.12558	0	47	0	0	-13.5346	33.05136612
1	-13.4308	GO:0006809	M1	1	0	GO Biological Processes	19	nitric oxide biosynthetic process	-13.4308	26.09318	17.08992	30242	76	183	12	6.557377	1.829835	1545|1728|3383|3643|4842|4843|4846|5743|7099|7124|7442|9475	CYP1B1|NQO1|ICAM1|INSR|NOS1|NOS2|NOS3|PTGS2|TLR4|TNF|TRPV1|ROCK2	-11.5226	0	48	1	1	-13.4308	75.11674118
1	-13.0843	GO:0046209	M1	1	0	GO Biological Processes	19	nitric oxide metabolic process	-13.0843	24.48249	16.51202	30242	81	183	12	6.557377	1.829835	1545|1728|3383|3643|4842|4843|4846|5743|7099|7124|7442|9475	CYP1B1|NQO1|ICAM1|INSR|NOS1|NOS2|NOS3|PTGS2|TLR4|TNF|TRPV1|ROCK2	-11.1915	0	48	0	0	-13.4308	75.11674118
1	-13.0178	GO:2001057	M1	1	0	GO Biological Processes	19	reactive nitrogen species metabolic process	-13.0178	24.18393	16.40267	30242	82	183	12	6.557377	1.829835	1545|1728|3383|3643|4842|4843|4846|5743|7099|7124|7442|9475	CYP1B1|NQO1|ICAM1|INSR|NOS1|NOS2|NOS3|PTGS2|TLR4|TNF|TRPV1|ROCK2	-11.1272	0	48	0	0	-13.4308	75.11674118
1	-12.7184	GO:0002526	M1	1	0	GO Biological Processes	19	acute inflammatory response	-12.7184	19.1816	15.04104	30242	112	183	13	7.103825	1.898976	134|1268|1910|1991|2147|2335|3383|3569|4988|5743|7124|7442|114548	ADORA1|CNR1|EDNRB|ELANE|F2|FN1|ICAM1|IL6|OPRM1|PTGS2|TNF|TRPV1|NLRP3	-10.8446	0	48	0	0	-13.4308	75.11674118
1	-11.0989	GO:0031649	M1	1	0	GO Biological Processes	19	heat generation	-11.0989	64.26655	20.94936	30242	18	183	7	3.825137	1.417845	153|154|1268|1910|5743|7124|7442	ADRB1|ADRB2|CNR1|EDNRB|PTGS2|TNF|TRPV1	-9.33907	0	48	0	0	-13.4308	75.11674118
1	-9.24988	GO:0006953	M1	1	0	GO Biological Processes	19	acute-phase response	-9.24988	26.98071	14.20143	30242	49	183	8	4.371585	1.511428	1268|1910|2147|2335|3569|5743|7124|7442	CNR1|EDNRB|F2|FN1|IL6|PTGS2|TNF|TRPV1	-7.61856	0	48	0	0	-13.4308	75.11674118
1	-9.10749	GO:0001659	M1	1	0	GO Biological Processes	19	temperature homeostasis	-9.10749	11.39702	10.73168	30242	174	183	12	6.557377	1.829835	134|153|154|1268|1813|1910|3356|5743|7099|7124|7442|729230	ADORA1|ADRB1|ADRB2|CNR1|DRD2|EDNRB|HTR2A|PTGS2|TLR4|TNF|TRPV1|CCR2	-7.47971	0	48	0	0	-13.4308	75.11674118
1	-8.46275	GO:0001660	M1	1	0	GO Biological Processes	19	fever generation	-8.46275	75.11674	19.18328	30242	11	183	5	2.73224	1.205087	1268|1910|5743|7124|7442	CNR1|EDNRB|PTGS2|TNF|TRPV1	-6.88741	0	48	0	0	-13.4308	75.11674118
1	-7.80103	GO:0002673	M1	1	0	GO Biological Processes	19	regulation of acute inflammatory response	-7.80103	24.09995	12.49706	30242	48	183	7	3.825137	1.417845	134|1268|1910|3569|5743|7124|114548	ADORA1|CNR1|EDNRB|IL6|PTGS2|TNF|NLRP3	-6.28538	0	48	0	0	-13.4308	75.11674118
1	-7.00726	GO:0045428	M1	1	0	GO Biological Processes	19	regulation of nitric oxide biosynthetic process	-7.00726	18.65803	10.85962	30242	62	183	7	3.825137	1.417845	3383|3643|5743|7099|7124|7442|9475	ICAM1|INSR|PTGS2|TLR4|TNF|TRPV1|ROCK2	-5.5715	0	48	0	0	-13.4308	75.11674118

1	-6.91023	GO:0080164	M1	1	0	GO Biological Processes	19	regulation of nitric oxide metabolic process	-6.91023	18.07497	10.66942	30242	64	183	7	3.825137	1.417845	3383|3643|5743|7099|7124|7442|9475	ICAM1|INSR|PTGS2|TLR4|TNF|TRPV1|ROCK2	-5.4814	0	48	0	0	-13.4308	75.11674118
1	-6.83474	GO:0045429	M1	1	0	GO Biological Processes	19	positive regulation of nitric oxide biosynthetic process	-6.83474	24.78852	11.7467	30242	40	183	6	3.278689	1.316393	3383|3643|5743|7099|7124|7442	ICAM1|INSR|PTGS2|TLR4|TNF|TRPV1	-5.41254	0	48	0	0	-13.4308	75.11674118
1	-6.79682	GO:0031620	M1	1	0	GO Biological Processes	19	regulation of fever generation	-6.79682	73.44748	16.96056	30242	9	183	4	2.185792	1.080886	1268|1910|5743|7124	CNR1|EDNRB|PTGS2|TNF	-5.37898	0	48	0	0	-13.4308	75.11674118
1	-6.7682	GO:1904407	M1	1	0	GO Biological Processes	19	positive regulation of nitric oxide metabolic process	-6.7682	24.18393	11.59057	30242	41	183	6	3.278689	1.316393	3383|3643|5743|7099|7124|7442	ICAM1|INSR|PTGS2|TLR4|TNF|TRPV1	-5.35397	0	48	0	0	-13.4308	75.11674118
1	-5.90705	GO:0031650	M1	1	0	GO Biological Processes	19	regulation of heat generation	-5.90705	47.21624	13.49554	30242	14	183	4	2.185792	1.080886	1268|1910|5743|7124	CNR1|EDNRB|PTGS2|TNF	-4.56779	0	48	0	0	-13.4308	75.11674118
1	-5.37096	GO:0150077	M1	1	0	GO Biological Processes	19	regulation of neuroinflammatory response	-5.37096	20.6571	9.70662	30242	40	183	5	2.73224	1.205087	3569|4314|4318|5743|7124	IL6|MMP3|MMP9|PTGS2|TNF	-4.09037	0	48	0	0	-13.4308	75.11674118
1	-5.16186	GO:0150076	M1	1	0	GO Biological Processes	19	neuroinflammatory response	-5.16186	18.77919	9.208427	30242	44	183	5	2.73224	1.205087	3569|4314|4318|5743|7124	IL6|MMP3|MMP9|PTGS2|TNF	-3.90937	0	48	0	0	-13.4308	75.11674118
1	-5.12529	GO:0031622	M1	1	0	GO Biological Processes	19	positive regulation of fever generation	-5.12529	70.82436	14.41575	30242	7	183	3	1.639344	0.938686	1268|5743|7124	CNR1|PTGS2|TNF	-3.87821	0	48	0	0	-13.4308	75.11674118
1	-4.62504	GO:0002675	M1	1	0	GO Biological Processes	19	positive regulation of acute inflammatory response	-4.62504	23.60812	9.338469	30242	28	183	4	2.185792	1.080886	1268|3569|5743|7124	CNR1|IL6|PTGS2|TNF	-3.43978	0	48	0	0	-13.4308	75.11674118
1	-4.45963	GO:0031652	M1	1	0	GO Biological Processes	19	positive regulation of heat generation	-4.45963	45.07004	11.40644	30242	11	183	3	1.639344	0.938686	1268|5743|7124	CNR1|PTGS2|TNF	-3.30278	0	48	0	0	-13.4308	75.11674118
1	-3.94533	GO:0043154	M1	1	0	GO Biological Processes	19	negative regulation of cysteine-type endopeptidase activity involved in apoptotic process	-3.94533	10.59339	6.619286	30242	78	183	5	2.73224	1.205087	135|4318|5743|6197|7124	ADORA2A|MMP9|PTGS2|RPS6KA3|TNF	-2.8389	0	48	0	0	-13.4308	75.11674118
1	-3.74486	GO:2000117	M1	1	0	GO Biological Processes	19	negative regulation of cysteine-type endopeptidase activity	-3.74486	9.607955	6.23733	30242	86	183	5	2.73224	1.205087	135|4318|5743|6197|7124	ADORA2A|MMP9|PTGS2|RPS6KA3|TNF	-2.66416	0	48	0	0	-13.4308	75.11674118
1	-3.51421	GO:0045861	M1	1	0	GO Biological Processes	19	negative regulation of proteolysis	-3.51421	4.237355	4.760033	30242	351	183	9	4.918033	1.598524	135|351|2147|4318|5582|5743|6197|7124|7157	ADORA2A|APP|F2|MMP9|PRKCG|PTGS2|RPS6KA3|TNF|TP53	-2.45684	0	48	0	0	-13.4308	75.11674118
1	-3.27331	GO:0051346	M1	1	0	GO Biological Processes	19	negative regulation of hydrolase activity	-3.27331	3.924305	4.470045	30242	379	183	9	4.918033	1.598524	135|351|3551|4318|4842|5743|6197|7124|9475	ADORA2A|APP|IKBKB|MMP9|NOS1|PTGS2|RPS6KA3|TNF|ROCK2	-2.24197	0	48	0	0	-13.4308	75.11674118
1	-2.35142	GO:0010951	M1	1	0	GO Biological Processes	19	negative regulation of endopeptidase activity	-2.35142	3.934686	3.650145	30242	252	183	6	3.278689	1.316393	135|351|4318|5743|6197|7124	ADORA2A|APP|MMP9|PTGS2|RPS6KA3|TNF	-1.4136	0	48	0	0	-13.4308	75.11674118
1	-2.27036	GO:0010466	M1	1	0	GO Biological Processes	19	negative regulation of peptidase activity	-2.27036	3.784508	3.531991	30242	262	183	6	3.278689	1.316393	135|351|4318|5743|6197|7124	ADORA2A|APP|MMP9|PTGS2|RPS6KA3|TNF	-1.33896	0	48	0	0	-13.4308	75.11674118
1	-12.7953	GO:0097190	M1	1	0	GO Biological Processes	19	apoptotic signaling pathway	-12.7953	6.814715	11.052	30242	582	183	24	13.11475	2.495327	134|135|367|472|596|624|834|836|1545|3383|3558|4318|4846|5467|5591|5743|5970|5979|6326|7040|7046|7124|7132|7	ADORA1|ADORA2A|AR|ATM|BCL2|BDKRB2|CASP1|CASP3|CYP1B1|ICAM1|IL2|MMP9|NOS3|PPARD|PRK	-10.9169	0	49	1	1	-12.7953	11.92575066

																		157	DC|PTGS2|RELA|RET|SCN2A|TGFB1|TGFBR1|TNF|TNFRSF1A|TP53							
1	-6.97114	GO:0097191	M1	1	0	GO Biological Processes	19	extrinsic apoptotic signaling pathway	-6.97114	8.300571	8.460348	30242	219	183	11	6.010929	1.757049	367|596|3383|3558|4846|5970|5979|7040|7046|7124|7132	AR|BCL2|ICAM1|IL2|NOS3|RELA|RET|TGFB1|TGFBR1|TNF|TNFRSF1A	-5.53708	0	49	0	0	-12.7953	11.92575066
1	-6.52629	GO:2001233	M1	1	0	GO Biological Processes	19	regulation of apoptotic signaling pathway	-6.52629	6.03466	7.455857	30242	356	183	13	7.103825	1.898976	135|367|596|624|3383|4318|4846|5743|5970|5979|7046|7124|7157	ADORA2A|AR|BCL2|BDKRB2|ICAM1|MMP9|NOS3|PTGS2|RELA|RET|TGFBR1|TNF|TP53	-5.1358	0	49	0	0	-12.7953	11.92575066
1	-5.91935	GO:2001234	M1	1	0	GO Biological Processes	19	negative regulation of apoptotic signaling pathway	-5.91935	7.377537	7.475194	30242	224	183	10	5.464481	1.680144	367|596|624|3383|4318|4846|5743|5970|7046|7124	AR|BCL2|BDKRB2|ICAM1|MMP9|NOS3|PTGS2|RELA|TGFBR1|TNF	-4.57767	0	49	0	0	-12.7953	11.92575066
1	-5.66759	GO:2001237	M1	1	0	GO Biological Processes	19	negative regulation of extrinsic apoptotic signaling pathway	-5.66759	11.92575	8.409417	30242	97	183	7	3.825137	1.417845	367|596|3383|4846|5970|7046|7124	AR|BCL2|ICAM1|NOS3|RELA|TGFBR1|TNF	-4.35145	0	49	0	0	-12.7953	11.92575066
1	-5.38734	GO:2001236	M1	1	0	GO Biological Processes	19	regulation of extrinsic apoptotic signaling pathway	-5.38734	8.755329	7.454298	30242	151	183	8	4.371585	1.511428	367|596|3383|4846|5970|5979|7046|7124	AR|BCL2|ICAM1|NOS3|RELA|RET|TGFBR1|TNF	-4.10252	0	49	0	0	-12.7953	11.92575066
1	-3.84238	GO:0008625	M1	1	0	GO Biological Processes	19	extrinsic apoptotic signaling pathway via death domain receptors	-3.84238	10.07664	6.421737	30242	82	183	5	2.73224	1.205087	596|3383|4846|7124|7132	BCL2|ICAM1|NOS3|TNF|TNFRSF1A	-2.74951	0	49	0	0	-12.7953	11.92575066
1	-12.7609	GO:0035094	M1	1	0	GO Biological Processes	19	response to nicotine	-12.7609	35.9254	18.49617	30242	46	183	10	5.464481	1.680144	596|836|1268|1813|3066|4914|5465|5970|6531|7124	BCL2|CASP3|CNR1|DRD2|HDAC2|NTRK1|PPARA|RELA|SLC6A3|TNF	-10.8851	0	50	1	1	-12.7609	35.92539796
1	-11.351	GO:0060284	M1	1	0	GO Biological Processes	19	regulation of cell development	-11.351	6.913134	10.42403	30242	502	183	21	11.47541	2.356083	596|1813|1814|1910|1956|2147|2335|3066|3551|3569|4915|4988|5591|5970|5979|7040|7124|7132|7157|7442|9475	BCL2|DRD2|DRD3|EDNRB|EGFR|F2|FN1|HDAC2|IKBKB|IL6|NTRK2|OPRM1|PRKDC|RELA|RET|TGFB1|TNF|TNFRSF1A|TP53|TRPV1|ROCK2	-9.56668	0	50	0	0	-12.7609	35.92539796
1	-9.26867	GO:0042063	M1	1	0	GO Biological Processes	19	gliogenesis	-9.26867	8.23539	9.843474	30242	301	183	15	8.196721	2.027793	135|351|1128|1814|1956|2147|3066|3569|4915|5970|6347|7040|7099|7124|729230	ADORA2A|APP|CHRM1|DRD3|EGFR|F2|HDAC2|IL6|NTRK2|RELA|CCL2|TGFB1|TLR4|TNF|CCR2	-7.63378	0	50	0	0	-12.7609	35.92539796
1	-7.84491	GO:0010001	M1	1	0	GO Biological Processes	19	glial cell differentiation	-7.84491	8.813698	9.17911	30242	225	183	12	6.557377	1.829835	135|351|1814|1956|2147|3066|3569|4915|5970|7040|7099|7124	ADORA2A|APP|DRD3|EGFR|F2|HDAC2|IL6|NTRK2|RELA|TGFB1|TLR4|TNF	-6.32299	0	50	0	0	-12.7609	35.92539796
1	-7.41742	GO:0010720	M1	1	0	GO Biological Processes	19	positive regulation of cell development	-7.41742	7.209191	8.404746	30242	298	183	13	7.103825	1.898976	596|1813|1956|2335|3066|3569|4915|4988|5591|5970|5979|7040|7124	BCL2|DRD2|EGFR|FN1|HDAC2|IL6|NTRK2|OPRM1|PRKDC|RELA|RET|TGFB1|TNF	-5.93745	0	50	0	0	-12.7609	35.92539796
1	-6.64856	GO:0014013	M1	1	0	GO Biological Processes	19	regulation of	-6.64856	12.83548	9.388074	30242	103	183	8	4.371585	1.511428	1814|1956|2147|3066|3	DRD3|EGFR|F2|HDA	-5.24637	0	50	0	0	-12.7609	35.92539796

						Processes		gliogenesis										569|5970|7040|7124	C2|IL6|RELA|TGFB1|TNF							
1	-6.41687	GO:0050767	M1	1	0	GO Biological Processes	19	regulation of neurogenesis	-6.41687	5.90203	7.341541	30242	364	183	13	7.103825	1.898976	1813|1814|1956|2147|2335|3066|3569|4915|4988|5970|7040|7124|7157	DRD2|DRD3|EGFR|F2|FN1|HDAC2|IL6|NTRK2|OPRM1|RELA|TGFB1|TNF|TP53	-5.0338	0	50	0	0	-12.7609	35.92539796
1	-6.23111	GO:0051960	M1	1	0	GO Biological Processes	19	regulation of nervous system development	-6.23111	5.222563	6.985758	30242	443	183	14	7.650273	1.964856	1813|1814|1956|2147|2335|3066|3569|4914|4915|4988|5970|7040|7124|7157	DRD2|DRD3|EGFR|F2|FN1|HDAC2|IL6|NTRK1|NTRK2|OPRM1|RELA|TGFB1|TNF|TP53	-4.8612	0	50	0	0	-12.7609	35.92539796
1	-6.12933	GO:0008544	M1	1	0	GO Biological Processes	19	epidermis development	-6.12933	6.120623	7.230451	30242	324	183	12	6.557377	1.829835	427|596|836|1956|2263|3066|5465|5467|5970|7124|7421|9475	ASAH1|BCL2|CASP3|EGFR|FGFR2|HDAC2|PPARA|PPARD|RELA|TNF|VDR|ROCK2	-4.77057	0	50	0	0	-12.7609	35.92539796
1	-6.03396	GO:0051962	M1	1	0	GO Biological Processes	19	positive regulation of nervous system development	-6.03396	6.683181	7.346304	30242	272	183	11	6.010929	1.757049	1813|1956|2335|3066|3569|4914|4915|4988|5970|7040|7124	DRD2|EGFR|FN1|HDAC2|IL6|NTRK1|NTRK2|OPRM1|RELA|TGFB1|TNF	-4.68431	0	50	0	0	-12.7609	35.92539796
1	-5.90186	GO:0050769	M1	1	0	GO Biological Processes	19	positive regulation of neurogenesis	-5.90186	7.344748	7.453467	30242	225	183	10	5.464481	1.680144	1813|1956|2335|3066|3569|4915|4988|5970|7040|7124	DRD2|EGFR|FN1|HDAC2|IL6|NTRK2|OPRM1|RELA|TGFB1|TNF	-4.56381	0	50	0	0	-12.7609	35.92539796
1	-5.51711	GO:0014015	M1	1	0	GO Biological Processes	19	positive regulation of gliogenesis	-5.51711	15.02335	8.898754	30242	66	183	6	3.278689	1.316393	1956|3066|3569|5970|7040|7124	EGFR|HDAC2|IL6|RELA|TGFB1|TNF	-4.216	0	50	0	0	-12.7609	35.92539796
1	-5.38218	GO:0042303	M1	1	0	GO Biological Processes	19	molting cycle	-5.38218	10.81119	7.932581	30242	107	183	7	3.825137	1.417845	596|1956|2263|3066|5743|5970|7124	BCL2|EGFR|FGFR2|HDAC2|PTGS2|RELA|TNF	-4.10001	0	50	0	0	-12.7609	35.92539796
1	-5.38218	GO:0042633	M1	1	0	GO Biological Processes	19	hair cycle	-5.38218	10.81119	7.932581	30242	107	183	7	3.825137	1.417845	596|1956|2263|3066|5743|5970|7124	BCL2|EGFR|FGFR2|HDAC2|PTGS2|RELA|TNF	-4.10001	0	50	0	0	-12.7609	35.92539796
1	-5.29696	GO:0043588	M1	1	0	GO Biological Processes	19	skin development	-5.29696	6.28353	6.714749	30242	263	183	10	5.464481	1.680144	427|596|836|1956|2263|3066|5970|7124|7421|9475	ASAH1|BCL2|CASP3|EGFR|FGFR2|HDAC2|RELA|TNF|VDR|ROCK2	-4.02834	0	50	0	0	-12.7609	35.92539796
1	-5.15752	GO:0045685	M1	1	0	GO Biological Processes	19	regulation of glial cell differentiation	-5.15752	13.04659	8.204431	30242	76	183	6	3.278689	1.316393	1814|2147|3066|3569|5970|7040	DRD3|F2|HDAC2|IL6|RELA|TGFB1	-3.90602	0	50	0	0	-12.7609	35.92539796
1	-4.9968	GO:0001942	M1	1	0	GO Biological Processes	19	hair follicle development	-4.9968	12.24125	7.904428	30242	81	183	6	3.278689	1.316393	596|1956|2263|3066|5970|7124	BCL2|EGFR|FGFR2|HDAC2|RELA|TNF	-3.76129	0	50	0	0	-12.7609	35.92539796
1	-4.90554	GO:0022404	M1	1	0	GO Biological Processes	19	molting cycle process	-4.90554	11.80406	7.736806	30242	84	183	6	3.278689	1.316393	596|1956|2263|3066|5970|7124	BCL2|EGFR|FGFR2|HDAC2|RELA|TNF	-3.68453	0	50	0	0	-12.7609	35.92539796
1	-4.90554	GO:0022405	M1	1	0	GO Biological Processes	19	hair cycle process	-4.90554	11.80406	7.736806	30242	84	183	6	3.278689	1.316393	596|1956|2263|3066|5970|7124	BCL2|EGFR|FGFR2|HDAC2|RELA|TNF	-3.68453	0	50	0	0	-12.7609	35.92539796
1	-4.87592	GO:0098773	M1	1	0	GO Biological Processes	19	skin epidermis development	-4.87592	11.66519	7.682813	30242	85	183	6	3.278689	1.316393	596|1956|2263|3066|5970|7124	BCL2|EGFR|FGFR2|HDAC2|RELA|TNF	-3.65994	0	50	0	0	-12.7609	35.92539796
1	-12.7041	GO:0032355	M1	1	0	GO Biological Processes	19	response to estradiol	-12.7041	16.40848	14.3092	30242	141	183	14	7.650273	1.964856	595|836|890|1544|1588|1728|1956|2099|2100|2155|5159|5743|6532|7040	CCND1|CASP3|CCNA2|CYP1A2|CYP19A1|NQO1|EGFR|ESR1|ESR2|F7|PDGFRB|PTGS2|SLC6A4|TGFB1	-10.8365	0	51	1	1	-12.7041	17.39545585
1	-4.08863	GO:0071392	M1	1	0	GO Biological Processes	19	cellular response to estradiol stimulus	-4.08863	17.39546	7.890771	30242	38	183	4	2.185792	1.080886	890|1956|2099|2100	CCNA2|EGFR|ESR1|ESR2	-2.96862	0	51	0	0	-12.7041	17.39545585

<table>
<tr><td>1</td><td>-3.56418</td><td>GO:0000079</td><td>M1</td><td>1</td><td>0</td><td>GO Biological Processes</td><td>19</td><td>regulation of cyclin-dependent protein serine/threonine kinase activity</td><td>-3.56418</td><td>8.790257</td><td>5.902321</td><td>30242</td><td>94</td><td>183</td><td>5</td><td>2.73224</td><td>1.205087</td><td>595|836|890|1956|4221</td><td>CCND1|CASP3|CCNA2|EGFR|MEN1</td><td>-2.50365</td><td>0</td><td>51</td><td>0</td><td>0</td><td>-12.7041</td><td>17.39545585</td></tr>
<tr><td>1</td><td>-3.48019</td><td>GO:1904029</td><td>M1</td><td>1</td><td>0</td><td>GO Biological Processes</td><td>19</td><td>regulation of cyclin-dependent protein kinase activity</td><td>-3.48019</td><td>8.431471</td><td>5.749416</td><td>30242</td><td>98</td><td>183</td><td>5</td><td>2.73224</td><td>1.205087</td><td>595|836|890|1956|4221</td><td>CCND1|CASP3|CCNA2|EGFR|MEN1</td><td>-2.42816</td><td>0</td><td>51</td><td>0</td><td>0</td><td>-12.7041</td><td>17.39545585</td></tr>
<tr><td>1</td><td>-12.5928</td><td>GO:0048771</td><td>M1</td><td>1</td><td>0</td><td>GO Biological Processes</td><td>19</td><td>tissue remodeling</td><td>-12.5928</td><td>14.08439</td><td>13.58339</td><td>30242</td><td>176</td><td>183</td><td>15</td><td>8.196721</td><td>2.027793</td><td>154|760|1636|1956|2149|3351|3558|3569|4313|4846|7040|7157|7421|9475|729230</td><td>ADRB2|CA2|ACE|EGFR|F2R|HTR1B|IL2|IL6|MMP2|NOS3|TGFB1|TP53|VDR|ROCK2|CCR2</td><td>-10.7353</td><td>0</td><td>52</td><td>1</td><td>1</td><td>-12.5928</td><td>24.48249342</td></tr>
<tr><td>1</td><td>-12.1225</td><td>GO:0048871</td><td>M1</td><td>1</td><td>0</td><td>GO Biological Processes</td><td>19</td><td>multicellular organismal homeostasis</td><td>-12.1225</td><td>7.114775</td><td>10.87734</td><td>30242</td><td>511</td><td>183</td><td>22</td><td>12.02186</td><td>2.404073</td><td>134|153|154|596|760|1080|1268|1813|1910|1956|2149|3356|3569|4846|5290|5743|6513|7099|7124|7442|59341|729230</td><td>ADORA1|ADRB1|ADRB2|BCL2|CA2|CFTR|CNR1|DRD2|EDNRB|EGFR|F2R|HTR2A|IL6|NOS3|PIK3CA|PTGS2|SLC2A1|TLR4|TNF|TRPV1|TRPV4|CCR2</td><td>-10.2883</td><td>0</td><td>52</td><td>0</td><td>0</td><td>-12.5928</td><td>24.48249342</td></tr>
<tr><td>1</td><td>-9.01631</td><td>GO:0060249</td><td>M1</td><td>1</td><td>0</td><td>GO Biological Processes</td><td>19</td><td>anatomical structure homeostasis</td><td>-9.01631</td><td>7.894435</td><td>9.582106</td><td>30242</td><td>314</td><td>183</td><td>15</td><td>8.196721</td><td>2.027793</td><td>134|154|596|760|1268|1956|2149|3351|3569|4846|5743|6513|6530|7099|729230</td><td>ADORA1|ADRB2|BCL2|CA2|CNR1|EGFR|F2R|HTR1B|IL6|NOS3|PTGS2|SLC2A1|SLC6A2|TLR4|CCR2</td><td>-7.40239</td><td>0</td><td>52</td><td>0</td><td>0</td><td>-12.5928</td><td>24.48249342</td></tr>
<tr><td>1</td><td>-6.09702</td><td>GO:0001894</td><td>M1</td><td>1</td><td>0</td><td>GO Biological Processes</td><td>19</td><td>tissue homeostasis</td><td>-6.09702</td><td>6.78293</td><td>7.41958</td><td>30242</td><td>268</td><td>183</td><td>11</td><td>6.010929</td><td>1.757049</td><td>154|596|760|1956|2149|3569|4846|5743|6513|7099|729230</td><td>ADRB2|BCL2|CA2|EGFR|F2R|IL6|NOS3|PTGS2|SLC2A1|TLR4|CCR2</td><td>-4.74114</td><td>0</td><td>52</td><td>0</td><td>0</td><td>-12.5928</td><td>24.48249342</td></tr>
<tr><td>1</td><td>-4.68993</td><td>GO:0048873</td><td>M1</td><td>1</td><td>0</td><td>GO Biological Processes</td><td>19</td><td>homeostasis of number of cells within a tissue</td><td>-4.68993</td><td>24.48249</td><td>9.524697</td><td>30242</td><td>27</td><td>183</td><td>4</td><td>2.185792</td><td>1.080886</td><td>596|2149|4846|729230</td><td>BCL2|F2R|NOS3|CCR2</td><td>-3.49655</td><td>0</td><td>52</td><td>0</td><td>0</td><td>-12.5928</td><td>24.48249342</td></tr>
<tr><td>1</td><td>-12.3707</td><td>GO:0014074</td><td>M1</td><td>1</td><td>0</td><td>GO Biological Processes</td><td>19</td><td>response to purine-containing compound</td><td>-12.3707</td><td>15.52749</td><td>13.87034</td><td>30242</td><td>149</td><td>183</td><td>14</td><td>7.650273</td><td>1.964856</td><td>135|328|351|834|1080|1723|3066|5024|5138|5743|5970|6531|6532|7442</td><td>ADORA2A|APEX1|APP|CASP1|CFTR|DHODH|HDAC2|P2RX3|PDE2A|PTGS2|RELA|SLC6A3|SLC6A4|TRPV1</td><td>-10.523</td><td>0</td><td>53</td><td>1</td><td>1</td><td>-12.3707</td><td>16.27529392</td></tr>
<tr><td>1</td><td>-11.787</td><td>GO:0046683</td><td>M1</td><td>1</td><td>0</td><td>GO Biological Processes</td><td>19</td><td>response to organophosphorus</td><td>-11.787</td><td>16.27529</td><td>13.72327</td><td>30242</td><td>132</td><td>183</td><td>13</td><td>7.103825</td><td>1.898976</td><td>328|351|834|1080|1645|5024|5138|5743|5970|6531|6532|7298|7442</td><td>APEX1|APP|CASP1|CFTR|AKR1C1|P2RX3|PDE2A|PTGS2|RELA|SLC6A3|SLC6A4|TYMS|TRPV1</td><td>-9.97664</td><td>0</td><td>53</td><td>0</td><td>0</td><td>-12.3707</td><td>16.27529392</td></tr>
<tr><td>1</td><td>-4.62554</td><td>GO:0051591</td><td>M1</td><td>1</td><td>0</td><td>GO Biological Processes</td><td>19</td><td>response to cAMP</td><td>-4.62554</td><td>10.54831</td><td>7.234316</td><td>30242</td><td>94</td><td>183</td><td>6</td><td>3.278689</td><td>1.316393</td><td>328|351|1080|5138|5970|6531</td><td>APEX1|APP|CFTR|PDE2A|RELA|SLC6A3</td><td>-3.43978</td><td>0</td><td>53</td><td>0</td><td>0</td><td>-12.3707</td><td>16.27529392</td></tr>
<tr><td>1</td><td>-3.48949</td><td>GO:0071320</td><td>M1</td><td>1</td><td>0</td><td>GO Biological Processes</td><td>19</td><td>cellular response to cAMP</td><td>-3.48949</td><td>12.24125</td><td>6.451052</td><td>30242</td><td>54</td><td>183</td><td>4</td><td>2.185792</td><td>1.080886</td><td>328|351|1080|5138</td><td>APEX1|APP|CFTR|PDE2A</td><td>-2.43683</td><td>0</td><td>53</td><td>0</td><td>0</td><td>-12.3707</td><td>16.27529392</td></tr>
<tr><td>1</td><td>-12.2901</td><td>GO:0050673</td><td>M1</td><td>1</td><td>0</td><td>GO Biological Processes</td><td>19</td><td>epithelial cell proliferation</td><td>-12.2901</td><td>7.746414</td><td>11.22494</td><td>30242</td><td>448</td><td>183</td><td>21</td><td>11.47541</td><td>2.356083</td><td>185|367|595|1910|1956|2099|2263|3357|3569|4221|5241|5293|5467|5468|5591|6331|6347|7040|7046|7124|7421</td><td>AGTR1|AR|CCND1|EDNRB|EGFR|ESR1|FGFR2|HTR2B|IL6|MEN1|PGR|PIK3CD|PPARD|PPARG|PRKDC|SCN5A|CCL2|TGFB1|TGFBR1|TNF|VDR</td><td>-10.4483</td><td>0</td><td>54</td><td>1</td><td>1</td><td>-12.2901</td><td>10.01556549</td></tr>
</table>

1	-11.4466	GO:0050678	M1	1	0	GO Biological Processes	19	regulation of epithelial cell proliferation	-11.4466	8.050974	10.93531	30242	390	183	19	10.38251	2.254873	185|367|595|1910|1956|2263|3357|4221|5241|5293|5467|5468|5591|6331|6347|7040|7046|7124|7421	AGTR1|AR|CCND1|EDNRB|EGFR|FGFR2|HTR2B|MEN1|PGR|PIK3CD|PPARD|PPARG|PRKDC|SCN5A|CCL2|TGFB1|TGFBR1|TNF|VDR	-9.65378	0	54	0	0	-12.2901	10.01556549
1	-7.14604	GO:0050680	M1	1	0	GO Biological Processes	19	negative regulation of epithelial cell proliferation	-7.14604	10.01557	9.060539	30242	165	183	10	5.464481	1.680144	367|2263|4221|5467|5468|6347|7040|7046|7124|7421	AR|FGFR2|MEN1|PPARD|PPARG|CCL2|TGFB1|TGFBR1|TNF|VDR	-5.69591	0	54	0	0	-12.2901	10.01556549
1	-5.28775	GO:0050679	M1	1	0	GO Biological Processes	19	positive regulation of epithelial cell proliferation	-5.28775	7.18508	6.967115	30242	207	183	9	4.918033	1.598524	185|367|595|1956|2263|3357|5293|6331|7046	AGTR1|AR|CCND1|EGFR|FGFR2|HTR2B|PIK3CD|SCN5A|TGFBR1	-4.02068	0	54	0	0	-12.2901	10.01556549
1	-3.85212	GO:0001936	M1	1	0	GO Biological Processes	19	regulation of endothelial cell proliferation	-3.85212	6.252961	5.591825	30242	185	183	7	3.825137	1.417845	185|3357|5293|5468|6347|7046|7124	AGTR1|HTR2B|PIK3CD|PPARG|CCL2|TGFBR1|TNF	-2.75789	0	54	0	0	-12.2901	10.01556549
1	-3.64423	GO:0001935	M1	1	0	GO Biological Processes	19	endothelial cell proliferation	-3.64423	5.783989	5.296355	30242	200	183	7	3.825137	1.417845	185|3357|5293|5468|6347|7046|7124	AGTR1|HTR2B|PIK3CD|PPARG|CCL2|TGFBR1|TNF	-2.57503	0	54	0	0	-12.2901	10.01556549
1	-2.90504	GO:0001937	M1	1	0	GO Biological Processes	19	negative regulation of endothelial cell proliferation	-2.90504	8.58477	5.199631	30242	77	183	4	2.185792	1.080886	5468|6347|7046|7124	PPARG|CCL2|TGFBR1|TNF	-1.911	0	54	0	0	-12.2901	10.01556549
1	-2.32802	GO:0001938	M1	1	0	GO Biological Processes	19	positive regulation of endothelial cell proliferation	-2.32802	5.955201	4.080866	30242	111	183	4	2.185792	1.080886	185|3357|5293|7046	AGTR1|HTR2B|PIK3CD|TGFBR1	-1.39211	0	54	0	0	-12.2901	10.01556549
1	-12.0998	GO:0030155	M1	1	0	GO Biological Processes	19	regulation of cell adhesion	-12.0998	5.721275	10.22282	30242	751	183	26	14.20765	2.580833	100|135|472|596|836|1545|1991|2335|3383|3558|3569|3576|4221|5290|5291|5465|5970|5979|6347|6401|7040|7124|9475|59341|114548|729230	ADA|ADORA2A|ATM|BCL2|CASP3|CYP1B1|ELANE|FN1|ICAM1|IL2|IL6|CXCL8|MEN1|PIK3CA|PIK3CB|PPARA|RELA|RET|CCL2|SELE|TGFB1|TNF|ROCK2|TRPV4|NLRP3|CCR2	-10.2712	0	55	1	1	-12.0998	21.55523877
1	-9.87452	GO:0007159	M1	1	0	GO Biological Processes	19	leukocyte cell-cell adhesion	-9.87452	7.572415	9.938559	30242	371	183	17	9.289617	2.145862	100|135|836|1991|3383|3558|3569|3683|5290|5465|5970|6347|6401|6793|7124|114548|729230	ADA|ADORA2A|CASP3|ELANE|ICAM1|IL2|IL6|ITGAL|PIK3CA|PPARA|RELA|CCL2|SELE|STK10|TNF|NLRP3|CCR2	-8.20497	0	55	0	0	-12.0998	21.55523877
1	-9.84771	GO:0002521	M1	1	0	GO Biological Processes	19	leukocyte differentiation	-9.84771	6.086808	9.332773	30242	543	183	20	10.92896	2.306387	100|351|472|596|760|1230|3558|3569|4318|4914|5293|5468|5591|5734|7040|7099|7124|7157|114548|729230	ADA|APP|ATM|BCL2|CA2|CCR1|IL2|IL6|MMP9|NTRK1|PIK3CD|PPARG|PRKDC|PTGER4|TGFB1|TLR4|TNF|TP53|NLRP3|CCR2	-8.18065	0	55	0	0	-12.0998	21.55523877
1	-8.61541	GO:1903037	M1	1	0	GO Biological Processes	19	regulation of leukocyte cell-cell adhesion	-8.61541	7.377537	9.172333	30242	336	183	15	8.196721	2.027793	100|135|836|1991|3383|3558|3569|5290|5465|5970|6347|6401|7124|114548|729230	ADA|ADORA2A|CASP3|ELANE|ICAM1|IL2|IL6|PIK3CA|PPARA|RELA|CCL2|SELE|TNF|	-7.02365	0	55	0	0	-12.0998	21.55523877

																			NLRP3|CCR2							
1	-8.59989	GO:0022407	M1	1	0	GO Biological Processes	19	regulation of cell-cell adhesion	-8.59989	6.243036	8.743305	30242	450	183	17	9.289617	2.145862	100|135|836|1991|3383|3558|3569|5290|5465|5970|6347|6401|7040|7124|59341|114548|729230	ADA|ADORA2A|CASP3|ELANE|ICAM1|IL2|IL6|PIK3CA|PPARA|RELA|CCL2|SELE|TGFB1|TNF|TRPV4|NLRP3|CCR2	-7.01029	0	55	0	0	-12.0998	21.55523877
1	-7.94052	GO:0046649	M1	1	0	GO Biological Processes	19	lymphocyte activation	-7.94052	4.477927	7.653141	30242	775	183	21	11.47541	2.356083	100|135|472|596|836|3383|3558|3569|3683|4914|5290|5293|5591|5734|6347|7040|7099|7157|29110|114548|729230	ADA|ADORA2A|ATM|BCL2|CASP3|ICAM1|IL2|IL6|ITGAL|NTRK1|PIK3CA|PIK3CD|PRKDC|PTGER4|CCL2|TGFB1|TLR4|TP53|TBK1|NLRP3|CCR2	-6.41012	0	55	0	0	-12.0998	21.55523877
1	-7.78572	GO:0050778	M1	1	0	GO Biological Processes	19	positive regulation of immune response	-7.78572	5.155326	7.863342	30242	577	183	18	9.836066	2.201413	100|596|1268|1991|3551|3558|3569|5142|5290|5293|5591|5970|7040|7099|7124|29110|114548|729230	ADA|BCL2|CNR1|ELANE|IKBKB|IL2|IL6|PDE4B|PIK3CA|PIK3CD|PRKDC|RELA|TGFB1|TLR4|TNF|TBK1|NLRP3|CCR2	-6.27098	0	55	0	0	-12.0998	21.55523877
1	-7.71747	GO:0002263	M1	1	0	GO Biological Processes	19	cell activation involved in immune response	-7.71747	7.645334	8.73229	30242	281	183	13	7.103825	1.898976	100|351|3383|3558|3569|3683|5293|5734|7040|7099|7157|114548|729230	ADA|APP|ICAM1|IL2|IL6|ITGAL|PIK3CD|PTGER4|TGFB1|TLR4|TP53|NLRP3|CCR2	-6.2108	0	55	0	0	-12.0998	21.55523877
1	-7.65762	GO:0002252	M1	1	0	GO Biological Processes	19	immune effector process	-7.65762	4.771854	7.632656	30242	658	183	19	10.38251	2.254873	100|351|1636|1991|2147|3383|3558|3569|3683|4843|5293|5591|5734|7040|7099|7124|7157|114548|729230	ADA|APP|ACE|ELANE|F2|ICAM1|IL2|IL6|ITGAL|NOS2|PIK3CD|PRKDC|PTGER4|TGFB1|TLR4|TNF|TP53|NLRP3|CCR2	-6.15537	0	55	0	0	-12.0998	21.55523877
1	-7.55442	GO:1903039	M1	1	0	GO Biological Processes	19	positive regulation of leukocyte cell-cell adhesion	-7.55442	8.297414	8.837348	30242	239	183	12	6.557377	1.829835	100|1991|3383|3558|3569|5290|5970|6347|6401|7124|114548|729230	ADA|ELANE|ICAM1|IL2|IL6|PIK3CA|RELA|CCL2|SELE|TNF|NLRP3|CCR2	-6.06259	0	55	0	0	-12.0998	21.55523877
1	-7.25524	GO:0042110	M1	1	0	GO Biological Processes	19	T cell activation	-7.25524	5.407176	7.666301	30242	489	183	16	8.743169	2.088053	100|135|596|836|3383|3558|3569|3683|5290|5293|5591|5734|6347|7157|114548|729230	ADA|ADORA2A|BCL2|CASP3|ICAM1|IL2|IL6|ITGAL|PIK3CA|PIK3CD|PRKDC|PTGER4|CCL2|TP53|NLRP3|CCR2	-5.79724	0	55	0	0	-12.0998	21.55523877
1	-7.10091	GO:0045785	M1	1	0	GO Biological Processes	19	positive regulation of cell adhesion	-7.10091	5.672431	7.676715	30242	437	183	15	8.196721	2.027793	100|472|1991|2335|3383|3558|3569|5290|5970|5979|6347|6401|7124|114548|729230	ADA|ATM|ELANE|FN1|ICAM1|IL2|IL6|PIK3CA|RELA|RET|CCL2|SELE|TNF|NLRP3|CCR2	-5.65389	0	55	0	0	-12.0998	21.55523877
1	-7.00706	GO:0050865	M1	1	0	GO Biological Processes	19	regulation of cell activation	-7.00706	4.576343	7.191923	30242	650	183	18	9.836066	2.201413	100|135|472|596|836|1268|2147|3558|3569|4846|5159|5290|5591|6347|7040|7099|114548|729230	ADA|ADORA2A|ATM|BCL2|CASP3|CNR1|F2|IL2|IL6|NOS3|PDGFRB|PIK3CA|PRKDC|CCL2|TGFB1|TLR4|NLRP3|CCR2	-5.5715	0	55	0	0	-12.0998	21.55523877

<table>
<tr><td>1</td><td>-6.85479</td><td>GO:0002366</td><td>M1</td><td>1</td><td>0</td><td>GO Biological Processes</td><td>19</td><td>leukocyte activation involved in immune response</td><td>-6.85479</td><td>7.159141</td><td>8.035062</td><td>30242</td><td>277</td><td>183</td><td>12</td><td>6.557377</td><td>1.829835</td><td>100|3383|3558|3569|3683|5293|5734|7040|7099|7157|114548|729230</td><td>ADA|ICAM1|IL2|IL6|ITGAL|PIK3CD|PTGER4|TGFB1|TLR4|TP53|NLRP3|CCR2</td><td>-5.42893</td><td>0</td><td>55</td><td>0</td><td>0</td><td>-12.0998</td><td>21.55523877</td></tr>
<tr><td>1</td><td>-6.73801</td><td>GO:0022409</td><td>M1</td><td>1</td><td>0</td><td>GO Biological Processes</td><td>19</td><td>positive regulation of cell-cell adhesion</td><td>-6.73801</td><td>6.982683</td><td>7.903785</td><td>30242</td><td>284</td><td>183</td><td>12</td><td>6.557377</td><td>1.829835</td><td>100|1991|3383|3558|3569|5290|5970|6347|6401|7124|114548|729230</td><td>ADA|ELANE|ICAM1|IL2|IL6|PIK3CA|RELA|CCL2|SELE|TNF|NLRP3|CCR2</td><td>-5.32627</td><td>0</td><td>55</td><td>0</td><td>0</td><td>-12.0998</td><td>21.55523877</td></tr>
<tr><td>1</td><td>-6.4694</td><td>GO:0002285</td><td>M1</td><td>1</td><td>0</td><td>GO Biological Processes</td><td>19</td><td>lymphocyte activation involved in immune response</td><td>-6.4694</td><td>8.474709</td><td>8.170477</td><td>30242</td><td>195</td><td>183</td><td>10</td><td>5.464481</td><td>1.680144</td><td>100|3383|3558|3569|3683|5734|7040|7099|7157|114548</td><td>ADA|ICAM1|IL2|IL6|ITGAL|PTGER4|TGFB1|TLR4|TP53|NLRP3</td><td>-5.08298</td><td>0</td><td>55</td><td>0</td><td>0</td><td>-12.0998</td><td>21.55523877</td></tr>
<tr><td>1</td><td>-5.9909</td><td>GO:0042113</td><td>M1</td><td>1</td><td>0</td><td>GO Biological Processes</td><td>19</td><td>B cell activation</td><td>-5.9909</td><td>5.937371</td><td>7.079648</td><td>30242</td><td>334</td><td>183</td><td>12</td><td>6.557377</td><td>1.829835</td><td>100|472|596|836|3558|3569|4914|5293|5591|7040|7099|7157</td><td>ADA|ATM|BCL2|CASP3|IL2|IL6|NTRK1|PIK3CD|PRKDC|TGFB1|TLR4|TP53</td><td>-4.64373</td><td>0</td><td>55</td><td>0</td><td>0</td><td>-12.0998</td><td>21.55523877</td></tr>
<tr><td>1</td><td>-5.71977</td><td>GO:0032943</td><td>M1</td><td>1</td><td>0</td><td>GO Biological Processes</td><td>19</td><td>mononuclear cell proliferation</td><td>-5.71977</td><td>6.204181</td><td>6.984437</td><td>30242</td><td>293</td><td>183</td><td>11</td><td>6.010929</td><td>1.757049</td><td>100|472|596|836|1636|3558|3569|7099|7157|29110|729230</td><td>ADA|ATM|BCL2|CASP3|ACE|IL2|IL6|TLR4|TP53|TBK1|CCR2</td><td>-4.39711</td><td>0</td><td>55</td><td>0</td><td>0</td><td>-12.0998</td><td>21.55523877</td></tr>
<tr><td>1</td><td>-5.64484</td><td>GO:1903131</td><td>M1</td><td>1</td><td>0</td><td>GO Biological Processes</td><td>19</td><td>mononuclear cell differentiation</td><td>-5.64484</td><td>5.031238</td><td>6.54593</td><td>30242</td><td>427</td><td>183</td><td>13</td><td>7.103825</td><td>1.898976</td><td>100|472|596|3558|3569|4914|5293|5468|5591|5734|7157|114548|729230</td><td>ADA|ATM|BCL2|IL2|IL6|NTRK1|PIK3CD|PPARG|PRKDC|PTGER4|TP53|NLRP3|CCR2</td><td>-4.33252</td><td>0</td><td>55</td><td>0</td><td>0</td><td>-12.0998</td><td>21.55523877</td></tr>
<tr><td>1</td><td>-5.60904</td><td>GO:0030183</td><td>M1</td><td>1</td><td>0</td><td>GO Biological Processes</td><td>19</td><td>B cell differentiation</td><td>-5.60904</td><td>9.376274</td><td>7.778688</td><td>30242</td><td>141</td><td>183</td><td>8</td><td>4.371585</td><td>1.511428</td><td>100|472|596|3558|3569|4914|5591|7157</td><td>ADA|ATM|BCL2|IL2|IL6|NTRK1|PRKDC|TP53</td><td>-4.29955</td><td>0</td><td>55</td><td>0</td><td>0</td><td>-12.0998</td><td>21.55523877</td></tr>
<tr><td>1</td><td>-5.59621</td><td>GO:0030888</td><td>M1</td><td>1</td><td>0</td><td>GO Biological Processes</td><td>19</td><td>regulation of B cell proliferation</td><td>-5.59621</td><td>15.49283</td><td>9.055955</td><td>30242</td><td>64</td><td>183</td><td>6</td><td>3.278689</td><td>1.316393</td><td>100|472|596|836|3558|7099</td><td>ADA|ATM|BCL2|CASP3|IL2|TLR4</td><td>-4.28953</td><td>0</td><td>55</td><td>0</td><td>0</td><td>-12.0998</td><td>21.55523877</td></tr>
<tr><td>1</td><td>-5.48281</td><td>GO:0030098</td><td>M1</td><td>1</td><td>0</td><td>GO Biological Processes</td><td>19</td><td>lymphocyte differentiation</td><td>-5.48281</td><td>5.302358</td><td>6.532443</td><td>30242</td><td>374</td><td>183</td><td>12</td><td>6.557377</td><td>1.829835</td><td>100|472|596|3558|3569|4914|5293|5591|5734|7157|114548|729230</td><td>ADA|ATM|BCL2|IL2|IL6|NTRK1|PIK3CD|PRKDC|PTGER4|TP53|NLRP3|CCR2</td><td>-4.18555</td><td>0</td><td>55</td><td>0</td><td>0</td><td>-12.0998</td><td>21.55523877</td></tr>
<tr><td>1</td><td>-5.4706</td><td>GO:0002443</td><td>M1</td><td>1</td><td>0</td><td>GO Biological Processes</td><td>19</td><td>leukocyte mediated immunity</td><td>-5.4706</td><td>4.849523</td><td>6.368605</td><td>30242</td><td>443</td><td>183</td><td>13</td><td>7.103825</td><td>1.898976</td><td>1636|1991|2147|3383|3558|3569|4843|5293|7040|7099|7124|114548|729230</td><td>ACE|ELANE|F2|ICAM1|IL2|IL6|NOS2|PIK3CD|TGFB1|TLR4|TNF|NLRP3|CCR2</td><td>-4.17553</td><td>0</td><td>55</td><td>0</td><td>0</td><td>-12.0998</td><td>21.55523877</td></tr>
<tr><td>1</td><td>-5.46659</td><td>GO:0051249</td><td>M1</td><td>1</td><td>0</td><td>GO Biological Processes</td><td>19</td><td>regulation of lymphocyte activation</td><td>-5.46659</td><td>4.492419</td><td>6.237215</td><td>30242</td><td>515</td><td>183</td><td>14</td><td>7.650273</td><td>1.964856</td><td>100|135|472|596|836|3558|3569|5290|5591|6347|7040|7099|114548|729230</td><td>ADA|ADORA2A|ATM|BCL2|CASP3|IL2|IL6|PIK3CA|PRKDC|CCL2|TGFB1|TLR4|NLRP3|CCR2</td><td>-4.17207</td><td>0</td><td>55</td><td>0</td><td>0</td><td>-12.0998</td><td>21.55523877</td></tr>
<tr><td>1</td><td>-5.40936</td><td>GO:0071887</td><td>M1</td><td>1</td><td>0</td><td>GO Biological Processes</td><td>19</td><td>leukocyte apoptotic process</td><td>-5.40936</td><td>10.91319</td><td>7.977371</td><td>30242</td><td>106</td><td>183</td><td>7</td><td>3.825137</td><td>1.417845</td><td>100|836|3558|3569|5291|5293|7157</td><td>ADA|CASP3|IL2|IL6|PIK3CB|PIK3CD|TP53</td><td>-4.12079</td><td>0</td><td>55</td><td>0</td><td>0</td><td>-12.0998</td><td>21.55523877</td></tr>
<tr><td>1</td><td>-5.38543</td><td>GO:0030217</td><td>M1</td><td>1</td><td>0</td><td>GO Biological Processes</td><td>19</td><td>T cell differentiation</td><td>-5.38543</td><td>6.430227</td><td>6.821327</td><td>30242</td><td>257</td><td>183</td><td>10</td><td>5.464481</td><td>1.680144</td><td>100|596|3558|3569|5293|5591|5734|7157|114548|729230</td><td>ADA|BCL2|IL2|IL6|PIK3CD|PRKDC|PTGER4|TP53|NLRP3|CCR2</td><td>-4.10114</td><td>0</td><td>55</td><td>0</td><td>0</td><td>-12.0998</td><td>21.55523877</td></tr>
<tr><td>1</td><td>-5.38218</td><td>GO:0002824</td><td>M1</td><td>1</td><td>0</td><td>GO Biological Processes</td><td>19</td><td>positive regulation of adaptive immune response based on somatic recombination of immune receptors built from</td><td>-5.38218</td><td>10.81119</td><td>7.932581</td><td>30242</td><td>107</td><td>183</td><td>7</td><td>3.825137</td><td>1.417845</td><td>100|3558|3569|7040|7124|114548|729230</td><td>ADA|IL2|IL6|TGFB1|TNF|NLRP3|CCR2</td><td>-4.10001</td><td>0</td><td>55</td><td>0</td><td>0</td><td>-12.0998</td><td>21.55523877</td></tr>
</table>

								immunoglobulin superfamily domains																		
1	-5.35274	GO:0070661	M1	1	0	GO Biological Processes	19	leukocyte proliferation	-5.35274	5.680704	6.56791	30242	320	183	11	6.010929	1.757049	100|472|596|836|1636|3558|3569|7099|7157|29110|729230	ADA|ATM|BCL2|CASP3|ACE|IL2|IL6|TLR4|TP53|TBK1|CCR2	-4.07689	0	55	0	0	-12.0998	21.55523877
1	-5.32612	GO:0002694	M1	1	0	GO Biological Processes	19	regulation of leukocyte activation	-5.32612	4.090516	5.996444	30242	606	183	15	8.196721	2.027793	100|135|472|596|836|1268|3558|3569|5290|5591|6347|7040|7099|114548|729230	ADA|ADORA2A|ATM|BCL2|CASP3|CNR1|IL2|IL6|PIK3CA|PRKDC|CCL2|TGFB1|TLR4|NLRP3|CCR2	-4.05441	0	55	0	0	-12.0998	21.55523877
1	-5.28263	GO:0046631	M1	1	0	GO Biological Processes	19	alpha-beta T cell activation	-5.28263	8.474709	7.303159	30242	156	183	8	4.371585	1.511428	100|135|596|3558|3569|5734|114548|729230	ADA|ADORA2A|BCL2|IL2|IL6|PTGER4|NLRP3|CCR2	-4.01607	0	55	0	0	-12.0998	21.55523877
1	-5.2504	GO:0002821	M1	1	0	GO Biological Processes	19	positive regulation of adaptive immune response	-5.2504	10.32855	7.717205	30242	112	183	7	3.825137	1.417845	100|3558|3569|7040|7124|114548|729230	ADA|IL2|IL6|TGFB1|TNF|NLRP3|CCR2	-3.98791	0	55	0	0	-12.0998	21.55523877
1	-5.22161	GO:1902107	M1	1	0	GO Biological Processes	19	positive regulation of leukocyte differentiation	-5.22161	8.314809	7.215681	30242	159	183	8	4.371585	1.511428	100|760|1230|3558|5591|7040|7124|114548	ADA|CA2|CCR1|IL2|PRKDC|TGFB1|TNF|NLRP3	-3.96264	0	55	0	0	-12.0998	21.55523877
1	-5.22161	GO:1903708	M1	1	0	GO Biological Processes	19	positive regulation of hemopoiesis	-5.22161	8.314809	7.215681	30242	159	183	8	4.371585	1.511428	100|760|1230|3558|5591|7040|7124|114548	ADA|CA2|CCR1|IL2|PRKDC|TGFB1|TNF|NLRP3	-3.96264	0	55	0	0	-12.0998	21.55523877
1	-5.19952	GO:0002286	M1	1	0	GO Biological Processes	19	T cell activation involved in immune response	-5.19952	10.14735	7.634822	30242	114	183	7	3.825137	1.417845	3383|3558|3569|3683|5734|7157|114548	ICAM1|IL2|IL6|ITGAL|PTGER4|TP53|NLRP3	-3.94355	0	55	0	0	-12.0998	21.55523877
1	-5.08418	GO:0002700	M1	1	0	GO Biological Processes	19	regulation of production of molecular mediator of immune response	-5.08418	7.964185	7.020219	30242	166	183	8	4.371585	1.511428	3558|3569|5591|7040|7099|7124|114548|729230	IL2|IL6|PRKDC|TGFB1|TLR4|TNF|NLRP3|CCR2	-3.84002	0	55	0	0	-12.0998	21.55523877
1	-5.04515	GO:1902105	M1	1	0	GO Biological Processes	19	regulation of leukocyte differentiation	-5.04515	5.881026	6.413904	30242	281	183	10	5.464481	1.680144	100|760|1230|3558|5591|7040|7099|7124|114548|729230	ADA|CA2|CCR1|IL2|PRKDC|TGFB1|TLR4|TNF|NLRP3|CCR2	-3.80437	0	55	0	0	-12.0998	21.55523877
1	-4.97407	GO:0050867	M1	1	0	GO Biological Processes	19	positive regulation of cell activation	-4.97407	4.721624	5.992735	30242	420	183	12	6.557377	1.829835	100|596|3558|3569|5159|5290|5591|6347|7040|7099|114548|729230	ADA|BCL2|IL2|IL6|PDGFRB|PIK3CA|PRKDC|CCL2|TGFB1|TLR4|NLRP3|CCR2	-3.7414	0	55	0	0	-12.0998	21.55523877
1	-4.96526	GO:0002703	M1	1	0	GO Biological Processes	19	regulation of leukocyte mediated immunity	-4.96526	6.552033	6.55137	30242	227	183	9	4.918033	1.598524	3383|3558|3569|4843|7040|7099|7124|114548|729230	ICAM1|IL2|IL6|NOS2|TGFB1|TLR4|TNF|NLRP3|CCR2	-3.73495	0	55	0	0	-12.0998	21.55523877
1	-4.92631	GO:0046651	M1	1	0	GO Biological Processes	19	lymphocyte proliferation	-4.92631	5.698511	6.273107	30242	290	183	10	5.464481	1.680144	100|472|596|836|3558|3569|7099|7157|29110|729230	ADA|ATM|BCL2|CASP3|IL2|IL6|TLR4|TP53|TBK1|CCR2	-3.70114	0	55	0	0	-12.0998	21.55523877
1	-4.84977	GO:0051251	M1	1	0	GO Biological Processes	19	positive regulation of lymphocyte activation	-4.84977	5.021616	6.006217	30242	362	183	11	6.010929	1.757049	100|596|3558|3569|5290|5591|6347|7040|7099|114548|729230	ADA|BCL2|IL2|IL6|PIK3CA|PRKDC|CCL2|TGFB1|TLR4|NLRP3|CCR2	-3.63516	0	55	0	0	-12.0998	21.55523877
1	-4.76198	GO:1903706	M1	1	0	GO Biological Processes	19	regulation of hemopoiesis	-4.76198	4.913041	5.909074	30242	370	183	11	6.010929	1.757049	100|760|1230|3558|4792|5591|7040|7099|7124|114548|729230	ADA|CA2|CCR1|IL2|NFKBIA|PRKDC|TGFB1|TLR4|TNF|NLRP3|CCR2	-3.5559	0	55	0	0	-12.0998	21.55523877
1	-4.54796	GO:0002718	M1	1	0	GO Biological	19	regulation of cytokine	-4.54796	10.22207	7.098116	30242	97	183	6	3.278689	1.316393	3569|7040|7099|7124|1	IL6|TGFB1|TLR4|TNF	-3.37728	0	55	0	0	-12.0998	21.55523877

						Processes		production involved in immune response										14548|729230	|NLRP3|CCR2							
1	-4.53073	GO:0050864	M1	1	0	GO Biological Processes	19	regulation of B cell activation	-4.53073	6.677044	6.253344	30242	198	183	8	4.371585	1.511428	100|472|596|836|3558|3569|7040|7099	ADA|ATM|BCL2|CASP3|IL2|IL6|TGFB1|TLR4	-3.3625	0	55	0	0	-12.0998	21.55523877
1	-4.4977	GO:0002367	M1	1	0	GO Biological Processes	19	cytokine production involved in immune response	-4.4977	10.01557	7.010576	30242	99	183	6	3.278689	1.316393	3569|7040|7099|7124|114548|729230	IL6|TGFB1|TLR4|TNF|NLRP3|CCR2	-3.33635	0	55	0	0	-12.0998	21.55523877
1	-4.4977	GO:0042100	M1	1	0	GO Biological Processes	19	B cell proliferation	-4.4977	10.01557	7.010576	30242	99	183	6	3.278689	1.316393	100|472|596|836|3558|7099	ADA|ATM|BCL2|CASP3|IL2|TLR4	-3.33635	0	55	0	0	-12.0998	21.55523877
1	-4.36513	GO:0002696	M1	1	0	GO Biological Processes	19	positive regulation of leukocyte activation	-4.36513	4.44456	5.472469	30242	409	183	11	6.010929	1.757049	100|596|3558|3569|5290|5591|6347|7040|7099|114548|729230	ADA|BCL2|IL2|IL6|PIK3CA|PRKDC|CCL2|TGFB1|TLR4|NLRP3|CCR2	-3.21642	0	55	0	0	-12.0998	21.55523877
1	-4.32729	GO:0002697	M1	1	0	GO Biological Processes	19	regulation of immune effector process	-4.32729	4.846241	5.573233	30242	341	183	10	5.464481	1.680144	3383|3558|3569|4843|5591|7040|7099|7124|114548|729230	ICAM1|IL2|IL6|NOS2|PRKDC|TGFB1|TLR4|TNF|NLRP3|CCR2	-3.18362	0	55	0	0	-12.0998	21.55523877
1	-4.19646	GO:0046632	M1	1	0	GO Biological Processes	19	alpha-beta T cell differentiation	-4.19646	8.853044	6.496566	30242	112	183	6	3.278689	1.316393	100|596|3558|3569|5734|114548	ADA|BCL2|IL2|IL6|PTGER4|NLRP3	-3.06456	0	55	0	0	-12.0998	21.55523877
1	-4.12558	GO:0050670	M1	1	0	GO Biological Processes	19	regulation of lymphocyte proliferation	-4.12558	5.849799	5.710024	30242	226	183	8	4.371585	1.511428	100|472|596|836|3558|3569|7099|729230	ADA|ATM|BCL2|CASP3|IL2|IL6|TLR4|CCR2	-3.00074	0	55	0	0	-12.0998	21.55523877
1	-4.11295	GO:0002822	M1	1	0	GO Biological Processes	19	regulation of adaptive immune response based on somatic recombination of immune receptors built from immunoglobulin superfamily domains	-4.11295	6.885701	5.968891	30242	168	183	7	3.825137	1.417845	100|3558|3569|7040|7124|114548|729230	ADA|IL2|IL6|TGFB1|TNF|NLRP3|CCR2	-2.99032	0	55	0	0	-12.0998	21.55523877
1	-4.09893	GO:0032944	M1	1	0	GO Biological Processes	19	regulation of mononuclear cell proliferation	-4.09893	5.798485	5.67474	30242	228	183	8	4.371585	1.511428	100|472|596|836|3558|3569|7099|729230	ADA|ATM|BCL2|CASP3|IL2|IL6|TLR4|CCR2	-2.97776	0	55	0	0	-12.0998	21.55523877
1	-3.98232	GO:0002699	M1	1	0	GO Biological Processes	19	positive regulation of immune effector process	-3.98232	5.57829	5.52099	30242	237	183	8	4.371585	1.511428	3558|3569|4843|7040|7099|7124|114548|729230	IL2|IL6|NOS2|TGFB1|TLR4|TNF|NLRP3|CCR2	-2.87127	0	55	0	0	-12.0998	21.55523877
1	-3.91602	GO:0030890	M1	1	0	GO Biological Processes	19	positive regulation of B cell proliferation	-3.91602	15.73875	7.457926	30242	42	183	4	2.185792	1.080886	100|596|3558|7099	ADA|BCL2|IL2|TLR4	-2.81521	0	55	0	0	-12.0998	21.55523877
1	-3.91417	GO:0002440	M1	1	0	GO Biological Processes	19	production of molecular mediator of immune response	-3.91417	4.797779	5.244197	30242	310	183	9	4.918033	1.598524	1991|3558|3569|5591|7040|7099|7124|114548|729230	ELANE|IL2|IL6|PRKDC|TGFB1|TLR4|TNF|NLRP3|CCR2	-2.8137	0	55	0	0	-12.0998	21.55523877
1	-3.88133	GO:0002819	M1	1	0	GO Biological Processes	19	regulation of adaptive immune response	-3.88133	6.3213	5.633682	30242	183	183	7	3.825137	1.417845	100|3558|3569|7040|7124|114548|729230	ADA|IL2|IL6|TGFB1|TNF|NLRP3|CCR2	-2.78226	0	55	0	0	-12.0998	21.55523877
1	-3.87087	GO:0070663	M1	1	0	GO Biological Processes	19	regulation of leukocyte proliferation	-3.87087	5.374206	5.374913	30242	246	183	8	4.371585	1.511428	100|472|596|836|3558|3569|7099|729230	ADA|ATM|BCL2|CASP3|IL2|IL6|TLR4|CCR2	-2.77284	0	55	0	0	-12.0998	21.55523877
1	-3.86758	GO:2000106	M1	1	0	GO Biological Processes	19	regulation of leukocyte apoptotic process	-3.86758	10.20104	6.46983	30242	81	183	5	2.73224	1.205087	100|3558|5291|5293|7157	ADA|IL2|PIK3CB|PIK3CD|TP53	-2.77024	0	55	0	0	-12.0998	21.55523877
1	-3.84238	GO:0002312	M1	1	0	GO Biological Processes	19	B cell activation involved in immune response	-3.84238	10.07664	6.421737	30242	82	183	5	2.73224	1.205087	100|3558|3569|7040|7099	ADA|IL2|IL6|TGFB1|TLR4	-2.74951	0	55	0	0	-12.0998	21.55523877
1	-3.83632	GO:0032620	M1	1	0	GO Biological	19	interleukin-17	-3.83632	15.02335	7.263155	30242	44	183	4	2.185792	1.080886	3558|3569|7040|7099	IL2|IL6|TGFB1|TLR4	-2.74414	0	55	0	0	-12.0998	21.55523877

						Processes		production																		
1	-3.83632	GO:0032660	M1	1	0	GO Biological Processes	19	regulation of interleukin-17 production	-3.83632	15.02335	7.263155	30242	44	183	4	2.185792	1.080886	3558|3569|7040|7099	IL2|IL6|TGFB1|TLR4	-2.74414	0	55	0	0	-12.0998	21.55523877
1	-3.74931	GO:0002705	M1	1	0	GO Biological Processes	19	positive regulation of leukocyte mediated immunity	-3.74931	7.344748	5.764795	30242	135	183	6	3.278689	1.316393	3558|3569|4843|7040|7124|114548	IL2|IL6|NOS2|TGFB1|TNF|NLRP3	-2.66793	0	55	0	0	-12.0998	21.55523877
1	-3.71458	GO:0050671	M1	1	0	GO Biological Processes	19	positive regulation of lymphocyte proliferation	-3.71458	7.237525	5.709389	30242	137	183	6	3.278689	1.316393	100|596|3558|3569|7099|729230	ADA|BCL2|IL2|IL6|TLR4|CCR2	-2.6372	0	55	0	0	-12.0998	21.55523877
1	-3.71136	GO:0050863	M1	1	0	GO Biological Processes	19	regulation of T cell activation	-3.71136	4.507004	4.998123	30242	330	183	9	4.918033	1.598524	100|135|836|3558|3569|5290|6347|114548|729230	ADA|ADORA2A|CASP3|IL2|IL6|PIK3CA|CCL2|NLRP3|CCR2	-2.6343	0	55	0	0	-12.0998	21.55523877
1	-3.69744	GO:0032946	M1	1	0	GO Biological Processes	19	positive regulation of mononuclear cell proliferation	-3.69744	7.18508	5.682103	30242	138	183	6	3.278689	1.316393	100|596|3558|3569|7099|729230	ADA|BCL2|IL2|IL6|TLR4|CCR2	-2.62334	0	55	0	0	-12.0998	21.55523877
1	-3.5022	GO:0070665	M1	1	0	GO Biological Processes	19	positive regulation of leukocyte proliferation	-3.5022	6.610273	5.37453	30242	150	183	6	3.278689	1.316393	100|596|3558|3569|7099|729230	ADA|BCL2|IL2|IL6|TLR4|CCR2	-2.44798	0	55	0	0	-12.0998	21.55523877
1	-3.47142	GO:0050871	M1	1	0	GO Biological Processes	19	positive regulation of B cell activation	-3.47142	6.523296	5.326542	30242	152	183	6	3.278689	1.316393	100|596|3558|3569|7040|7099	ADA|BCL2|IL2|IL6|TGFB1|TLR4	-2.4197	0	55	0	0	-12.0998	21.55523877
1	-3.45213	GO:0032740	M1	1	0	GO Biological Processes	19	positive regulation of interleukin-17 production	-3.45213	21.55524	7.694542	30242	23	183	3	1.639344	0.938686	3558|3569|7040	IL2|IL6|TGFB1	-2.40475	0	55	0	0	-12.0998	21.55523877
1	-3.45175	GO:0002460	M1	1	0	GO Biological Processes	19	adaptive immune response based on somatic recombination of immune receptors built from immunoglobulin superfamily domains	-3.45175	4.154501	4.68478	30242	358	183	9	4.918033	1.598524	100|3383|3558|3569|7040|7099|7124|114548|729230	ADA|ICAM1|IL2|IL6|TGFB1|TLR4|TNF|NLRP3|CCR2	-2.40468	0	55	0	0	-12.0998	21.55523877
1	-3.44186	GO:0050870	M1	1	0	GO Biological Processes	19	positive regulation of T cell activation	-3.44186	5.355545	5.012531	30242	216	183	7	3.825137	1.417845	100|3558|3569|5290|6347|114548|729230	ADA|IL2|IL6|PIK3CA|CCL2|NLRP3|CCR2	-2.3951	0	55	0	0	-12.0998	21.55523877
1	-3.36119	GO:0046634	M1	1	0	GO Biological Processes	19	regulation of alpha-beta T cell activation	-3.36119	7.94504	5.535675	30242	104	183	5	2.73224	1.205087	100|135|3558|114548|729230	ADA|ADORA2A|IL2|NLRP3|CCR2	-2.32024	0	55	0	0	-12.0998	21.55523877
1	-3.25934	GO:0002260	M1	1	0	GO Biological Processes	19	lymphocyte homeostasis	-3.25934	10.66173	5.94193	30242	62	183	4	2.185792	1.080886	100|596|836|3558	ADA|BCL2|CASP3|IL2	-2.22861	0	55	0	0	-12.0998	21.55523877
1	-3.24094	GO:0002706	M1	1	0	GO Biological Processes	19	regulation of lymphocyte mediated immunity	-3.24094	5.90203	4.971316	30242	168	183	6	3.278689	1.316393	3558|3569|7040|7124|114548|729230	IL2|IL6|TGFB1|TNF|NLRP3|CCR2	-2.21287	0	55	0	0	-12.0998	21.55523877
1	-3.19652	GO:0002708	M1	1	0	GO Biological Processes	19	positive regulation of lymphocyte mediated immunity	-3.19652	7.312249	5.245264	30242	113	183	5	2.73224	1.205087	3558|3569|7040|7124|114548	IL2|IL6|TGFB1|TNF|NLRP3	-2.17227	0	55	0	0	-12.0998	21.55523877
1	-3.19466	GO:0002313	M1	1	0	GO Biological Processes	19	mature B cell differentiation involved in immune response	-3.19466	17.70609	6.900587	30242	28	183	3	1.639344	0.938686	100|3558|3569	ADA|IL2|IL6	-2.17099	0	55	0	0	-12.0998	21.55523877
1	-3.15625	GO:0042093	M1	1	0	GO Biological Processes	19	T-helper cell differentiation	-3.15625	10.01557	5.72098	30242	66	183	4	2.185792	1.080886	3558|3569|5734|114548	IL2|IL6|PTGER4|NLRP3	-2.13694	0	55	0	0	-12.0998	21.55523877
1	-3.10726	GO:0002294	M1	1	0	GO Biological Processes	19	CD4-positive, alpha-beta T cell differentiation involved in immune response	-3.10726	9.72099	5.617462	30242	68	183	4	2.185792	1.080886	3558|3569|5734|114548	IL2|IL6|PTGER4|NLRP3	-2.09198	0	55	0	0	-12.0998	21.55523877

1	-3.09483	GO:0002702	M1	1	0	GO Biological Processes	19	positive regulation of production of molecular mediator of immune response	-3.09483	6.943564	5.06883	30242	119	183	5	2.73224	1.205087	3558|3569|7040|7099|114548	IL2|IL6|TGFB1|TLR4|NLRP3	-2.08212	0	55	0	0	-12.0998	21.55523877
1	-3.08337	GO:0002287	M1	1	0	GO Biological Processes	19	alpha-beta T cell activation involved in immune response	-3.08337	9.580106	5.567296	30242	69	183	4	2.185792	1.080886	3558|3569|5734|114548	IL2|IL6|PTGER4|NLRP3	-2.07151	0	55	0	0	-12.0998	21.55523877
1	-3.08337	GO:0002293	M1	1	0	GO Biological Processes	19	alpha-beta T cell differentiation involved in immune response	-3.08337	9.580106	5.567296	30242	69	183	4	2.185792	1.080886	3558|3569|5734|114548	IL2|IL6|PTGER4|NLRP3	-2.07151	0	55	0	0	-12.0998	21.55523877
1	-3.06307	GO:0001782	M1	1	0	GO Biological Processes	19	B cell homeostasis	-3.06307	15.9926	6.516457	30242	31	183	3	1.639344	0.938686	100|596|836	ADA|BCL2|CASP3	-2.05432	0	55	0	0	-12.0998	21.55523877
1	-3.06307	GO:0002828	M1	1	0	GO Biological Processes	19	regulation of type 2 immune response	-3.06307	15.9926	6.516457	30242	31	183	3	1.639344	0.938686	3569|114548|729230	IL6|NLRP3|CCR2	-2.05432	0	55	0	0	-12.0998	21.55523877
1	-2.98278	GO:0002335	M1	1	0	GO Biological Processes	19	mature B cell differentiation	-2.98278	15.02335	6.288932	30242	33	183	3	1.639344	0.938686	100|3558|3569	ADA|IL2|IL6	-1.98159	0	55	0	0	-12.0998	21.55523877
1	-2.94761	GO:0002292	M1	1	0	GO Biological Processes	19	T cell differentiation involved in immune response	-2.94761	8.813698	5.28637	30242	75	183	4	2.185792	1.080886	3558|3569|5734|114548	IL2|IL6|PTGER4|NLRP3	-1.94919	0	55	0	0	-12.0998	21.55523877
1	-2.87173	GO:0042092	M1	1	0	GO Biological Processes	19	type 2 immune response	-2.87173	13.7714	5.982457	30242	36	183	3	1.639344	0.938686	3569|114548|729230	IL6|NLRP3|CCR2	-1.88231	0	55	0	0	-12.0998	21.55523877
1	-2.83693	GO:0002369	M1	1	0	GO Biological Processes	19	T cell cytokine production	-2.83693	13.3992	5.888322	30242	37	183	3	1.639344	0.938686	3569|114548|729230	IL6|NLRP3|CCR2	-1.8502	0	55	0	0	-12.0998	21.55523877
1	-2.83693	GO:0002724	M1	1	0	GO Biological Processes	19	regulation of T cell cytokine production	-2.83693	13.3992	5.888322	30242	37	183	3	1.639344	0.938686	3569|114548|729230	IL6|NLRP3|CCR2	-1.8502	0	55	0	0	-12.0998	21.55523877
1	-2.83693	GO:0043029	M1	1	0	GO Biological Processes	19	T cell homeostasis	-2.83693	13.3992	5.888322	30242	37	183	3	1.639344	0.938686	596|836|3558	BCL2|CASP3|IL2	-1.8502	0	55	0	0	-12.0998	21.55523877
1	-2.78437	GO:0043367	M1	1	0	GO Biological Processes	19	CD4-positive, alpha-beta T cell differentiation	-2.78437	7.964185	4.957209	30242	83	183	4	2.185792	1.080886	3558|3569|5734|114548	IL2|IL6|PTGER4|NLRP3	-1.8027	0	55	0	0	-12.0998	21.55523877
1	-2.45882	GO:0035710	M1	1	0	GO Biological Processes	19	CD4-positive, alpha-beta T cell activation	-2.45882	6.48066	4.326111	30242	102	183	4	2.185792	1.080886	3558|3569|5734|114548	IL2|IL6|PTGER4|NLRP3	-1.51081	0	55	0	0	-12.0998	21.55523877
1	-2.43567	GO:0002639	M1	1	0	GO Biological Processes	19	positive regulation of immunoglobulin production	-2.43567	9.72099	4.863495	30242	51	183	3	1.639344	0.938686	3558|3569|7040	IL2|IL6|TGFB1	-1.49059	0	55	0	0	-12.0998	21.55523877
1	-2.42864	GO:0045621	M1	1	0	GO Biological Processes	19	positive regulation of lymphocyte differentiation	-2.42864	6.356032	4.269126	30242	104	183	4	2.185792	1.080886	100|3558|5591|114548	ADA|IL2|PRKDC|NLRP3	-1.48429	0	55	0	0	-12.0998	21.55523877
1	-2.37344	GO:0045619	M1	1	0	GO Biological Processes	19	regulation of lymphocyte differentiation	-2.37344	4.74876	3.869424	30242	174	183	5	2.73224	1.205087	100|3558|5591|114548|729230	ADA|IL2|PRKDC|NLRP3|CCR2	-1.43249	0	55	0	0	-12.0998	21.55523877
1	-2.36548	GO:0070228	M1	1	0	GO Biological Processes	19	regulation of lymphocyte apoptotic process	-2.36548	9.180935	4.694821	30242	54	183	3	1.639344	0.938686	100|3558|7157	ADA|IL2|TP53	-1.4267	0	55	0	0	-12.0998	21.55523877
1	-2.32802	GO:0002456	M1	1	0	GO Biological Processes	19	T cell mediated immunity	-2.32802	5.955201	4.080866	30242	111	183	4	2.185792	1.080886	3383|3569|114548|729230	ICAM1|IL6|NLRP3|CCR2	-1.39211	0	55	0	0	-12.0998	21.55523877
1	-2.24341	GO:0002449	M1	1	0	GO Biological Processes	19	lymphocyte mediated immunity	-2.24341	3.29572	3.375504	30242	351	183	7	3.825137	1.417845	3383|3558|3569|7040|7124|114548|729230	ICAM1|IL2|IL6|TGFB1|TNF|NLRP3|CCR2	-1.31319	0	55	0	0	-12.0998	21.55523877
1	-2.12246	GO:0002720	M1	1	0	GO Biological Processes	19	positive regulation of cytokine production	-2.12246	7.511674	4.132094	30242	66	183	3	1.639344	0.938686	3569|7099|114548	IL6|TLR4|NLRP3	-1.20133	0	55	0	0	-12.0998	21.55523877

								involved in immune response																		
1	-2.11312	GO:0042098	M1	1	0	GO Biological Processes	19	T cell proliferation	-2.11312	4.110866	3.452698	30242	201	183	5	2.73224	1.205087	836|3558|3569|7157|729230	CASP3|IL2|IL6|TP53|CCR2	-1.19222	0	55	0	0	-12.0998	21.55523877
1	-2.10448	GO:0046635	M1	1	0	GO Biological Processes	19	positive regulation of alpha-beta T cell activation	-2.10448	7.39956	4.091666	30242	67	183	3	1.639344	0.938686	100|114548|729230	ADA|NLRP3|CCR2	-1.18457	0	55	0	0	-12.0998	21.55523877
1	-2.08679	GO:0046637	M1	1	0	GO Biological Processes	19	regulation of alpha-beta T cell differentiation	-2.08679	7.290743	4.052064	30242	68	183	3	1.639344	0.938686	100|3558|114548	ADA|IL2|NLRP3	-1.16956	0	55	0	0	-12.0998	21.55523877
1	-2.01885	GO:0070227	M1	1	0	GO Biological Processes	19	lymphocyte apoptotic process	-2.01885	6.885701	3.901334	30242	72	183	3	1.639344	0.938686	100|3558|7157	ADA|IL2|TP53	-1.10504	0	55	0	0	-12.0998	21.55523877
1	-11.9019	GO:0014062	M1	1	0	GO Biological Processes	19	regulation of serotonin secretion	-11.9019	123.9426	27.13539	30242	8	183	6	3.278689	1.316393	1268|3350|3351|4129|6532|11255	CNR1|HTR1A|HTR1B|MAOB|SLC6A4|HRH3	-10.0843	0	56	1	1	-11.9019	132.2054645
1	-11.0312	GO:0001820	M1	1	0	GO Biological Processes	19	serotonin secretion	-11.0312	99.1541	24.22208	30242	10	183	6	3.278689	1.316393	1268|3350|3351|4129|6532|11255	CNR1|HTR1A|HTR1B|MAOB|SLC6A4|HRH3	-9.27615	0	56	0	0	-11.9019	132.2054645
1	-10.7167	GO:0006837	M1	1	0	GO Biological Processes	19	serotonin transport	-10.7167	57.83989	19.84006	30242	20	183	7	3.825137	1.417845	1268|3350|3351|4129|4842|6532|11255	CNR1|HTR1A|HTR1B|MAOB|NOS1|SLC6A4|HRH3	-8.98157	0	56	0	0	-11.9019	132.2054645
1	-8.18997	GO:0014063	M1	1	0	GO Biological Processes	19	negative regulation of serotonin secretion	-8.18997	132.2055	22.89306	30242	5	183	4	2.185792	1.080886	1268|3351|4129|11255	CNR1|HTR1B|MAOB|HRH3	-6.63248	0	56	0	0	-11.9019	132.2054645
1	-5.73198	GO:0070050	M1	1	0	GO Biological Processes	19	neuron cellular homeostasis	-5.73198	24.30248	10.60759	30242	34	183	5	2.73224	1.205087	134|1268|3351|3569|6530	ADORA1|CNR1|HTR1B|IL6|SLC6A2	-4.40873	0	56	0	0	-11.9019	132.2054645
1	-4.596	GO:0099171	M1	1	0	GO Biological Processes	19	presynaptic modulation of chemical synaptic transmission	-4.596	49.57705	11.98765	30242	10	183	3	1.639344	0.938686	1268|3351|5582	CNR1|HTR1B|PRKCG	-3.4183	0	56	0	0	-11.9019	132.2054645
1	-3.93862	GO:0099509	M1	1	0	GO Biological Processes	19	regulation of presynaptic cytosolic calcium ion concentration	-3.93862	30.98566	9.360942	30242	16	183	3	1.639344	0.938686	134|1268|3351	ADORA1|CNR1|HTR1B	-2.83536	0	56	0	0	-11.9019	132.2054645
1	-2.87173	GO:0051385	M1	1	0	GO Biological Processes	19	response to mineralocorticoid	-2.87173	13.7714	5.982457	30242	36	183	3	1.639344	0.938686	595|3351|4129	CCND1|HTR1B|MAOB	-1.88231	0	56	0	0	-11.9019	132.2054645
1	-11.7728	GO:0007507	M1	1	0	GO Biological Processes	19	heart development	-11.7728	6.442215	10.41624	30242	590	183	23	12.56831	2.450458	148|775|836|1909|1956|2050|2263|2335|3357|3643|4846|5138|5159|5465|5467|5591|6331|7040|7046|7132|7157|9475|10203	ADRA1A|CACNA1C|CASP3|EDNRA|EGFR|EPHB4|FGFR2|FN1|HTR2B|INSR|NOS3|PDE2A|PDGFRB|PPARA|PPARD|PRKDC|SCN5A|TGFB1|TGFBR1|TNFRSF1A|TP53|ROCK2|CALCRL	-9.96597	0	57	1	1	-11.7728	27.5428051
1	-8.46911	GO:0060485	M1	1	0	GO Biological Processes	19	mesenchyme development	-8.46911	7.923273	9.275574	30242	292	183	14	7.650273	1.964856	596|1909|1910|2263|2335|3066|3357|3569|4846|5159|5979|7040|7046|9475	BCL2|EDNRA|EDNRB|FGFR2|FN1|HDAC2|HTR2B|IL6|NOS3|PDGFRB|RET|TGFB1|TGFBR1|ROCK2	-6.89013	0	57	0	0	-11.7728	27.5428051
1	-7.57452	GO:0048762	M1	1	0	GO Biological Processes	19	mesenchymal cell differentiation	-7.57452	8.332277	8.860824	30242	238	183	12	6.557377	1.829835	596|1909|1910|2263|2335|3066|3357|3569|5979|7040|7046|9475	BCL2|EDNRA|EDNRB|FGFR2|FN1|HDAC2|HTR2B|IL6|RET|TGFB1|TGFBR1|ROCK2	-6.08097	0	57	0	0	-11.7728	27.5428051
1	-5.45718	GO:0048598	M1	1	0	GO Biological Processes	19	embryonic morphogenesis	-5.45718	4.194336	6.119148	30242	591	183	15	8.196721	2.027793	367|775|836|2263|2335|3066|3357|4313|4318|4	AR|CACNA1C|CASP3|FGFR2|FN1|HDAC2|	-4.1632	0	57	0	0	-11.7728	27.5428051

																		325|5979|7040|7046|7157|9475	HTR2B|MMP2|MMP9|MMP16|RET|TGFB1|TGFBR1|TP53|ROCK2							
1	-4.5259	GO:0045165	M1	1	0	GO Biological Processes	19	cell fate commitment	-4.5259	5.764773	5.997133	30242	258	183	9	4.918033	1.598524	367|596|836|2263|3569|5468|5591|7046|7157	AR|BCL2|CASP3|FGFR2|IL6|PPARG|PRKDC|TGFBR1|TP53	-3.35848	0	57	0	0	-11.7728	27.5428051
1	-3.85879	GO:0003007	M1	1	0	GO Biological Processes	19	heart morphogenesis	-3.85879	5.352448	5.359126	30242	247	183	8	4.371585	1.511428	2050|2263|3357|3643|4846|7040|7046|7157	EPHB4|FGFR2|HTR2B|INSR|NOS3|TGFB1|TGFBR1|TP53	-2.76214	0	57	0	0	-11.7728	27.5428051
1	-3.77899	GO:0060391	M1	1	0	GO Biological Processes	19	positive regulation of SMAD protein signal transduction	-3.77899	27.54281	8.789081	30242	18	183	3	1.639344	0.938686	5468|7040|7046	PPARG|TGFB1|TGFBR1	-2.69427	0	57	0	0	-11.7728	27.5428051
1	-3.5529	GO:0010718	M1	1	0	GO Biological Processes	19	positive regulation of epithelial to mesenchymal transition	-3.5529	12.71206	6.595381	30242	52	183	4	2.185792	1.080886	3066|3569|7040|7046	HDAC2|IL6|TGFB1|TGFBR1	-2.49333	0	57	0	0	-11.7728	27.5428051
1	-3.36726	GO:0001837	M1	1	0	GO Biological Processes	19	epithelial to mesenchymal transition	-3.36726	6.236107	5.165149	30242	159	183	6	3.278689	1.316393	2263|3066|3569|7040|7046|9475	FGFR2|HDAC2|IL6|TGFB1|TGFBR1|ROCK2	-2.3257	0	57	0	0	-11.7728	27.5428051
1	-3.33848	GO:0003205	M1	1	0	GO Biological Processes	19	cardiac chamber development	-3.33848	6.15864	5.120806	30242	161	183	6	3.278689	1.316393	2263|5138|6331|7040|7046|7157	FGFR2|PDE2A|SCN5A|TGFB1|TGFBR1|TP53	-2.30086	0	57	0	0	-11.7728	27.5428051
1	-3.06307	GO:0060390	M1	1	0	GO Biological Processes	19	regulation of SMAD protein signal transduction	-3.06307	15.9926	6.516457	30242	31	183	3	1.639344	0.938686	5468|7040|7046	PPARG|TGFB1|TGFBR1	-2.05432	0	57	0	0	-11.7728	27.5428051
1	-3.03025	GO:0003231	M1	1	0	GO Biological Processes	19	cardiac ventricle development	-3.03025	6.717757	4.957861	30242	123	183	5	2.73224	1.205087	2263|5138|6331|7040|7046	FGFR2|PDE2A|SCN5A|TGFB1|TGFBR1	-2.02376	0	57	0	0	-11.7728	27.5428051
1	-2.89337	GO:0072175	M1	1	0	GO Biological Processes	19	epithelial tube formation	-2.89337	6.259728	4.72532	30242	132	183	5	2.73224	1.205087	836|2263|5293|5979|7040	CASP3|FGFR2|PIK3CD|RET|TGFB1	-1.90097	0	57	0	0	-11.7728	27.5428051
1	-2.77031	GO:0003203	M1	1	0	GO Biological Processes	19	endocardial cushion morphogenesis	-2.77031	12.71206	5.710538	30242	39	183	3	1.639344	0.938686	4846|7040|7046	NOS3|TGFB1|TGFBR1	-1.79128	0	57	0	0	-11.7728	27.5428051
1	-2.68773	GO:0016331	M1	1	0	GO Biological Processes	19	morphogenesis of embryonic epithelium	-2.68773	5.620981	4.382097	30242	147	183	5	2.73224	1.205087	367|836|2263|5979|7040	AR|CASP3|FGFR2|RET|TGFB1	-1.7168	0	57	0	0	-11.7728	27.5428051
1	-2.6749	GO:0035148	M1	1	0	GO Biological Processes	19	tube formation	-2.6749	5.583001	4.360911	30242	148	183	5	2.73224	1.205087	836|2263|5293|5979|7040	CASP3|FGFR2|PIK3CD|RET|TGFB1	-1.70578	0	57	0	0	-11.7728	27.5428051
1	-2.56339	GO:0048701	M1	1	0	GO Biological Processes	19	embryonic cranial skeleton morphogenesis	-2.56339	10.77762	5.178139	30242	46	183	3	1.639344	0.938686	2263|4325|7046	FGFR2|MMP16|TGFBR1	-1.6072	0	57	0	0	-11.7728	27.5428051
1	-2.56339	GO:0055010	M1	1	0	GO Biological Processes	19	ventricular cardiac muscle tissue morphogenesis	-2.56339	10.77762	5.178139	30242	46	183	3	1.639344	0.938686	2263|7040|7046	FGFR2|TGFB1|TGFBR1	-1.6072	0	57	0	0	-11.7728	27.5428051
1	-2.55362	GO:0055017	M1	1	0	GO Biological Processes	19	cardiac muscle tissue growth	-2.55362	6.885701	4.506666	30242	96	183	4	2.185792	1.080886	148|2263|5465|7046	ADRA1A|FGFR2|PPARA|TGFBR1	-1.59769	0	57	0	0	-11.7728	27.5428051
1	-2.53666	GO:0003197	M1	1	0	GO Biological Processes	19	endocardial cushion development	-2.53666	10.54831	5.111451	30242	47	183	3	1.639344	0.938686	4846|7040|7046	NOS3|TGFB1|TGFBR1	-1.58173	0	57	0	0	-11.7728	27.5428051
1	-2.5054	GO:0010717	M1	1	0	GO Biological Processes	19	regulation of epithelial to mesenchymal transition	-2.5054	6.677044	4.414515	30242	99	183	4	2.185792	1.080886	3066|3569|7040|7046	HDAC2|IL6|TGFB1|TGFBR1	-1.55246	0	57	0	0	-11.7728	27.5428051
1	-2.48503	GO:0010862	M1	1	0	GO Biological Processes	19	positive regulation of pathway-restricted SMAD protein phosphorylation	-2.48503	10.11777	4.983904	30242	49	183	3	1.639344	0.938686	5468|7040|7046	PPARG|TGFB1|TGFBR1	-1.53506	0	57	0	0	-11.7728	27.5428051

1	-2.44365	GO:0003279	M1	1	0	GO Biological Processes	19	cardiac septum development	-2.44365	6.417741	4.29743	30242	103	183	4	2.185792	1.080886	2263|5138|7046|7157	FGFR2|PDE2A|TGFBR1|TP53	-1.49711	0	57	0	0	-11.7728	27.5428051
1	-2.42864	GO:0060419	M1	1	0	GO Biological Processes	19	heart growth	-2.42864	6.356032	4.269126	30242	104	183	4	2.185792	1.080886	148|2263|5465|7046	ADRA1A|FGFR2|PPARA|TGFBR1	-1.48429	0	57	0	0	-11.7728	27.5428051
1	-2.39911	GO:0046620	M1	1	0	GO Biological Processes	19	regulation of organ growth	-2.39911	6.236107	4.213616	30242	106	183	4	2.185792	1.080886	2263|5465|6532|7046	FGFR2|PPARA|SLC6A4|TGFBR1	-1.45671	0	57	0	0	-11.7728	27.5428051
1	-2.3686	GO:0043009	M1	1	0	GO Biological Processes	19	chordate embryonic development	-2.3686	2.700275	3.315625	30242	612	183	10	5.464481	1.680144	100|367|836|1909|2263|4325|5591|7040|7046|7157	ADA|AR|CASP3|EDNRA|FGFR2|MMP16|PRKDC|TGFB1|TGFBR1|TP53	-1.42837	0	57	0	0	-11.7728	27.5428051
1	-2.34304	GO:0003229	M1	1	0	GO Biological Processes	19	ventricular cardiac muscle tissue development	-2.34304	9.014009	4.641491	30242	55	183	3	1.639344	0.938686	2263|7040|7046	FGFR2|TGFB1|TGFBR1	-1.40593	0	57	0	0	-11.7728	27.5428051
1	-2.34304	GO:0072132	M1	1	0	GO Biological Processes	19	mesenchyme morphogenesis	-2.34304	9.014009	4.641491	30242	55	183	3	1.639344	0.938686	4846|7040|7046	NOS3|TGFB1|TGFBR1	-1.40593	0	57	0	0	-11.7728	27.5428051
1	-2.28713	GO:0090100	M1	1	0	GO Biological Processes	19	positive regulation of transmembrane receptor protein serine/threonine kinase signaling pathway	-2.28713	5.798485	4.005049	30242	114	183	4	2.185792	1.080886	4221|5468|7040|7046	MEN1|PPARG|TGFB1|TGFBR1	-1.35454	0	57	0	0	-11.7728	27.5428051
1	-2.27247	GO:0009792	M1	1	0	GO Biological Processes	19	embryo development ending in birth or egg hatching	-2.27247	2.614823	3.201111	30242	632	183	10	5.464481	1.680144	100|367|836|1909|2263|4325|5591|7040|7046|7157	ADA|AR|CASP3|EDNRA|FGFR2|MMP16|PRKDC|TGFB1|TGFBR1|TP53	-1.34083	0	57	0	0	-11.7728	27.5428051
1	-2.2372	GO:0055008	M1	1	0	GO Biological Processes	19	cardiac muscle tissue morphogenesis	-2.2372	8.262842	4.393843	30242	60	183	3	1.639344	0.938686	2263|7040|7046	FGFR2|TGFB1|TGFBR1	-1.30863	0	57	0	0	-11.7728	27.5428051
1	-2.19759	GO:0060393	M1	1	0	GO Biological Processes	19	regulation of pathway-restricted SMAD protein phosphorylation	-2.19759	7.996298	4.302698	30242	62	183	3	1.639344	0.938686	5468|7040|7046	PPARG|TGFB1|TGFBR1	-1.27182	0	57	0	0	-11.7728	27.5428051
1	-2.19639	GO:0003206	M1	1	0	GO Biological Processes	19	cardiac chamber morphogenesis	-2.19639	5.463036	3.838174	30242	121	183	4	2.185792	1.080886	2263|7040|7046|7157	FGFR2|TGFB1|TGFBR1|TP53	-1.27108	0	57	0	0	-11.7728	27.5428051
1	-2.14076	GO:0060389	M1	1	0	GO Biological Processes	19	pathway-restricted SMAD protein phosphorylation	-2.14076	7.627238	4.173377	30242	65	183	3	1.639344	0.938686	5468|7040|7046	PPARG|TGFB1|TGFBR1	-1.21893	0	57	0	0	-11.7728	27.5428051
1	-2.08679	GO:0060411	M1	1	0	GO Biological Processes	19	cardiac septum morphogenesis	-2.08679	7.290743	4.052064	30242	68	183	3	1.639344	0.938686	2263|7046|7157	FGFR2|TGFBR1|TP53	-1.16956	0	57	0	0	-11.7728	27.5428051
1	-2.08679	GO:1904888	M1	1	0	GO Biological Processes	19	cranial skeletal system development	-2.08679	7.290743	4.052064	30242	68	183	3	1.639344	0.938686	2263|4325|7046	FGFR2|MMP16|TGFBR1	-1.16956	0	57	0	0	-11.7728	27.5428051
1	-2.03543	GO:0003281	M1	1	0	GO Biological Processes	19	ventricular septum development	-2.03543	6.982683	3.937918	30242	71	183	3	1.639344	0.938686	2263|5138|7046	FGFR2|PDE2A|TGFBR1	-1.12071	0	57	0	0	-11.7728	27.5428051
1	-2.03543	GO:0060415	M1	1	0	GO Biological Processes	19	muscle tissue morphogenesis	-2.03543	6.982683	3.937918	30242	71	183	3	1.639344	0.938686	2263|7040|7046	FGFR2|TGFB1|TGFBR1	-1.12071	0	57	0	0	-11.7728	27.5428051
1	-2.01885	GO:0003208	M1	1	0	GO Biological Processes	19	cardiac ventricle morphogenesis	-2.01885	6.885701	3.901334	30242	72	183	3	1.639344	0.938686	2263|7040|7046	FGFR2|TGFB1|TGFBR1	-1.10504	0	57	0	0	-11.7728	27.5428051
1	-2.01885	GO:0055021	M1	1	0	GO Biological Processes	19	regulation of cardiac muscle tissue growth	-2.01885	6.885701	3.901334	30242	72	183	3	1.639344	0.938686	2263|5465|7046	FGFR2|PPARA|TGFBR1	-1.10504	0	57	0	0	-11.7728	27.5428051
1	-11.7485	GO:0032963	M1	1	0	GO Biological Processes	19	collagen metabolic process	-11.7485	19.0681	14.40152	30242	104	183	12	6.557377	1.829835	2147|2149|3066|3569|4312|4313|4314|4318|4325|5159|5467|7040	F2|F2R|HDAC2|IL6|MMP1|MMP2|MMP3|MMP9|MMP16|PDGFRB|PPARD|TGFB1	-9.9434	0	58	1	1	-11.7485	61.97131148

1	-8.22379	GO:0032965	M1	1	0	GO Biological Processes	19	regulation of collagen biosynthetic process	-8.22379	27.54281	13.43088	30242	42	183	7	3.825137	1.417845	2147|2149|3066|3569|5159|5467|7040	F2|F2R|HDAC2|IL6|PDGFRB|PPARD|TGFB1	-6.66529	0	58	0	0	-11.7485	61.97131148
1	-7.86728	GO:0010712	M1	1	0	GO Biological Processes	19	regulation of collagen metabolic process	-7.86728	24.61272	12.64049	30242	47	183	7	3.825137	1.417845	2147|2149|3066|3569|5159|5467|7040	F2|F2R|HDAC2|IL6|PDGFRB|PPARD|TGFB1	-6.34247	0	58	0	0	-11.7485	61.97131148
1	-7.6111	GO:0032964	M1	1	0	GO Biological Processes	19	collagen biosynthetic process	-7.6111	22.68231	12.09172	30242	51	183	7	3.825137	1.417845	2147|2149|3066|3569|5159|5467|7040	F2|F2R|HDAC2|IL6|PDGFRB|PPARD|TGFB1	-6.11496	0	58	0	0	-11.7485	61.97131148
1	-6.25448	GO:0032967	M1	1	0	GO Biological Processes	19	positive regulation of collagen biosynthetic process	-6.25448	30.60312	12.00727	30242	27	183	5	2.73224	1.205087	2147|2149|3066|5159|7040	F2|F2R|HDAC2|PDGFRB|TGFB1	-4.88197	0	58	0	0	-11.7485	61.97131148
1	-6.17118	GO:0010714	M1	1	0	GO Biological Processes	19	positive regulation of collagen metabolic process	-6.17118	29.51015	11.77635	30242	28	183	5	2.73224	1.205087	2147|2149|3066|5159|7040	F2|F2R|HDAC2|PDGFRB|TGFB1	-4.80962	0	58	0	0	-11.7485	61.97131148
1	-5.77441	GO:0090594	M1	1	0	GO Biological Processes	19	inflammatory response to wounding	-5.77441	44.06849	13.01799	30242	15	183	4	2.185792	1.080886	2149|7040|7099|729230	F2R|TGFB1|TLR4|CCR2	-4.44707	0	58	0	0	-11.7485	61.97131148
1	-4.92311	GO:0002246	M1	1	0	GO Biological Processes	19	wound healing involved in inflammatory response	-4.92311	61.97131	13.45734	30242	8	183	3	1.639344	0.938686	2149|7040|7099	F2R|TGFB1|TLR4	-3.69979	0	58	0	0	-11.7485	61.97131148
1	-3.70629	GO:0097709	M1	1	0	GO Biological Processes	19	connective tissue replacement	-3.70629	26.09318	8.536899	30242	19	183	3	1.639344	0.938686	2149|7040|9475	F2R|TGFB1|ROCK2	-2.63055	0	58	0	0	-11.7485	61.97131148
1	-2.83693	GO:0090218	M1	1	0	GO Biological Processes	19	positive regulation of lipid kinase activity	-2.83693	13.3992	5.888322	30242	37	183	3	1.639344	0.938686	2147|5159|7040	F2|PDGFRB|TGFB1	-1.8502	0	58	0	0	-11.7485	61.97131148
1	-2.59076	GO:0035307	M1	1	0	GO Biological Processes	19	positive regulation of protein dephosphorylation	-2.59076	11.01712	5.246911	30242	45	183	3	1.639344	0.938686	134|5159|7040	ADORA1|PDGFRB|TGFB1	-1.63232	0	58	0	0	-11.7485	61.97131148
1	-2.25757	GO:0035306	M1	1	0	GO Biological Processes	19	positive regulation of dephosphorylation	-2.25757	8.40289	4.441017	30242	59	183	3	1.639344	0.938686	134|5159|7040	ADORA1|PDGFRB|TGFB1	-1.32711	0	58	0	0	-11.7485	61.97131148
1	-2.03543	GO:0043550	M1	1	0	GO Biological Processes	19	regulation of lipid kinase activity	-2.03543	6.982683	3.937918	30242	71	183	3	1.639344	0.938686	2147|5159|7040	F2|PDGFRB|TGFB1	-1.12071	0	58	0	0	-11.7485	61.97131148
1	-11.6195	GO:0040008	M1	1	0	GO Biological Processes	19	regulation of growth	-11.6195	6.000248	10.14197	30242	661	183	24	13.11475	2.495327	153|154|185|351|367|596|1813|1956|2100|2147|2263|2335|3558|3643|5290|5465|5467|5591|6197|6531|6532|7040|7046|7157	ADRB1|ADRB2|AGTR1|APP|AR|BCL2|DRD2|EGFR|ESR2|F2|FGFR2|FN1|IL2|INSR|PIK3CA|PPARA|PPARD|PRKDC|RPS6KA3|SLC6A3|SLC6A4|TGFB1|TGFBR1|TP53	-9.81798	0	59	1	1	-11.6195	8.294744393
1	-9.67916	GO:0048638	M1	1	0	GO Biological Processes	19	regulation of developmental growth	-9.67916	8.012452	9.994027	30242	330	183	16	8.743169	2.088053	153|154|351|367|596|1813|2263|2335|3643|5290|5465|5467|5591|6531|6532|7046	ADRB1|ADRB2|APP|AR|BCL2|DRD2|FGFR2|FN1|INSR|PIK3CA|PPARA|PPARD|PRKDC|SLC6A3|SLC6A4|TGFBR1	-8.021	0	59	0	0	-11.6195	8.294744393
1	-9.58007	GO:0048589	M1	1	0	GO Biological Processes	19	developmental growth	-9.58007	5.543759	8.963459	30242	626	183	21	11.47541	2.356083	148|153|154|351|367|596|1813|2099|2263|2335|3643|5159|5290|5465|5467|5591|6531|6532|7046|7157|7298	ADRA1A|ADRB1|ADRB2|APP|AR|BCL2|DRD2|ESR1|FGFR2|FN1|INSR|PDGFRB|PIK3CA|PPARA|PPARD|PRKDC|SLC6A3|SLC6A	-7.93063	0	59	0	0	-11.6195	8.294744393

<table>
<tr><td></td><td></td><td></td><td></td><td></td><td></td><td></td><td></td><td></td><td></td><td></td><td></td><td></td><td></td><td></td><td></td><td></td><td></td><td></td><td>4|TGFBR1|TP53|TYMS</td><td></td><td></td><td></td><td></td><td></td><td></td><td></td></tr>
<tr><td>1</td><td>-8.13828</td><td>GO:0045927</td><td>M1</td><td>1</td><td>0</td><td>GO Biological Processes</td><td>19</td><td>positive regulation of growth</td><td>-8.13828</td><td>8.294744</td><td>9.199385</td><td>30242</td><td>259</td><td>183</td><td>13</td><td>7.103825</td><td>1.898976</td><td>596|1813|1956|2147|2263|2335|3558|3643|5467|5591|6197|6531|7046</td><td>BCL2|DRD2|EGFR|F2|FGFR2|FN1|IL2|INSR|PPARD|PRKDC|RPS6KA3|SLC6A3|TGFBR1</td><td>-6.59131</td><td>0</td><td>59</td><td>0</td><td>0</td><td>-11.6195</td><td>8.294744393</td></tr>
<tr><td>1</td><td>-6.52072</td><td>GO:0016049</td><td>M1</td><td>1</td><td>0</td><td>GO Biological Processes</td><td>19</td><td>cell growth</td><td>-6.52072</td><td>5.111036</td><td>7.121376</td><td>30242</td><td>485</td><td>183</td><td>15</td><td>8.196721</td><td>2.027793</td><td>148|185|351|596|1956|2100|2147|2335|3558|5465|5467|6197|7040|7046|7157</td><td>ADRA1A|AGTR1|APP|BCL2|EGFR|ESR2|F2|FN1|IL2|PPARA|PPARD|RPS6KA3|TGFB1|TGFBR1|TP53</td><td>-5.13091</td><td>0</td><td>59</td><td>0</td><td>0</td><td>-11.6195</td><td>8.294744393</td></tr>
<tr><td>1</td><td>-5.75784</td><td>GO:0001558</td><td>M1</td><td>1</td><td>0</td><td>GO Biological Processes</td><td>19</td><td>regulation of cell growth</td><td>-5.75784</td><td>5.151892</td><td>6.661318</td><td>30242</td><td>417</td><td>183</td><td>13</td><td>7.103825</td><td>1.898976</td><td>185|596|1956|2100|2147|2335|3558|5465|5467|6197|7040|7046|7157</td><td>AGTR1|BCL2|EGFR|ESR2|F2|FN1|IL2|PPARA|PPARD|RPS6KA3|TGFB1|TGFBR1|TP53</td><td>-4.43226</td><td>0</td><td>59</td><td>0</td><td>0</td><td>-11.6195</td><td>8.294744393</td></tr>
<tr><td>1</td><td>-4.93499</td><td>GO:0048639</td><td>M1</td><td>1</td><td>0</td><td>GO Biological Processes</td><td>19</td><td>positive regulation of developmental growth</td><td>-4.93499</td><td>7.598015</td><td>6.810391</td><td>30242</td><td>174</td><td>183</td><td>8</td><td>4.371585</td><td>1.511428</td><td>596|1813|2263|2335|3643|5467|5591|6531</td><td>BCL2|DRD2|FGFR2|FN1|INSR|PPARD|PRKDC|SLC6A3</td><td>-3.70734</td><td>0</td><td>59</td><td>0</td><td>0</td><td>-11.6195</td><td>8.294744393</td></tr>
<tr><td>1</td><td>-4.14564</td><td>GO:0030307</td><td>M1</td><td>1</td><td>0</td><td>GO Biological Processes</td><td>19</td><td>positive regulation of cell growth</td><td>-4.14564</td><td>6.968662</td><td>6.016686</td><td>30242</td><td>166</td><td>183</td><td>7</td><td>3.825137</td><td>1.417845</td><td>596|1956|2147|2335|3558|6197|7046</td><td>BCL2|EGFR|F2|FN1|IL2|RPS6KA3|TGFBR1</td><td>-3.01747</td><td>0</td><td>59</td><td>0</td><td>0</td><td>-11.6195</td><td>8.294744393</td></tr>
<tr><td>1</td><td>-11.5084</td><td>GO:0051953</td><td>M1</td><td>1</td><td>0</td><td>GO Biological Processes</td><td>19</td><td>negative regulation of amine transport</td><td>-11.5084</td><td>48.96499</td><td>19.455</td><td>30242</td><td>27</td><td>183</td><td>8</td><td>4.371585</td><td>1.511428</td><td>134|150|151|152|1268|1813|3351|11255</td><td>ADORA1|ADRA2A|ADRA2B|ADRA2C|CNR1|DRD2|HTR1B|HRH3</td><td>-9.71039</td><td>0</td><td>60</td><td>1</td><td>1</td><td>-11.5084</td><td>123.942623</td></tr>
<tr><td>1</td><td>-11.4783</td><td>GO:0010469</td><td>M1</td><td>1</td><td>0</td><td>GO Biological Processes</td><td>19</td><td>regulation of signaling receptor activity</td><td>-11.4783</td><td>13.37339</td><td>12.73469</td><td>30242</td><td>173</td><td>183</td><td>14</td><td>7.650273</td><td>1.964856</td><td>134|150|151|152|154|351|2100|3066|4988|5465|5468|6347|7124|729230</td><td>ADORA1|ADRA2A|ADRA2B|ADRA2C|ADRB2|APP|ESR2|HDAC2|OPRM1|PPARA|PPARG|CCL2|TNF|CCR2</td><td>-9.68367</td><td>0</td><td>60</td><td>0</td><td>0</td><td>-11.5084</td><td>123.942623</td></tr>
<tr><td>1</td><td>-9.93528</td><td>GO:0015874</td><td>M1</td><td>1</td><td>0</td><td>GO Biological Processes</td><td>19</td><td>norepinephrine transport</td><td>-9.93528</td><td>46.27191</td><td>17.6689</td><td>30242</td><td>25</td><td>183</td><td>7</td><td>3.825137</td><td>1.417845</td><td>135|150|151|152|6530|6531|11255</td><td>ADORA2A|ADRA2A|ADRA2B|ADRA2C|SLC6A2|SLC6A3|HRH3</td><td>-8.25649</td><td>0</td><td>60</td><td>0</td><td>0</td><td>-11.5084</td><td>123.942623</td></tr>
<tr><td>1</td><td>-9.56153</td><td>GO:2000273</td><td>M1</td><td>1</td><td>0</td><td>GO Biological Processes</td><td>19</td><td>positive regulation of signaling receptor activity</td><td>-9.56153</td><td>29.37899</td><td>14.86475</td><td>30242</td><td>45</td><td>183</td><td>8</td><td>4.371585</td><td>1.511428</td><td>134|150|151|152|154|3066|6347|729230</td><td>ADORA1|ADRA2A|ADRA2B|ADRA2C|ADRB2|HDAC2|CCL2|CCR2</td><td>-7.91342</td><td>0</td><td>60</td><td>0</td><td>0</td><td>-11.5084</td><td>123.942623</td></tr>
<tr><td>1</td><td>-7.83233</td><td>GO:0033604</td><td>M1</td><td>1</td><td>0</td><td>GO Biological Processes</td><td>19</td><td>negative regulation of catecholamine secretion</td><td>-7.83233</td><td>59.0203</td><td>16.94243</td><td>30242</td><td>14</td><td>183</td><td>5</td><td>2.73224</td><td>1.205087</td><td>150|151|152|1268|1813</td><td>ADRA2A|ADRA2B|ADRA2C|CNR1|DRD2</td><td>-6.31304</td><td>0</td><td>60</td><td>0</td><td>0</td><td>-11.5084</td><td>123.942623</td></tr>
<tr><td>1</td><td>-7.49778</td><td>GO:0014061</td><td>M1</td><td>1</td><td>0</td><td>GO Biological Processes</td><td>19</td><td>regulation of norepinephrine secretion</td><td>-7.49778</td><td>51.64276</td><td>15.80969</td><td>30242</td><td>16</td><td>183</td><td>5</td><td>2.73224</td><td>1.205087</td><td>135|150|151|152|11255</td><td>ADORA2A|ADRA2A|ADRA2B|ADRA2C|HRH3</td><td>-6.01022</td><td>0</td><td>60</td><td>0</td><td>0</td><td>-11.5084</td><td>123.942623</td></tr>
<tr><td>1</td><td>-7.34865</td><td>GO:0048243</td><td>M1</td><td>1</td><td>0</td><td>GO Biological Processes</td><td>19</td><td>norepinephrine secretion</td><td>-7.34865</td><td>48.60495</td><td>15.31898</td><td>30242</td><td>17</td><td>183</td><td>5</td><td>2.73224</td><td>1.205087</td><td>135|150|151|152|11255</td><td>ADORA2A|ADRA2A|ADRA2B|ADRA2C|HRH3</td><td>-5.87776</td><td>0</td><td>60</td><td>0</td><td>0</td><td>-11.5084</td><td>123.942623</td></tr>
<tr><td>1</td><td>-6.06147</td><td>GO:0032811</td><td>M1</td><td>1</td><td>0</td><td>GO Biological Processes</td><td>19</td><td>negative regulation of epinephrine secretion</td><td>-6.06147</td><td>123.9426</td><td>19.18635</td><td>30242</td><td>4</td><td>183</td><td>3</td><td>1.639344</td><td>0.938686</td><td>150|151|152</td><td>ADRA2A|ADRA2B|ADRA2C</td><td>-4.70747</td><td>0</td><td>60</td><td>0</td><td>0</td><td>-11.5084</td><td>123.942623</td></tr>
<tr><td>1</td><td>-5.66547</td><td>GO:0035624</td><td>M1</td><td>1</td><td>0</td><td>GO Biological Processes</td><td>19</td><td>receptor transactivation</td><td>-5.66547</td><td>99.1541</td><td>17.12618</td><td>30242</td><td>5</td><td>183</td><td>3</td><td>1.639344</td><td>0.938686</td><td>150|151|152</td><td>ADRA2A|ADRA2B|ADRA2C</td><td>-4.35145</td><td>0</td><td>60</td><td>0</td><td>0</td><td>-11.5084</td><td>123.942623</td></tr>
<tr><td>1</td><td>-5.36639</td><td>GO:0014060</td><td>M1</td><td>1</td><td>0</td><td>GO Biological</td><td>19</td><td>regulation of</td><td>-5.36639</td><td>82.62842</td><td>15.60239</td><td>30242</td><td>6</td><td>183</td><td>3</td><td>1.639344</td><td>0.938686</td><td>150|151|152</td><td>ADRA2A|ADRA2B|A</td><td>-4.08918</td><td>0</td><td>60</td><td>0</td><td>0</td><td>-11.5084</td><td>123.942623</td></tr>
</table>

						Processes		epinephrine secretion											DRA2C							
1	-5.12529	GO:0048242	M1	1	0	GO Biological Processes	19	epinephrine secretion	-5.12529	70.82436	14.41575	30242	7	183	3	1.639344	0.938686	150|151|152	ADRA2A|ADRA2B|ADRA2C	-3.87821	0	60	0	0	-11.5084	123.942623
1	-4.92311	GO:0010700	M1	1	0	GO Biological Processes	19	negative regulation of norepinephrine secretion	-4.92311	61.97131	13.45734	30242	8	183	3	1.639344	0.938686	150|151|152	ADRA2A|ADRA2B|ADRA2C	-3.69979	0	60	0	0	-11.5084	123.942623
1	-4.7398	GO:0032147	M1	1	0	GO Biological Processes	19	activation of protein kinase activity	-4.7398	8.63282	6.90928	30242	134	183	7	3.825137	1.417845	150|151|152|154|1813|3643|5290	ADRA2A|ADRA2B|ADRA2C|ADRB2|DRD2|INSR|PIK3CA	-3.54033	0	60	0	0	-11.5084	123.942623
1	-4.596	GO:0048241	M1	1	0	GO Biological Processes	19	epinephrine transport	-4.596	49.57705	11.98765	30242	10	183	3	1.639344	0.938686	150|151|152	ADRA2A|ADRA2B|ADRA2C	-3.4183	0	60	0	0	-11.5084	123.942623
1	-4.44459	GO:0032148	M1	1	0	GO Biological Processes	19	activation of protein kinase B activity	-4.44459	21.32346	8.83349	30242	31	183	4	2.185792	1.080886	150|151|152|3643	ADRA2A|ADRA2B|ADRA2C|INSR	-3.29079	0	60	0	0	-11.5084	123.942623
1	-11.4788	GO:0051090	M1	1	0	GO Biological Processes	19	regulation of DNA-binding transcription factor activity	-11.4788	7.511674	10.73576	30242	440	183	20	10.92896	2.306387	140|351|367|1545|2099|2100|3066|3383|3551|3569|4221|4792|4914|4985|5468|5970|7099|7124|8986|114548	ADORA3|APP|AR|CYP1B1|ESR1|ESR2|HDAC2|ICAM1|IKBKB|IL6|MEN1|NFKBIA|NTRK1|OPRD1|PPARG|RELA|TLR4|TNF|RPS6KA4|NLRP3	-9.68367	0	61	1	1	-11.4788	22.03424408
1	-10.1541	GO:0051091	M1	1	0	GO Biological Processes	19	positive regulation of DNA-binding transcription factor activity	-10.1541	9.534048	10.7832	30242	260	183	15	8.196721	2.027793	351|367|2099|2100|3383|3551|3569|4914|4985|5468|5970|7099|7124|8986|114548	APP|AR|ESR1|ESR2|ICAM1|IKBKB|IL6|NTRK1|OPRD1|PPARG|RELA|TLR4|TNF|RPS6KA4|NLRP3	-8.45502	0	61	0	0	-11.4788	22.03424408
1	-8.58373	GO:0051092	M1	1	0	GO Biological Processes	19	positive regulation of NF-kappaB transcription factor activity	-8.58373	11.88121	10.52823	30242	153	183	11	6.010929	1.757049	351|367|3383|3551|3569|4914|5970|7099|7124|8986|114548	APP|AR|ICAM1|IKBKB|IL6|NTRK1|RELA|TLR4|TNF|RPS6KA4|NLRP3	-6.9952	0	61	0	0	-11.4788	22.03424408
1	-6.62474	GO:0070555	M1	1	0	GO Biological Processes	19	response to interleukin-1	-6.62474	10.40078	8.792092	30242	143	183	9	4.918033	1.598524	351|3383|3551|3569|3576|5970|6347|6401|8986	APP|ICAM1|IKBKB|IL6|CXCL8|RELA|CCL2|SELE|RPS6KA4	-5.22602	0	61	0	0	-11.4788	22.03424408
1	-5.22483	GO:0071347	M1	1	0	GO Biological Processes	19	cellular response to interleukin-1	-5.22483	10.23715	7.675756	30242	113	183	7	3.825137	1.417845	3383|3551|3569|3576|5970|6347|8986	ICAM1|IKBKB|IL6|CXCL8|RELA|CCL2|RPS6KA4	-3.96486	0	61	0	0	-11.4788	22.03424408
1	-4.50254	GO:0070498	M1	1	0	GO Biological Processes	19	interleukin-1-mediated signaling pathway	-4.50254	22.03424	8.993612	30242	30	183	4	2.185792	1.080886	3551|3569|5970|8986	IKBKB|IL6|RELA|RPS6KA4	-3.33837	0	61	0	0	-11.4788	22.03424408
1	-2.59076	GO:0071354	M1	1	0	GO Biological Processes	19	cellular response to interleukin-6	-2.59076	11.01712	5.246911	30242	45	183	3	1.639344	0.938686	3383|3569|5970	ICAM1|IL6|RELA	-1.63232	0	61	0	0	-11.4788	22.03424408
1	-2.48503	GO:0070741	M1	1	0	GO Biological Processes	19	response to interleukin-6	-2.48503	10.11777	4.983904	30242	49	183	3	1.639344	0.938686	3383|3569|5970	ICAM1|IL6|RELA	-1.53506	0	61	0	0	-11.4788	22.03424408
1	-11.371	GO:0006970	M1	1	0	GO Biological Processes	19	response to osmotic stress	-11.371	21.38618	14.68546	30242	85	183	11	6.010929	1.757049	624|836|1956|3778|5243|5743|6326|6513|7124|7157|59341	BDKRB2|CASP3|EGFR|KCNMA1|ABCB1|PTGS2|SCN2A|SLC2A1|TNF|TP53|TRPV4	-9.58323	0	62	1	1	-11.371	82.6284153
1	-7.05003	GO:0008627	M1	1	0	GO Biological Processes	19	intrinsic apoptotic signaling pathway in response to osmotic stress	-7.05003	82.62842	18.01669	30242	8	183	4	2.185792	1.080886	624|836|5743|6326	BDKRB2|CASP3|PTGS2|SCN2A	-5.61068	0	62	0	0	-11.371	82.6284153
1	-6.70343	GO:0071470	M1	1	0	GO Biological Processes	19	cellular response to osmotic stress	-6.70343	23.60812	11.43989	30242	42	183	6	3.278689	1.316393	624|836|5743|6326|6513|59341	BDKRB2|CASP3|PTGS2|SCN2A|SLC2A1|T	-5.29491	0	62	0	0	-11.371	82.6284153

<table>
<tr><td></td><td></td><td></td><td></td><td></td><td></td><td></td><td></td><td></td><td></td><td></td><td></td><td></td><td></td><td></td><td></td><td></td><td></td><td></td><td>RPV4</td><td></td><td></td><td></td><td></td><td></td><td></td><td></td></tr>
<tr><td>1</td><td>-5.90705</td><td>GO:0047484</td><td>M1</td><td>1</td><td>0</td><td>GO Biological Processes</td><td>19</td><td>regulation of response to osmotic stress</td><td>-5.90705</td><td>47.21624</td><td>13.49554</td><td>30242</td><td>14</td><td>183</td><td>4</td><td>2.185792</td><td>1.080886</td><td>624|5243|5743|59341</td><td>BDKRB2|ABCB1|PTGS2|TRPV4</td><td>-4.56779</td><td>0</td><td>62</td><td>0</td><td>0</td><td>-11.371</td><td>82.6284153</td></tr>
<tr><td>1</td><td>-11.3003</td><td>GO:0007612</td><td>M1</td><td>1</td><td>0</td><td>GO Biological Processes</td><td>19</td><td>learning</td><td>-11.3003</td><td>14.91902</td><td>13.06344</td><td>30242</td><td>144</td><td>183</td><td>13</td><td>7.103825</td><td>1.898976</td><td>351|1812|1813|1814|2558|2902|2903|2915|3156|3643|4915|4986|5743</td><td>APP|DRD1|DRD2|DRD3|GABRA5|GRIN1|GRIN2A|GRM5|HMGCR|INSR|NTRK2|OPRK1|PTGS2</td><td>-9.51928</td><td>0</td><td>63</td><td>1</td><td>1</td><td>-11.3003</td><td>26.29085941</td></tr>
<tr><td>1</td><td>-10.5856</td><td>GO:0030534</td><td>M1</td><td>1</td><td>0</td><td>GO Biological Processes</td><td>19</td><td>adult behavior</td><td>-10.5856</td><td>15.25448</td><td>12.70836</td><td>30242</td><td>130</td><td>183</td><td>12</td><td>6.557377</td><td>1.829835</td><td>351|1812|1813|1814|1815|2566|3066|3356|4985|4986|4988|5465</td><td>APP|DRD1|DRD2|DRD3|DRD4|GABRG2|HDAC2|HTR2A|OPRD1|OPRK1|OPRM1|PPARA</td><td>-8.8594</td><td>0</td><td>63</td><td>0</td><td>0</td><td>-11.3003</td><td>26.29085941</td></tr>
<tr><td>1</td><td>-8.94426</td><td>GO:0008306</td><td>M1</td><td>1</td><td>0</td><td>GO Biological Processes</td><td>19</td><td>associative learning</td><td>-8.94426</td><td>19.0681</td><td>12.4667</td><td>30242</td><td>78</td><td>183</td><td>9</td><td>4.918033</td><td>1.598524</td><td>351|1812|1813|1814|2558|2902|2903|3156|4986</td><td>APP|DRD1|DRD2|DRD3|GABRA5|GRIN1|GRIN2A|HMGCR|OPRK1</td><td>-7.33261</td><td>0</td><td>63</td><td>0</td><td>0</td><td>-11.3003</td><td>26.29085941</td></tr>
<tr><td>1</td><td>-8.0758</td><td>GO:0008542</td><td>M1</td><td>1</td><td>0</td><td>GO Biological Processes</td><td>19</td><td>visual learning</td><td>-8.0758</td><td>26.29086</td><td>13.09897</td><td>30242</td><td>44</td><td>183</td><td>7</td><td>3.825137</td><td>1.417845</td><td>351|1812|1813|1814|2902|2903|3156</td><td>APP|DRD1|DRD2|DRD3|GRIN1|GRIN2A|HMGCR</td><td>-6.53399</td><td>0</td><td>63</td><td>0</td><td>0</td><td>-11.3003</td><td>26.29085941</td></tr>
<tr><td>1</td><td>-7.84411</td><td>GO:0007626</td><td>M1</td><td>1</td><td>0</td><td>GO Biological Processes</td><td>19</td><td>locomotory behavior</td><td>-7.84411</td><td>10.09903</td><td>9.553416</td><td>30242</td><td>180</td><td>183</td><td>11</td><td>6.010929</td><td>1.757049</td><td>135|351|1812|1813|1814|1815|2915|3358|4985|4986|6531</td><td>ADORA2A|APP|DRD1|DRD2|DRD3|DRD4|GRM5|HTR2C|OPRD1|OPRK1|SLC6A3</td><td>-6.32299</td><td>0</td><td>63</td><td>0</td><td>0</td><td>-11.3003</td><td>26.29085941</td></tr>
<tr><td>1</td><td>-7.67301</td><td>GO:0007632</td><td>M1</td><td>1</td><td>0</td><td>GO Biological Processes</td><td>19</td><td>visual behavior</td><td>-7.67301</td><td>23.13596</td><td>12.22288</td><td>30242</td><td>50</td><td>183</td><td>7</td><td>3.825137</td><td>1.417845</td><td>351|1812|1813|1814|2902|2903|3156</td><td>APP|DRD1|DRD2|DRD3|GRIN1|GRIN2A|HMGCR</td><td>-6.16899</td><td>0</td><td>63</td><td>0</td><td>0</td><td>-11.3003</td><td>26.29085941</td></tr>
<tr><td>1</td><td>-4.62504</td><td>GO:0019098</td><td>M1</td><td>1</td><td>0</td><td>GO Biological Processes</td><td>19</td><td>reproductive behavior</td><td>-4.62504</td><td>23.60812</td><td>9.338469</td><td>30242</td><td>28</td><td>183</td><td>4</td><td>2.185792</td><td>1.080886</td><td>351|1812|3066|4986</td><td>APP|DRD1|HDAC2|OPRK1</td><td>-3.43978</td><td>0</td><td>63</td><td>0</td><td>0</td><td>-11.3003</td><td>26.29085941</td></tr>
<tr><td>1</td><td>-4.1404</td><td>GO:0008344</td><td>M1</td><td>1</td><td>0</td><td>GO Biological Processes</td><td>19</td><td>adult locomotory behavior</td><td>-4.1404</td><td>11.6378</td><td>7.00201</td><td>30242</td><td>71</td><td>183</td><td>5</td><td>2.73224</td><td>1.205087</td><td>351|1812|1813|1815|4985</td><td>APP|DRD1|DRD2|DRD4|OPRD1</td><td>-3.01298</td><td>0</td><td>63</td><td>0</td><td>0</td><td>-11.3003</td><td>26.29085941</td></tr>
<tr><td>1</td><td>-3.76048</td><td>GO:1900271</td><td>M1</td><td>1</td><td>0</td><td>GO Biological Processes</td><td>19</td><td>regulation of long-term synaptic potentiation</td><td>-3.76048</td><td>14.37016</td><td>7.080715</td><td>30242</td><td>46</td><td>183</td><td>4</td><td>2.185792</td><td>1.080886</td><td>134|135|351|1813</td><td>ADORA1|ADORA2A|APP|DRD2</td><td>-2.67844</td><td>0</td><td>63</td><td>0</td><td>0</td><td>-11.3003</td><td>26.29085941</td></tr>
<tr><td>1</td><td>-3.57263</td><td>GO:0007617</td><td>M1</td><td>1</td><td>0</td><td>GO Biological Processes</td><td>19</td><td>mating behavior</td><td>-3.57263</td><td>23.60812</td><td>8.086415</td><td>30242</td><td>21</td><td>183</td><td>3</td><td>1.639344</td><td>0.938686</td><td>351|1812|3066</td><td>APP|DRD1|HDAC2</td><td>-2.51178</td><td>0</td><td>63</td><td>0</td><td>0</td><td>-11.3003</td><td>26.29085941</td></tr>
<tr><td>1</td><td>-3.5109</td><td>GO:0048169</td><td>M1</td><td>1</td><td>0</td><td>GO Biological Processes</td><td>19</td><td>regulation of long-term neuronal synaptic plasticity</td><td>-3.5109</td><td>22.53502</td><td>7.883985</td><td>30242</td><td>22</td><td>183</td><td>3</td><td>1.639344</td><td>0.938686</td><td>351|1813|2915</td><td>APP|DRD2|GRM5</td><td>-2.45542</td><td>0</td><td>63</td><td>0</td><td>0</td><td>-11.3003</td><td>26.29085941</td></tr>
<tr><td>1</td><td>-3.45213</td><td>GO:1900273</td><td>M1</td><td>1</td><td>0</td><td>GO Biological Processes</td><td>19</td><td>positive regulation of long-term synaptic potentiation</td><td>-3.45213</td><td>21.55524</td><td>7.694542</td><td>30242</td><td>23</td><td>183</td><td>3</td><td>1.639344</td><td>0.938686</td><td>135|351|1813</td><td>ADORA2A|APP|DRD2</td><td>-2.40475</td><td>0</td><td>63</td><td>0</td><td>0</td><td>-11.3003</td><td>26.29085941</td></tr>
<tr><td>1</td><td>-2.48503</td><td>GO:0048168</td><td>M1</td><td>1</td><td>0</td><td>GO Biological Processes</td><td>19</td><td>regulation of neuronal synaptic plasticity</td><td>-2.48503</td><td>10.11777</td><td>4.983904</td><td>30242</td><td>49</td><td>183</td><td>3</td><td>1.639344</td><td>0.938686</td><td>351|1813|2915</td><td>APP|DRD2|GRM5</td><td>-1.53506</td><td>0</td><td>63</td><td>0</td><td>0</td><td>-11.3003</td><td>26.29085941</td></tr>
<tr><td>1</td><td>-11.2684</td><td>GO:0048514</td><td>M1</td><td>1</td><td>0</td><td>GO Biological Processes</td><td>19</td><td>blood vessel morphogenesis</td><td>-11.2684</td><td>5.773164</td><td>9.874284</td><td>30242</td><td>687</td><td>183</td><td>24</td><td>13.11475</td><td>2.495327</td><td>185|1545|1909|2050|2263|2335|3569|3576|4313|4846|4915|5159|5290|5291|5293|5468|5743|6347|7040|7046|7124|9475|10203|729230</td><td>AGTR1|CYP1B1|EDNRA|EPHB4|FGFR2|FN1|IL6|CXCL8|MMP2|NOS3|NTRK2|PDGFRB|PIK3CA|PIK3CB|PIK3CD|PPARG|PTGS2|CCL2|TGFB1|TGFBR1|TNF|ROCK2|CALCRL|</td><td>-9.49071</td><td>0</td><td>64</td><td>1</td><td>1</td><td>-11.2684</td><td>7.399559579</td></tr>
</table>

																			CCR2							
1	-11.1011	GO:0001568	M1	1	0	GO Biological Processes	19	blood vessel development	-11.1011	5.40055	9.619055	30242	765	183	25	13.6612	2.538762	185|1545|1909|2050|2263|2335|3569|3576|4313|4846|4915|5138|5159|5290|5291|5293|5468|5743|6347|7040|7046|7124|9475|10203|729230	AGTR1|CYP1B1|EDNRA|EPHB4|FGFR2|FN1|IL6|CXCL8|MMP2|NOS3|NTRK2|PDE2A|PDGFRB|PIK3CA|PIK3CB|PIK3CD|PPARG|PTGS2|CCL2|TGFB1|TGFBR1|TNF|ROCK2|CALCRL|CCR2	-9.33907	0	64	0	0	-11.2684	7.399559579
1	-10.8567	GO:0001525	M1	1	0	GO Biological Processes	19	angiogenesis	-10.8567	6.130945	9.845914	30242	593	183	22	12.02186	2.404073	185|1545|1909|2050|2263|2335|3569|3576|4313|4846|5159|5290|5291|5293|5468|5743|6347|7046|7124|9475|10203|729230	AGTR1|CYP1B1|EDNRA|EPHB4|FGFR2|FN1|IL6|CXCL8|MMP2|NOS3|PDGFRB|PIK3CA|PIK3CB|PIK3CD|PPARG|PTGS2|CCL2|TGFBR1|TNF|ROCK2|CALCRL|CCR2	-9.11093	0	64	0	0	-11.2684	7.399559579
1	-9.05696	GO:0001667	M1	1	0	GO Biological Processes	19	ameboidal-type cell migration	-9.05696	6.223061	8.981104	30242	478	183	18	9.836066	2.201413	1545|1910|2050|2335|3357|4318|4846|5290|5291|5293|5467|5468|5743|5979|7040|7046|7124|9475	CYP1B1|EDNRB|EPHB4|FN1|HTR2B|MMP9|NOS3|PIK3CA|PIK3CB|PIK3CD|PPARD|PPARG|PTGS2|RET|TGFB1|TGFBR1|TNF|ROCK2	-7.43616	0	64	0	0	-11.2684	7.399559579
1	-7.33963	GO:0010631	M1	1	0	GO Biological Processes	19	epithelial cell migration	-7.33963	6.444556	8.097183	30242	359	183	14	7.650273	1.964856	1545|2050|4318|4846|5290|5291|5293|5467|5468|5743|7040|7046|7124|9475	CYP1B1|EPHB4|MMP9|NOS3|PIK3CA|PIK3CB|PIK3CD|PPARD|PPARG|PTGS2|TGFB1|TGFBR1|TNF|ROCK2	-5.87037	0	64	0	0	-11.2684	7.399559579
1	-7.29492	GO:0090132	M1	1	0	GO Biological Processes	19	epithelium migration	-7.29492	6.391148	8.051589	30242	362	183	14	7.650273	1.964856	1545|2050|4318|4846|5290|5291|5293|5467|5468|5743|7040|7046|7124|9475	CYP1B1|EPHB4|MMP9|NOS3|PIK3CA|PIK3CB|PIK3CD|PPARD|PPARG|PTGS2|TGFB1|TGFBR1|TNF|ROCK2	-5.83133	0	64	0	0	-11.2684	7.399559579
1	-7.22137	GO:0090130	M1	1	0	GO Biological Processes	19	tissue migration	-7.22137	6.304075	7.976734	30242	367	183	14	7.650273	1.964856	1545|2050|4318|4846|5290|5291|5293|5467|5468|5743|7040|7046|7124|9475	CYP1B1|EPHB4|MMP9|NOS3|PIK3CA|PIK3CB|PIK3CD|PPARD|PPARG|PTGS2|TGFB1|TGFBR1|TNF|ROCK2	-5.76811	0	64	0	0	-11.2684	7.399559579
1	-6.78764	GO:0043542	M1	1	0	GO Biological Processes	19	endothelial cell migration	-6.78764	7.057231	7.959495	30242	281	183	12	6.557377	1.829835	1545|2050|4846|5290|5291|5293|5468|5743|7040|7046|7124|9475	CYP1B1|EPHB4|NOS3|PIK3CA|PIK3CB|PIK3CD|PPARG|PTGS2|TGFB1|TGFBR1|TNF|ROCK2	-5.37126	0	64	0	0	-11.2684	7.399559579
1	-4.29549	GO:0045765	M1	1	0	GO Biological Processes	19	regulation of angiogenesis	-4.29549	4.803978	5.536464	30242	344	183	10	5.464481	1.680144	1545|3569|3576|4846|5291|5293|5468|7124|9475|729230	CYP1B1|IL6|CXCL8|NOS3|PIK3CB|PIK3CD|PPARG|TNF|ROCK2|CCR2	-3.15297	0	64	0	0	-11.2684	7.399559579
1	-4.2329	GO:1901342	M1	1	0	GO Biological Processes	19	regulation of vasculature	-4.2329	4.721624	5.464184	30242	350	183	10	5.464481	1.680144	1545|3569|3576|4846|5291|5293|5468|7124|94	CYP1B1|IL6|CXCL8|NOS3|PIK3CB|PIK3CD|	-3.09596	0	64	0	0	-11.2684	7.399559579

								development										75|729230	PPARG|TNF|ROCK2|CCR2							
1	-4.09959	GO:0010632	M1	1	0	GO Biological Processes	19	regulation of epithelial cell migration	-4.09959	5.076148	5.470525	30242	293	183	9	4.918033	1.598524	4318|4846|5291|5293|5468|5743|7040|7124|9475	MMP9|NOS3|PIK3CB|PIK3CD|PPARG|PTGS2|TGFB1|TNF|ROCK2	-2.97806	0	64	0	0	-11.2684	7.399559579
1	-4.03348	GO:0010594	M1	1	0	GO Biological Processes	19	regulation of endothelial cell migration	-4.03348	5.674054	5.588333	30242	233	183	8	4.371585	1.511428	4846|5291|5293|5468|5743|7040|7124|9475	NOS3|PIK3CB|PIK3CD|PPARG|PTGS2|TGFB1|TNF|ROCK2	-2.91745	0	64	0	0	-11.2684	7.399559579
1	-3.97124	GO:0010634	M1	1	0	GO Biological Processes	19	positive regulation of epithelial cell migration	-3.97124	6.535581	5.763092	30242	177	183	7	3.825137	1.417845	4318|4846|5291|5293|5743|7040|9475	MMP9|NOS3|PIK3CB|PIK3CD|PTGS2|TGFB1|ROCK2	-2.86187	0	64	0	0	-11.2684	7.399559579
1	-3.76689	GO:0010595	M1	1	0	GO Biological Processes	19	positive regulation of endothelial cell migration	-3.76689	7.39956	5.792925	30242	134	183	6	3.278689	1.316393	4846|5291|5293|5743|7040|9475	NOS3|PIK3CB|PIK3CD|PTGS2|TGFB1|ROCK2	-2.68385	0	64	0	0	-11.2684	7.399559579
1	-3.12232	GO:0043534	M1	1	0	GO Biological Processes	19	blood vessel endothelial cell migration	-3.12232	5.601926	4.791065	30242	177	183	6	3.278689	1.316393	2050|4846|5468|5743|7040|7124	EPHB4|NOS3|PPARG|PTGS2|TGFB1|TNF	-2.10503	0	64	0	0	-11.2684	7.399559579
1	-2.66218	GO:0016525	M1	1	0	GO Biological Processes	19	negative regulation of angiogenesis	-2.66218	5.545531	4.339917	30242	149	183	5	2.73224	1.205087	5291|5468|7124|9475|729230	PIK3CB|PPARG|TNF|ROCK2|CCR2	-1.69434	0	64	0	0	-11.2684	7.399559579
1	-2.63702	GO:2000181	M1	1	0	GO Biological Processes	19	negative regulation of blood vessel morphogenesis	-2.63702	5.47208	4.298492	30242	151	183	5	2.73224	1.205087	5291|5468|7124|9475|729230	PIK3CB|PPARG|TNF|ROCK2|CCR2	-1.67353	0	64	0	0	-11.2684	7.399559579
1	-2.62459	GO:0043535	M1	1	0	GO Biological Processes	19	regulation of blood vessel endothelial cell migration	-2.62459	5.43608	4.278056	30242	152	183	5	2.73224	1.205087	4846|5468|5743|7040|7124	NOS3|PPARG|PTGS2|TGFB1|TNF	-1.66186	0	64	0	0	-11.2684	7.399559579
1	-2.62459	GO:1901343	M1	1	0	GO Biological Processes	19	negative regulation of vasculature development	-2.62459	5.43608	4.278056	30242	152	183	5	2.73224	1.205087	5291|5468|7124|9475|729230	PIK3CB|PPARG|TNF|ROCK2|CCR2	-1.66186	0	64	0	0	-11.2684	7.399559579
1	-11.1106	GO:0045834	M1	1	0	GO Biological Processes	19	positive regulation of lipid metabolic process	-11.1106	14.41838	12.81141	30242	149	183	13	7.103825	1.898976	134|185|2147|3356|3357|3358|5159|5465|5467|5468|5743|7040|7124	ADORA1|AGTR1|F2|HTR2A|HTR2B|HTR2C|PDGFRB|PPARA|PPARD|PPARG|PTGS2|TGFB1|TNF	-9.34596	0	65	1	1	-11.1106	165.2568306
1	-10.6459	GO:0019216	M1	1	0	GO Biological Processes	19	regulation of lipid metabolic process	-10.6459	8.487511	10.68741	30242	331	183	17	9.289617	2.145862	134|150|185|427|1133|1268|2147|3356|3357|3358|5159|5465|5467|5468|5743|7040|7124	ADORA1|ADRA2A|AGTR1|ASAH1|CHRM5|CNR1|F2|HTR2A|HTR2B|HTR2C|PDGFRB|PPARA|PPARD|PPARG|PTGS2|TGFB1|TNF	-8.91383	0	65	0	0	-11.1106	165.2568306
1	-7.73886	GO:0045444	M1	1	0	GO Biological Processes	19	fat cell differentiation	-7.73886	8.622096	9.053723	30242	230	183	12	6.557377	1.829835	153|154|595|3356|3358|3569|5467|5468|5743|7040|7124|59341	ADRB1|ADRB2|CCND1|HTR2A|HTR2C|IL6|PPARD|PPARG|PTGS2|TGFB1|TNF|TRPV4	-6.22862	0	65	0	0	-11.1106	165.2568306
1	-6.72992	GO:0045598	M1	1	0	GO Biological Processes	19	regulation of fat cell differentiation	-6.72992	10.70008	8.94364	30242	139	183	9	4.918033	1.598524	3356|3358|3569|5467|5468|5743|7040|7124|59341	HTR2A|HTR2C|IL6|PPARD|PPARG|PTGS2|TGFB1|TNF|TRPV4	-5.31926	0	65	0	0	-11.1106	165.2568306
1	-6.66159	GO:0007208	M1	1	0	GO Biological Processes	19	phospholipase C-activating serotonin receptor signaling pathway	-6.66159	165.2568	22.19917	30242	3	183	3	1.639344	0.938686	3356|3357|3358	HTR2A|HTR2B|HTR2C	-5.258	0	65	0	0	-11.1106	165.2568306
1	-6.66159	GO:0010513	M1	1	0	GO Biological	19	positive regulation of	-6.66159	165.2568	22.19917	30242	3	183	3	1.639344	0.938686	3356|3357|3358	HTR2A|HTR2B|HTR2	-5.258	0	65	0	0	-11.1106	165.2568306

						Processes		phosphatidylinositol biosynthetic process											C							
1	-5.66547	GO:0010511	M1	1	0	GO Biological Processes	19	regulation of phosphatidylinositol biosynthetic process	-5.66547	99.1541	17.12618	30242	5	183	3	1.639344	0.938686	3356|3357|3358	HTR2A|HTR2B|HTR2C	-4.35145	0	65	0	0	-11.1106	165.2568306
1	-4.96597	GO:0046889	M1	1	0	GO Biological Processes	19	positive regulation of lipid biosynthetic process	-4.96597	12.09196	7.847585	30242	82	183	6	3.278689	1.316393	3356|3357|3358|5465|5743|7124	HTR2A|HTR2B|HTR2C|PPARA|PTGS2|TNF	-3.73519	0	65	0	0	-11.1106	165.2568306
1	-4.596	GO:0071073	M1	1	0	GO Biological Processes	19	positive regulation of phospholipid biosynthetic process	-4.596	49.57705	11.98765	30242	10	183	3	1.639344	0.938686	3356|3357|3358	HTR2A|HTR2B|HTR2C	-3.4183	0	65	0	0	-11.1106	165.2568306
1	-4.38865	GO:0019934	M1	1	0	GO Biological Processes	19	cGMP-mediated signaling	-4.38865	20.6571	8.680715	30242	32	183	4	2.185792	1.080886	1910|3357|3358|5138	EDNRB|HTR2B|HTR2C|PDE2A	-3.23843	0	65	0	0	-11.1106	165.2568306
1	-4.29322	GO:0045600	M1	1	0	GO Biological Processes	19	positive regulation of fat cell differentiation	-4.29322	12.51946	7.309867	30242	66	183	5	2.73224	1.205087	3356|3358|5467|5468|5743	HTR2A|HTR2C|PPARD|PPARG|PTGS2	-3.15147	0	65	0	0	-11.1106	165.2568306
1	-4.08863	GO:1903725	M1	1	0	GO Biological Processes	19	regulation of phospholipid metabolic process	-4.08863	17.39546	7.890771	30242	38	183	4	2.185792	1.080886	1133|3356|3357|3358	CHRM5|HTR2A|HTR2B|HTR2C	-2.96862	0	65	0	0	-11.1106	165.2568306
1	-4.06476	GO:0046890	M1	1	0	GO Biological Processes	19	regulation of lipid biosynthetic process	-4.06476	6.764899	5.898644	30242	171	183	7	3.825137	1.417845	427|3356|3357|3358|5465|5743|7124	ASAH1|HTR2A|HTR2B|HTR2C|PPARA|PTGS2|TNF	-2.94614	0	65	0	0	-11.1106	165.2568306
1	-4.02686	GO:1903727	M1	1	0	GO Biological Processes	19	positive regulation of phospholipid metabolic process	-4.02686	33.05137	9.68793	30242	15	183	3	1.639344	0.938686	3356|3357|3358	HTR2A|HTR2B|HTR2C	-2.91294	0	65	0	0	-11.1106	165.2568306
1	-3.77899	GO:0071071	M1	1	0	GO Biological Processes	19	regulation of phospholipid biosynthetic process	-3.77899	27.54281	8.789081	30242	18	183	3	1.639344	0.938686	3356|3357|3358	HTR2A|HTR2B|HTR2C	-2.69427	0	65	0	0	-11.1106	165.2568306
1	-2.63793	GO:0019935	M1	1	0	GO Biological Processes	19	cyclic-nucleotide-mediated signaling	-2.63793	7.264037	4.669393	30242	91	183	4	2.185792	1.080886	1910|3357|3358|5138	EDNRB|HTR2B|HTR2C|PDE2A	-1.67418	0	65	0	0	-11.1106	165.2568306
1	-10.8342	GO:0009895	M1	1	0	GO Biological Processes	19	negative regulation of catabolic process	-10.8342	8.72474	10.87345	30242	322	183	17	9.289617	2.145862	134|148|150|596|1268|1956|2903|3156|3357|4843|5290|5465|5582|5970|7124|7157|10135	ADORA1|ADRA1A|ADRA2A|BCL2|CNR1|EGFR|GRIN2A|HMGCR|HTR2B|NOS2|PIK3CA|PPARA|PRKCG|RELA|TNF|TP53|NAMPT	-9.09254	0	66	1	1	-10.8342	11.3970228
1	-7.05729	GO:0010506	M1	1	0	GO Biological Processes	19	regulation of autophagy	-7.05729	6.713559	8.0172	30242	320	183	13	7.103825	1.898976	148|154|472|596|834|836|3357|5290|5291|7157|10135|29110|225689	ADRA1A|ADRB2|ATM|BCL2|CASP1|CASP3|HTR2B|PIK3CA|PIK3CB|TP53|NAMPT|TBK1|MAPK15	-5.61468	0	66	0	0	-10.8342	11.3970228
1	-6.05742	GO:0006914	M1	1	0	GO Biological Processes	19	autophagy	-6.05742	4.694796	6.682874	30242	528	183	15	8.196721	2.027793	148|154|472|596|834|836|1813|1814|3357|5290|5291|7157|10135|29110|225689	ADRA1A|ADRB2|ATM|BCL2|CASP1|CASP3|DRD2|DRD3|HTR2B|PIK3CA|PIK3CB|TP53|NAMPT|TBK1|MAPK15	-4.70467	0	66	0	0	-10.8342	11.3970228
1	-6.05742	GO:0061919	M1	1	0	GO Biological Processes	19	process utilizing autophagic mechanism	-6.05742	4.694796	6.682874	30242	528	183	15	8.196721	2.027793	148|154|472|596|834|836|1813|1814|3357|5290|5291|7157|10135|29110|225689	ADRA1A|ADRB2|ATM|BCL2|CASP1|CASP3|DRD2|DRD3|HTR2B|PIK3CA|PIK3CB|TP5	-4.70467	0	66	0	0	-10.8342	11.3970228

																			3|NAMPT|TBK1|MAPK15							
1	-4.81782	GO:0010507	M1	1	0	GO Biological Processes	19	negative regulation of autophagy	-4.81782	11.39702	7.577489	30242	87	183	6	3.278689	1.316393	148|596|3357|5290|7157|10135	ADRA1A|BCL2|HTR2B|PIK3CA|TP53|NAMPT	-3.60816	0	66	0	0	-10.8342	11.3970228
1	-3.66197	GO:0031330	M1	1	0	GO Biological Processes	19	negative regulation of cellular catabolic process	-3.66197	5.007783	5.103183	30242	264	183	8	4.371585	1.511428	148|596|1268|3357|5290|5582|7157|10135	ADRA1A|BCL2|CNR1|HTR2B|PIK3CA|PRKCG|TP53|NAMPT	-2.59082	0	66	0	0	-10.8342	11.3970228
1	-10.8034	GO:1901654	M1	1	0	GO Biological Processes	19	response to ketone	-10.8034	11.92575	11.91189	30242	194	183	14	7.650273	1.964856	367|595|1080|1728|1956|2155|2908|3383|4129|5734|5970|7040|7298|9475	AR|CCND1|CFTR|NQO1|EGFR|F7|NR3C1|ICAM1|MAOB|PTGER4|RELA|TGFB1|TYMS|ROCK2	-9.06532	0	67	1	1	-10.8034	17.0955342
1	-5.69791	GO:1901655	M1	1	0	GO Biological Processes	19	cellular response to ketone	-5.69791	12.04998	8.460938	30242	96	183	7	3.825137	1.417845	367|1080|1956|2908|3383|5734|9475	AR|CFTR|EGFR|NR3C1|ICAM1|PTGER4|ROCK2	-4.37699	0	67	0	0	-10.8034	17.0955342
1	-4.12226	GO:0044403	M1	1	0	GO Biological Processes	19	biological process involved in symbiotic interaction	-4.12226	5.111036	5.4983	30242	291	183	9	4.918033	1.598524	185|1956|1991|2147|2335|3356|3383|3576|3643	AGTR1|EGFR|ELANE|F2|FN1|HTR2A|ICAM1|CXCL8|INSR	-2.99883	0	67	0	0	-10.8034	17.0955342
1	-3.14917	GO:0071549	M1	1	0	GO Biological Processes	19	cellular response to dexamethasone stimulus	-3.14917	17.09553	6.766184	30242	29	183	3	1.639344	0.938686	1956|2908|3383	EGFR|NR3C1|ICAM1	-2.13101	0	67	0	0	-10.8034	17.0955342
1	-2.80545	GO:0051701	M1	1	0	GO Biological Processes	19	biological process involved in interaction with host	-2.80545	4.860495	4.31677	30242	204	183	6	3.278689	1.316393	185|1956|3356|3383|3576|3643	AGTR1|EGFR|HTR2A|ICAM1|CXCL8|INSR	-1.82086	0	67	0	0	-10.8034	17.0955342
1	-2.77031	GO:0071548	M1	1	0	GO Biological Processes	19	response to dexamethasone	-2.77031	12.71206	5.710538	30242	39	183	3	1.639344	0.938686	1956|2908|3383	EGFR|NR3C1|ICAM1	-1.79128	0	67	0	0	-10.8034	17.0955342
1	-2.62459	GO:0044409	M1	1	0	GO Biological Processes	19	entry into host	-2.62459	5.43608	4.278056	30242	152	183	5	2.73224	1.205087	185|1956|3356|3383|3576	AGTR1|EGFR|HTR2A|ICAM1|CXCL8	-1.66186	0	67	0	0	-10.8034	17.0955342
1	-2.35251	GO:0052126	M1	1	0	GO Biological Processes	19	movement in host environment	-2.35251	4.694796	3.835706	30242	176	183	5	2.73224	1.205087	185|1956|3356|3383|3576	AGTR1|EGFR|HTR2A|ICAM1|CXCL8	-1.41445	0	67	0	0	-10.8034	17.0955342
1	-2.32104	GO:0071385	M1	1	0	GO Biological Processes	19	cellular response to glucocorticoid stimulus	-2.32104	8.853044	4.589503	30242	56	183	3	1.639344	0.938686	1956|2908|3383	EGFR|NR3C1|ICAM1	-1.38595	0	67	0	0	-10.8034	17.0955342
1	-2.21721	GO:0071384	M1	1	0	GO Biological Processes	19	cellular response to corticosteroid stimulus	-2.21721	8.127385	4.347751	30242	61	183	3	1.639344	0.938686	1956|2908|3383	EGFR|NR3C1|ICAM1	-1.29004	0	67	0	0	-10.8034	17.0955342
1	-10.4254	GO:0045744	M1	1	0	GO Biological Processes	19	negative regulation of G protein-coupled receptor signaling pathway	-10.4254	27.54281	15.23221	30242	54	183	9	4.918033	1.598524	100|154|1813|1814|3351|3357|3576|4988|5142	ADA|ADRB2|DRD2|DRD3|HTR1B|HTR2B|CXCL8|OPRM1|PDE4B	-8.71086	0	68	1	1	-10.4254	38.88396014
1	-9.16272	GO:0008277	M1	1	0	GO Biological Processes	19	regulation of G protein-coupled receptor signaling pathway	-9.16272	13.46537	11.32595	30242	135	183	11	6.010929	1.757049	100|154|1813|1814|2147|3351|3357|3576|4988|5142|9475	ADA|ADRB2|DRD2|DRD3|F2|HTR1B|HTR2B|CXCL8|OPRM1|PDE4B|ROCK2	-7.53377	0	68	0	0	-10.4254	38.88396014
1	-8.69216	GO:0031623	M1	1	0	GO Biological Processes	19	receptor internalization	-8.69216	14.49621	11.26459	30242	114	183	10	5.464481	1.680144	1813|1814|1815|3351|3357|3576|3577|3643|6401|10203	DRD2|DRD3|DRD4|HTR1B|HTR2B|CXCL8|CXCR1|INSR|SELE|CALCRL	-7.09277	0	68	0	0	-10.4254	38.88396014
1	-8.34327	GO:0006898	M1	1	0	GO Biological Processes	19	receptor-mediated endocytosis	-8.34327	8.627867	9.430379	30242	249	183	13	7.103825	1.898976	154|351|1813|1814|1815|3351|3357|3576|3577|3643|5291|6401|10203	ADRB2|APP|DRD2|DRD3|DRD4|HTR1B|HTR2B|CXCL8|CXCR1|INSR|PIK3CB|SELE|C	-6.77772	0	68	0	0	-10.4254	38.88396014

																			ALCRL							
1	-8.18352	GO:0043112	M1	1	0	GO Biological Processes	19	receptor metabolic process	-8.18352	10.88518	9.994852	30242	167	183	11	6.010929	1.757049	1813|1814|1815|3351|3357|3576|3577|3643|6401|7040|10203	DRD2|DRD3|DRD4|HTR1B|HTR2B|CXCL8|CXCR1|INSR|SELE|TGFB1|CALCRL	-6.62702	0	68	0	0	-10.4254	38.88396014
1	-6.62602	GO:0002029	M1	1	0	GO Biological Processes	19	desensitization of G protein-coupled receptor signaling pathway	-6.62602	35.9254	13.07379	30242	23	183	5	2.73224	1.205087	154|1813|1814|3351|3357	ADRB2|DRD2|DRD3|HTR1B|HTR2B	-5.22661	0	68	0	0	-10.4254	38.88396014
1	-6.62602	GO:0022401	M1	1	0	GO Biological Processes	19	negative adaptation of signaling pathway	-6.62602	35.9254	13.07379	30242	23	183	5	2.73224	1.205087	154|1813|1814|3351|3357	ADRB2|DRD2|DRD3|HTR1B|HTR2B	-5.22661	0	68	0	0	-10.4254	38.88396014
1	-6.52669	GO:0023058	M1	1	0	GO Biological Processes	19	adaptation of signaling pathway	-6.52669	34.42851	12.7828	30242	24	183	5	2.73224	1.205087	154|1813|1814|3351|3357	ADRB2|DRD2|DRD3|HTR1B|HTR2B	-5.1358	0	68	0	0	-10.4254	38.88396014
1	-5.53708	GO:0002031	M1	1	0	GO Biological Processes	19	G protein-coupled receptor internalization	-5.53708	38.88396	12.19082	30242	17	183	4	2.185792	1.080886	1813|1814|3351|3357	DRD2|DRD3|HTR1B|HTR2B	-4.23375	0	68	0	0	-10.4254	38.88396014
1	-5.44059	GO:0032922	M1	1	0	GO Biological Processes	19	circadian regulation of gene expression	-5.44059	14.58149	8.748259	30242	68	183	6	3.278689	1.316393	1453|1813|1814|3066|5465|10135	CSNK1D|DRD2|DRD3|HDAC2|PPARA|NAMPT	-4.14824	0	68	0	0	-10.4254	38.88396014
1	-5.21212	GO:0046717	M1	1	0	GO Biological Processes	19	acid secretion	-5.21212	19.21591	9.326639	30242	43	183	5	2.73224	1.205087	1133|1813|1814|3351|7442	CHRM5|DRD2|DRD3|HTR1B|TRPV1	-3.95416	0	68	0	0	-10.4254	38.88396014
1	-5.19952	GO:0048259	M1	1	0	GO Biological Processes	19	regulation of receptor-mediated endocytosis	-5.19952	10.14735	7.634822	30242	114	183	7	3.825137	1.417845	351|1813|1814|1815|3643|5291|6401	APP|DRD2|DRD3|DRD4|INSR|PIK3CB|SELE	-3.94355	0	68	0	0	-10.4254	38.88396014
1	-4.6034	GO:0002090	M1	1	0	GO Biological Processes	19	regulation of receptor internalization	-4.6034	14.49621	7.957747	30242	57	183	5	2.73224	1.205087	1813|1814|1815|3643|6401	DRD2|DRD3|DRD4|INSR|SELE	-3.41983	0	68	0	0	-10.4254	38.88396014
1	-3.84368	GO:0006897	M1	1	0	GO Biological Processes	19	endocytosis	-3.84368	3.377891	4.728975	30242	636	183	13	7.103825	1.898976	154|351|1813|1814|1815|3351|3357|3576|3577|3643|5291|6401|10203	ADRB2|APP|DRD2|DRD3|DRD4|HTR1B|HTR2B|CXCL8|CXCR1|INSR|PIK3CB|SELE|CALCRL	-2.74979	0	68	0	0	-10.4254	38.88396014
1	-3.67499	GO:0051781	M1	1	0	GO Biological Processes	19	positive regulation of cell division	-3.67499	9.284092	6.106762	30242	89	183	5	2.73224	1.205087	1813|1814|2263|3357|7040	DRD2|DRD3|FGFR2|HTR2B|TGFB1	-2.60351	0	68	0	0	-10.4254	38.88396014
1	-3.46616	GO:0030100	M1	1	0	GO Biological Processes	19	regulation of endocytosis	-3.46616	5.405597	5.046437	30242	214	183	7	3.825137	1.417845	351|1813|1814|1815|3643|5291|6401	APP|DRD2|DRD3|DRD4|INSR|PIK3CB|SELE	-2.41476	0	68	0	0	-10.4254	38.88396014
1	-3.45874	GO:0051898	M1	1	0	GO Biological Processes	19	negative regulation of protein kinase B signaling	-3.45874	12.01868	6.381713	30242	55	183	4	2.185792	1.080886	1813|1814|5291|5465	DRD2|DRD3|PIK3CB|PPARA	-2.40826	0	68	0	0	-10.4254	38.88396014
1	-3.24195	GO:0002092	M1	1	0	GO Biological Processes	19	positive regulation of receptor internalization	-3.24195	18.36187	7.04212	30242	27	183	3	1.639344	0.938686	1813|3643|6401	DRD2|INSR|SELE	-2.21358	0	68	0	0	-10.4254	38.88396014
1	-2.38839	GO:0048260	M1	1	0	GO Biological Processes	19	positive regulation of receptor-mediated endocytosis	-2.38839	9.35416	4.749553	30242	53	183	3	1.639344	0.938686	1813|3643|6401	DRD2|INSR|SELE	-1.44695	0	68	0	0	-10.4254	38.88396014
1	-2.34216	GO:0051302	M1	1	0	GO Biological Processes	19	regulation of cell division	-2.34216	4.668272	3.819037	30242	177	183	5	2.73224	1.205087	1813|1814|2263|3357|7040	DRD2|DRD3|FGFR2|HTR2B|TGFB1	-1.40555	0	68	0	0	-10.4254	38.88396014
1	-10.3948	GO:0022600	M1	1	0	GO Biological Processes	19	digestive system process	-10.3948	17.47909	13.13497	30242	104	183	11	6.010929	1.757049	150|1128|1131|1133|1645|1814|4986|6523|7099|7421|7442	ADRA2A|CHRM1|CHRM3|CHRM5|AKR1C1|DRD3|OPRK1|SLC5A1|TLR4|VDR|TRPV1	-8.68413	0	69	1	1	-10.3948	17.47908785
1	-9.09428	GO:0007586	M1	1	0	GO Biological Processes	19	digestion	-9.09428	13.2688	11.22999	30242	137	183	11	6.010929	1.757049	150|1128|1131|1133|1645|1814|4986|6523|709	ADRA2A|CHRM1|CHRM3|CHRM5|AKR1C	-7.47001	0	69	0	0	-10.3948	17.47908785

																		9|7421|7442	1|DRD3|OPRK1|SLC5A1|TLR4|VDR|TRPV1							
1	-3.95744	GO:0050892	M1	1	0	GO Biological Processes	19	intestinal absorption	-3.95744	16.12262	7.560397	30242	41	183	4	2.185792	1.080886	150|1645|6523|7421	ADRA2A|AKR1C1|SLC5A1|VDR	-2.8503	0	69	0	0	-10.3948	17.47908785
1	-10.3916	GO:0001101	M1	1	0	GO Biological Processes	19	response to acid chemical	-10.3916	14.6895	12.43818	30242	135	183	12	6.557377	1.829835	836|1723|1728|2155|2902|3383|4313|4914|4915|5290|5970|7124	CASP3|DHODH|NQO1|F7|GRIN1|ICAM1|MMP2|NTRK1|NTRK2|PIK3CA|RELA|TNF	-8.68413	0	70	1	1	-10.3916	55.0856102
1	-8.61802	GO:0043200	M1	1	0	GO Biological Processes	19	response to amino acid	-8.61802	14.24628	11.15292	30242	116	183	10	5.464481	1.680144	836|1723|1728|2155|2902|3383|4313|4915|5970|7124	CASP3|DHODH|NQO1|F7|GRIN1|ICAM1|MMP2|NTRK2|RELA|TNF	-7.02518	0	70	0	0	-10.3916	55.0856102
1	-6.77076	GO:0050435	M1	1	0	GO Biological Processes	19	amyloid-beta metabolic process	-6.77076	17.26564	10.39971	30242	67	183	7	3.825137	1.417845	836|1636|4915|5970|7124|9475|23621	CASP3|ACE|NTRK2|RELA|TNF|ROCK2|BACE1	-5.35581	0	70	0	0	-10.3916	55.0856102
1	-5.89613	GO:0034205	M1	1	0	GO Biological Processes	19	amyloid-beta formation	-5.89613	17.39546	9.667222	30242	57	183	6	3.278689	1.316393	836|4915|5970|7124|9475|23621	CASP3|NTRK2|RELA|TNF|ROCK2|BACE1	-4.56049	0	70	0	0	-10.3916	55.0856102
1	-5.44059	GO:0042987	M1	1	0	GO Biological Processes	19	amyloid precursor protein catabolic process	-5.44059	14.58149	8.748259	30242	68	183	6	3.278689	1.316393	836|4915|5970|7124|9475|23621	CASP3|NTRK2|RELA|TNF|ROCK2|BACE1	-4.14824	0	70	0	0	-10.3916	55.0856102
1	-5.23453	GO:1902004	M1	1	0	GO Biological Processes	19	positive regulation of amyloid-beta formation	-5.23453	33.05137	11.18758	30242	20	183	4	2.185792	1.080886	836|5970|7124|9475	CASP3|RELA|TNF|ROCK2	-3.97355	0	70	0	0	-10.3916	55.0856102
1	-4.97262	GO:1902003	M1	1	0	GO Biological Processes	19	regulation of amyloid-beta formation	-4.97262	17.21425	8.771899	30242	48	183	5	2.73224	1.205087	836|4915|5970|7124|9475	CASP3|NTRK2|RELA|TNF|ROCK2	-3.74133	0	70	0	0	-10.3916	55.0856102
1	-4.82801	GO:1902993	M1	1	0	GO Biological Processes	19	positive regulation of amyloid precursor protein catabolic process	-4.82801	26.44109	9.92925	30242	25	183	4	2.185792	1.080886	836|5970|7124|9475	CASP3|RELA|TNF|ROCK2	-3.61611	0	70	0	0	-10.3916	55.0856102
1	-4.74896	GO:0071316	M1	1	0	GO Biological Processes	19	cellular response to nicotine	-4.74896	55.08561	12.6619	30242	9	183	3	1.639344	0.938686	4914|5970|7124	NTRK1|RELA|TNF	-3.54861	0	70	0	0	-10.3916	55.0856102
1	-4.67964	GO:1902991	M1	1	0	GO Biological Processes	19	regulation of amyloid precursor protein catabolic process	-4.67964	15.02335	8.121934	30242	55	183	5	2.73224	1.205087	836|4915|5970|7124|9475	CASP3|NTRK2|RELA|TNF|ROCK2	-3.48755	0	70	0	0	-10.3916	55.0856102
1	-4.59938	GO:0042982	M1	1	0	GO Biological Processes	19	amyloid precursor protein metabolic process	-4.59938	10.43727	7.188241	30242	95	183	6	3.278689	1.316393	836|4915|5970|7124|9475|23621	CASP3|NTRK2|RELA|TNF|ROCK2|BACE1	-3.4183	0	70	0	0	-10.3916	55.0856102
1	-3.98134	GO:0034248	M1	1	0	GO Biological Processes	19	regulation of cellular amide metabolic process	-3.98134	3.727598	4.952462	30242	532	183	12	6.557377	1.829835	351|836|2915|3569|4915|5591|5970|6197|7124|7157|7298|9475	APP|CASP3|GRM5|IL6|NTRK2|PRKDC|RELA|RPS6KA3|TNF|TP53|TYMS|ROCK2	-2.87065	0	70	0	0	-10.3916	55.0856102
1	-3.32425	GO:0034250	M1	1	0	GO Biological Processes	19	positive regulation of cellular amide metabolic process	-3.32425	6.120623	5.098915	30242	162	183	6	3.278689	1.316393	836|3569|5591|5970|7124|9475	CASP3|IL6|PRKDC|RELA|TNF|ROCK2	-2.28723	0	70	0	0	-10.3916	55.0856102
1	-2.09634	GO:0006417	M1	1	0	GO Biological Processes	19	regulation of translation	-2.09634	2.81888	3.09753	30242	469	183	8	4.371585	1.511428	351|2915|3569|5591|6197|7124|7298|9475	APP|GRM5|IL6|PRKDC|RPS6KA3|TNF|TYMS|ROCK2	-1.17705	0	70	0	0	-10.3916	55.0856102
1	-10.321	GO:0034612	M1	1	0	GO Biological Processes	19	response to tumor necrosis factor	-10.321	9.797836	10.96457	30242	253	183	15	8.196721	2.027793	427|834|836|3383|3551|3576|4792|5743|5970|6347|6401|7124|7132|7157|7442	ASAH1|CASP1|CASP3|ICAM1|IKBKB|CXCL8|NFKBIA|PTGS2|RELA|CCL2|SELE|TNF|TNFRSF1A|TP53|TRP	-8.61637	0	71	1	1	-10.321	11.68482641

																			V1							
1	-7.75986	GO:0071356	M1	1	0	GO Biological Processes	19	cellular response to tumor necrosis factor	-7.75986	8.659747	9.078494	30242	229	183	12	6.557377	1.829835	427|834|3383|3551|3576|4792|5970|6347|7124|7132|7157|7442	ASAH1|CASP1|ICAM1|IKBKB|CXCL8|NFKBIA|RELA|CCL2|TNF|TNFRSF1A|TP53|TRPV1	-6.24693	0	71	0	0	-10.321	11.68482641
1	-7.46635	GO:0019221	M1	1	0	GO Biological Processes	19	cytokine-mediated signaling pathway	-7.46635	5.601926	7.862445	30242	472	183	16	8.743169	2.088053	834|1230|3551|3569|3576|3577|4792|5468|5970|6347|7124|7132|7157|8986|29110|729230	CASP1|CCR1|IKBKB|IL6|CXCL8|CXCR1|NFKBIA|PPARG|RELA|CCL2|TNF|TNFRSF1A|TP53|RPS6KA4|TBK1|CCR2	-5.98302	0	71	0	0	-10.321	11.68482641
1	-6.78764	GO:0007249	M1	1	0	GO Biological Processes	19	I-kappaB kinase/NF-kappaB signaling	-6.78764	7.057231	7.959495	30242	281	183	12	6.557377	1.829835	834|2099|2149|3357|3551|4792|5970|7099|7124|7132|9475|29110	CASP1|ESR1|F2R|HTR2B|IKBKB|NFKBIA|RELA|TLR4|TNF|TNFRSF1A|ROCK2|TBK1	-5.37126	0	71	0	0	-10.321	11.68482641
1	-5.66753	GO:0043123	M1	1	0	GO Biological Processes	19	positive regulation of I-kappaB kinase/NF-kappaB signaling	-5.66753	7.996298	7.467849	30242	186	183	9	4.918033	1.598524	834|2149|3357|3551|5970|7099|7124|7132|29110	CASP1|F2R|HTR2B|IKBKB|RELA|TLR4|TNF|TNFRSF1A|TBK1	-4.35145	0	71	0	0	-10.321	11.68482641
1	-5.60797	GO:0033209	M1	1	0	GO Biological Processes	19	tumor necrosis factor-mediated signaling pathway	-5.60797	11.68483	8.308606	30242	99	183	7	3.825137	1.417845	834|3551|4792|5970|7124|7132|7157	CASP1|IKBKB|NFKBIA|RELA|TNF|TNFRSF1A|TP53	-4.29904	0	71	0	0	-10.321	11.68482641
1	-5.50723	GO:0043122	M1	1	0	GO Biological Processes	19	regulation of I-kappaB kinase/NF-kappaB signaling	-5.50723	6.636821	6.968837	30242	249	183	10	5.464481	1.680144	834|2099|2149|3357|3551|5970|7099|7124|7132|29110	CASP1|ESR1|F2R|HTR2B|IKBKB|RELA|TLR4|TNF|TNFRSF1A|TBK1	-4.20667	0	71	0	0	-10.321	11.68482641
1	-3.35667	GO:0009615	M1	1	0	GO Biological Processes	19	response to virus	-3.35667	4.030654	4.570319	30242	369	183	9	4.918033	1.598524	596|1129|1543|3551|3569|4986|5970|7124|29110	BCL2|CHRM2|CYP1A1|IKBKB|IL6|OPRK1|RELA|TNF|TBK1	-2.31602	0	71	0	0	-10.321	11.68482641
1	-10.1821	GO:0030855	M1	1	0	GO Biological Processes	19	epithelial cell differentiation	-10.1821	5.654199	9.307827	30242	643	183	22	12.02186	2.404073	367|427|595|836|1543|1645|2099|2263|3066|3383|3551|4221|4318|4914|5138|5241|5468|7124|7132|7298|7421|9475	AR|ASAH1|CCND1|CASP3|CYP1A1|AKR1C1|ESR1|FGFR2|HDAC2|ICAM1|IKBKB|MEN1|MMP9|NTRK1|PDE2A|PGR|PPARG|TNF|TNFRSF1A|TYMS|VDR|ROCK2	-8.48025	0	72	1	1	-10.1821	5.654199492
1	-2.10432	GO:0009913	M1	1	0	GO Biological Processes	19	epidermal cell differentiation	-2.10432	4.090516	3.43869	30242	202	183	5	2.73224	1.205087	427|836|3066|7421|9475	ASAH1|CASP3|HDAC2|VDR|ROCK2	-1.18457	0	72	0	0	-10.1821	5.654199492
1	-10.1641	GO:0071466	M1	1	0	GO Biological Processes	19	cellular response to xenobiotic stimulus	-10.1641	12.13751	11.59526	30242	177	183	13	7.103825	1.898976	1543|1544|1545|1557|1645|1728|1956|2902|3351|3757|4842|4843|5142	CYP1A1|CYP1A2|CYP1B1|CYP2C19|AKR1C1|NQO1|EGFR|GRIN1|HTR1B|KCNH2|NOS1|NOS2|PDE4B	-8.46367	0	73	1	1	-10.1641	66.10273224
1	-6.57703	GO:0097267	M1	1	0	GO Biological Processes	19	omega-hydroxylase P450 pathway	-6.57703	66.10273	16.06579	30242	10	183	4	2.185792	1.080886	1543|1544|1545|1557	CYP1A1|CYP1A2|CYP1B1|CYP2C19	-5.18107	0	73	0	0	-10.1641	66.10273224
1	-6.3976	GO:0006805	M1	1	0	GO Biological Processes	19	xenobiotic metabolic process	-6.3976	11.9104	8.985284	30242	111	183	8	4.371585	1.511428	1543|1544|1545|1557|1645|1728|2902|4842	CYP1A1|CYP1A2|CYP1B1|CYP2C19|AKR1C1|NQO1|GRIN1|NOS1	-5.01719	0	73	0	0	-10.1641	66.10273224
1	-6.25073	GO:0006720	M1	1	0	GO Biological	19	isoprenoid metabolic	-6.25073	11.39702	8.753941	30242	116	183	8	4.371585	1.511428	1543|1544|1545|1557|1	CYP1A1|CYP1A2|CY	-4.87888	0	73	0	0	-10.1641	66.10273224

						Processes		process										645|1956|3156|5467	P1B1|CYP2C19|AKR1C1|EGFR|HMGCR|PPARD							
1	-5.66759	GO:0006721	M1	1	0	GO Biological Processes	19	terpenoid metabolic process	-5.66759	11.92575	8.409417	30242	97	183	7	3.825137	1.417845	1543|1544|1545|1557|1645|1956|5467	CYP1A1|CYP1A2|CYP1B1|CYP2C19|AKR1C1|EGFR|PPARD	-4.35145	0	73	0	0	-10.1641	66.10273224
1	-5.48428	GO:0008210	M1	1	0	GO Biological Processes	19	estrogen metabolic process	-5.48428	21.74432	9.983783	30242	38	183	5	2.73224	1.205087	1543|1544|1545|1588|7364	CYP1A1|CYP1A2|CYP1B1|CYP19A1|UGT2B7	-4.18647	0	73	0	0	-10.1641	66.10273224
1	-5.32939	GO:0019373	M1	1	0	GO Biological Processes	19	epoxygenase P450 pathway	-5.32939	34.79091	11.49593	30242	19	183	4	2.185792	1.080886	1543|1544|1545|1557	CYP1A1|CYP1A2|CYP1B1|CYP2C19	-4.05665	0	73	0	0	-10.1641	66.10273224
1	-4.81782	GO:0016101	M1	1	0	GO Biological Processes	19	diterpenoid metabolic process	-4.81782	11.39702	7.577489	30242	87	183	6	3.278689	1.316393	1543|1544|1545|1645|1956|5467	CYP1A1|CYP1A2|CYP1B1|AKR1C1|EGFR|PPARD	-3.60816	0	73	0	0	-10.1641	66.10273224
1	-4.67755	GO:0034754	M1	1	0	GO Biological Processes	19	cellular hormone metabolic process	-4.67755	8.44378	6.813509	30242	137	183	7	3.825137	1.417845	1543|1544|1545|1588|1645|2099|7364	CYP1A1|CYP1A2|CYP1B1|CYP19A1|AKR1C1|ESR1|UGT2B7	-3.48589	0	73	0	0	-10.1641	66.10273224
1	-4.56637	GO:0042743	M1	1	0	GO Biological Processes	19	hydrogen peroxide metabolic process	-4.56637	14.24628	7.878723	30242	58	183	5	2.73224	1.205087	1543|1544|1956|4129|4314	CYP1A1|CYP1A2|EGFR|MAOB|MMP3	-3.3924	0	73	0	0	-10.1641	66.10273224
1	-4.45935	GO:0019748	M1	1	0	GO Biological Processes	19	secondary metabolic process	-4.45935	13.54564	7.652923	30242	61	183	5	2.73224	1.205087	596|1543|1544|1545|1645	BCL2|CYP1A1|CYP1A2|CYP1B1|AKR1C1	-3.30278	0	73	0	0	-10.1641	66.10273224
1	-4.23506	GO:0042445	M1	1	0	GO Biological Processes	19	hormone metabolic process	-4.23506	6.064471	5.855506	30242	218	183	8	4.371585	1.511428	1543|1544|1545|1588|1636|1645|2099|7364	CYP1A1|CYP1A2|CYP1B1|CYP19A1|ACE|AKR1C1|ESR1|UGT2B7	-3.0975	0	73	0	0	-10.1641	66.10273224
1	-4.13489	GO:0042178	M1	1	0	GO Biological Processes	19	xenobiotic catabolic process	-4.13489	17.8656	8.009394	30242	37	183	4	2.185792	1.080886	1543|1544|1557|4842	CYP1A1|CYP1A2|CYP2C19|NOS1	-3.00894	0	73	0	0	-10.1641	66.10273224
1	-3.86758	GO:0001523	M1	1	0	GO Biological Processes	19	retinoid metabolic process	-3.86758	10.20104	6.46983	30242	81	183	5	2.73224	1.205087	1543|1544|1545|1645|5467	CYP1A1|CYP1A2|CYP1B1|AKR1C1|PPARD	-2.77024	0	73	0	0	-10.1641	66.10273224
1	-3.77899	GO:0009404	M1	1	0	GO Biological Processes	19	toxin metabolic process	-3.77899	27.54281	8.789081	30242	18	183	3	1.639344	0.938686	1543|1544|1545	CYP1A1|CYP1A2|CYP1B1	-2.69427	0	73	0	0	-10.1641	66.10273224
1	-3.77899	GO:0050665	M1	1	0	GO Biological Processes	19	hydrogen peroxide biosynthetic process	-3.77899	27.54281	8.789081	30242	18	183	3	1.639344	0.938686	1543|1544|4129	CYP1A1|CYP1A2|MAOB	-2.69427	0	73	0	0	-10.1641	66.10273224
1	-3.65322	GO:0006778	M1	1	0	GO Biological Processes	19	porphyrin-containing compound metabolic process	-3.65322	13.49035	6.82741	30242	49	183	4	2.185792	1.080886	1543|1544|4363|5498	CYP1A1|CYP1A2|ABCC1|PPOX	-2.58304	0	73	0	0	-10.1641	66.10273224
1	-3.39998	GO:0034308	M1	1	0	GO Biological Processes	19	primary alcohol metabolic process	-3.39998	8.100825	5.604974	30242	102	183	5	2.73224	1.205087	217|1543|1544|1545|1645	ALDH2|CYP1A1|CYP1A2|CYP1B1|AKR1C1	-2.35475	0	73	0	0	-10.1641	66.10273224
1	-3.3416	GO:0033013	M1	1	0	GO Biological Processes	19	tetrapyrrole metabolic process	-3.3416	11.20385	6.121324	30242	59	183	4	2.185792	1.080886	1543|1544|4363|5498	CYP1A1|CYP1A2|ABCC1|PPOX	-2.30368	0	73	0	0	-10.1641	66.10273224
1	-2.46008	GO:0042572	M1	1	0	GO Biological Processes	19	retinol metabolic process	-2.46008	9.91541	4.922851	30242	50	183	3	1.639344	0.938686	1543|1544|1545	CYP1A1|CYP1A2|CYP1B1	-1.51158	0	73	0	0	-10.1641	66.10273224
1	-2.00253	GO:0070988	M1	1	0	GO Biological Processes	19	demethylation	-2.00253	6.791377	3.865442	30242	73	183	3	1.639344	0.938686	328|1543|1544	APEX1|CYP1A1|CYP1A2	-1.09008	0	73	0	0	-10.1641	66.10273224
1	-10.0127	GO:0097696	M1	1	0	GO Biological Processes	19	receptor signaling pathway via STAT	-10.0127	11.80406	11.40682	30242	182	183	13	7.103825	1.898976	1545|2147|2149|3066|3558|3569|5468|5979|6347|7040|7124|7132|729230	CYP1B1|F2|F2R|HDAC2|IL2|IL6|PPARG|RET|CCL2|TGFB1|TNF|TNFRSF1A|CCR2	-8.32321	0	74	1	1	-10.0127	55.0856102
1	-8.50682	GO:0060965	M1	1	0	GO Biological Processes	19	negative regulation of gene silencing by	-8.50682	45.07004	16.13407	30242	22	183	6	3.278689	1.316393	2099|3569|5468|7040|7124|7157	ESR1|IL6|PPARG|TGFB1|TNF|TP53	-6.92574	0	74	0	0	-10.0127	55.0856102

								miRNA																		
1	-8.25497	GO:0060149	M1	1	0	GO Biological Processes	19	negative regulation of posttranscriptional gene silencing	-8.25497	41.31421	15.41584	30242	24	183	6	3.278689	1.316393	2099|3569|5468|7040|7124|7157	ESR1|IL6|PPARG|TGFB1|TNF|TP53	-6.69346	0	74	0	0	-10.0127	55.0856102
1	-8.25497	GO:0060967	M1	1	0	GO Biological Processes	19	negative regulation of gene silencing by RNA	-8.25497	41.31421	15.41584	30242	24	183	6	3.278689	1.316393	2099|3569|5468|7040|7124|7157	ESR1|IL6|PPARG|TGFB1|TNF|TP53	-6.69346	0	74	0	0	-10.0127	55.0856102
1	-8.13796	GO:1903798	M1	1	0	GO Biological Processes	19	regulation of production of miRNAs involved in gene silencing by miRNA	-8.13796	39.66164	15.08902	30242	25	183	6	3.278689	1.316393	1956|2099|3569|7040|7124|7157	EGFR|ESR1|IL6|TGFB1|TNF|TP53	-6.59131	0	74	0	0	-10.0127	55.0856102
1	-8.07602	GO:0007259	M1	1	0	GO Biological Processes	19	receptor signaling pathway via JAK-STAT	-8.07602	10.63056	9.853984	30242	171	183	11	6.010929	1.757049	1545|2147|2149|3066|3558|3569|5979|6347|7124|7132|729230	CYP1B1|F2|F2R|HDAC2|IL2|IL6|RET|CCL2|TNF|TNFRSF1A|CCR2	-6.53399	0	74	0	0	-10.0127	55.0856102
1	-8.0262	GO:0070920	M1	1	0	GO Biological Processes	19	regulation of production of small RNA involved in gene silencing by RNA	-8.0262	38.13619	14.78094	30242	26	183	6	3.278689	1.316393	1956|2099|3569|7040|7124|7157	EGFR|ESR1|IL6|TGFB1|TNF|TP53	-6.48727	0	74	0	0	-10.0127	55.0856102
1	-7.7363	GO:0060964	M1	1	0	GO Biological Processes	19	regulation of gene silencing by miRNA	-7.7363	23.60812	12.35793	30242	49	183	7	3.825137	1.417845	1956|2099|3569|5468|7040|7124|7157	EGFR|ESR1|IL6|PPARG|TGFB1|TNF|TP53	-6.22784	0	74	0	0	-10.0127	55.0856102
1	-7.71819	GO:0060969	M1	1	0	GO Biological Processes	19	negative regulation of gene silencing	-7.71819	34.19107	13.95275	30242	29	183	6	3.278689	1.316393	2099|3569|5468|7040|7124|7157	ESR1|IL6|PPARG|TGFB1|TNF|TP53	-6.2108	0	74	0	0	-10.0127	55.0856102
1	-7.65837	GO:1903799	M1	1	0	GO Biological Processes	19	negative regulation of production of miRNAs involved in gene silencing by miRNA	-7.65837	55.08561	16.34806	30242	15	183	5	2.73224	1.205087	2099|3569|7040|7124|7157	ESR1|IL6|TGFB1|TNF|TP53	-6.15537	0	74	0	0	-10.0127	55.0856102
1	-7.55053	GO:0060147	M1	1	0	GO Biological Processes	19	regulation of posttranscriptional gene silencing	-7.55053	22.24611	11.96426	30242	52	183	7	3.825137	1.417845	1956|2099|3569|5468|7040|7124|7157	EGFR|ESR1|IL6|PPARG|TGFB1|TNF|TP53	-6.05955	0	74	0	0	-10.0127	55.0856102
1	-7.49122	GO:0060966	M1	1	0	GO Biological Processes	19	regulation of gene silencing by RNA	-7.49122	21.82637	11.84032	30242	53	183	7	3.825137	1.417845	1956|2099|3569|5468|7040|7124|7157	EGFR|ESR1|IL6|PPARG|TGFB1|TNF|TP53	-6.00535	0	74	0	0	-10.0127	55.0856102
1	-6.64034	GO:0014002	M1	1	0	GO Biological Processes	19	astrocyte development	-6.64034	23.05909	11.29437	30242	43	183	6	3.278689	1.316393	135|351|1956|3569|7099|7124	ADORA2A|APP|EGFR|IL6|TLR4|TNF	-5.23954	0	74	0	0	-10.0127	55.0856102
1	-6.43191	GO:0048143	M1	1	0	GO Biological Processes	19	astrocyte activation	-6.43191	33.05137	12.50913	30242	25	183	5	2.73224	1.205087	135|351|1956|3569|7124	ADORA2A|APP|EGFR|IL6|TNF	-5.04616	0	74	0	0	-10.0127	55.0856102
1	-6.30862	GO:1904892	M1	1	0	GO Biological Processes	19	regulation of receptor signaling pathway via STAT	-6.30862	11.59697	8.844746	30242	114	183	8	4.371585	1.511428	1545|2147|2149|3569|5468|5979|7040|7124	CYP1B1|F2|F2R|IL6|PPARG|RET|TGFB1|TNF	-4.93284	0	74	0	0	-10.0127	55.0856102
1	-6.29286	GO:1904894	M1	1	0	GO Biological Processes	19	positive regulation of receptor signaling pathway via STAT	-6.29286	20.23553	10.51442	30242	49	183	6	3.278689	1.316393	1545|2147|2149|3569|7040|7124	CYP1B1|F2|F2R|IL6|TGFB1|TNF	-4.91774	0	74	0	0	-10.0127	55.0856102
1	-6.20876	GO:1903800	M1	1	0	GO Biological Processes	19	positive regulation of production of miRNAs involved in gene silencing by miRNA	-6.20876	55.08561	14.62142	30242	12	183	4	2.185792	1.080886	1956|3569|7040|7157	EGFR|IL6|TGFB1|TP53	-4.84272	0	74	0	0	-10.0127	55.0856102
1	-6.19981	GO:0048708	M1	1	0	GO Biological Processes	19	astrocyte differentiation	-6.19981	14.28145	9.339027	30242	81	183	7	3.825137	1.417845	135|351|1956|2147|3569|7099|7124	ADORA2A|APP|EGFR|F2|IL6|TLR4|TNF	-4.83441	0	74	0	0	-10.0127	55.0856102
1	-6.16326	GO:0060968	M1	1	0	GO Biological Processes	19	regulation of gene silencing	-6.16326	14.10729	9.273433	30242	82	183	7	3.825137	1.417845	1956|2099|3569|5468|7040|7124|7157	EGFR|ESR1|IL6|PPARG|TGFB1|TNF|TP53	-4.80234	0	74	0	0	-10.0127	55.0856102
1	-6.13627	GO:0035196	M1	1	0	GO Biological Processes	19	production of miRNAs involved in gene	-6.13627	19.0681	10.17464	30242	52	183	6	3.278689	1.316393	1956|2099|3569|7040|7124|7157	EGFR|ESR1|IL6|TGFB1|TNF|TP53	-4.77661	0	74	0	0	-10.0127	55.0856102

								silencing by miRNA																		
1	-6.03729	GO:0031050	M1	1	0	GO Biological Processes	19	dsRNA processing	-6.03729	18.36187	9.963515	30242	54	183	6	3.278689	1.316393	1956|2099|3569|7040|7124|7157	EGFR|ESR1|IL6|TGFB1|TNF|TP53	-4.68703	0	74	0	0	-10.0127	55.0856102
1	-6.03729	GO:0070918	M1	1	0	GO Biological Processes	19	production of small RNA involved in gene silencing by RNA	-6.03729	18.36187	9.963515	30242	54	183	6	3.278689	1.316393	1956|2099|3569|7040|7124|7157	EGFR|ESR1|IL6|TGFB1|TNF|TP53	-4.68703	0	74	0	0	-10.0127	55.0856102
1	-5.98763	GO:0034103	M1	1	0	GO Biological Processes	19	regulation of tissue remodeling	-5.98763	13.29653	8.961873	30242	87	183	7	3.825137	1.417845	760|1956|3558|3569|7040|7157|9475	CA2|EGFR|IL2|IL6|TGFB1|TP53|ROCK2	-4.64106	0	74	0	0	-10.0127	55.0856102
1	-5.67808	GO:0010573	M1	1	0	GO Biological Processes	19	vascular endothelial growth factor production	-5.67808	15.9926	9.220393	30242	62	183	6	3.278689	1.316393	1545|3569|5743|7040|7124|729230	CYP1B1|IL6|PTGS2|TGFB1|TNF|CCR2	-4.35832	0	74	0	0	-10.0127	55.0856102
1	-5.16186	GO:0046427	M1	1	0	GO Biological Processes	19	positive regulation of receptor signaling pathway via JAK-STAT	-5.16186	18.77919	9.208427	30242	44	183	5	2.73224	1.205087	1545|2147|2149|3569|7124	CYP1B1|F2|F2R|IL6|TNF	-3.90937	0	74	0	0	-10.0127	55.0856102
1	-5.14962	GO:0021782	M1	1	0	GO Biological Processes	19	glial cell development	-5.14962	9.972395	7.554453	30242	116	183	7	3.825137	1.417845	135|351|1956|3569|4915|7099|7124	ADORA2A|APP|EGFR|IL6|NTRK2|TLR4|TNF	-3.8996	0	74	0	0	-10.0127	55.0856102
1	-4.73339	GO:0046849	M1	1	0	GO Biological Processes	19	bone remodeling	-4.73339	11.01712	7.425787	30242	90	183	6	3.278689	1.316393	154|760|1956|3351|3569|7040	ADRB2|CA2|EGFR|HTR1B|IL6|TGFB1	-3.53566	0	74	0	0	-10.0127	55.0856102
1	-4.62504	GO:0010575	M1	1	0	GO Biological Processes	19	positive regulation of vascular endothelial growth factor production	-4.62504	23.60812	9.338469	30242	28	183	4	2.185792	1.080886	1545|3569|5743|7040	CYP1B1|IL6|PTGS2|TGFB1	-3.43978	0	74	0	0	-10.0127	55.0856102
1	-4.56637	GO:0010574	M1	1	0	GO Biological Processes	19	regulation of vascular endothelial growth factor production	-4.56637	14.24628	7.878723	30242	58	183	5	2.73224	1.205087	1545|3569|5743|7040|729230	CYP1B1|IL6|PTGS2|TGFB1|CCR2	-3.3924	0	74	0	0	-10.0127	55.0856102
1	-4.50254	GO:2000637	M1	1	0	GO Biological Processes	19	positive regulation of gene silencing by miRNA	-4.50254	22.03424	8.993612	30242	30	183	4	2.185792	1.080886	1956|3569|7040|7157	EGFR|IL6|TGFB1|TP53	-3.33837	0	74	0	0	-10.0127	55.0856102
1	-4.44459	GO:0060148	M1	1	0	GO Biological Processes	19	positive regulation of posttranscriptional gene silencing	-4.44459	21.32346	8.83349	30242	31	183	4	2.185792	1.080886	1956|3569|7040|7157	EGFR|IL6|TGFB1|TP53	-3.29079	0	74	0	0	-10.0127	55.0856102
1	-4.40051	GO:0046425	M1	1	0	GO Biological Processes	19	regulation of receptor signaling pathway via JAK-STAT	-4.40051	9.626611	6.842752	30242	103	183	6	3.278689	1.316393	1545|2147|2149|3569|5979|7124	CYP1B1|F2|F2R|IL6|RET|TNF	-3.24867	0	74	0	0	-10.0127	55.0856102
1	-4.20006	GO:0042531	M1	1	0	GO Biological Processes	19	positive regulation of tyrosine phosphorylation of STAT protein	-4.20006	11.97513	7.121335	30242	69	183	5	2.73224	1.205087	3066|3558|3569|7124|7132	HDAC2|IL2|IL6|TNF|TNFRSF1A	-3.06779	0	74	0	0	-10.0127	55.0856102
1	-4.18252	GO:0060251	M1	1	0	GO Biological Processes	19	regulation of glial cell proliferation	-4.18252	18.36187	8.132751	30242	36	183	4	2.185792	1.080886	1128|1956|3569|7124	CHRM1|EGFR|IL6|TNF	-3.05249	0	74	0	0	-10.0127	55.0856102
1	-3.92585	GO:0010721	M1	1	0	GO Biological Processes	19	negative regulation of cell development	-3.92585	6.426655	5.697651	30242	180	183	7	3.825137	1.417845	1814|1910|2147|3569|7124|7157|7442	DRD3|EDNRB|F2|IL6|TNF|TP53|TRPV1	-2.82364	0	74	0	0	-10.0127	55.0856102
1	-3.74486	GO:0042509	M1	1	0	GO Biological Processes	19	regulation of tyrosine phosphorylation of STAT protein	-3.74486	9.607955	6.23733	30242	86	183	5	2.73224	1.205087	3066|3558|3569|7124|7132	HDAC2|IL2|IL6|TNF|TNFRSF1A	-2.66416	0	74	0	0	-10.0127	55.0856102
1	-3.67499	GO:0007260	M1	1	0	GO Biological Processes	19	tyrosine phosphorylation of STAT protein	-3.67499	9.284092	6.106762	30242	89	183	5	2.73224	1.205087	3066|3558|3569|7124|7132	HDAC2|IL2|IL6|TNF|TNFRSF1A	-2.60351	0	74	0	0	-10.0127	55.0856102
1	-3.63764	GO:0060252	M1	1	0	GO Biological Processes	19	positive regulation of glial cell proliferation	-3.63764	24.78852	8.303425	30242	20	183	3	1.639344	0.938686	1956|3569|7124	EGFR|IL6|TNF	-2.56974	0	74	0	0	-10.0127	55.0856102

1	-3.58562	GO:0014009	M1	1	0	GO Biological Processes	19	glial cell proliferation	-3.58562	12.96132	6.670552	30242	51	183	4	2.185792	1.080886	1128|1956|3569|7124	CHRM1|EGFR|IL6|TNF	-2.5219	0	74	0	0	-10.0127	55.0856102
1	-3.4286	GO:0045599	M1	1	0	GO Biological Processes	19	negative regulation of fat cell differentiation	-3.4286	11.80406	6.314145	30242	56	183	4	2.185792	1.080886	3569|7040|7124|59341	IL6|TGFB1|TNF|TRPV4	-2.38276	0	74	0	0	-10.0127	55.0856102
1	-3.18136	GO:0045453	M1	1	0	GO Biological Processes	19	bone resorption	-3.18136	10.16965	5.774413	30242	65	183	4	2.185792	1.080886	154|760|1956|3569	ADRB2|CA2|EGFR|IL6	-2.15944	0	74	0	0	-10.0127	55.0856102
1	-2.78049	GO:0050768	M1	1	0	GO Biological Processes	19	negative regulation of neurogenesis	-2.78049	5.90203	4.536058	30242	140	183	5	2.73224	1.205087	1814|2147|3569|7124|7157	DRD3|F2|IL6|TNF|TP53	-1.79909	0	74	0	0	-10.0127	55.0856102
1	-2.71369	GO:0051961	M1	1	0	GO Biological Processes	19	negative regulation of nervous system development	-2.71369	5.698511	4.425059	30242	145	183	5	2.73224	1.205087	1814|2147|3569|7124|7157	DRD3|F2|IL6|TNF|TP53	-1.74017	0	74	0	0	-10.0127	55.0856102
1	-2.6188	GO:0045124	M1	1	0	GO Biological Processes	19	regulation of bone resorption	-2.6188	11.26751	5.317883	30242	44	183	3	1.639344	0.938686	760|1956|3569	CA2|EGFR|IL6	-1.65683	0	74	0	0	-10.0127	55.0856102
1	-2.51055	GO:1903053	M1	1	0	GO Biological Processes	19	regulation of extracellular matrix organization	-2.51055	10.32855	5.046739	30242	48	183	3	1.639344	0.938686	3569|7040|7132	IL6|TGFB1|TNFRSF1A	-1.55686	0	74	0	0	-10.0127	55.0856102
1	-2.43567	GO:0046850	M1	1	0	GO Biological Processes	19	regulation of bone remodeling	-2.43567	9.72099	4.863495	30242	51	183	3	1.639344	0.938686	760|1956|3569	CA2|EGFR|IL6	-1.49059	0	74	0	0	-10.0127	55.0856102
1	-9.98118	GO:0032787	M1	1	0	GO Biological Processes	19	monocarboxylic acid metabolic process	-9.98118	5.832594	9.289338	30242	595	183	21	11.47541	2.356083	351|427|1268|1543|1544|1545|1557|1645|2166|3356|3643|5465|5467|5468|5742|5743|6916|7132|7157|7364|51179	APP|ASAH1|CNR1|CYP1A1|CYP1A2|CYP1B1|CYP2C19|AKR1C1|FAAH|HTR2A|INSR|PPARA|PPARD|PPARG|PTGS1|PTGS2|TBXAS1|TNFRSF1A|TP53|UGT2B7|HAO2	-8.29709	0	75	1	1	-9.98118	55.0856102
1	-9.59847	GO:0006690	M1	1	0	GO Biological Processes	19	icosanoid metabolic process	-9.59847	14.77907	11.94781	30242	123	183	11	6.010929	1.757049	1543|1544|1545|1557|1645|2166|4363|5742|5743|6916|7132	CYP1A1|CYP1A2|CYP1B1|CYP2C19|AKR1C1|FAAH|ABCC1|PTGS1|PTGS2|TBXAS1|TNFRSF1A	-7.94656	0	75	0	0	-9.98118	55.0856102
1	-8.6297	GO:0006631	M1	1	0	GO Biological Processes	19	fatty acid metabolic process	-8.6297	6.779767	8.963803	30242	390	183	16	8.743169	2.088053	427|1268|1543|1544|1545|1557|1645|2166|5465|5467|5468|5742|5743|6916|7132|51179	ASAH1|CNR1|CYP1A1|CYP1A2|CYP1B1|CYP2C19|AKR1C1|FAAH|PPARA|PPARD|PPARG|PTGS1|PTGS2|TBXAS1|TNFRSF1A|HAO2	-7.03469	0	75	0	0	-9.98118	55.0856102
1	-8.61802	GO:0033559	M1	1	0	GO Biological Processes	19	unsaturated fatty acid metabolic process	-8.61802	14.24628	11.15292	30242	116	183	10	5.464481	1.680144	1543|1544|1545|1557|1645|2166|5742|5743|6916|7132	CYP1A1|CYP1A2|CYP1B1|CYP2C19|AKR1C1|FAAH|PTGS1|PTGS2|TBXAS1|TNFRSF1A	-7.02518	0	75	0	0	-9.98118	55.0856102
1	-8.58054	GO:0019369	M1	1	0	GO Biological Processes	19	arachidonic acid metabolic process	-8.58054	22.40771	12.84255	30242	59	183	8	4.371585	1.511428	1543|1544|1545|1557|2166|5742|5743|6916	CYP1A1|CYP1A2|CYP1B1|CYP2C19|FAAH|PTGS1|PTGS2|TBXAS1	-6.99308	0	75	0	0	-9.98118	55.0856102
1	-7.45652	GO:0120254	M1	1	0	GO Biological Processes	19	olefinic compound metabolic process	-7.45652	10.8011	9.483159	30242	153	183	10	5.464481	1.680144	1543|1544|1545|1557|1645|2166|2902|5742|5743|6916	CYP1A1|CYP1A2|CYP1B1|CYP2C19|AKR1C1|FAAH|GRIN1|PTGS1|PTGS2|TBXAS1	-5.97487	0	75	0	0	-9.98118	55.0856102

1	-6.36765	GO:0001676	M1	1	0	GO Biological Processes	19	long-chain fatty acid metabolic process	-6.36765	11.80406	8.937844	30242	112	183	8	4.371585	1.511428	1543|1544|1545|1557|2166|5742|5743|6916	CYP1A1|CYP1A2|CYP1B1|CYP2C19|FAAH|PTGS1|PTGS2|TBXAS1	-4.98923	0	75	0	0	-9.98118	55.0856102
1	-4.928	GO:0006692	M1	1	0	GO Biological Processes	19	prostanoid metabolic process	-4.928	16.86294	8.670916	30242	49	183	5	2.73224	1.205087	1645|5742|5743|6916|7132	AKR1C1|PTGS1|PTGS2|TBXAS1|TNFRSF1A	-3.70236	0	75	0	0	-9.98118	55.0856102
1	-4.928	GO:0006693	M1	1	0	GO Biological Processes	19	prostaglandin metabolic process	-4.928	16.86294	8.670916	30242	49	183	5	2.73224	1.205087	1645|5742|5743|6916|7132	AKR1C1|PTGS1|PTGS2|TBXAS1|TNFRSF1A	-3.70236	0	75	0	0	-9.98118	55.0856102
1	-4.74896	GO:0019371	M1	1	0	GO Biological Processes	19	cyclooxygenase pathway	-4.74896	55.08561	12.6619	30242	9	183	3	1.639344	0.938686	5742|5743|6916	PTGS1|PTGS2|TBXAS1	-3.54861	0	75	0	0	-9.98118	55.0856102
1	-3.20691	GO:0070542	M1	1	0	GO Biological Processes	19	response to fatty acid	-3.20691	10.32855	5.829017	30242	64	183	4	2.185792	1.080886	1645|5444|5743|6916	AKR1C1|PON1|PTGS2|TBXAS1	-2.18207	0	75	0	0	-9.98118	55.0856102
1	-3.06307	GO:0001516	M1	1	0	GO Biological Processes	19	prostaglandin biosynthetic process	-3.06307	15.9926	6.516457	30242	31	183	3	1.639344	0.938686	5742|5743|6916	PTGS1|PTGS2|TBXAS1	-2.05432	0	75	0	0	-9.98118	55.0856102
1	-3.06307	GO:0046457	M1	1	0	GO Biological Processes	19	prostanoid biosynthetic process	-3.06307	15.9926	6.516457	30242	31	183	3	1.639344	0.938686	5742|5743|6916	PTGS1|PTGS2|TBXAS1	-2.05432	0	75	0	0	-9.98118	55.0856102
1	-2.49395	GO:0006633	M1	1	0	GO Biological Processes	19	fatty acid biosynthetic process	-2.49395	5.069228	4.064531	30242	163	183	5	2.73224	1.205087	1543|1544|5742|5743|6916	CYP1A1|CYP1A2|PTGS1|PTGS2|TBXAS1	-1.54175	0	75	0	0	-9.98118	55.0856102
1	-2.43567	GO:0006636	M1	1	0	GO Biological Processes	19	unsaturated fatty acid biosynthetic process	-2.43567	9.72099	4.863495	30242	51	183	3	1.639344	0.938686	5742|5743|6916	PTGS1|PTGS2|TBXAS1	-1.49059	0	75	0	0	-9.98118	55.0856102
1	-2.32104	GO:0046456	M1	1	0	GO Biological Processes	19	icosanoid biosynthetic process	-2.32104	8.853044	4.589503	30242	56	183	3	1.639344	0.938686	5742|5743|6916	PTGS1|PTGS2|TBXAS1	-1.38595	0	75	0	0	-9.98118	55.0856102
1	-2.00274	GO:0072330	M1	1	0	GO Biological Processes	19	monocarboxylic acid biosynthetic process	-2.00274	3.861141	3.277323	30242	214	183	5	2.73224	1.205087	1543|1544|5742|5743|6916	CYP1A1|CYP1A2|PTGS1|PTGS2|TBXAS1	-1.09008	0	75	0	0	-9.98118	55.0856102
1	-9.93528	GO:1904996	M1	1	0	GO Biological Processes	19	positive regulation of leukocyte adhesion to vascular endothelial cell	-9.93528	46.27191	17.6689	30242	25	183	7	3.825137	1.417845	1991|3383|3569|5970|6401|7124|729230	ELANE|ICAM1|IL6|RELA|SELE|TNF|CCR2	-8.25649	0	76	1	1	-9.93528	46.27191257
1	-8.37985	GO:1904994	M1	1	0	GO Biological Processes	19	regulation of leukocyte adhesion to vascular endothelial cell	-8.37985	28.91995	13.78679	30242	40	183	7	3.825137	1.417845	1991|3383|3569|5970|6401|7124|729230	ELANE|ICAM1|IL6|RELA|SELE|TNF|CCR2	-6.81115	0	76	0	0	-9.93528	46.27191257
1	-7.26575	GO:0061756	M1	1	0	GO Biological Processes	19	leukocyte adhesion to vascular endothelial cell	-7.26575	20.2947	11.3767	30242	57	183	7	3.825137	1.417845	1991|3383|3569|5970|6401|7124|729230	ELANE|ICAM1|IL6|RELA|SELE|TNF|CCR2	-5.80696	0	76	0	0	-9.93528	46.27191257
1	-5.54342	GO:0002691	M1	1	0	GO Biological Processes	19	regulation of cellular extravasation	-5.54342	22.332	10.13047	30242	37	183	5	2.73224	1.205087	1991|3383|5734|6401|729230	ELANE|ICAM1|PTGER4|SELE|CCR2	-4.23786	0	76	0	0	-9.93528	46.27191257
1	-4.45963	GO:1903238	M1	1	0	GO Biological Processes	19	positive regulation of leukocyte tethering or rolling	-4.45963	45.07004	11.40644	30242	11	183	3	1.639344	0.938686	1991|6401|729230	ELANE|SELE|CCR2	-3.30278	0	76	0	0	-9.93528	46.27191257
1	-4.33459	GO:0050901	M1	1	0	GO Biological Processes	19	leukocyte tethering or rolling	-4.33459	20.03113	8.534729	30242	33	183	4	2.185792	1.080886	1991|6401|7124|729230	ELANE|SELE|TNF|CCR2	-3.18937	0	76	0	0	-9.93528	46.27191257
1	-3.93862	GO:1903236	M1	1	0	GO Biological Processes	19	regulation of leukocyte tethering or rolling	-3.93862	30.98566	9.360942	30242	16	183	3	1.639344	0.938686	1991|6401|729230	ELANE|SELE|CCR2	-2.83536	0	76	0	0	-9.93528	46.27191257
1	-3.70629	GO:0002523	M1	1	0	GO Biological Processes	19	leukocyte migration involved in inflammatory response	-3.70629	26.09318	8.536899	30242	19	183	3	1.639344	0.938686	1991|6401|114548	ELANE|SELE|NLRP3	-2.63055	0	76	0	0	-9.93528	46.27191257
1	-2.56339	GO:0007157	M1	1	0	GO Biological Processes	19	heterophilic cell-cell adhesion via plasma membrane cell adhesion molecules	-2.56339	10.77762	5.178139	30242	46	183	3	1.639344	0.938686	3383|3683|6401	ICAM1|ITGAL|SELE	-1.6072	0	76	0	0	-9.93528	46.27191257

<table>
<tr><td>1</td><td>-9.87332</td><td>GO:0048608</td><td>M1</td><td>1</td><td>0</td><td>GO Biological Processes</td><td>19</td><td>reproductive structure development</td><td>-9.87332</td><td>6.999113</td><td>9.718201</td><td>30242</td><td>425</td><td>183</td><td>18</td><td>9.836066</td><td>2.201413</td><td>100|367|595|596|836|1588|2099|2263|3383|4221|4914|5159|5241|5467|5468|5743|7046|7421</td><td>ADA|AR|CCND1|BCL2|CASP3|CYP19A1|ESR1|FGFR2|ICAM1|MEN1|NTRK1|PDGFRB|PGR|PPARD|PPARG|PTGS2|TGFBR1|VDR</td><td>-8.20497</td><td>0</td><td>77</td><td>1</td><td>1</td><td>-9.87332</td><td>94.43247463</td></tr>
<tr><td>1</td><td>-9.824</td><td>GO:0061458</td><td>M1</td><td>1</td><td>0</td><td>GO Biological Processes</td><td>19</td><td>reproductive system development</td><td>-9.824</td><td>6.950054</td><td>9.673174</td><td>30242</td><td>428</td><td>183</td><td>18</td><td>9.836066</td><td>2.201413</td><td>100|367|595|596|836|1588|2099|2263|3383|4221|4914|5159|5241|5467|5468|5743|7046|7421</td><td>ADA|AR|CCND1|BCL2|CASP3|CYP19A1|ESR1|FGFR2|ICAM1|MEN1|NTRK1|PDGFRB|PGR|PPARD|PPARG|PTGS2|TGFBR1|VDR</td><td>-8.1595</td><td>0</td><td>77</td><td>0</td><td>0</td><td>-9.87332</td><td>94.43247463</td></tr>
<tr><td>1</td><td>-7.349</td><td>GO:0060745</td><td>M1</td><td>1</td><td>0</td><td>GO Biological Processes</td><td>19</td><td>mammary gland branching involved in pregnancy</td><td>-7.349</td><td>94.43247</td><td>19.28982</td><td>30242</td><td>7</td><td>183</td><td>4</td><td>2.185792</td><td>1.080886</td><td>367|2099|5241|7421</td><td>AR|ESR1|PGR|VDR</td><td>-5.87776</td><td>0</td><td>77</td><td>0</td><td>0</td><td>-9.87332</td><td>94.43247463</td></tr>
<tr><td>1</td><td>-7.31194</td><td>GO:0022612</td><td>M1</td><td>1</td><td>0</td><td>GO Biological Processes</td><td>19</td><td>gland morphogenesis</td><td>-7.31194</td><td>12.49842</td><td>9.806155</td><td>30242</td><td>119</td><td>183</td><td>9</td><td>4.918033</td><td>1.598524</td><td>367|596|2099|2263|3569|5241|7040|7124|7421</td><td>AR|BCL2|ESR1|FGFR2|IL6|PGR|TGFB1|TNF|VDR</td><td>-5.84593</td><td>0</td><td>77</td><td>0</td><td>0</td><td>-9.87332</td><td>94.43247463</td></tr>
<tr><td>1</td><td>-7.04539</td><td>GO:0030522</td><td>M1</td><td>1</td><td>0</td><td>GO Biological Processes</td><td>19</td><td>intracellular receptor signaling pathway</td><td>-7.04539</td><td>7.455195</td><td>8.25087</td><td>30242</td><td>266</td><td>183</td><td>12</td><td>6.557377</td><td>1.829835</td><td>367|2099|2100|2908|4792|5241|5465|5467|5468|5970|7099|7421</td><td>AR|ESR1|ESR2|NR3C1|NFKBIA|PGR|PPARA|PPARD|PPARG|RELA|TLR4|VDR</td><td>-5.60756</td><td>0</td><td>77</td><td>0</td><td>0</td><td>-9.87332</td><td>94.43247463</td></tr>
<tr><td>1</td><td>-6.93122</td><td>GO:0008406</td><td>M1</td><td>1</td><td>0</td><td>GO Biological Processes</td><td>19</td><td>gonad development</td><td>-6.93122</td><td>8.225453</td><td>8.411724</td><td>30242</td><td>221</td><td>183</td><td>11</td><td>6.010929</td><td>1.757049</td><td>367|595|596|836|1588|2099|3383|4914|5159|5241|7046</td><td>AR|CCND1|BCL2|CASP3|CYP19A1|ESR1|ICAM1|NTRK1|PDGFRB|PGR|TGFBR1</td><td>-5.49941</td><td>0</td><td>77</td><td>0</td><td>0</td><td>-9.87332</td><td>94.43247463</td></tr>
<tr><td>1</td><td>-6.8139</td><td>GO:0045137</td><td>M1</td><td>1</td><td>0</td><td>GO Biological Processes</td><td>19</td><td>development of primary sexual characteristics</td><td>-6.8139</td><td>8.00804</td><td>8.269451</td><td>30242</td><td>227</td><td>183</td><td>11</td><td>6.010929</td><td>1.757049</td><td>367|595|596|836|1588|2099|3383|4914|5159|5241|7046</td><td>AR|CCND1|BCL2|CASP3|CYP19A1|ESR1|ICAM1|NTRK1|PDGFRB|PGR|TGFBR1</td><td>-5.3945</td><td>0</td><td>77</td><td>0</td><td>0</td><td>-9.87332</td><td>94.43247463</td></tr>
<tr><td>1</td><td>-6.43006</td><td>GO:0043627</td><td>M1</td><td>1</td><td>0</td><td>GO Biological Processes</td><td>19</td><td>response to estrogen</td><td>-6.43006</td><td>15.42397</td><td>9.758561</td><td>30242</td><td>75</td><td>183</td><td>7</td><td>3.825137</td><td>1.417845</td><td>367|595|760|2099|2155|4986|5159</td><td>AR|CCND1|CA2|ESR1|F7|OPRK1|PDGFRB</td><td>-5.04498</td><td>0</td><td>77</td><td>0</td><td>0</td><td>-9.87332</td><td>94.43247463</td></tr>
<tr><td>1</td><td>-6.11526</td><td>GO:0060562</td><td>M1</td><td>1</td><td>0</td><td>GO Biological Processes</td><td>19</td><td>epithelial tube morphogenesis</td><td>-6.11526</td><td>6.101791</td><td>7.215088</td><td>30242</td><td>325</td><td>183</td><td>12</td><td>6.557377</td><td>1.829835</td><td>367|596|836|1909|2099|2263|5241|5293|5979|7040|7124|7421</td><td>AR|BCL2|CASP3|EDNRA|ESR1|FGFR2|PGR|PIK3CD|RET|TGFB1|TNF|VDR</td><td>-4.75813</td><td>0</td><td>77</td><td>0</td><td>0</td><td>-9.87332</td><td>94.43247463</td></tr>
<tr><td>1</td><td>-6.01407</td><td>GO:0060603</td><td>M1</td><td>1</td><td>0</td><td>GO Biological Processes</td><td>19</td><td>mammary gland duct morphogenesis</td><td>-6.01407</td><td>27.54281</td><td>11.34891</td><td>30242</td><td>30</td><td>183</td><td>5</td><td>2.73224</td><td>1.205087</td><td>367|2099|2263|5241|7421</td><td>AR|ESR1|FGFR2|PGR|VDR</td><td>-4.66566</td><td>0</td><td>77</td><td>0</td><td>0</td><td>-9.87332</td><td>94.43247463</td></tr>
<tr><td>1</td><td>-5.97197</td><td>GO:0007548</td><td>M1</td><td>1</td><td>0</td><td>GO Biological Processes</td><td>19</td><td>sex differentiation</td><td>-5.97197</td><td>6.586323</td><td>7.27449</td><td>30242</td><td>276</td><td>183</td><td>11</td><td>6.010929</td><td>1.757049</td><td>367|595|596|836|1588|2099|3383|4914|5159|5241|7046</td><td>AR|CCND1|BCL2|CASP3|CYP19A1|ESR1|ICAM1|NTRK1|PDGFRB|PGR|TGFBR1</td><td>-4.62602</td><td>0</td><td>77</td><td>0</td><td>0</td><td>-9.87332</td><td>94.43247463</td></tr>
<tr><td>1</td><td>-5.70272</td><td>GO:0030879</td><td>M1</td><td>1</td><td>0</td><td>GO Biological Processes</td><td>19</td><td>mammary gland development</td><td>-5.70272</td><td>9.650034</td><td>7.917629</td><td>30242</td><td>137</td><td>183</td><td>8</td><td>4.371585</td><td>1.511428</td><td>367|595|1723|2099|2263|5241|6531|7421</td><td>AR|CCND1|DHODH|ESR1|FGFR2|PGR|SLC6A3|VDR</td><td>-4.38122</td><td>0</td><td>77</td><td>0</td><td>0</td><td>-9.87332</td><td>94.43247463</td></tr>
<tr><td>1</td><td>-5.60904</td><td>GO:0008584</td><td>M1</td><td>1</td><td>0</td><td>GO Biological Processes</td><td>19</td><td>male gonad development</td><td>-5.60904</td><td>9.376274</td><td>7.778688</td><td>30242</td><td>141</td><td>183</td><td>8</td><td>4.371585</td><td>1.511428</td><td>367|595|596|2099|3383|4914|5159|7046</td><td>AR|CCND1|BCL2|ESR1|ICAM1|NTRK1|PDGFRB|TGFBR1</td><td>-4.29955</td><td>0</td><td>77</td><td>0</td><td>0</td><td>-9.87332</td><td>94.43247463</td></tr>
<tr><td>1</td><td>-5.58608</td><td>GO:0046546</td><td>M1</td><td>1</td><td>0</td><td>GO Biological Processes</td><td>19</td><td>development of primary male sexual characteristics</td><td>-5.58608</td><td>9.310244</td><td>7.744815</td><td>30242</td><td>142</td><td>183</td><td>8</td><td>4.371585</td><td>1.511428</td><td>367|595|596|2099|3383|4914|5159|7046</td><td>AR|CCND1|BCL2|ESR1|ICAM1|NTRK1|PDGFRB|TGFBR1</td><td>-4.27996</td><td>0</td><td>77</td><td>0</td><td>0</td><td>-9.87332</td><td>94.43247463</td></tr>
</table>

<table>
<tr><td>1</td><td>-5.48086</td><td>GO:0001763</td><td>M1</td><td>1</td><td>0</td><td>GO Biological Processes</td><td>19</td><td>morphogenesis of a branching structure</td><td>-5.48086</td><td>7.588324</td><td>7.220146</td><td>30242</td><td>196</td><td>183</td><td>9</td><td>4.918033</td><td>1.598524</td><td>367|596|1813|1909|2099|2263|5241|7124|7421</td><td>AR|BCL2|DRD2|EDNRA|ESR1|FGFR2|PGR|TNF|VDR</td><td>-4.18415</td><td>0</td><td>77</td><td>0</td><td>0</td><td>-9.87332</td><td>94.43247463</td></tr>
<tr><td>1</td><td>-5.47854</td><td>GO:0061180</td><td>M1</td><td>1</td><td>0</td><td>GO Biological Processes</td><td>19</td><td>mammary gland epithelium development</td><td>-5.47854</td><td>14.79912</td><td>8.822699</td><td>30242</td><td>67</td><td>183</td><td>6</td><td>3.278689</td><td>1.316393</td><td>367|595|2099|2263|5241|7421</td><td>AR|CCND1|ESR1|FGFR2|PGR|VDR</td><td>-4.18238</td><td>0</td><td>77</td><td>0</td><td>0</td><td>-9.87332</td><td>94.43247463</td></tr>
<tr><td>1</td><td>-5.40326</td><td>GO:0042698</td><td>M1</td><td>1</td><td>0</td><td>GO Biological Processes</td><td>19</td><td>ovulation cycle</td><td>-5.40326</td><td>14.37016</td><td>8.675375</td><td>30242</td><td>69</td><td>183</td><td>6</td><td>3.278689</td><td>1.316393</td><td>836|1956|2099|4986|4988|5241</td><td>CASP3|EGFR|ESR1|OPRK1|OPRM1|PGR</td><td>-4.1163</td><td>0</td><td>77</td><td>0</td><td>0</td><td>-9.87332</td><td>94.43247463</td></tr>
<tr><td>1</td><td>-5.38734</td><td>GO:0048754</td><td>M1</td><td>1</td><td>0</td><td>GO Biological Processes</td><td>19</td><td>branching morphogenesis of an epithelial tube</td><td>-5.38734</td><td>8.755329</td><td>7.454298</td><td>30242</td><td>151</td><td>183</td><td>8</td><td>4.371585</td><td>1.511428</td><td>367|596|1909|2099|2263|5241|7124|7421</td><td>AR|BCL2|EDNRA|ESR1|FGFR2|PGR|TNF|VDR</td><td>-4.10252</td><td>0</td><td>77</td><td>0</td><td>0</td><td>-9.87332</td><td>94.43247463</td></tr>
<tr><td>1</td><td>-5.33923</td><td>GO:0071383</td><td>M1</td><td>1</td><td>0</td><td>GO Biological Processes</td><td>19</td><td>cellular response to steroid hormone stimulus</td><td>-5.33923</td><td>7.290743</td><td>7.034251</td><td>30242</td><td>204</td><td>183</td><td>9</td><td>4.918033</td><td>1.598524</td><td>367|1956|2099|2100|2908|3383|5241|5465|5467</td><td>AR|EGFR|ESR1|ESR2|NR3C1|ICAM1|PGR|PPARA|PPARD</td><td>-4.06545</td><td>0</td><td>77</td><td>0</td><td>0</td><td>-9.87332</td><td>94.43247463</td></tr>
<tr><td>1</td><td>-5.21212</td><td>GO:0060443</td><td>M1</td><td>1</td><td>0</td><td>GO Biological Processes</td><td>19</td><td>mammary gland morphogenesis</td><td>-5.21212</td><td>19.21591</td><td>9.326639</td><td>30242</td><td>43</td><td>183</td><td>5</td><td>2.73224</td><td>1.205087</td><td>367|2099|2263|5241|7421</td><td>AR|ESR1|FGFR2|PGR|VDR</td><td>-3.95416</td><td>0</td><td>77</td><td>0</td><td>0</td><td>-9.87332</td><td>94.43247463</td></tr>
<tr><td>1</td><td>-5.10341</td><td>GO:0046661</td><td>M1</td><td>1</td><td>0</td><td>GO Biological Processes</td><td>19</td><td>male sex differentiation</td><td>-5.10341</td><td>8.012452</td><td>7.047434</td><td>30242</td><td>165</td><td>183</td><td>8</td><td>4.371585</td><td>1.511428</td><td>367|595|596|2099|3383|4914|5159|7046</td><td>AR|CCND1|BCL2|ESR1|ICAM1|NTRK1|PDGFRB|TGFBR1</td><td>-3.8573</td><td>0</td><td>77</td><td>0</td><td>0</td><td>-9.87332</td><td>94.43247463</td></tr>
<tr><td>1</td><td>-5.05972</td><td>GO:0060444</td><td>M1</td><td>1</td><td>0</td><td>GO Biological Processes</td><td>19</td><td>branching involved in mammary gland duct morphogenesis</td><td>-5.05972</td><td>30.0467</td><td>10.63401</td><td>30242</td><td>22</td><td>183</td><td>4</td><td>2.185792</td><td>1.080886</td><td>367|2099|5241|7421</td><td>AR|ESR1|PGR|VDR</td><td>-3.8175</td><td>0</td><td>77</td><td>0</td><td>0</td><td>-9.87332</td><td>94.43247463</td></tr>
<tr><td>1</td><td>-4.79345</td><td>GO:0061138</td><td>M1</td><td>1</td><td>0</td><td>GO Biological Processes</td><td>19</td><td>morphogenesis of a branching epithelium</td><td>-4.79345</td><td>7.264037</td><td>6.613507</td><td>30242</td><td>182</td><td>183</td><td>8</td><td>4.371585</td><td>1.511428</td><td>367|596|1909|2099|2263|5241|7124|7421</td><td>AR|BCL2|EDNRA|ESR1|FGFR2|PGR|TNF|VDR</td><td>-3.58602</td><td>0</td><td>77</td><td>0</td><td>0</td><td>-9.87332</td><td>94.43247463</td></tr>
<tr><td>1</td><td>-4.69813</td><td>GO:0043401</td><td>M1</td><td>1</td><td>0</td><td>GO Biological Processes</td><td>19</td><td>steroid hormone mediated signaling pathway</td><td>-4.69813</td><td>8.505866</td><td>6.845105</td><td>30242</td><td>136</td><td>183</td><td>7</td><td>3.825137</td><td>1.417845</td><td>367|2099|2100|2908|5241|5465|5467</td><td>AR|ESR1|ESR2|NR3C1|PGR|PPARA|PPARD</td><td>-3.50345</td><td>0</td><td>77</td><td>0</td><td>0</td><td>-9.87332</td><td>94.43247463</td></tr>
<tr><td>1</td><td>-4.65889</td><td>GO:0009755</td><td>M1</td><td>1</td><td>0</td><td>GO Biological Processes</td><td>19</td><td>hormone-mediated signaling pathway</td><td>-4.65889</td><td>6.958182</td><td>6.428213</td><td>30242</td><td>190</td><td>183</td><td>8</td><td>4.371585</td><td>1.511428</td><td>367|2099|2100|2908|5241|5465|5467|5468</td><td>AR|ESR1|ESR2|NR3C1|PGR|PPARA|PPARD|PPARG</td><td>-3.46766</td><td>0</td><td>77</td><td>0</td><td>0</td><td>-9.87332</td><td>94.43247463</td></tr>
<tr><td>1</td><td>-4.6102</td><td>GO:0048729</td><td>M1</td><td>1</td><td>0</td><td>GO Biological Processes</td><td>19</td><td>tissue morphogenesis</td><td>-4.6102</td><td>3.768071</td><td>5.406878</td><td>30242</td><td>614</td><td>183</td><td>14</td><td>7.650273</td><td>1.964856</td><td>367|596|836|1909|2099|2263|4846|5241|5293|5979|7040|7046|7124|7421</td><td>AR|BCL2|CASP3|EDNRA|ESR1|FGFR2|NOS3|PGR|PIK3CD|RET|TGFB1|TGFBR1|TNF|VDR</td><td>-3.42579</td><td>0</td><td>77</td><td>0</td><td>0</td><td>-9.87332</td><td>94.43247463</td></tr>
<tr><td>1</td><td>-4.59938</td><td>GO:0008585</td><td>M1</td><td>1</td><td>0</td><td>GO Biological Processes</td><td>19</td><td>female gonad development</td><td>-4.59938</td><td>10.43727</td><td>7.188241</td><td>30242</td><td>95</td><td>183</td><td>6</td><td>3.278689</td><td>1.316393</td><td>596|836|1588|2099|3383|5241</td><td>BCL2|CASP3|CYP19A1|ESR1|ICAM1|PGR</td><td>-3.4183</td><td>0</td><td>77</td><td>0</td><td>0</td><td>-9.87332</td><td>94.43247463</td></tr>
<tr><td>1</td><td>-4.596</td><td>GO:0060736</td><td>M1</td><td>1</td><td>0</td><td>GO Biological Processes</td><td>19</td><td>prostate gland growth</td><td>-4.596</td><td>49.57705</td><td>11.98765</td><td>30242</td><td>10</td><td>183</td><td>3</td><td>1.639344</td><td>0.938686</td><td>367|2099|2263</td><td>AR|ESR1|FGFR2</td><td>-3.4183</td><td>0</td><td>77</td><td>0</td><td>0</td><td>-9.87332</td><td>94.43247463</td></tr>
<tr><td>1</td><td>-4.473</td><td>GO:0046545</td><td>M1</td><td>1</td><td>0</td><td>GO Biological Processes</td><td>19</td><td>development of primary female sexual characteristics</td><td>-4.473</td><td>9.91541</td><td>6.967735</td><td>30242</td><td>100</td><td>183</td><td>6</td><td>3.278689</td><td>1.316393</td><td>596|836|1588|2099|3383|5241</td><td>BCL2|CASP3|CYP19A1|ESR1|ICAM1|PGR</td><td>-3.31285</td><td>0</td><td>77</td><td>0</td><td>0</td><td>-9.87332</td><td>94.43247463</td></tr>
<tr><td>1</td><td>-4.20273</td><td>GO:0002009</td><td>M1</td><td>1</td><td>0</td><td>GO Biological Processes</td><td>19</td><td>morphogenesis of an epithelium</td><td>-4.20273</td><td>3.934686</td><td>5.18391</td><td>30242</td><td>504</td><td>183</td><td>12</td><td>6.557377</td><td>1.829835</td><td>367|596|836|1909|2099|2263|5241|5293|5979|7040|7124|7421</td><td>AR|BCL2|CASP3|EDNRA|ESR1|FGFR2|PGR|PIK3CD|RET|TGFB1|TNF|VDR</td><td>-3.06971</td><td>0</td><td>77</td><td>0</td><td>0</td><td>-9.87332</td><td>94.43247463</td></tr>
<tr><td>1</td><td>-4.15361</td><td>GO:0046660</td><td>M1</td><td>1</td><td>0</td><td>GO Biological Processes</td><td>19</td><td>female sex differentiation</td><td>-4.15361</td><td>8.697728</td><td>6.424897</td><td>30242</td><td>114</td><td>183</td><td>6</td><td>3.278689</td><td>1.316393</td><td>596|836|1588|2099|3383|5241</td><td>BCL2|CASP3|CYP19A1|ESR1|ICAM1|PGR</td><td>-3.02508</td><td>0</td><td>77</td><td>0</td><td>0</td><td>-9.87332</td><td>94.43247463</td></tr>
<tr><td>1</td><td>-3.77899</td><td>GO:0060749</td><td>M1</td><td>1</td><td>0</td><td>GO Biological Processes</td><td>19</td><td>mammary gland alveolus development</td><td>-3.77899</td><td>27.54281</td><td>8.789081</td><td>30242</td><td>18</td><td>183</td><td>3</td><td>1.639344</td><td>0.938686</td><td>367|595|2099</td><td>AR|CCND1|ESR1</td><td>-2.69427</td><td>0</td><td>77</td><td>0</td><td>0</td><td>-9.87332</td><td>94.43247463</td></tr>
</table>

1	-3.77899	GO:0061377	M1	1	0	GO Biological Processes	19	mammary gland lobule development	-3.77899	27.54281	8.789081	30242	18	183	3	1.639344	0.938686	367|595|2099	AR|CCND1|ESR1	-2.69427	0	77	0	0	-9.87332	94.43247463
1	-3.61905	GO:0060688	M1	1	0	GO Biological Processes	19	regulation of morphogenesis of a branching structure	-3.61905	13.22055	6.74786	30242	50	183	4	2.185792	1.080886	367|2099|2263|7124	AR|ESR1|FGFR2|TNF	-2.55243	0	77	0	0	-9.87332	94.43247463
1	-3.34249	GO:0060740	M1	1	0	GO Biological Processes	19	prostate gland epithelium morphogenesis	-3.34249	19.83082	7.349367	30242	25	183	3	1.639344	0.938686	367|2099|2263	AR|ESR1|FGFR2	-2.30396	0	77	0	0	-9.87332	94.43247463
1	-3.24195	GO:0060512	M1	1	0	GO Biological Processes	19	prostate gland morphogenesis	-3.24195	18.36187	7.04212	30242	27	183	3	1.639344	0.938686	367|2099|2263	AR|ESR1|FGFR2	-2.21358	0	77	0	0	-9.87332	94.43247463
1	-3.18136	GO:1905330	M1	1	0	GO Biological Processes	19	regulation of morphogenesis of an epithelium	-3.18136	10.16965	5.774413	30242	65	183	4	2.185792	1.080886	367|2099|5293|7124	AR|ESR1|PIK3CD|TNF	-2.15944	0	77	0	0	-9.87332	94.43247463
1	-3.14493	GO:0030518	M1	1	0	GO Biological Processes	19	intracellular steroid hormone receptor signaling pathway	-3.14493	7.123139	5.155476	30242	116	183	5	2.73224	1.205087	367|2099|2100|2908|5241	AR|ESR1|ESR2|NR3C1|PGR	-2.12706	0	77	0	0	-9.87332	94.43247463
1	-2.59076	GO:0022602	M1	1	0	GO Biological Processes	19	ovulation cycle process	-2.59076	11.01712	5.246911	30242	45	183	3	1.639344	0.938686	836|2099|5241	CASP3|ESR1|PGR	-1.63232	0	77	0	0	-9.87332	94.43247463
1	-2.59076	GO:0030850	M1	1	0	GO Biological Processes	19	prostate gland development	-2.59076	11.01712	5.246911	30242	45	183	3	1.639344	0.938686	367|2099|2263	AR|ESR1|FGFR2	-1.63232	0	77	0	0	-9.87332	94.43247463
1	-2.38839	GO:0001541	M1	1	0	GO Biological Processes	19	ovarian follicle development	-2.38839	9.35416	4.749553	30242	53	183	3	1.639344	0.938686	596|2099|3383	BCL2|ESR1|ICAM1	-1.44695	0	77	0	0	-9.87332	94.43247463
1	-2.36548	GO:0030520	M1	1	0	GO Biological Processes	19	intracellular estrogen receptor signaling pathway	-2.36548	9.180935	4.694821	30242	54	183	3	1.639344	0.938686	367|2099|2100	AR|ESR1|ESR2	-1.4267	0	77	0	0	-9.87332	94.43247463
1	-9.66495	GO:0045986	M1	1	0	GO Biological Processes	19	negative regulation of smooth muscle contraction	-9.66495	66.10273	19.67812	30242	15	183	6	3.278689	1.316393	134|152|154|3778|5743|10203	ADORA1|ADRA2C|ADRB2|KCNMA1|PTGS2|CALCRL	-8.0093	0	78	1	1	-9.66495	66.10273224
1	-8.37772	GO:0045932	M1	1	0	GO Biological Processes	19	negative regulation of muscle contraction	-8.37772	43.11048	15.76342	30242	23	183	6	3.278689	1.316393	134|152|154|3778|5743|10203	ADORA1|ADRA2C|ADRB2|KCNMA1|PTGS2|CALCRL	-6.81115	0	78	0	0	-9.66495	66.10273224
1	-4.28228	GO:0090075	M1	1	0	GO Biological Processes	19	relaxation of muscle	-4.28228	19.44198	8.395032	30242	34	183	4	2.185792	1.080886	134|3778|5142|5290	ADORA1|KCNMA1|PDE4B|PIK3CA	-3.14168	0	78	0	0	-9.66495	66.10273224
1	-9.47753	GO:0007589	M1	1	0	GO Biological Processes	19	body fluid secretion	-9.47753	17.39546	12.48819	30242	95	183	10	5.464481	1.680144	100|134|595|1128|1131|1723|1956|4986|6531|7421	ADA|ADORA1|CCND1|CHRM1|CHRM3|DHODH|EGFR|OPRK1|SLC6A3|VDR	-7.833	0	79	1	1	-9.47753	41.31420765
1	-6.83474	GO:0032941	M1	1	0	GO Biological Processes	19	secretion by tissue	-6.83474	24.78852	11.7467	30242	40	183	6	3.278689	1.316393	100|134|1128|1131|1956|4986	ADA|ADORA1|CHRM1|CHRM3|EGFR|OPRK1	-5.41254	0	79	0	0	-9.47753	41.31420765
1	-4.33663	GO:0046541	M1	1	0	GO Biological Processes	19	saliva secretion	-4.33663	41.31421	10.89848	30242	12	183	3	1.639344	0.938686	1128|1131|4986	CHRM1|CHRM3|OPRK1	-3.18987	0	79	0	0	-9.47753	41.31420765
1	-9.31744	GO:0045123	M1	1	0	GO Biological Processes	19	cellular extravasation	-9.31744	20.94805	13.13019	30242	71	183	9	4.918033	1.598524	1991|3383|3683|5293|5734|6347|6401|7124|729230	ELANE|ICAM1|ITGAL|PIK3CD|PTGER4|CCL2|SELE|TNF|CCR2	-7.68016	0	80	1	1	-9.31744	55.0856102
1	-6.22221	GO:0072676	M1	1	0	GO Biological Processes	19	lymphocyte migration	-6.22221	11.29961	8.709368	30242	117	183	8	4.371585	1.511428	351|3383|3683|5293|5979|6347|6793|729230	APP|ICAM1|ITGAL|PIK3CD|RET|CCL2|STK10|CCR2	-4.85359	0	80	0	0	-9.31744	55.0856102
1	-6.20876	GO:0072683	M1	1	0	GO Biological Processes	19	T cell extravasation	-6.20876	55.08561	14.62142	30242	12	183	4	2.185792	1.080886	3383|3683|6347|729230	ICAM1|ITGAL|CCL2|CCR2	-4.84272	0	80	0	0	-9.31744	55.0856102

1	-5.51711	GO:0072678	M1	1	0	GO Biological Processes	19	T cell migration	-5.51711	15.02335	8.898754	30242	66	183	6	3.278689	1.316393	351|3383|3683|5293|6347|729230	APP|ICAM1|ITGAL|PIK3CD|CCL2|CCR2	-4.216	0	80	0	0	-9.31744	55.0856102
1	-5.17445	GO:0071675	M1	1	0	GO Biological Processes	19	regulation of mononuclear cell migration	-5.17445	10.05911	7.594391	30242	115	183	7	3.825137	1.417845	351|1230|6347|6793|7124|59341|729230	APP|CCR1|CCL2|STK10|TNF|TRPV4|CCR2	-3.91998	0	80	0	0	-9.31744	55.0856102
1	-4.90168	GO:2000310	M1	1	0	GO Biological Processes	19	regulation of NMDA receptor activity	-4.90168	27.54281	10.14976	30242	24	183	4	2.185792	1.080886	351|4988|6347|729230	APP|OPRM1|CCL2|CCR2	-3.68342	0	80	0	0	-9.31744	55.0856102
1	-4.39116	GO:0099601	M1	1	0	GO Biological Processes	19	regulation of neurotransmitter receptor activity	-4.39116	13.11562	7.511037	30242	63	183	5	2.73224	1.205087	154|351|4988|6347|729230	ADRB2|APP|OPRM1|CCL2|CCR2	-3.23972	0	80	0	0	-9.31744	55.0856102
1	-3.28627	GO:2000401	M1	1	0	GO Biological Processes	19	regulation of lymphocyte migration	-3.28627	10.83651	6.000337	30242	61	183	4	2.185792	1.080886	351|6347|6793|729230	APP|CCL2|STK10|CCR2	-2.25445	0	80	0	0	-9.31744	55.0856102
1	-3.18136	GO:0071677	M1	1	0	GO Biological Processes	19	positive regulation of mononuclear cell migration	-3.18136	10.16965	5.774413	30242	65	183	4	2.185792	1.080886	351|1230|7124|729230	APP|CCR1|TNF|CCR2	-2.15944	0	80	0	0	-9.31744	55.0856102
1	-2.15937	GO:0048247	M1	1	0	GO Biological Processes	19	lymphocyte chemotaxis	-2.15937	7.746414	4.215549	30242	64	183	3	1.639344	0.938686	5293|6347|729230	PIK3CD|CCL2|CCR2	-1.23591	0	80	0	0	-9.31744	55.0856102
1	-9.25129	GO:0009743	M1	1	0	GO Biological Processes	19	response to carbohydrate	-9.25129	9.108644	10.12576	30242	254	183	14	7.650273	1.964856	150|836|1080|1728|3156|3383|4221|4986|5024|5290|5467|5743|10135|10203	ADRA2A|CASP3|CFTR|NQO1|HMGCR|ICAM1|MEN1|OPRK1|P2RX3|PIK3CA|PPARD|PTGS2|NAMPT|CALCRL	-7.61856	0	81	1	1	-9.25129	13.04659189
1	-8.11828	GO:0042593	M1	1	0	GO Biological Processes	19	glucose homeostasis	-8.11828	8.262842	9.17697	30242	260	183	13	7.103825	1.898976	150|1080|1268|3156|3383|3569|3643|4221|4986|5290|5467|5468|59341	ADRA2A|CFTR|CNR1|HMGCR|ICAM1|IL6|INSR|MEN1|OPRK1|PIK3CA|PPARD|PPARG|TRPV4	-6.57357	0	81	0	0	-9.25129	13.04659189
1	-8.09837	GO:0033500	M1	1	0	GO Biological Processes	19	carbohydrate homeostasis	-8.09837	8.231183	9.154675	30242	261	183	13	7.103825	1.898976	150|1080|1268|3156|3383|3569|3643|4221|4986|5290|5467|5468|59341	ADRA2A|CFTR|CNR1|HMGCR|ICAM1|IL6|INSR|MEN1|OPRK1|PIK3CA|PPARD|PPARG|TRPV4	-6.55463	0	81	0	0	-9.25129	13.04659189
1	-6.95113	GO:0009746	M1	1	0	GO Biological Processes	19	response to hexose	-6.95113	8.262842	8.435959	30242	220	183	11	6.010929	1.757049	150|836|1080|3156|3383|4221|4986|5290|5467|5743|10135	ADRA2A|CASP3|CFTR|HMGCR|ICAM1|MEN1|OPRK1|PIK3CA|PPARD|PTGS2|NAMPT	-5.51857	0	81	0	0	-9.25129	13.04659189
1	-6.83321	GO:0034284	M1	1	0	GO Biological Processes	19	response to monosaccharide	-6.83321	8.043474	8.292798	30242	226	183	11	6.010929	1.757049	150|836|1080|3156|3383|4221|4986|5290|5467|5743|10135	ADRA2A|CASP3|CFTR|HMGCR|ICAM1|MEN1|OPRK1|PIK3CA|PPARD|PTGS2|NAMPT	-5.41174	0	81	0	0	-9.25129	13.04659189
1	-6.12181	GO:0071322	M1	1	0	GO Biological Processes	19	cellular response to carbohydrate stimulus	-6.12181	9.068972	8.084479	30242	164	183	9	4.918033	1.598524	150|1080|3156|3383|4221|4986|5290|5467|10203	ADRA2A|CFTR|HMGCR|ICAM1|MEN1|OPRK1|PIK3CA|PPARD|CALCRL	-4.76405	0	81	0	0	-9.25129	13.04659189
1	-5.36609	GO:0071333	M1	1	0	GO Biological Processes	19	cellular response to glucose stimulus	-5.36609	8.697728	7.423515	30242	152	183	8	4.371585	1.511428	150|1080|3156|3383|4221|4986|5290|5467	ADRA2A|CFTR|HMGCR|ICAM1|MEN1|OPRK1|PIK3CA|PPARD	-4.08918	0	81	0	0	-9.25129	13.04659189
1	-5.32405	GO:0071331	M1	1	0	GO Biological Processes	19	cellular response to hexose stimulus	-5.32405	8.58477	7.362791	30242	154	183	8	4.371585	1.511428	150|1080|3156|3383|4221|4986|5290|5467	ADRA2A|CFTR|HMGCR|ICAM1|MEN1|OPRK1|PIK3CA|PPARD	-4.05287	0	81	0	0	-9.25129	13.04659189

1	-5.30327	GO:0071326	M1	1	0	GO Biological Processes	19	cellular response to monosaccharide stimulus	-5.30327	8.529385	7.332841	30242	155	183	8	4.371585	1.511428	150|1080|3156|3383|4221|4986|5290|5467	ADRA2A|CFTR|HMGCR|ICAM1|MEN1|OPRK1|PIK3CA|PPARD	-4.03311	0	81	0	0	-9.25129	13.04659189
1	-5.18734	GO:0009749	M1	1	0	GO Biological Processes	19	response to glucose	-5.18734	6.982683	6.836782	30242	213	183	9	4.918033	1.598524	150|836|1080|3156|3383|4221|4986|5290|5467	ADRA2A|CASP3|CFTR|HMGCR|ICAM1|MEN1|OPRK1|PIK3CA|PPARD	-3.93187	0	81	0	0	-9.25129	13.04659189
1	-4.9532	GO:0001678	M1	1	0	GO Biological Processes	19	cellular glucose homeostasis	-4.9532	7.641934	6.835881	30242	173	183	8	4.371585	1.511428	150|1080|3156|3383|4221|4986|5290|5467	ADRA2A|CFTR|HMGCR|ICAM1|MEN1|OPRK1|PIK3CA|PPARD	-3.72383	0	81	0	0	-9.25129	13.04659189
1	-4.35796	GO:0046888	M1	1	0	GO Biological Processes	19	negative regulation of hormone secretion	-4.35796	12.91069	7.442486	30242	64	183	5	2.73224	1.205087	134|150|1813|3156|4986	ADORA1|ADRA2A|DRD2|HMGCR|OPRK1	-3.21003	0	81	0	0	-9.25129	13.04659189
1	-3.20691	GO:0061178	M1	1	0	GO Biological Processes	19	regulation of insulin secretion involved in cellular response to glucose stimulus	-3.20691	10.32855	5.829017	30242	64	183	4	2.185792	1.080886	150|1080|3156|5467	ADRA2A|CFTR|HMGCR|PPARD	-2.18207	0	81	0	0	-9.25129	13.04659189
1	-2.99148	GO:0035773	M1	1	0	GO Biological Processes	19	insulin secretion involved in cellular response to glucose stimulus	-2.99148	9.055169	5.376405	30242	73	183	4	2.185792	1.080886	150|1080|3156|5467	ADRA2A|CFTR|HMGCR|PPARD	-1.98834	0	81	0	0	-9.25129	13.04659189
1	-2.80314	GO:0046676	M1	1	0	GO Biological Processes	19	negative regulation of insulin secretion	-2.80314	13.04659	5.797758	30242	38	183	3	1.639344	0.938686	150|1813|3156	ADRA2A|DRD2|HMGCR	-1.82068	0	81	0	0	-9.25129	13.04659189
1	-2.64755	GO:0090278	M1	1	0	GO Biological Processes	19	negative regulation of peptide hormone secretion	-2.64755	11.52955	5.391182	30242	43	183	3	1.639344	0.938686	150|1813|3156	ADRA2A|DRD2|HMGCR	-1.68278	0	81	0	0	-9.25129	13.04659189
1	-2.6188	GO:0002792	M1	1	0	GO Biological Processes	19	negative regulation of peptide secretion	-2.6188	11.26751	5.317883	30242	44	183	3	1.639344	0.938686	150|1813|3156	ADRA2A|DRD2|HMGCR	-1.65683	0	81	0	0	-9.25129	13.04659189
1	-9.19381	GO:0060322	M1	1	0	GO Biological Processes	19	head development	-9.19381	4.775009	8.42166	30242	796	183	23	12.56831	2.450458	351|596|836|1453|1812|1813|1909|2263|2558|2902|2903|2904|3362|4129|4221|4313|4915|5591|6331|6513|6531|6532|7157	APP|BCL2|CASP3|CSNK1D|DRD1|DRD2|EDNRA|FGFR2|GABRA5|GRIN1|GRIN2A|GRIN2B|HTR6|MAOB|MEN1|MMP2|NTRK2|PRKDC|SCN5A|SLC2A1|SLC6A3|SLC6A4|TP53	-7.56367	0	82	1	1	-9.19381	8.5477671
1	-8.15695	GO:0007420	M1	1	0	GO Biological Processes	19	brain development	-8.15695	4.608756	7.824586	30242	753	183	21	11.47541	2.356083	351|596|836|1453|1812|1813|2263|2558|2902|2903|2904|3362|4129|4221|4915|5591|6331|6513|6531|6532|7157	APP|BCL2|CASP3|CSNK1D|DRD1|DRD2|FGFR2|GABRA5|GRIN1|GRIN2A|GRIN2B|HTR6|MAOB|MEN1|NTRK2|PRKDC|SCN5A|SLC2A1|SLC6A3|SLC6A4|TP53	-6.60442	0	82	0	0	-9.19381	8.5477671
1	-3.93871	GO:0030900	M1	1	0	GO Biological Processes	19	forebrain development	-3.93871	4.348864	5.125854	30242	380	183	10	5.464481	1.680144	351|836|1812|1813|2263|3362|4915|6331|6513|6531	APP|CASP3|DRD1|DRD2|FGFR2|HTR6|NTRK2|SCN5A|SLC2A1|SLC6A3	-2.83536	0	82	0	0	-9.19381	8.5477671
1	-3.06595	GO:0021537	M1	1	0	GO Biological Processes	19	telencephalon development	-3.06595	4.627191	4.49339	30242	250	183	7	3.825137	1.417845	836|1812|1813|3362|4915|6331|6513	CASP3|DRD1|DRD2|HTR6|NTRK2|SCN5A|SLC2A1	-2.05579	0	82	0	0	-9.19381	8.5477671

<table>
<tr><td>1</td><td>-2.82347</td><td>GO:0021954</td><td>M1</td><td>1</td><td>0</td><td>GO Biological Processes</td><td>19</td><td>central nervous system neuron development</td><td>-2.82347</td><td>8.160831</td><td>5.035231</td><td>30242</td><td>81</td><td>183</td><td>4</td><td>2.185792</td><td>1.080886</td><td>1812|1813|2263|4915</td><td>DRD1|DRD2|FGFR2|NTRK2</td><td>-1.83834</td><td>0</td><td>82</td><td>0</td><td>0</td><td>-9.19381</td><td>8.5477671</td></tr>
<tr><td>1</td><td>-2.27832</td><td>GO:0022029</td><td>M1</td><td>1</td><td>0</td><td>GO Biological Processes</td><td>19</td><td>telencephalon cell migration</td><td>-2.27832</td><td>8.547767</td><td>4.489319</td><td>30242</td><td>58</td><td>183</td><td>3</td><td>1.639344</td><td>0.938686</td><td>1812|1813|3362</td><td>DRD1|DRD2|HTR6</td><td>-1.34621</td><td>0</td><td>82</td><td>0</td><td>0</td><td>-9.19381</td><td>8.5477671</td></tr>
<tr><td>1</td><td>-2.21721</td><td>GO:0021885</td><td>M1</td><td>1</td><td>0</td><td>GO Biological Processes</td><td>19</td><td>forebrain cell migration</td><td>-2.21721</td><td>8.127385</td><td>4.347751</td><td>30242</td><td>61</td><td>183</td><td>3</td><td>1.639344</td><td>0.938686</td><td>1812|1813|3362</td><td>DRD1|DRD2|HTR6</td><td>-1.29004</td><td>0</td><td>82</td><td>0</td><td>0</td><td>-9.19381</td><td>8.5477671</td></tr>
<tr><td>1</td><td>-8.89542</td><td>GO:0062013</td><td>M1</td><td>1</td><td>0</td><td>GO Biological Processes</td><td>19</td><td>positive regulation of small molecule metabolic process</td><td>-8.89542</td><td>12.71206</td><td>10.95372</td><td>30242</td><td>143</td><td>183</td><td>11</td><td>6.010929</td><td>1.757049</td><td>351|3356|3643|4842|4843|4846|5465|5467|5468|5743|7124</td><td>APP|HTR2A|INSR|NOS1|NOS2|NOS3|PPARA|PPARD|PPARG|PTGS2|TNF</td><td>-7.28715</td><td>0</td><td>83</td><td>1</td><td>1</td><td>-8.89542</td><td>26.90227475</td></tr>
<tr><td>1</td><td>-8.14883</td><td>GO:0045981</td><td>M1</td><td>1</td><td>0</td><td>GO Biological Processes</td><td>19</td><td>positive regulation of nucleotide metabolic process</td><td>-8.14883</td><td>26.90227</td><td>13.2621</td><td>30242</td><td>43</td><td>183</td><td>7</td><td>3.825137</td><td>1.417845</td><td>351|3356|3643|4842|4843|4846|5465</td><td>APP|HTR2A|INSR|NOS1|NOS2|NOS3|PPARA</td><td>-6.59827</td><td>0</td><td>83</td><td>0</td><td>0</td><td>-8.89542</td><td>26.90227475</td></tr>
<tr><td>1</td><td>-8.14883</td><td>GO:1900544</td><td>M1</td><td>1</td><td>0</td><td>GO Biological Processes</td><td>19</td><td>positive regulation of purine nucleotide metabolic process</td><td>-8.14883</td><td>26.90227</td><td>13.2621</td><td>30242</td><td>43</td><td>183</td><td>7</td><td>3.825137</td><td>1.417845</td><td>351|3356|3643|4842|4843|4846|5465</td><td>APP|HTR2A|INSR|NOS1|NOS2|NOS3|PPARA</td><td>-6.59827</td><td>0</td><td>83</td><td>0</td><td>0</td><td>-8.89542</td><td>26.90227475</td></tr>
<tr><td>1</td><td>-7.33254</td><td>GO:0090407</td><td>M1</td><td>1</td><td>0</td><td>GO Biological Processes</td><td>19</td><td>organophosphate biosynthetic process</td><td>-7.33254</td><td>5.117242</td><td>7.596297</td><td>30242</td><td>549</td><td>183</td><td>17</td><td>9.289617</td><td>2.145862</td><td>100|472|1723|3356|3357|3358|4842|4843|4846|5290|5291|5293|5465|5467|7040|7298|10135</td><td>ADA|ATM|DHODH|HTR2A|HTR2B|HTR2C|NOS1|NOS2|NOS3|PIK3CA|PIK3CB|PIK3CD|PPARA|PPARD|TGFB1|TYMS|NAMPT</td><td>-5.86491</td><td>0</td><td>83</td><td>0</td><td>0</td><td>-8.89542</td><td>26.90227475</td></tr>
<tr><td>1</td><td>-7.24309</td><td>GO:0009117</td><td>M1</td><td>1</td><td>0</td><td>GO Biological Processes</td><td>19</td><td>nucleotide metabolic process</td><td>-7.24309</td><td>5.396141</td><td>7.655049</td><td>30242</td><td>490</td><td>183</td><td>16</td><td>8.743169</td><td>2.088053</td><td>100|351|1723|3156|3356|3643|4842|4843|4846|5138|5142|5465|7040|7298|8654|10135</td><td>ADA|APP|DHODH|HMGCR|HTR2A|INSR|NOS1|NOS2|NOS3|PDE2A|PDE4B|PPARA|TGFB1|TYMS|PDE5A|NAMPT</td><td>-5.78747</td><td>0</td><td>83</td><td>0</td><td>0</td><td>-8.89542</td><td>26.90227475</td></tr>
<tr><td>1</td><td>-7.14702</td><td>GO:0006753</td><td>M1</td><td>1</td><td>0</td><td>GO Biological Processes</td><td>19</td><td>nucleoside phosphate metabolic process</td><td>-7.14702</td><td>5.309456</td><td>7.566131</td><td>30242</td><td>498</td><td>183</td><td>16</td><td>8.743169</td><td>2.088053</td><td>100|351|1723|3156|3356|3643|4842|4843|4846|5138|5142|5465|7040|7298|8654|10135</td><td>ADA|APP|DHODH|HMGCR|HTR2A|INSR|NOS1|NOS2|NOS3|PDE2A|PDE4B|PPARA|TGFB1|TYMS|PDE5A|NAMPT</td><td>-5.69611</td><td>0</td><td>83</td><td>0</td><td>0</td><td>-8.89542</td><td>26.90227475</td></tr>
<tr><td>1</td><td>-6.82791</td><td>GO:0062012</td><td>M1</td><td>1</td><td>0</td><td>GO Biological Processes</td><td>19</td><td>regulation of small molecule metabolic process</td><td>-6.82791</td><td>6.412952</td><td>7.773319</td><td>30242</td><td>335</td><td>183</td><td>13</td><td>7.103825</td><td>1.898976</td><td>351|1268|3356|3643|4842|4843|4846|5465|5467|5468|5743|7124|7157</td><td>APP|CNR1|HTR2A|INSR|NOS1|NOS2|NOS3|PPARA|PPARD|PPARG|PTGS2|TNF|TP53</td><td>-5.40717</td><td>0</td><td>83</td><td>0</td><td>0</td><td>-8.89542</td><td>26.90227475</td></tr>
<tr><td>1</td><td>-6.35906</td><td>GO:0055086</td><td>M1</td><td>1</td><td>0</td><td>GO Biological Processes</td><td>19</td><td>nucleobase-containing small molecule metabolic process</td><td>-6.35906</td><td>4.638788</td><td>6.843167</td><td>30242</td><td>570</td><td>183</td><td>16</td><td>8.743169</td><td>2.088053</td><td>100|351|1723|3156|3356|3643|4842|4843|4846|5138|5142|5465|7040|7298|8654|10135</td><td>ADA|APP|DHODH|HMGCR|HTR2A|INSR|NOS1|NOS2|NOS3|PDE2A|PDE4B|PPARA|TGFB1|TYMS|PDE5A|NAMPT</td><td>-4.98131</td><td>0</td><td>83</td><td>0</td><td>0</td><td>-8.89542</td><td>26.90227475</td></tr>
<tr><td>1</td><td>-6.09163</td><td>GO:1900542</td><td>M1</td><td>1</td><td>0</td><td>GO Biological Processes</td><td>19</td><td>regulation of purine nucleotide metabolic process</td><td>-6.09163</td><td>13.7714</td><td>9.145624</td><td>30242</td><td>84</td><td>183</td><td>7</td><td>3.825137</td><td>1.417845</td><td>351|3356|3643|4842|4843|4846|5465</td><td>APP|HTR2A|INSR|NOS1|NOS2|NOS3|PPARA</td><td>-4.73638</td><td>0</td><td>83</td><td>0</td><td>0</td><td>-8.89542</td><td>26.90227475</td></tr>
<tr><td>1</td><td>-6.02185</td><td>GO:0006140</td><td>M1</td><td>1</td><td>0</td><td>GO Biological Processes</td><td>19</td><td>regulation of nucleotide metabolic process</td><td>-6.02185</td><td>13.45114</td><td>9.022102</td><td>30242</td><td>86</td><td>183</td><td>7</td><td>3.825137</td><td>1.417845</td><td>351|3356|3643|4842|4843|4846|5465</td><td>APP|HTR2A|INSR|NOS1|NOS2|NOS3|PPARA</td><td>-4.67282</td><td>0</td><td>83</td><td>0</td><td>0</td><td>-8.89542</td><td>26.90227475</td></tr>
<tr><td>1</td><td>-5.99404</td><td>GO:0006163</td><td>M1</td><td>1</td><td>0</td><td>GO Biological</td><td>19</td><td>purine nucleotide</td><td>-5.99404</td><td>5.411433</td><td>6.903598</td><td>30242</td><td>397</td><td>183</td><td>13</td><td>7.103825</td><td>1.898976</td><td>100|351|3156|3356|364</td><td>ADA|APP|HMGCR|H</td><td>-4.64624</td><td>0</td><td>83</td><td>0</td><td>0</td><td>-8.89542</td><td>26.90227475</td></tr>
</table>

						Processes		metabolic process										3|4842|4843|4846|5138|5142|5465|7040|8654	TR2A|INSR|NOS1|NOS2|NOS3|PDE2A|PDE4B|PPARA|TGFB1|PDE5A							
1	-5.8743	GO:0043467	M1	1	0	GO Biological Processes	19	regulation of generation of precursor metabolites and energy	-5.8743	10.16965	8.175062	30242	130	183	8	4.371585	1.511428	351|3356|3643|4843|5290|5465|7157|59341	APP|HTR2A|INSR|NOS2|PIK3CA|PPARA|TP53|TRPV4	-4.54164	0	83	0	0	-8.89542	26.90227475
1	-5.76932	GO:0072521	M1	1	0	GO Biological Processes	19	purine-containing compound metabolic process	-5.76932	5.164276	6.67306	30242	416	183	13	7.103825	1.898976	100|351|3156|3356|3643|4842|4843|4846|5138|5142|5465|7040|8654	ADA|APP|HMGCR|HTR2A|INSR|NOS1|NOS2|NOS3|PDE2A|PDE4B|PPARA|TGFB1|PDE5A	-4.44257	0	83	0	0	-8.89542	26.90227475
1	-5.71625	GO:0006091	M1	1	0	GO Biological Processes	19	generation of precursor metabolites and energy	-5.71625	4.721624	6.480504	30242	490	183	14	7.650273	1.964856	217|351|1544|1588|2936|3356|3643|4129|4843|5290|5465|5467|7157|59341	ALDH2|APP|CYP1A2|CYP19A1|GSR|HTR2A|INSR|MAOB|NOS2|PIK3CA|PPARA|PPARD|TP53|TRPV4	-4.39416	0	83	0	0	-8.89542	26.90227475
1	-4.97258	GO:0009896	M1	1	0	GO Biological Processes	19	positive regulation of catabolic process	-4.97258	4.357685	5.86494	30242	493	183	13	7.103825	1.898976	134|150|154|351|1453|3356|3569|3643|5291|5465|7124|9475|29110	ADORA1|ADRA2A|ADRB2|APP|CSNK1D|HTR2A|IL6|INSR|PIK3CB|PPARA|TNF|ROCK2|TBK1	-3.74133	0	83	0	0	-8.89542	26.90227475
1	-4.89258	GO:0031331	M1	1	0	GO Biological Processes	19	positive regulation of cellular catabolic process	-4.89258	4.633369	5.906873	30242	428	183	12	6.557377	1.829835	150|154|351|1453|3356|3569|3643|5291|5465|7124|9475|29110	ADRA2A|ADRB2|APP|CSNK1D|HTR2A|IL6|INSR|PIK3CB|PPARA|TNF|ROCK2|TBK1	-3.67478	0	83	0	0	-8.89542	26.90227475
1	-4.64115	GO:0043470	M1	1	0	GO Biological Processes	19	regulation of carbohydrate catabolic process	-4.64115	14.75507	8.038787	30242	56	183	5	2.73224	1.205087	351|3356|3643|5465|7157	APP|HTR2A|INSR|PPARA|TP53	-3.45334	0	83	0	0	-8.89542	26.90227475
1	-4.59319	GO:0009259	M1	1	0	GO Biological Processes	19	ribonucleotide metabolic process	-4.59319	4.709392	5.722909	30242	386	183	11	6.010929	1.757049	100|351|1723|3156|3356|3643|5138|5142|5465|7040|8654	ADA|APP|DHODH|HMGCR|HTR2A|INSR|PDE2A|PDE4B|PPARA|TGFB1|PDE5A	-3.41591	0	83	0	0	-8.89542	26.90227475
1	-4.57899	GO:0009165	M1	1	0	GO Biological Processes	19	nucleotide biosynthetic process	-4.57899	5.855557	6.063432	30242	254	183	9	4.918033	1.598524	100|1723|4842|4843|4846|5465|7040|7298|10135	ADA|DHODH|NOS1|NOS2|NOS3|PPARA|TGFB1|TYMS|NAMPT	-3.40254	0	83	0	0	-8.89542	26.90227475
1	-4.55232	GO:1901293	M1	1	0	GO Biological Processes	19	nucleoside phosphate biosynthetic process	-4.55232	5.80981	6.030107	30242	256	183	9	4.918033	1.598524	100|1723|4842|4843|4846|5465|7040|7298|10135	ADA|DHODH|NOS1|NOS2|NOS3|PPARA|TGFB1|TYMS|NAMPT	-3.38	0	83	0	0	-8.89542	26.90227475
1	-4.48207	GO:0019693	M1	1	0	GO Biological Processes	19	ribose phosphate metabolic process	-4.48207	4.578905	5.600747	30242	397	183	11	6.010929	1.757049	100|351|1723|3156|3356|3643|5138|5142|5465|7040|8654	ADA|APP|DHODH|HMGCR|HTR2A|INSR|PDE2A|PDE4B|PPARA|TGFB1|PDE5A	-3.32152	0	83	0	0	-8.89542	26.90227475
1	-4.08863	GO:1903580	M1	1	0	GO Biological Processes	19	positive regulation of ATP metabolic process	-4.08863	17.39546	7.890771	30242	38	183	4	2.185792	1.080886	351|3356|3643|5465	APP|HTR2A|INSR|PPARA	-2.96862	0	83	0	0	-8.89542	26.90227475
1	-4.04312	GO:0009150	M1	1	0	GO Biological Processes	19	purine ribonucleotide metabolic process	-4.04312	4.478505	5.245691	30242	369	183	10	5.464481	1.680144	100|351|3156|3356|3643|5138|5142|5465|7040|8654	ADA|APP|HMGCR|HTR2A|INSR|PDE2A|PDE4B|PPARA|TGFB1|PDE5A	-2.92668	0	83	0	0	-8.89542	26.90227475
1	-3.79794	GO:0006110	M1	1	0	GO Biological Processes	19	regulation of glycolytic process	-3.79794	14.6895	7.170479	30242	45	183	4	2.185792	1.080886	351|3356|3643|5465	APP|HTR2A|INSR|PPARA	-2.70849	0	83	0	0	-8.89542	26.90227475

1	-3.72127	GO:1903578	M1	1	0	GO Biological Processes	19	regulation of ATP metabolic process	-3.72127	9.497519	6.193105	30242	87	183	5	2.73224	1.205087	351|3356|3643|5465|7157	APP|HTR2A|INSR|PPARA|TP53	-2.64322	0	83	0	0	-8.89542	26.90227475
1	-3.70629	GO:0045821	M1	1	0	GO Biological Processes	19	positive regulation of glycolytic process	-3.70629	26.09318	8.536899	30242	19	183	3	1.639344	0.938686	351|3356|3643	APP|HTR2A|INSR	-2.63055	0	83	0	0	-8.89542	26.90227475
1	-3.10958	GO:0006109	M1	1	0	GO Biological Processes	19	regulation of carbohydrate metabolic process	-3.10958	5.570455	4.771802	30242	178	183	6	3.278689	1.316393	351|3356|3643|5465|7040|7157	APP|HTR2A|INSR|PPARA|TGFB1|TP53	-2.09344	0	83	0	0	-8.89542	26.90227475
1	-2.95132	GO:0006164	M1	1	0	GO Biological Processes	19	purine nucleotide biosynthetic process	-2.95132	5.191314	4.533919	30242	191	183	6	3.278689	1.316393	100|4842|4843|4846|5465|7040	ADA|NOS1|NOS2|NOS3|PPARA|TGFB1	-1.95179	0	83	0	0	-8.89542	26.90227475
1	-2.94761	GO:0045913	M1	1	0	GO Biological Processes	19	positive regulation of carbohydrate metabolic process	-2.94761	8.813698	5.28637	30242	75	183	4	2.185792	1.080886	351|3356|3643|5465	APP|HTR2A|INSR|PPARA	-1.94919	0	83	0	0	-8.89542	26.90227475
1	-2.84911	GO:0072522	M1	1	0	GO Biological Processes	19	purine-containing compound biosynthetic process	-2.84911	4.957705	4.381576	30242	200	183	6	3.278689	1.316393	100|4842|4843|4846|5465|7040	ADA|NOS1|NOS2|NOS3|PPARA|TGFB1	-1.8605	0	83	0	0	-8.89542	26.90227475
1	-2.82347	GO:0006096	M1	1	0	GO Biological Processes	19	glycolytic process	-2.82347	8.160831	5.035231	30242	81	183	4	2.185792	1.080886	351|3356|3643|5465	APP|HTR2A|INSR|PPARA	-1.83834	0	83	0	0	-8.89542	26.90227475
1	-2.80379	GO:0006757	M1	1	0	GO Biological Processes	19	ATP generation from ADP	-2.80379	8.061309	4.995889	30242	82	183	4	2.185792	1.080886	351|3356|3643|5465	APP|HTR2A|INSR|PPARA	-1.82068	0	83	0	0	-8.89542	26.90227475
1	-2.65542	GO:0046031	M1	1	0	GO Biological Processes	19	ADP metabolic process	-2.65542	7.344748	4.703422	30242	90	183	4	2.185792	1.080886	351|3356|3643|5465	APP|HTR2A|INSR|PPARA	-1.68835	0	83	0	0	-8.89542	26.90227475
1	-2.60002	GO:0016052	M1	1	0	GO Biological Processes	19	carbohydrate catabolic process	-2.60002	5.365482	4.23772	30242	154	183	5	2.73224	1.205087	351|3356|3643|5465|7157	APP|HTR2A|INSR|PPARA|TP53	-1.63931	0	83	0	0	-8.89542	26.90227475
1	-2.5054	GO:0006165	M1	1	0	GO Biological Processes	19	nucleoside diphosphate phosphorylation	-2.5054	6.677044	4.414515	30242	99	183	4	2.185792	1.080886	351|3356|3643|5465	APP|HTR2A|INSR|PPARA	-1.55246	0	83	0	0	-8.89542	26.90227475
1	-2.47417	GO:0046939	M1	1	0	GO Biological Processes	19	nucleotide phosphorylation	-2.47417	6.544825	4.355179	30242	101	183	4	2.185792	1.080886	351|3356|3643|5465	APP|HTR2A|INSR|PPARA	-1.52493	0	83	0	0	-8.89542	26.90227475
1	-2.44365	GO:0009135	M1	1	0	GO Biological Processes	19	purine nucleoside diphosphate metabolic process	-2.44365	6.417741	4.29743	30242	103	183	4	2.185792	1.080886	351|3356|3643|5465	APP|HTR2A|INSR|PPARA	-1.49711	0	83	0	0	-8.89542	26.90227475
1	-2.44365	GO:0009179	M1	1	0	GO Biological Processes	19	purine ribonucleoside diphosphate metabolic process	-2.44365	6.417741	4.29743	30242	103	183	4	2.185792	1.080886	351|3356|3643|5465	APP|HTR2A|INSR|PPARA	-1.49711	0	83	0	0	-8.89542	26.90227475
1	-2.39911	GO:0006090	M1	1	0	GO Biological Processes	19	pyruvate metabolic process	-2.39911	6.236107	4.213616	30242	106	183	4	2.185792	1.080886	351|3356|3643|5465	APP|HTR2A|INSR|PPARA	-1.45671	0	83	0	0	-8.89542	26.90227475
1	-2.39911	GO:0009185	M1	1	0	GO Biological Processes	19	ribonucleoside diphosphate metabolic process	-2.39911	6.236107	4.213616	30242	106	183	4	2.185792	1.080886	351|3356|3643|5465	APP|HTR2A|INSR|PPARA	-1.45671	0	83	0	0	-8.89542	26.90227475
1	-2.31425	GO:0009141	M1	1	0	GO Biological Processes	19	nucleoside triphosphate metabolic process	-2.31425	5.90203	4.055288	30242	112	183	4	2.185792	1.080886	100|5465|7040|7298	ADA|PPARA|TGFB1|TYMS	-1.37977	0	83	0	0	-8.89542	26.90227475
1	-2.15936	GO:0009132	M1	1	0	GO Biological Processes	19	nucleoside diphosphate metabolic process	-2.15936	5.330866	3.770586	30242	124	183	4	2.185792	1.080886	351|3356|3643|5465	APP|HTR2A|INSR|PPARA	-1.23591	0	83	0	0	-8.89542	26.90227475
1	-2.1559	GO:0046034	M1	1	0	GO Biological Processes	19	ATP metabolic process	-2.1559	3.57957	3.365243	30242	277	183	6	3.278689	1.316393	351|3356|3643|5465|7040|7157	APP|HTR2A|INSR|PPARA|TGFB1|TP53	-1.23268	0	83	0	0	-8.89542	26.90227475
1	-8.83196	GO:0061614	M1	1	0	GO Biological Processes	19	pri-miRNA transcription by RNA polymerase II	-8.83196	24.03736	13.3426	30242	55	183	8	4.371585	1.511428	2908|5465|5467|5468|5970|7040|7124|7157	NR3C1|PPARA|PPARD|PPARG|RELA|TGFB1|TNF|TP53	-7.22816	0	84	1	1	-8.83196	24.03735718
1	-8.83196	GO:1902893	M1	1	0	GO Biological Processes	19	regulation of pri-miRNA transcription by RNA polymerase II	-8.83196	24.03736	13.3426	30242	55	183	8	4.371585	1.511428	2908|5465|5467|5468|5970|7040|7124|7157	NR3C1|PPARA|PPARD|PPARG|RELA|TGFB1|TNF|TP53	-7.22816	0	84	0	0	-8.83196	24.03735718

<table>
<tr><td>1</td><td>-6.70343</td><td>GO:1902895</td><td>M1</td><td>1</td><td>0</td><td>GO Biological Processes</td><td>19</td><td>positive regulation of pri-miRNA transcription by RNA polymerase II</td><td>-6.70343</td><td>23.60812</td><td>11.43989</td><td>30242</td><td>42</td><td>183</td><td>6</td><td>3.278689</td><td>1.316393</td><td>2908|5468|5970|7040|7124|7157</td><td>NR3C1|PPARG|RELA|TGFB1|TNF|TP53</td><td>-5.29491</td><td>0</td><td>84</td><td>0</td><td>0</td><td>-8.83196</td><td>24.03735718</td></tr>
<tr><td>1</td><td>-6.22649</td><td>GO:0071559</td><td>M1</td><td>1</td><td>0</td><td>GO Biological Processes</td><td>19</td><td>response to transforming growth factor beta</td><td>-6.22649</td><td>6.991635</td><td>7.570736</td><td>30242</td><td>260</td><td>183</td><td>11</td><td>6.010929</td><td>1.757049</td><td>2263|2908|3066|4221|5138|5465|5468|7040|7046|7157|9475</td><td>FGFR2|NR3C1|HDAC2|MEN1|PDE2A|PPARA|PPARG|TGFB1|TGFBR1|TP53|ROCK2</td><td>-4.85723</td><td>0</td><td>84</td><td>0</td><td>0</td><td>-8.83196</td><td>24.03735718</td></tr>
<tr><td>1</td><td>-5.43058</td><td>GO:0071560</td><td>M1</td><td>1</td><td>0</td><td>GO Biological Processes</td><td>19</td><td>cellular response to transforming growth factor beta stimulus</td><td>-5.43058</td><td>6.506174</td><td>6.875898</td><td>30242</td><td>254</td><td>183</td><td>10</td><td>5.464481</td><td>1.680144</td><td>2263|2908|3066|4221|5138|5465|5468|7040|7046|7157</td><td>FGFR2|NR3C1|HDAC2|MEN1|PDE2A|PPARA|PPARG|TGFB1|TGFBR1|TP53</td><td>-4.14034</td><td>0</td><td>84</td><td>0</td><td>0</td><td>-8.83196</td><td>24.03735718</td></tr>
<tr><td>1</td><td>-2.93777</td><td>GO:0017015</td><td>M1</td><td>1</td><td>0</td><td>GO Biological Processes</td><td>19</td><td>regulation of transforming growth factor beta receptor signaling pathway</td><td>-2.93777</td><td>6.405304</td><td>4.800365</td><td>30242</td><td>129</td><td>183</td><td>5</td><td>2.73224</td><td>1.205087</td><td>4221|5465|5468|7040|7157</td><td>MEN1|PPARA|PPARG|TGFB1|TP53</td><td>-1.94099</td><td>0</td><td>84</td><td>0</td><td>0</td><td>-8.83196</td><td>24.03735718</td></tr>
<tr><td>1</td><td>-2.89337</td><td>GO:1903844</td><td>M1</td><td>1</td><td>0</td><td>GO Biological Processes</td><td>19</td><td>regulation of cellular response to transforming growth factor beta stimulus</td><td>-2.89337</td><td>6.259728</td><td>4.72532</td><td>30242</td><td>132</td><td>183</td><td>5</td><td>2.73224</td><td>1.205087</td><td>4221|5465|5468|7040|7157</td><td>MEN1|PPARA|PPARG|TGFB1|TP53</td><td>-1.90097</td><td>0</td><td>84</td><td>0</td><td>0</td><td>-8.83196</td><td>24.03735718</td></tr>
<tr><td>1</td><td>-2.82715</td><td>GO:0007179</td><td>M1</td><td>1</td><td>0</td><td>GO Biological Processes</td><td>19</td><td>transforming growth factor beta receptor signaling pathway</td><td>-2.82715</td><td>4.908619</td><td>4.34896</td><td>30242</td><td>202</td><td>183</td><td>6</td><td>3.278689</td><td>1.316393</td><td>4221|5465|5468|7040|7046|7157</td><td>MEN1|PPARA|PPARG|TGFB1|TGFBR1|TP53</td><td>-1.84068</td><td>0</td><td>84</td><td>0</td><td>0</td><td>-8.83196</td><td>24.03735718</td></tr>
<tr><td>1</td><td>-2.72684</td><td>GO:0010821</td><td>M1</td><td>1</td><td>0</td><td>GO Biological Processes</td><td>19</td><td>regulation of mitochondrion organization</td><td>-2.72684</td><td>5.738084</td><td>4.446841</td><td>30242</td><td>144</td><td>183</td><td>5</td><td>2.73224</td><td>1.205087</td><td>1723|4318|5138|5468|7157</td><td>DHODH|MMP9|PDE2A|PPARG|TP53</td><td>-1.75305</td><td>0</td><td>84</td><td>0</td><td>0</td><td>-8.83196</td><td>24.03735718</td></tr>
<tr><td>1</td><td>-2.51055</td><td>GO:0045912</td><td>M1</td><td>1</td><td>0</td><td>GO Biological Processes</td><td>19</td><td>negative regulation of carbohydrate metabolic process</td><td>-2.51055</td><td>10.32855</td><td>5.046739</td><td>30242</td><td>48</td><td>183</td><td>3</td><td>1.639344</td><td>0.938686</td><td>5465|7040|7157</td><td>PPARA|TGFB1|TP53</td><td>-1.55686</td><td>0</td><td>84</td><td>0</td><td>0</td><td>-8.83196</td><td>24.03735718</td></tr>
<tr><td>1</td><td>-2.30231</td><td>GO:0090092</td><td>M1</td><td>1</td><td>0</td><td>GO Biological Processes</td><td>19</td><td>regulation of transmembrane receptor protein serine/threonine kinase signaling pathway</td><td>-2.30231</td><td>3.843182</td><td>3.578542</td><td>30242</td><td>258</td><td>183</td><td>6</td><td>3.278689</td><td>1.316393</td><td>4221|5465|5468|7040|7046|7157</td><td>MEN1|PPARA|PPARG|TGFB1|TGFBR1|TP53</td><td>-1.3683</td><td>0</td><td>84</td><td>0</td><td>0</td><td>-8.83196</td><td>24.03735718</td></tr>
<tr><td>1</td><td>-8.69967</td><td>GO:0051899</td><td>M1</td><td>1</td><td>0</td><td>GO Biological Processes</td><td>19</td><td>membrane depolarization</td><td>-8.69967</td><td>17.91942</td><td>12.04349</td><td>30242</td><td>83</td><td>183</td><td>9</td><td>4.918033</td><td>1.598524</td><td>135|596|775|834|3757|6326|6331|6335|6336</td><td>ADORA2A|BCL2|CACNA1C|CASP1|KCNH2|SCN2A|SCN5A|SCN9A|SCN10A</td><td>-7.09918</td><td>0</td><td>85</td><td>1</td><td>1</td><td>-8.69967</td><td>45.07004471</td></tr>
<tr><td>1</td><td>-8.17344</td><td>GO:0006942</td><td>M1</td><td>1</td><td>0</td><td>GO Biological Processes</td><td>19</td><td>regulation of striated muscle contraction</td><td>-8.17344</td><td>15.65591</td><td>11.16321</td><td>30242</td><td>95</td><td>183</td><td>9</td><td>4.918033</td><td>1.598524</td><td>134|147|148|775|4842|5142|6331|6336|59341</td><td>ADORA1|ADRA1B|ADRA1A|CACNA1C|NOS1|PDE4B|SCN5A|SCN10A|TRPV4</td><td>-6.61794</td><td>0</td><td>85</td><td>0</td><td>0</td><td>-8.69967</td><td>45.07004471</td></tr>
<tr><td>1</td><td>-8.02647</td><td>GO:0019226</td><td>M1</td><td>1</td><td>0</td><td>GO Biological Processes</td><td>19</td><td>transmission of nerve impulse</td><td>-8.02647</td><td>19.16021</td><td>11.78345</td><td>30242</td><td>69</td><td>183</td><td>8</td><td>4.371585</td><td>1.511428</td><td>1133|1812|4915|5024|6326|6331|6335|6336</td><td>CHRM5|DRD1|NTRK2|P2RX3|SCN2A|SCN5A|SCN9A|SCN10A</td><td>-6.48727</td><td>0</td><td>85</td><td>0</td><td>0</td><td>-8.69967</td><td>45.07004471</td></tr>
<tr><td>1</td><td>-8.0084</td><td>GO:0001508</td><td>M1</td><td>1</td><td>0</td><td>GO Biological Processes</td><td>19</td><td>action potential</td><td>-8.0084</td><td>12.3326</td><td>10.25835</td><td>30242</td><td>134</td><td>183</td><td>10</td><td>5.464481</td><td>1.680144</td><td>148|775|1268|3757|4915|5024|6326|6331|6335|6336</td><td>ADRA1A|CACNA1C|CNR1|KCNH2|NTRK2|P2RX3|SCN2A|SCN5A|SCN9A|SCN10A</td><td>-6.47233</td><td>0</td><td>85</td><td>0</td><td>0</td><td>-8.69967</td><td>45.07004471</td></tr>
<tr><td>1</td><td>-7.91567</td><td>GO:0060048</td><td>M1</td><td>1</td><td>0</td><td>GO Biological Processes</td><td>19</td><td>cardiac muscle contraction</td><td>-7.91567</td><td>12.06254</td><td>10.12587</td><td>30242</td><td>137</td><td>183</td><td>10</td><td>5.464481</td><td>1.680144</td><td>134|147|148|775|3757|4842|5142|5290|6331|63</td><td>ADORA1|ADRA1B|ADRA1A|CACNA1C|K</td><td>-6.38714</td><td>0</td><td>85</td><td>0</td><td>0</td><td>-8.69967</td><td>45.07004471</td></tr>
</table>

																		36	CNH2|NOS1|PDE4B|PIK3CA|SCN5A|SCN10A							
1	-7.86923	GO:0006941	M1	1	0	GO Biological Processes	19	striated muscle contraction	-7.86923	10.15545	9.585754	30242	179	183	11	6.010929	1.757049	134|147|148|775|3757|4842|5142|5290|6331|6336|59341	ADORA1|ADRA1B|ADRA1A|CACNA1C|KCNH2|NOS1|PDE4B|PIK3CA|SCN5A|SCN10A|TRPV4	-6.34349	0	85	0	0	-8.69967	45.07004471
1	-7.64355	GO:0055117	M1	1	0	GO Biological Processes	19	regulation of cardiac muscle contraction	-7.64355	17.16954	11.0848	30242	77	183	8	4.371585	1.511428	134|147|148|775|4842|5142|6331|6336	ADORA1|ADRA1B|ADRA1A|CACNA1C|NOS1|PDE4B|SCN5A|SCN10A	-6.14479	0	85	0	0	-8.69967	45.07004471
1	-7.24664	GO:0035637	M1	1	0	GO Biological Processes	19	multicellular organismal signaling	-7.24664	10.2644	9.196455	30242	161	183	10	5.464481	1.680144	775|1133|1812|3757|4915|5024|6326|6331|6335|6336	CACNA1C|CHRM5|DRD1|KCNH2|NTRK2|P2RX3|SCN2A|SCN5A|SCN9A|SCN10A	-5.79023	0	85	0	0	-8.69967	45.07004471
1	-7.12062	GO:0086010	M1	1	0	GO Biological Processes	19	membrane depolarization during action potential	-7.12062	27.54281	12.43334	30242	36	183	6	3.278689	1.316393	775|3757|6326|6331|6335|6336	CACNA1C|KCNH2|SCN2A|SCN5A|SCN9A|SCN10A	-5.67283	0	85	0	0	-8.69967	45.07004471
1	-5.86815	GO:0019228	M1	1	0	GO Biological Processes	19	neuronal action potential	-5.86815	25.82138	10.9613	30242	32	183	5	2.73224	1.205087	5024|6326|6331|6335|6336	P2RX3|SCN2A|SCN5A|SCN9A|SCN10A	-4.53728	0	85	0	0	-8.69967	45.07004471
1	-5.25965	GO:0086003	M1	1	0	GO Biological Processes	19	cardiac muscle cell contraction	-5.25965	13.58275	8.398331	30242	73	183	6	3.278689	1.316393	134|775|3757|5142|5290|6331	ADORA1|CACNA1C|KCNH2|PDE4B|PIK3CA|SCN5A	-3.99614	0	85	0	0	-8.69967	45.07004471
1	-4.54796	GO:0070252	M1	1	0	GO Biological Processes	19	actin-mediated cell contraction	-4.54796	10.22207	7.098116	30242	97	183	6	3.278689	1.316393	134|775|3757|5142|5290|6331	ADORA1|CACNA1C|KCNH2|PDE4B|PIK3CA|SCN5A	-3.37728	0	85	0	0	-8.69967	45.07004471
1	-4.45963	GO:0086016	M1	1	0	GO Biological Processes	19	AV node cell action potential	-4.45963	45.07004	11.40644	30242	11	183	3	1.639344	0.938686	775|6331|6336	CACNA1C|SCN5A|SCN10A	-3.30278	0	85	0	0	-8.69967	45.07004471
1	-4.45963	GO:0086027	M1	1	0	GO Biological Processes	19	AV node cell to bundle of His cell signaling	-4.45963	45.07004	11.40644	30242	11	183	3	1.639344	0.938686	775|6331|6336	CACNA1C|SCN5A|SCN10A	-3.30278	0	85	0	0	-8.69967	45.07004471
1	-4.28228	GO:0086004	M1	1	0	GO Biological Processes	19	regulation of cardiac muscle cell contraction	-4.28228	19.44198	8.395032	30242	34	183	4	2.185792	1.080886	134|775|5142|6331	ADORA1|CACNA1C|PDE4B|SCN5A	-3.14168	0	85	0	0	-8.69967	45.07004471
1	-4.12183	GO:0086067	M1	1	0	GO Biological Processes	19	AV node cell to bundle of His cell communication	-4.12183	35.41218	10.04865	30242	14	183	3	1.639344	0.938686	775|6331|6336	CACNA1C|SCN5A|SCN10A	-2.99883	0	85	0	0	-8.69967	45.07004471
1	-4.04368	GO:1903115	M1	1	0	GO Biological Processes	19	regulation of actin filament-based movement	-4.04368	16.94942	7.776577	30242	39	183	4	2.185792	1.080886	134|775|5142|6331	ADORA1|CACNA1C|PDE4B|SCN5A	-2.92688	0	85	0	0	-8.69967	45.07004471
1	-3.89432	GO:0030048	M1	1	0	GO Biological Processes	19	actin filament-based movement	-3.89432	7.807409	5.998306	30242	127	183	6	3.278689	1.316393	134|775|3757|5142|5290|6331	ADORA1|CACNA1C|KCNH2|PDE4B|PIK3CA|SCN5A	-2.7949	0	85	0	0	-8.69967	45.07004471
1	-3.45874	GO:0098900	M1	1	0	GO Biological Processes	19	regulation of action potential	-3.45874	12.01868	6.381713	30242	55	183	4	2.185792	1.080886	148|775|1268|6331	ADRA1A|CACNA1C|CNR1|SCN5A	-2.40826	0	85	0	0	-8.69967	45.07004471
1	-2.94459	GO:0086019	M1	1	0	GO Biological Processes	19	cell-cell signaling involved in cardiac conduction	-2.94459	14.58149	6.182471	30242	34	183	3	1.639344	0.938686	775|6331|6336	CACNA1C|SCN5A|SCN10A	-1.94754	0	85	0	0	-8.69967	45.07004471
1	-2.92617	GO:0086001	M1	1	0	GO Biological Processes	19	cardiac muscle cell action potential	-2.92617	8.697728	5.242601	30242	76	183	4	2.185792	1.080886	775|3757|6331|6336	CACNA1C|KCNH2|SCN5A|SCN10A	-1.92994	0	85	0	0	-8.69967	45.07004471
1	-2.90759	GO:0086005	M1	1	0	GO Biological Processes	19	ventricular cardiac muscle cell action	-2.90759	14.16487	6.080415	30242	35	183	3	1.639344	0.938686	775|3757|6331	CACNA1C|KCNH2|SCN5A	-1.91274	0	85	0	0	-8.69967	45.07004471

								potential																		
1	-2.77031	GO:0086091	M1	1	0	GO Biological Processes	19	regulation of heart rate by cardiac conduction	-2.77031	12.71206	5.710538	30242	39	183	3	1.639344	0.938686	775|3757|6331	CACNA1C|KCNH2|SCN5A	-1.79128	0	85	0	0	-8.69967	45.07004471
1	-2.59076	GO:0003254	M1	1	0	GO Biological Processes	19	regulation of membrane depolarization	-2.59076	11.01712	5.246911	30242	45	183	3	1.639344	0.938686	596|6331|6336	BCL2|SCN5A|SCN10A	-1.63232	0	85	0	0	-8.69967	45.07004471
1	-2.5054	GO:0061337	M1	1	0	GO Biological Processes	19	cardiac conduction	-2.5054	6.677044	4.414515	30242	99	183	4	2.185792	1.080886	775|3757|6331|6336	CACNA1C|KCNH2|SCN5A|SCN10A	-1.55246	0	85	0	0	-8.69967	45.07004471
1	-2.36548	GO:0086002	M1	1	0	GO Biological Processes	19	cardiac muscle cell action potential involved in contraction	-2.36548	9.180935	4.694821	30242	54	183	3	1.639344	0.938686	775|3757|6331	CACNA1C|KCNH2|SCN5A	-1.4267	0	85	0	0	-8.69967	45.07004471
1	-2.2372	GO:0086065	M1	1	0	GO Biological Processes	19	cell communication involved in cardiac conduction	-2.2372	8.262842	4.393843	30242	60	183	3	1.639344	0.938686	775|6331|6336	CACNA1C|SCN5A|SCN10A	-1.30863	0	85	0	0	-8.69967	45.07004471
1	-8.51542	GO:0001776	M1	1	0	GO Biological Processes	19	leukocyte homeostasis	-8.51542	17.09553	11.73064	30242	87	183	9	4.918033	1.598524	100|596|836|3558|3569|5142|5291|5293|729230	ADA|BCL2|CASP3|IL2|IL6|PDE4B|PIK3CB|PIK3CD|CCR2	-6.93222	0	86	1	1	-8.51542	61.97131148
1	-7.34865	GO:0001780	M1	1	0	GO Biological Processes	19	neutrophil homeostasis	-7.34865	48.60495	15.31898	30242	17	183	5	2.73224	1.205087	3569|5142|5291|5293|729230	IL6|PDE4B|PIK3CB|PIK3CD|CCR2	-5.87776	0	86	0	0	-8.51542	61.97131148
1	-6.92307	GO:0048872	M1	1	0	GO Biological Processes	19	homeostasis of number of cells	-6.92307	7.264037	8.112147	30242	273	183	12	6.557377	1.829835	100|596|836|2149|3558|3569|4846|5142|5291|5293|5591|729230	ADA|BCL2|CASP3|F2R|IL2|IL6|NOS3|PDE4B|PIK3CB|PIK3CD|PRKDC|CCR2	-5.49206	0	86	0	0	-8.51542	61.97131148
1	-6.2347	GO:0046488	M1	1	0	GO Biological Processes	19	phosphatidylinositol metabolic process	-6.2347	9.35416	8.240947	30242	159	183	9	4.918033	1.598524	472|1133|3356|3357|3358|5159|5290|5291|5293	ATM|CHRM5|HTR2A|HTR2B|HTR2C|PDGFRB|PIK3CA|PIK3CB|PIK3CD	-4.8635	0	86	0	0	-8.51542	61.97131148
1	-5.53708	GO:0036092	M1	1	0	GO Biological Processes	19	phosphatidylinositol-3-phosphate biosynthetic process	-5.53708	38.88396	12.19082	30242	17	183	4	2.185792	1.080886	472|5290|5291|5293	ATM|PIK3CA|PIK3CB|PIK3CD	-4.23375	0	86	0	0	-8.51542	61.97131148
1	-4.92311	GO:0001781	M1	1	0	GO Biological Processes	19	neutrophil apoptotic process	-4.92311	61.97131	13.45734	30242	8	183	3	1.639344	0.938686	3569|5291|5293	IL6|PIK3CB|PIK3CD	-3.69979	0	86	0	0	-8.51542	61.97131148
1	-4.80364	GO:0006661	M1	1	0	GO Biological Processes	19	phosphatidylinositol biosynthetic process	-4.80364	8.830518	7.008093	30242	131	183	7	3.825137	1.417845	472|3356|3357|3358|5290|5291|5293	ATM|HTR2A|HTR2B|HTR2C|PIK3CA|PIK3CB|PIK3CD	-3.59532	0	86	0	0	-8.51542	61.97131148
1	-4.72551	GO:0006650	M1	1	0	GO Biological Processes	19	glycerophospholipid metabolic process	-4.72551	5.40055	6.036805	30242	306	183	10	5.464481	1.680144	472|1133|3356|3357|3358|5159|5290|5291|5293|5444	ATM|CHRM5|HTR2A|HTR2B|HTR2C|PDGFRB|PIK3CA|PIK3CB|PIK3CD|PON1	-3.52823	0	86	0	0	-8.51542	61.97131148
1	-4.61381	GO:0006644	M1	1	0	GO Biological Processes	19	phospholipid metabolic process	-4.61381	4.73392	5.745616	30242	384	183	11	6.010929	1.757049	472|1133|3356|3357|3358|5159|5290|5291|5293|5444|5467	ATM|CHRM5|HTR2A|HTR2B|HTR2C|PDGFRB|PIK3CA|PIK3CB|PIK3CD|PON1|PPARD	-3.42898	0	86	0	0	-8.51542	61.97131148
1	-4.56263	GO:0033028	M1	1	0	GO Biological Processes	19	myeloid cell apoptotic process	-4.56263	22.79405	9.161705	30242	29	183	4	2.185792	1.080886	596|3569|5291|5293	BCL2|IL6|PIK3CB|PIK3CD	-3.38949	0	86	0	0	-8.51542	61.97131148
1	-4.5321	GO:0046486	M1	1	0	GO Biological Processes	19	glycerolipid metabolic process	-4.5321	4.637309	5.655714	30242	392	183	11	6.010929	1.757049	472|1133|2166|3356|3357|3358|5159|5290|5291|5293|5444	ATM|CHRM5|FAAH|HTR2A|HTR2B|HTR2C|PDGFRB|PIK3CA|PIK3CB|PIK3CD|PON1	-3.36265	0	86	0	0	-8.51542	61.97131148
1	-4.28107	GO:0002262	M1	1	0	GO Biological Processes	19	myeloid cell homeostasis	-4.28107	7.321505	6.216094	30242	158	183	7	3.825137	1.417845	836|3569|5142|5291|5293|5591|729230	CASP3|IL6|PDE4B|PIK3CB|PIK3CD|PRKDC|CCR2	-3.14085	0	86	0	0	-8.51542	61.97131148

<table>
<tr><td>1</td><td>-4.08257</td><td>GO:0035924</td><td>M1</td><td>1</td><td>0</td><td>GO Biological Processes</td><td>19</td><td>cellular response to vascular endothelial growth factor stimulus</td><td>-4.08257</td><td>11.31896</td><td>6.887368</td><td>30242</td><td>73</td><td>183</td><td>5</td><td>2.73224</td><td>1.205087</td><td>5159|5290|5291|5293|5970</td><td>PDGFRB|PIK3CA|PIK3CB|PIK3CD|RELA</td><td>-2.96323</td><td>0</td><td>86</td><td>0</td><td>0</td><td>-8.51542</td><td>61.97131148</td></tr>
<tr><td>1</td><td>-3.78751</td><td>GO:0008654</td><td>M1</td><td>1</td><td>0</td><td>GO Biological Processes</td><td>19</td><td>phospholipid biosynthetic process</td><td>-3.78751</td><td>5.225512</td><td>5.266172</td><td>30242</td><td>253</td><td>183</td><td>8</td><td>4.371585</td><td>1.511428</td><td>472|3356|3357|3358|5290|5291|5293|5467</td><td>ATM|HTR2A|HTR2B|HTR2C|PIK3CA|PIK3CB|PIK3CD|PPARD</td><td>-2.69874</td><td>0</td><td>86</td><td>0</td><td>0</td><td>-8.51542</td><td>61.97131148</td></tr>
<tr><td>1</td><td>-3.65322</td><td>GO:0038084</td><td>M1</td><td>1</td><td>0</td><td>GO Biological Processes</td><td>19</td><td>vascular endothelial growth factor signaling pathway</td><td>-3.65322</td><td>13.49035</td><td>6.82741</td><td>30242</td><td>49</td><td>183</td><td>4</td><td>2.185792</td><td>1.080886</td><td>5159|5290|5291|5293</td><td>PDGFRB|PIK3CA|PIK3CB|PIK3CD</td><td>-2.58304</td><td>0</td><td>86</td><td>0</td><td>0</td><td>-8.51542</td><td>61.97131148</td></tr>
<tr><td>1</td><td>-3.57263</td><td>GO:0006925</td><td>M1</td><td>1</td><td>0</td><td>GO Biological Processes</td><td>19</td><td>inflammatory cell apoptotic process</td><td>-3.57263</td><td>23.60812</td><td>8.086415</td><td>30242</td><td>21</td><td>183</td><td>3</td><td>1.639344</td><td>0.938686</td><td>3569|5291|5293</td><td>IL6|PIK3CB|PIK3CD</td><td>-2.51178</td><td>0</td><td>86</td><td>0</td><td>0</td><td>-8.51542</td><td>61.97131148</td></tr>
<tr><td>1</td><td>-3.50313</td><td>GO:0046474</td><td>M1</td><td>1</td><td>0</td><td>GO Biological Processes</td><td>19</td><td>glycerophospholipid biosynthetic process</td><td>-3.50313</td><td>5.482454</td><td>5.098102</td><td>30242</td><td>211</td><td>183</td><td>7</td><td>3.825137</td><td>1.417845</td><td>472|3356|3357|3358|5290|5291|5293</td><td>ATM|HTR2A|HTR2B|HTR2C|PIK3CA|PIK3CB|PIK3CD</td><td>-2.4486</td><td>0</td><td>86</td><td>0</td><td>0</td><td>-8.51542</td><td>61.97131148</td></tr>
<tr><td>1</td><td>-3.39607</td><td>GO:0033032</td><td>M1</td><td>1</td><td>0</td><td>GO Biological Processes</td><td>19</td><td>regulation of myeloid cell apoptotic process</td><td>-3.39607</td><td>20.6571</td><td>7.516725</td><td>30242</td><td>24</td><td>183</td><td>3</td><td>1.639344</td><td>0.938686</td><td>596|5291|5293</td><td>BCL2|PIK3CB|PIK3CD</td><td>-2.35299</td><td>0</td><td>86</td><td>0</td><td>0</td><td>-8.51542</td><td>61.97131148</td></tr>
<tr><td>1</td><td>-3.29117</td><td>GO:2000108</td><td>M1</td><td>1</td><td>0</td><td>GO Biological Processes</td><td>19</td><td>positive regulation of leukocyte apoptotic process</td><td>-3.29117</td><td>19.0681</td><td>7.191458</td><td>30242</td><td>26</td><td>183</td><td>3</td><td>1.639344</td><td>0.938686</td><td>5291|5293|7157</td><td>PIK3CB|PIK3CD|TP53</td><td>-2.25835</td><td>0</td><td>86</td><td>0</td><td>0</td><td>-8.51542</td><td>61.97131148</td></tr>
<tr><td>1</td><td>-3.20691</td><td>GO:0046854</td><td>M1</td><td>1</td><td>0</td><td>GO Biological Processes</td><td>19</td><td>phosphatidylinositol phosphate biosynthetic process</td><td>-3.20691</td><td>10.32855</td><td>5.829017</td><td>30242</td><td>64</td><td>183</td><td>4</td><td>2.185792</td><td>1.080886</td><td>472|5290|5291|5293</td><td>ATM|PIK3CA|PIK3CB|PIK3CD</td><td>-2.18207</td><td>0</td><td>86</td><td>0</td><td>0</td><td>-8.51542</td><td>61.97131148</td></tr>
<tr><td>1</td><td>-3.0458</td><td>GO:0045017</td><td>M1</td><td>1</td><td>0</td><td>GO Biological Processes</td><td>19</td><td>glycerolipid biosynthetic process</td><td>-3.0458</td><td>4.590468</td><td>4.465801</td><td>30242</td><td>252</td><td>183</td><td>7</td><td>3.825137</td><td>1.417845</td><td>472|3356|3357|3358|5290|5291|5293</td><td>ATM|HTR2A|HTR2B|HTR2C|PIK3CA|PIK3CB|PIK3CD</td><td>-2.0379</td><td>0</td><td>86</td><td>0</td><td>0</td><td>-8.51542</td><td>61.97131148</td></tr>
<tr><td>1</td><td>-2.08679</td><td>GO:0070527</td><td>M1</td><td>1</td><td>0</td><td>GO Biological Processes</td><td>19</td><td>platelet aggregation</td><td>-2.08679</td><td>7.290743</td><td>4.052064</td><td>30242</td><td>68</td><td>183</td><td>3</td><td>1.639344</td><td>0.938686</td><td>2335|3569|5291</td><td>FN1|IL6|PIK3CB</td><td>-1.16956</td><td>0</td><td>86</td><td>0</td><td>0</td><td>-8.51542</td><td>61.97131148</td></tr>
<tr><td>1</td><td>-8.46118</td><td>GO:0042596</td><td>M1</td><td>1</td><td>0</td><td>GO Biological Processes</td><td>19</td><td>fear response</td><td>-8.46118</td><td>29.66148</td><td>13.97469</td><td>30242</td><td>39</td><td>183</td><td>7</td><td>3.825137</td><td>1.417845</td><td>150|153|596|1815|2558|3350|3358</td><td>ADRA2A|ADRB1|BCL2|DRD4|GABRA5|HTR1A|HTR2C</td><td>-6.88741</td><td>0</td><td>87</td><td>1</td><td>1</td><td>-8.46118</td><td>29.66148242</td></tr>
<tr><td>1</td><td>-5.66716</td><td>GO:0001662</td><td>M1</td><td>1</td><td>0</td><td>GO Biological Processes</td><td>19</td><td>behavioral fear response</td><td>-5.66716</td><td>23.60812</td><td>10.44194</td><td>30242</td><td>35</td><td>183</td><td>5</td><td>2.73224</td><td>1.205087</td><td>596|1815|2558|3350|3358</td><td>BCL2|DRD4|GABRA5|HTR1A|HTR2C</td><td>-4.35145</td><td>0</td><td>87</td><td>0</td><td>0</td><td>-8.46118</td><td>29.66148242</td></tr>
<tr><td>1</td><td>-5.60435</td><td>GO:0002209</td><td>M1</td><td>1</td><td>0</td><td>GO Biological Processes</td><td>19</td><td>behavioral defense response</td><td>-5.60435</td><td>22.95234</td><td>10.28305</td><td>30242</td><td>36</td><td>183</td><td>5</td><td>2.73224</td><td>1.205087</td><td>596|1815|2558|3350|3358</td><td>BCL2|DRD4|GABRA5|HTR1A|HTR2C</td><td>-4.29598</td><td>0</td><td>87</td><td>0</td><td>0</td><td>-8.46118</td><td>29.66148242</td></tr>
<tr><td>1</td><td>-8.46118</td><td>GO:0007618</td><td>M1</td><td>1</td><td>0</td><td>GO Biological Processes</td><td>19</td><td>mating</td><td>-8.46118</td><td>29.66148</td><td>13.97469</td><td>30242</td><td>39</td><td>183</td><td>7</td><td>3.825137</td><td>1.417845</td><td>100|351|1268|1812|1910|3066|6532</td><td>ADA|APP|CNR1|DRD1|EDNRB|HDAC2|SLC6A4</td><td>-6.88741</td><td>0</td><td>88</td><td>1</td><td>1</td><td>-8.46118</td><td>70.82435597</td></tr>
<tr><td>1</td><td>-5.23453</td><td>GO:0007620</td><td>M1</td><td>1</td><td>0</td><td>GO Biological Processes</td><td>19</td><td>copulation</td><td>-5.23453</td><td>33.05137</td><td>11.18758</td><td>30242</td><td>20</td><td>183</td><td>4</td><td>2.185792</td><td>1.080886</td><td>100|1268|1910|6532</td><td>ADA|CNR1|EDNRB|SLC6A4</td><td>-3.97355</td><td>0</td><td>88</td><td>0</td><td>0</td><td>-8.46118</td><td>70.82435597</td></tr>
<tr><td>1</td><td>-5.12529</td><td>GO:0060405</td><td>M1</td><td>1</td><td>0</td><td>GO Biological Processes</td><td>19</td><td>regulation of penile erection</td><td>-5.12529</td><td>70.82436</td><td>14.41575</td><td>30242</td><td>7</td><td>183</td><td>3</td><td>1.639344</td><td>0.938686</td><td>100|1268|1910</td><td>ADA|CNR1|EDNRB</td><td>-3.87821</td><td>0</td><td>88</td><td>0</td><td>0</td><td>-8.46118</td><td>70.82435597</td></tr>
<tr><td>1</td><td>-4.596</td><td>GO:0043084</td><td>M1</td><td>1</td><td>0</td><td>GO Biological Processes</td><td>19</td><td>penile erection</td><td>-4.596</td><td>49.57705</td><td>11.98765</td><td>30242</td><td>10</td><td>183</td><td>3</td><td>1.639344</td><td>0.938686</td><td>100|1268|1910</td><td>ADA|CNR1|EDNRB</td><td>-3.4183</td><td>0</td><td>88</td><td>0</td><td>0</td><td>-8.46118</td><td>70.82435597</td></tr>
<tr><td>1</td><td>-4.08071</td><td>GO:2000241</td><td>M1</td><td>1</td><td>0</td><td>GO Biological Processes</td><td>19</td><td>regulation of reproductive process</td><td>-4.08071</td><td>6.804693</td><td>5.921871</td><td>30242</td><td>170</td><td>183</td><td>7</td><td>3.825137</td><td>1.417845</td><td>100|367|1268|1910|2099|3066|225689</td><td>ADA|AR|CNR1|EDNRB|ESR1|HDAC2|MAPK15</td><td>-2.96173</td><td>0</td><td>88</td><td>0</td><td>0</td><td>-8.46118</td><td>70.82435597</td></tr>
<tr><td>1</td><td>-2.82347</td><td>GO:2000243</td><td>M1</td><td>1</td><td>0</td><td>GO Biological Processes</td><td>19</td><td>positive regulation of reproductive process</td><td>-2.82347</td><td>8.160831</td><td>5.035231</td><td>30242</td><td>81</td><td>183</td><td>4</td><td>2.185792</td><td>1.080886</td><td>367|1910|3066|225689</td><td>AR|EDNRB|HDAC2|MAPK15</td><td>-1.83834</td><td>0</td><td>88</td><td>0</td><td>0</td><td>-8.46118</td><td>70.82435597</td></tr>
<tr><td>1</td><td>-8.37772</td><td>GO:0010888</td><td>M1</td><td>1</td><td>0</td><td>GO Biological Processes</td><td>19</td><td>negative regulation of lipid storage</td><td>-8.37772</td><td>43.11048</td><td>15.76342</td><td>30242</td><td>23</td><td>183</td><td>6</td><td>3.278689</td><td>1.316393</td><td>3569|4792|5465|5467|5468|7124</td><td>IL6|NFKBIA|PPARA|PPARD|PPARG|TNF</td><td>-6.81115</td><td>0</td><td>89</td><td>1</td><td>1</td><td>-8.37772</td><td>43.11047755</td></tr>
</table>

1	-5.89613	GO:0010883	M1	1	0	GO Biological Processes	19	regulation of lipid storage	-5.89613	17.39546	9.667222	30242	57	183	6	3.278689	1.316393	3569|4792|5465|5467|5468|7124	IL6|NFKBIA|PPARA|PPARD|PPARG|TNF	-4.56049	0	89	0	0	-8.37772	43.11047755
1	-5.76521	GO:1905952	M1	1	0	GO Biological Processes	19	regulation of lipid localization	-5.76521	8.21719	7.598749	30242	181	183	9	4.918033	1.598524	185|1588|3569|4792|5444|5465|5467|5468|7124	AGTR1|CYP19A1|IL6|NFKBIA|PON1|PPARA|PPARD|PPARG|TNF	-4.43905	0	89	0	0	-8.37772	43.11047755
1	-5.51711	GO:1905953	M1	1	0	GO Biological Processes	19	negative regulation of lipid localization	-5.51711	15.02335	8.898754	30242	66	183	6	3.278689	1.316393	3569|4792|5465|5467|5468|7124	IL6|NFKBIA|PPARA|PPARD|PPARG|TNF	-4.216	0	89	0	0	-8.37772	43.11047755
1	-5.48428	GO:0010742	M1	1	0	GO Biological Processes	19	macrophage derived foam cell differentiation	-5.48428	21.74432	9.983783	30242	38	183	5	2.73224	1.205087	185|4792|5465|5468|7040	AGTR1|NFKBIA|PPARA|PPARG|TGFB1	-4.18647	0	89	0	0	-8.37772	43.11047755
1	-5.48428	GO:0090077	M1	1	0	GO Biological Processes	19	foam cell differentiation	-5.48428	21.74432	9.983783	30242	38	183	5	2.73224	1.205087	185|4792|5465|5468|7040	AGTR1|NFKBIA|PPARA|PPARG|TGFB1	-4.18647	0	89	0	0	-8.37772	43.11047755
1	-4.81782	GO:0019915	M1	1	0	GO Biological Processes	19	lipid storage	-4.81782	11.39702	7.577489	30242	87	183	6	3.278689	1.316393	3569|4792|5465|5467|5468|7124	IL6|NFKBIA|PPARA|PPARD|PPARG|TNF	-3.60816	0	89	0	0	-8.37772	43.11047755
1	-4.38865	GO:0010743	M1	1	0	GO Biological Processes	19	regulation of macrophage derived foam cell differentiation	-4.38865	20.6571	8.680715	30242	32	183	4	2.185792	1.080886	185|4792|5465|5468	AGTR1|NFKBIA|PPARA|PPARG	-3.23843	0	89	0	0	-8.37772	43.11047755
1	-4.22463	GO:0010745	M1	1	0	GO Biological Processes	19	negative regulation of macrophage derived foam cell differentiation	-4.22463	38.13619	10.44945	30242	13	183	3	1.639344	0.938686	4792|5465|5468	NFKBIA|PPARA|PPARG	-3.09123	0	89	0	0	-8.37772	43.11047755
1	-3.77899	GO:0030730	M1	1	0	GO Biological Processes	19	sequestering of triglyceride	-3.77899	27.54281	8.789081	30242	18	183	3	1.639344	0.938686	5465|5468|7124	PPARA|PPARG|TNF	-2.69427	0	89	0	0	-8.37772	43.11047755
1	-3.51777	GO:0032368	M1	1	0	GO Biological Processes	19	regulation of lipid transport	-3.51777	6.654637	5.398853	30242	149	183	6	3.278689	1.316393	185|1588|4792|5444|5465|5468	AGTR1|CYP19A1|NFKBIA|PON1|PPARA|PPARG	-2.46009	0	89	0	0	-8.37772	43.11047755
1	-3.29117	GO:0010875	M1	1	0	GO Biological Processes	19	positive regulation of cholesterol efflux	-3.29117	19.0681	7.191458	30242	26	183	3	1.639344	0.938686	4792|5444|5468	NFKBIA|PON1|PPARG	-2.25835	0	89	0	0	-8.37772	43.11047755
1	-3.15625	GO:2000272	M1	1	0	GO Biological Processes	19	negative regulation of signaling receptor activity	-3.15625	10.01557	5.72098	30242	66	183	4	2.185792	1.080886	2100|5465|5468|7124	ESR2|PPARA|PPARG|TNF	-2.13694	0	89	0	0	-8.37772	43.11047755
1	-3.12807	GO:0030301	M1	1	0	GO Biological Processes	19	cholesterol transport	-3.12807	7.062258	5.126254	30242	117	183	5	2.73224	1.205087	1080|1645|4792|5444|5468	CFTR|AKR1C1|NFKBIA|PON1|PPARG	-2.11049	0	89	0	0	-8.37772	43.11047755
1	-2.92284	GO:0015918	M1	1	0	GO Biological Processes	19	sterol transport	-2.92284	6.356032	4.775088	30242	130	183	5	2.73224	1.205087	1080|1645|4792|5444|5468	CFTR|AKR1C1|NFKBIA|PON1|PPARG	-1.92688	0	89	0	0	-8.37772	43.11047755
1	-2.89337	GO:0045667	M1	1	0	GO Biological Processes	19	regulation of osteoblast differentiation	-2.89337	6.259728	4.72532	30242	132	183	5	2.73224	1.205087	2263|3569|4221|5468|7124	FGFR2|IL6|MEN1|PPARG|TNF	-1.90097	0	89	0	0	-8.37772	43.11047755
1	-2.80314	GO:0032373	M1	1	0	GO Biological Processes	19	positive regulation of sterol transport	-2.80314	13.04659	5.797758	30242	38	183	3	1.639344	0.938686	4792|5444|5468	NFKBIA|PON1|PPARG	-1.82068	0	89	0	0	-8.37772	43.11047755
1	-2.80314	GO:0032376	M1	1	0	GO Biological Processes	19	positive regulation of cholesterol transport	-2.80314	13.04659	5.797758	30242	38	183	3	1.639344	0.938686	4792|5444|5468	NFKBIA|PON1|PPARG	-1.82068	0	89	0	0	-8.37772	43.11047755
1	-2.76521	GO:0032370	M1	1	0	GO Biological Processes	19	positive regulation of lipid transport	-2.76521	7.869373	4.91917	30242	84	183	4	2.185792	1.080886	1588|4792|5444|5468	CYP19A1|NFKBIA|PON1|PPARG	-1.78724	0	89	0	0	-8.37772	43.11047755
1	-2.54493	GO:0001649	M1	1	0	GO Biological Processes	19	osteoblast differentiation	-2.54493	4.311048	3.932948	30242	230	183	6	3.278689	1.316393	249|2263|3569|4221|5468|7124	ALPL|FGFR2|IL6|MEN1|PPARG|TNF	-1.58925	0	89	0	0	-8.37772	43.11047755
1	-2.43567	GO:0010874	M1	1	0	GO Biological Processes	19	regulation of cholesterol efflux	-2.43567	9.72099	4.863495	30242	51	183	3	1.639344	0.938686	4792|5444|5468	NFKBIA|PON1|PPARG	-1.49059	0	89	0	0	-8.37772	43.11047755
1	-2.43567	GO:0045668	M1	1	0	GO Biological Processes	19	negative regulation of osteoblast	-2.43567	9.72099	4.863495	30242	51	183	3	1.639344	0.938686	4221|5468|7124	MEN1|PPARG|TNF	-1.49059	0	89	0	0	-8.37772	43.11047755

								differentiation																		
1	-2.34194	GO:1905954	M1	1	0	GO Biological Processes	19	positive regulation of lipid localization	-2.34194	6.009339	4.106759	30242	110	183	4	2.185792	1.080886	1588|4792|5444|5468	CYP19A1|NFKBIA|PON1|PPARG	-1.40555	0	89	0	0	-8.37772	43.11047755
1	-2.06939	GO:0033344	M1	1	0	GO Biological Processes	19	cholesterol efflux	-2.06939	7.18508	4.013257	30242	69	183	3	1.639344	0.938686	4792|5444|5468	NFKBIA|PON1|PPARG	-1.15307	0	89	0	0	-8.37772	43.11047755
1	-8.25282	GO:1902532	M1	1	0	GO Biological Processes	19	negative regulation of intracellular signal transduction	-8.25282	5.529039	8.270392	30242	538	183	18	9.836066	2.201413	148|472|596|624|1813|1814|2099|3156|4221|4318|5138|5291|5465|5468|5743|5970|7099|114548	ADRA1A|ATM|BCL2|BDKRB2|DRD2|DRD3|ESR1|HMGCR|MEN1|MMP9|PDE2A|PIK3CB|PPARA|PPARG|PTGS2|RELA|TLR4|NLRP3	-6.69232	0	90	1	1	-8.25282	14.58148505
1	-7.8621	GO:0045936	M1	1	0	GO Biological Processes	19	negative regulation of phosphate metabolic process	-7.8621	5.982148	8.232959	30242	442	183	16	8.743169	2.088053	135|624|836|1813|3156|3551|3558|4221|5291|5465|5468|5591|7040|7124|7157|9475	ADORA2A|BDKRB2|CASP3|DRD2|HMGCR|IKBKB|IL2|MEN1|PIK3CB|PPARA|PPARG|PRKDC|TGFB1|TNF|TP53|ROCK2	-6.33821	0	90	0	0	-8.25282	14.58148505
1	-7.8484	GO:0010563	M1	1	0	GO Biological Processes	19	negative regulation of phosphorus metabolic process	-7.8484	5.968644	8.220065	30242	443	183	16	8.743169	2.088053	135|624|836|1813|3156|3551|3558|4221|5291|5465|5468|5591|7040|7124|7157|9475	ADORA2A|BDKRB2|CASP3|DRD2|HMGCR|IKBKB|IL2|MEN1|PIK3CB|PPARA|PPARG|PRKDC|TGFB1|TNF|TP53|ROCK2	-6.32543	0	90	0	0	-8.25282	14.58148505
1	-7.76908	GO:0043086	M1	1	0	GO Biological Processes	19	negative regulation of catalytic activity	-7.76908	4.376284	7.517556	30242	793	183	21	11.47541	2.356083	135|351|836|1268|1728|1813|1814|1910|3156|3551|4221|4318|4842|5291|5468|5743|6197|7124|7157|9475|729230	ADORA2A|APP|CASP3|CNR1|NQO1|DRD2|DRD3|EDNRB|HMGCR|IKBKB|MEN1|MMP9|NOS1|PIK3CB|PPARG|PTGS2|RPS6KA3|TNF|TP53|ROCK2|CCR2	-6.25524	0	90	0	0	-8.25282	14.58148505
1	-7.70999	GO:0031400	M1	1	0	GO Biological Processes	19	negative regulation of protein modification process	-7.70999	5.433977	7.934401	30242	517	183	17	9.289617	2.145862	135|624|836|1813|3066|3156|3551|3558|4221|5291|5468|5582|5591|5970|7040|7124|9475	ADORA2A|BDKRB2|CASP3|DRD2|HDAC2|HMGCR|IKBKB|IL2|MEN1|PIK3CB|PPARG|PRKCG|PRKDC|RELA|TGFB1|TNF|ROCK2	-6.20508	0	90	0	0	-8.25282	14.58148505
1	-5.34301	GO:0042326	M1	1	0	GO Biological Processes	19	negative regulation of phosphorylation	-5.34301	5.137518	6.38343	30242	386	183	12	6.557377	1.829835	135|624|836|1813|3156|3558|4221|5291|5465|5468|5591|7040	ADORA2A|BDKRB2|CASP3|DRD2|HMGCR|IL2|MEN1|PIK3CB|PPARA|PPARG|PRKDC|TGFB1	-4.0682	0	90	0	0	-8.25282	14.58148505
1	-5.06809	GO:0001933	M1	1	0	GO Biological Processes	19	negative regulation of protein phosphorylation	-5.06809	5.299782	6.24887	30242	343	183	11	6.010929	1.757049	135|624|836|1813|3156|3558|4221|5291|5468|5591|7040	ADORA2A|BDKRB2|CASP3|DRD2|HMGCR|IL2|MEN1|PIK3CB|PPARG|PRKDC|TGFB1	-3.82441	0	90	0	0	-8.25282	14.58148505
1	-3.07846	GO:0071901	M1	1	0	GO Biological Processes	19	negative regulation of protein serine/threonine	-3.07846	6.885701	5.040612	30242	120	183	5	2.73224	1.205087	836|3156|4221|5291|5468	CASP3|HMGCR|MEN1|PIK3CB|PPARG	-2.06717	0	90	0	0	-8.25282	14.58148505

<table>
<tr><td></td><td></td><td></td><td></td><td></td><td></td><td></td><td></td><td>kinase activity</td><td></td><td></td><td></td><td></td><td></td><td></td><td></td><td></td><td></td><td></td><td></td><td></td><td></td><td></td><td></td><td></td><td></td><td></td></tr>
<tr><td>1</td><td>-2.94459</td><td>GO:0010661</td><td>M1</td><td>1</td><td>0</td><td>GO Biological Processes</td><td>19</td><td>positive regulation of muscle cell apoptotic process</td><td>-2.94459</td><td>14.58149</td><td>6.182471</td><td>30242</td><td>34</td><td>183</td><td>3</td><td>1.639344</td><td>0.938686</td><td>3156|5468|7157</td><td>HMGCR|PPARG|TP53</td><td>-1.94754</td><td>0</td><td>90</td><td>0</td><td>0</td><td>-8.25282</td><td>14.58148505</td></tr>
<tr><td>1</td><td>-2.87288</td><td>GO:0051348</td><td>M1</td><td>1</td><td>0</td><td>GO Biological Processes</td><td>19</td><td>negative regulation of transferase activity</td><td>-2.87288</td><td>4.284436</td><td>4.229814</td><td>30242</td><td>270</td><td>183</td><td>7</td><td>3.825137</td><td>1.417845</td><td>135|836|3156|4221|5291|5468|7157</td><td>ADORA2A|CASP3|HMGCR|MEN1|PIK3CB|PPARG|TP53</td><td>-1.88231</td><td>0</td><td>90</td><td>0</td><td>0</td><td>-8.25282</td><td>14.58148505</td></tr>
<tr><td>1</td><td>-2.71089</td><td>GO:0006469</td><td>M1</td><td>1</td><td>0</td><td>GO Biological Processes</td><td>19</td><td>negative regulation of protein kinase activity</td><td>-2.71089</td><td>4.655122</td><td>4.176934</td><td>30242</td><td>213</td><td>183</td><td>6</td><td>3.278689</td><td>1.316393</td><td>135|836|3156|4221|5291|5468</td><td>ADORA2A|CASP3|HMGCR|MEN1|PIK3CB|PPARG</td><td>-1.73762</td><td>0</td><td>90</td><td>0</td><td>0</td><td>-8.25282</td><td>14.58148505</td></tr>
<tr><td>1</td><td>-2.47198</td><td>GO:0033673</td><td>M1</td><td>1</td><td>0</td><td>GO Biological Processes</td><td>19</td><td>negative regulation of kinase activity</td><td>-2.47198</td><td>4.166139</td><td>3.826178</td><td>30242</td><td>238</td><td>183</td><td>6</td><td>3.278689</td><td>1.316393</td><td>135|836|3156|4221|5291|5468</td><td>ADORA2A|CASP3|HMGCR|MEN1|PIK3CB|PPARG</td><td>-1.52299</td><td>0</td><td>90</td><td>0</td><td>0</td><td>-8.25282</td><td>14.58148505</td></tr>
<tr><td>1</td><td>-2.31151</td><td>GO:0043409</td><td>M1</td><td>1</td><td>0</td><td>GO Biological Processes</td><td>19</td><td>negative regulation of MAPK cascade</td><td>-2.31151</td><td>4.590468</td><td>3.769768</td><td>30242</td><td>180</td><td>183</td><td>5</td><td>2.73224</td><td>1.205087</td><td>3156|4221|5291|5468|7099</td><td>HMGCR|MEN1|PIK3CB|PPARG|TLR4</td><td>-1.37727</td><td>0</td><td>90</td><td>0</td><td>0</td><td>-8.25282</td><td>14.58148505</td></tr>
<tr><td>1</td><td>-2.25757</td><td>GO:0043407</td><td>M1</td><td>1</td><td>0</td><td>GO Biological Processes</td><td>19</td><td>negative regulation of MAP kinase activity</td><td>-2.25757</td><td>8.40289</td><td>4.441017</td><td>30242</td><td>59</td><td>183</td><td>3</td><td>1.639344</td><td>0.938686</td><td>3156|5291|5468</td><td>HMGCR|PIK3CB|PPARG</td><td>-1.32711</td><td>0</td><td>90</td><td>0</td><td>0</td><td>-8.25282</td><td>14.58148505</td></tr>
<tr><td>1</td><td>-8.23403</td><td>GO:0048565</td><td>M1</td><td>1</td><td>0</td><td>GO Biological Processes</td><td>19</td><td>digestive tract development</td><td>-8.23403</td><td>13.01235</td><td>10.58461</td><td>30242</td><td>127</td><td>183</td><td>10</td><td>5.464481</td><td>1.680144</td><td>100|596|1543|1910|1956|2263|3576|5979|7124|7298</td><td>ADA|BCL2|CYP1A1|EDNRB|EGFR|FGFR2|CXCL8|RET|TNF|TYMS</td><td>-6.67454</td><td>0</td><td>91</td><td>1</td><td>1</td><td>-8.23403</td><td>21.32346201</td></tr>
<tr><td>1</td><td>-7.91567</td><td>GO:0055123</td><td>M1</td><td>1</td><td>0</td><td>GO Biological Processes</td><td>19</td><td>digestive system development</td><td>-7.91567</td><td>12.06254</td><td>10.12587</td><td>30242</td><td>137</td><td>183</td><td>10</td><td>5.464481</td><td>1.680144</td><td>100|596|1543|1910|1956|2263|3576|5979|7124|7298</td><td>ADA|BCL2|CYP1A1|EDNRB|EGFR|FGFR2|CXCL8|RET|TNF|TYMS</td><td>-6.38714</td><td>0</td><td>91</td><td>0</td><td>0</td><td>-8.23403</td><td>21.32346201</td></tr>
<tr><td>1</td><td>-4.44459</td><td>GO:0048566</td><td>M1</td><td>1</td><td>0</td><td>GO Biological Processes</td><td>19</td><td>embryonic digestive tract development</td><td>-4.44459</td><td>21.32346</td><td>8.83349</td><td>30242</td><td>31</td><td>183</td><td>4</td><td>2.185792</td><td>1.080886</td><td>100|2263|3576|7124</td><td>ADA|FGFR2|CXCL8|TNF</td><td>-3.29079</td><td>0</td><td>91</td><td>0</td><td>0</td><td>-8.23403</td><td>21.32346201</td></tr>
<tr><td>1</td><td>-3.97124</td><td>GO:0030324</td><td>M1</td><td>1</td><td>0</td><td>GO Biological Processes</td><td>19</td><td>lung development</td><td>-3.97124</td><td>6.535581</td><td>5.763092</td><td>30242</td><td>177</td><td>183</td><td>7</td><td>3.825137</td><td>1.417845</td><td>100|1544|1956|2263|5159|5241|7124</td><td>ADA|CYP1A2|EGFR|FGFR2|PDGFRB|PGR|TNF</td><td>-2.86187</td><td>0</td><td>91</td><td>0</td><td>0</td><td>-8.23403</td><td>21.32346201</td></tr>
<tr><td>1</td><td>-3.91091</td><td>GO:0030323</td><td>M1</td><td>1</td><td>0</td><td>GO Biological Processes</td><td>19</td><td>respiratory tube development</td><td>-3.91091</td><td>6.391148</td><td>5.676167</td><td>30242</td><td>181</td><td>183</td><td>7</td><td>3.825137</td><td>1.417845</td><td>100|1544|1956|2263|5159|5241|7124</td><td>ADA|CYP1A2|EGFR|FGFR2|PDGFRB|PGR|TNF</td><td>-2.81115</td><td>0</td><td>91</td><td>0</td><td>0</td><td>-8.23403</td><td>21.32346201</td></tr>
<tr><td>1</td><td>-3.60485</td><td>GO:0060541</td><td>M1</td><td>1</td><td>0</td><td>GO Biological Processes</td><td>19</td><td>respiratory system development</td><td>-3.60485</td><td>5.698511</td><td>5.240852</td><td>30242</td><td>203</td><td>183</td><td>7</td><td>3.825137</td><td>1.417845</td><td>100|1544|1956|2263|5159|5241|7124</td><td>ADA|CYP1A2|EGFR|FGFR2|PDGFRB|PGR|TNF</td><td>-2.5392</td><td>0</td><td>91</td><td>0</td><td>0</td><td>-8.23403</td><td>21.32346201</td></tr>
<tr><td>1</td><td>-3.02224</td><td>GO:0050850</td><td>M1</td><td>1</td><td>0</td><td>GO Biological Processes</td><td>19</td><td>positive regulation of calcium-mediated signaling</td><td>-3.02224</td><td>15.49283</td><td>6.400135</td><td>30242</td><td>32</td><td>183</td><td>3</td><td>1.639344</td><td>0.938686</td><td>100|5024|7124</td><td>ADA|P2RX3|TNF</td><td>-2.01659</td><td>0</td><td>91</td><td>0</td><td>0</td><td>-8.23403</td><td>21.32346201</td></tr>
<tr><td>1</td><td>-2.70729</td><td>GO:0048286</td><td>M1</td><td>1</td><td>0</td><td>GO Biological Processes</td><td>19</td><td>lung alveolus development</td><td>-2.70729</td><td>12.09196</td><td>5.545313</td><td>30242</td><td>41</td><td>183</td><td>3</td><td>1.639344</td><td>0.938686</td><td>100|2263|5241</td><td>ADA|FGFR2|PGR</td><td>-1.73455</td><td>0</td><td>91</td><td>0</td><td>0</td><td>-8.23403</td><td>21.32346201</td></tr>
<tr><td>1</td><td>-2.32091</td><td>GO:0048568</td><td>M1</td><td>1</td><td>0</td><td>GO Biological Processes</td><td>19</td><td>embryonic organ development</td><td>-2.32091</td><td>3.081712</td><td>3.388308</td><td>30242</td><td>429</td><td>183</td><td>8</td><td>4.371585</td><td>1.511428</td><td>100|2263|3576|4325|7040|7046|7124|7157</td><td>ADA|FGFR2|CXCL8|MMP16|TGFB1|TGFBR1|TNF|TP53</td><td>-1.38595</td><td>0</td><td>91</td><td>0</td><td>0</td><td>-8.23403</td><td>21.32346201</td></tr>
<tr><td>1</td><td>-2.00253</td><td>GO:0050848</td><td>M1</td><td>1</td><td>0</td><td>GO Biological Processes</td><td>19</td><td>regulation of calcium-mediated signaling</td><td>-2.00253</td><td>6.791377</td><td>3.865442</td><td>30242</td><td>73</td><td>183</td><td>3</td><td>1.639344</td><td>0.938686</td><td>100|5024|7124</td><td>ADA|P2RX3|TNF</td><td>-1.09008</td><td>0</td><td>91</td><td>0</td><td>0</td><td>-8.23403</td><td>21.32346201</td></tr>
<tr><td>1</td><td>-8.16698</td><td>GO:0030162</td><td>M1</td><td>1</td><td>0</td><td>GO Biological Processes</td><td>19</td><td>regulation of proteolysis</td><td>-8.16698</td><td>4.614885</td><td>7.832535</td><td>30242</td><td>752</td><td>183</td><td>21</td><td>11.47541</td><td>2.356083</td><td>135|150|351|834|1453|2147|2149|2335|2902|2903|2904|3066|4318|5468|5582|5743|6197|7124|7157|9475|114548</td><td>ADORA2A|ADRA2A|APP|CASP1|CSNK1D|F2|F2R|FN1|GRIN1|GRIN2A|GRIN2B|HDAC2|MMP9|PPARG|PR</td><td>-6.61247</td><td>0</td><td>92</td><td>1</td><td>1</td><td>-8.16698</td><td>9.089125683</td></tr>
</table>

																			KCG|PTGS2|RPS6KA3|TNF|TP53|ROCK2|NLRP3							
1	-7.57452	GO:2000116	M1	1	0	GO Biological Processes	19	regulation of cysteine-type endopeptidase activity	-7.57452	8.332277	8.860824	30242	238	183	12	6.557377	1.829835	135|834|2149|2902|2903|2904|4318|5468|5743|6197|7124|114548	ADORA2A|CASP1|F2R|GRIN1|GRIN2A|GRIN2B|MMP9|PPARG|PTGS2|RPS6KA3|TNF|NLRP3	-6.08097	0	92	0	0	-8.16698	9.089125683
1	-7.37235	GO:0010952	M1	1	0	GO Biological Processes	19	positive regulation of peptidase activity	-7.37235	9.089126	8.955473	30242	200	183	11	6.010929	1.757049	351|834|2149|2335|2902|2903|2904|5468|7124|9475|114548	APP|CASP1|F2R|FN1|GRIN1|GRIN2A|GRIN2B|PPARG|TNF|ROCK2|NLRP3	-5.8932	0	92	0	0	-8.16698	9.089125683
1	-7.09191	GO:0045862	M1	1	0	GO Biological Processes	19	positive regulation of proteolysis	-7.09191	6.15318	7.845435	30242	376	183	14	7.650273	1.964856	150|351|834|1453|2149|2335|2902|2903|2904|3066|5468|7124|9475|114548	ADRA2A|APP|CASP1|CSNK1D|F2R|FN1|GRIN1|GRIN2A|GRIN2B|HDAC2|PPARG|TNF|ROCK2|NLRP3	-5.64566	0	92	0	0	-8.16698	9.089125683
1	-6.76561	GO:0052547	M1	1	0	GO Biological Processes	19	regulation of peptidase activity	-6.76561	5.342354	7.354834	30242	464	183	15	8.196721	2.027793	135|351|834|2149|2335|2902|2903|2904|4318|5468|5743|6197|7124|9475|114548	ADORA2A|APP|CASP1|F2R|FN1|GRIN1|GRIN2A|GRIN2B|MMP9|PPARG|PTGS2|RPS6KA3|TNF|ROCK2|NLRP3	-5.3521	0	92	0	0	-8.16698	9.089125683
1	-6.32538	GO:0052548	M1	1	0	GO Biological Processes	19	regulation of endopeptidase activity	-6.32538	5.318611	7.078902	30242	435	183	14	7.650273	1.964856	135|351|834|2149|2902|2903|2904|4318|5468|5743|6197|7124|9475|114548	ADORA2A|APP|CASP1|F2R|GRIN1|GRIN2A|GRIN2B|MMP9|PPARG|PTGS2|RPS6KA3|TNF|ROCK2|NLRP3	-4.94894	0	92	0	0	-8.16698	9.089125683
1	-5.74543	GO:0010950	M1	1	0	GO Biological Processes	19	positive regulation of endopeptidase activity	-5.74543	8.172041	7.57217	30242	182	183	9	4.918033	1.598524	834|2149|2902|2903|2904|5468|7124|9475|114548	CASP1|F2R|GRIN1|GRIN2A|GRIN2B|PPARG|TNF|ROCK2|NLRP3	-4.4216	0	92	0	0	-8.16698	9.089125683
1	-5.38734	GO:2001056	M1	1	0	GO Biological Processes	19	positive regulation of cysteine-type endopeptidase activity	-5.38734	8.755329	7.454298	30242	151	183	8	4.371585	1.511428	834|2149|2902|2903|2904|5468|7124|114548	CASP1|F2R|GRIN1|GRIN2A|GRIN2B|PPARG|TNF|NLRP3	-4.10252	0	92	0	0	-8.16698	9.089125683
1	-5.20385	GO:0043281	M1	1	0	GO Biological Processes	19	regulation of cysteine-type endopeptidase activity involved in apoptotic process	-5.20385	7.01562	6.858151	30242	212	183	9	4.918033	1.598524	135|834|2149|4318|5468|5743|6197|7124|114548	ADORA2A|CASP1|F2R|MMP9|PPARG|PTGS2|RPS6KA3|TNF|NLRP3	-3.94638	0	92	0	0	-8.16698	9.089125683
1	-2.89337	GO:0043280	M1	1	0	GO Biological Processes	19	positive regulation of cysteine-type endopeptidase activity involved in apoptotic process	-2.89337	6.259728	4.72532	30242	132	183	5	2.73224	1.205087	834|2149|5468|7124|114548	CASP1|F2R|PPARG|TNF|NLRP3	-1.90097	0	92	0	0	-8.16698	9.089125683
1	-2.80379	GO:0006919	M1	1	0	GO Biological Processes	19	activation of cysteine-type endopeptidase activity involved in apoptotic process	-2.80379	8.061309	4.995889	30242	82	183	4	2.185792	1.080886	834|2149|5468|7124	CASP1|F2R|PPARG|TNF	-1.82068	0	92	0	0	-8.16698	9.089125683
1	-8.13796	GO:0050951	M1	1	0	GO Biological Processes	19	sensory perception of temperature stimulus	-8.13796	39.66164	15.08902	30242	25	183	6	3.278689	1.316393	134|3356|4914|4986|7442|8989	ADORA1|HTR2A|NTRK1|OPRK1|TRPV1|TRPA1	-6.59131	0	93	1	1	-8.13796	47.21623731
1	-8.13796	GO:0062149	M1	1	0	GO Biological	19	detection of stimulus	-8.13796	39.66164	15.08902	30242	25	183	6	3.278689	1.316393	134|3356|4914|7442|89	ADORA1|HTR2A|NT	-6.59131	0	93	0	0	-8.13796	47.21623731

						Processes		involved in sensory perception of pain											89|23621	RK1|TRPV1|TRPA1|BACE1							
1	-5.90705	GO:0050966	M1	1	0	GO Biological Processes	19	detection of mechanical stimulus involved in sensory perception of pain	-5.90705	47.21624	13.49554	30242	14	183	4	2.185792	1.080886	3356|4914|8989|23621	HTR2A|NTRK1|TRPA1|BACE1	-4.56779	0	93	0	0	-8.13796	47.21623731
1	-5.65153	GO:0050965	M1	1	0	GO Biological Processes	19	detection of temperature stimulus involved in sensory perception of pain	-5.65153	41.31421	12.58532	30242	16	183	4	2.185792	1.080886	134|3356|4914|7442	ADORA1|HTR2A|NTRK1|TRPV1	-4.33864	0	93	0	0	-8.13796	47.21623731
1	-5.32939	GO:0050961	M1	1	0	GO Biological Processes	19	detection of temperature stimulus involved in sensory perception	-5.32939	34.79091	11.49593	30242	19	183	4	2.185792	1.080886	134|3356|4914|7442	ADORA1|HTR2A|NTRK1|TRPV1	-4.05665	0	93	0	0	-8.13796	47.21623731
1	-4.91689	GO:0050954	M1	1	0	GO Biological Processes	19	sensory perception of mechanical stimulus	-4.91689	7.554598	6.785102	30242	175	183	8	4.371585	1.511428	836|2558|3356|3383|4914|7442|8989|23621	CASP3|GABRA5|HTR2A|ICAM1|NTRK1|TRPV1|TRPA1|BACE1	-3.69403	0	93	0	0	-8.13796	47.21623731
1	-4.90168	GO:0016048	M1	1	0	GO Biological Processes	19	detection of temperature stimulus	-4.90168	27.54281	10.14976	30242	24	183	4	2.185792	1.080886	134|3356|4914|7442	ADORA1|HTR2A|NTRK1|TRPV1	-3.68342	0	93	0	0	-8.13796	47.21623731
1	-4.33459	GO:0050974	M1	1	0	GO Biological Processes	19	detection of mechanical stimulus involved in sensory perception	-4.33459	20.03113	8.534729	30242	33	183	4	2.185792	1.080886	3356|4914|8989|23621	HTR2A|NTRK1|TRPA1|BACE1	-3.18937	0	93	0	0	-8.13796	47.21623731
1	-3.74931	GO:0009581	M1	1	0	GO Biological Processes	19	detection of external stimulus	-3.74931	7.344748	5.764795	30242	135	183	6	3.278689	1.316393	134|3356|4914|7442|8989|23621	ADORA1|HTR2A|NTRK1|TRPV1|TRPA1|BACE1	-2.66793	0	93	0	0	-8.13796	47.21623731
1	-3.69744	GO:0009582	M1	1	0	GO Biological Processes	19	detection of abiotic stimulus	-3.69744	7.18508	5.682103	30242	138	183	6	3.278689	1.316393	134|3356|4914|7442|8989|23621	ADORA1|HTR2A|NTRK1|TRPV1|TRPA1|BACE1	-2.62334	0	93	0	0	-8.13796	47.21623731
1	-3.58562	GO:0050982	M1	1	0	GO Biological Processes	19	detection of mechanical stimulus	-3.58562	12.96132	6.670552	30242	51	183	4	2.185792	1.080886	3356|4914|8989|23621	HTR2A|NTRK1|TRPA1|BACE1	-2.5219	0	93	0	0	-8.13796	47.21623731
1	-8.02208	GO:0001973	M1	1	0	GO Biological Processes	19	G protein-coupled adenosine receptor signaling pathway	-8.02208	63.56032	17.60334	30242	13	183	5	2.73224	1.205087	100|134|135|136|140	ADA|ADORA1|ADORA2A|ADORA2B|ADORA3	-6.48507	0	94	1	1	-8.02208	99.15409836
1	-8.02208	GO:0035588	M1	1	0	GO Biological Processes	19	G protein-coupled purinergic receptor signaling pathway	-8.02208	63.56032	17.60334	30242	13	183	5	2.73224	1.205087	100|134|135|136|140	ADA|ADORA1|ADORA2A|ADORA2B|ADORA3	-6.48507	0	94	0	0	-8.02208	99.15409836
1	-7.27712	GO:0035590	M1	1	0	GO Biological Processes	19	purinergic nucleotide receptor signaling pathway	-7.27712	29.16297	12.82016	30242	34	183	6	3.278689	1.316393	100|134|135|136|140|5024	ADA|ADORA1|ADORA2A|ADORA2B|ADORA3|P2RX3	-5.81673	0	94	0	0	-8.02208	99.15409836
1	-5.66547	GO:0042321	M1	1	0	GO Biological Processes	19	negative regulation of circadian sleep/wake cycle, sleep	-5.66547	99.1541	17.12618	30242	5	183	3	1.639344	0.938686	100|134|1813	ADA|ADORA1|DRD2	-4.35145	0	94	0	0	-8.02208	99.15409836
1	-4.74896	GO:0070255	M1	1	0	GO Biological Processes	19	regulation of mucus secretion	-4.74896	55.08561	12.6619	30242	9	183	3	1.639344	0.938686	100|134|1956	ADA|ADORA1|EGFR	-3.54861	0	94	0	0	-8.02208	99.15409836
1	-4.02686	GO:0042754	M1	1	0	GO Biological Processes	19	negative regulation of circadian rhythm	-4.02686	33.05137	9.68793	30242	15	183	3	1.639344	0.938686	100|134|1813	ADA|ADORA1|DRD2	-2.91294	0	94	0	0	-8.02208	99.15409836
1	-4.02686	GO:0070254	M1	1	0	GO Biological Processes	19	mucus secretion	-4.02686	33.05137	9.68793	30242	15	183	3	1.639344	0.938686	100|134|1956	ADA|ADORA1|EGFR	-2.91294	0	94	0	0	-8.02208	99.15409836
1	-3.85624	GO:0048521	M1	1	0	GO Biological Processes	19	negative regulation of behavior	-3.85624	29.16297	9.062669	30242	17	183	3	1.639344	0.938686	100|134|1813	ADA|ADORA1|DRD2	-2.76097	0	94	0	0	-8.02208	99.15409836
1	-3.68815	GO:0002686	M1	1	0	GO Biological	19	negative regulation of	-3.68815	13.7714	6.909319	30242	48	183	4	2.185792	1.080886	100|134|5734|6347	ADA|ADORA1|PTGE	-2.6157	0	94	0	0	-8.02208	99.15409836

<table>
<tr><td></td><td></td><td></td><td></td><td></td><td></td><td>Processes</td><td></td><td>leukocyte migration</td><td></td><td></td><td></td><td></td><td></td><td></td><td></td><td></td><td></td><td></td><td>R4|CCL2</td><td></td><td></td><td></td><td></td><td></td><td></td><td></td></tr>
<tr><td>1</td><td>-8.00458</td><td>GO:0045933</td><td>M1</td><td>1</td><td>0</td><td>GO Biological Processes</td><td>19</td><td>positive regulation of muscle contraction</td><td>-8.00458</td><td>25.70662</td><td>12.94118</td><td>30242</td><td>45</td><td>183</td><td>7</td><td>3.825137</td><td>1.417845</td><td>100|148|151|1131|2149|5743|59341</td><td>ADA|ADRA1A|ADRA2B|CHRM3|F2R|PTGS2|TRPV4</td><td>-6.46946</td><td>0</td><td>95</td><td>1</td><td>1</td><td>-8.00458</td><td>31.98519302</td></tr>
<tr><td>1</td><td>-7.53222</td><td>GO:0045987</td><td>M1</td><td>1</td><td>0</td><td>GO Biological Processes</td><td>19</td><td>positive regulation of smooth muscle contraction</td><td>-7.53222</td><td>31.98519</td><td>13.46756</td><td>30242</td><td>31</td><td>183</td><td>6</td><td>3.278689</td><td>1.316393</td><td>100|148|151|1131|2149|5743</td><td>ADA|ADRA1A|ADRA2B|CHRM3|F2R|PTGS2</td><td>-6.04296</td><td>0</td><td>95</td><td>0</td><td>0</td><td>-8.00458</td><td>31.98519302</td></tr>
<tr><td>1</td><td>-8.00327</td><td>GO:0034330</td><td>M1</td><td>1</td><td>0</td><td>GO Biological Processes</td><td>19</td><td>cell junction organization</td><td>-8.00327</td><td>4.74876</td><td>7.806911</td><td>30242</td><td>696</td><td>183</td><td>20</td><td>10.92896</td><td>2.306387</td><td>351|596|1636|1812|1813|2149|2335|2554|2566|2904|2915|3551|3643|4914|4915|7040|7046|7124|9475|59341</td><td>APP|BCL2|ACE|DRD1|DRD2|F2R|FN1|GABRA1|GABRG2|GRIN2B|GRM5|IKBKB|INSR|NTRK1|NTRK2|TGFB1|TGFBR1|TNF|ROCK2|TRPV4</td><td>-6.4691</td><td>0</td><td>96</td><td>1</td><td>1</td><td>-8.00327</td><td>15.59026704</td></tr>
<tr><td>1</td><td>-6.4955</td><td>GO:0034329</td><td>M1</td><td>1</td><td>0</td><td>GO Biological Processes</td><td>19</td><td>cell junction assembly</td><td>-6.4955</td><td>5.495477</td><td>7.247564</td><td>30242</td><td>421</td><td>183</td><td>14</td><td>7.650273</td><td>1.964856</td><td>351|596|1636|1812|1813|2335|2554|2566|3551|4914|4915|7124|9475|59341</td><td>APP|BCL2|ACE|DRD1|DRD2|FN1|GABRA1|GABRG2|IKBKB|NTRK1|NTRK2|TNF|ROCK2|TRPV4</td><td>-5.10773</td><td>0</td><td>96</td><td>0</td><td>0</td><td>-8.00327</td><td>15.59026704</td></tr>
<tr><td>1</td><td>-4.91278</td><td>GO:0050808</td><td>M1</td><td>1</td><td>0</td><td>GO Biological Processes</td><td>19</td><td>synapse organization</td><td>-4.91278</td><td>4.655122</td><td>5.928138</td><td>30242</td><td>426</td><td>183</td><td>12</td><td>6.557377</td><td>1.829835</td><td>351|1812|1813|2149|2554|2566|2904|2915|3643|4914|4915|7124</td><td>APP|DRD1|DRD2|F2R|GABRA1|GABRG2|GRIN2B|GRM5|INSR|NTRK1|NTRK2|TNF</td><td>-3.69038</td><td>0</td><td>96</td><td>0</td><td>0</td><td>-8.00327</td><td>15.59026704</td></tr>
<tr><td>1</td><td>-4.75899</td><td>GO:1990090</td><td>M1</td><td>1</td><td>0</td><td>GO Biological Processes</td><td>19</td><td>cellular response to nerve growth factor stimulus</td><td>-4.75899</td><td>15.59027</td><td>8.294938</td><td>30242</td><td>53</td><td>183</td><td>5</td><td>2.73224</td><td>1.205087</td><td>351|1453|4914|4915|7442</td><td>APP|CSNK1D|NTRK1|NTRK2|TRPV1</td><td>-3.55409</td><td>0</td><td>96</td><td>0</td><td>0</td><td>-8.00327</td><td>15.59026704</td></tr>
<tr><td>1</td><td>-4.64115</td><td>GO:1990089</td><td>M1</td><td>1</td><td>0</td><td>GO Biological Processes</td><td>19</td><td>response to nerve growth factor</td><td>-4.64115</td><td>14.75507</td><td>8.038787</td><td>30242</td><td>56</td><td>183</td><td>5</td><td>2.73224</td><td>1.205087</td><td>351|1453|4914|4915|7442</td><td>APP|CSNK1D|NTRK1|NTRK2|TRPV1</td><td>-3.45334</td><td>0</td><td>96</td><td>0</td><td>0</td><td>-8.00327</td><td>15.59026704</td></tr>
<tr><td>1</td><td>-3.94088</td><td>GO:0007416</td><td>M1</td><td>1</td><td>0</td><td>GO Biological Processes</td><td>19</td><td>synapse assembly</td><td>-3.94088</td><td>6.462558</td><td>5.719298</td><td>30242</td><td>179</td><td>183</td><td>7</td><td>3.825137</td><td>1.417845</td><td>351|1812|1813|2554|2566|4914|4915</td><td>APP|DRD1|DRD2|GABRA1|GABRG2|NTRK1|NTRK2</td><td>-2.83536</td><td>0</td><td>96</td><td>0</td><td>0</td><td>-8.00327</td><td>15.59026704</td></tr>
<tr><td>1</td><td>-3.85519</td><td>GO:0061564</td><td>M1</td><td>1</td><td>0</td><td>GO Biological Processes</td><td>19</td><td>axon development</td><td>-3.85519</td><td>3.892559</td><td>4.915313</td><td>30242</td><td>467</td><td>183</td><td>11</td><td>6.010929</td><td>1.757049</td><td>351|596|836|1268|1813|2050|2263|2335|4914|4915|5979</td><td>APP|BCL2|CASP3|CNR1|DRD2|EPHB4|FGFR2|FN1|NTRK1|NTRK2|RET</td><td>-2.76026</td><td>0</td><td>96</td><td>0</td><td>0</td><td>-8.00327</td><td>15.59026704</td></tr>
<tr><td>1</td><td>-3.59188</td><td>GO:1901888</td><td>M1</td><td>1</td><td>0</td><td>GO Biological Processes</td><td>19</td><td>regulation of cell junction assembly</td><td>-3.59188</td><td>5.670578</td><td>5.222597</td><td>30242</td><td>204</td><td>183</td><td>7</td><td>3.825137</td><td>1.417845</td><td>351|1636|3551|4914|4915|7124|9475</td><td>APP|ACE|IKBKB|NTRK1|NTRK2|TNF|ROCK2</td><td>-2.52655</td><td>0</td><td>96</td><td>0</td><td>0</td><td>-8.00327</td><td>15.59026704</td></tr>
<tr><td>1</td><td>-3.1711</td><td>GO:0120035</td><td>M1</td><td>1</td><td>0</td><td>GO Biological Processes</td><td>19</td><td>regulation of plasma membrane bounded cell projection organization</td><td>-3.1711</td><td>3.032235</td><td>4.0996</td><td>30242</td><td>654</td><td>183</td><td>12</td><td>6.557377</td><td>1.829835</td><td>1268|2335|2904|3066|3383|4914|4915|5290|5979|7046|59341|225689</td><td>CNR1|FN1|GRIN2B|HDAC2|ICAM1|NTRK1|NTRK2|PIK3CA|RET|TGFBR1|TRPV4|MAPK15</td><td>-2.15005</td><td>0</td><td>96</td><td>0</td><td>0</td><td>-8.00327</td><td>15.59026704</td></tr>
<tr><td>1</td><td>-3.06907</td><td>GO:0031344</td><td>M1</td><td>1</td><td>0</td><td>GO Biological Processes</td><td>19</td><td>regulation of cell projection organization</td><td>-3.06907</td><td>2.951015</td><td>3.990763</td><td>30242</td><td>672</td><td>183</td><td>12</td><td>6.557377</td><td>1.829835</td><td>1268|2335|2904|3066|3383|4914|4915|5290|5979|7046|59341|225689</td><td>CNR1|FN1|GRIN2B|HDAC2|ICAM1|NTRK1|NTRK2|PIK3CA|RET|TGFBR1|TRPV4|MAPK15</td><td>-2.05863</td><td>0</td><td>96</td><td>0</td><td>0</td><td>-8.00327</td><td>15.59026704</td></tr>
<tr><td>1</td><td>-2.97339</td><td>GO:0007409</td><td>M1</td><td>1</td><td>0</td><td>GO Biological Processes</td><td>19</td><td>axonogenesis</td><td>-2.97339</td><td>3.558161</td><td>4.109316</td><td>30242</td><td>418</td><td>183</td><td>9</td><td>4.918033</td><td>1.598524</td><td>351|596|1813|2050|2263|2335|4914|4915|5979</td><td>APP|BCL2|DRD2|EPHB4|FGFR2|FN1|NTRK1|NTRK2|RET</td><td>-1.97275</td><td>0</td><td>96</td><td>0</td><td>0</td><td>-8.00327</td><td>15.59026704</td></tr>
</table>

<table>
<tr><td>1</td><td>-2.94949</td><td>GO:0000904</td><td>M1</td><td>1</td><td>0</td><td>GO Biological Processes</td><td>19</td><td>cell morphogenesis involved in differentiation</td><td>-2.94949</td><td>2.857467</td><td>3.862541</td><td>30242</td><td>694</td><td>183</td><td>12</td><td>6.557377</td><td>1.829835</td><td>351|367|596|836|1813|2050|2263|2335|4914|4915|5591|5979</td><td>APP|AR|BCL2|CASP3|DRD2|EPHB4|FGFR2|FN1|NTRK1|NTRK2|PRKDC|RET</td><td>-1.95023</td><td>0</td><td>96</td><td>0</td><td>0</td><td>-8.00327</td><td>15.59026704</td></tr>
<tr><td>1</td><td>-2.87173</td><td>GO:0038179</td><td>M1</td><td>1</td><td>0</td><td>GO Biological Processes</td><td>19</td><td>neurotrophin signaling pathway</td><td>-2.87173</td><td>13.7714</td><td>5.982457</td><td>30242</td><td>36</td><td>183</td><td>3</td><td>1.639344</td><td>0.938686</td><td>836|4914|4915</td><td>CASP3|NTRK1|NTRK2</td><td>-1.88231</td><td>0</td><td>96</td><td>0</td><td>0</td><td>-8.00327</td><td>15.59026704</td></tr>
<tr><td>1</td><td>-2.8609</td><td>GO:0048812</td><td>M1</td><td>1</td><td>0</td><td>GO Biological Processes</td><td>19</td><td>neuron projection morphogenesis</td><td>-2.8609</td><td>2.955813</td><td>3.823468</td><td>30242</td><td>615</td><td>183</td><td>11</td><td>6.010929</td><td>1.757049</td><td>135|351|596|1813|1956|2050|2263|2335|4914|4915|5979</td><td>ADORA2A|APP|BCL2|DRD2|EGFR|EPHB4|FGFR2|FN1|NTRK1|NTRK2|RET</td><td>-1.87201</td><td>0</td><td>96</td><td>0</td><td>0</td><td>-8.00327</td><td>15.59026704</td></tr>
<tr><td>1</td><td>-2.77854</td><td>GO:0120039</td><td>M1</td><td>1</td><td>0</td><td>GO Biological Processes</td><td>19</td><td>plasma membrane bounded cell projection morphogenesis</td><td>-2.77854</td><td>2.885437</td><td>3.731511</td><td>30242</td><td>630</td><td>183</td><td>11</td><td>6.010929</td><td>1.757049</td><td>135|351|596|1813|1956|2050|2263|2335|4914|4915|5979</td><td>ADORA2A|APP|BCL2|DRD2|EGFR|EPHB4|FGFR2|FN1|NTRK1|NTRK2|RET</td><td>-1.79741</td><td>0</td><td>96</td><td>0</td><td>0</td><td>-8.00327</td><td>15.59026704</td></tr>
<tr><td>1</td><td>-2.75705</td><td>GO:0048858</td><td>M1</td><td>1</td><td>0</td><td>GO Biological Processes</td><td>19</td><td>cell projection morphogenesis</td><td>-2.75705</td><td>2.867232</td><td>3.707445</td><td>30242</td><td>634</td><td>183</td><td>11</td><td>6.010929</td><td>1.757049</td><td>135|351|596|1813|1956|2050|2263|2335|4914|4915|5979</td><td>ADORA2A|APP|BCL2|DRD2|EGFR|EPHB4|FGFR2|FN1|NTRK1|NTRK2|RET</td><td>-1.77934</td><td>0</td><td>96</td><td>0</td><td>0</td><td>-8.00327</td><td>15.59026704</td></tr>
<tr><td>1</td><td>-2.73148</td><td>GO:0050807</td><td>M1</td><td>1</td><td>0</td><td>GO Biological Processes</td><td>19</td><td>regulation of synapse organization</td><td>-2.73148</td><td>4.699246</td><td>4.207324</td><td>30242</td><td>211</td><td>183</td><td>6</td><td>3.278689</td><td>1.316393</td><td>351|1813|2904|4914|4915|7124</td><td>APP|DRD2|GRIN2B|NTRK1|NTRK2|TNF</td><td>-1.75717</td><td>0</td><td>96</td><td>0</td><td>0</td><td>-8.00327</td><td>15.59026704</td></tr>
<tr><td>1</td><td>-2.6969</td><td>GO:0032989</td><td>M1</td><td>1</td><td>0</td><td>GO Biological Processes</td><td>19</td><td>cellular component morphogenesis</td><td>-2.6969</td><td>2.665433</td><td>3.588836</td><td>30242</td><td>744</td><td>183</td><td>12</td><td>6.557377</td><td>1.829835</td><td>135|351|596|1813|1956|2050|2263|2335|4914|4915|5159|5979</td><td>ADORA2A|APP|BCL2|DRD2|EGFR|EPHB4|FGFR2|FN1|NTRK1|NTRK2|PDGFRB|RET</td><td>-1.72467</td><td>0</td><td>96</td><td>0</td><td>0</td><td>-8.00327</td><td>15.59026704</td></tr>
<tr><td>1</td><td>-2.66268</td><td>GO:0032990</td><td>M1</td><td>1</td><td>0</td><td>GO Biological Processes</td><td>19</td><td>cell part morphogenesis</td><td>-2.66268</td><td>2.788075</td><td>3.601418</td><td>30242</td><td>652</td><td>183</td><td>11</td><td>6.010929</td><td>1.757049</td><td>135|351|596|1813|1956|2050|2263|2335|4914|4915|5979</td><td>ADORA2A|APP|BCL2|DRD2|EGFR|EPHB4|FGFR2|FN1|NTRK1|NTRK2|RET</td><td>-1.69458</td><td>0</td><td>96</td><td>0</td><td>0</td><td>-8.00327</td><td>15.59026704</td></tr>
<tr><td>1</td><td>-2.66044</td><td>GO:0050803</td><td>M1</td><td>1</td><td>0</td><td>GO Biological Processes</td><td>19</td><td>regulation of synapse structure or activity</td><td>-2.66044</td><td>4.548353</td><td>4.102581</td><td>30242</td><td>218</td><td>183</td><td>6</td><td>3.278689</td><td>1.316393</td><td>351|1813|2904|4914|4915|7124</td><td>APP|DRD2|GRIN2B|NTRK1|NTRK2|TNF</td><td>-1.69311</td><td>0</td><td>96</td><td>0</td><td>0</td><td>-8.00327</td><td>15.59026704</td></tr>
<tr><td>1</td><td>-2.23032</td><td>GO:0031346</td><td>M1</td><td>1</td><td>0</td><td>GO Biological Processes</td><td>19</td><td>positive regulation of cell projection organization</td><td>-2.23032</td><td>3.277048</td><td>3.357686</td><td>30242</td><td>353</td><td>183</td><td>7</td><td>3.825137</td><td>1.417845</td><td>1268|2335|4914|4915|5290|5979|7046</td><td>CNR1|FN1|NTRK1|NTRK2|PIK3CA|RET|TGFBR1</td><td>-1.30221</td><td>0</td><td>96</td><td>0</td><td>0</td><td>-8.00327</td><td>15.59026704</td></tr>
<tr><td>1</td><td>-2.21418</td><td>GO:0048667</td><td>M1</td><td>1</td><td>0</td><td>GO Biological Processes</td><td>19</td><td>cell morphogenesis involved in neuron differentiation</td><td>-2.21418</td><td>2.734028</td><td>3.184406</td><td>30242</td><td>544</td><td>183</td><td>9</td><td>4.918033</td><td>1.598524</td><td>351|596|1813|2050|2263|2335|4914|4915|5979</td><td>APP|BCL2|DRD2|EPHB4|FGFR2|FN1|NTRK1|NTRK2|RET</td><td>-1.28748</td><td>0</td><td>96</td><td>0</td><td>0</td><td>-8.00327</td><td>15.59026704</td></tr>
<tr><td>1</td><td>-2.10448</td><td>GO:0042490</td><td>M1</td><td>1</td><td>0</td><td>GO Biological Processes</td><td>19</td><td>mechanoreceptor differentiation</td><td>-2.10448</td><td>7.39956</td><td>4.091666</td><td>30242</td><td>67</td><td>183</td><td>3</td><td>1.639344</td><td>0.938686</td><td>2558|4914|4915</td><td>GABRA5|NTRK1|NTRK2</td><td>-1.18457</td><td>0</td><td>96</td><td>0</td><td>0</td><td>-8.00327</td><td>15.59026704</td></tr>
<tr><td>1</td><td>-7.81668</td><td>GO:0007205</td><td>M1</td><td>1</td><td>0</td><td>GO Biological Processes</td><td>19</td><td>protein kinase C-activating G protein-coupled receptor signaling pathway</td><td>-7.81668</td><td>35.41218</td><td>14.21423</td><td>30242</td><td>28</td><td>183</td><td>6</td><td>3.278689</td><td>1.316393</td><td>135|1128|2149|3351|3357|3558</td><td>ADORA2A|CHRM1|F2R|HTR1B|HTR2B|IL2</td><td>-6.29922</td><td>0</td><td>97</td><td>1</td><td>1</td><td>-7.81668</td><td>99.15409836</td></tr>
<tr><td>1</td><td>-5.66547</td><td>GO:0071502</td><td>M1</td><td>1</td><td>0</td><td>GO Biological Processes</td><td>19</td><td>cellular response to temperature stimulus</td><td>-5.66547</td><td>99.1541</td><td>17.12618</td><td>30242</td><td>5</td><td>183</td><td>3</td><td>1.639344</td><td>0.938686</td><td>3351|3357|7442</td><td>HTR1B|HTR2B|TRPV1</td><td>-4.35145</td><td>0</td><td>97</td><td>0</td><td>0</td><td>-7.81668</td><td>99.15409836</td></tr>
<tr><td>1</td><td>-7.80218</td><td>GO:0046777</td><td>M1</td><td>1</td><td>0</td><td>GO Biological Processes</td><td>19</td><td>protein autophosphorylation</td><td>-7.80218</td><td>8.736044</td><td>9.128492</td><td>30242</td><td>227</td><td>183</td><td>12</td><td>6.557377</td><td>1.829835</td><td>472|1636|1956|2050|2263|3643|4914|4915|5159|5582|6793|225689</td><td>ATM|ACE|EGFR|EPHB4|FGFR2|INSR|NTRK1|NTRK2|PDGFRB|PRKCG|STK10|MAPK15</td><td>-6.28562</td><td>0</td><td>98</td><td>1</td><td>1</td><td>-7.80218</td><td>23.60811866</td></tr>
<tr><td>1</td><td>-3.57263</td><td>GO:0038083</td><td>M1</td><td>1</td><td>0</td><td>GO Biological Processes</td><td>19</td><td>peptidyl-tyrosine autophosphorylation</td><td>-3.57263</td><td>23.60812</td><td>8.086415</td><td>30242</td><td>21</td><td>183</td><td>3</td><td>1.639344</td><td>0.938686</td><td>1636|1956|4914</td><td>ACE|EGFR|NTRK1</td><td>-2.51178</td><td>0</td><td>98</td><td>0</td><td>0</td><td>-7.80218</td><td>23.60811866</td></tr>
</table>

<table>
<tr><td></td><td></td><td></td><td></td><td></td><td></td><td></td><td></td><td></td><td></td><td></td><td></td><td></td><td></td><td></td><td></td><td></td><td></td><td></td><td></td><td></td><td></td><td></td><td></td><td></td><td></td><td></td></tr>
<tr><td>1</td><td>-7.7363</td><td>GO:0014075</td><td>M1</td><td>1</td><td>0</td><td>GO Biological Processes</td><td>19</td><td>response to amine</td><td>-7.7363</td><td>23.60812</td><td>12.35793</td><td>30242</td><td>49</td><td>183</td><td>7</td><td>3.825137</td><td>1.417845</td><td>135|1728|1813|1815|2903|3066|3383</td><td>ADORA2A|NQO1|DRD2|DRD4|GRIN2A|HDAC2|ICAM1</td><td>-6.22784</td><td>0</td><td>99</td><td>1</td><td>1</td><td>-7.7363</td><td>82.6284153</td></tr>
<tr><td>1</td><td>-7.35926</td><td>GO:0001975</td><td>M1</td><td>1</td><td>0</td><td>GO Biological Processes</td><td>19</td><td>response to amphetamine</td><td>-7.35926</td><td>30.0467</td><td>13.02632</td><td>30242</td><td>33</td><td>183</td><td>6</td><td>3.278689</td><td>1.316393</td><td>135|1813|1815|2903|3066|3383</td><td>ADORA2A|DRD2|DRD4|GRIN2A|HDAC2|ICAM1</td><td>-5.88178</td><td>0</td><td>99</td><td>0</td><td>0</td><td>-7.7363</td><td>82.6284153</td></tr>
<tr><td>1</td><td>-7.05003</td><td>GO:0048149</td><td>M1</td><td>1</td><td>0</td><td>GO Biological Processes</td><td>19</td><td>behavioral response to ethanol</td><td>-7.05003</td><td>82.62842</td><td>18.01669</td><td>30242</td><td>8</td><td>183</td><td>4</td><td>2.185792</td><td>1.080886</td><td>1813|1815|3066|4988</td><td>DRD2|DRD4|HDAC2|OPRM1</td><td>-5.61068</td><td>0</td><td>99</td><td>0</td><td>0</td><td>-7.7363</td><td>82.6284153</td></tr>
<tr><td>1</td><td>-4.9788</td><td>GO:0051590</td><td>M1</td><td>1</td><td>0</td><td>GO Biological Processes</td><td>19</td><td>positive regulation of neurotransmitter transport</td><td>-4.9788</td><td>28.74032</td><td>10.38417</td><td>30242</td><td>23</td><td>183</td><td>4</td><td>2.185792</td><td>1.080886</td><td>135|1268|1813|1815</td><td>ADORA2A|CNR1|DRD2|DRD4</td><td>-3.74566</td><td>0</td><td>99</td><td>0</td><td>0</td><td>-7.7363</td><td>82.6284153</td></tr>
<tr><td>1</td><td>-4.13489</td><td>GO:0051954</td><td>M1</td><td>1</td><td>0</td><td>GO Biological Processes</td><td>19</td><td>positive regulation of amine transport</td><td>-4.13489</td><td>17.8656</td><td>8.009394</td><td>30242</td><td>37</td><td>183</td><td>4</td><td>2.185792</td><td>1.080886</td><td>135|1813|1815|4986</td><td>ADORA2A|DRD2|DRD4|OPRK1</td><td>-3.00894</td><td>0</td><td>99</td><td>0</td><td>0</td><td>-7.7363</td><td>82.6284153</td></tr>
<tr><td>1</td><td>-3.77899</td><td>GO:0031000</td><td>M1</td><td>1</td><td>0</td><td>GO Biological Processes</td><td>19</td><td>response to caffeine</td><td>-3.77899</td><td>27.54281</td><td>8.789081</td><td>30242</td><td>18</td><td>183</td><td>3</td><td>1.639344</td><td>0.938686</td><td>135|1723|3066</td><td>ADORA2A|DHODH|HDAC2</td><td>-2.69427</td><td>0</td><td>99</td><td>0</td><td>0</td><td>-7.7363</td><td>82.6284153</td></tr>
<tr><td>1</td><td>-3.24195</td><td>GO:0048520</td><td>M1</td><td>1</td><td>0</td><td>GO Biological Processes</td><td>19</td><td>positive regulation of behavior</td><td>-3.24195</td><td>18.36187</td><td>7.04212</td><td>30242</td><td>27</td><td>183</td><td>3</td><td>1.639344</td><td>0.938686</td><td>135|3066|4986</td><td>ADORA2A|HDAC2|OPRK1</td><td>-2.21358</td><td>0</td><td>99</td><td>0</td><td>0</td><td>-7.7363</td><td>82.6284153</td></tr>
<tr><td>1</td><td>-7.64522</td><td>GO:0043266</td><td>M1</td><td>1</td><td>0</td><td>GO Biological Processes</td><td>19</td><td>regulation of potassium ion transport</td><td>-7.64522</td><td>13.64506</td><td>10.31928</td><td>30242</td><td>109</td><td>183</td><td>9</td><td>4.918033</td><td>1.598524</td><td>134|150|1812|1813|1814|3356|3757|4842|4986</td><td>ADORA1|ADRA2A|DRD1|DRD2|DRD3|HTR2A|KCNH2|NOS1|OPRK1</td><td>-6.14559</td><td>0</td><td>100</td><td>1</td><td>1</td><td>-7.64522</td><td>16.52568306</td></tr>
<tr><td>1</td><td>-6.55342</td><td>GO:0006813</td><td>M1</td><td>1</td><td>0</td><td>GO Biological Processes</td><td>19</td><td>potassium ion transport</td><td>-6.55342</td><td>7.542843</td><td>7.956893</td><td>30242</td><td>241</td><td>183</td><td>11</td><td>6.010929</td><td>1.757049</td><td>134|150|1812|1813|1814|3356|3757|3778|4842|4986|51305</td><td>ADORA1|ADRA2A|DRD1|DRD2|DRD3|HTR2A|KCNH2|KCNMA1|NOS1|OPRK1|KCNK9</td><td>-5.16039</td><td>0</td><td>100</td><td>0</td><td>0</td><td>-7.64522</td><td>16.52568306</td></tr>
<tr><td>1</td><td>-4.88437</td><td>GO:0043268</td><td>M1</td><td>1</td><td>0</td><td>GO Biological Processes</td><td>19</td><td>positive regulation of potassium ion transport</td><td>-4.88437</td><td>16.52568</td><td>8.572868</td><td>30242</td><td>50</td><td>183</td><td>5</td><td>2.73224</td><td>1.205087</td><td>134|150|1812|3757|4986</td><td>ADORA1|ADRA2A|DRD1|KCNH2|OPRK1</td><td>-3.66748</td><td>0</td><td>100</td><td>0</td><td>0</td><td>-7.64522</td><td>16.52568306</td></tr>
<tr><td>1</td><td>-2.77031</td><td>GO:0043267</td><td>M1</td><td>1</td><td>0</td><td>GO Biological Processes</td><td>19</td><td>negative regulation of potassium ion transport</td><td>-2.77031</td><td>12.71206</td><td>5.710538</td><td>30242</td><td>39</td><td>183</td><td>3</td><td>1.639344</td><td>0.938686</td><td>3356|3757|4842</td><td>HTR2A|KCNH2|NOS1</td><td>-1.79128</td><td>0</td><td>100</td><td>0</td><td>0</td><td>-7.64522</td><td>16.52568306</td></tr>
<tr><td>1</td><td>-7.60563</td><td>GO:0080135</td><td>M1</td><td>1</td><td>0</td><td>GO Biological Processes</td><td>19</td><td>regulation of cellular response to stress</td><td>-7.60563</td><td>4.735867</td><td>7.589144</td><td>30242</td><td>663</td><td>183</td><td>19</td><td>10.38251</td><td>2.254873</td><td>351|472|596|624|1956|3156|4221|4314|4986|4988|5582|5591|5743|7099|7124|7157|9475|10135|59341</td><td>APP|ATM|BCL2|BDKRB2|EGFR|HMGCR|MEN1|MMP3|OPRK1|OPRM1|PRKCG|PRKDC|PTGS2|TLR4|TNF|TP53|ROCK2|NAMPT|TRPV4</td><td>-6.11035</td><td>0</td><td>101</td><td>1</td><td>1</td><td>-7.60563</td><td>23.60811866</td></tr>
<tr><td>1</td><td>-7.00726</td><td>GO:0032757</td><td>M1</td><td>1</td><td>0</td><td>GO Biological Processes</td><td>19</td><td>positive regulation of interleukin-8 production</td><td>-7.00726</td><td>18.65803</td><td>10.85962</td><td>30242</td><td>62</td><td>183</td><td>7</td><td>3.825137</td><td>1.417845</td><td>1991|2149|3569|4843|5970|7099|7124</td><td>ELANE|F2R|IL6|NOS2|RELA|TLR4|TNF</td><td>-5.5715</td><td>0</td><td>101</td><td>0</td><td>0</td><td>-7.60563</td><td>23.60811866</td></tr>
<tr><td>1</td><td>-6.55342</td><td>GO:0051403</td><td>M1</td><td>1</td><td>0</td><td>GO Biological Processes</td><td>19</td><td>stress-activated MAPK cascade</td><td>-6.55342</td><td>7.542843</td><td>7.956893</td><td>30242</td><td>241</td><td>183</td><td>11</td><td>6.010929</td><td>1.757049</td><td>136|351|1956|3156|3551|4221|4986|5734|7099|7124|59341</td><td>ADORA2B|APP|EGFR|HMGCR|IKBKB|MEN1|OPRK1|PTGER4|TLR4|TNF|TRPV4</td><td>-5.16039</td><td>0</td><td>101</td><td>0</td><td>0</td><td>-7.60563</td><td>23.60811866</td></tr>
<tr><td>1</td><td>-6.51135</td><td>GO:0032732</td><td>M1</td><td>1</td><td>0</td><td>GO Biological Processes</td><td>19</td><td>positive regulation of interleukin-1 production</td><td>-6.51135</td><td>15.84655</td><td>9.909295</td><td>30242</td><td>73</td><td>183</td><td>7</td><td>3.825137</td><td>1.417845</td><td>351|834|3066|3569|7099|7124|114548</td><td>APP|CASP1|HDAC2|IL6|TLR4|TNF|NLRP3</td><td>-5.1229</td><td>0</td><td>101</td><td>0</td><td>0</td><td>-7.60563</td><td>23.60811866</td></tr>
<tr><td>1</td><td>-6.41226</td><td>GO:0031098</td><td>M1</td><td>1</td><td>0</td><td>GO Biological Processes</td><td>19</td><td>stress-activated protein kinase signaling cascade</td><td>-6.41226</td><td>7.300503</td><td>7.789351</td><td>30242</td><td>249</td><td>183</td><td>11</td><td>6.010929</td><td>1.757049</td><td>136|351|1956|3156|3551|4221|4986|5734|7099|7124|59341</td><td>ADORA2B|APP|EGFR|HMGCR|IKBKB|MEN1|OPRK1|PTGER4|TLR4|TNF|TRPV4</td><td>-5.02986</td><td>0</td><td>101</td><td>0</td><td>0</td><td>-7.60563</td><td>23.60811866</td></tr>
</table>

<table>
<tr><td>1</td><td>-5.90186</td><td>GO:0002274</td><td>M1</td><td>1</td><td>0</td><td>GO Biological Processes</td><td>19</td><td>myeloid leukocyte activation</td><td>-5.90186</td><td>7.344748</td><td>7.453467</td><td>30242</td><td>225</td><td>183</td><td>10</td><td>5.464481</td><td>1.680144</td><td>351|1268|3569|3576|5293|7099|7124|7442|10135|729230</td><td>APP|CNR1|IL6|CXCL8|PIK3CD|TLR4|TNF|TRPV1|NAMPT|CCR2</td><td>-4.56381</td><td>0</td><td>101</td><td>0</td><td>0</td><td>-7.60563</td><td>23.60811866</td></tr>
<tr><td>1</td><td>-5.89966</td><td>GO:0032612</td><td>M1</td><td>1</td><td>0</td><td>GO Biological Processes</td><td>19</td><td>interleukin-1 production</td><td>-5.89966</td><td>10.24849</td><td>8.213435</td><td>30242</td><td>129</td><td>183</td><td>8</td><td>4.371585</td><td>1.511428</td><td>351|834|2149|3066|3569|7099|7124|114548</td><td>APP|CASP1|F2R|HDAC2|IL6|TLR4|TNF|NLRP3</td><td>-4.56281</td><td>0</td><td>101</td><td>0</td><td>0</td><td>-7.60563</td><td>23.60811866</td></tr>
<tr><td>1</td><td>-5.89966</td><td>GO:0032652</td><td>M1</td><td>1</td><td>0</td><td>GO Biological Processes</td><td>19</td><td>regulation of interleukin-1 production</td><td>-5.89966</td><td>10.24849</td><td>8.213435</td><td>30242</td><td>129</td><td>183</td><td>8</td><td>4.371585</td><td>1.511428</td><td>351|834|2149|3066|3569|7099|7124|114548</td><td>APP|CASP1|F2R|HDAC2|IL6|TLR4|TNF|NLRP3</td><td>-4.56281</td><td>0</td><td>101</td><td>0</td><td>0</td><td>-7.60563</td><td>23.60811866</td></tr>
<tr><td>1</td><td>-5.79102</td><td>GO:0032755</td><td>M1</td><td>1</td><td>0</td><td>GO Biological Processes</td><td>19</td><td>positive regulation of interleukin-6 production</td><td>-5.79102</td><td>12.43869</td><td>8.620203</td><td>30242</td><td>93</td><td>183</td><td>7</td><td>3.825137</td><td>1.417845</td><td>351|2149|3569|4843|7099|7124|59341</td><td>APP|F2R|IL6|NOS2|TLR4|TNF|TRPV4</td><td>-4.46309</td><td>0</td><td>101</td><td>0</td><td>0</td><td>-7.60563</td><td>23.60811866</td></tr>
<tr><td>1</td><td>-5.67808</td><td>GO:0032731</td><td>M1</td><td>1</td><td>0</td><td>GO Biological Processes</td><td>19</td><td>positive regulation of interleukin-1 beta production</td><td>-5.67808</td><td>15.9926</td><td>9.220393</td><td>30242</td><td>62</td><td>183</td><td>6</td><td>3.278689</td><td>1.316393</td><td>351|834|3569|7099|7124|114548</td><td>APP|CASP1|IL6|TLR4|TNF|NLRP3</td><td>-4.35832</td><td>0</td><td>101</td><td>0</td><td>0</td><td>-7.60563</td><td>23.60811866</td></tr>
<tr><td>1</td><td>-5.521</td><td>GO:0032677</td><td>M1</td><td>1</td><td>0</td><td>GO Biological Processes</td><td>19</td><td>regulation of interleukin-8 production</td><td>-5.521</td><td>11.34116</td><td>8.162701</td><td>30242</td><td>102</td><td>183</td><td>7</td><td>3.825137</td><td>1.417845</td><td>1991|2149|3569|4843|5970|7099|7124</td><td>ELANE|F2R|IL6|NOS2|RELA|TLR4|TNF</td><td>-4.21822</td><td>0</td><td>101</td><td>0</td><td>0</td><td>-7.60563</td><td>23.60811866</td></tr>
<tr><td>1</td><td>-5.49264</td><td>GO:0032637</td><td>M1</td><td>1</td><td>0</td><td>GO Biological Processes</td><td>19</td><td>interleukin-8 production</td><td>-5.49264</td><td>11.23105</td><td>8.115413</td><td>30242</td><td>103</td><td>183</td><td>7</td><td>3.825137</td><td>1.417845</td><td>1991|2149|3569|4843|5970|7099|7124</td><td>ELANE|F2R|IL6|NOS2|RELA|TLR4|TNF</td><td>-4.19318</td><td>0</td><td>101</td><td>0</td><td>0</td><td>-7.60563</td><td>23.60811866</td></tr>
<tr><td>1</td><td>-5.29473</td><td>GO:0002534</td><td>M1</td><td>1</td><td>0</td><td>GO Biological Processes</td><td>19</td><td>cytokine production involved in inflammatory response</td><td>-5.29473</td><td>13.7714</td><td>8.465518</td><td>30242</td><td>72</td><td>183</td><td>6</td><td>3.278689</td><td>1.316393</td><td>2147|3569|4843|5465|7099|7124</td><td>F2|IL6|NOS2|PPARA|TLR4|TNF</td><td>-4.02714</td><td>0</td><td>101</td><td>0</td><td>0</td><td>-7.60563</td><td>23.60811866</td></tr>
<tr><td>1</td><td>-5.29473</td><td>GO:1900015</td><td>M1</td><td>1</td><td>0</td><td>GO Biological Processes</td><td>19</td><td>regulation of cytokine production involved in inflammatory response</td><td>-5.29473</td><td>13.7714</td><td>8.465518</td><td>30242</td><td>72</td><td>183</td><td>6</td><td>3.278689</td><td>1.316393</td><td>2147|3569|4843|5465|7099|7124</td><td>F2|IL6|NOS2|PPARA|TLR4|TNF</td><td>-4.02714</td><td>0</td><td>101</td><td>0</td><td>0</td><td>-7.60563</td><td>23.60811866</td></tr>
<tr><td>1</td><td>-5.27623</td><td>GO:0032611</td><td>M1</td><td>1</td><td>0</td><td>GO Biological Processes</td><td>19</td><td>interleukin-1 beta production</td><td>-5.27623</td><td>10.4216</td><td>7.75918</td><td>30242</td><td>111</td><td>183</td><td>7</td><td>3.825137</td><td>1.417845</td><td>351|834|2149|3569|7099|7124|114548</td><td>APP|CASP1|F2R|IL6|TLR4|TNF|NLRP3</td><td>-4.01119</td><td>0</td><td>101</td><td>0</td><td>0</td><td>-7.60563</td><td>23.60811866</td></tr>
<tr><td>1</td><td>-5.27623</td><td>GO:0032651</td><td>M1</td><td>1</td><td>0</td><td>GO Biological Processes</td><td>19</td><td>regulation of interleukin-1 beta production</td><td>-5.27623</td><td>10.4216</td><td>7.75918</td><td>30242</td><td>111</td><td>183</td><td>7</td><td>3.825137</td><td>1.417845</td><td>351|834|2149|3569|7099|7124|114548</td><td>APP|CASP1|F2R|IL6|TLR4|TNF|NLRP3</td><td>-4.01119</td><td>0</td><td>101</td><td>0</td><td>0</td><td>-7.60563</td><td>23.60811866</td></tr>
<tr><td>1</td><td>-5.17445</td><td>GO:1901222</td><td>M1</td><td>1</td><td>0</td><td>GO Biological Processes</td><td>19</td><td>regulation of NIK/NF-kappaB signaling</td><td>-5.17445</td><td>10.05911</td><td>7.594391</td><td>30242</td><td>115</td><td>183</td><td>7</td><td>3.825137</td><td>1.417845</td><td>351|1956|4792|5970|7099|7124|114548</td><td>APP|EGFR|NFKBIA|RELA|TLR4|TNF|NLRP3</td><td>-3.91998</td><td>0</td><td>101</td><td>0</td><td>0</td><td>-7.60563</td><td>23.60811866</td></tr>
<tr><td>1</td><td>-5.04612</td><td>GO:0007254</td><td>M1</td><td>1</td><td>0</td><td>GO Biological Processes</td><td>19</td><td>JNK cascade</td><td>-5.04612</td><td>7.869373</td><td>6.966465</td><td>30242</td><td>168</td><td>183</td><td>8</td><td>4.371585</td><td>1.511428</td><td>136|351|1956|4221|5734|7099|7124|59341</td><td>ADORA2B|APP|EGFR|MEN1|PTGER4|TLR4|TNF|TRPV4</td><td>-3.80487</td><td>0</td><td>101</td><td>0</td><td>0</td><td>-7.60563</td><td>23.60811866</td></tr>
<tr><td>1</td><td>-5.01826</td><td>GO:0001774</td><td>M1</td><td>1</td><td>0</td><td>GO Biological Processes</td><td>19</td><td>microglial cell activation</td><td>-5.01826</td><td>17.58051</td><td>8.875969</td><td>30242</td><td>47</td><td>183</td><td>5</td><td>2.73224</td><td>1.205087</td><td>351|3569|7124|7442|10135</td><td>APP|IL6|TNF|TRPV1|NAMPT</td><td>-3.77989</td><td>0</td><td>101</td><td>0</td><td>0</td><td>-7.60563</td><td>23.60811866</td></tr>
<tr><td>1</td><td>-4.88331</td><td>GO:0001818</td><td>M1</td><td>1</td><td>0</td><td>GO Biological Processes</td><td>19</td><td>negative regulation of cytokine production</td><td>-4.88331</td><td>5.06358</td><td>6.043385</td><td>30242</td><td>359</td><td>183</td><td>11</td><td>6.010929</td><td>1.757049</td><td>1991|2147|2335|3569|5465|5734|7040|7099|7124|8986|114548</td><td>ELANE|F2|FN1|IL6|PPARA|PTGER4|TGFB1|TLR4|TNF|RPS6KA4|NLRP3</td><td>-3.66687</td><td>0</td><td>101</td><td>0</td><td>0</td><td>-7.60563</td><td>23.60811866</td></tr>
<tr><td>1</td><td>-4.70593</td><td>GO:0050829</td><td>M1</td><td>1</td><td>0</td><td>GO Biological Processes</td><td>19</td><td>defense response to Gram-negative bacterium</td><td>-4.70593</td><td>10.89605</td><td>7.376805</td><td>30242</td><td>91</td><td>183</td><td>6</td><td>3.278689</td><td>1.316393</td><td>834|1991|2147|3569|4843|7099</td><td>CASP1|ELANE|F2|IL6|NOS2|TLR4</td><td>-3.51082</td><td>0</td><td>101</td><td>0</td><td>0</td><td>-7.60563</td><td>23.60811866</td></tr>
<tr><td>1</td><td>-4.57807</td><td>GO:0032872</td><td>M1</td><td>1</td><td>0</td><td>GO Biological Processes</td><td>19</td><td>regulation of stress-activated MAPK cascade</td><td>-4.57807</td><td>6.779767</td><td>6.317765</td><td>30242</td><td>195</td><td>183</td><td>8</td><td>4.371585</td><td>1.511428</td><td>351|1956|3156|4221|4986|7099|7124|59341</td><td>APP|EGFR|HMGCR|MEN1|OPRK1|TLR4|TNF|TRPV4</td><td>-3.40204</td><td>0</td><td>101</td><td>0</td><td>0</td><td>-7.60563</td><td>23.60811866</td></tr>
<tr><td>1</td><td>-4.53073</td><td>GO:0070302</td><td>M1</td><td>1</td><td>0</td><td>GO Biological Processes</td><td>19</td><td>regulation of stress-activated protein kinase</td><td>-4.53073</td><td>6.677044</td><td>6.253344</td><td>30242</td><td>198</td><td>183</td><td>8</td><td>4.371585</td><td>1.511428</td><td>351|1956|3156|4221|4986|7099|7124|59341</td><td>APP|EGFR|HMGCR|MEN1|OPRK1|TLR4|TN</td><td>-3.3625</td><td>0</td><td>101</td><td>0</td><td>0</td><td>-7.60563</td><td>23.60811866</td></tr>
</table>

								signaling cascade											F|TRPV4							
1	-4.52269	GO:0002532	M1	1	0	GO Biological Processes	19	production of molecular mediator involved in inflammatory response	-4.52269	10.11777	7.054031	30242	98	183	6	3.278689	1.316393	2147|3569|4843|5465|7099|7124	F2|IL6|NOS2|PPARA|TLR4|TNF	-3.35649	0	101	0	0	-7.60563	23.60811866
1	-4.52269	GO:0032642	M1	1	0	GO Biological Processes	19	regulation of chemokine production	-4.52269	10.11777	7.054031	30242	98	183	6	3.278689	1.316393	351|1991|3569|7099|7124|59341	APP|ELANE|IL6|TLR4|TNF|TRPV4	-3.35649	0	101	0	0	-7.60563	23.60811866
1	-4.4997	GO:0038061	M1	1	0	GO Biological Processes	19	NIK/NF-kappaB signaling	-4.4997	7.923273	6.542893	30242	146	183	7	3.825137	1.417845	351|1956|4792|5970|7099|7124|114548	APP|EGFR|NFKBIA|RELA|TLR4|TNF|NLRP3	-3.33635	0	101	0	0	-7.60563	23.60811866
1	-4.4977	GO:0032602	M1	1	0	GO Biological Processes	19	chemokine production	-4.4977	10.01557	7.010576	30242	99	183	6	3.278689	1.316393	351|1991|3569|7099|7124|59341	APP|ELANE|IL6|TLR4|TNF|TRPV4	-3.33635	0	101	0	0	-7.60563	23.60811866
1	-4.49437	GO:0031663	M1	1	0	GO Biological Processes	19	lipopolysaccharide-mediated signaling pathway	-4.49437	13.7714	7.726389	30242	60	183	5	2.73224	1.205087	4792|6347|7040|7099|7124	NFKBIA|CCL2|TGFB1|TLR4|TNF	-3.33342	0	101	0	0	-7.60563	23.60811866
1	-4.33034	GO:0042116	M1	1	0	GO Biological Processes	19	macrophage activation	-4.33034	9.35416	6.722786	30242	106	183	6	3.278689	1.316393	351|3569|7099|7124|7442|10135	APP|IL6|TLR4|TNF|TRPV1|NAMPT	-3.18628	0	101	0	0	-7.60563	23.60811866
1	-4.20006	GO:1901224	M1	1	0	GO Biological Processes	19	positive regulation of NIK/NF-kappaB signaling	-4.20006	11.97513	7.121335	30242	69	183	5	2.73224	1.205087	351|1956|5970|7099|7124	APP|EGFR|RELA|TLR4|TNF	-3.06779	0	101	0	0	-7.60563	23.60811866
1	-4.16215	GO:0032635	M1	1	0	GO Biological Processes	19	interleukin-6 production	-4.16215	7.010896	6.040881	30242	165	183	7	3.825137	1.417845	351|2149|3569|4843|7099|7124|59341	APP|F2R|IL6|NOS2|TLR4|TNF|TRPV4	-3.03324	0	101	0	0	-7.60563	23.60811866
1	-4.16215	GO:0032675	M1	1	0	GO Biological Processes	19	regulation of interleukin-6 production	-4.16215	7.010896	6.040881	30242	165	183	7	3.825137	1.417845	351|2149|3569|4843|7099|7124|59341	APP|F2R|IL6|NOS2|TLR4|TNF|TRPV4	-3.03324	0	101	0	0	-7.60563	23.60811866
1	-4.1404	GO:0032722	M1	1	0	GO Biological Processes	19	positive regulation of chemokine production	-4.1404	11.6378	7.00201	30242	71	183	5	2.73224	1.205087	351|3569|7099|7124|59341	APP|IL6|TLR4|TNF|TRPV4	-3.01298	0	101	0	0	-7.60563	23.60811866
1	-4.11126	GO:0032729	M1	1	0	GO Biological Processes	19	positive regulation of interferon-gamma production	-4.11126	11.47617	6.944123	30242	72	183	5	2.73224	1.205087	3558|5142|7099|7124|729230	IL2|PDE4B|TLR4|TNF|CCR2	-2.98937	0	101	0	0	-7.60563	23.60811866
1	-3.99998	GO:0010543	M1	1	0	GO Biological Processes	19	regulation of platelet activation	-3.99998	16.52568	7.666537	30242	40	183	4	2.185792	1.080886	2147|3569|4846|7099	F2|IL6|NOS3|TLR4	-2.8875	0	101	0	0	-7.60563	23.60811866
1	-3.91317	GO:0032874	M1	1	0	GO Biological Processes	19	positive regulation of stress-activated MAPK cascade	-3.91317	7.869373	6.028928	30242	126	183	6	3.278689	1.316393	351|3156|4986|7099|7124|59341	APP|HMGCR|OPRK1|TLR4|TNF|TRPV4	-2.81306	0	101	0	0	-7.60563	23.60811866
1	-3.87563	GO:0070304	M1	1	0	GO Biological Processes	19	positive regulation of stress-activated protein kinase signaling cascade	-3.87563	7.746414	5.968018	30242	128	183	6	3.278689	1.316393	351|3156|4986|7099|7124|59341	APP|HMGCR|OPRK1|TLR4|TNF|TRPV4	-2.77725	0	101	0	0	-7.60563	23.60811866
1	-3.76689	GO:0046328	M1	1	0	GO Biological Processes	19	regulation of JNK cascade	-3.76689	7.39956	5.792925	30242	134	183	6	3.278689	1.316393	351|1956|4221|7099|7124|59341	APP|EGFR|MEN1|TLR4|TNF|TRPV4	-2.68385	0	101	0	0	-7.60563	23.60811866
1	-3.57263	GO:0007252	M1	1	0	GO Biological Processes	19	I-kappaB phosphorylation	-3.57263	23.60812	8.086415	30242	21	183	3	1.639344	0.938686	3551|7099|7124	IKBKB|TLR4|TNF	-2.51178	0	101	0	0	-7.60563	23.60811866
1	-3.48725	GO:0042742	M1	1	0	GO Biological Processes	19	defense response to bacterium	-3.48725	4.201445	4.727544	30242	354	183	9	4.918033	1.598524	834|1991|2147|3569|4843|7099|7124|7132|29110	CASP1|ELANE|F2|IL6|NOS2|TLR4|TNF|TNFRSF1A|TBK1	-2.43491	0	101	0	0	-7.60563	23.60811866
1	-3.38048	GO:0032760	M1	1	0	GO Biological Processes	19	positive regulation of tumor necrosis factor production	-3.38048	8.022176	5.570091	30242	103	183	5	2.73224	1.205087	351|3066|3569|7099|729230	APP|HDAC2|IL6|TLR4|CCR2	-2.338	0	101	0	0	-7.60563	23.60811866
1	-3.30456	GO:1903557	M1	1	0	GO Biological Processes	19	positive regulation of tumor necrosis factor	-3.30456	7.722282	5.435123	30242	107	183	5	2.73224	1.205087	351|3066|3569|7099|729230	APP|HDAC2|IL6|TLR4|CCR2	-2.26935	0	101	0	0	-7.60563	23.60811866

								superfamily cytokine production																	
1	-3.29117	GO:1900017	M1	1	0	GO Biological Processes	19	positive regulation of cytokine production involved in inflammatory response	-3.29117	19.0681	7.191458	30242	26	183	3	1.639344	0.938686	3569|7099|7124	IL6|TLR4|TNF	-2.25835	0	101	0	0	-7.60563	23.60811866
1	-3.21407	GO:0032609	M1	1	0	GO Biological Processes	19	interferon-gamma production	-3.21407	7.377537	5.275927	30242	112	183	5	2.73224	1.205087	3558|5142|7099|7124|729230	IL2|PDE4B|TLR4|TNF|CCR2	-2.18776	0	101	0	0	-7.60563	23.60811866
1	-3.21407	GO:0032649	M1	1	0	GO Biological Processes	19	regulation of interferon-gamma production	-3.21407	7.377537	5.275927	30242	112	183	5	2.73224	1.205087	3558|5142|7099|7124|729230	IL2|PDE4B|TLR4|TNF|CCR2	-2.18776	0	101	0	0	-7.60563	23.60811866
1	-2.67313	GO:0046330	M1	1	0	GO Biological Processes	19	positive regulation of JNK cascade	-2.67313	7.427273	4.737976	30242	89	183	4	2.185792	1.080886	351|7099|7124|59341	APP|TLR4|TNF|TRPV4	-1.70426	0	101	0	0	-7.60563	23.60811866
1	-2.30143	GO:0032640	M1	1	0	GO Biological Processes	19	tumor necrosis factor production	-2.30143	4.565106	3.753586	30242	181	183	5	2.73224	1.205087	351|3066|3569|7099|729230	APP|HDAC2|IL6|TLR4|CCR2	-1.3679	0	101	0	0	-7.60563	23.60811866
1	-2.30143	GO:0032680	M1	1	0	GO Biological Processes	19	regulation of tumor necrosis factor production	-2.30143	4.565106	3.753586	30242	181	183	5	2.73224	1.205087	351|3066|3569|7099|729230	APP|HDAC2|IL6|TLR4|CCR2	-1.3679	0	101	0	0	-7.60563	23.60811866
1	-2.24236	GO:0071706	M1	1	0	GO Biological Processes	19	tumor necrosis factor superfamily cytokine production	-2.24236	4.418632	3.658905	30242	187	183	5	2.73224	1.205087	351|3066|3569|7099|729230	APP|HDAC2|IL6|TLR4|CCR2	-1.31261	0	101	0	0	-7.60563	23.60811866
1	-2.24236	GO:1903555	M1	1	0	GO Biological Processes	19	regulation of tumor necrosis factor superfamily cytokine production	-2.24236	4.418632	3.658905	30242	187	183	5	2.73224	1.205087	351|3066|3569|7099|729230	APP|HDAC2|IL6|TLR4|CCR2	-1.31261	0	101	0	0	-7.60563	23.60811866
1	-7.53222	GO:0070633	M1	1	0	GO Biological Processes	19	transepithelial transport	-7.53222	31.98519	13.46756	30242	31	183	6	3.278689	1.316393	1080|1910|4363|5243|6523|9429	CFTR|EDNRB|ABCC1|ABCB1|SLC5A1|ABCG2	-6.04296	0	102	1	1	-7.53222	49.57704918
1	-4.596	GO:0042045	M1	1	0	GO Biological Processes	19	epithelial fluid transport	-4.596	49.57705	11.98765	30242	10	183	3	1.639344	0.938686	1080|1910|6523	CFTR|EDNRB|SLC5A1	-3.4183	0	102	0	0	-7.53222	49.57704918
1	-2.98278	GO:0042044	M1	1	0	GO Biological Processes	19	fluid transport	-2.98278	15.02335	6.288932	30242	33	183	3	1.639344	0.938686	1080|1910|6523	CFTR|EDNRB|SLC5A1	-1.98159	0	102	0	0	-7.53222	49.57704918
1	-2.63793	GO:0006885	M1	1	0	GO Biological Processes	19	regulation of pH	-2.63793	7.264037	4.669393	30242	91	183	4	2.185792	1.080886	596|760|1080|1910	BCL2|CA2|CFTR|EDNRB	-1.67418	0	102	0	0	-7.53222	49.57704918
1	-2.44365	GO:0030004	M1	1	0	GO Biological Processes	19	cellular monovalent inorganic cation homeostasis	-2.44365	6.417741	4.29743	30242	103	183	4	2.185792	1.080886	596|760|1080|3778	BCL2|CA2|CFTR|KCNMA1	-1.49711	0	102	0	0	-7.53222	49.57704918
1	-7.44423	GO:0007190	M1	1	0	GO Biological Processes	19	activation of adenylate cyclase activity	-7.44423	30.98566	13.24188	30242	32	183	6	3.278689	1.316393	136|140|153|154|1812|1909	ADORA2B|ADORA3|ADRB1|ADRB2|DRD1|EDNRA	-5.96341	0	103	1	1	-7.44423	55.0856102
1	-6.20876	GO:0002024	M1	1	0	GO Biological Processes	19	diet induced thermogenesis	-6.20876	55.08561	14.62142	30242	12	183	4	2.185792	1.080886	153|154|7442|59341	ADRB1|ADRB2|TRPV1|TRPV4	-4.84272	0	103	0	0	-7.44423	55.0856102
1	-4.928	GO:0009409	M1	1	0	GO Biological Processes	19	response to cold	-4.928	16.86294	8.670916	30242	49	183	5	2.73224	1.205087	153|154|4792|5024|8989	ADRB1|ADRB2|NFKBIA|P2RX3|TRPA1	-3.70236	0	103	0	0	-7.44423	55.0856102
1	-4.90168	GO:0002021	M1	1	0	GO Biological Processes	19	response to dietary excess	-4.90168	27.54281	10.14976	30242	24	183	4	2.185792	1.080886	153|154|7442|59341	ADRB1|ADRB2|TRPV1|TRPV4	-3.68342	0	103	0	0	-7.44423	55.0856102
1	-3.61905	GO:0050873	M1	1	0	GO Biological Processes	19	brown fat cell differentiation	-3.61905	13.22055	6.74786	30242	50	183	4	2.185792	1.080886	153|154|5743|59341	ADRB1|ADRB2|PTGS2|TRPV4	-2.55243	0	103	0	0	-7.44423	55.0856102
1	-3.39647	GO:1990845	M1	1	0	GO Biological Processes	19	adaptive thermogenesis	-3.39647	6.315548	5.210258	30242	157	183	6	3.278689	1.316393	153|154|7099|7442|59341|729230	ADRB1|ADRB2|TLR4|TRPV1|TRPV4|CCR2	-2.35299	0	103	0	0	-7.44423	55.0856102
1	-2.31425	GO:0048640	M1	1	0	GO Biological	19	negative regulation of	-2.31425	5.90203	4.055288	30242	112	183	4	2.185792	1.080886	153|154|5465|6532	ADRB1|ADRB2|PPAR	-1.37977	0	103	0	0	-7.44423	55.0856102

						Processes		developmental growth											A|SLC6A4							
1	-7.349	GO:0014832	M1	1	0	GO Biological Processes	19	urinary bladder smooth muscle contraction	-7.349	94.43247	19.28982	30242	7	183	4	2.185792	1.080886	3356|3778|5024|7442	HTR2A|KCNMA1|P2RX3|TRPV1	-5.87776	0	104	1	1	-7.349	94.43247463
1	-6.79682	GO:0014848	M1	1	0	GO Biological Processes	19	urinary tract smooth muscle contraction	-6.79682	73.44748	16.96056	30242	9	183	4	2.185792	1.080886	3356|3778|5024|7442	HTR2A|KCNMA1|P2RX3|TRPV1	-5.37898	0	104	0	0	-7.349	94.43247463
1	-6.05112	GO:0048266	M1	1	0	GO Biological Processes	19	behavioral response to pain	-6.05112	50.84826	14.0264	30242	13	183	4	2.185792	1.080886	4914|5024|6335|7442	NTRK1|P2RX3|SCN9A|TRPV1	-4.69899	0	104	0	0	-7.349	94.43247463
1	-5.65153	GO:0007635	M1	1	0	GO Biological Processes	19	chemosensory behavior	-5.65153	41.31421	12.58532	30242	16	183	4	2.185792	1.080886	4914|5024|5582|7442	NTRK1|P2RX3|PRKCG|TRPV1	-4.33864	0	104	0	0	-7.349	94.43247463
1	-5.36639	GO:0060073	M1	1	0	GO Biological Processes	19	micturition	-5.36639	82.62842	15.60239	30242	6	183	3	1.639344	0.938686	148|3778|7442	ADRA1A|KCNMA1|TRPV1	-4.08918	0	104	0	0	-7.349	94.43247463
1	-4.33459	GO:0033198	M1	1	0	GO Biological Processes	19	response to ATP	-4.33459	20.03113	8.534729	30242	33	183	4	2.185792	1.080886	834|5024|5743|7442	CASP1|P2RX3|PTGS2|TRPV1	-3.18937	0	104	0	0	-7.349	94.43247463
1	-3.77899	GO:0071318	M1	1	0	GO Biological Processes	19	cellular response to ATP	-3.77899	27.54281	8.789081	30242	18	183	3	1.639344	0.938686	5024|5743|7442	P2RX3|PTGS2|TRPV1	-2.69427	0	104	0	0	-7.349	94.43247463
1	-7.34865	GO:1902894	M1	1	0	GO Biological Processes	19	negative regulation of pri-miRNA transcription by RNA polymerase II	-7.34865	48.60495	15.31898	30242	17	183	5	2.73224	1.205087	5465|5467|5468|5970|7040	PPARA|PPARD|PPARG|RELA|TGFB1	-5.87776	0	105	1	1	-7.34865	48.60495018
1	-5.33986	GO:0016042	M1	1	0	GO Biological Processes	19	lipid catabolic process	-5.33986	5.663007	6.553403	30242	321	183	11	6.010929	1.757049	134|150|427|1268|1544|1545|1588|2166|5465|5467|7124	ADORA1|ADRA2A|ASAH1|CNR1|CYP1A2|CYP1B1|CYP19A1|FAAH|PPARA|PPARD|TNF	-4.06557	0	105	0	0	-7.34865	48.60495018
1	-4.45963	GO:0010887	M1	1	0	GO Biological Processes	19	negative regulation of cholesterol storage	-4.45963	45.07004	11.40644	30242	11	183	3	1.639344	0.938686	5465|5467|5468	PPARA|PPARD|PPARG	-3.30278	0	105	0	0	-7.34865	48.60495018
1	-4.04368	GO:0045923	M1	1	0	GO Biological Processes	19	positive regulation of fatty acid metabolic process	-4.04368	16.94942	7.776577	30242	39	183	4	2.185792	1.080886	5465|5467|5468|5743	PPARA|PPARD|PPARG|PTGS2	-2.92688	0	105	0	0	-7.34865	48.60495018
1	-3.70629	GO:0010885	M1	1	0	GO Biological Processes	19	regulation of cholesterol storage	-3.70629	26.09318	8.536899	30242	19	183	3	1.639344	0.938686	5465|5467|5468	PPARA|PPARD|PPARG	-2.63055	0	105	0	0	-7.34865	48.60495018
1	-3.58581	GO:0019217	M1	1	0	GO Biological Processes	19	regulation of fatty acid metabolic process	-3.58581	8.884776	5.941974	30242	93	183	5	2.73224	1.205087	1268|5465|5467|5468|5743	CNR1|PPARA|PPARD|PPARG|PTGS2	-2.5219	0	105	0	0	-7.34865	48.60495018
1	-3.57263	GO:0010878	M1	1	0	GO Biological Processes	19	cholesterol storage	-3.57263	23.60812	8.086415	30242	21	183	3	1.639344	0.938686	5465|5467|5468	PPARA|PPARD|PPARG	-2.51178	0	105	0	0	-7.34865	48.60495018
1	-3.50313	GO:0042180	M1	1	0	GO Biological Processes	19	cellular ketone metabolic process	-3.50313	5.482454	5.098102	30242	211	183	7	3.825137	1.417845	1268|1645|3156|5465|5467|5468|5743	CNR1|AKR1C1|HMGCR|PPARA|PPARD|PPARG|PTGS2	-2.4486	0	105	0	0	-7.34865	48.60495018
1	-3.49074	GO:0030258	M1	1	0	GO Biological Processes	19	lipid modification	-3.49074	5.456593	5.080771	30242	212	183	7	3.825137	1.417845	185|1133|1268|1543|5465|5467|51179	AGTR1|CHRM5|CNR1|CYP1A1|PPARA|PPARD|HAO2	-2.43746	0	105	0	0	-7.34865	48.60495018
1	-2.98278	GO:0046320	M1	1	0	GO Biological Processes	19	regulation of fatty acid oxidation	-2.98278	15.02335	6.288932	30242	33	183	3	1.639344	0.938686	1268|5465|5467	CNR1|PPARA|PPARD	-1.98159	0	105	0	0	-7.34865	48.60495018
1	-2.87883	GO:0010565	M1	1	0	GO Biological Processes	19	regulation of cellular ketone metabolic process	-2.87883	6.212663	4.70082	30242	133	183	5	2.73224	1.205087	1268|5465|5467|5468|5743	CNR1|PPARA|PPARD|PPARG|PTGS2	-1.88751	0	105	0	0	-7.34865	48.60495018
1	-2.69054	GO:0044242	M1	1	0	GO Biological Processes	19	cellular lipid catabolic process	-2.69054	4.611819	4.146919	30242	215	183	6	3.278689	1.316393	427|1268|1545|2166|5465|5467	ASAH1|CNR1|CYP1B1|FAAH|PPARA|PPARD	-1.71857	0	105	0	0	-7.34865	48.60495018
1	-2.4897	GO:0009062	M1	1	0	GO Biological	19	fatty acid catabolic	-2.4897	6.610273	4.384644	30242	100	183	4	2.185792	1.080886	1268|2166|5465|5467	CNR1|FAAH|PPARA|P	-1.53774	0	105	0	0	-7.34865	48.60495018

						Processes		process											PARD							
1	-2.44365	GO:0019395	M1	1	0	GO Biological Processes	19	fatty acid oxidation	-2.44365	6.417741	4.29743	30242	103	183	4	2.185792	1.080886	1268|5465|5467|51179	CNR1|PPARA|PPARD|HAO2	-1.49711	0	105	0	0	-7.34865	48.60495018
1	-2.37022	GO:0034440	M1	1	0	GO Biological Processes	19	lipid oxidation	-2.37022	6.120623	4.159515	30242	108	183	4	2.185792	1.080886	1268|5465|5467|51179	CNR1|PPARA|PPARD|HAO2	-1.42951	0	105	0	0	-7.34865	48.60495018
1	-2.18392	GO:0072329	M1	1	0	GO Biological Processes	19	monocarboxylic acid catabolic process	-2.18392	5.418257	3.815397	30242	122	183	4	2.185792	1.080886	1268|2166|5465|5467	CNR1|FAAH|PPARA|PPARD	-1.25885	0	105	0	0	-7.34865	48.60495018
1	-7.27712	GO:0051482	M1	1	0	GO Biological Processes	19	positive regulation of cytosolic calcium ion concentration involved in phospholipase C-activating G protein-coupled signaling pathway	-7.27712	29.16297	12.82016	30242	34	183	6	3.278689	1.316393	185|1812|1813|1814|2149|3358	AGTR1|DRD1|DRD2|DRD3|F2R|HTR2C	-5.81673	0	106	1	1	-7.27712	29.16297011
1	-4.13489	GO:0001990	M1	1	0	GO Biological Processes	19	regulation of systemic arterial blood pressure by hormone	-4.13489	17.8656	8.009394	30242	37	183	4	2.185792	1.080886	185|1636|2149|4846	AGTR1|ACE|F2R|NOS3	-3.00894	0	106	0	0	-7.27712	29.16297011
1	-3.81751	GO:0050886	M1	1	0	GO Biological Processes	19	endocrine process	-3.81751	9.955231	6.374465	30242	83	183	5	2.73224	1.205087	185|1636|2149|4846|4986	AGTR1|ACE|F2R|NOS3|OPRK1	-2.7267	0	106	0	0	-7.27712	29.16297011
1	-3.45213	GO:0003081	M1	1	0	GO Biological Processes	19	regulation of systemic arterial blood pressure by renin-angiotensin	-3.45213	21.55524	7.694542	30242	23	183	3	1.639344	0.938686	185|1636|2149	AGTR1|ACE|F2R	-2.40475	0	106	0	0	-7.27712	29.16297011
1	-3.34249	GO:0003071	M1	1	0	GO Biological Processes	19	renal system process involved in regulation of systemic arterial blood pressure	-3.34249	19.83082	7.349367	30242	25	183	3	1.639344	0.938686	134|185|2149	ADORA1|AGTR1|F2R	-2.30396	0	106	0	0	-7.27712	29.16297011
1	-2.87173	GO:0044060	M1	1	0	GO Biological Processes	19	regulation of endocrine process	-2.87173	13.7714	5.982457	30242	36	183	3	1.639344	0.938686	185|2149|4986	AGTR1|F2R|OPRK1	-1.88231	0	106	0	0	-7.27712	29.16297011
1	-7.22231	GO:0044070	M1	1	0	GO Biological Processes	19	regulation of anion transport	-7.22231	15.19603	10.34626	30242	87	183	8	4.371585	1.511428	134|135|760|1080|2915|3351|5243|11255	ADORA1|ADORA2A|CA2|CFTR|GRM5|HTR1B|ABCB1|HRH3	-5.76811	0	107	1	1	-7.22231	55.0856102
1	-5.30231	GO:0006821	M1	1	0	GO Biological Processes	19	chloride transport	-5.30231	10.51634	7.801693	30242	110	183	7	3.825137	1.417845	1080|2554|2558|2566|2915|5243|6523	CFTR|GABRA1|GABRA5|GABRG2|GRM5|ABCB1|SLC5A1	-4.03267	0	107	0	0	-7.22231	55.0856102
1	-4.77626	GO:0015698	M1	1	0	GO Biological Processes	19	inorganic anion transport	-4.77626	7.224342	6.589737	30242	183	183	8	4.371585	1.511428	1080|2554|2558|2566|2915|4363|5243|6523	CFTR|GABRA1|GABRA5|GABRG2|GRM5|ABCC1|ABCB1|SLC5A1	-3.56972	0	107	0	0	-7.22231	55.0856102
1	-4.75751	GO:0007214	M1	1	0	GO Biological Processes	19	gamma-aminobutyric acid signaling pathway	-4.75751	25.42413	9.721283	30242	26	183	4	2.185792	1.080886	2554|2558|2566|3350	GABRA1|GABRA5|GABRG2|HTR1A	-3.55409	0	107	0	0	-7.22231	55.0856102
1	-4.74896	GO:0010359	M1	1	0	GO Biological Processes	19	regulation of anion channel activity	-4.74896	55.08561	12.6619	30242	9	183	3	1.639344	0.938686	1080|2915|5243	CFTR|GRM5|ABCB1	-3.54861	0	107	0	0	-7.22231	55.0856102
1	-4.65202	GO:0098661	M1	1	0	GO Biological Processes	19	inorganic anion transmembrane transport	-4.65202	10.66173	7.281089	30242	93	183	6	3.278689	1.316393	1080|2554|2558|2566|2915|6523	CFTR|GABRA1|GABRA5|GABRG2|GRM5|SLC5A1	-3.46165	0	107	0	0	-7.22231	55.0856102
1	-4.596	GO:2001225	M1	1	0	GO Biological Processes	19	regulation of chloride transport	-4.596	49.57705	11.98765	30242	10	183	3	1.639344	0.938686	1080|2915|5243	CFTR|GRM5|ABCB1	-3.4183	0	107	0	0	-7.22231	55.0856102
1	-4.26166	GO:1902476	M1	1	0	GO Biological Processes	19	chloride transmembrane transport	-4.26166	12.3326	7.245688	30242	67	183	5	2.73224	1.205087	1080|2554|2558|2566|6523	CFTR|GABRA1|GABRA5|GABRG2|SLC5A1	-3.12258	0	107	0	0	-7.22231	55.0856102
1	-3.85879	GO:0098656	M1	1	0	GO Biological	19	anion transmembrane	-3.85879	5.352448	5.359126	30242	247	183	8	4.371585	1.511428	1080|2554|2558|2566|2	CFTR|GABRA1|GAB	-2.76214	0	107	0	0	-7.22231	55.0856102

						Processes		transport										915|4363|5243|6523	RA5|GABRG2|GRM5|ABCC1|ABCB1|SLC5A1							
1	-3.34249	GO:1903959	M1	1	0	GO Biological Processes	19	regulation of anion transmembrane transport	-3.34249	19.83082	7.349367	30242	25	183	3	1.639344	0.938686	1080|2915|5243	CFTR|GRM5|ABCB1	-2.30396	0	107	0	0	-7.22231	55.0856102
1	-2.64755	GO:1903793	M1	1	0	GO Biological Processes	19	positive regulation of anion transport	-2.64755	11.52955	5.391182	30242	43	183	3	1.639344	0.938686	135|1080|5243	ADORA2A|CFTR|ABCB1	-1.68278	0	107	0	0	-7.22231	55.0856102
1	-7.19763	GO:0045454	M1	1	0	GO Biological Processes	19	cell redox homeostasis	-7.19763	28.32974	12.6227	30242	35	183	6	3.278689	1.316393	328|1728|2936|4842|4843|4846	APEX1|NQO1|GSR|NOS1|NOS2|NOS3	-5.74515	0	108	1	1	-7.19763	49.57704918
1	-5.43561	GO:0044282	M1	1	0	GO Biological Processes	19	small molecule catabolic process	-5.43561	5.246249	6.482069	30242	378	183	12	6.557377	1.829835	100|217|1268|1543|2166|4842|4843|4846|5444|5465|5467|7157	ADA|ALDH2|CNR1|CYP1A1|FAAH|NOS1|NOS2|NOS3|PON1|PPARA|PPARD|TP53	-4.14435	0	108	0	0	-7.19763	49.57704918
1	-5.05972	GO:0031281	M1	1	0	GO Biological Processes	19	positive regulation of cyclase activity	-5.05972	30.0467	10.63401	30242	22	183	4	2.185792	1.080886	775|4842|4843|4846	CACNA1C|NOS1|NOS2|NOS3	-3.8175	0	108	0	0	-7.19763	49.57704918
1	-4.9788	GO:0051349	M1	1	0	GO Biological Processes	19	positive regulation of lyase activity	-4.9788	28.74032	10.38417	30242	23	183	4	2.185792	1.080886	775|4842|4843|4846	CACNA1C|NOS1|NOS2|NOS3	-3.74566	0	108	0	0	-7.19763	49.57704918
1	-4.90168	GO:0030810	M1	1	0	GO Biological Processes	19	positive regulation of nucleotide biosynthetic process	-4.90168	27.54281	10.14976	30242	24	183	4	2.185792	1.080886	4842|4843|4846|5465	NOS1|NOS2|NOS3|PPARA	-3.68342	0	108	0	0	-7.19763	49.57704918
1	-4.90168	GO:1900373	M1	1	0	GO Biological Processes	19	positive regulation of purine nucleotide biosynthetic process	-4.90168	27.54281	10.14976	30242	24	183	4	2.185792	1.080886	4842|4843|4846|5465	NOS1|NOS2|NOS3|PPARA	-3.68342	0	108	0	0	-7.19763	49.57704918
1	-4.68993	GO:0007263	M1	1	0	GO Biological Processes	19	nitric oxide mediated signal transduction	-4.68993	24.48249	9.524697	30242	27	183	4	2.185792	1.080886	1956|4842|4843|4846	EGFR|NOS1|NOS2|NOS3	-3.49655	0	108	0	0	-7.19763	49.57704918
1	-4.596	GO:0031284	M1	1	0	GO Biological Processes	19	positive regulation of guanylate cyclase activity	-4.596	49.57705	11.98765	30242	10	183	3	1.639344	0.938686	4842|4843|4846	NOS1|NOS2|NOS3	-3.4183	0	108	0	0	-7.19763	49.57704918
1	-4.45963	GO:0006527	M1	1	0	GO Biological Processes	19	arginine catabolic process	-4.45963	45.07004	11.40644	30242	11	183	3	1.639344	0.938686	4842|4843|4846	NOS1|NOS2|NOS3	-3.30278	0	108	0	0	-7.19763	49.57704918
1	-4.22463	GO:0031282	M1	1	0	GO Biological Processes	19	regulation of guanylate cyclase activity	-4.22463	38.13619	10.44945	30242	13	183	3	1.639344	0.938686	4842|4843|4846	NOS1|NOS2|NOS3	-3.09123	0	108	0	0	-7.19763	49.57704918
1	-3.98232	GO:0046395	M1	1	0	GO Biological Processes	19	carboxylic acid catabolic process	-3.98232	5.57829	5.52099	30242	237	183	8	4.371585	1.511428	1268|2166|4842|4843|4846|5444|5465|5467	CNR1|FAAH|NOS1|NOS2|NOS3|PON1|PPARA|PPARD	-2.87127	0	108	0	0	-7.19763	49.57704918
1	-3.95744	GO:1900371	M1	1	0	GO Biological Processes	19	regulation of purine nucleotide biosynthetic process	-3.95744	16.12262	7.560397	30242	41	183	4	2.185792	1.080886	4842|4843|4846|5465	NOS1|NOS2|NOS3|PPARA	-2.8503	0	108	0	0	-7.19763	49.57704918
1	-3.93862	GO:0017014	M1	1	0	GO Biological Processes	19	protein nitrosylation	-3.93862	30.98566	9.360942	30242	16	183	3	1.639344	0.938686	1636|4842|4843	ACE|NOS1|NOS2	-2.83536	0	108	0	0	-7.19763	49.57704918
1	-3.93862	GO:0018119	M1	1	0	GO Biological Processes	19	peptidyl-cysteine S-nitrosylation	-3.93862	30.98566	9.360942	30242	16	183	3	1.639344	0.938686	1636|4842|4843	ACE|NOS1|NOS2	-2.83536	0	108	0	0	-7.19763	49.57704918
1	-3.93217	GO:0016054	M1	1	0	GO Biological Processes	19	organic acid catabolic process	-3.93217	5.485704	5.455162	30242	241	183	8	4.371585	1.511428	1268|2166|4842|4843|4846|5444|5465|5467	CNR1|FAAH|NOS1|NOS2|NOS3|PON1|PPARA|PPARD	-2.82961	0	108	0	0	-7.19763	49.57704918
1	-3.91602	GO:0030808	M1	1	0	GO Biological Processes	19	regulation of nucleotide biosynthetic process	-3.91602	15.73875	7.457926	30242	42	183	4	2.185792	1.080886	4842|4843|4846|5465	NOS1|NOS2|NOS3|PPARA	-2.81521	0	108	0	0	-7.19763	49.57704918
1	-3.63764	GO:0006525	M1	1	0	GO Biological Processes	19	arginine metabolic process	-3.63764	24.78852	8.303425	30242	20	183	3	1.639344	0.938686	4842|4843|4846	NOS1|NOS2|NOS3	-2.56974	0	108	0	0	-7.19763	49.57704918

<table>
<tr><td>1</td><td>-3.19466</td><td>GO:0009065</td><td>M1</td><td>1</td><td>0</td><td>GO Biological Processes</td><td>19</td><td>glutamine family amino acid catabolic process</td><td>-3.19466</td><td>17.70609</td><td>6.900587</td><td>30242</td><td>28</td><td>183</td><td>3</td><td>1.639344</td><td>0.938686</td><td>4842|4843|4846</td><td>NOS1|NOS2|NOS3</td><td>-2.17099</td><td>0</td><td>108</td><td>0</td><td>0</td><td>-7.19763</td><td>49.57704918</td></tr>
<tr><td>1</td><td>-2.48503</td><td>GO:0018198</td><td>M1</td><td>1</td><td>0</td><td>GO Biological Processes</td><td>19</td><td>peptidyl-cysteine modification</td><td>-2.48503</td><td>10.11777</td><td>4.983904</td><td>30242</td><td>49</td><td>183</td><td>3</td><td>1.639344</td><td>0.938686</td><td>1636|4842|4843</td><td>ACE|NOS1|NOS2</td><td>-1.53506</td><td>0</td><td>108</td><td>0</td><td>0</td><td>-7.19763</td><td>49.57704918</td></tr>
<tr><td>1</td><td>-7.16868</td><td>GO:0045787</td><td>M1</td><td>1</td><td>0</td><td>GO Biological Processes</td><td>19</td><td>positive regulation of cell cycle</td><td>-7.16868</td><td>6.863702</td><td>8.136438</td><td>30242</td><td>313</td><td>183</td><td>13</td><td>7.103825</td><td>1.898976</td><td>328|351|595|1543|1813|1814|1956|2263|3643|5159|6532|9475|225689</td><td>APEX1|APP|CCND1|CYP1A1|DRD2|DRD3|EGFR|FGFR2|INSR|PDGFRB|SLC6A4|ROCK2|MAPK15</td><td>-5.71699</td><td>0</td><td>109</td><td>1</td><td>1</td><td>-7.16868</td><td>14.37015918</td></tr>
<tr><td>1</td><td>-6.64441</td><td>GO:0090068</td><td>M1</td><td>1</td><td>0</td><td>GO Biological Processes</td><td>19</td><td>positive regulation of cell cycle process</td><td>-6.64441</td><td>7.702649</td><td>8.065565</td><td>30242</td><td>236</td><td>183</td><td>11</td><td>6.010929</td><td>1.757049</td><td>328|351|595|1543|1813|1814|1956|3643|5159|9475|225689</td><td>APEX1|APP|CCND1|CYP1A1|DRD2|DRD3|EGFR|INSR|PDGFRB|ROCK2|MAPK15</td><td>-5.24291</td><td>0</td><td>109</td><td>0</td><td>0</td><td>-7.16868</td><td>14.37015918</td></tr>
<tr><td>1</td><td>-4.13251</td><td>GO:1901989</td><td>M1</td><td>1</td><td>0</td><td>GO Biological Processes</td><td>19</td><td>positive regulation of cell cycle phase transition</td><td>-4.13251</td><td>8.622096</td><td>6.389718</td><td>30242</td><td>115</td><td>183</td><td>6</td><td>3.278689</td><td>1.316393</td><td>328|351|595|1543|1956|225689</td><td>APEX1|APP|CCND1|CYP1A1|EGFR|MAPK15</td><td>-3.00693</td><td>0</td><td>109</td><td>0</td><td>0</td><td>-7.16868</td><td>14.37015918</td></tr>
<tr><td>1</td><td>-3.76048</td><td>GO:1900087</td><td>M1</td><td>1</td><td>0</td><td>GO Biological Processes</td><td>19</td><td>positive regulation of G1/S transition of mitotic cell cycle</td><td>-3.76048</td><td>14.37016</td><td>7.080715</td><td>30242</td><td>46</td><td>183</td><td>4</td><td>2.185792</td><td>1.080886</td><td>328|595|1543|1956</td><td>APEX1|CCND1|CYP1A1|EGFR</td><td>-2.67844</td><td>0</td><td>109</td><td>0</td><td>0</td><td>-7.16868</td><td>14.37015918</td></tr>
<tr><td>1</td><td>-3.58581</td><td>GO:1901992</td><td>M1</td><td>1</td><td>0</td><td>GO Biological Processes</td><td>19</td><td>positive regulation of mitotic cell cycle phase transition</td><td>-3.58581</td><td>8.884776</td><td>5.941974</td><td>30242</td><td>93</td><td>183</td><td>5</td><td>2.73224</td><td>1.205087</td><td>328|351|595|1543|1956</td><td>APEX1|APP|CCND1|CYP1A1|EGFR</td><td>-2.5219</td><td>0</td><td>109</td><td>0</td><td>0</td><td>-7.16868</td><td>14.37015918</td></tr>
<tr><td>1</td><td>-3.37005</td><td>GO:1902808</td><td>M1</td><td>1</td><td>0</td><td>GO Biological Processes</td><td>19</td><td>positive regulation of cell cycle G1/S phase transition</td><td>-3.37005</td><td>11.39702</td><td>6.184021</td><td>30242</td><td>58</td><td>183</td><td>4</td><td>2.185792</td><td>1.080886</td><td>328|595|1543|1956</td><td>APEX1|CCND1|CYP1A1|EGFR</td><td>-2.32819</td><td>0</td><td>109</td><td>0</td><td>0</td><td>-7.16868</td><td>14.37015918</td></tr>
<tr><td>1</td><td>-3.06223</td><td>GO:0045931</td><td>M1</td><td>1</td><td>0</td><td>GO Biological Processes</td><td>19</td><td>positive regulation of mitotic cell cycle</td><td>-3.06223</td><td>6.828795</td><td>5.012716</td><td>30242</td><td>121</td><td>183</td><td>5</td><td>2.73224</td><td>1.205087</td><td>328|351|595|1543|1956</td><td>APEX1|APP|CCND1|CYP1A1|EGFR</td><td>-2.05405</td><td>0</td><td>109</td><td>0</td><td>0</td><td>-7.16868</td><td>14.37015918</td></tr>
<tr><td>1</td><td>-2.64955</td><td>GO:0051592</td><td>M1</td><td>1</td><td>0</td><td>GO Biological Processes</td><td>19</td><td>response to calcium ion</td><td>-2.64955</td><td>5.508561</td><td>4.319111</td><td>30242</td><td>150</td><td>183</td><td>5</td><td>2.73224</td><td>1.205087</td><td>595|673|1956|3778|6331</td><td>CCND1|BRAF|EGFR|KCNMA1|SCN5A</td><td>-1.68325</td><td>0</td><td>109</td><td>0</td><td>0</td><td>-7.16868</td><td>14.37015918</td></tr>
<tr><td>1</td><td>-7.07896</td><td>GO:0003085</td><td>M1</td><td>1</td><td>0</td><td>GO Biological Processes</td><td>19</td><td>negative regulation of systemic arterial blood pressure</td><td>-7.07896</td><td>43.48864</td><td>14.45497</td><td>30242</td><td>19</td><td>183</td><td>5</td><td>2.73224</td><td>1.205087</td><td>134|148|153|154|7442</td><td>ADORA1|ADRA1A|ADRB1|ADRB2|TRPV1</td><td>-5.63348</td><td>0</td><td>110</td><td>1</td><td>1</td><td>-7.07896</td><td>43.48863963</td></tr>
<tr><td>1</td><td>-4.82801</td><td>GO:0045822</td><td>M1</td><td>1</td><td>0</td><td>GO Biological Processes</td><td>19</td><td>negative regulation of heart contraction</td><td>-4.82801</td><td>26.44109</td><td>9.92925</td><td>30242</td><td>25</td><td>183</td><td>4</td><td>2.185792</td><td>1.080886</td><td>134|148|3558|7442</td><td>ADORA1|ADRA1A|IL2|TRPV1</td><td>-3.61611</td><td>0</td><td>110</td><td>0</td><td>0</td><td>-7.07896</td><td>43.48863963</td></tr>
<tr><td>1</td><td>-4.75751</td><td>GO:1903523</td><td>M1</td><td>1</td><td>0</td><td>GO Biological Processes</td><td>19</td><td>negative regulation of blood circulation</td><td>-4.75751</td><td>25.42413</td><td>9.721283</td><td>30242</td><td>26</td><td>183</td><td>4</td><td>2.185792</td><td>1.080886</td><td>134|148|3558|7442</td><td>ADORA1|ADRA1A|IL2|TRPV1</td><td>-3.55409</td><td>0</td><td>110</td><td>0</td><td>0</td><td>-7.07896</td><td>43.48863963</td></tr>
<tr><td>1</td><td>-4.73339</td><td>GO:0061097</td><td>M1</td><td>1</td><td>0</td><td>GO Biological Processes</td><td>19</td><td>regulation of protein tyrosine kinase activity</td><td>-4.73339</td><td>11.01712</td><td>7.425787</td><td>30242</td><td>90</td><td>183</td><td>6</td><td>3.278689</td><td>1.316393</td><td>134|148|150|351|1636|2915</td><td>ADORA1|ADRA1A|ADRA2A|APP|ACE|GRM5</td><td>-3.53566</td><td>0</td><td>110</td><td>0</td><td>0</td><td>-7.07896</td><td>43.48863963</td></tr>
<tr><td>1</td><td>-4.67964</td><td>GO:0061098</td><td>M1</td><td>1</td><td>0</td><td>GO Biological Processes</td><td>19</td><td>positive regulation of protein tyrosine kinase activity</td><td>-4.67964</td><td>15.02335</td><td>8.121934</td><td>30242</td><td>55</td><td>183</td><td>5</td><td>2.73224</td><td>1.205087</td><td>134|148|150|1636|2915</td><td>ADORA1|ADRA1A|ADRA2A|ACE|GRM5</td><td>-3.48755</td><td>0</td><td>110</td><td>0</td><td>0</td><td>-7.07896</td><td>43.48863963</td></tr>
<tr><td>1</td><td>-3.93862</td><td>GO:0003084</td><td>M1</td><td>1</td><td>0</td><td>GO Biological Processes</td><td>19</td><td>positive regulation of systemic arterial blood pressure</td><td>-3.93862</td><td>30.98566</td><td>9.360942</td><td>30242</td><td>16</td><td>183</td><td>3</td><td>1.639344</td><td>0.938686</td><td>134|148|1636</td><td>ADORA1|ADRA1A|ACE</td><td>-2.83536</td><td>0</td><td>110</td><td>0</td><td>0</td><td>-7.07896</td><td>43.48863963</td></tr>
<tr><td>1</td><td>-7.07236</td><td>GO:1902806</td><td>M1</td><td>1</td><td>0</td><td>GO Biological Processes</td><td>19</td><td>regulation of cell cycle G1/S phase transition</td><td>-7.07236</td><td>9.836716</td><td>8.961615</td><td>30242</td><td>168</td><td>183</td><td>10</td><td>5.464481</td><td>1.680144</td><td>328|472|595|596|1543|1956|4221|5591|6347|7157</td><td>APEX1|ATM|CCND1|BCL2|CYP1A1|EGFR|MEN1|PRKDC|CCL2|TP53</td><td>-5.62843</td><td>0</td><td>111</td><td>1</td><td>1</td><td>-7.07236</td><td>82.6284153</td></tr>
<tr><td>1</td><td>-6.81378</td><td>GO:0007346</td><td>M1</td><td>1</td><td>0</td><td>GO Biological Processes</td><td>19</td><td>regulation of mitotic cell cycle</td><td>-6.81378</td><td>5.38881</td><td>7.400909</td><td>30242</td><td>460</td><td>183</td><td>15</td><td>8.196721</td><td>2.027793</td><td>328|351|472|595|596|1543|1814|1956|3643|515</td><td>APEX1|APP|ATM|CCND1|BCL2|CYP1A1|D</td><td>-5.3945</td><td>0</td><td>111</td><td>0</td><td>0</td><td>-7.07236</td><td>82.6284153</td></tr>
</table>

																		9|5591|6347|7040|7124|7157	RD3|EGFR|INSR|PDGFRB|PRKDC|CCL2|TGFB1|TNF|TP53							
1	-6.67276	GO:0097193	M1	1	0	GO Biological Processes	19	intrinsic apoptotic signaling pathway	-6.67276	6.885701	7.830751	30242	288	183	12	6.557377	1.829835	472|596|624|836|1545|4318|5591|5743|6326|7124|7132|7157	ATM|BCL2|BDKRB2|CASP3|CYP1B1|MMP9|PRKDC|PTGS2|SCN2A|TNF|TNFRSF1A|TP53	-5.26636	0	111	0	0	-7.07236	82.6284153
1	-6.65072	GO:2000045	M1	1	0	GO Biological Processes	19	regulation of G1/S transition of mitotic cell cycle	-6.65072	10.47402	8.829412	30242	142	183	9	4.918033	1.598524	328|472|595|596|1543|1956|5591|6347|7157	APEX1|ATM|CCND1|BCL2|CYP1A1|EGFR|PRKDC|CCL2|TP53	-5.24783	0	111	0	0	-7.07236	82.6284153
1	-6.55342	GO:0044843	M1	1	0	GO Biological Processes	19	cell cycle G1/S phase transition	-6.55342	7.542843	7.956893	30242	241	183	11	6.010929	1.757049	328|472|595|596|890|1543|1956|4221|5591|6347|7157	APEX1|ATM|CCND1|BCL2|CCNA2|CYP1A1|EGFR|MEN1|PRKDC|CCL2|TP53	-5.16039	0	111	0	0	-7.07236	82.6284153
1	-6.47299	GO:0010212	M1	1	0	GO Biological Processes	19	response to ionizing radiation	-6.47299	9.981956	8.575662	30242	149	183	9	4.918033	1.598524	472|595|596|836|3383|4221|5591|7157|10135	ATM|CCND1|BCL2|CASP3|ICAM1|MEN1|PRKDC|TP53|NAMPT	-5.08589	0	111	0	0	-7.07236	82.6284153
1	-6.28001	GO:0010564	M1	1	0	GO Biological Processes	19	regulation of cell cycle process	-6.28001	4.308844	6.664444	30242	652	183	17	9.289617	2.145862	328|351|472|595|596|1543|1813|1814|1956|3643|4221|5159|5591|6347|7157|9475|225689	APEX1|APP|ATM|CCND1|BCL2|CYP1A1|DRD2|DRD3|EGFR|INSR|MEN1|PDGFRB|PRKDC|CCL2|TP53|ROCK2|MAPK15	-4.9062	0	111	0	0	-7.07236	82.6284153
1	-5.79102	GO:1902807	M1	1	0	GO Biological Processes	19	negative regulation of cell cycle G1/S phase transition	-5.79102	12.43869	8.620203	30242	93	183	7	3.825137	1.417845	472|595|596|4221|5591|6347|7157	ATM|CCND1|BCL2|MEN1|PRKDC|CCL2|TP53	-4.46309	0	111	0	0	-7.07236	82.6284153
1	-5.6405	GO:0051052	M1	1	0	GO Biological Processes	19	regulation of DNA metabolic process	-5.6405	5.493302	6.701283	30242	361	183	12	6.557377	1.829835	472|1956|3558|3569|4221|5159|5290|5582|5591|7040|7157|225689	ATM|EGFR|IL2|IL6|MEN1|PDGFRB|PIK3CA|PRKCG|PRKDC|TGFB1|TP53|MAPK15	-4.32874	0	111	0	0	-7.07236	82.6284153
1	-5.43765	GO:0044770	M1	1	0	GO Biological Processes	19	cell cycle phase transition	-5.43765	4.466401	6.209068	30242	518	183	14	7.650273	1.964856	328|351|472|595|596|890|1543|1956|4221|5243|5591|6347|7157|225689	APEX1|APP|ATM|CCND1|BCL2|CCNA2|CYP1A1|EGFR|MEN1|ABCB1|PRKDC|CCL2|TP53|MAPK15	-4.14584	0	111	0	0	-7.07236	82.6284153
1	-5.39158	GO:0051054	M1	1	0	GO Biological Processes	19	positive regulation of DNA metabolic process	-5.39158	7.39956	7.102762	30242	201	183	9	4.918033	1.598524	472|1956|3558|3569|5159|5582|5591|7040|225689	ATM|EGFR|IL2|IL6|PDGFRB|PRKCG|PRKDC|TGFB1|MAPK15	-4.10515	0	111	0	0	-7.07236	82.6284153
1	-5.36639	GO:0002326	M1	1	0	GO Biological Processes	19	B cell lineage commitment	-5.36639	82.62842	15.60239	30242	6	183	3	1.639344	0.938686	596|5591|7157	BCL2|PRKDC|TP53	-4.08918	0	111	0	0	-7.07236	82.6284153
1	-5.35446	GO:0045786	M1	1	0	GO Biological Processes	19	negative regulation of cell cycle	-5.35446	5.150862	6.39561	30242	385	183	12	6.557377	1.829835	472|595|596|836|1956|4221|5591|5743|6347|7040|7124|7157	ATM|CCND1|BCL2|CASP3|EGFR|MEN1|PRKDC|PTGS2|CCL2|TGFB1|TNF|TP53	-4.07808	0	111	0	0	-7.07236	82.6284153
1	-5.29759	GO:1901987	M1	1	0	GO Biological Processes	19	regulation of cell cycle phase transition	-5.29759	5.084826	6.335129	30242	390	183	12	6.557377	1.829835	328|351|472|595|596|1543|1956|4221|5591|6347|7157|225689	APEX1|APP|ATM|CCND1|BCL2|CYP1A1|EGFR|MEN1|PRKDC|CCL2|TP53|MAPK15	-4.02846	0	111	0	0	-7.07236	82.6284153
1	-5.17092	GO:0000082	M1	1	0	GO Biological Processes	19	G1/S transition of mitotic cell cycle	-5.17092	6.950054	6.81555	30242	214	183	9	4.918033	1.598524	328|472|595|596|1543|1956|5591|6347|7157	APEX1|ATM|CCND1|BCL2|CYP1A1|EGFR|	-3.91695	0	111	0	0	-7.07236	82.6284153

																			PRKDC|CCL2|TP53							
1	-5.01333	GO:1903047	M1	1	0	GO Biological Processes	19	mitotic cell cycle process	-5.01333	3.642024	5.622282	30242	726	183	16	8.743169	2.088053	328|351|472|595|596|890|1543|1814|1956|3643|5159|5243|5591|6347|7157|9475	APEX1|APP|ATM|CCND1|BCL2|CCNA2|CYP1A1|DRD3|EGFR|INSR|PDGFRB|ABCB1|PRKDC|CCL2|TP53|ROCK2	-3.77591	0	111	0	0	-7.07236	82.6284153
1	-4.93309	GO:0044772	M1	1	0	GO Biological Processes	19	mitotic cell cycle phase transition	-4.93309	4.67708	5.949536	30242	424	183	12	6.557377	1.829835	328|351|472|595|596|890|1543|1956|5243|5591|6347|7157	APEX1|APP|ATM|CCND1|BCL2|CCNA2|CYP1A1|EGFR|ABCB1|PRKDC|CCL2|TP53	-3.70605	0	111	0	0	-7.07236	82.6284153
1	-4.90554	GO:2000134	M1	1	0	GO Biological Processes	19	negative regulation of G1/S transition of mitotic cell cycle	-4.90554	11.80406	7.736806	30242	84	183	6	3.278689	1.316393	472|595|596|5591|6347|7157	ATM|CCND1|BCL2|PRKDC|CCL2|TP53	-3.68453	0	111	0	0	-7.07236	82.6284153
1	-4.84541	GO:0045930	M1	1	0	GO Biological Processes	19	negative regulation of mitotic cell cycle	-4.84541	6.328985	6.398873	30242	235	183	9	4.918033	1.598524	472|595|596|1956|5591|6347|7040|7124|7157	ATM|CCND1|BCL2|EGFR|PRKDC|CCL2|TGFB1|TNF|TP53	-3.63125	0	111	0	0	-7.07236	82.6284153
1	-4.81178	GO:1901990	M1	1	0	GO Biological Processes	19	regulation of mitotic cell cycle phase transition	-4.81178	5.526984	6.13809	30242	299	183	10	5.464481	1.680144	328|351|472|595|596|1543|1956|5591|6347|7157	APEX1|APP|ATM|CCND1|BCL2|CYP1A1|EGFR|PRKDC|CCL2|TP53	-3.60257	0	111	0	0	-7.07236	82.6284153
1	-4.64115	GO:0010332	M1	1	0	GO Biological Processes	19	response to gamma radiation	-4.64115	14.75507	8.038787	30242	56	183	5	2.73224	1.205087	472|596|4221|5591|7157	ATM|BCL2|MEN1|PRKDC|TP53	-3.45334	0	111	0	0	-7.07236	82.6284153
1	-4.56263	GO:0002360	M1	1	0	GO Biological Processes	19	T cell lineage commitment	-4.56263	22.79405	9.161705	30242	29	183	4	2.185792	1.080886	596|3569|5591|7157	BCL2|IL6|PRKDC|TP53	-3.38949	0	111	0	0	-7.07236	82.6284153
1	-4.50254	GO:0031571	M1	1	0	GO Biological Processes	19	mitotic G1 DNA damage checkpoint signaling	-4.50254	22.03424	8.993612	30242	30	183	4	2.185792	1.080886	472|595|5591|7157	ATM|CCND1|PRKDC|TP53	-3.33837	0	111	0	0	-7.07236	82.6284153
1	-4.4977	GO:0008630	M1	1	0	GO Biological Processes	19	intrinsic apoptotic signaling pathway in response to DNA damage	-4.4977	10.01557	7.010576	30242	99	183	6	3.278689	1.316393	472|596|5591|7124|7132|7157	ATM|BCL2|PRKDC|TNF|TNFRSF1A|TP53	-3.33635	0	111	0	0	-7.07236	82.6284153
1	-4.44459	GO:0044819	M1	1	0	GO Biological Processes	19	mitotic G1/S transition checkpoint signaling	-4.44459	21.32346	8.83349	30242	31	183	4	2.185792	1.080886	472|595|5591|7157	ATM|CCND1|PRKDC|TP53	-3.29079	0	111	0	0	-7.07236	82.6284153
1	-4.33663	GO:0010623	M1	1	0	GO Biological Processes	19	programmed cell death involved in cell development	-4.33663	41.31421	10.89848	30242	12	183	3	1.639344	0.938686	596|4914|5591	BCL2|NTRK1|PRKDC	-3.18987	0	111	0	0	-7.07236	82.6284153
1	-4.33459	GO:0010165	M1	1	0	GO Biological Processes	19	response to X-ray	-4.33459	20.03113	8.534729	30242	33	183	4	2.185792	1.080886	472|595|836|7157	ATM|CCND1|CASP3|TP53	-3.18937	0	111	0	0	-7.07236	82.6284153
1	-3.80251	GO:0007569	M1	1	0	GO Biological Processes	19	cell aging	-3.80251	7.511674	5.850064	30242	132	183	6	3.278689	1.316393	472|596|3383|5591|7157|10135	ATM|BCL2|ICAM1|PRKDC|TP53|NAMPT	-2.71238	0	111	0	0	-7.07236	82.6284153
1	-3.61905	GO:0051972	M1	1	0	GO Biological Processes	19	regulation of telomerase activity	-3.61905	13.22055	6.74786	30242	50	183	4	2.185792	1.080886	472|4221|7157|225689	ATM|MEN1|TP53|MAPK15	-2.55243	0	111	0	0	-7.07236	82.6284153
1	-3.2497	GO:2000278	M1	1	0	GO Biological Processes	19	regulation of DNA biosynthetic process	-3.2497	7.511674	5.338403	30242	110	183	5	2.73224	1.205087	472|4221|5159|7157|225689	ATM|MEN1|PDGFRB|TP53|MAPK15	-2.21986	0	111	0	0	-7.07236	82.6284153
1	-3.17915	GO:0002244	M1	1	0	GO Biological Processes	19	hematopoietic progenitor cell differentiation	-3.17915	7.248107	5.214973	30242	114	183	5	2.73224	1.205087	596|1636|5591|7040|7157	BCL2|ACE|PRKDC|TGFB1|TP53	-2.15752	0	111	0	0	-7.07236	82.6284153
1	-3.10534	GO:0060218	M1	1	0	GO Biological Processes	19	hematopoietic stem cell differentiation	-3.10534	16.52568	6.638317	30242	30	183	3	1.639344	0.938686	1636|5591|7157	ACE|PRKDC|TP53	-2.09149	0	111	0	0	-7.07236	82.6284153
1	-3.09693	GO:1901991	M1	1	0	GO Biological	19	negative regulation of	-3.09693	5.539335	4.752684	30242	179	183	6	3.278689	1.316393	472|595|596|5591|6347|	ATM|CCND1|BCL2|P	-2.08365	0	111	0	0	-7.07236	82.6284153

						Processes		mitotic cell cycle phase transition										7157	RKDC|CCL2|TP53							
1	-3.0761	GO:1901988	M1	1	0	GO Biological Processes	19	negative regulation of cell cycle phase transition	-3.0761	4.645774	4.507294	30242	249	183	7	3.825137	1.417845	472|595|596|4221|5591|6347|7157	ATM|CCND1|BCL2|MEN1|PRKDC|CCL2|TP53	-2.06538	0	111	0	0	-7.07236	82.6284153
1	-3.03489	GO:0071897	M1	1	0	GO Biological Processes	19	DNA biosynthetic process	-3.03489	5.38881	4.659207	30242	184	183	6	3.278689	1.316393	472|4221|5159|7157|7298|225689	ATM|MEN1|PDGFRB|TP53|TYMS|MAPK15	-2.02783	0	111	0	0	-7.07236	82.6284153
1	-2.94761	GO:0033077	M1	1	0	GO Biological Processes	19	T cell differentiation in thymus	-2.94761	8.813698	5.28637	30242	75	183	4	2.185792	1.080886	100|596|5591|7157	ADA|BCL2|PRKDC|TP53	-1.94919	0	111	0	0	-7.07236	82.6284153
1	-2.91395	GO:0006974	M1	1	0	GO Biological Processes	19	cellular response to DNA damage stimulus	-2.91395	2.688784	3.774791	30242	799	183	13	7.103825	1.898976	328|472|595|596|836|1956|4221|5582|5591|7124|7132|7157|225689	APEX1|ATM|CCND1|BCL2|CASP3|EGFR|MEN1|PRKCG|PRKDC|TNF|TNFRSF1A|TP53|MAPK15	-1.91827	0	111	0	0	-7.07236	82.6284153
1	-2.82347	GO:0044773	M1	1	0	GO Biological Processes	19	mitotic DNA damage checkpoint signaling	-2.82347	8.160831	5.035231	30242	81	183	4	2.185792	1.080886	472|595|5591|7157	ATM|CCND1|PRKDC|TP53	-1.83834	0	111	0	0	-7.07236	82.6284153
1	-2.77031	GO:0016233	M1	1	0	GO Biological Processes	19	telomere capping	-2.77031	12.71206	5.710538	30242	39	183	3	1.639344	0.938686	472|5591|225689	ATM|PRKDC|MAPK15	-1.79128	0	111	0	0	-7.07236	82.6284153
1	-2.74631	GO:0044774	M1	1	0	GO Biological Processes	19	mitotic DNA integrity checkpoint signaling	-2.74631	7.776792	4.881754	30242	85	183	4	2.185792	1.080886	472|595|5591|7157	ATM|CCND1|PRKDC|TP53	-1.76965	0	111	0	0	-7.07236	82.6284153
1	-2.66348	GO:0010948	M1	1	0	GO Biological Processes	19	negative regulation of cell cycle process	-2.66348	3.934686	3.945371	30242	294	183	7	3.825137	1.417845	472|595|596|4221|5591|6347|7157	ATM|CCND1|BCL2|MEN1|PRKDC|CCL2|TP53	-1.69513	0	111	0	0	-7.07236	82.6284153
1	-2.65052	GO:2001020	M1	1	0	GO Biological Processes	19	regulation of response to DNA damage stimulus	-2.65052	4.527584	4.08798	30242	219	183	6	3.278689	1.316393	472|596|1956|5582|5591|7157	ATM|BCL2|EGFR|PRKCG|PRKDC|TP53	-1.6837	0	111	0	0	-7.07236	82.6284153
1	-2.60359	GO:0090398	M1	1	0	GO Biological Processes	19	cellular senescence	-2.60359	7.107821	4.602859	30242	93	183	4	2.185792	1.080886	472|5591|7157|10135	ATM|PRKDC|TP53|NAMPT	-1.64263	0	111	0	0	-7.07236	82.6284153
1	-2.59076	GO:0048538	M1	1	0	GO Biological Processes	19	thymus development	-2.59076	11.01712	5.246911	30242	45	183	3	1.639344	0.938686	596|5591|7046	BCL2|PRKDC|TGFBR1	-1.63232	0	111	0	0	-7.07236	82.6284153
1	-2.41379	GO:2001022	M1	1	0	GO Biological Processes	19	positive regulation of response to DNA damage stimulus	-2.41379	6.295498	4.241191	30242	105	183	4	2.185792	1.080886	472|1956|5582|5591	ATM|EGFR|PRKCG|PRKDC	-1.46994	0	111	0	0	-7.07236	82.6284153
1	-2.38839	GO:2000772	M1	1	0	GO Biological Processes	19	regulation of cellular senescence	-2.38839	9.35416	4.749553	30242	53	183	3	1.639344	0.938686	5591|7157|10135	PRKDC|TP53|NAMPT	-1.44695	0	111	0	0	-7.07236	82.6284153
1	-2.27377	GO:0000077	M1	1	0	GO Biological Processes	19	DNA damage checkpoint signaling	-2.27377	5.748064	3.980374	30242	115	183	4	2.185792	1.080886	472|595|5591|7157	ATM|CCND1|PRKDC|TP53	-1.3419	0	111	0	0	-7.07236	82.6284153
1	-2.21721	GO:0090342	M1	1	0	GO Biological Processes	19	regulation of cell aging	-2.21721	8.127385	4.347751	30242	61	183	3	1.639344	0.938686	5591|7157|10135	PRKDC|TP53|NAMPT	-1.29004	0	111	0	0	-7.07236	82.6284153
1	-2.17158	GO:0031570	M1	1	0	GO Biological Processes	19	DNA integrity checkpoint signaling	-2.17158	5.374206	3.792869	30242	123	183	4	2.185792	1.080886	472|595|5591|7157	ATM|CCND1|PRKDC|TP53	-1.24698	0	111	0	0	-7.07236	82.6284153
1	-2.12246	GO:2000573	M1	1	0	GO Biological Processes	19	positive regulation of DNA biosynthetic process	-2.12246	7.511674	4.132094	30242	66	183	3	1.639344	0.938686	472|5159|225689	ATM|PDGFRB|MAPK15	-1.20133	0	111	0	0	-7.07236	82.6284153
1	-2.09993	GO:0007093	M1	1	0	GO Biological Processes	19	mitotic cell cycle checkpoint signaling	-2.09993	5.124243	3.662675	30242	129	183	4	2.185792	1.080886	472|595|5591|7157	ATM|CCND1|PRKDC|TP53	-1.18041	0	111	0	0	-7.07236	82.6284153
1	-2.07692	GO:0000723	M1	1	0	GO Biological Processes	19	telomere maintenance	-2.07692	5.04601	3.621065	30242	131	183	4	2.185792	1.080886	328|472|5591|225689	APEX1|ATM|PRKDC|MAPK15	-1.16015	0	111	0	0	-7.07236	82.6284153
1	-2.01885	GO:0071479	M1	1	0	GO Biological Processes	19	cellular response to ionizing radiation	-2.01885	6.885701	3.901334	30242	72	183	3	1.639344	0.938686	472|7157|10135	ATM|TP53|NAMPT	-1.10504	0	111	0	0	-7.07236	82.6284153

1	-2.00253	GO:0045739	M1	1	0	GO Biological Processes	19	positive regulation of DNA repair	-2.00253	6.791377	3.865442	30242	73	183	3	1.639344	0.938686	1956|5582|5591	EGFR|PRKCG|PRKDC	-1.09008	0	111	0	0	-7.07236	82.6284153
1	-7.05708	GO:0040014	M1	1	0	GO Biological Processes	19	regulation of multicellular organism growth	-7.05708	18.9639	10.95809	30242	61	183	7	3.825137	1.417845	153|154|351|596|1813|5290|6531	ADRB1|ADRB2|APP|BCL2|DRD2|PIK3CA|SLC6A3	-5.61468	0	112	1	1	-7.05708	25.82137978
1	-6.92237	GO:0035264	M1	1	0	GO Biological Processes	19	multicellular organism growth	-6.92237	11.26751	9.224294	30242	132	183	9	4.918033	1.598524	153|154|351|367|596|1813|5290|6531|7157	ADRB1|ADRB2|APP|AR|BCL2|DRD2|PIK3CA|SLC6A3|TP53	-5.49206	0	112	0	0	-7.05708	25.82137978
1	-5.86815	GO:0010039	M1	1	0	GO Biological Processes	19	response to iron ion	-5.86815	25.82138	10.9613	30242	32	183	5	2.73224	1.205087	595|596|1543|1813|6531	CCND1|BCL2|CYP1A1|DRD2|SLC6A3	-4.53728	0	112	0	0	-7.05708	25.82137978
1	-3.14917	GO:0040018	M1	1	0	GO Biological Processes	19	positive regulation of multicellular organism growth	-3.14917	17.09553	6.766184	30242	29	183	3	1.639344	0.938686	596|1813|6531	BCL2|DRD2|SLC6A3	-2.13101	0	112	0	0	-7.05708	25.82137978
1	-2.96803	GO:0035270	M1	1	0	GO Biological Processes	19	endocrine system development	-2.96803	6.506174	4.851731	30242	127	183	5	2.73224	1.205087	1813|3569|4221|6531|7046	DRD2|IL6|MEN1|SLC6A3|TGFBR1	-1.96823	0	112	0	0	-7.05708	25.82137978
1	-7.04813	GO:0031214	M1	1	0	GO Biological Processes	19	biomineral tissue development	-7.04813	9.778511	8.929192	30242	169	183	10	5.464481	1.680144	154|249|1080|1230|2263|4846|5465|5743|7040|9475	ADRB2|ALPL|CFTR|CCR1|FGFR2|NOS3|PPARA|PTGS2|TGFB1|ROCK2	-5.60955	0	113	1	1	-7.04813	22.03424408
1	-7.00015	GO:0110148	M1	1	0	GO Biological Processes	19	biomineralization	-7.00015	9.664142	8.865149	30242	171	183	10	5.464481	1.680144	154|249|1080|1230|2263|4846|5465|5743|7040|9475	ADRB2|ALPL|CFTR|CCR1|FGFR2|NOS3|PPARA|PTGS2|TGFB1|ROCK2	-5.56535	0	113	0	0	-7.04813	22.03424408
1	-4.54796	GO:0070167	M1	1	0	GO Biological Processes	19	regulation of biomineral tissue development	-4.54796	10.22207	7.098116	30242	97	183	6	3.278689	1.316393	154|1080|1230|4846|7040|9475	ADRB2|CFTR|CCR1|NOS3|TGFB1|ROCK2	-3.37728	0	113	0	0	-7.04813	22.03424408
1	-4.50254	GO:0070168	M1	1	0	GO Biological Processes	19	negative regulation of biomineral tissue development	-4.50254	22.03424	8.993612	30242	30	183	4	2.185792	1.080886	1230|4846|7040|9475	CCR1|NOS3|TGFB1|ROCK2	-3.33837	0	113	0	0	-7.04813	22.03424408
1	-4.4977	GO:0110149	M1	1	0	GO Biological Processes	19	regulation of biomineralization	-4.4977	10.01557	7.010576	30242	99	183	6	3.278689	1.316393	154|1080|1230|4846|7040|9475	ADRB2|CFTR|CCR1|NOS3|TGFB1|ROCK2	-3.33635	0	113	0	0	-7.04813	22.03424408
1	-4.44459	GO:0110150	M1	1	0	GO Biological Processes	19	negative regulation of biomineralization	-4.44459	21.32346	8.83349	30242	31	183	4	2.185792	1.080886	1230|4846|7040|9475	CCR1|NOS3|TGFB1|ROCK2	-3.29079	0	113	0	0	-7.04813	22.03424408
1	-4.32532	GO:0003170	M1	1	0	GO Biological Processes	19	heart valve development	-4.32532	12.71206	7.375448	30242	65	183	5	2.73224	1.205087	4846|5138|7040|7132|9475	NOS3|PDE2A|TGFB1|TNFRSF1A|ROCK2	-3.18203	0	113	0	0	-7.04813	22.03424408
1	-4.13489	GO:0003176	M1	1	0	GO Biological Processes	19	aortic valve development	-4.13489	17.8656	8.009394	30242	37	183	4	2.185792	1.080886	4846|7040|7132|9475	NOS3|TGFB1|TNFRSF1A|ROCK2	-3.00894	0	113	0	0	-7.04813	22.03424408
1	-4.0543	GO:0006801	M1	1	0	GO Biological Processes	19	superoxide metabolic process	-4.0543	11.166	6.831706	30242	74	183	5	2.73224	1.205087	1728|1956|4843|4846|7040	NQO1|EGFR|NOS2|NOS3|TGFB1	-2.93678	0	113	0	0	-7.04813	22.03424408
1	-3.95744	GO:1905314	M1	1	0	GO Biological Processes	19	semi-lunar valve development	-3.95744	16.12262	7.560397	30242	41	183	4	2.185792	1.080886	4846|7040|7132|9475	NOS3|TGFB1|TNFRSF1A|ROCK2	-2.8503	0	113	0	0	-7.04813	22.03424408
1	-3.76048	GO:0001974	M1	1	0	GO Biological Processes	19	blood vessel remodeling	-3.76048	14.37016	7.080715	30242	46	183	4	2.185792	1.080886	1636|4846|7040|729230	ACE|NOS3|TGFB1|CCR2	-2.67844	0	113	0	0	-7.04813	22.03424408
1	-3.16196	GO:0030278	M1	1	0	GO Biological Processes	19	regulation of ossification	-3.16196	7.18508	5.185046	30242	115	183	5	2.73224	1.205087	154|596|1230|5734|7040	ADRB2|BCL2|CCR1|PTGER4|TGFB1	-2.14148	0	113	0	0	-7.04813	22.03424408
1	-3.09483	GO:0030282	M1	1	0	GO Biological Processes	19	bone mineralization	-3.09483	6.943564	5.06883	30242	119	183	5	2.73224	1.205087	154|1230|2263|5743|7040	ADRB2|CCR1|FGFR2|PTGS2|TGFB1	-2.08212	0	113	0	0	-7.04813	22.03424408
1	-3.02224	GO:0003180	M1	1	0	GO Biological Processes	19	aortic valve morphogenesis	-3.02224	15.49283	6.400135	30242	32	183	3	1.639344	0.938686	4846|7040|9475	NOS3|TGFB1|ROCK2	-2.01659	0	113	0	0	-7.04813	22.03424408
1	-2.48503	GO:0070169	M1	1	0	GO Biological	19	positive regulation of	-2.48503	10.11777	4.983904	30242	49	183	3	1.639344	0.938686	154|1080|7040	ADRB2|CFTR|TGFB1	-1.53506	0	113	0	0	-7.04813	22.03424408

						Processes		biomineral tissue development																		
1	-2.46008	GO:0110151	M1	1	0	GO Biological Processes	19	positive regulation of biomineralization	-2.46008	9.91541	4.922851	30242	50	183	3	1.639344	0.938686	154|1080|7040	ADRB2|CFTR|TGFB1	-1.51158	0	113	0	0	-7.04813	22.03424408
1	-2.34304	GO:0003179	M1	1	0	GO Biological Processes	19	heart valve morphogenesis	-2.34304	9.014009	4.641491	30242	55	183	3	1.639344	0.938686	4846|7040|9475	NOS3|TGFB1|ROCK2	-1.40593	0	113	0	0	-7.04813	22.03424408
1	-6.95615	GO:0014047	M1	1	0	GO Biological Processes	19	glutamate secretion	-6.95615	41.31421	14.07174	30242	20	183	5	2.73224	1.205087	134|135|4915|7442|11255	ADORA1|ADORA2A|NTRK2|TRPV1|HRH3	-5.52285	0	114	1	1	-6.95615	41.31420765
1	-4.64115	GO:0015800	M1	1	0	GO Biological Processes	19	acidic amino acid transport	-4.64115	14.75507	8.038787	30242	56	183	5	2.73224	1.205087	134|135|4915|7442|11255	ADORA1|ADORA2A|NTRK2|TRPV1|HRH3	-3.45334	0	114	0	0	-6.95615	41.31420765
1	-4.26166	GO:0032890	M1	1	0	GO Biological Processes	19	regulation of organic acid transport	-4.26166	12.3326	7.245688	30242	67	183	5	2.73224	1.205087	134|135|3351|5465|11255	ADORA1|ADORA2A|HTR1B|PPARA|HRH3	-3.12258	0	114	0	0	-6.95615	41.31420765
1	-4.22463	GO:0051956	M1	1	0	GO Biological Processes	19	negative regulation of amino acid transport	-4.22463	38.13619	10.44945	30242	13	183	3	1.639344	0.938686	134|3351|11255	ADORA1|HTR1B|HRH3	-3.09123	0	114	0	0	-6.95615	41.31420765
1	-4.18252	GO:0051955	M1	1	0	GO Biological Processes	19	regulation of amino acid transport	-4.18252	18.36187	8.132751	30242	36	183	4	2.185792	1.080886	134|135|3351|11255	ADORA1|ADORA2A|HTR1B|HRH3	-3.05249	0	114	0	0	-6.95615	41.31420765
1	-4.02686	GO:0014048	M1	1	0	GO Biological Processes	19	regulation of glutamate secretion	-4.02686	33.05137	9.68793	30242	15	183	3	1.639344	0.938686	134|135|11255	ADORA1|ADORA2A|HRH3	-2.91294	0	114	0	0	-6.95615	41.31420765
1	-3.76876	GO:0006835	M1	1	0	GO Biological Processes	19	dicarboxylic acid transport	-3.76876	9.72099	6.282284	30242	85	183	5	2.73224	1.205087	134|135|4915|7442|11255	ADORA1|ADORA2A|NTRK2|TRPV1|HRH3	-2.68505	0	114	0	0	-6.95615	41.31420765
1	-3.58128	GO:0006865	M1	1	0	GO Biological Processes	19	amino acid transport	-3.58128	6.838214	5.49843	30242	145	183	6	3.278689	1.316393	134|135|3351|4915|7442|11255	ADORA1|ADORA2A|HTR1B|NTRK2|TRPV1|HRH3	-2.51788	0	114	0	0	-6.95615	41.31420765
1	-3.5109	GO:0032891	M1	1	0	GO Biological Processes	19	negative regulation of organic acid transport	-3.5109	22.53502	7.883985	30242	22	183	3	1.639344	0.938686	134|3351|11255	ADORA1|HTR1B|HRH3	-2.45542	0	114	0	0	-6.95615	41.31420765
1	-3.34249	GO:1903792	M1	1	0	GO Biological Processes	19	negative regulation of anion transport	-3.34249	19.83082	7.349367	30242	25	183	3	1.639344	0.938686	134|3351|11255	ADORA1|HTR1B|HRH3	-2.30396	0	114	0	0	-6.95615	41.31420765
1	-6.85261	GO:0002064	M1	1	0	GO Biological Processes	19	epithelial cell development	-6.85261	8.079223	8.316289	30242	225	183	11	6.010929	1.757049	367|2099|3383|3551|4914|5138|5241|7124|7132|7298|9475	AR|ESR1|ICAM1|IKBKB|NTRK1|PDE2A|PGR|TNF|TNFRSF1A|TYMS|ROCK2	-5.42748	0	115	1	1	-6.85261	99.15409836
1	-6.18734	GO:0061028	M1	1	0	GO Biological Processes	19	establishment of endothelial barrier	-6.18734	19.44198	10.28467	30242	51	183	6	3.278689	1.316393	3383|3551|5138|7124|7132|9475	ICAM1|IKBKB|PDE2A|TNF|TNFRSF1A|ROCK2	-4.82323	0	115	0	0	-6.85261	99.15409836
1	-5.92523	GO:0035303	M1	1	0	GO Biological Processes	19	regulation of dephosphorylation	-5.92523	10.32855	8.25223	30242	128	183	8	4.371585	1.511428	134|1133|1813|3551|5159|7040|7124|9475	ADORA1|CHRM5|DRD2|IKBKB|PDGFRB|TGFB1|TNF|ROCK2	-4.58295	0	115	0	0	-6.85261	99.15409836
1	-5.88751	GO:0035304	M1	1	0	GO Biological Processes	19	regulation of protein dephosphorylation	-5.88751	12.85331	8.78697	30242	90	183	7	3.825137	1.417845	134|1813|3551|5159|7040|7124|9475	ADORA1|DRD2|IKBKB|PDGFRB|TGFB1|TNF|ROCK2	-4.55306	0	115	0	0	-6.85261	99.15409836
1	-5.74639	GO:0016311	M1	1	0	GO Biological Processes	19	dephosphorylation	-5.74639	5.139567	6.649614	30242	418	183	13	7.103825	1.898976	52|134|249|596|761|1133|1813|3551|5159|5444|7040|7124|9475	ACP1|ADORA1|ALPL|BCL2|CA3|CHRM5|DRD2|IKBKB|PDGFRB|PON1|TGFB1|TNF|ROCK2	-4.4216	0	115	0	0	-6.85261	99.15409836
1	-5.66547	GO:0035509	M1	1	0	GO Biological Processes	19	negative regulation of myosin-light-chain-phosphatase activity	-5.66547	99.1541	17.12618	30242	5	183	3	1.639344	0.938686	3551|7124|9475	IKBKB|TNF|ROCK2	-4.35145	0	115	0	0	-6.85261	99.15409836
1	-5.42999	GO:1901550	M1	1	0	GO Biological Processes	19	regulation of endothelial cell	-5.42999	36.72374	11.82915	30242	18	183	4	2.185792	1.080886	3551|7124|7132|9475	IKBKB|TNF|TNFRSF1A|ROCK2	-4.14034	0	115	0	0	-6.85261	99.15409836

								development																		
1	-5.42999	GO:1903140	M1	1	0	GO Biological Processes	19	regulation of establishment of endothelial barrier	-5.42999	36.72374	11.82915	30242	18	183	4	2.185792	1.080886	3551|7124|7132|9475	IKBKB|TNF|TNFRSF1A|ROCK2	-4.14034	0	115	0	0	-6.85261	99.15409836
1	-5.40326	GO:0001885	M1	1	0	GO Biological Processes	19	endothelial cell development	-5.40326	14.37016	8.675375	30242	69	183	6	3.278689	1.316393	3383|3551|5138|7124|7132|9475	ICAM1|IKBKB|PDE2A|TNF|TNFRSF1A|ROCK2	-4.1163	0	115	0	0	-6.85261	99.15409836
1	-5.36639	GO:1903347	M1	1	0	GO Biological Processes	19	negative regulation of bicellular tight junction assembly	-5.36639	82.62842	15.60239	30242	6	183	3	1.639344	0.938686	3551|7124|9475	IKBKB|TNF|ROCK2	-4.08918	0	115	0	0	-6.85261	99.15409836
1	-5.12529	GO:0035507	M1	1	0	GO Biological Processes	19	regulation of myosin-light-chain-phosphatase activity	-5.12529	70.82436	14.41575	30242	7	183	3	1.639344	0.938686	3551|7124|9475	IKBKB|TNF|ROCK2	-3.87821	0	115	0	0	-6.85261	99.15409836
1	-4.56637	GO:0043666	M1	1	0	GO Biological Processes	19	regulation of phosphoprotein phosphatase activity	-4.56637	14.24628	7.878723	30242	58	183	5	2.73224	1.205087	1813|3551|5159|7124|9475	DRD2|IKBKB|PDGFRB|TNF|ROCK2	-3.3924	0	115	0	0	-6.85261	99.15409836
1	-4.46888	GO:0045216	M1	1	0	GO Biological Processes	19	cell-cell junction organization	-4.46888	6.544825	6.169499	30242	202	183	8	4.371585	1.511428	1636|2149|3551|7040|7046|7124|9475|59341	ACE|F2R|IKBKB|TGFB1|TGFBR1|TNF|ROCK2|TRPV4	-3.30913	0	115	0	0	-6.85261	99.15409836
1	-4.38865	GO:1901889	M1	1	0	GO Biological Processes	19	negative regulation of cell junction assembly	-4.38865	20.6571	8.680715	30242	32	183	4	2.185792	1.080886	1636|3551|7124|9475	ACE|IKBKB|TNF|ROCK2	-3.23843	0	115	0	0	-6.85261	99.15409836
1	-4.33391	GO:0030856	M1	1	0	GO Biological Processes	19	regulation of epithelial cell differentiation	-4.33391	7.463212	6.294499	30242	155	183	7	3.825137	1.417845	595|3551|4318|7124|7132|7421|9475	CCND1|IKBKB|MMP9|TNF|TNFRSF1A|VDR|ROCK2	-3.18907	0	115	0	0	-6.85261	99.15409836
1	-4.22668	GO:0006470	M1	1	0	GO Biological Processes	19	protein dephosphorylation	-4.22668	5.274154	5.626524	30242	282	183	9	4.918033	1.598524	52|134|596|1813|3551|5159|7040|7124|9475	ACP1|ADORA1|BCL2|DRD2|IKBKB|PDGFRB|TGFB1|TNF|ROCK2	-3.09139	0	115	0	0	-6.85261	99.15409836
1	-3.97078	GO:0045446	M1	1	0	GO Biological Processes	19	endothelial cell differentiation	-3.97078	8.061309	6.122853	30242	123	183	6	3.278689	1.316393	3383|3551|5138|7124|7132|9475	ICAM1|IKBKB|PDE2A|TNF|TNFRSF1A|ROCK2	-2.86187	0	115	0	0	-6.85261	99.15409836
1	-3.79297	GO:0010921	M1	1	0	GO Biological Processes	19	regulation of phosphatase activity	-3.79297	9.836716	6.327988	30242	84	183	5	2.73224	1.205087	1813|3551|5159|7124|9475	DRD2|IKBKB|PDGFRB|TNF|ROCK2	-2.70386	0	115	0	0	-6.85261	99.15409836
1	-3.68815	GO:0045601	M1	1	0	GO Biological Processes	19	regulation of endothelial cell differentiation	-3.68815	13.7714	6.909319	30242	48	183	4	2.185792	1.080886	3551|7124|7132|9475	IKBKB|TNF|TNFRSF1A|ROCK2	-2.6157	0	115	0	0	-6.85261	99.15409836
1	-3.64685	GO:0003158	M1	1	0	GO Biological Processes	19	endothelium development	-3.64685	7.032206	5.601851	30242	141	183	6	3.278689	1.316393	3383|3551|5138|7124|7132|9475	ICAM1|IKBKB|PDE2A|TNF|TNFRSF1A|ROCK2	-2.57732	0	115	0	0	-6.85261	99.15409836
1	-3.45213	GO:2000810	M1	1	0	GO Biological Processes	19	regulation of bicellular tight junction assembly	-3.45213	21.55524	7.694542	30242	23	183	3	1.639344	0.938686	3551|7124|9475	IKBKB|TNF|ROCK2	-2.40475	0	115	0	0	-6.85261	99.15409836
1	-3.29117	GO:0032515	M1	1	0	GO Biological Processes	19	negative regulation of phosphoprotein phosphatase activity	-3.29117	19.0681	7.191458	30242	26	183	3	1.639344	0.938686	3551|7124|9475	IKBKB|TNF|ROCK2	-2.25835	0	115	0	0	-6.85261	99.15409836
1	-3.28627	GO:0030865	M1	1	0	GO Biological Processes	19	cortical cytoskeleton organization	-3.28627	10.83651	6.000337	30242	61	183	4	2.185792	1.080886	3551|7124|9475|59341	IKBKB|TNF|ROCK2|TRPV4	-2.25445	0	115	0	0	-6.85261	99.15409836
1	-3.10109	GO:0072657	M1	1	0	GO Biological Processes	19	protein localization to membrane	-3.10109	3.166943	4.089739	30242	574	183	11	6.010929	1.757049	134|367|673|1956|2903|3551|3683|7040|7124|7132|9475	ADORA1|AR|BRAF|EGFR|GRIN2A|IKBKB|ITGAL|TGFB1|TNF|TNFRSF1A|ROCK2	-2.08753	0	115	0	0	-6.85261	99.15409836
1	-2.98149	GO:1990778	M1	1	0	GO Biological Processes	19	protein localization to cell periphery	-2.98149	3.946432	4.23125	30242	335	183	8	4.371585	1.511428	367|1956|2903|3551|7040|7124|7132|9475	AR|EGFR|GRIN2A|IKBKB|TGFB1|TNF|TNF	-1.98057	0	115	0	0	-6.85261	99.15409836

																			RSF1A|ROCK2							
1	-2.94459	GO:0035308	M1	1	0	GO Biological Processes	19	negative regulation of protein dephosphorylation	-2.94459	14.58149	6.182471	30242	34	183	3	1.639344	0.938686	3551|7124|9475	IKBKB|TNF|ROCK2	-1.94754	0	115	0	0	-6.85261	99.15409836
1	-2.87173	GO:0010923	M1	1	0	GO Biological Processes	19	negative regulation of phosphatase activity	-2.87173	13.7714	5.982457	30242	36	183	3	1.639344	0.938686	3551|7124|9475	IKBKB|TNF|ROCK2	-1.88231	0	115	0	0	-6.85261	99.15409836
1	-2.82347	GO:0120193	M1	1	0	GO Biological Processes	19	tight junction organization	-2.82347	8.160831	5.035231	30242	81	183	4	2.185792	1.080886	3551|7046|7124|9475	IKBKB|TGFBR1|TNF|ROCK2	-1.83834	0	115	0	0	-6.85261	99.15409836
1	-2.73837	GO:0030866	M1	1	0	GO Biological Processes	19	cortical actin cytoskeleton organization	-2.73837	12.39426	5.626453	30242	40	183	3	1.639344	0.938686	3551|7124|9475	IKBKB|TNF|ROCK2	-1.7638	0	115	0	0	-6.85261	99.15409836
1	-2.73081	GO:0072659	M1	1	0	GO Biological Processes	19	protein localization to plasma membrane	-2.73081	4.044748	4.036721	30242	286	183	7	3.825137	1.417845	367|1956|3551|7040|7124|7132|9475	AR|EGFR|IKBKB|TGFB1|TNF|TNFRSF1A|ROCK2	-1.75677	0	115	0	0	-6.85261	99.15409836
1	-2.68773	GO:0007043	M1	1	0	GO Biological Processes	19	cell-cell junction assembly	-2.68773	5.620981	4.382097	30242	147	183	5	2.73224	1.205087	1636|3551|7124|9475|59341	ACE|IKBKB|TNF|ROCK2|TRPV4	-1.7168	0	115	0	0	-6.85261	99.15409836
1	-2.66782	GO:0051668	M1	1	0	GO Biological Processes	19	localization within membrane	-2.66782	2.792358	3.607213	30242	651	183	11	6.010929	1.757049	134|367|673|1956|2903|3551|3683|7040|7124|7132|9475	ADORA1|AR|BRAF|EGFR|GRIN2A|IKBKB|ITGAL|TGFB1|TNF|TNFRSF1A|ROCK2	-1.69921	0	115	0	0	-6.85261	99.15409836
1	-2.59076	GO:0035305	M1	1	0	GO Biological Processes	19	negative regulation of dephosphorylation	-2.59076	11.01712	5.246911	30242	45	183	3	1.639344	0.938686	3551|7124|9475	IKBKB|TNF|ROCK2	-1.63232	0	115	0	0	-6.85261	99.15409836
1	-2.42864	GO:1903076	M1	1	0	GO Biological Processes	19	regulation of protein localization to plasma membrane	-2.42864	6.356032	4.269126	30242	104	183	4	2.185792	1.080886	367|1956|7040|7124	AR|EGFR|TGFB1|TNF	-1.48429	0	115	0	0	-6.85261	99.15409836
1	-2.16328	GO:1903829	M1	1	0	GO Biological Processes	19	positive regulation of cellular protein localization	-2.16328	3.59254	3.375996	30242	276	183	6	3.278689	1.316393	1956|2147|5743|7040|7124|9475	EGFR|F2|PTGS2|TGFB1|TNF|ROCK2	-1.2389	0	115	0	0	-6.85261	99.15409836
1	-2.14725	GO:1904375	M1	1	0	GO Biological Processes	19	regulation of protein localization to cell periphery	-2.14725	5.288219	3.748542	30242	125	183	4	2.185792	1.080886	367|1956|7040|7124	AR|EGFR|TGFB1|TNF	-1.22496	0	115	0	0	-6.85261	99.15409836
1	-2.05227	GO:0070830	M1	1	0	GO Biological Processes	19	bicellular tight junction assembly	-2.05227	7.082436	3.975217	30242	70	183	3	1.639344	0.938686	3551|7124|9475	IKBKB|TNF|ROCK2	-1.13619	0	115	0	0	-6.85261	99.15409836
1	-6.85138	GO:1901216	M1	1	0	GO Biological Processes	19	positive regulation of neuron death	-6.85138	13.62943	9.720717	30242	97	183	8	4.371585	1.511428	134|836|1728|2904|7099|7124|7157|23621	ADORA1|CASP3|NQO1|GRIN2B|TLR4|TNF|TP53|BACE1	-5.42698	0	116	1	1	-6.85138	29.16297011
1	-4.56637	GO:0043525	M1	1	0	GO Biological Processes	19	positive regulation of neuron apoptotic process	-4.56637	14.24628	7.878723	30242	58	183	5	2.73224	1.205087	836|1728|7124|7157|23621	CASP3|NQO1|TNF|TP53|BACE1	-3.3924	0	116	0	0	-6.85138	29.16297011
1	-3.85624	GO:0034349	M1	1	0	GO Biological Processes	19	glial cell apoptotic process	-3.85624	29.16297	9.062669	30242	17	183	3	1.639344	0.938686	836|6347|7157	CASP3|CCL2|TP53	-2.76097	0	116	0	0	-6.85138	29.16297011
1	-3.7239	GO:0046677	M1	1	0	GO Biological Processes	19	response to antibiotic	-3.7239	14.06441	6.99371	30242	47	183	4	2.185792	1.080886	249|836|1543|7157	ALPL|CASP3|CYP1A1|TP53	-2.64452	0	116	0	0	-6.85138	29.16297011
1	-2.76691	GO:0016241	M1	1	0	GO Biological Processes	19	regulation of macroautophagy	-2.76691	5.860171	4.513433	30242	141	183	5	2.73224	1.205087	154|836|5290|7157|29110	ADRB2|CASP3|PIK3CA|TP53|TBK1	-1.78815	0	116	0	0	-6.85138	29.16297011
1	-2.06939	GO:0061912	M1	1	0	GO Biological Processes	19	selective autophagy	-2.06939	7.18508	4.013257	30242	69	183	3	1.639344	0.938686	154|7157|29110	ADRB2|TP53|TBK1	-1.15307	0	116	0	0	-6.85138	29.16297011
1	-6.70343	GO:0071312	M1	1	0	GO Biological Processes	19	cellular response to alkaloid	-6.70343	23.60812	11.43989	30242	42	183	6	3.278689	1.316393	836|890|3351|3383|4988|7442	CASP3|CCNA2|HTR1B|ICAM1|OPRM1|TRPV1	-5.29491	0	117	1	1	-6.70343	27.5428051

1	-5.2251	GO:0002437	M1	1	0	GO Biological Processes	19	inflammatory response to antigenic stimulus	-5.2251	13.3992	8.33245	30242	74	183	6	3.278689	1.316393	1268|1991|3383|4988|7124|10800	CNR1|ELANE|ICAM1|OPRM1|TNF|CYSLTR1	-3.96486	0	117	0	0	-6.70343	27.5428051
1	-4.90168	GO:0002438	M1	1	0	GO Biological Processes	19	acute inflammatory response to antigenic stimulus	-4.90168	27.54281	10.14976	30242	24	183	4	2.185792	1.080886	1268|1991|3383|4988	CNR1|ELANE|ICAM1|OPRM1	-3.68342	0	117	0	0	-6.70343	27.5428051
1	-6.68144	GO:0010522	M1	1	0	GO Biological Processes	19	regulation of calcium ion transport into cytosol	-6.68144	12.96132	9.441564	30242	102	183	8	4.371585	1.511428	596|775|1812|2147|2149|2902|4842|5024	BCL2|CACNA1C|DRD1|F2|F2R|GRIN1|NOS1|P2RX3	-5.27433	0	118	1	1	-6.68144	21.55523877
1	-6.08352	GO:0051928	M1	1	0	GO Biological Processes	19	positive regulation of calcium ion transport	-6.08352	10.83651	8.494347	30242	122	183	8	4.371585	1.511428	1230|1812|2147|2149|2902|5024|5159|6347	CCR1|DRD1|F2|F2R|GRIN1|P2RX3|PDGFRB|CCL2	-4.7289	0	118	0	0	-6.68144	21.55523877
1	-4.7398	GO:0061041	M1	1	0	GO Biological Processes	19	regulation of wound healing	-4.7398	8.63282	6.90928	30242	134	183	7	3.825137	1.417845	150|2147|2149|2155|3156|4846|7124	ADRA2A|F2|F2R|F7|HMGCR|NOS3|TNF	-3.54033	0	118	0	0	-6.68144	21.55523877
1	-4.71891	GO:0010524	M1	1	0	GO Biological Processes	19	positive regulation of calcium ion transport into cytosol	-4.71891	15.30156	8.207283	30242	54	183	5	2.73224	1.205087	1812|2147|2149|2902|5024	DRD1|F2|F2R|GRIN1|P2RX3	-3.52206	0	118	0	0	-6.68144	21.55523877
1	-4.12924	GO:1903034	M1	1	0	GO Biological Processes	19	regulation of response to wounding	-4.12924	6.926933	5.99269	30242	167	183	7	3.825137	1.417845	150|2147|2149|2155|3156|4846|7124	ADRA2A|F2|F2R|F7|HMGCR|NOS3|TNF	-3.00403	0	118	0	0	-6.68144	21.55523877
1	-3.91905	GO:0051279	M1	1	0	GO Biological Processes	19	regulation of release of sequestered calcium ion into cytosol	-3.91905	10.45929	6.568577	30242	79	183	5	2.73224	1.205087	775|1812|2147|2149|4842	CACNA1C|DRD1|F2|F2R|NOS1	-2.81719	0	118	0	0	-6.68144	21.55523877
1	-3.45213	GO:0030194	M1	1	0	GO Biological Processes	19	positive regulation of blood coagulation	-3.45213	21.55524	7.694542	30242	23	183	3	1.639344	0.938686	2147|2149|2155	F2|F2R|F7	-2.40475	0	118	0	0	-6.68144	21.55523877
1	-3.45213	GO:1900048	M1	1	0	GO Biological Processes	19	positive regulation of hemostasis	-3.45213	21.55524	7.694542	30242	23	183	3	1.639344	0.938686	2147|2149|2155	F2|F2R|F7	-2.40475	0	118	0	0	-6.68144	21.55523877
1	-3.39607	GO:0050820	M1	1	0	GO Biological Processes	19	positive regulation of coagulation	-3.39607	20.6571	7.516725	30242	24	183	3	1.639344	0.938686	2147|2149|2155	F2|F2R|F7	-2.35299	0	118	0	0	-6.68144	21.55523877
1	-3.3416	GO:0090303	M1	1	0	GO Biological Processes	19	positive regulation of wound healing	-3.3416	11.20385	6.121324	30242	59	183	4	2.185792	1.080886	150|2147|2149|2155	ADRA2A|F2|F2R|F7	-2.30368	0	118	0	0	-6.68144	21.55523877
1	-3.15625	GO:0030193	M1	1	0	GO Biological Processes	19	regulation of blood coagulation	-3.15625	10.01557	5.72098	30242	66	183	4	2.185792	1.080886	2147|2149|2155|4846	F2|F2R|F7|NOS3	-2.13694	0	118	0	0	-6.68144	21.55523877
1	-3.10726	GO:1900046	M1	1	0	GO Biological Processes	19	regulation of hemostasis	-3.10726	9.72099	5.617462	30242	68	183	4	2.185792	1.080886	2147|2149|2155|4846	F2|F2R|F7|NOS3	-2.09198	0	118	0	0	-6.68144	21.55523877
1	-3.0367	GO:0050818	M1	1	0	GO Biological Processes	19	regulation of coagulation	-3.0367	9.310244	5.469964	30242	71	183	4	2.185792	1.080886	2147|2149|2155|4846	F2|F2R|F7|NOS3	-2.02937	0	118	0	0	-6.68144	21.55523877
1	-3.01392	GO:1903036	M1	1	0	GO Biological Processes	19	positive regulation of response to wounding	-3.01392	9.180935	5.422729	30242	72	183	4	2.185792	1.080886	150|2147|2149|2155	ADRA2A|F2|F2R|F7	-2.00966	0	118	0	0	-6.68144	21.55523877
1	-2.88421	GO:0061045	M1	1	0	GO Biological Processes	19	negative regulation of wound healing	-2.88421	8.474709	5.157432	30242	78	183	4	2.185792	1.080886	2147|3156|4846|7124	F2|HMGCR|NOS3|TNF	-1.89235	0	118	0	0	-6.68144	21.55523877
1	-2.73837	GO:0051281	M1	1	0	GO Biological Processes	19	positive regulation of release of sequestered calcium ion into cytosol	-2.73837	12.39426	5.626453	30242	40	183	3	1.639344	0.938686	1812|2147|2149	DRD1|F2|F2R	-1.7638	0	118	0	0	-6.68144	21.55523877
1	-2.58673	GO:1903035	M1	1	0	GO Biological Processes	19	negative regulation of response to wounding	-2.58673	7.032206	4.570326	30242	94	183	4	2.185792	1.080886	2147|3156|4846|7124	F2|HMGCR|NOS3|TNF	-1.62879	0	118	0	0	-6.68144	21.55523877
1	-2.00253	GO:1904427	M1	1	0	GO Biological Processes	19	positive regulation of calcium ion transmembrane transport	-2.00253	6.791377	3.865442	30242	73	183	3	1.639344	0.938686	1812|2147|2149	DRD1|F2|F2R	-1.09008	0	118	0	0	-6.68144	21.55523877
1	-6.66159	GO:0099552	M1	1	0	GO Biological	19	trans-synaptic signaling	-6.66159	165.2568	22.19917	30242	3	183	3	1.639344	0.938686	1268|2149|2915	CNR1|F2R|GRM5	-5.258	0	119	1	1	-6.66159	165.2568306

						Processes		by lipid, modulating synaptic transmission																		
1	-6.66159	GO:0099553	M1	1	0	GO Biological Processes	19	trans-synaptic signaling by endocannabinoid, modulating synaptic transmission	-6.66159	165.2568	22.19917	30242	3	183	3	1.639344	0.938686	1268|2149|2915	CNR1|F2R|GRM5	-5.258	0	119	0	0	-6.66159	165.2568306
1	-6.38279	GO:0099550	M1	1	0	GO Biological Processes	19	trans-synaptic signaling, modulating synaptic transmission	-6.38279	60.09339	15.29486	30242	11	183	4	2.185792	1.080886	1268|2149|2915|4915	CNR1|F2R|GRM5|NTRK2	-5.00371	0	119	0	0	-6.66159	165.2568306
1	-5.12529	GO:0099541	M1	1	0	GO Biological Processes	19	trans-synaptic signaling by lipid	-5.12529	70.82436	14.41575	30242	7	183	3	1.639344	0.938686	1268|2149|2915	CNR1|F2R|GRM5	-3.87821	0	119	0	0	-6.66159	165.2568306
1	-5.12529	GO:0099542	M1	1	0	GO Biological Processes	19	trans-synaptic signaling by endocannabinoid	-5.12529	70.82436	14.41575	30242	7	183	3	1.639344	0.938686	1268|2149|2915	CNR1|F2R|GRM5	-3.87821	0	119	0	0	-6.66159	165.2568306
1	-2.19759	GO:0043954	M1	1	0	GO Biological Processes	19	cellular component maintenance	-2.19759	7.996298	4.302698	30242	62	183	3	1.639344	0.938686	2149|2904|3643	F2R|GRIN2B|INSR	-1.27182	0	119	0	0	-6.66159	165.2568306
1	-6.6159	GO:0051129	M1	1	0	GO Biological Processes	19	negative regulation of cellular component organization	-6.6159	4.304809	6.857024	30242	691	183	18	9.836066	2.201413	351|367|472|1636|2904|3066|3551|4985|5290|5444|5468|5734|7040|7124|7132|7157|9475|59341	APP|AR|ATM|ACE|GRIN2B|HDAC2|IKBKB|OPRD1|PIK3CA|PON1|PPARG|PTGER4|TGFB1|TNF|TNFRSF1A|TP53|ROCK2|TRPV4	-5.21788	0	120	1	1	-6.6159	4.304808901
1	-6.34129	GO:0014829	M1	1	0	GO Biological Processes	19	vascular associated smooth muscle contraction	-6.34129	31.78016	12.25111	30242	26	183	5	2.73224	1.205087	151|1131|1909|1910|3356	ADRA2B|CHRM3|EDNRA|EDNRB|HTR2A	-4.96419	0	121	1	1	-6.34129	49.57704918
1	-6.23114	GO:0048863	M1	1	0	GO Biological Processes	19	stem cell differentiation	-6.23114	7.983422	7.866399	30242	207	183	10	5.464481	1.680144	1636|1909|1910|2099|2263|2335|3357|5591|5979|7157	ACE|EDNRA|EDNRB|ESR1|FGFR2|FN1|HTR2B|PRKDC|RET|TP53	-4.8612	0	121	0	0	-6.34129	49.57704918
1	-5.01826	GO:0048483	M1	1	0	GO Biological Processes	19	autonomic nervous system development	-5.01826	17.58051	8.875969	30242	47	183	5	2.73224	1.205087	1909|1910|2335|4914|5979	EDNRA|EDNRB|FN1|NTRK1|RET	-3.77989	0	121	0	0	-6.34129	49.57704918
1	-4.99	GO:0048469	M1	1	0	GO Biological Processes	19	cell maturation	-4.99	7.731314	6.887479	30242	171	183	8	4.371585	1.511428	351|596|1080|1910|5241|5468|5979|7298	APP|BCL2|CFTR|EDNRB|PGR|PPARG|RET|TYMS	-3.75496	0	121	0	0	-6.34129	49.57704918
1	-4.93484	GO:0071695	M1	1	0	GO Biological Processes	19	anatomical structure maturation	-4.93484	6.49481	6.512565	30242	229	183	9	4.918033	1.598524	351|596|1080|1910|4313|5241|5468|5979|7298	APP|BCL2|CFTR|EDNRB|MMP2|PGR|PPARG|RET|TYMS	-3.70734	0	121	0	0	-6.34129	49.57704918
1	-4.81782	GO:0014031	M1	1	0	GO Biological Processes	19	mesenchymal cell development	-4.81782	11.39702	7.577489	30242	87	183	6	3.278689	1.316393	596|1909|1910|2335|3357|5979	BCL2|EDNRA|EDNRB|FN1|HTR2B|RET	-3.60816	0	121	0	0	-6.34129	49.57704918
1	-4.596	GO:0014041	M1	1	0	GO Biological Processes	19	regulation of neuron maturation	-4.596	49.57705	11.98765	30242	10	183	3	1.639344	0.938686	596|1910|5979	BCL2|EDNRB|RET	-3.4183	0	121	0	0	-6.34129	49.57704918
1	-4.5622	GO:0045664	M1	1	0	GO Biological Processes	19	regulation of neuron differentiation	-4.5622	6.745177	6.296141	30242	196	183	8	4.371585	1.511428	151|152|351|596|1453|1910|5979|6532	ADRA2B|ADRA2C|APP|BCL2|CSNK1D|EDNRB|RET|SLC6A4	-3.38947	0	121	0	0	-6.34129	49.57704918
1	-4.25043	GO:0021700	M1	1	0	GO Biological Processes	19	developmental maturation	-4.25043	5.311827	5.655764	30242	280	183	9	4.918033	1.598524	351|596|1080|1910|4313|5241|5468|5979|7298	APP|BCL2|CFTR|EDNRB|MMP2|PGR|PPARG|RET|TYMS	-3.11173	0	121	0	0	-6.34129	49.57704918
1	-4.22463	GO:0007494	M1	1	0	GO Biological Processes	19	midgut development	-4.22463	38.13619	10.44945	30242	13	183	3	1.639344	0.938686	1910|1956|5979	EDNRB|EGFR|RET	-3.09123	0	121	0	0	-6.34129	49.57704918
1	-4.02686	GO:0048484	M1	1	0	GO Biological Processes	19	enteric nervous system development	-4.02686	33.05137	9.68793	30242	15	183	3	1.639344	0.938686	1909|1910|5979	EDNRA|EDNRB|RET	-2.91294	0	121	0	0	-6.34129	49.57704918
1	-3.84238	GO:0014032	M1	1	0	GO Biological	19	neural crest cell	-3.84238	10.07664	6.421737	30242	82	183	5	2.73224	1.205087	1909|1910|2335|3357|5	EDNRA|EDNRB|FN1|	-2.74951	0	121	0	0	-6.34129	49.57704918

						Processes		development										979	HTR2B|RET							
1	-3.79794	GO:0042551	M1	1	0	GO Biological Processes	19	neuron maturation	-3.79794	14.6895	7.170479	30242	45	183	4	2.185792	1.080886	351|596|1910|5979	APP|BCL2|EDNRB|RET	-2.70849	0	121	0	0	-6.34129	49.57704918
1	-3.72127	GO:0048864	M1	1	0	GO Biological Processes	19	stem cell development	-3.72127	9.497519	6.193105	30242	87	183	5	2.73224	1.205087	1909|1910|2335|3357|5979	EDNRA|EDNRB|FN1|HTR2B|RET	-2.64322	0	121	0	0	-6.34129	49.57704918
1	-3.60769	GO:0014033	M1	1	0	GO Biological Processes	19	neural crest cell differentiation	-3.60769	8.981349	5.982228	30242	92	183	5	2.73224	1.205087	1909|1910|2335|3357|5979	EDNRA|EDNRB|FN1|HTR2B|RET	-2.54172	0	121	0	0	-6.34129	49.57704918
1	-3.57263	GO:1903429	M1	1	0	GO Biological Processes	19	regulation of cell maturation	-3.57263	23.60812	8.086415	30242	21	183	3	1.639344	0.938686	596|1910|5979	BCL2|EDNRB|RET	-2.51178	0	121	0	0	-6.34129	49.57704918
1	-3.4286	GO:0001755	M1	1	0	GO Biological Processes	19	neural crest cell migration	-3.4286	11.80406	6.314145	30242	56	183	4	2.185792	1.080886	1910|2335|3357|5979	EDNRB|FN1|HTR2B|RET	-2.38276	0	121	0	0	-6.34129	49.57704918
1	-3.18136	GO:0038034	M1	1	0	GO Biological Processes	19	signal transduction in absence of ligand	-3.18136	10.16965	5.774413	30242	65	183	4	2.185792	1.080886	596|3558|5979|7124	BCL2|IL2|RET|TNF	-2.15944	0	121	0	0	-6.34129	49.57704918
1	-3.18136	GO:0097192	M1	1	0	GO Biological Processes	19	extrinsic apoptotic signaling pathway in absence of ligand	-3.18136	10.16965	5.774413	30242	65	183	4	2.185792	1.080886	596|3558|5979|7124	BCL2|IL2|RET|TNF	-2.15944	0	121	0	0	-6.34129	49.57704918
1	-2.64755	GO:2001239	M1	1	0	GO Biological Processes	19	regulation of extrinsic apoptotic signaling pathway in absence of ligand	-2.64755	11.52955	5.391182	30242	43	183	3	1.639344	0.938686	596|5979|7124	BCL2|RET|TNF	-1.68278	0	121	0	0	-6.34129	49.57704918
1	-2.17831	GO:0032835	M1	1	0	GO Biological Processes	19	glomerulus development	-2.17831	7.869373	4.258644	30242	63	183	3	1.639344	0.938686	596|5159|5979	BCL2|PDGFRB|RET	-1.25347	0	121	0	0	-6.34129	49.57704918
1	-6.28841	GO:1901361	M1	1	0	GO Biological Processes	19	organic cyclic compound catabolic process	-6.28841	4.898918	6.901038	30242	506	183	15	8.196721	2.027793	100|328|472|1543|1544|1588|3569|4128|4129|4363|5138|5142|6531|8654|9475	ADA|APEX1|ATM|CYP1A1|CYP1A2|CYP19A1|IL6|MAOA|MAOB|ABCC1|PDE2A|PDE4B|SLC6A3|PDE5A|ROCK2	-4.91394	0	122	1	1	-6.28841	35.41217799
1	-5.8952	GO:0019439	M1	1	0	GO Biological Processes	19	aromatic compound catabolic process	-5.8952	4.891323	6.655521	30242	473	183	14	7.650273	1.964856	100|328|472|1543|3569|4128|4129|4363|5138|5142|5444|6531|8654|9475	ADA|APEX1|ATM|CYP1A1|IL6|MAOA|MAOB|ABCC1|PDE2A|PDE4B|PON1|SLC6A3|PDE5A|ROCK2	-4.56016	0	122	0	0	-6.28841	35.41217799
1	-4.12183	GO:0009214	M1	1	0	GO Biological Processes	19	cyclic nucleotide catabolic process	-4.12183	35.41218	10.04865	30242	14	183	3	1.639344	0.938686	5138|5142|8654	PDE2A|PDE4B|PDE5A	-2.99883	0	122	0	0	-6.28841	35.41217799
1	-3.87567	GO:0009154	M1	1	0	GO Biological Processes	19	purine ribonucleotide catabolic process	-3.87567	15.37273	7.358911	30242	43	183	4	2.185792	1.080886	100|5138|5142|8654	ADA|PDE2A|PDE4B|PDE5A	-2.77725	0	122	0	0	-6.28841	35.41217799
1	-3.7239	GO:0006195	M1	1	0	GO Biological Processes	19	purine nucleotide catabolic process	-3.7239	14.06441	6.99371	30242	47	183	4	2.185792	1.080886	100|5138|5142|8654	ADA|PDE2A|PDE4B|PDE5A	-2.64452	0	122	0	0	-6.28841	35.41217799
1	-3.68815	GO:0009261	M1	1	0	GO Biological Processes	19	ribonucleotide catabolic process	-3.68815	13.7714	6.909319	30242	48	183	4	2.185792	1.080886	100|5138|5142|8654	ADA|PDE2A|PDE4B|PDE5A	-2.6157	0	122	0	0	-6.28841	35.41217799
1	-3.52087	GO:0072523	M1	1	0	GO Biological Processes	19	purine-containing compound catabolic process	-3.52087	12.47221	6.522246	30242	53	183	4	2.185792	1.080886	100|5138|5142|8654	ADA|PDE2A|PDE4B|PDE5A	-2.46287	0	122	0	0	-6.28841	35.41217799
1	-3.3088	GO:0046700	M1	1	0	GO Biological Processes	19	heterocycle catabolic process	-3.3088	3.624053	4.4054	30242	456	183	10	5.464481	1.680144	100|328|472|1543|3569|4363|5138|5142|8654|9475	ADA|APEX1|ATM|CYP1A1|IL6|ABCC1|PDE2A|PDE4B|PDE5A|ROCK2	-2.27299	0	122	0	0	-6.28841	35.41217799
1	-2.96938	GO:0009166	M1	1	0	GO Biological Processes	19	nucleotide catabolic process	-2.96938	8.932802	5.330962	30242	74	183	4	2.185792	1.080886	100|5138|5142|8654	ADA|PDE2A|PDE4B|PDE5A	-1.9693	0	122	0	0	-6.28841	35.41217799

1	-2.76521	GO:1901292	M1	1	0	GO Biological Processes	19	nucleoside phosphate catabolic process	-2.76521	7.869373	4.91917	30242	84	183	4	2.185792	1.080886	100|5138|5142|8654	ADA|PDE2A|PDE4B|PDE5A	-1.78724	0	122	0	0	-6.28841	35.41217799
1	-2.73837	GO:0009187	M1	1	0	GO Biological Processes	19	cyclic nucleotide metabolic process	-2.73837	12.39426	5.626453	30242	40	183	3	1.639344	0.938686	5138|5142|8654	PDE2A|PDE4B|PDE5A	-1.7638	0	122	0	0	-6.28841	35.41217799
1	-2.67635	GO:0044270	M1	1	0	GO Biological Processes	19	cellular nitrogen compound catabolic process	-2.67635	3.219289	3.75067	30242	462	183	9	4.918033	1.598524	100|328|472|3569|4363|5138|5142|8654|9475	ADA|APEX1|ATM|IL6|ABCC1|PDE2A|PDE4B|PDE5A|ROCK2	-1.70671	0	122	0	0	-6.28841	35.41217799
1	-2.57583	GO:0046434	M1	1	0	GO Biological Processes	19	organophosphate catabolic process	-2.57583	5.296693	4.198081	30242	156	183	5	2.73224	1.205087	100|5138|5142|5444|8654	ADA|PDE2A|PDE4B|PON1|PDE5A	-1.61814	0	122	0	0	-6.28841	35.41217799
1	-2.42527	GO:0034655	M1	1	0	GO Biological Processes	19	nucleobase-containing compound catabolic process	-2.42527	3.20887	3.522318	30242	412	183	8	4.371585	1.511428	100|328|472|3569|5138|5142|8654|9475	ADA|APEX1|ATM|IL6|PDE2A|PDE4B|PDE5A|ROCK2	-1.48117	0	122	0	0	-6.28841	35.41217799
1	-2.37344	GO:1901136	M1	1	0	GO Biological Processes	19	carbohydrate derivative catabolic process	-2.37344	4.74876	3.869424	30242	174	183	5	2.73224	1.205087	100|5138|5142|7040|8654	ADA|PDE2A|PDE4B|TGFB1|PDE5A	-1.43249	0	122	0	0	-6.28841	35.41217799
1	-6.19395	GO:0007613	M1	1	0	GO Biological Processes	19	memory	-6.19395	11.20385	8.665332	30242	118	183	8	4.371585	1.511428	1268|1813|2903|3356|3643|5743|6326|6532	CNR1|DRD2|GRIN2A|HTR2A|INSR|PTGS2|SCN2A|SLC6A4	-4.82919	0	123	1	1	-6.19395	11.20385292
1	-6.17118	GO:1990776	M1	1	0	GO Biological Processes	19	response to angiotensin	-6.17118	29.51015	11.77635	30242	28	183	5	2.73224	1.205087	185|760|5743|5970|9475	AGTR1|CA2|PTGS2|RELA|ROCK2	-4.80962	0	124	1	1	-6.17118	35.41217799
1	-4.82801	GO:1904385	M1	1	0	GO Biological Processes	19	cellular response to angiotensin	-4.82801	26.44109	9.92925	30242	25	183	4	2.185792	1.080886	185|760|5970|9475	AGTR1|CA2|RELA|ROCK2	-3.61611	0	124	0	0	-6.17118	35.41217799
1	-4.12183	GO:0038166	M1	1	0	GO Biological Processes	19	angiotensin-activated signaling pathway	-4.12183	35.41218	10.04865	30242	14	183	3	1.639344	0.938686	185|760|9475	AGTR1|CA2|ROCK2	-2.99883	0	124	0	0	-6.17118	35.41217799
1	-6.14002	GO:0044089	M1	1	0	GO Biological Processes	19	positive regulation of cellular component biogenesis	-6.14002	4.767024	6.760789	30242	520	183	15	8.196721	2.027793	472|2099|3383|4312|4314|4914|4915|5290|7040|7046|7099|7124|7157|9475|225689	ATM|ESR1|ICAM1|MMP1|MMP3|NTRK1|NTRK2|PIK3CA|TGFB1|TGFBR1|TLR4|TNF|TP53|ROCK2|MAPK15	-4.77973	0	125	1	1	-6.14002	6.24920788
1	-4.8725	GO:0043254	M1	1	0	GO Biological Processes	19	regulation of protein-containing complex assembly	-4.8725	4.611819	5.885738	30242	430	183	12	6.557377	1.829835	472|2099|3383|4312|4314|4985|5734|7040|7099|7124|7157|225689	ATM|ESR1|ICAM1|MMP1|MMP3|OPRD1|PTGER4|TGFB1|TLR4|TNF|TP53|MAPK15	-3.65698	0	125	0	0	-6.14002	6.24920788
1	-4.80169	GO:0031334	M1	1	0	GO Biological Processes	19	positive regulation of protein-containing complex assembly	-4.80169	6.249208	6.343501	30242	238	183	9	4.918033	1.598524	472|2099|3383|4312|4314|7040|7099|7124|7157	ATM|ESR1|ICAM1|MMP1|MMP3|TGFB1|TLR4|TNF|TP53	-3.59382	0	125	0	0	-6.14002	6.24920788
1	-5.93981	GO:0042133	M1	1	0	GO Biological Processes	19	neurotransmitter metabolic process	-5.93981	26.65433	11.15052	30242	31	183	5	2.73224	1.205087	4128|4129|4842|6531|6532	MAOA|MAOB|NOS1|SLC6A3|SLC6A4	-4.59631	0	126	1	1	-5.93981	55.0856102
1	-4.74896	GO:0042420	M1	1	0	GO Biological Processes	19	dopamine catabolic process	-4.74896	55.08561	12.6619	30242	9	183	3	1.639344	0.938686	4128|4129|6531	MAOA|MAOB|SLC6A3	-3.54861	0	126	0	0	-5.93981	55.0856102
1	-4.596	GO:0019614	M1	1	0	GO Biological Processes	19	catechol-containing compound catabolic process	-4.596	49.57705	11.98765	30242	10	183	3	1.639344	0.938686	4128|4129|6531	MAOA|MAOB|SLC6A3	-3.4183	0	126	0	0	-5.93981	55.0856102
1	-4.596	GO:0042424	M1	1	0	GO Biological Processes	19	catecholamine catabolic process	-4.596	49.57705	11.98765	30242	10	183	3	1.639344	0.938686	4128|4129|6531	MAOA|MAOB|SLC6A3	-3.4183	0	126	0	0	-5.93981	55.0856102
1	-4.22463	GO:0019336	M1	1	0	GO Biological Processes	19	phenol-containing compound catabolic process	-4.22463	38.13619	10.44945	30242	13	183	3	1.639344	0.938686	4128|4129|6531	MAOA|MAOB|SLC6A3	-3.09123	0	126	0	0	-5.93981	55.0856102
1	-3.77899	GO:0042136	M1	1	0	GO Biological Processes	19	neurotransmitter biosynthetic process	-3.77899	27.54281	8.789081	30242	18	183	3	1.639344	0.938686	4842|6531|6532	NOS1|SLC6A3|SLC6A4	-2.69427	0	126	0	0	-5.93981	55.0856102
1	-3.24195	GO:0042402	M1	1	0	GO Biological	19	cellular biogenic amine	-3.24195	18.36187	7.04212	30242	27	183	3	1.639344	0.938686	4128|4129|6531	MAOA|MAOB|SLC6A	-2.21358	0	126	0	0	-5.93981	55.0856102

						Processes		catabolic process											3							
1	-3.14917	GO:0009310	M1	1	0	GO Biological Processes	19	amine catabolic process	-3.14917	17.09553	6.766184	30242	29	183	3	1.639344	0.938686	4128|4129|6531	MAOA|MAOB|SLC6A3	-2.13101	0	126	0	0	-5.93981	55.0856102
1	-2.88421	GO:1901616	M1	1	0	GO Biological Processes	19	organic hydroxy compound catabolic process	-2.88421	8.474709	5.157432	30242	78	183	4	2.185792	1.080886	217|4128|4129|6531	ALDH2|MAOA|MAOB|SLC6A3	-1.89235	0	126	0	0	-5.93981	55.0856102
1	-5.8743	GO:0042476	M1	1	0	GO Biological Processes	19	odontogenesis	-5.8743	10.16965	8.175062	30242	130	183	8	4.371585	1.511428	249|760|1080|2263|3066|5465|6331|6336	ALPL|CA2|CFTR|FGFR2|HDAC2|PPARA|SCN5A|SCN10A	-4.54164	0	127	1	1	-5.8743	18.36187007
1	-4.73339	GO:0042475	M1	1	0	GO Biological Processes	19	odontogenesis of dentin-containing tooth	-4.73339	11.01712	7.425787	30242	90	183	6	3.278689	1.316393	760|1080|3066|5465|6331|6336	CA2|CFTR|HDAC2|PPARA|SCN5A|SCN10A	-3.53566	0	127	0	0	-5.8743	18.36187007
1	-3.24195	GO:0034505	M1	1	0	GO Biological Processes	19	tooth mineralization	-3.24195	18.36187	7.04212	30242	27	183	3	1.639344	0.938686	249|1080|5465	ALPL|CFTR|PPARA	-2.21358	0	127	0	0	-5.8743	18.36187007
1	-5.82531	GO:0035265	M1	1	0	GO Biological Processes	19	organ growth	-5.82531	8.355682	7.67973	30242	178	183	9	4.918033	1.598524	148|367|596|2099|2263|5159|5465|6532|7046	ADRA1A|AR|BCL2|ESR1|FGFR2|PDGFRB|PPARA|SLC6A4|TGFBR1	-4.49502	0	128	1	1	-5.82531	21.32346201
1	-4.44459	GO:0070723	M1	1	0	GO Biological Processes	19	response to cholesterol	-4.44459	21.32346	8.83349	30242	31	183	4	2.185792	1.080886	2155|3156|7040|7046	F7|HMGCR|TGFB1|TGFBR1	-3.29079	0	128	0	0	-5.82531	21.32346201
1	-4.18252	GO:0036314	M1	1	0	GO Biological Processes	19	response to sterol	-4.18252	18.36187	8.132751	30242	36	183	4	2.185792	1.080886	2155|3156|7040|7046	F7|HMGCR|TGFB1|TGFBR1	-3.05249	0	128	0	0	-5.82531	21.32346201
1	-3.83033	GO:0061061	M1	1	0	GO Biological Processes	19	muscle structure development	-3.83033	3.367302	4.715472	30242	638	183	13	7.103825	1.898976	148|596|836|1910|2263|3156|4842|5159|5465|5467|7040|7046|7124	ADRA1A|BCL2|CASP3|EDNRB|FGFR2|HMGCR|NOS1|PDGFRB|PPARA|PPARD|TGFB1|TGFBR1|TNF	-2.73849	0	128	0	0	-5.82531	21.32346201
1	-3.39607	GO:0048641	M1	1	0	GO Biological Processes	19	regulation of skeletal muscle tissue development	-3.39607	20.6571	7.516725	30242	24	183	3	1.639344	0.938686	596|3156|7040	BCL2|HMGCR|TGFB1	-2.35299	0	128	0	0	-5.82531	21.32346201
1	-3.22463	GO:0014706	M1	1	0	GO Biological Processes	19	striated muscle tissue development	-3.22463	3.863147	4.41151	30242	385	183	9	4.918033	1.598524	148|596|2263|3156|5159|5465|6331|7040|7046	ADRA1A|BCL2|FGFR2|HMGCR|PDGFRB|PPARA|SCN5A|TGFB1|TGFBR1	-2.19714	0	128	0	0	-5.82531	21.32346201
1	-3.21661	GO:0042692	M1	1	0	GO Biological Processes	19	muscle cell differentiation	-3.21661	3.853139	4.401868	30242	386	183	9	4.918033	1.598524	148|596|836|1910|2263|4842|5159|5465|7040	ADRA1A|BCL2|CASP3|EDNRB|FGFR2|NOS1|PDGFRB|PPARA|TGFB1	-2.18971	0	128	0	0	-5.82531	21.32346201
1	-3.20193	GO:0048738	M1	1	0	GO Biological Processes	19	cardiac muscle tissue development	-3.20193	4.881003	4.680128	30242	237	183	7	3.825137	1.417845	148|2263|5159|5465|6331|7040|7046	ADRA1A|FGFR2|PDGFRB|PPARA|SCN5A|TGFB1|TGFBR1	-2.17738	0	128	0	0	-5.82531	21.32346201
1	-3.07671	GO:0060537	M1	1	0	GO Biological Processes	19	muscle tissue development	-3.07671	3.681464	4.233642	30242	404	183	9	4.918033	1.598524	148|596|2263|3156|5159|5465|6331|7040|7046	ADRA1A|BCL2|FGFR2|HMGCR|PDGFRB|PPARA|SCN5A|TGFB1|TGFBR1	-2.0657	0	128	0	0	-5.82531	21.32346201
1	-2.83693	GO:0016202	M1	1	0	GO Biological Processes	19	regulation of striated muscle tissue development	-2.83693	13.3992	5.888322	30242	37	183	3	1.639344	0.938686	596|3156|7040	BCL2|HMGCR|TGFB1	-1.8502	0	128	0	0	-5.82531	21.32346201
1	-2.77031	GO:1901861	M1	1	0	GO Biological Processes	19	regulation of muscle tissue development	-2.77031	12.71206	5.710538	30242	39	183	3	1.639344	0.938686	596|3156|7040	BCL2|HMGCR|TGFB1	-1.79128	0	128	0	0	-5.82531	21.32346201
1	-2.7394	GO:0051146	M1	1	0	GO Biological Processes	19	striated muscle cell differentiation	-2.7394	4.05894	4.048373	30242	285	183	7	3.825137	1.417845	148|596|836|4842|5159|5465|7040	ADRA1A|BCL2|CASP3|NOS1|PDGFRB|PPA	-1.7638	0	128	0	0	-5.82531	21.32346201

																			RA|TGFB1							
1	-2.70729	GO:0048634	M1	1	0	GO Biological Processes	19	regulation of muscle organ development	-2.70729	12.09196	5.545313	30242	41	183	3	1.639344	0.938686	596|3156|7040	BCL2|HMGCR|TGFB1	-1.73455	0	128	0	0	-5.82531	21.32346201
1	-2.56386	GO:0035051	M1	1	0	GO Biological Processes	19	cardiocyte differentiation	-2.56386	5.262956	4.178516	30242	157	183	5	2.73224	1.205087	148|1956|5159|5465|7040	ADRA1A|EGFR|PDGFRB|PPARA|TGFB1	-1.6072	0	128	0	0	-5.82531	21.32346201
1	-2.14725	GO:0055007	M1	1	0	GO Biological Processes	19	cardiac muscle cell differentiation	-2.14725	5.288219	3.748542	30242	125	183	4	2.185792	1.080886	148|5159|5465|7040	ADRA1A|PDGFRB|PPARA|TGFB1	-1.22496	0	128	0	0	-5.82531	21.32346201
1	-5.80648	GO:0032768	M1	1	0	GO Biological Processes	19	regulation of monooxygenase activity	-5.80648	16.80578	9.481937	30242	59	183	6	3.278689	1.316393	1268|1956|3357|5291|7124|7421	CNR1|EGFR|HTR2B|PIK3CB|TNF|VDR	-4.47679	0	129	1	1	-5.80648	20.65710383
1	-5.38218	GO:0051341	M1	1	0	GO Biological Processes	19	regulation of oxidoreductase activity	-5.38218	10.81119	7.932581	30242	107	183	7	3.825137	1.417845	185|1268|1956|3357|5291|7124|7421	AGTR1|CNR1|EGFR|HTR2B|PIK3CB|TNF|VDR	-4.10001	0	129	0	0	-5.80648	20.65710383
1	-5.16186	GO:0050999	M1	1	0	GO Biological Processes	19	regulation of nitric-oxide synthase activity	-5.16186	18.77919	9.208427	30242	44	183	5	2.73224	1.205087	1268|1956|3357|5291|7124	CNR1|EGFR|HTR2B|PIK3CB|TNF	-3.90937	0	129	0	0	-5.80648	20.65710383
1	-4.53004	GO:0051353	M1	1	0	GO Biological Processes	19	positive regulation of oxidoreductase activity	-4.53004	14.00482	7.80163	30242	59	183	5	2.73224	1.205087	185|3357|5291|7124|7421	AGTR1|HTR2B|PIK3CB|TNF|VDR	-3.36222	0	129	0	0	-5.80648	20.65710383
1	-4.28228	GO:0032770	M1	1	0	GO Biological Processes	19	positive regulation of monooxygenase activity	-4.28228	19.44198	8.395032	30242	34	183	4	2.185792	1.080886	3357|5291|7124|7421	HTR2B|PIK3CB|TNF|VDR	-3.14168	0	129	0	0	-5.80648	20.65710383
1	-3.39607	GO:0051000	M1	1	0	GO Biological Processes	19	positive regulation of nitric-oxide synthase activity	-3.39607	20.6571	7.516725	30242	24	183	3	1.639344	0.938686	3357|5291|7124	HTR2B|PIK3CB|TNF	-2.35299	0	129	0	0	-5.80648	20.65710383
1	-5.79935	GO:0007423	M1	1	0	GO Biological Processes	19	sensory organ development	-5.79935	4.474463	6.440038	30242	554	183	15	8.196721	2.027793	596|775|890|1543|1545|1813|1956|2263|2558|3066|4915|5159|5979|6531|7046	BCL2|CACNA1C|CCNA2|CYP1A1|CYP1B1|DRD2|EGFR|FGFR2|GABRA5|HDAC2|NTRK2|PDGFRB|RET|SLC6A3|TGFBR1	-4.47024	0	130	1	1	-5.79935	22.53502235
1	-4.75117	GO:0001654	M1	1	0	GO Biological Processes	19	eye development	-4.75117	4.899798	5.897128	30242	371	183	11	6.010929	1.757049	596|775|1543|1545|1813|3066|4915|5159|5979|6531|7046	BCL2|CACNA1C|CYP1A1|CYP1B1|DRD2|HDAC2|NTRK2|PDGFRB|RET|SLC6A3|TGFBR1	-3.54861	0	130	0	0	-5.79935	22.53502235
1	-4.70829	GO:0150063	M1	1	0	GO Biological Processes	19	visual system development	-4.70829	4.847534	5.849768	30242	375	183	11	6.010929	1.757049	596|775|1543|1545|1813|3066|4915|5159|5979|6531|7046	BCL2|CACNA1C|CYP1A1|CYP1B1|DRD2|HDAC2|NTRK2|PDGFRB|RET|SLC6A3|TGFBR1	-3.5123	0	130	0	0	-5.79935	22.53502235
1	-4.645	GO:0048880	M1	1	0	GO Biological Processes	19	sensory system development	-4.645	4.771195	5.77997	30242	381	183	11	6.010929	1.757049	596|775|1543|1545|1813|3066|4915|5159|5979|6531|7046	BCL2|CACNA1C|CYP1A1|CYP1B1|DRD2|HDAC2|NTRK2|PDGFRB|RET|SLC6A3|TGFBR1	-3.45548	0	130	0	0	-5.79935	22.53502235
1	-4.53696	GO:0043010	M1	1	0	GO Biological Processes	19	camera-type eye development	-4.53696	5.1322	5.816541	30242	322	183	10	5.464481	1.680144	775|1543|1545|1813|3066|4915|5159|5979|6531|7046	CACNA1C|CYP1A1|CYP1B1|DRD2|HDAC2|NTRK2|PDGFRB|RET|SLC6A3|TGFBR1	-3.3671	0	130	0	0	-5.79935	22.53502235
1	-3.5109	GO:0055093	M1	1	0	GO Biological Processes	19	response to hyperoxia	-3.5109	22.53502	7.883985	30242	22	183	3	1.639344	0.938686	1543|3066|5159	CYP1A1|HDAC2|PDGFRB	-2.45542	0	130	0	0	-5.79935	22.53502235
1	-3.10534	GO:0036296	M1	1	0	GO Biological Processes	19	response to increased	-3.10534	16.52568	6.638317	30242	30	183	3	1.639344	0.938686	1543|3066|5159	CYP1A1|HDAC2|PDG	-2.09149	0	130	0	0	-5.79935	22.53502235

						Processes		oxygen levels											FRB							
1	-5.60351	GO:0051098	M1	1	0	GO Biological Processes	19	regulation of binding	-5.60351	5.448027	6.661603	30242	364	183	12	6.557377	1.829835	154|351|1636|3066|4221|4318|4792|5444|5465|5468|7040|7046	ADRB2|APP|ACE|HDAC2|MEN1|MMP9|NFKBIA|PON1|PPARA|PPARG|TGFB1|TGFBR1	-4.2957	0	131	1	1	-5.60351	10.11776514
1	-4.03317	GO:0051099	M1	1	0	GO Biological Processes	19	positive regulation of binding	-4.03317	6.686693	5.852746	30242	173	183	7	3.825137	1.417845	351|1636|4221|4318|5444|5468|7040	APP|ACE|MEN1|MMP9|PON1|PPARG|TGFB1	-2.91745	0	131	0	0	-5.60351	10.11776514
1	-3.68431	GO:0043393	M1	1	0	GO Biological Processes	19	regulation of protein binding	-3.68431	5.87207	5.352996	30242	197	183	7	3.825137	1.417845	154|351|1636|4221|4318|5465|7046	ADRB2|APP|ACE|MEN1|MMP9|PPARA|TGFBR1	-2.61218	0	131	0	0	-5.60351	10.11776514
1	-3.11137	GO:0051101	M1	1	0	GO Biological Processes	19	regulation of DNA binding	-3.11137	7.002408	5.097375	30242	118	183	5	2.73224	1.205087	3066|4318|4792|5468|7040	HDAC2|MMP9|NFKBIA|PPARG|TGFB1	-2.09465	0	131	0	0	-5.60351	10.11776514
1	-2.74631	GO:0032092	M1	1	0	GO Biological Processes	19	positive regulation of protein binding	-2.74631	7.776792	4.881754	30242	85	183	4	2.185792	1.080886	351|1636|4221|4318	APP|ACE|MEN1|MMP9	-1.76965	0	131	0	0	-5.60351	10.11776514
1	-2.48503	GO:0030225	M1	1	0	GO Biological Processes	19	macrophage differentiation	-2.48503	10.11777	4.983904	30242	49	183	3	1.639344	0.938686	351|4318|7040	APP|MMP9|TGFB1	-1.53506	0	131	0	0	-5.60351	10.11776514
1	-2.32104	GO:0043388	M1	1	0	GO Biological Processes	19	positive regulation of DNA binding	-2.32104	8.853044	4.589503	30242	56	183	3	1.639344	0.938686	4318|5468|7040	MMP9|PPARG|TGFB1	-1.38595	0	131	0	0	-5.60351	10.11776514
1	-5.37746	GO:0030099	M1	1	0	GO Biological Processes	19	myeloid cell differentiation	-5.37746	5.17776	6.420097	30242	383	183	12	6.557377	1.829835	351|760|836|1230|4318|4792|5293|5468|5591|7040|7099|7124	APP|CA2|CASP3|CCR1|MMP9|NFKBIA|PIK3CD|PPARG|PRKDC|TGFB1|TLR4|TNF	-4.09582	0	132	1	1	-5.37746	21.55523877
1	-5.25391	GO:0002573	M1	1	0	GO Biological Processes	19	myeloid leukocyte differentiation	-5.25391	7.116323	6.923099	30242	209	183	9	4.918033	1.598524	351|760|1230|4318|5293|5468|7040|7099|7124	APP|CA2|CCR1|MMP9|PIK3CD|PPARG|TGFB1|TLR4|TNF	-3.99091	0	132	0	0	-5.37746	21.55523877
1	-4.0543	GO:0002637	M1	1	0	GO Biological Processes	19	regulation of immunoglobulin production	-4.0543	11.166	6.831706	30242	74	183	5	2.73224	1.205087	3558|3569|5591|7040|7124	IL2|IL6|PRKDC|TGFB1|TNF	-2.93678	0	132	0	0	-5.37746	21.55523877
1	-3.50313	GO:0045637	M1	1	0	GO Biological Processes	19	regulation of myeloid cell differentiation	-3.50313	5.482454	5.098102	30242	211	183	7	3.825137	1.417845	760|1230|4792|5591|7040|7099|7124	CA2|CCR1|NFKBIA|PRKDC|TGFB1|TLR4|TNF	-2.4486	0	132	0	0	-5.37746	21.55523877
1	-3.45213	GO:0045662	M1	1	0	GO Biological Processes	19	negative regulation of myoblast differentiation	-3.45213	21.55524	7.694542	30242	23	183	3	1.639344	0.938686	5467|7040|7124	PPARD|TGFB1|TNF	-2.40475	0	132	0	0	-5.37746	21.55523877
1	-3.37005	GO:0002763	M1	1	0	GO Biological Processes	19	positive regulation of myeloid leukocyte differentiation	-3.37005	11.39702	6.184021	30242	58	183	4	2.185792	1.080886	760|1230|7040|7124	CA2|CCR1|TGFB1|TNF	-2.32819	0	132	0	0	-5.37746	21.55523877
1	-3.36119	GO:0045639	M1	1	0	GO Biological Processes	19	positive regulation of myeloid cell differentiation	-3.36119	7.94504	5.535675	30242	104	183	5	2.73224	1.205087	760|1230|5591|7040|7124	CA2|CCR1|PRKDC|TGFB1|TNF	-2.32024	0	132	0	0	-5.37746	21.55523877
1	-3.29117	GO:0045672	M1	1	0	GO Biological Processes	19	positive regulation of osteoclast differentiation	-3.29117	19.0681	7.191458	30242	26	183	3	1.639344	0.938686	760|1230|7124	CA2|CCR1|TNF	-2.25835	0	132	0	0	-5.37746	21.55523877
1	-3.2497	GO:0002698	M1	1	0	GO Biological Processes	19	negative regulation of immune effector process	-3.2497	7.511674	5.338403	30242	110	183	5	2.73224	1.205087	3558|5591|7040|7124|729230	IL2|PRKDC|TGFB1|TNF|CCR2	-2.21986	0	132	0	0	-5.37746	21.55523877
1	-3.20691	GO:0045670	M1	1	0	GO Biological Processes	19	regulation of osteoclast differentiation	-3.20691	10.32855	5.829017	30242	64	183	4	2.185792	1.080886	760|1230|7099|7124	CA2|CCR1|TLR4|TNF	-2.18207	0	132	0	0	-5.37746	21.55523877
1	-3.07846	GO:0002761	M1	1	0	GO Biological Processes	19	regulation of myeloid leukocyte	-3.07846	6.885701	5.040612	30242	120	183	5	2.73224	1.205087	760|1230|7040|7099|7124	CA2|CCR1|TGFB1|TLR4|TNF	-2.06717	0	132	0	0	-5.37746	21.55523877

								differentiation																		
1	-3.02224	GO:0007435	M1	1	0	GO Biological Processes	19	salivary gland morphogenesis	-3.02224	15.49283	6.400135	30242	32	183	3	1.639344	0.938686	2263|7040|7124	FGFR2|TGFB1|TNF	-2.01659	0	132	0	0	-5.37746	21.55523877
1	-2.94459	GO:0007431	M1	1	0	GO Biological Processes	19	salivary gland development	-2.94459	14.58149	6.182471	30242	34	183	3	1.639344	0.938686	2263|7040|7124	FGFR2|TGFB1|TNF	-1.94754	0	132	0	0	-5.37746	21.55523877
1	-2.77031	GO:0002701	M1	1	0	GO Biological Processes	19	negative regulation of production of molecular mediator of immune response	-2.77031	12.71206	5.710538	30242	39	183	3	1.639344	0.938686	5591|7040|7124	PRKDC|TGFB1|TNF	-1.79128	0	132	0	0	-5.37746	21.55523877
1	-2.76521	GO:0045445	M1	1	0	GO Biological Processes	19	myoblast differentiation	-2.76521	7.869373	4.91917	30242	84	183	4	2.185792	1.080886	3156|5467|7040|7124	HMGCR|PPARD|TGFB1|TNF	-1.78724	0	132	0	0	-5.37746	21.55523877
1	-2.73837	GO:0002714	M1	1	0	GO Biological Processes	19	positive regulation of B cell mediated immunity	-2.73837	12.39426	5.626453	30242	40	183	3	1.639344	0.938686	3558|7040|7124	IL2|TGFB1|TNF	-1.7638	0	132	0	0	-5.37746	21.55523877
1	-2.73837	GO:0002891	M1	1	0	GO Biological Processes	19	positive regulation of immunoglobulin mediated immune response	-2.73837	12.39426	5.626453	30242	40	183	3	1.639344	0.938686	3558|7040|7124	IL2|TGFB1|TNF	-1.7638	0	132	0	0	-5.37746	21.55523877
1	-2.59076	GO:0035272	M1	1	0	GO Biological Processes	19	exocrine system development	-2.59076	11.01712	5.246911	30242	45	183	3	1.639344	0.938686	2263|7040|7124	FGFR2|TGFB1|TNF	-1.63232	0	132	0	0	-5.37746	21.55523877
1	-2.58673	GO:0030316	M1	1	0	GO Biological Processes	19	osteoclast differentiation	-2.58673	7.032206	4.570326	30242	94	183	4	2.185792	1.080886	760|1230|7099|7124	CA2|CCR1|TLR4|TNF	-1.62879	0	132	0	0	-5.37746	21.55523877
1	-2.43567	GO:0045661	M1	1	0	GO Biological Processes	19	regulation of myoblast differentiation	-2.43567	9.72099	4.863495	30242	51	183	3	1.639344	0.938686	5467|7040|7124	PPARD|TGFB1|TNF	-1.49059	0	132	0	0	-5.37746	21.55523877
1	-2.29947	GO:0016447	M1	1	0	GO Biological Processes	19	somatic recombination of immunoglobulin gene segments	-2.29947	8.697728	4.538797	30242	57	183	3	1.639344	0.938686	3558|5591|7040	IL2|PRKDC|TGFB1	-1.36665	0	132	0	0	-5.37746	21.55523877
1	-2.2372	GO:0002712	M1	1	0	GO Biological Processes	19	regulation of B cell mediated immunity	-2.2372	8.262842	4.393843	30242	60	183	3	1.639344	0.938686	3558|7040|7124	IL2|TGFB1|TNF	-1.30863	0	132	0	0	-5.37746	21.55523877
1	-2.2372	GO:0002889	M1	1	0	GO Biological Processes	19	regulation of immunoglobulin mediated immune response	-2.2372	8.262842	4.393843	30242	60	183	3	1.639344	0.938686	3558|7040|7124	IL2|TGFB1|TNF	-1.30863	0	132	0	0	-5.37746	21.55523877
1	-2.10448	GO:0016445	M1	1	0	GO Biological Processes	19	somatic diversification of immunoglobulins	-2.10448	7.39956	4.091666	30242	67	183	3	1.639344	0.938686	3558|5591|7040	IL2|PRKDC|TGFB1	-1.18457	0	132	0	0	-5.37746	21.55523877
1	-2.08679	GO:0002562	M1	1	0	GO Biological Processes	19	somatic diversification of immune receptors via germline recombination within a single locus	-2.08679	7.290743	4.052064	30242	68	183	3	1.639344	0.938686	3558|5591|7040	IL2|PRKDC|TGFB1	-1.16956	0	132	0	0	-5.37746	21.55523877
1	-2.08679	GO:0016444	M1	1	0	GO Biological Processes	19	somatic cell DNA recombination	-2.08679	7.290743	4.052064	30242	68	183	3	1.639344	0.938686	3558|5591|7040	IL2|PRKDC|TGFB1	-1.16956	0	132	0	0	-5.37746	21.55523877
1	-5.37096	GO:0006730	M1	1	0	GO Biological Processes	19	one-carbon metabolic process	-5.37096	20.6571	9.70662	30242	40	183	5	2.73224	1.205087	759|760|761|762|7298	CA1|CA2|CA3|CA4|TYMS	-4.09037	0	133	1	1	-5.37096	20.65710383
1	-5.36639	GO:1990962	M1	1	0	GO Biological Processes	19	xenobiotic transport across blood-brain barrier	-5.36639	82.62842	15.60239	30242	6	183	3	1.639344	0.938686	4363|5243|9429	ABCC1|ABCB1|ABCG2	-4.08918	0	134	1	1	-5.36639	82.6284153
1	-4.81782	GO:0150104	M1	1	0	GO Biological Processes	19	transport across blood-brain barrier	-4.81782	11.39702	7.577489	30242	87	183	6	3.278689	1.316393	3643|4363|5243|6513|6523|9429	INSR|ABCC1|ABCB1|SLC2A1|SLC5A1|ABCG2	-3.60816	0	134	0	0	-5.36639	82.6284153
1	-4.78932	GO:0010232	M1	1	0	GO Biological Processes	19	vascular transport	-4.78932	11.26751	7.526107	30242	88	183	6	3.278689	1.316393	3643|4363|5243|6513|6523|9429	INSR|ABCC1|ABCB1|SLC2A1|SLC5A1|ABC	-3.58234	0	134	0	0	-5.36639	82.6284153

																			G2							
1	-3.68815	GO:0042908	M1	1	0	GO Biological Processes	19	xenobiotic transport	-3.68815	13.7714	6.909319	30242	48	183	4	2.185792	1.080886	4363|5243|6513|9429	ABCC1|ABCB1|SLC2A1|ABCG2	-2.6157	0	134	0	0	-5.36639	82.6284153
1	-2.92617	GO:0140115	M1	1	0	GO Biological Processes	19	export across plasma membrane	-2.92617	8.697728	5.242601	30242	76	183	4	2.185792	1.080886	3757|4363|5243|9429	KCNH2|ABCC1|ABCB1|ABCG2	-1.92994	0	134	0	0	-5.36639	82.6284153
1	-2.56339	GO:0051180	M1	1	0	GO Biological Processes	19	vitamin transport	-2.56339	10.77762	5.178139	30242	46	183	3	1.639344	0.938686	4363|6513|9429	ABCC1|SLC2A1|ABCG2	-1.6072	0	134	0	0	-5.36639	82.6284153
1	-5.32866	GO:0007173	M1	1	0	GO Biological Processes	19	epidermal growth factor receptor signaling pathway	-5.32866	10.61282	7.844755	30242	109	183	7	3.825137	1.417845	134|150|673|1956|4318|5290|7040	ADORA1|ADRA2A|BRAF|EGFR|MMP9|PIK3CA|TGFB1	-4.05644	0	135	1	1	-5.32866	26.4410929
1	-5.00552	GO:0038127	M1	1	0	GO Biological Processes	19	ERBB signaling pathway	-5.00552	9.481949	7.324609	30242	122	183	7	3.825137	1.417845	134|150|673|1956|4318|5290|7040	ADORA1|ADRA2A|BRAF|EGFR|MMP9|PIK3CA|TGFB1	-3.76905	0	135	0	0	-5.32866	26.4410929
1	-4.82801	GO:0050995	M1	1	0	GO Biological Processes	19	negative regulation of lipid catabolic process	-4.82801	26.44109	9.92925	30242	25	183	4	2.185792	1.080886	134|150|1268|7124	ADORA1|ADRA2A|CNR1|TNF	-3.61611	0	135	0	0	-5.32866	26.4410929
1	-4.6034	GO:0033619	M1	1	0	GO Biological Processes	19	membrane protein proteolysis	-4.6034	14.49621	7.957747	30242	57	183	5	2.73224	1.205087	150|5979|7040|7124|23621	ADRA2A|RET|TGFB1|TNF|BACE1	-3.41983	0	135	0	0	-5.32866	26.4410929
1	-4.45935	GO:0050994	M1	1	0	GO Biological Processes	19	regulation of lipid catabolic process	-4.45935	13.54564	7.652923	30242	61	183	5	2.73224	1.205087	134|150|1268|5465|7124	ADORA1|ADRA2A|CNR1|PPARA|TNF	-3.30278	0	135	0	0	-5.32866	26.4410929
1	-3.10534	GO:0007176	M1	1	0	GO Biological Processes	19	regulation of epidermal growth factor-activated receptor activity	-3.10534	16.52568	6.638317	30242	30	183	3	1.639344	0.938686	134|150|351	ADORA1|ADRA2A|APP	-2.09149	0	135	0	0	-5.32866	26.4410929
1	-2.99148	GO:0042058	M1	1	0	GO Biological Processes	19	regulation of epidermal growth factor receptor signaling pathway	-2.99148	9.055169	5.376405	30242	73	183	4	2.185792	1.080886	134|150|1956|4318	ADORA1|ADRA2A|EGFR|MMP9	-1.98834	0	135	0	0	-5.32866	26.4410929
1	-2.87173	GO:0045742	M1	1	0	GO Biological Processes	19	positive regulation of epidermal growth factor receptor signaling pathway	-2.87173	13.7714	5.982457	30242	36	183	3	1.639344	0.938686	134|150|4318	ADORA1|ADRA2A|MMP9	-1.88231	0	135	0	0	-5.32866	26.4410929
1	-2.86368	GO:1901184	M1	1	0	GO Biological Processes	19	regulation of ERBB signaling pathway	-2.86368	8.367434	5.115981	30242	79	183	4	2.185792	1.080886	134|150|1956|4318	ADORA1|ADRA2A|EGFR|MMP9	-1.87452	0	135	0	0	-5.32866	26.4410929
1	-2.80314	GO:1901186	M1	1	0	GO Biological Processes	19	positive regulation of ERBB signaling pathway	-2.80314	13.04659	5.797758	30242	38	183	3	1.639344	0.938686	134|150|4318	ADORA1|ADRA2A|MMP9	-1.82068	0	135	0	0	-5.32866	26.4410929
1	-2.56339	GO:0006509	M1	1	0	GO Biological Processes	19	membrane protein ectodomain proteolysis	-2.56339	10.77762	5.178139	30242	46	183	3	1.639344	0.938686	150|7124|23621	ADRA2A|TNF|BACE1	-1.6072	0	135	0	0	-5.32866	26.4410929
1	-2.45882	GO:0045833	M1	1	0	GO Biological Processes	19	negative regulation of lipid metabolic process	-2.45882	6.48066	4.326111	30242	102	183	4	2.185792	1.080886	134|150|1268|7124	ADORA1|ADRA2A|CNR1|TNF	-1.51081	0	135	0	0	-5.32866	26.4410929
1	-5.27623	GO:1904659	M1	1	0	GO Biological Processes	19	glucose transmembrane transport	-5.27623	10.4216	7.75918	30242	111	183	7	3.825137	1.417845	673|1909|3643|5467|6513|6523|7124	BRAF|EDNRA|INSR|PPARD|SLC2A1|SLC5A1|TNF	-4.01119	0	136	1	1	-5.27623	18.88649493
1	-5.19952	GO:0008645	M1	1	0	GO Biological Processes	19	hexose transmembrane transport	-5.19952	10.14735	7.634822	30242	114	183	7	3.825137	1.417845	673|1909|3643|5467|6513|6523|7124	BRAF|EDNRA|INSR|PPARD|SLC2A1|SLC5A1|TNF	-3.94355	0	136	0	0	-5.27623	18.88649493
1	-5.14962	GO:0015749	M1	1	0	GO Biological Processes	19	monosaccharide transmembrane transport	-5.14962	9.972395	7.554453	30242	116	183	7	3.825137	1.417845	673|1909|3643|5467|6513|6523|7124	BRAF|EDNRA|INSR|PPARD|SLC2A1|SLC5A1|TNF	-3.8996	0	136	0	0	-5.27623	18.88649493
1	-5.10068	GO:0034219	M1	1	0	GO Biological Processes	19	carbohydrate transmembrane transport	-5.10068	9.803371	7.476013	30242	118	183	7	3.825137	1.417845	673|1909|3643|5467|6513|6523|7124	BRAF|EDNRA|INSR|PPARD|SLC2A1|SLC5A1|TNF	-3.85555	0	136	0	0	-5.27623	18.88649493

1	-4.38792	GO:0008643	M1	1	0	GO Biological Processes	19	carbohydrate transport	-4.38792	7.610512	6.375029	30242	152	183	7	3.825137	1.417845	673|1909|3643|5467|6513|6523|7124	BRAF|EDNRA|INSR|PPARD|SLC2A1|SLC5A1|TNF	-3.23843	0	136	0	0	-5.27623	18.88649493
1	-4.23163	GO:0043243	M1	1	0	GO Biological Processes	19	positive regulation of protein-containing complex disassembly	-4.23163	18.88649	8.261175	30242	35	183	4	2.185792	1.080886	154|3643|7124|59341	ADRB2|INSR|TNF|TRPV4	-3.09596	0	136	0	0	-5.27623	18.88649493
1	-3.54805	GO:0005975	M1	1	0	GO Biological Processes	19	carbohydrate metabolic process	-3.54805	3.344152	4.498258	30242	593	183	12	6.557377	1.829835	217|351|673|3356|3643|5290|5465|5467|6513|7040|7124|7157	ALDH2|APP|BRAF|HTR2A|INSR|PIK3CA|PPARA|PPARD|SLC2A1|TGFB1|TNF|TP53	-2.48879	0	136	0	0	-5.27623	18.88649493
1	-3.06223	GO:0043244	M1	1	0	GO Biological Processes	19	regulation of protein-containing complex disassembly	-3.06223	6.828795	5.012716	30242	121	183	5	2.73224	1.205087	154|3643|5290|7124|59341	ADRB2|INSR|PIK3CA|TNF|TRPV4	-2.05405	0	136	0	0	-5.27623	18.88649493
1	-2.99627	GO:0005996	M1	1	0	GO Biological Processes	19	monosaccharide metabolic process	-2.99627	4.501159	4.398076	30242	257	183	7	3.825137	1.417845	3643|5290|5465|5467|6513|7124|7157	INSR|PIK3CA|PPARA|PPARD|SLC2A1|TNF|TP53	-1.99258	0	136	0	0	-5.27623	18.88649493
1	-2.96938	GO:0046323	M1	1	0	GO Biological Processes	19	glucose import	-2.96938	8.932802	5.330962	30242	74	183	4	2.185792	1.080886	3643|6513|6523|7124	INSR|SLC2A1|SLC5A1|TNF	-1.9693	0	136	0	0	-5.27623	18.88649493
1	-2.90504	GO:0010827	M1	1	0	GO Biological Processes	19	regulation of glucose transmembrane transport	-2.90504	8.58477	5.199631	30242	77	183	4	2.185792	1.080886	673|1909|3643|7124	BRAF|EDNRA|INSR|TNF	-1.911	0	136	0	0	-5.27623	18.88649493
1	-2.89384	GO:0006006	M1	1	0	GO Biological Processes	19	glucose metabolic process	-2.89384	5.058883	4.448131	30242	196	183	6	3.278689	1.316393	3643|5290|5465|5467|7124|7157	INSR|PIK3CA|PPARA|PPARD|TNF|TP53	-1.90097	0	136	0	0	-5.27623	18.88649493
1	-2.80314	GO:0097242	M1	1	0	GO Biological Processes	19	amyloid-beta clearance	-2.80314	13.04659	5.797758	30242	38	183	3	1.639344	0.938686	3156|3643|7124	HMGCR|INSR|TNF	-1.82068	0	136	0	0	-5.27623	18.88649493
1	-2.48093	GO:0019318	M1	1	0	GO Biological Processes	19	hexose metabolic process	-2.48093	4.183717	3.839266	30242	237	183	6	3.278689	1.316393	3643|5290|5465|5467|7124|7157	INSR|PIK3CA|PPARA|PPARD|TNF|TP53	-1.53145	0	136	0	0	-5.27623	18.88649493
1	-5.11592	GO:0001655	M1	1	0	GO Biological Processes	19	urogenital system development	-5.11592	5.362316	6.302255	30242	339	183	11	6.010929	1.757049	185|367|596|760|1636|2099|2263|4318|5159|5979|7046	AGTR1|AR|BCL2|CA2|ACE|ESR1|FGFR2|MMP9|PDGFRB|RET|TGFBR1	-3.86932	0	137	1	1	-5.11592	12.3942623
1	-5.02805	GO:0009791	M1	1	0	GO Biological Processes	19	post-embryonic development	-5.02805	12.39426	7.962281	30242	80	183	6	3.278689	1.316393	596|1544|2263|2566|6335|7046	BCL2|CYP1A2|FGFR2|GABRG2|SCN9A|TGFBR1	-3.78824	0	137	0	0	-5.11592	12.3942623
1	-4.08833	GO:0001822	M1	1	0	GO Biological Processes	19	kidney development	-4.08833	5.058883	5.456732	30242	294	183	9	4.918033	1.598524	185|596|760|1636|2263|4318|5159|5979|7046	AGTR1|BCL2|CA2|ACE|FGFR2|MMP9|PDGFRB|RET|TGFBR1	-2.96862	0	137	0	0	-5.11592	12.3942623
1	-3.98898	GO:0072001	M1	1	0	GO Biological Processes	19	renal system development	-3.98898	4.908619	5.335343	30242	303	183	9	4.918033	1.598524	185|596|760|1636|2263|4318|5159|5979|7046	AGTR1|BCL2|CA2|ACE|FGFR2|MMP9|PDGFRB|RET|TGFBR1	-2.87721	0	137	0	0	-5.11592	12.3942623
1	-5.10068	GO:0071346	M1	1	0	GO Biological Processes	19	cellular response to interferon-gamma	-5.10068	9.803371	7.476013	30242	118	183	7	3.825137	1.417845	834|3383|4843|5468|6347|7099|7157	CASP1|ICAM1|NOS2|PPARG|CCL2|TLR4|TP53	-3.85555	0	138	1	1	-5.10068	30.98565574
1	-4.59692	GO:0034341	M1	1	0	GO Biological Processes	19	response to interferon-gamma	-4.59692	8.20424	6.690269	30242	141	183	7	3.825137	1.417845	834|3383|4843|5468|6347|7099|7157	CASP1|ICAM1|NOS2|PPARG|CCL2|TLR4|TP53	-3.4183	0	138	0	0	-5.10068	30.98565574
1	-3.93862	GO:0051770	M1	1	0	GO Biological Processes	19	positive regulation of nitric-oxide synthase biosynthetic process	-3.93862	30.98566	9.360942	30242	16	183	3	1.639344	0.938686	6347|7099|10135	CCL2|TLR4|NAMPT	-2.83536	0	138	0	0	-5.10068	30.98565574
1	-3.57263	GO:0051767	M1	1	0	GO Biological Processes	19	nitric-oxide synthase biosynthetic process	-3.57263	23.60812	8.086415	30242	21	183	3	1.639344	0.938686	6347|7099|10135	CCL2|TLR4|NAMPT	-2.51178	0	138	0	0	-5.10068	30.98565574

1	-3.57263	GO:0051769	M1	1	0	GO Biological Processes	19	regulation of nitric-oxide synthase biosynthetic process	-3.57263	23.60812	8.086415	30242	21	183	3	1.639344	0.938686	6347|7099|10135	CCL2|TLR4|NAMPT	-2.51178	0	138	0	0	-5.10068	30.98565574
1	-5.09666	GO:0030029	M1	1	0	GO Biological Processes	19	actin filament-based process	-5.09666	3.516103	5.624023	30242	799	183	17	9.289617	2.145862	134|150|596|775|3383|3551|3757|5142|5159|5290|5734|6331|6401|7046|7124|9475|59341	ADORA1|ADRA2A|BCL2|CACNA1C|ICAM1|IKBKB|KCNH2|PDE4B|PDGFRB|PIK3CA|PTGER4|SCN5A|SELE|TGFBR1|TNF|ROCK2|TRPV4	-3.85201	0	139	1	1	-5.09666	4.152181673
1	-3.7758	GO:0032970	M1	1	0	GO Biological Processes	19	regulation of actin filament-based process	-3.7758	4.152182	4.939246	30242	398	183	10	5.464481	1.680144	134|775|3383|5142|5159|5290|5734|6331|7046|9475	ADORA1|CACNA1C|ICAM1|PDE4B|PDGFRB|PIK3CA|PTGER4|SCN5A|TGFBR1|ROCK2	-2.69175	0	139	0	0	-5.09666	4.152181673
1	-2.70171	GO:0097435	M1	1	0	GO Biological Processes	19	supramolecular fiber organization	-2.70171	2.66902	3.594088	30242	743	183	12	6.557377	1.829835	351|596|1453|1545|3383|5159|5290|5734|7046|9475|23621|59341	APP|BCL2|CSNK1D|CYP1B1|ICAM1|PDGFRB|PIK3CA|PTGER4|TGFBR1|ROCK2|BACE1|TRPV4	-1.72922	0	139	0	0	-5.09666	4.152181673
1	-2.36904	GO:0030036	M1	1	0	GO Biological Processes	19	actin cytoskeleton organization	-2.36904	2.549544	3.267088	30242	713	183	11	6.010929	1.757049	150|596|3383|3551|5159|5290|5734|7046|7124|9475|59341	ADRA2A|BCL2|ICAM1|IKBKB|PDGFRB|PIK3CA|PTGER4|TGFBR1|TNF|ROCK2|TRPV4	-1.42857	0	139	0	0	-5.09666	4.152181673
1	-2.04518	GO:1902903	M1	1	0	GO Biological Processes	19	regulation of supramolecular fiber organization	-2.04518	3.02036	3.104751	30242	383	183	7	3.825137	1.417845	351|3383|5290|5734|7046|9475|59341	APP|ICAM1|PIK3CA|PTGER4|TGFBR1|ROCK2|TRPV4	-1.12932	0	139	0	0	-5.09666	4.152181673
1	-2.04417	GO:1902905	M1	1	0	GO Biological Processes	19	positive regulation of supramolecular fiber organization	-2.04417	3.953513	3.343097	30242	209	183	5	2.73224	1.205087	351|3383|7046|9475|59341	APP|ICAM1|TGFBR1|ROCK2|TRPV4	-1.12854	0	139	0	0	-5.09666	4.152181673
1	-5.02805	GO:0048145	M1	1	0	GO Biological Processes	19	regulation of fibroblast proliferation	-5.02805	12.39426	7.962281	30242	80	183	6	3.278689	1.316393	890|2099|2335|5159|5591|7157	CCNA2|ESR1|FN1|PDGFRB|PRKDC|TP53	-3.78824	0	140	1	1	-5.02805	17.21425319
1	-4.9968	GO:0048144	M1	1	0	GO Biological Processes	19	fibroblast proliferation	-4.9968	12.24125	7.904428	30242	81	183	6	3.278689	1.316393	890|2099|2335|5159|5591|7157	CCNA2|ESR1|FN1|PDGFRB|PRKDC|TP53	-3.76129	0	140	0	0	-5.02805	17.21425319
1	-4.97262	GO:0048146	M1	1	0	GO Biological Processes	19	positive regulation of fibroblast proliferation	-4.97262	17.21425	8.771899	30242	48	183	5	2.73224	1.205087	890|2099|2335|5159|5591	CCNA2|ESR1|FN1|PDGFRB|PRKDC	-3.74133	0	140	0	0	-5.02805	17.21425319
1	-5.01826	GO:0043114	M1	1	0	GO Biological Processes	19	regulation of vascular permeability	-5.01826	17.58051	8.875969	30242	47	183	5	2.73224	1.205087	135|624|5138|7040|59341	ADORA2A|BDKRB2|PDE2A|TGFB1|TRPV4	-3.77989	0	141	1	1	-5.01826	29.16297011
1	-3.85624	GO:0043117	M1	1	0	GO Biological Processes	19	positive regulation of vascular permeability	-3.85624	29.16297	9.062669	30242	17	183	3	1.639344	0.938686	5138|7040|59341	PDE2A|TGFB1|TRPV4	-2.76097	0	141	0	0	-5.01826	29.16297011
1	-3.26353	GO:0007162	M1	1	0	GO Biological Processes	19	negative regulation of cell adhesion	-3.26353	4.363217	4.590858	30242	303	183	8	4.371585	1.511428	135|836|1545|3558|4221|5465|7040|59341	ADORA2A|CASP3|CYP1B1|IL2|MEN1|PPARA|TGFB1|TRPV4	-2.2325	0	141	0	0	-5.01826	29.16297011
1	-2.89384	GO:0022408	M1	1	0	GO Biological Processes	19	negative regulation of cell-cell adhesion	-2.89384	5.058883	4.448131	30242	196	183	6	3.278689	1.316393	135|836|3558|5465|7040|59341	ADORA2A|CASP3|IL2|PPARA|TGFB1|TRPV4	-1.90097	0	141	0	0	-5.01826	29.16297011
1	-5.01554	GO:0016032	M1	1	0	GO Biological Processes	19	viral process	-5.01554	4.767024	6.036482	30242	416	183	12	6.557377	1.829835	185|596|1956|3356|3383|3576|3643|5465|6347|7124|7157|9475	AGTR1|BCL2|EGFR|HTR2A|ICAM1|CXCL8|INSR|PPARA|CCL2|T	-3.77764	0	142	1	1	-5.01554	8.26284153

																			NF|TP53|ROCK2							
1	-4.58302	GO:0019058	M1	1	0	GO Biological Processes	19	viral life cycle	-4.58302	5.196756	5.870212	30242	318	183	10	5.464481	1.680144	185|596|1956|3356|3383|3576|5465|6347|7124|9475	AGTR1|BCL2|EGFR|HTR2A|ICAM1|CXCL8|PPARA|CCL2|TNF|ROCK2	-3.40615	0	142	0	0	-5.01554	8.26284153
1	-3.82055	GO:0019079	M1	1	0	GO Biological Processes	19	viral genome replication	-3.82055	7.569015	5.879084	30242	131	183	6	3.278689	1.316393	596|3576|5465|6347|7124|9475	BCL2|CXCL8|PPARA|CCL2|TNF|ROCK2	-2.7294	0	142	0	0	-5.01554	8.26284153
1	-3.43963	GO:1904035	M1	1	0	GO Biological Processes	19	regulation of epithelial cell apoptotic process	-3.43963	8.262842	5.67619	30242	100	183	5	2.73224	1.205087	3383|3569|5465|6347|7124	ICAM1|IL6|PPARA|CCL2|TNF	-2.39318	0	142	0	0	-5.01554	8.26284153
1	-3.01448	GO:1904019	M1	1	0	GO Biological Processes	19	epithelial cell apoptotic process	-3.01448	6.663582	4.93089	30242	124	183	5	2.73224	1.205087	3383|3569|5465|6347|7124	ICAM1|IL6|PPARA|CCL2|TNF	-2.00966	0	142	0	0	-5.01554	8.26284153
1	-2.74631	GO:0045069	M1	1	0	GO Biological Processes	19	regulation of viral genome replication	-2.74631	7.776792	4.881754	30242	85	183	4	2.185792	1.080886	596|3576|5465|7124	BCL2|CXCL8|PPARA|TNF	-1.76965	0	142	0	0	-5.01554	8.26284153
1	-2.19759	GO:2000351	M1	1	0	GO Biological Processes	19	regulation of endothelial cell apoptotic process	-2.19759	7.996298	4.302698	30242	62	183	3	1.639344	0.938686	3383|6347|7124	ICAM1|CCL2|TNF	-1.27182	0	142	0	0	-5.01554	8.26284153
1	-2.10448	GO:0072577	M1	1	0	GO Biological Processes	19	endothelial cell apoptotic process	-2.10448	7.39956	4.091666	30242	67	183	3	1.639344	0.938686	3383|6347|7124	ICAM1|CCL2|TNF	-1.18457	0	142	0	0	-5.01554	8.26284153
1	-4.86241	GO:0010638	M1	1	0	GO Biological Processes	19	positive regulation of organelle organization	-4.86241	4.254136	5.753991	30242	505	183	13	7.103825	1.898976	154|472|1814|3383|3643|4318|5159|7046|7124|7157|9475|59341|225689	ADRB2|ATM|DRD3|ICAM1|INSR|MMP9|PDGFRB|TGFBR1|TNF|TP53|ROCK2|TRPV4|MAPK15	-3.64734	0	143	1	1	-4.86241	11.52954632
1	-2.64755	GO:0045840	M1	1	0	GO Biological Processes	19	positive regulation of mitotic nuclear division	-2.64755	11.52955	5.391182	30242	43	183	3	1.639344	0.938686	1814|3643|5159	DRD3|INSR|PDGFRB	-1.68278	0	143	0	0	-4.86241	11.52954632
1	-2.34194	GO:0007088	M1	1	0	GO Biological Processes	19	regulation of mitotic nuclear division	-2.34194	6.009339	4.106759	30242	110	183	4	2.185792	1.080886	472|1814|3643|5159	ATM|DRD3|INSR|PDGFRB	-1.40555	0	143	0	0	-4.86241	11.52954632
1	-2.29947	GO:0051785	M1	1	0	GO Biological Processes	19	positive regulation of nuclear division	-2.29947	8.697728	4.538797	30242	57	183	3	1.639344	0.938686	1814|3643|5159	DRD3|INSR|PDGFRB	-1.36665	0	143	0	0	-4.86241	11.52954632
1	-4.84437	GO:0001501	M1	1	0	GO Biological Processes	19	skeletal system development	-4.84437	4.237355	5.735835	30242	507	183	13	7.103825	1.898976	249|2263|4313|4318|4325|5734|6197|7040|7046|7157|7298|7421|59341	ALPL|FGFR2|MMP2|MMP9|MMP16|PTGER4|RPS6KA3|TGFB1|TGFBR1|TP53|TYMS|VDR|TRPV4	-3.63065	0	144	1	1	-4.84437	9.180935033
1	-2.7947	GO:0060348	M1	1	0	GO Biological Processes	19	bone development	-2.7947	4.836785	4.300832	30242	205	183	6	3.278689	1.316393	249|2263|4325|5734|7157|59341	ALPL|FGFR2|MMP16|PTGER4|TP53|TRPV4	-1.81276	0	144	0	0	-4.84437	9.180935033
1	-2.64065	GO:0048705	M1	1	0	GO Biological Processes	19	skeletal system morphogenesis	-2.64065	4.507004	4.073467	30242	220	183	6	3.278689	1.316393	249|2263|4313|4325|7046|59341	ALPL|FGFR2|MMP2|MMP16|TGFBR1|TRPV4	-1.67639	0	144	0	0	-4.84437	9.180935033
1	-2.60359	GO:0060349	M1	1	0	GO Biological Processes	19	bone morphogenesis	-2.60359	7.107821	4.602859	30242	93	183	4	2.185792	1.080886	249|2263|4325|59341	ALPL|FGFR2|MMP16|TRPV4	-1.64263	0	144	0	0	-4.84437	9.180935033
1	-2.36548	GO:0060350	M1	1	0	GO Biological Processes	19	endochondral bone morphogenesis	-2.36548	9.180935	4.694821	30242	54	183	3	1.639344	0.938686	249|4325|59341	ALPL|MMP16|TRPV4	-1.4267	0	144	0	0	-4.84437	9.180935033
1	-4.76118	GO:0014910	M1	1	0	GO Biological Processes	19	regulation of smooth muscle cell migration	-4.76118	11.14091	7.475548	30242	89	183	6	3.278689	1.316393	328|596|1636|5159|5467|7099	APEX1|BCL2|ACE|PDGFRB|PPARD|TLR4	-3.55553	0	145	1	1	-4.76118	22.53502235
1	-4.54796	GO:0014909	M1	1	0	GO Biological Processes	19	smooth muscle cell migration	-4.54796	10.22207	7.098116	30242	97	183	6	3.278689	1.316393	328|596|1636|5159|5467|7099	APEX1|BCL2|ACE|PDGFRB|PPARD|TLR4	-3.37728	0	145	0	0	-4.76118	22.53502235
1	-4.24017	GO:0014812	M1	1	0	GO Biological Processes	19	muscle cell migration	-4.24017	9.014009	6.570048	30242	110	183	6	3.278689	1.316393	328|596|1636|5159|5467|7099	APEX1|BCL2|ACE|PDGFRB|PPARD|TLR4	-3.10224	0	145	0	0	-4.76118	22.53502235
1	-3.5217	GO:0036473	M1	1	0	GO Biological	19	cell death in response to	-3.5217	8.607127	5.824756	30242	96	183	5	2.73224	1.205087	596|1545|4314|7099|10	BCL2|CYP1B1|MMP3|	-2.46307	0	145	0	0	-4.76118	22.53502235

						Processes		oxidative stress											280	TLR4|SIGMAR1							
1	-3.5109	GO:0036120	M1	1	0	GO Biological Processes	19	cellular response to platelet-derived growth factor stimulus	-3.5109	22.53502	7.883985	30242	22	183	3	1.639344	0.938686	890|5159|7099	CCNA2|PDGFRB|TLR4	-2.45542	0	145	0	0	-4.76118	22.53502235
1	-3.45213	GO:0036119	M1	1	0	GO Biological Processes	19	response to platelet-derived growth factor	-3.45213	21.55524	7.694542	30242	23	183	3	1.639344	0.938686	890|5159|7099	CCNA2|PDGFRB|TLR4	-2.40475	0	145	0	0	-4.76118	22.53502235
1	-2.53666	GO:0014911	M1	1	0	GO Biological Processes	19	positive regulation of smooth muscle cell migration	-2.53666	10.54831	5.111451	30242	47	183	3	1.639344	0.938686	596|5159|7099	BCL2|PDGFRB|TLR4	-1.58173	0	145	0	0	-4.76118	22.53502235
1	-2.01093	GO:0043583	M1	1	0	GO Biological Processes	19	ear development	-2.01093	3.879268	3.290318	30242	213	183	5	2.73224	1.205087	596|890|2263|2558|5159	BCL2|CCNA2|FGFR2|GABRA5|PDGFRB	-1.09734	0	145	0	0	-4.76118	22.53502235
1	-4.68993	GO:0009651	M1	1	0	GO Biological Processes	19	response to salt stress	-4.68993	24.48249	9.524697	30242	27	183	4	2.185792	1.080886	624|7124|7157|59341	BDKRB2|TNF|TP53|TRPV4	-3.49655	0	146	1	1	-4.68993	24.48249342
1	-3.7239	GO:0097300	M1	1	0	GO Biological Processes	19	programmed necrotic cell death	-3.7239	14.06441	6.99371	30242	47	183	4	2.185792	1.080886	427|834|7124|7157	ASAH1|CASP1|TNF|TP53	-2.64452	0	146	0	0	-4.68993	24.48249342
1	-3.2329	GO:0070265	M1	1	0	GO Biological Processes	19	necrotic cell death	-3.2329	10.4925	5.88484	30242	63	183	4	2.185792	1.080886	427|834|7124|7157	ASAH1|CASP1|TNF|TP53	-2.20512	0	146	0	0	-4.68993	24.48249342
1	-2.98337	GO:2001235	M1	1	0	GO Biological Processes	19	positive regulation of apoptotic signaling pathway	-2.98337	6.557811	4.877831	30242	126	183	5	2.73224	1.205087	135|5979|7046|7124|7157	ADORA2A|RET|TGFBR1|TNF|TP53	-1.98159	0	146	0	0	-4.68993	24.48249342
1	-4.64676	GO:0045926	M1	1	0	GO Biological Processes	19	negative regulation of growth	-4.64676	5.973138	6.148322	30242	249	183	9	4.918033	1.598524	153|154|596|2100|5465|5467|6532|7040|7157	ADRB1|ADRB2|BCL2|ESR2|PPARA|PPARD|SLC6A4|TGFB1|TP53	-3.45682	0	147	1	1	-4.64676	17.0955342
1	-3.5529	GO:2000378	M1	1	0	GO Biological Processes	19	negative regulation of reactive oxygen species metabolic process	-3.5529	12.71206	6.595381	30242	52	183	4	2.185792	1.080886	596|4314|5465|7157	BCL2|MMP3|PPARA|TP53	-2.49333	0	147	0	0	-4.64676	17.0955342
1	-3.45398	GO:0031669	M1	1	0	GO Biological Processes	19	cellular response to nutrient levels	-3.45398	5.380455	5.029431	30242	215	183	7	3.825137	1.417845	596|3383|5465|6513|7157|7421|10135	BCL2|ICAM1|PPARA|SLC2A1|TP53|VDR|NAMPT	-2.40475	0	147	0	0	-4.64676	17.0955342
1	-3.14917	GO:1902253	M1	1	0	GO Biological Processes	19	regulation of intrinsic apoptotic signaling pathway by p53 class mediator	-3.14917	17.09553	6.766184	30242	29	183	3	1.639344	0.938686	596|624|7157	BCL2|BDKRB2|TP53	-2.13101	0	147	0	0	-4.64676	17.0955342
1	-3.10685	GO:0031668	M1	1	0	GO Biological Processes	19	cellular response to extracellular stimulus	-3.10685	4.70243	4.549452	30242	246	183	7	3.825137	1.417845	596|3383|5465|6513|7157|7421|10135	BCL2|ICAM1|PPARA|SLC2A1|TP53|VDR|NAMPT	-2.09186	0	147	0	0	-4.64676	17.0955342
1	-2.98668	GO:0030308	M1	1	0	GO Biological Processes	19	negative regulation of cell growth	-2.98668	5.274154	4.586848	30242	188	183	6	3.278689	1.316393	596|2100|5465|5467|7040|7157	BCL2|ESR2|PPARA|PPARD|TGFB1|TP53	-1.98382	0	147	0	0	-4.64676	17.0955342
1	-2.60359	GO:1901796	M1	1	0	GO Biological Processes	19	regulation of signal transduction by p53 class mediator	-2.60359	7.107821	4.602859	30242	93	183	4	2.185792	1.080886	472|596|624|7157	ATM|BCL2|BDKRB2|TP53	-1.64263	0	147	0	0	-4.64676	17.0955342
1	-2.52128	GO:2001243	M1	1	0	GO Biological Processes	19	negative regulation of intrinsic apoptotic signaling pathway	-2.52128	6.745177	4.444801	30242	98	183	4	2.185792	1.080886	596|624|4318|5743	BCL2|BDKRB2|MMP9|PTGS2	-1.5666	0	147	0	0	-4.64676	17.0955342
1	-2.51055	GO:0042149	M1	1	0	GO Biological Processes	19	cellular response to glucose starvation	-2.51055	10.32855	5.046739	30242	48	183	3	1.639344	0.938686	596|6513|7157	BCL2|SLC2A1|TP53	-1.55686	0	147	0	0	-4.64676	17.0955342
1	-2.4826	GO:2001242	M1	1	0	GO Biological Processes	19	regulation of intrinsic apoptotic signaling pathway	-2.4826	5.038318	4.046079	30242	164	183	5	2.73224	1.205087	596|624|4318|5743|7157	BCL2|BDKRB2|MMP9|PTGS2|TP53	-1.53287	0	147	0	0	-4.64676	17.0955342
1	-2.25757	GO:0001836	M1	1	0	GO Biological	19	release of cytochrome c	-2.25757	8.40289	4.441017	30242	59	183	3	1.639344	0.938686	596|4318|7157	BCL2|MMP9|TP53	-1.32711	0	147	0	0	-4.64676	17.0955342

						Processes		from mitochondria																		
1	-2.14888	GO:0042594	M1	1	0	GO Biological Processes	19	response to starvation	-2.14888	4.194336	3.509651	30242	197	183	5	2.73224	1.205087	596|1723|5465|6513|7157	BCL2|DHODH|PPARA|SLC2A1|TP53	-1.2259	0	147	0	0	-4.64676	17.0955342
1	-4.56637	GO:0002931	M1	1	0	GO Biological Processes	19	response to ischemia	-4.56637	14.24628	7.878723	30242	58	183	5	2.73224	1.205087	596|1728|5291|7157|9475	BCL2|NQO1|PIK3CB|TP53|ROCK2	-3.3924	0	148	1	1	-4.56637	14.2462785
1	-2.67703	GO:0033574	M1	1	0	GO Biological Processes	19	response to testosterone	-2.67703	11.80406	5.466942	30242	42	183	3	1.639344	0.938686	367|1728|9475	AR|NQO1|ROCK2	-1.70713	0	148	0	0	-4.56637	14.2462785
1	-2.16005	GO:0031589	M1	1	0	GO Biological Processes	19	cell-substrate adhesion	-2.16005	3.178016	3.261913	30242	364	183	7	3.825137	1.417845	596|2335|3683|4221|5291|5467|9475	BCL2|FN1|ITGAL|MEN1|PIK3CB|PPARD|ROCK2	-1.23591	0	148	0	0	-4.56637	14.2462785
1	-4.52269	GO:0043502	M1	1	0	GO Biological Processes	19	regulation of muscle adaptation	-4.52269	10.11777	7.054031	30242	98	183	6	3.278689	1.316393	148|4846|5465|6331|7132|9475	ADRA1A|NOS3|PPARA|SCN5A|TNFRSF1A|ROCK2	-3.35649	0	149	1	1	-4.52269	10.11776514
1	-4.4977	GO:0003300	M1	1	0	GO Biological Processes	19	cardiac muscle hypertrophy	-4.4977	10.01557	7.010576	30242	99	183	6	3.278689	1.316393	148|3066|3357|5465|7132|9475	ADRA1A|HDAC2|HTR2B|PPARA|TNFRSF1A|ROCK2	-3.33635	0	149	0	0	-4.52269	10.11776514
1	-4.42441	GO:0014897	M1	1	0	GO Biological Processes	19	striated muscle hypertrophy	-4.42441	9.72099	6.883837	30242	102	183	6	3.278689	1.316393	148|3066|3357|5465|7132|9475	ADRA1A|HDAC2|HTR2B|PPARA|TNFRSF1A|ROCK2	-3.27179	0	149	0	0	-4.52269	10.11776514
1	-4.37687	GO:0014896	M1	1	0	GO Biological Processes	19	muscle hypertrophy	-4.37687	9.534048	6.802223	30242	104	183	6	3.278689	1.316393	148|3066|3357|5465|7132|9475	ADRA1A|HDAC2|HTR2B|PPARA|TNFRSF1A|ROCK2	-3.22777	0	149	0	0	-4.52269	10.11776514
1	-4.01007	GO:0043500	M1	1	0	GO Biological Processes	19	muscle adaptation	-4.01007	8.194554	6.187258	30242	121	183	6	3.278689	1.316393	148|4846|5465|6331|7132|9475	ADRA1A|NOS3|PPARA|SCN5A|TNFRSF1A|ROCK2	-2.89687	0	149	0	0	-4.52269	10.11776514
1	-3.08337	GO:0010611	M1	1	0	GO Biological Processes	19	regulation of cardiac muscle hypertrophy	-3.08337	9.580106	5.567296	30242	69	183	4	2.185792	1.080886	148|5465|7132|9475	ADRA1A|PPARA|TNFRSF1A|ROCK2	-2.07151	0	149	0	0	-4.52269	10.11776514
1	-3.01392	GO:0014743	M1	1	0	GO Biological Processes	19	regulation of muscle hypertrophy	-3.01392	9.180935	5.422729	30242	72	183	4	2.185792	1.080886	148|5465|7132|9475	ADRA1A|PPARA|TNFRSF1A|ROCK2	-2.00966	0	149	0	0	-4.52269	10.11776514
1	-4.42441	GO:0002444	M1	1	0	GO Biological Processes	19	myeloid leukocyte mediated immunity	-4.42441	9.72099	6.883837	30242	102	183	6	3.278689	1.316393	1636|1991|2147|3569|5293|729230	ACE|ELANE|F2|IL6|PIK3CD|CCR2	-3.27179	0	150	1	1	-4.42441	18.36187007
1	-4.18252	GO:0002446	M1	1	0	GO Biological Processes	19	neutrophil mediated immunity	-4.18252	18.36187	8.132751	30242	36	183	4	2.185792	1.080886	1636|1991|2147|3569	ACE|ELANE|F2|IL6	-3.05249	0	150	0	0	-4.42441	18.36187007
1	-3.11805	GO:0006959	M1	1	0	GO Biological Processes	19	humoral immune response	-3.11805	4.144372	4.405174	30242	319	183	8	4.371585	1.511428	596|1991|2147|3569|3576|6347|7124|729230	BCL2|ELANE|F2|IL6|CXCL8|CCL2|TNF|CCR2	-2.10105	0	150	0	0	-4.42441	18.36187007
1	-2.14725	GO:0001909	M1	1	0	GO Biological Processes	19	leukocyte mediated cytotoxicity	-2.14725	5.288219	3.748542	30242	125	183	4	2.185792	1.080886	1991|2147|3383|4843	ELANE|F2|ICAM1|NOS2	-1.22496	0	150	0	0	-4.42441	18.36187007
1	-2.08679	GO:0031640	M1	1	0	GO Biological Processes	19	killing of cells of other organism	-2.08679	7.290743	4.052064	30242	68	183	3	1.639344	0.938686	1991|2147|4843	ELANE|F2|NOS2	-1.16956	0	150	0	0	-4.42441	18.36187007
1	-4.35943	GO:0007265	M1	1	0	GO Biological Processes	19	Ras protein signal transduction	-4.35943	4.889255	5.610435	30242	338	183	10	5.464481	1.680144	148|150|185|890|2149|4914|5159|5291|7157|9475	ADRA1A|ADRA2A|AGTR1|CCNA2|F2R|NTRK1|PDGFRB|PIK3CB|TP53|ROCK2	-3.21112	0	151	1	1	-4.35943	10.32855191
1	-3.69744	GO:0007266	M1	1	0	GO Biological Processes	19	Rho protein signal transduction	-3.69744	7.18508	5.682103	30242	138	183	6	3.278689	1.316393	148|150|185|2149|5159|9475	ADRA1A|ADRA2A|AGTR1|F2R|PDGFRB|ROCK2	-2.62334	0	151	0	0	-4.35943	10.32855191
1	-3.20691	GO:0046579	M1	1	0	GO Biological Processes	19	positive regulation of Ras protein signal transduction	-3.20691	10.32855	5.829017	30242	64	183	4	2.185792	1.080886	2149|4914|5159|5291	F2R|NTRK1|PDGFRB|PIK3CB	-2.18207	0	151	0	0	-4.35943	10.32855191

<table>
<tr><td>1</td><td>-3.01392</td><td>GO:0051057</td><td>M1</td><td>1</td><td>0</td><td>GO Biological Processes</td><td>19</td><td>positive regulation of small GTPase mediated signal transduction</td><td>-3.01392</td><td>9.180935</td><td>5.422729</td><td>30242</td><td>72</td><td>183</td><td>4</td><td>2.185792</td><td>1.080886</td><td>2149|4914|5159|5291</td><td>F2R|NTRK1|PDGFRB|PIK3CB</td><td>-2.00966</td><td>0</td><td>151</td><td>0</td><td>0</td><td>-4.35943</td><td>10.32855191</td></tr>
<tr><td>1</td><td>-2.984</td><td>GO:0007264</td><td>M1</td><td>1</td><td>0</td><td>GO Biological Processes</td><td>19</td><td>small GTPase mediated signal transduction</td><td>-2.984</td><td>3.285424</td><td>4.03296</td><td>30242</td><td>503</td><td>183</td><td>10</td><td>5.464481</td><td>1.680144</td><td>148|150|185|890|2149|4914|5159|5291|7157|9475</td><td>ADRA1A|ADRA2A|AGTR1|CCNA2|F2R|NTRK1|PDGFRB|PIK3CB|TP53|ROCK2</td><td>-1.98159</td><td>0</td><td>151</td><td>0</td><td>0</td><td>-4.35943</td><td>10.32855191</td></tr>
<tr><td>1</td><td>-2.21368</td><td>GO:0046578</td><td>M1</td><td>1</td><td>0</td><td>GO Biological Processes</td><td>19</td><td>regulation of Ras protein signal transduction</td><td>-2.21368</td><td>4.348864</td><td>3.61305</td><td>30242</td><td>190</td><td>183</td><td>5</td><td>2.73224</td><td>1.205087</td><td>148|2149|4914|5159|5291</td><td>ADRA1A|F2R|NTRK1|PDGFRB|PIK3CB</td><td>-1.28721</td><td>0</td><td>151</td><td>0</td><td>0</td><td>-4.35943</td><td>10.32855191</td></tr>
<tr><td>1</td><td>-4.34936</td><td>GO:0050866</td><td>M1</td><td>1</td><td>0</td><td>GO Biological Processes</td><td>19</td><td>negative regulation of cell activation</td><td>-4.34936</td><td>6.295498</td><td>6.008426</td><td>30242</td><td>210</td><td>183</td><td>8</td><td>4.371585</td><td>1.511428</td><td>135|472|836|1268|2147|3558|4846|729230</td><td>ADORA2A|ATM|CASP3|CNR1|F2|IL2|NOS3|CCR2</td><td>-3.20182</td><td>0</td><td>152</td><td>1</td><td>1</td><td>-4.34936</td><td>10.11776514</td></tr>
<tr><td>1</td><td>-2.99862</td><td>GO:0002695</td><td>M1</td><td>1</td><td>0</td><td>GO Biological Processes</td><td>19</td><td>negative regulation of leukocyte activation</td><td>-2.99862</td><td>5.302358</td><td>4.604743</td><td>30242</td><td>187</td><td>183</td><td>6</td><td>3.278689</td><td>1.316393</td><td>135|472|836|1268|3558|729230</td><td>ADORA2A|ATM|CASP3|CNR1|IL2|CCR2</td><td>-1.99464</td><td>0</td><td>152</td><td>0</td><td>0</td><td>-4.34936</td><td>10.11776514</td></tr>
<tr><td>1</td><td>-2.48503</td><td>GO:0008038</td><td>M1</td><td>1</td><td>0</td><td>GO Biological Processes</td><td>19</td><td>neuron recognition</td><td>-2.48503</td><td>10.11777</td><td>4.983904</td><td>30242</td><td>49</td><td>183</td><td>3</td><td>1.639344</td><td>0.938686</td><td>351|836|1268</td><td>APP|CASP3|CNR1</td><td>-1.53506</td><td>0</td><td>152</td><td>0</td><td>0</td><td>-4.34936</td><td>10.11776514</td></tr>
<tr><td>1</td><td>-4.30745</td><td>GO:0032526</td><td>M1</td><td>1</td><td>0</td><td>GO Biological Processes</td><td>19</td><td>response to retinoic acid</td><td>-4.30745</td><td>9.266738</td><td>6.683852</td><td>30242</td><td>107</td><td>183</td><td>6</td><td>3.278689</td><td>1.316393</td><td>472|2263|3066|5159|5979|6532</td><td>ATM|FGFR2|HDAC2|PDGFRB|RET|SLC6A4</td><td>-3.16454</td><td>0</td><td>153</td><td>1</td><td>1</td><td>-4.30745</td><td>12.51945686</td></tr>
<tr><td>1</td><td>-4.29322</td><td>GO:0071300</td><td>M1</td><td>1</td><td>0</td><td>GO Biological Processes</td><td>19</td><td>cellular response to retinoic acid</td><td>-4.29322</td><td>12.51946</td><td>7.309867</td><td>30242</td><td>66</td><td>183</td><td>5</td><td>2.73224</td><td>1.205087</td><td>472|2263|3066|5979|6532</td><td>ATM|FGFR2|HDAC2|RET|SLC6A4</td><td>-3.15147</td><td>0</td><td>153</td><td>0</td><td>0</td><td>-4.30745</td><td>12.51945686</td></tr>
<tr><td>1</td><td>-4.0543</td><td>GO:0051881</td><td>M1</td><td>1</td><td>0</td><td>GO Biological Processes</td><td>19</td><td>regulation of mitochondrial membrane potential</td><td>-4.0543</td><td>11.166</td><td>6.831706</td><td>30242</td><td>74</td><td>183</td><td>5</td><td>2.73224</td><td>1.205087</td><td>135|596|834|4985|7442</td><td>ADORA2A|BCL2|CASP1|OPRD1|TRPV1</td><td>-2.93678</td><td>0</td><td>154</td><td>1</td><td>1</td><td>-4.0543</td><td>11.16600207</td></tr>
<tr><td>1</td><td>-4.01007</td><td>GO:0042177</td><td>M1</td><td>1</td><td>0</td><td>GO Biological Processes</td><td>19</td><td>negative regulation of protein catabolic process</td><td>-4.01007</td><td>8.194554</td><td>6.187258</td><td>30242</td><td>121</td><td>183</td><td>6</td><td>3.278689</td><td>1.316393</td><td>1956|2903|3156|4843|5582|5970</td><td>EGFR|GRIN2A|HMGCR|NOS2|PRKCG|RELA</td><td>-2.89687</td><td>0</td><td>155</td><td>1</td><td>1</td><td>-4.01007</td><td>8.194553584</td></tr>
<tr><td>1</td><td>-3.17691</td><td>GO:0042176</td><td>M1</td><td>1</td><td>0</td><td>GO Biological Processes</td><td>19</td><td>regulation of protein catabolic process</td><td>-3.17691</td><td>3.803866</td><td>4.354141</td><td>30242</td><td>391</td><td>183</td><td>9</td><td>4.918033</td><td>1.598524</td><td>150|1453|1956|2903|3156|4843|5582|5970|7124</td><td>ADRA2A|CSNK1D|EGFR|GRIN2A|HMGCR|NOS2|PRKCG|RELA|TNF</td><td>-2.15557</td><td>0</td><td>155</td><td>0</td><td>0</td><td>-4.01007</td><td>8.194553584</td></tr>
<tr><td>1</td><td>-3.99998</td><td>GO:0032094</td><td>M1</td><td>1</td><td>0</td><td>GO Biological Processes</td><td>19</td><td>response to food</td><td>-3.99998</td><td>16.52568</td><td>7.666537</td><td>30242</td><td>40</td><td>183</td><td>4</td><td>2.185792</td><td>1.080886</td><td>1543|4988|5465|5582</td><td>CYP1A1|OPRM1|PPARA|PRKCG</td><td>-2.8875</td><td>0</td><td>156</td><td>1</td><td>1</td><td>-3.99998</td><td>27.5428051</td></tr>
<tr><td>1</td><td>-3.77899</td><td>GO:0032095</td><td>M1</td><td>1</td><td>0</td><td>GO Biological Processes</td><td>19</td><td>regulation of response to food</td><td>-3.77899</td><td>27.54281</td><td>8.789081</td><td>30242</td><td>18</td><td>183</td><td>3</td><td>1.639344</td><td>0.938686</td><td>4988|5465|5582</td><td>OPRM1|PPARA|PRKCG</td><td>-2.69427</td><td>0</td><td>156</td><td>0</td><td>0</td><td>-3.99998</td><td>27.5428051</td></tr>
<tr><td>1</td><td>-3.5109</td><td>GO:0032098</td><td>M1</td><td>1</td><td>0</td><td>GO Biological Processes</td><td>19</td><td>regulation of appetite</td><td>-3.5109</td><td>22.53502</td><td>7.883985</td><td>30242</td><td>22</td><td>183</td><td>3</td><td>1.639344</td><td>0.938686</td><td>3358|4988|5465</td><td>HTR2C|OPRM1|PPARA</td><td>-2.45542</td><td>0</td><td>156</td><td>0</td><td>0</td><td>-3.99998</td><td>27.5428051</td></tr>
<tr><td>1</td><td>-3.34249</td><td>GO:0032104</td><td>M1</td><td>1</td><td>0</td><td>GO Biological Processes</td><td>19</td><td>regulation of response to extracellular stimulus</td><td>-3.34249</td><td>19.83082</td><td>7.349367</td><td>30242</td><td>25</td><td>183</td><td>3</td><td>1.639344</td><td>0.938686</td><td>4988|5465|5582</td><td>OPRM1|PPARA|PRKCG</td><td>-2.30396</td><td>0</td><td>156</td><td>0</td><td>0</td><td>-3.99998</td><td>27.5428051</td></tr>
<tr><td>1</td><td>-3.34249</td><td>GO:0032107</td><td>M1</td><td>1</td><td>0</td><td>GO Biological Processes</td><td>19</td><td>regulation of response to nutrient levels</td><td>-3.34249</td><td>19.83082</td><td>7.349367</td><td>30242</td><td>25</td><td>183</td><td>3</td><td>1.639344</td><td>0.938686</td><td>4988|5465|5582</td><td>OPRM1|PPARA|PRKCG</td><td>-2.30396</td><td>0</td><td>156</td><td>0</td><td>0</td><td>-3.99998</td><td>27.5428051</td></tr>
<tr><td>1</td><td>-3.97624</td><td>GO:0002253</td><td>M1</td><td>1</td><td>0</td><td>GO Biological Processes</td><td>19</td><td>activation of immune response</td><td>-3.97624</td><td>4.395128</td><td>5.168898</td><td>30242</td><td>376</td><td>183</td><td>10</td><td>5.464481</td><td>1.680144</td><td>100|596|3551|5142|5290|5293|5591|5970|7099|29110</td><td>ADA|BCL2|IKBKB|PDE4B|PIK3CA|PIK3CD|PRKDC|RELA|TLR4|TBK1</td><td>-2.8659</td><td>0</td><td>157</td><td>1</td><td>1</td><td>-3.97624</td><td>29.16297011</td></tr>
<tr><td>1</td><td>-3.97078</td><td>GO:0050852</td><td>M1</td><td>1</td><td>0</td><td>GO Biological Processes</td><td>19</td><td>T cell receptor signaling pathway</td><td>-3.97078</td><td>8.061309</td><td>6.122853</td><td>30242</td><td>123</td><td>183</td><td>6</td><td>3.278689</td><td>1.316393</td><td>100|3551|5142|5290|5293|5970</td><td>ADA|IKBKB|PDE4B|PIK3CA|PIK3CD|RELA</td><td>-2.86187</td><td>0</td><td>157</td><td>0</td><td>0</td><td>-3.97624</td><td>29.16297011</td></tr>
<tr><td>1</td><td>-3.85624</td><td>GO:2000811</td><td>M1</td><td>1</td><td>0</td><td>GO Biological Processes</td><td>19</td><td>negative regulation of anoikis</td><td>-3.85624</td><td>29.16297</td><td>9.062669</td><td>30242</td><td>17</td><td>183</td><td>3</td><td>1.639344</td><td>0.938686</td><td>596|4915|5290</td><td>BCL2|NTRK2|PIK3CA</td><td>-2.76097</td><td>0</td><td>157</td><td>0</td><td>0</td><td>-3.97624</td><td>29.16297011</td></tr>
</table>

1	-3.81504	GO:0002764	M1	1	0	GO Biological Processes	19	immune response-regulating signaling pathway	-3.81504	3.851324	4.871521	30242	472	183	11	6.010929	1.757049	100|596|2099|3551|4792|5142|5290|5293|5970|6197|7099	ADA|BCL2|ESR1|IKBKB|NFKBIA|PDE4B|PIK3CA|PIK3CD|RELA|RPS6KA3|TLR4	-2.72457	0	157	0	0	-3.97624	29.16297011
1	-3.39607	GO:2000209	M1	1	0	GO Biological Processes	19	regulation of anoikis	-3.39607	20.6571	7.516725	30242	24	183	3	1.639344	0.938686	596|4915|5290	BCL2|NTRK2|PIK3CA	-2.35299	0	157	0	0	-3.97624	29.16297011
1	-3.16975	GO:0050851	M1	1	0	GO Biological Processes	19	antigen receptor-mediated signaling pathway	-3.16975	4.819991	4.635848	30242	240	183	7	3.825137	1.417845	100|596|3551|5142|5290|5293|5970	ADA|BCL2|IKBKB|PDE4B|PIK3CA|PIK3CD|RELA	-2.14899	0	157	0	0	-3.97624	29.16297011
1	-2.94459	GO:0043276	M1	1	0	GO Biological Processes	19	anoikis	-2.94459	14.58149	6.182471	30242	34	183	3	1.639344	0.938686	596|4915|5290	BCL2|NTRK2|PIK3CA	-1.94754	0	157	0	0	-3.97624	29.16297011
1	-2.68845	GO:0002429	M1	1	0	GO Biological Processes	19	immune response-activating cell surface receptor signaling pathway	-2.68845	3.97525	3.979245	30242	291	183	7	3.825137	1.417845	100|596|3551|5142|5290|5293|5970	ADA|BCL2|IKBKB|PDE4B|PIK3CA|PIK3CD|RELA	-1.717	0	157	0	0	-3.97624	29.16297011
1	-2.68845	GO:0002757	M1	1	0	GO Biological Processes	19	immune response-activating signal transduction	-2.68845	3.97525	3.979245	30242	291	183	7	3.825137	1.417845	100|596|3551|5142|5290|5293|5970	ADA|BCL2|IKBKB|PDE4B|PIK3CA|PIK3CD|RELA	-1.717	0	157	0	0	-3.97624	29.16297011
1	-2.4973	GO:0002768	M1	1	0	GO Biological Processes	19	immune response-regulating cell surface receptor signaling pathway	-2.4973	3.672374	3.720123	30242	315	183	7	3.825137	1.417845	100|596|3551|5142|5290|5293|5970	ADA|BCL2|IKBKB|PDE4B|PIK3CA|PIK3CD|RELA	-1.54461	0	157	0	0	-3.97624	29.16297011
1	-3.93862	GO:0070431	M1	1	0	GO Biological Processes	19	nucleotide-binding oligomerization domain containing 2 signaling pathway	-3.93862	30.98566	9.360942	30242	16	183	3	1.639344	0.938686	4792|5970|7099	NFKBIA|RELA|TLR4	-2.83536	0	158	1	1	-3.93862	30.98565574
1	-3.45213	GO:0035994	M1	1	0	GO Biological Processes	19	response to muscle stretch	-3.45213	21.55524	7.694542	30242	23	183	3	1.639344	0.938686	4792|5290|5970	NFKBIA|PIK3CA|RELA	-2.40475	0	158	0	0	-3.93862	30.98565574
1	-3.34249	GO:0070423	M1	1	0	GO Biological Processes	19	nucleotide-binding oligomerization domain containing signaling pathway	-3.34249	19.83082	7.349367	30242	25	183	3	1.639344	0.938686	4792|5970|7099	NFKBIA|RELA|TLR4	-2.30396	0	158	0	0	-3.93862	30.98565574
1	-3.29117	GO:0035872	M1	1	0	GO Biological Processes	19	nucleotide-binding domain, leucine rich repeat containing receptor signaling pathway	-3.29117	19.0681	7.191458	30242	26	183	3	1.639344	0.938686	4792|5970|7099	NFKBIA|RELA|TLR4	-2.25835	0	158	0	0	-3.93862	30.98565574
1	-2.38839	GO:0043620	M1	1	0	GO Biological Processes	19	regulation of DNA-templated transcription in response to stress	-2.38839	9.35416	4.749553	30242	53	183	3	1.639344	0.938686	5970|6197|7157	RELA|RPS6KA3|TP53	-1.44695	0	158	0	0	-3.93862	30.98565574
1	-2.35251	GO:0002221	M1	1	0	GO Biological Processes	19	pattern recognition receptor signaling pathway	-2.35251	4.694796	3.835706	30242	176	183	5	2.73224	1.205087	2099|4792|5970|6197|7099	ESR1|NFKBIA|RELA|RPS6KA3|TLR4	-1.41445	0	158	0	0	-3.93862	30.98565574
1	-2.21721	GO:0002753	M1	1	0	GO Biological Processes	19	cytoplasmic pattern recognition receptor signaling pathway	-2.21721	8.127385	4.347751	30242	61	183	3	1.639344	0.938686	4792|5970|7099	NFKBIA|RELA|TLR4	-1.29004	0	158	0	0	-3.93862	30.98565574
1	-2.15936	GO:0002224	M1	1	0	GO Biological Processes	19	toll-like receptor signaling pathway	-2.15936	5.330866	3.770586	30242	124	183	4	2.185792	1.080886	2099|4792|6197|7099	ESR1|NFKBIA|RPS6KA3|TLR4	-1.23591	0	158	0	0	-3.93862	30.98565574
1	-3.85212	GO:0043433	M1	1	0	GO Biological Processes	19	negative regulation of DNA-binding transcription factor	-3.85212	6.252961	5.591825	30242	185	183	7	3.825137	1.417845	140|1545|2099|3066|4221|4792|114548	ADORA3|CYP1B1|ESR1|HDAC2|MEN1|NFKBIA|NLRP3	-2.75789	0	159	1	1	-3.85212	7.107820671

								activity																		
1	-2.60359	GO:0032088	M1	1	0	GO Biological Processes	19	negative regulation of NF-kappaB transcription factor activity	-2.60359	7.107821	4.602859	30242	93	183	4	2.185792	1.080886	140|1545|4792|114548	ADORA3|CYP1B1|NFKBIA|NLRP3	-1.64263	0	159	0	0	-3.85212	7.107820671
1	-3.77899	GO:0033189	M1	1	0	GO Biological Processes	19	response to vitamin A	-3.77899	27.54281	8.789081	30242	18	183	3	1.639344	0.938686	1543|5467|7298	CYP1A1|PPARD|TYMS	-2.69427	0	160	1	1	-3.77899	27.5428051
1	-3.30456	GO:0006766	M1	1	0	GO Biological Processes	19	vitamin metabolic process	-3.30456	7.722282	5.435123	30242	107	183	5	2.73224	1.205087	1543|4363|5467|6513|7124	CYP1A1|ABCC1|PPARD|SLC2A1|TNF	-2.26935	0	160	0	0	-3.77899	27.5428051
1	-2.6188	GO:0006775	M1	1	0	GO Biological Processes	19	fat-soluble vitamin metabolic process	-2.6188	11.26751	5.317883	30242	44	183	3	1.639344	0.938686	1543|5467|7124	CYP1A1|PPARD|TNF	-1.65683	0	160	0	0	-3.77899	27.5428051
1	-3.77583	GO:0061448	M1	1	0	GO Biological Processes	19	connective tissue development	-3.77583	5.20494	5.250966	30242	254	183	8	4.371585	1.511428	5159|5290|5467|7040|7046|7298|10135|59341	PDGFRB|PIK3CA|PPARD|TGFB1|TGFBR1|TYMS|NAMPT|TRPV4	-2.69175	0	161	1	1	-3.77583	11.80405933
1	-2.67703	GO:0060612	M1	1	0	GO Biological Processes	19	adipose tissue development	-2.67703	11.80406	5.466942	30242	42	183	3	1.639344	0.938686	5290|5467|10135	PIK3CA|PPARD|NAMPT	-1.70713	0	161	0	0	-3.77583	11.80405933
1	-2.48503	GO:0043457	M1	1	0	GO Biological Processes	19	regulation of cellular respiration	-2.48503	10.11777	4.983904	30242	49	183	3	1.639344	0.938686	4843|5290|59341	NOS2|PIK3CA|TRPV4	-1.53506	0	161	0	0	-3.77583	11.80405933
1	-3.7239	GO:0007595	M1	1	0	GO Biological Processes	19	lactation	-3.7239	14.06441	6.99371	30242	47	183	4	2.185792	1.080886	595|1723|6531|7421	CCND1|DHODH|SLC6A3|VDR	-2.64452	0	162	1	1	-3.7239	14.06441111
1	-3.72127	GO:0051262	M1	1	0	GO Biological Processes	19	protein tetramerization	-3.72127	9.497519	6.193105	30242	87	183	5	2.73224	1.205087	2902|2904|7157|7442|8989	GRIN1|GRIN2B|TP53|TRPV1|TRPA1	-2.64322	0	163	1	1	-3.72127	9.497519
1	-3.19115	GO:0051259	M1	1	0	GO Biological Processes	19	protein complex oligomerization	-3.19115	4.860495	4.665286	30242	238	183	7	3.825137	1.417845	2902|2904|5024|7157|7442|8989|10280	GRIN1|GRIN2B|P2RX3|TP53|TRPV1|TRPA1|SIGMAR1	-2.16777	0	163	0	0	-3.72127	9.497519
1	-3.65322	GO:1904707	M1	1	0	GO Biological Processes	19	positive regulation of vascular associated smooth muscle cell proliferation	-3.65322	13.49035	6.82741	30242	49	183	4	2.185792	1.080886	3351|4313|4318|7124	HTR1B|MMP2|MMP9|TNF	-2.58304	0	164	1	1	-3.65322	13.49035352
1	-3.65228	GO:1904705	M1	1	0	GO Biological Processes	19	regulation of vascular associated smooth muscle cell proliferation	-3.65228	9.180935	6.064605	30242	90	183	5	2.73224	1.205087	3351|4313|4318|5468|7124	HTR1B|MMP2|MMP9|PPARG|TNF	-2.58242	0	164	0	0	-3.65322	13.49035352
1	-3.62985	GO:1990874	M1	1	0	GO Biological Processes	19	vascular associated smooth muscle cell proliferation	-3.62985	9.080046	6.023099	30242	91	183	5	2.73224	1.205087	3351|4313|4318|5468|7124	HTR1B|MMP2|MMP9|PPARG|TNF	-2.56226	0	164	0	0	-3.65322	13.49035352
1	-3.58562	GO:0048013	M1	1	0	GO Biological Processes	19	ephrin receptor signaling pathway	-3.58562	12.96132	6.670552	30242	51	183	4	2.185792	1.080886	2050|4313|4318|4914	EPHB4|MMP2|MMP9|NTRK1	-2.5219	0	164	0	0	-3.65322	13.49035352
1	-3.52087	GO:0007566	M1	1	0	GO Biological Processes	19	embryo implantation	-3.52087	12.47221	6.522246	30242	53	183	4	2.185792	1.080886	4313|4318|5467|5743	MMP2|MMP9|PPARD|PTGS2	-2.46287	0	164	0	0	-3.65322	13.49035352
1	-2.84343	GO:0071229	M1	1	0	GO Biological Processes	19	cellular response to acid chemical	-2.84343	8.262842	5.075255	30242	80	183	4	2.185792	1.080886	4313|4915|5290|7124	MMP2|NTRK2|PIK3CA|TNF	-1.85509	0	164	0	0	-3.65322	13.49035352
1	-2.59076	GO:0035987	M1	1	0	GO Biological Processes	19	endodermal cell differentiation	-2.59076	11.01712	5.246911	30242	45	183	3	1.639344	0.938686	2335|4313|4318	FN1|MMP2|MMP9	-1.63232	0	164	0	0	-3.65322	13.49035352
1	-2.36548	GO:0001706	M1	1	0	GO Biological Processes	19	endoderm formation	-2.36548	9.180935	4.694821	30242	54	183	3	1.639344	0.938686	2335|4313|4318	FN1|MMP2|MMP9	-1.4267	0	164	0	0	-3.65322	13.49035352
1	-2.26179	GO:0007369	M1	1	0	GO Biological Processes	19	gastrulation	-2.26179	4.466401	3.690015	30242	185	183	5	2.73224	1.205087	2263|2335|4313|4318|7157	FGFR2|FN1|MMP2|MMP9|TP53	-1.33063	0	164	0	0	-3.65322	13.49035352
1	-2.19639	GO:0001704	M1	1	0	GO Biological Processes	19	formation of primary germ layer	-2.19639	5.463036	3.838174	30242	121	183	4	2.185792	1.080886	2263|2335|4313|4318	FGFR2|FN1|MMP2|MMP9	-1.27108	0	164	0	0	-3.65322	13.49035352

1	-2.03543	GO:0071230	M1	1	0	GO Biological Processes	19	cellular response to amino acid stimulus	-2.03543	6.982683	3.937918	30242	71	183	3	1.639344	0.938686	4313|4915|7124	MMP2|NTRK2|TNF	-1.12071	0	164	0	0	-3.65322	13.49035352
1	-3.60769	GO:0070509	M1	1	0	GO Biological Processes	19	calcium ion import	-3.60769	8.981349	5.982228	30242	92	183	5	2.73224	1.205087	775|5159|6347|7442|59341	CACNA1C|PDGFRB|CCL2|TRPV1|TRPV4	-2.54172	0	165	1	1	-3.60769	13.04659189
1	-3.45874	GO:1905517	M1	1	0	GO Biological Processes	19	macrophage migration	-3.45874	12.01868	6.381713	30242	55	183	4	2.185792	1.080886	1910|6347|59341|729230	EDNRB|CCL2|TRPV4|CCR2	-2.40826	0	165	0	0	-3.60769	13.04659189
1	-2.80314	GO:0048246	M1	1	0	GO Biological Processes	19	macrophage chemotaxis	-2.80314	13.04659	5.797758	30242	38	183	3	1.639344	0.938686	1910|6347|59341	EDNRB|CCL2|TRPV4	-1.82068	0	165	0	0	-3.60769	13.04659189
1	-3.5529	GO:0002218	M1	1	0	GO Biological Processes	19	activation of innate immune response	-3.5529	12.71206	6.595381	30242	52	183	4	2.185792	1.080886	3551|5591|7099|29110	IKBKB|PRKDC|TLR4|TBK1	-2.49333	0	166	1	1	-3.5529	12.71206389
1	-3.32425	GO:0060759	M1	1	0	GO Biological Processes	19	regulation of response to cytokine stimulus	-3.32425	6.120623	5.098915	30242	162	183	6	3.278689	1.316393	834|3551|3569|5468|7099|29110	CASP1|IKBKB|IL6|PPARG|TLR4|TBK1	-2.28723	0	166	0	0	-3.5529	12.71206389
1	-2.66044	GO:0045088	M1	1	0	GO Biological Processes	19	regulation of innate immune response	-2.66044	4.548353	4.102581	30242	218	183	6	3.278689	1.316393	1813|3551|5468|5591|7099|29110	DRD2|IKBKB|PPARG|PRKDC|TLR4|TBK1	-1.69311	0	166	0	0	-3.5529	12.71206389
1	-2.64955	GO:0001959	M1	1	0	GO Biological Processes	19	regulation of cytokine-mediated signaling pathway	-2.64955	5.508561	4.319111	30242	150	183	5	2.73224	1.205087	834|3551|3569|5468|29110	CASP1|IKBKB|IL6|PPARG|TBK1	-1.68325	0	166	0	0	-3.5529	12.71206389
1	-2.29947	GO:0060760	M1	1	0	GO Biological Processes	19	positive regulation of response to cytokine stimulus	-2.29947	8.697728	4.538797	30242	57	183	3	1.639344	0.938686	834|7099|29110	CASP1|TLR4|TBK1	-1.36665	0	166	0	0	-3.5529	12.71206389
1	-2.07692	GO:0045089	M1	1	0	GO Biological Processes	19	positive regulation of innate immune response	-2.07692	5.04601	3.621065	30242	131	183	4	2.185792	1.080886	3551|5591|7099|29110	IKBKB|PRKDC|TLR4|TBK1	-1.16015	0	166	0	0	-3.5529	12.71206389
1	-3.48949	GO:0006968	M1	1	0	GO Biological Processes	19	cellular defense response	-3.48949	12.24125	6.451052	30242	54	183	4	2.185792	1.080886	135|136|5970|729230	ADORA2A|ADORA2B|RELA|CCR2	-2.43683	0	167	1	1	-3.48949	12.24124671
1	-3.31284	GO:0016485	M1	1	0	GO Biological Processes	19	protein processing	-3.31284	5.096026	4.833289	30242	227	183	7	3.825137	1.417845	834|836|1636|2155|2903|4325|23621	CASP1|CASP3|ACE|F7|GRIN2A|MMP16|BACE1	-2.27673	0	168	1	1	-3.31284	5.096025613
1	-2.64701	GO:0051604	M1	1	0	GO Biological Processes	19	protein maturation	-2.64701	3.908101	3.923036	30242	296	183	7	3.825137	1.417845	834|836|1636|2155|2903|4325|23621	CASP1|CASP3|ACE|F7|GRIN2A|MMP16|BACE1	-1.68249	0	168	0	0	-3.31284	5.096025613
1	-3.28608	GO:0098659	M1	1	0	GO Biological Processes	19	inorganic cation import across plasma membrane	-3.28608	7.650779	5.40247	30242	108	183	5	2.73224	1.205087	3757|6523|7442|51305|59341	KCNH2|SLC5A1|TRPV1|KCNK9|TRPV4	-2.25445	0	169	1	1	-3.28608	7.650779194
1	-3.28608	GO:0099587	M1	1	0	GO Biological Processes	19	inorganic ion import across plasma membrane	-3.28608	7.650779	5.40247	30242	108	183	5	2.73224	1.205087	3757|6523|7442|51305|59341	KCNH2|SLC5A1|TRPV1|KCNK9|TRPV4	-2.25445	0	169	0	0	-3.28608	7.650779194
1	-3.10958	GO:0098739	M1	1	0	GO Biological Processes	19	import across plasma membrane	-3.10958	5.570455	4.771802	30242	178	183	6	3.278689	1.316393	3757|6513|6523|7442|51305|59341	KCNH2|SLC2A1|SLC5A1|TRPV1|KCNK9|TRPV4	-2.09344	0	169	0	0	-3.28608	7.650779194
1	-3.10534	GO:0008209	M1	1	0	GO Biological Processes	19	androgen metabolic process	-3.10534	16.52568	6.638317	30242	30	183	3	1.639344	0.938686	1588|2099|7364	CYP19A1|ESR1|UGT2B7	-2.09149	0	170	1	1	-3.10534	16.52568306
1	-2.64755	GO:0140353	M1	1	0	GO Biological Processes	19	lipid export from cell	-2.64755	11.52955	5.391182	30242	43	183	3	1.639344	0.938686	185|1588|4843	AGTR1|CYP19A1|NOS2	-1.68278	0	170	0	0	-3.10534	16.52568306
1	-3.01392	GO:0033627	M1	1	0	GO Biological Processes	19	cell adhesion mediated by integrin	-3.01392	9.180935	5.422729	30242	72	183	4	2.185792	1.080886	100|1545|3383|5979	ADA|CYP1B1|ICAM1|RET	-2.00966	0	171	1	1	-3.01392	10.32855191
1	-2.51055	GO:0033628	M1	1	0	GO Biological Processes	19	regulation of cell adhesion mediated by integrin	-2.51055	10.32855	5.046739	30242	48	183	3	1.639344	0.938686	100|1545|5979	ADA|CYP1B1|RET	-1.55686	0	171	0	0	-3.01392	10.32855191
1	-2.83693	GO:0032570	M1	1	0	GO Biological	19	response to	-2.83693	13.3992	5.888322	30242	37	183	3	1.639344	0.938686	5970|7040|7298	RELA|TGFB1|TYMS	-1.8502	0	172	1	1	-2.83693	13.39920248

						Processes		progesterone																		
1	-2.67703	GO:0045687	M1	1	0	GO Biological Processes	19	positive regulation of glial cell differentiation	-2.67703	11.80406	5.466942	30242	42	183	3	1.639344	0.938686	3066|5970|7040	HDAC2|RELA|TGFB1	-1.70713	0	172	0	0	-2.83693	13.39920248
1	-2.2372	GO:0006081	M1	1	0	GO Biological Processes	19	cellular aldehyde metabolic process	-2.2372	8.262842	4.393843	30242	60	183	3	1.639344	0.938686	1545|1645|5970	CYP1B1|AKR1C1|RELA	-1.30863	0	172	0	0	-2.83693	13.39920248
1	-2.82192	GO:0000086	M1	1	0	GO Biological Processes	19	G2/M transition of mitotic cell cycle	-2.82192	6.031271	4.605269	30242	137	183	5	2.73224	1.205087	351|472|595|890|5243	APP|ATM|CCND1|CCNA2|ABCB1	-1.83706	0	173	1	1	-2.82192	12.71206389
1	-2.77031	GO:0016572	M1	1	0	GO Biological Processes	19	histone phosphorylation	-2.77031	12.71206	5.710538	30242	39	183	3	1.639344	0.938686	472|890|8986	ATM|CCNA2|RPS6KA4	-1.79128	0	173	0	0	-2.82192	12.71206389
1	-2.6749	GO:0044839	M1	1	0	GO Biological Processes	19	cell cycle G2/M phase transition	-2.6749	5.583001	4.360911	30242	148	183	5	2.73224	1.205087	351|472|595|890|5243	APP|ATM|CCND1|CCNA2|ABCB1	-1.70578	0	173	0	0	-2.82192	12.71206389
1	-2.74631	GO:0042440	M1	1	0	GO Biological Processes	19	pigment metabolic process	-2.74631	7.776792	4.881754	30242	85	183	4	2.185792	1.080886	100|596|4363|5498	ADA|BCL2|ABCC1|PPOX	-1.76965	0	174	1	1	-2.74631	7.776792028
1	-2.73837	GO:0071276	M1	1	0	GO Biological Processes	19	cellular response to cadmium ion	-2.73837	12.39426	5.626453	30242	40	183	3	1.639344	0.938686	1544|1956|4318	CYP1A2|EGFR|MMP9	-1.7638	0	175	1	1	-2.73837	12.3942623
1	-2.08679	GO:0046686	M1	1	0	GO Biological Processes	19	response to cadmium ion	-2.08679	7.290743	4.052064	30242	68	183	3	1.639344	0.938686	1544|1956|4318	CYP1A2|EGFR|MMP9	-1.16956	0	175	0	0	-2.73837	12.3942623
1	-2.73837	GO:0009112	M1	1	0	GO Biological Processes	19	nucleobase metabolic process	-2.73837	12.39426	5.626453	30242	40	183	3	1.639344	0.938686	100|1723|7298	ADA|DHODH|TYMS	-1.7638	0	176	1	1	-2.73837	12.3942623
1	-2.67703	GO:0009124	M1	1	0	GO Biological Processes	19	nucleoside monophosphate biosynthetic process	-2.67703	11.80406	5.466942	30242	42	183	3	1.639344	0.938686	100|1723|7298	ADA|DHODH|TYMS	-1.70713	0	176	0	0	-2.73837	12.3942623
1	-2.49395	GO:0051100	M1	1	0	GO Biological Processes	19	negative regulation of binding	-2.49395	5.069228	4.064531	30242	163	183	5	2.73224	1.205087	154|1636|3066|4792|5465	ADRB2|ACE|HDAC2|NFKBIA|PPARA	-1.54175	0	177	1	1	-2.49395	5.069227933
1	-2.39911	GO:0090263	M1	1	0	GO Biological Processes	19	positive regulation of canonical Wnt signaling pathway	-2.39911	6.236107	4.213616	30242	106	183	4	2.185792	1.080886	1453|1956|2263|7040	CSNK1D|EGFR|FGFR2|TGFB1	-1.45671	0	178	1	1	-2.39911	8.5477671
1	-2.27832	GO:0007405	M1	1	0	GO Biological Processes	19	neuroblast proliferation	-2.27832	8.547767	4.489319	30242	58	183	3	1.639344	0.938686	1813|2263|7157	DRD2|FGFR2|TP53	-1.34621	0	178	0	0	-2.39911	8.5477671
1	-2.2164	GO:0198738	M1	1	0	GO Biological Processes	19	cell-cell signaling by wnt	-2.2164	2.957617	3.253465	30242	447	183	8	4.371585	1.511428	351|595|1453|1813|1956|2263|4988|7040	APP|CCND1|CSNK1D|DRD2|EGFR|FGFR2|OPRM1|TGFB1	-1.28947	0	178	0	0	-2.39911	8.5477671
1	-2.36294	GO:0022900	M1	1	0	GO Biological Processes	19	electron transport chain	-2.36294	4.721624	3.852501	30242	175	183	5	2.73224	1.205087	217|1544|1588|2936|4129	ALDH2|CYP1A2|CYP19A1|GSR|MAOB	-1.4244	0	179	1	1	-2.36294	4.721623731
1	-2.12246	GO:2000756	M1	1	0	GO Biological Processes	19	regulation of peptidyl-lysine acetylation	-2.12246	7.511674	4.132094	30242	66	183	3	1.639344	0.938686	3066|4842|8986	HDAC2|NOS1|RPS6KA4	-1.20133	0	180	1	1	-2.12246	7.511674118
1	-2.08679	GO:0007585	M1	1	0	GO Biological Processes	19	respiratory gaseous exchange by respiratory system	-2.08679	7.290743	4.052064	30242	68	183	3	1.639344	0.938686	134|1909|10800	ADORA1|EDNRA|CYSLTR1	-1.16956	0	181	1	1	-2.08679	7.290742527
1	-34.1199	GO:0097060	M1	1	0	GO Cellular Components	20	synaptic membrane	-34.1199	16.35354	23.62545	30242	384	183	38	20.76503	2.998466	134|135|148|152|775|1128|1129|1131|1132|1133|1268|1813|1814|2149|2554|2558|2566|2891|2902|2903|2904|2915|3351|3356|3778|4985|4986|4988|5024|5138|5582|6326|6336|6530|6531|6532|7442|10280	ADORA1|ADORA2A|ADRA1A|ADRA2C|CACNA1C|CHRM1|CHRM2|CHRM3|CHRM4|CHRM5|CNR1|DRD2|DRD3|F2R|GABRA1|GABRA5|GABRG2|GRIA2|GRIN1|GRIN2A|GRIN2B|GRM5|HTR1B|HTR2A|KCNMA1|OPRD1|OPRK1|OPRM1|P2RX3|PDE2A|PRKC	-30.8414	0	1	1	1	-34.1199	42.56615334

																			G|SCN2A|SCN10A|SLC6A2|SLC6A3|SLC6A4|TRPV1|SIGMAR1							
1	-28.0169	GO:0045211	M1	1	0	GO Cellular Components	20	postsynaptic membrane	-28.0169	17.89785	22.04452	30242	277	183	30	16.39344	2.736716	134|135|148|152|775|1128|1129|1131|1132|1133|1813|1814|2149|2554|2558|2566|2891|2902|2903|2904|2915|3356|3778|4985|4986|4988|6531|6532|7442|10280	ADORA1|ADORA2A|ADRA1A|ADRA2C|CACNA1C|CHRM1|CHRM2|CHRM3|CHRM4|CHRM5|DRD2|DRD3|F2R|GABRA1|GABRA5|GABRG2|GRIA2|GRIN1|GRIN2A|GRIN2B|GRM5|HTR2A|KCNMA1|OPRD1|OPRK1|OPRM1|SLC6A3|SLC6A4|TRPV1|SIGMAR1	-25.3405	0	1	0	0	-34.1199	42.56615334
1	-26.0799	GO:0098794	M1	1	0	GO Cellular Components	20	postsynapse	-26.0799	9.936328	17.71474	30242	632	183	38	20.76503	2.998466	134|135|148|152|351|775|1128|1129|1131|1132|1133|1812|1813|1814|1815|2149|2554|2558|2566|2891|2902|2903|2904|2915|3356|3778|4842|4915|4985|4986|4988|5024|5142|5582|6531|6532|7442|10280	ADORA1|ADORA2A|ADRA1A|ADRA2C|APP|CACNA1C|CHRM1|CHRM2|CHRM3|CHRM4|CHRM5|DRD1|DRD2|DRD3|DRD4|F2R|GABRA1|GABRA5|GABRG2|GRIA2|GRIN1|GRIN2A|GRIN2B|GRM5|HTR2A|KCNMA1|NOS1|NTRK2|OPRD1|OPRK1|OPRM1|P2RX3|PDE4B|PRKCG|SLC6A3|SLC6A4|TRPV1|SIGMAR1	-23.6464	0	1	0	0	-34.1199	42.56615334
1	-22.7497	GO:0099056	M1	1	0	GO Cellular Components	20	integral component of presynaptic membrane	-22.7497	42.56615	26.37651	30242	66	183	17	9.289617	2.145862	134|135|148|1128|1268|1813|2558|3351|3356|4985|4986|4988|5024|6326|6336|6531|6532	ADORA1|ADORA2A|ADRA1A|CHRM1|CNR1|DRD2|GABRA5|HTR1B|HTR2A|OPRD1|OPRK1|OPRM1|P2RX3|SCN2A|SCN10A|SLC6A3|SLC6A4	-20.3742	0	1	0	0	-34.1199	42.56615334
1	-22.0324	GO:0099699	M1	1	0	GO Cellular Components	20	integral component of synaptic membrane	-22.0324	23.13596	21.2058	30242	150	183	21	11.47541	2.356083	134|135|148|152|1128|1268|1813|1814|2554|2558|2903|3351|3356|4985|4986|4988|5024|6326|6336|6531|6532	ADORA1|ADORA2A|ADRA1A|ADRA2C|CHRM1|CNR1|DRD2|DRD3|GABRA1|GABRA5|GRIN2A|HTR1B|HTR2A|OPRD1|OPRK1|OPRM1|P2RX3|SCN2A|SCN10A|SLC6A3|SLC6A4	-19.7081	0	1	0	0	-34.1199	42.56615334
1	-21.6984	GO:0098889	M1	1	0	GO Cellular Components	20	intrinsic component of presynaptic membrane	-21.6984	37.45821	24.66586	30242	75	183	17	9.289617	2.145862	134|135|148|1128|1268|1813|2558|3351|3356|4985|4986|4988|5024|6326|6336|6531|6532	ADORA1|ADORA2A|ADRA1A|CHRM1|CNR1|DRD2|GABRA5|HTR1B|HTR2A|OPRD1|OPRK1|OPRM1|P2RX	-19.4612	0	1	0	0	-34.1199	42.56615334

																			3|SCN2A|SCN10A|SLC6A3|SLC6A4							
1	-21.2502	GO:0099240	M1	1	0	GO Cellular Components	20	intrinsic component of synaptic membrane	-21.2502	21.29076	20.26734	30242	163	183	21	11.47541	2.356083	134|135|148|152|1128|1268|1813|1814|2554|2558|2903|3351|3356|4985|4986|4988|5024|6326|6336|6531|6532	ADORA1|ADORA2A|ADRA1A|ADRA2C|CHRM1|CNR1|DRD2|DRD3|GABRA1|GABRA5|GRIN2A|HTR1B|HTR2A|OPRD1|OPRK1|OPRM1|P2RX3|SCN2A|SCN10A|SLC6A3|SLC6A4	-19.0509	0	1	0	0	-34.1199	42.56615334
1	-20.8017	GO:0042734	M1	1	0	GO Cellular Components	20	presynaptic membrane	-20.8017	22.63792	20.44909	30242	146	183	20	10.92896	2.306387	134|135|148|1128|1268|1813|2558|2903|3351|3356|4985|4986|4988|5024|5138|6326|6336|6530|6531|6532	ADORA1|ADORA2A|ADRA1A|CHRM1|CNR1|DRD2|GABRA5|GRIN2A|HTR1B|HTR2A|OPRD1|OPRK1|OPRM1|P2RX3|PDE2A|SCN2A|SCN10A|SLC6A2|SLC6A3|SLC6A4	-18.6371	0	1	0	0	-34.1199	42.56615334
1	-20.4067	GO:0098793	M1	1	0	GO Cellular Components	20	presynapse	-20.4067	9.856272	15.62817	30242	503	183	30	16.39344	2.736716	134|135|148|152|351|1128|1268|1813|2558|2902|2903|3351|3356|4915|4985|4986|4988|5024|5138|5142|5582|6326|6336|6513|6530|6531|6532|11255|23621|51305	ADORA1|ADORA2A|ADRA1A|ADRA2C|APP|CHRM1|CNR1|DRD2|GABRA5|GRIN1|GRIN2A|HTR1B|HTR2A|NTRK2|OPRD1|OPRK1|OPRM1|P2RX3|PDE2A|PDE4B|PRKCG|SCN2A|SCN10A|SLC2A1|SLC6A2|SLC6A3|SLC6A4|HRH3|BACE1|KCNK9	-18.2743	0	1	0	0	-34.1199	42.56615334
1	-16.707	GO:0099055	M1	1	0	GO Cellular Components	20	integral component of postsynaptic membrane	-16.707	22.59922	18.26434	30242	117	183	16	8.743169	2.088053	134|135|148|152|1128|1813|1814|2554|2558|2903|3356|4985|4986|4988|6531|6532	ADORA1|ADORA2A|ADRA1A|ADRA2C|CHRM1|DRD2|DRD3|GABRA1|GABRA5|GRIN2A|HTR2A|OPRD1|OPRK1|OPRM1|SLC6A3|SLC6A4	-14.6046	0	1	0	0	-34.1199	42.56615334
1	-16.4076	GO:0098936	M1	1	0	GO Cellular Components	20	intrinsic component of postsynaptic membrane	-16.4076	21.67303	17.85225	30242	122	183	16	8.743169	2.088053	134|135|148|152|1128|1813|1814|2554|2558|2903|3356|4985|4986|4988|6531|6532	ADORA1|ADORA2A|ADRA1A|ADRA2C|CHRM1|DRD2|DRD3|GABRA1|GABRA5|GRIN2A|HTR2A|OPRD1|OPRK1|OPRM1|SLC6A3|SLC6A4	-14.3331	0	1	0	0	-34.1199	42.56615334
1	-15.3627	GO:0030424	M1	1	0	GO Cellular Components	20	axon	-15.3627	7.107821	12.2923	30242	651	183	28	15.30055	2.661143	134|135|152|351|760|1128|1268|1813|2566|2902|3351|3356|3643|4914|4915|4985|4986|4988|5024|5582|5979|6326|6335|6336|6531|10280|23	ADORA1|ADORA2A|ADRA2C|APP|CA2|CHRM1|CNR1|DRD2|GABRG2|GRIN1|HTR1B|HTR2A|INSR|NTRK1|NTRK2|OPRD1|OPR	-13.3147	0	1	0	0	-34.1199	42.56615334

																		621|59341	K1|OPRM1|P2RX3|PRKCG|RET|SCN2A|SCN9A|SCN10A|SLC6A3|SIGMAR1|BACE1|TRPV4							
1	-13.1755	GO:0099572	M1	1	0	GO Cellular Components	20	postsynaptic specialization	-13.1755	9.284092	12.26796	30242	356	183	20	10.92896	2.306387	134|135|152|775|1128|1813|1814|2554|2558|2891|2902|2903|2904|2915|4842|4915|4985|5142|5582|10280	ADORA1|ADORA2A|ADRA2C|CACNA1C|CHRM1|DRD2|DRD3|GABRA1|GABRA5|GRIA2|GRIN1|GRIN2A|GRIN2B|GRM5|NOS1|NTRK2|OPRD1|PDE4B|PRKCG|SIGMAR1	-11.1757	0	1	0	0	-34.1199	42.56615334
1	-13.0622	GO:0098984	M1	1	0	GO Cellular Components	20	neuron to neuron synapse	-13.0622	9.155503	12.16307	30242	361	183	20	10.92896	2.306387	134|135|152|775|1128|1813|1814|2891|2902|2903|2904|2915|4842|4915|4985|5024|5142|5582|10280|23621	ADORA1|ADORA2A|ADRA2C|CACNA1C|CHRM1|DRD2|DRD3|GRIA2|GRIN1|GRIN2A|GRIN2B|GRM5|NOS1|NTRK2|OPRD1|P2RX3|PDE4B|PRKCG|SIGMAR1|BACE1	-11.0847	0	1	0	0	-34.1199	42.56615334
1	-11.6824	GO:0014069	M1	1	0	GO Cellular Components	20	postsynaptic density	-11.6824	9.014009	11.42143	30242	330	183	18	9.836066	2.201413	134|135|152|775|1128|1813|1814|2891|2902|2903|2904|2915|4842|4915|4985|5142|5582|10280	ADORA1|ADORA2A|ADRA2C|CACNA1C|CHRM1|DRD2|DRD3|GRIA2|GRIN1|GRIN2A|GRIN2B|GRM5|NOS1|NTRK2|OPRD1|PDE4B|PRKCG|SIGMAR1	-9.76562	0	1	0	0	-34.1199	42.56615334
1	-11.5731	GO:0032279	M1	1	0	GO Cellular Components	20	asymmetric synapse	-11.5731	8.879471	11.31539	30242	335	183	18	9.836066	2.201413	134|135|152|775|1128|1813|1814|2891|2902|2903|2904|2915|4842|4915|4985|5142|5582|10280	ADORA1|ADORA2A|ADRA2C|CACNA1C|CHRM1|DRD2|DRD3|GRIA2|GRIN1|GRIN2A|GRIN2B|GRM5|NOS1|NTRK2|OPRD1|PDE4B|PRKCG|SIGMAR1	-9.67475	0	1	0	0	-34.1199	42.56615334
1	-11.1312	GO:0099634	M1	1	0	GO Cellular Components	20	postsynaptic specialization membrane	-11.1312	16.94942	13.48685	30242	117	183	12	6.557377	1.829835	152|775|1128|1814|2554|2558|2891|2903|2904|2915|4985|10280	ADRA2C|CACNA1C|CHRM1|DRD3|GABRA1|GABRA5|GRIA2|GRIN2A|GRIN2B|GRM5|OPRD1|SIGMAR1	-9.28407	0	1	0	0	-34.1199	42.56615334
1	-9.7622	GO:0098839	M1	1	0	GO Cellular Components	20	postsynaptic density membrane	-9.7622	18.56818	12.95069	30242	89	183	10	5.464481	1.680144	152|775|1128|1814|2891|2903|2904|2915|4985|10280	ADRA2C|CACNA1C|CHRM1|DRD3|GRIA2|GRIN2A|GRIN2B|GRM5|OPRD1|SIGMAR1	-7.97503	0	1	0	0	-34.1199	42.56615334
1	-9.63689	GO:0043679	M1	1	0	GO Cellular Components	20	axon terminus	-9.63689	14.90021	12.00356	30242	122	183	11	6.010929	1.757049	134|152|1128|1813|2902|3351|4915|4985|4986|5024|5582	ADORA1|ADRA2C|CHRM1|DRD2|GRIN1|HTR1B|NTRK2|OPRD1|OPRK1|P2RX3|PRKCG	-7.86352	0	1	0	0	-34.1199	42.56615334

1	-9.40976	GO:0150034	M1	1	0	GO Cellular Components	20	distal axon	-9.40976	8.431471	9.990814	30242	294	183	15	8.196721	2.027793	134|152|351|1128|1268|1813|2902|3351|4915|4985|4986|5024|5582|10280|59341	ADORA1|ADRA2C|APP|CHRM1|CNR1|DRD2|GRIN1|HTR1B|NTRK2|OPRD1|OPRK1|P2RX3|PRKCG|SIGMAR1|TRPV4	-7.66272	0	1	0	0	-34.1199	42.56615334
1	-8.96066	GO:0044306	M1	1	0	GO Cellular Components	20	neuron projection terminus	-8.96066	12.89238	11.04394	30242	141	183	11	6.010929	1.757049	134|152|1128|1813|2902|3351|4915|4985|4986|5024|5582	ADORA1|ADRA2C|CHRM1|DRD2|GRIN1|HTR1B|NTRK2|OPRD1|OPRK1|P2RX3|PRKCG	-7.26192	0	1	0	0	-34.1199	42.56615334
1	-6.78348	GO:0098978	M1	1	0	GO Cellular Components	20	glutamatergic synapse	-6.78348	6.356032	7.726329	30242	338	183	13	7.103825	1.898976	135|148|152|1128|1268|1813|1814|1815|2903|3356|5970|6326|6336	ADORA2A|ADRA1A|ADRA2C|CHRM1|CNR1|DRD2|DRD3|DRD4|GRIN2A|HTR2A|RELA|SCN2A|SCN10A	-5.18619	0	1	0	0	-34.1199	42.56615334
1	-6.43006	GO:0099060	M1	1	0	GO Cellular Components	20	integral component of postsynaptic specialization membrane	-6.43006	15.42397	9.758561	30242	75	183	7	3.825137	1.417845	152|1128|1814|2554|2558|2903|4985	ADRA2C|CHRM1|DRD3|GABRA1|GABRA5|GRIN2A|OPRD1	-4.85911	0	1	0	0	-34.1199	42.56615334
1	-6.31249	GO:0098948	M1	1	0	GO Cellular Components	20	intrinsic component of postsynaptic specialization membrane	-6.31249	14.83074	9.542994	30242	78	183	7	3.825137	1.417845	152|1128|1814|2554|2558|2903|4985	ADRA2C|CHRM1|DRD3|GABRA1|GABRA5|GRIN2A|OPRD1	-4.75825	0	1	0	0	-34.1199	42.56615334
1	-4.97262	GO:0099061	M1	1	0	GO Cellular Components	20	integral component of postsynaptic density membrane	-4.97262	17.21425	8.771899	30242	48	183	5	2.73224	1.205087	152|1128|1814|2903|4985	ADRA2C|CHRM1|DRD3|GRIN2A|OPRD1	-3.47224	0	1	0	0	-34.1199	42.56615334
1	-4.84168	GO:0099146	M1	1	0	GO Cellular Components	20	intrinsic component of postsynaptic density membrane	-4.84168	16.20165	8.47761	30242	51	183	5	2.73224	1.205087	152|1128|1814|2903|4985	ADRA2C|CHRM1|DRD3|GRIN2A|OPRD1	-3.34848	0	1	0	0	-34.1199	42.56615334
1	-3.45874	GO:0043195	M1	1	0	GO Cellular Components	20	terminal bouton	-3.45874	12.01868	6.381713	30242	55	183	4	2.185792	1.080886	134|2902|4915|5024	ADORA1|GRIN1|NTRK2|P2RX3	-2.13446	0	1	0	0	-34.1199	42.56615334
1	-31.6576	GO:0030425	M1	1	0	GO Cellular Components	20	dendrite	-31.6576	11.12057	20.17548	30242	639	183	43	23.49727	3.134163	100|134|135|351|775|1128|1129|1131|1132|1133|1728|1812|1813|1815|2554|2558|2566|2891|2902|2903|3350|3351|3356|3357|3358|3362|3363|3643|4842|4914|4915|4985|4986|4988|5024|5142|5582|5979|7442|11255|23621|59340|729230	ADA|ADORA1|ADORA2A|APP|CACNA1C|CHRM1|CHRM2|CHRM3|CHRM4|CHRM5|NQO1|DRD1|DRD2|DRD4|GABRA1|GABRA5|GABRG2|GRIA2|GRIN1|GRIN2A|HTR1A|HTR1B|HTR2A|HTR2B|HTR2C|HTR6|HTR7|INSR|NOS1|NTRK1|NTRK2|OPRD1|OPRK1|OPRM1|P2RX3|PDE4B|PRKCG|RET|TRPV1|HRH3|BACE1|HRH4|CCR2	-28.7998	0	2	1	1	-31.6576	11.1205692
1	-31.6012	GO:0097447	M1	1	0	GO Cellular Components	20	dendritic tree	-31.6012	11.08587	20.13843	30242	641	183	43	23.49727	3.134163	100|134|135|351|775|1128|1129|1131|1132|1133|1728|1812|1813|1815|	ADA|ADORA1|ADORA2A|APP|CACNA1C|CHRM1|CHRM2|CHR	-28.7998	0	2	0	0	-31.6576	11.1205692

																		2554|2558|2566|2891|2902|2903|3350|3351|3356|3357|3358|3362|3363|3643|4842|4914|4915|4985|4986|4988|5024|5142|5582|5979|7442|11255|23621|59340|729230	M3|CHRM4|CHRM5|NQO1|DRD1|DRD2|DRD4|GABRA1|GABRA5|GABRG2|GRIA2|GRIN1|GRIN2A|HTR1A|HTR1B|HTR2A|HTR2B|HTR2C|HTR6|HTR7|INSR|NOS1|NTRK1|NTRK2|OPRD1|OPRK1|OPRM1|P2RX3|PDE4B|PRKCG|RET|TRPV1|HRH3|BACE1|HRH4|CCR2							
1	-14.1166	GO:0043025	M1	1	0	GO Cellular Components	20	neuronal cell body	-14.1166	7.838269	12.10365	30242	506	183	24	13.11475	2.495327	100|134|135|152|351|775|836|1723|1728|1813|2558|2891|3356|3643|4914|4986|4988|5024|5979|6530|6531|7442|23621|729230	ADA|ADORA1|ADORA2A|ADRA2C|APP|CACNA1C|CASP3|DHODH|NQO1|DRD2|GABRA5|GRIA2|HTR2A|INSR|NTRK1|OPRK1|OPRM1|P2RX3|RET|SLC6A2|SLC6A3|TRPV1|BACE1|CCR2	-12.0933	0	2	0	0	-31.6576	11.1205692
1	-12.876	GO:0044297	M1	1	0	GO Cellular Components	20	cell body	-12.876	6.873768	11.11524	30242	577	183	24	13.11475	2.495327	100|134|135|152|351|775|836|1723|1728|1813|2558|2891|3356|3643|4914|4986|4988|5024|5979|6530|6531|7442|23621|729230	ADA|ADORA1|ADORA2A|ADRA2C|APP|CACNA1C|CASP3|DHODH|NQO1|DRD2|GABRA5|GRIA2|HTR2A|INSR|NTRK1|OPRK1|OPRM1|P2RX3|RET|SLC6A2|SLC6A3|TRPV1|BACE1|CCR2	-10.9197	0	2	0	0	-31.6576	11.1205692
1	-26.7555	GO:0045121	M1	1	0	GO Cellular Components	20	membrane raft	-26.7555	15.20167	20.45591	30242	337	183	31	16.93989	2.772848	147|148|351|836|1268|1910|1956|2149|3356|3383|3551|3643|3778|4842|4846|4985|4988|5024|5743|5979|6331|6401|6513|6530|6531|6532|7046|7124|7132|9429|23621	ADRA1B|ADRA1A|APP|CASP3|CNR1|EDNRB|EGFR|F2R|HTR2A|ICAM1|IKBKB|INSR|KCNMA1|NOS1|NOS3|OPRD1|OPRM1|P2RX3|PTGS2|RET|SCN5A|SELE|SLC2A1|SLC6A2|SLC6A3|SLC6A4|TGFBR1|TNF|TNFRSF1A|ABCG2|BACE1	-24.2551	0	3	1	1	-26.7555	23.33037608
1	-26.7555	GO:0098857	M1	1	0	GO Cellular Components	20	membrane microdomain	-26.7555	15.20167	20.45591	30242	337	183	31	16.93989	2.772848	147|148|351|836|1268|1910|1956|2149|3356|3383|3551|3643|3778|4842|4846|4985|4988|5024|5743|5979|6331|6401|6513|6530|6531|6532|7046|7124|7132|9429|23621	ADRA1B|ADRA1A|APP|CASP3|CNR1|EDNRB|EGFR|F2R|HTR2A|ICAM1|IKBKB|INSR|KCNMA1|NOS1|NOS3|OPRD1|OPRM1|P2RX3|PTGS2|RET|SCN5A|SELE|SLC2A1|SLC6A2|SLC6A3|SLC6A4|	-24.2551	0	3	0	0	-26.7555	23.33037608

																			TGFBR1|TNF|TNFRSF1A|ABCG2|BACE1							
1	-12.8238	GO:0005901	M1	1	0	GO Cellular Components	20	caveola	-12.8238	23.33038	16.08599	30242	85	183	12	6.557377	1.829835	147|148|2149|3356|3643|3778|4846|5743|6331|6401|6513|6531	ADRA1B|ADRA1A|F2R|HTR2A|INSR|KCNMA1|NOS3|PTGS2|SCN5A|SELE|SLC2A1|SLC6A3	-10.8877	0	3	0	0	-26.7555	23.33037608
1	-11.1312	GO:0044853	M1	1	0	GO Cellular Components	20	plasma membrane raft	-11.1312	16.94942	13.48685	30242	117	183	12	6.557377	1.829835	147|148|2149|3356|3643|3778|4846|5743|6331|6401|6513|6531	ADRA1B|ADRA1A|F2R|HTR2A|INSR|KCNMA1|NOS3|PTGS2|SCN5A|SELE|SLC2A1|SLC6A3	-9.28407	0	3	0	0	-26.7555	23.33037608
1	-21.9662	GO:0043235	M1	1	0	GO Cellular Components	20	receptor complex	-21.9662	9.996632	16.28812	30242	529	183	32	17.48634	2.807934	150|154|351|1956|2050|2263|2554|2558|2566|2891|2902|2903|2904|3351|3356|3357|3358|3551|3569|3643|3683|4914|4915|5024|5159|5468|5979|7046|7099|7132|7421|10203	ADRA2A|ADRB2|APP|EGFR|EPHB4|FGFR2|GABRA1|GABRA5|GABRG2|GRIA2|GRIN1|GRIN2A|GRIN2B|HTR1B|HTR2A|HTR2B|HTR2C|IKBKB|IL6|INSR|ITGAL|NTRK1|NTRK2|P2RX3|PDGFRB|PPARG|RET|TGFBR1|TLR4|TNFRSF1A|VDR|CALCRL	-19.6876	0	4	1	1	-21.9662	9.996632475
1	-11.1462	GO:0043197	M1	1	0	GO Cellular Components	20	dendritic spine	-11.1462	12.6426	12.32607	30242	183	183	14	7.650273	1.964856	134|351|1812|1813|2891|2902|2903|4842|4915|4985|4988|5024|5142|7442	ADORA1|APP|DRD1|DRD2|GRIA2|GRIN1|GRIN2A|NOS1|NTRK2|OPRD1|OPRM1|P2RX3|PDE4B|TRPV1	-9.28407	0	5	1	1	-11.1462	12.64259906
1	-11.1141	GO:0044309	M1	1	0	GO Cellular Components	20	neuron spine	-11.1141	12.57389	12.28696	30242	184	183	14	7.650273	1.964856	134|351|1812|1813|2891|2902|2903|4842|4915|4985|4988|5024|5142|7442	ADORA1|APP|DRD1|DRD2|GRIA2|GRIN1|GRIN2A|NOS1|NTRK2|OPRD1|OPRM1|P2RX3|PDE4B|TRPV1	-9.28272	0	5	0	0	-11.1462	12.64259906
1	-5.27078	GO:0008021	M1	1	0	GO Cellular Components	20	synaptic vesicle	-5.27078	7.150536	6.945034	30242	208	183	9	4.918033	1.598524	351|1813|2902|2903|4985|4986|5142|23621|51305	APP|DRD2|GRIN1|GRIN2A|OPRD1|OPRK1|PDE4B|BACE1|KCNK9	-3.75569	0	5	0	0	-11.1462	12.64259906
1	-4.98059	GO:0070382	M1	1	0	GO Cellular Components	20	exocytic vesicle	-4.98059	6.581024	6.570949	30242	226	183	9	4.918033	1.598524	351|1813|2902|2903|4985|4986|5142|23621|51305	APP|DRD2|GRIN1|GRIN2A|OPRD1|OPRK1|PDE4B|BACE1|KCNK9	-3.47292	0	5	0	0	-11.1462	12.64259906
1	-3.647	GO:0030133	M1	1	0	GO Cellular Components	20	transport vesicle	-3.647	4.001376	4.791952	30242	413	183	10	5.464481	1.680144	351|762|1813|2902|2903|4985|4986|5142|23621|51305	APP|CA4|DRD2|GRIN1|GRIN2A|OPRD1|OPRK1|PDE4B|BACE1|KCNK9	-2.2834	0	5	0	0	-11.1462	12.64259906
1	-3.38181	GO:0043204	M1	1	0	GO Cellular Components	20	perikaryon	-3.38181	6.275576	5.187606	30242	158	183	6	3.278689	1.316393	351|775|1813|4986|4988|729230	APP|CACNA1C|DRD2|OPRK1|OPRM1|CCR2	-2.06233	0	5	0	0	-11.1462	12.64259906
1	-10.6902	GO:0034702	M1	1	0	GO Cellular Components	20	ion channel complex	-10.6902	9.376274	11.02658	30242	282	183	16	8.743169	2.088053	775|1080|2554|2558|2566|2891|2902|2903|290	CACNA1C|CFTR|GABRA1|GABRA5|GAB	-8.87406	0	6	1	1	-10.6902	55.0856102

																		4|3757|3778|5142|6326|6331|6335|6336	RG2|GRIA2|GRIN1|GRIN2A|GRIN2B|KCNH2|KCNMA1|PDE4B|SCN2A|SCN5A|SCN9A|SCN10A							
1	-9.59104	GO:0098797	M1	1	0	GO Cellular Components	20	plasma membrane protein complex	-9.59104	5.261433	8.841887	30242	691	183	22	12.02186	2.404073	775|836|1956|2891|2902|2903|2904|3551|3569|3643|3683|3757|3778|5142|5979|6326|6331|6335|6336|6531|7046|10203	CACNA1C|CASP3|EGFR|GRIA2|GRIN1|GRIN2A|GRIN2B|IKBKB|IL6|INSR|ITGAL|KCNH2|KCNMA1|PDE4B|RET|SCN2A|SCN5A|SCN9A|SCN10A|SLC6A3|TGFBR1|CALCRL	-7.83103	0	6	0	0	-10.6902	55.0856102
1	-9.02549	GO:1902495	M1	1	0	GO Cellular Components	20	transmembrane transporter complex	-9.02549	7.224342	9.347794	30242	366	183	16	8.743169	2.088053	775|1080|2554|2558|2566|2891|2902|2903|2904|3757|3778|5142|6326|6331|6335|6336	CACNA1C|CFTR|GABRA1|GABRA5|GABRG2|GRIA2|GRIN1|GRIN2A|GRIN2B|KCNH2|KCNMA1|PDE4B|SCN2A|SCN5A|SCN9A|SCN10A	-7.31517	0	6	0	0	-10.6902	55.0856102
1	-8.77472	GO:1990351	M1	1	0	GO Cellular Components	20	transporter complex	-8.77472	6.939919	9.103895	30242	381	183	16	8.743169	2.088053	775|1080|2554|2558|2566|2891|2902|2903|2904|3757|3778|5142|6326|6331|6335|6336	CACNA1C|CFTR|GABRA1|GABRA5|GABRG2|GRIA2|GRIN1|GRIN2A|GRIN2B|KCNH2|KCNMA1|PDE4B|SCN2A|SCN5A|SCN9A|SCN10A	-7.10898	0	6	0	0	-10.6902	55.0856102
1	-8.17982	GO:0034703	M1	1	0	GO Cellular Components	20	cation channel complex	-8.17982	9.443247	9.579953	30242	210	183	12	6.557377	1.829835	775|2891|2902|2903|2904|3757|3778|5142|6326|6331|6335|6336	CACNA1C|GRIA2|GRIN1|GRIN2A|GRIN2B|KCNH2|KCNMA1|PDE4B|SCN2A|SCN5A|SCN9A|SCN10A	-6.52455	0	6	0	0	-10.6902	55.0856102
1	-4.74896	GO:0017146	M1	1	0	GO Cellular Components	20	NMDA selective glutamate receptor complex	-4.74896	55.08561	12.6619	30242	9	183	3	1.639344	0.938686	2902|2903|2904	GRIN1|GRIN2A|GRIN2B	-3.26978	0	6	0	0	-10.6902	55.0856102
1	-4.72551	GO:0098802	M1	1	0	GO Cellular Components	20	plasma membrane signaling receptor complex	-4.72551	5.40055	6.036805	30242	306	183	10	5.464481	1.680144	2891|2902|2903|2904|3551|3569|3643|3683|7046|10203	GRIA2|GRIN1|GRIN2A|GRIN2B|IKBKB|IL6|INSR|ITGAL|TGFBR1|CALCRL	-3.25317	0	6	0	0	-10.6902	55.0856102
1	-3.99998	GO:0008328	M1	1	0	GO Cellular Components	20	ionotropic glutamate receptor complex	-3.99998	16.52568	7.666537	30242	40	183	4	2.185792	1.080886	2891|2902|2903|2904	GRIA2|GRIN1|GRIN2A|GRIN2B	-2.58477	0	6	0	0	-10.6902	55.0856102
1	-3.91602	GO:0098878	M1	1	0	GO Cellular Components	20	neurotransmitter receptor complex	-3.91602	15.73875	7.457926	30242	42	183	4	2.185792	1.080886	2891|2902|2903|2904	GRIA2|GRIN1|GRIN2A|GRIN2B	-2.51256	0	6	0	0	-10.6902	55.0856102
1	-3.65322	GO:0060076	M1	1	0	GO Cellular Components	20	excitatory synapse	-3.65322	13.49035	6.82741	30242	49	183	4	2.185792	1.080886	2263|2891|2902|5142	FGFR2|GRIA2|GRIN1|PDE4B	-2.2834	0	6	0	0	-10.6902	55.0856102
1	-9.86196	GO:0032589	M1	1	0	GO Cellular Components	20	neuron projection membrane	-9.86196	23.98889	14.13809	30242	62	183	9	4.918033	1.598524	134|135|2554|2558|2566|3643|4985|4988|7442	ADORA1|ADORA2A|GABRA1|GABRA5|GABRG2|INSR|OPRD1|OPRM1|TRPV1	-8.06056	0	7	1	1	-9.86196	26.90227475
1	-9.36076	GO:0031253	M1	1	0	GO Cellular Components	20	cell projection membrane	-9.36076	7.619911	9.677295	30242	347	183	16	8.743169	2.088053	134|135|762|1812|1813|1956|2554|2558|2566|3	ADORA1|ADORA2A|CA4|DRD1|DRD2|EG	-7.62631	0	7	0	0	-9.86196	26.90227475

																		643|4985|4988|6523|7442|9429|59341	FR|GABRA1|GABRA5|GABRG2|INSR|OPRD1|OPRM1|SLC5A1|TRPV1|ABCG2|TRPV4							
1	-8.14883	GO:0032590	M1	1	0	GO Cellular Components	20	dendrite membrane	-8.14883	26.90227	13.2621	30242	43	183	7	3.825137	1.417845	2554|2558|2566|3643|4985|4988|7442	GABRA1|GABRA5|GABRG2|INSR|OPRD1|OPRM1|TRPV1	-6.50378	0	7	0	0	-9.86196	26.90227475
1	-7.81915	GO:0031256	M1	1	0	GO Cellular Components	20	leading edge membrane	-7.81915	10.04323	9.521331	30242	181	183	11	6.010929	1.757049	134|135|1956|2554|2558|2566|3643|4985|4988|7442|59341	ADORA1|ADORA2A|EGFR|GABRA1|GABRA5|GABRG2|INSR|OPRD1|OPRM1|TRPV1|TRPV4	-6.20338	0	7	0	0	-9.86196	26.90227475
1	-6.68154	GO:0098982	M1	1	0	GO Cellular Components	20	GABA-ergic synapse	-6.68154	16.76519	10.22941	30242	69	183	7	3.825137	1.417845	148|1268|1813|1814|2554|2558|2566	ADRA1A|CNR1|DRD2|DRD3|GABRA1|GABRA5|GABRG2	-5.09322	0	7	0	0	-9.86196	26.90227475
1	-6.47078	GO:0031252	M1	1	0	GO Cellular Components	20	cell leading edge	-6.47078	5.469493	7.223011	30242	423	183	14	7.650273	1.964856	134|135|351|1956|2554|2558|2566|3643|4985|4988|5290|7099|7442|59341	ADORA1|ADORA2A|APP|EGFR|GABRA1|GABRA5|GABRG2|INSR|OPRD1|OPRM1|PIK3CA|TLR4|TRPV1|TRPV4	-4.89123	0	7	0	0	-9.86196	26.90227475
1	-3.70629	GO:1902711	M1	1	0	GO Cellular Components	20	GABA-A receptor complex	-3.70629	26.09318	8.536899	30242	19	183	3	1.639344	0.938686	2554|2558|2566	GABRA1|GABRA5|GABRG2	-2.31986	0	7	0	0	-9.86196	26.90227475
1	-3.63764	GO:1902710	M1	1	0	GO Cellular Components	20	GABA receptor complex	-3.63764	24.78852	8.303425	30242	20	183	3	1.639344	0.938686	2554|2558|2566	GABRA1|GABRA5|GABRG2	-2.2834	0	7	0	0	-9.86196	26.90227475
1	-3.61905	GO:0034707	M1	1	0	GO Cellular Components	20	chloride channel complex	-3.61905	13.22055	6.74786	30242	50	183	4	2.185792	1.080886	1080|2554|2558|2566	CFTR|GABRA1|GABRA5|GABRG2	-2.27502	0	7	0	0	-9.86196	26.90227475
1	-9.03513	GO:0009897	M1	1	0	GO Cellular Components	20	external side of plasma membrane	-9.03513	6.673079	9.146082	30242	421	183	17	9.289617	2.145862	100|762|1230|1636|2147|2891|2936|3383|3577|3643|5243|6401|7099|7124|7442|9429|729230	ADA|CA4|CCR1|ACE|F2|GRIA2|GSR|ICAM1|CXCR1|INSR|ABCB1|SELE|TLR4|TNF|TRPV1|ABCG2|CCR2	-7.31517	0	8	1	1	-9.03513	6.67307867
1	-7.87058	GO:0098552	M1	1	0	GO Cellular Components	20	side of membrane	-7.87058	4.921442	7.811191	30242	638	183	19	10.38251	2.254873	52|100|762|1230|1636|2147|2891|2936|3383|3551|3577|3643|5243|6401|7099|7124|7442|9429|729230	ACP1|ADA|CA4|CCR1|ACE|F2|GRIA2|GSR|ICAM1|IKBKB|CXCR1|INSR|ABCB1|SELE|TLR4|TNF|TRPV1|ABCG2|CCR2	-6.24527	0	8	0	0	-9.03513	6.67307867
1	-8.88688	GO:0098665	M1	1	0	GO Cellular Components	20	serotonin receptor complex	-8.88688	165.2568	25.63382	30242	4	183	4	2.185792	1.080886	3351|3356|3357|3358	HTR1B|HTR2A|HTR2B|HTR2C	-7.21042	0	9	1	1	-8.88688	165.2568306
1	-8.88688	GO:0098666	M1	1	0	GO Cellular Components	20	G protein-coupled serotonin receptor complex	-8.88688	165.2568	25.63382	30242	4	183	4	2.185792	1.080886	3351|3356|3357|3358	HTR1B|HTR2A|HTR2B|HTR2C	-7.21042	0	9	0	0	-8.88688	165.2568306
1	-7.93116	GO:0045177	M1	1	0	GO Cellular Components	20	apical part of cell	-7.93116	6.050593	8.298028	30242	437	183	16	8.743169	2.088053	154|351|760|762|1080|1814|1956|2335|3778|4363|5159|5243|6513|6523|9429|59341	ADRB2|APP|CA2|CA4|CFTR|DRD3|EGFR|FN1|KCNMA1|ABCC1|PDGFRB|ABCB1|SLC2A1|SLC5A1|ABCG2|TRPV4	-6.29609	0	10	1	1	-7.93116	6.05059334
1	-6.34992	GO:0016324	M1	1	0	GO Cellular Components	20	apical plasma membrane	-6.34992	5.822056	7.271808	30242	369	183	13	7.103825	1.898976	154|762|1080|1956|2335|3778|4363|5159|5243|	ADRB2|CA4|CFTR|EGFR|FN1|KCNMA1|A	-4.7874	0	10	0	0	-7.93116	6.05059334

																		6513|6523|9429|59341	BCC1|PDGFRB|ABCB1|SLC2A1|SLC5A1|ABCG2|TRPV4							
1	-4.03348	GO:0016323	M1	1	0	GO Cellular Components	20	basolateral plasma membrane	-4.03348	5.674054	5.588333	30242	233	183	8	4.371585	1.511428	134|150|760|762|1131|1956|4363|6513	ADORA1|ADRA2A|CA2|CA4|CHRM3|EGFR|ABCC1|SLC2A1	-2.61229	0	10	0	0	-7.93116	6.05059334
1	-3.71824	GO:0009925	M1	1	0	GO Cellular Components	20	basal plasma membrane	-3.71824	5.104458	5.176122	30242	259	183	8	4.371585	1.511428	134|150|760|762|1131|1956|4363|6513	ADORA1|ADRA2A|CA2|CA4|CHRM3|EGFR|ABCC1|SLC2A1	-2.3262	0	10	0	0	-7.93116	6.05059334
1	-3.51109	GO:0045178	M1	1	0	GO Cellular Components	20	basal part of cell	-3.51109	4.755592	4.90837	30242	278	183	8	4.371585	1.511428	134|150|760|762|1131|1956|4363|6513	ADORA1|ADRA2A|CA2|CA4|CHRM3|EGFR|ABCC1|SLC2A1	-2.18195	0	10	0	0	-7.93116	6.05059334
1	-6.79682	GO:0098691	M1	1	0	GO Cellular Components	20	dopaminergic synapse	-6.79682	73.44748	16.96056	30242	9	183	4	2.185792	1.080886	148|150|1813|6531	ADRA1A|ADRA2A|DRD2|SLC6A3	-5.19039	0	11	1	1	-6.79682	73.44748027
1	-6.20212	GO:0048471	M1	1	0	GO Cellular Components	20	perinuclear region of cytoplasm	-6.20212	4.030654	6.5037	30242	738	183	18	9.836066	2.201413	328|351|762|1453|1956|3757|4842|4843|4915|5138|5142|5290|5582|6331|6401|6523|7099|729230	APEX1|APP|CA4|CSNK1D|EGFR|KCNH2|NOS1|NOS2|NTRK2|PDE2A|PDE4B|PIK3CA|PRKCG|SCN5A|SELE|SLC5A1|TLR4|CCR2	-4.65599	0	12	1	1	-6.20212	4.030654405
1	-5.94566	GO:0005769	M1	1	0	GO Cellular Components	20	early endosome	-5.94566	5.357453	6.85385	30242	401	183	13	7.103825	1.898976	153|154|351|427|1080|1956|2149|4914|4915|5979|6523|7099|23621	ADRB1|ADRB2|APP|ASAH1|CFTR|EGFR|F2R|NTRK1|NTRK2|RET|SLC5A1|TLR4|BACE1	-4.4075	0	13	1	1	-5.94566	5.357453361
1	-2.8764	GO:0010008	M1	1	0	GO Cellular Components	20	endosome membrane	-2.8764	3.178016	3.90895	30242	520	183	10	5.464481	1.680144	154|1080|1956|3643|4914|4915|5979|6532|7099|23621	ADRB2|CFTR|EGFR|INSR|NTRK1|NTRK2|RET|SLC6A4|TLR4|BACE1	-1.61071	0	13	0	0	-5.94566	5.357453361
1	-2.15797	GO:0055037	M1	1	0	GO Cellular Components	20	recycling endosome	-2.15797	4.215735	3.524126	30242	196	183	5	2.73224	1.205087	351|1080|4914|7124|23621	APP|CFTR|NTRK1|TNF|BACE1	-0.97286	0	13	0	0	-5.94566	5.357453361
1	-2.1195	GO:0005770	M1	1	0	GO Cellular Components	20	late endosome	-2.1195	3.516103	3.312214	30242	282	183	6	3.278689	1.316393	1956|2149|2904|3643|4914|23621	EGFR|F2R|GRIN2B|INSR|NTRK1|BACE1	-0.93789	0	13	0	0	-5.94566	5.357453361
1	-5.67902	GO:0042383	M1	1	0	GO Cellular Components	20	sarcolemma	-5.67902	9.580106	7.882363	30242	138	183	8	4.371585	1.511428	52|148|775|4842|4988|6326|6331|6513	ACP1|ADRA1A|CACNA1C|NOS1|OPRM1|SCN2A|SCN5A|SLC2A1	-4.14868	0	14	1	1	-5.67902	38.88396014
1	-5.53708	GO:0001518	M1	1	0	GO Cellular Components	20	voltage-gated sodium channel complex	-5.53708	38.88396	12.19082	30242	17	183	4	2.185792	1.080886	6326|6331|6335|6336	SCN2A|SCN5A|SCN9A|SCN10A	-4.01443	0	14	0	0	-5.67902	38.88396014
1	-4.75751	GO:0034706	M1	1	0	GO Cellular Components	20	sodium channel complex	-4.75751	25.42413	9.721283	30242	26	183	4	2.185792	1.080886	6326|6331|6335|6336	SCN2A|SCN5A|SCN9A|SCN10A	-3.27138	0	14	0	0	-5.67902	38.88396014
1	-3.64474	GO:0005911	M1	1	0	GO Cellular Components	20	cell-cell junction	-3.64474	3.679808	4.685701	30242	494	183	11	6.010929	1.757049	351|595|5290|5582|6326|6331|6513|6523|7046|59341|225689	APP|CCND1|PIK3CA|PRKCG|SCN2A|SCN5A|SLC2A1|SLC5A1|TGFBR1|TRPV4|MAPK15	-2.2834	0	14	0	0	-5.67902	38.88396014
1	-3.61905	GO:0014704	M1	1	0	GO Cellular Components	20	intercalated disc	-3.61905	13.22055	6.74786	30242	50	183	4	2.185792	1.080886	5290|6326|6331|6513	PIK3CA|SCN2A|SCN5A|SLC2A1	-2.27502	0	14	0	0	-5.67902	38.88396014
1	-3.58562	GO:0030315	M1	1	0	GO Cellular Components	20	T-tubule	-3.58562	12.96132	6.670552	30242	51	183	4	2.185792	1.080886	148|775|6326|6331	ADRA1A|CACNA1C|SCN2A|SCN5A	-2.24662	0	14	0	0	-5.67902	38.88396014
1	-3.01392	GO:0044291	M1	1	0	GO Cellular	20	cell-cell contact zone	-3.01392	9.180935	5.422729	30242	72	183	4	2.185792	1.080886	5290|6326|6331|6513	PIK3CA|SCN2A|SCN5	-1.72246	0	14	0	0	-5.67902	38.88396014

						Components													A|SLC2A1							
1	-2.92284	GO:0030018	M1	1	0	GO Cellular Components	20	Z disc	-2.92284	6.356032	4.775088	30242	130	183	5	2.73224	1.205087	148|775|5142|6331|6513	ADRA1A|CACNA1C|PDE4B|SCN5A|SLC2A1	-1.64431	0	14	0	0	-5.67902	38.88396014
1	-2.76691	GO:0031674	M1	1	0	GO Cellular Components	20	I band	-2.76691	5.860171	4.513433	30242	141	183	5	2.73224	1.205087	148|775|5142|6331|6513	ADRA1A|CACNA1C|PDE4B|SCN5A|SLC2A1	-1.51369	0	14	0	0	-5.67902	38.88396014
1	-2.73148	GO:0030017	M1	1	0	GO Cellular Components	20	sarcomere	-2.73148	4.699246	4.207324	30242	211	183	6	3.278689	1.316393	148|775|4313|5142|6331|6513	ADRA1A|CACNA1C|MMP2|PDE4B|SCN5A|SLC2A1	-1.48565	0	14	0	0	-5.67902	38.88396014
1	-2.53564	GO:0030016	M1	1	0	GO Cellular Components	20	myofibril	-2.53564	4.292385	3.919338	30242	231	183	6	3.278689	1.316393	148|775|4313|5142|6331|6513	ADRA1A|CACNA1C|MMP2|PDE4B|SCN5A|SLC2A1	-1.30244	0	14	0	0	-5.67902	38.88396014
1	-2.46308	GO:0043292	M1	1	0	GO Cellular Components	20	contractile fiber	-2.46308	4.148707	3.813162	30242	239	183	6	3.278689	1.316393	148|775|4313|5142|6331|6513	ADRA1A|CACNA1C|MMP2|PDE4B|SCN5A|SLC2A1	-1.23764	0	14	0	0	-5.67902	38.88396014
1	-4.62504	GO:0032809	M1	1	0	GO Cellular Components	20	neuronal cell body membrane	-4.62504	23.60812	9.338469	30242	28	183	4	2.185792	1.080886	2558|3643|6530|6531	GABRA5|INSR|SLC6A2|SLC6A3	-3.15943	0	15	1	1	-4.62504	23.60811866
1	-4.44459	GO:0044298	M1	1	0	GO Cellular Components	20	cell body membrane	-4.44459	21.32346	8.83349	30242	31	183	4	2.185792	1.080886	2558|3643|6530|6531	GABRA5|INSR|SLC6A2|SLC6A3	-2.98561	0	15	0	0	-4.62504	23.60811866
1	-4.31869	GO:0031012	M1	1	0	GO Cellular Components	20	extracellular matrix	-4.31869	3.769015	5.207458	30242	570	183	13	7.103825	1.898976	249|1991|2147|2155|2263|2335|3383|4312|4313|4314|4318|4325|7040	ALPL|ELANE|F2|F7|FGFR2|FN1|ICAM1|MMP1|MMP2|MMP3|MMP9|MMP16|TGFB1	-2.86624	0	16	1	1	-4.31869	7.945039933
1	-4.31096	GO:0030312	M1	1	0	GO Cellular Components	20	external encapsulating structure	-4.31096	3.762415	5.199687	30242	571	183	13	7.103825	1.898976	249|1991|2147|2155|2263|2335|3383|4312|4313|4314|4318|4325|7040	ALPL|ELANE|F2|F7|FGFR2|FN1|ICAM1|MMP1|MMP2|MMP3|MMP9|MMP16|TGFB1	-2.86495	0	16	0	0	-4.31869	7.945039933
1	-3.36119	GO:0005796	M1	1	0	GO Cellular Components	20	Golgi lumen	-3.36119	7.94504	5.535675	30242	104	183	5	2.73224	1.205087	351|2147|2155|4325|7040	APP|F2|F7|MMP16|TGFB1	-2.04645	0	16	0	0	-4.31869	7.945039933
1	-3.17151	GO:0005788	M1	1	0	GO Cellular Components	20	endoplasmic reticulum lumen	-3.17151	4.223817	4.473349	30242	313	183	8	4.371585	1.511428	351|2147|2155|2335|3569|4221|5743|23621	APP|F2|F7|FN1|IL6|MEN1|PTGS2|BACE1	-1.86611	0	16	0	0	-4.31869	7.945039933
1	-2.91637	GO:0062023	M1	1	0	GO Cellular Components	20	collagen-containing extracellular matrix	-2.91637	3.491341	4.040636	30242	426	183	9	4.918033	1.598524	1991|2147|2155|2263|2335|3383|4313|4318|7040	ELANE|F2|F7|FGFR2|FN1|ICAM1|MMP2|MMP9|TGFB1	-1.64217	0	16	0	0	-4.31869	7.945039933
1	-2.70066	GO:0072562	M1	1	0	GO Cellular Components	20	blood microparticle	-2.70066	5.659481	4.403479	30242	146	183	5	2.73224	1.205087	2147|2335|5444|6513|7040	F2|FN1|PON1|SLC2A1|TGFB1	-1.45956	0	16	0	0	-4.31869	7.945039933
1	-2.40862	GO:0031983	M1	1	0	GO Cellular Components	20	vesicle lumen	-2.40862	3.537608	3.599899	30242	327	183	7	3.825137	1.417845	100|351|1956|1991|2335|7040|23621	ADA|APP|EGFR|ELANE|FN1|TGFB1|BACE1	-1.19456	0	16	0	0	-4.31869	7.945039933
1	-2.10448	GO:0031093	M1	1	0	GO Cellular Components	20	platelet alpha granule lumen	-2.10448	7.39956	4.091666	30242	67	183	3	1.639344	0.938686	351|2335|7040	APP|FN1|TGFB1	-0.92632	0	16	0	0	-4.31869	7.945039933
1	-4.07255	GO:0031968	M1	1	0	GO Cellular Components	20	organelle outer membrane	-4.07255	5.748064	5.639873	30242	230	183	8	4.371585	1.511428	596|1268|4128|4129|5138|5241|5743|10280	BCL2|CNR1|MAOA|MAOB|PDE2A|PGR|PTGS2|SIGMAR1	-2.63287	0	17	1	1	-4.07255	5.748063673
1	-4.04644	GO:0019867	M1	1	0	GO Cellular Components	20	outer membrane	-4.04644	5.698511	5.605413	30242	232	183	8	4.371585	1.511428	596|1268|4128|4129|5138|5241|5743|10280	BCL2|CNR1|MAOA|MAOB|PDE2A|PGR|PTGS2|SIGMAR1	-2.61917	0	17	0	0	-4.07255	5.748063673
1	-2.7947	GO:0005741	M1	1	0	GO Cellular Components	20	mitochondrial outer membrane	-2.7947	4.836785	4.300832	30242	205	183	6	3.278689	1.316393	596|1268|4128|4129|5138|5241	BCL2|CNR1|MAOA|MAOB|PDE2A|PGR	-1.53736	0	17	0	0	-4.07255	5.748063673

1	-2.73076	GO:0031966	M1	1	0	GO Cellular Components	20	mitochondrial membrane	-2.73076	2.690749	3.62578	30242	737	183	12	6.557377	1.829835	249|596|1268|1543|1723|4128|4129|5138|5241|5498|7298|9429	ALPL|BCL2|CNR1|CYP1A1|DHODH|MAOA|MAOB|PDE2A|PGR|PPOX|TYMS|ABCG2	-1.48565	0	17	0	0	-4.07255	5.748063673
1	-2.49883	GO:0005740	M1	1	0	GO Cellular Components	20	mitochondrial envelope	-2.49883	2.519799	3.37078	30242	787	183	12	6.557377	1.829835	249|596|1268|1543|1723|4128|4129|5138|5241|5498|7298|9429	ALPL|BCL2|CNR1|CYP1A1|DHODH|MAOA|MAOB|PDE2A|PGR|PPOX|TYMS|ABCG2	-1.26952	0	17	0	0	-4.07255	5.748063673
1	-4.0548	GO:0031965	M1	1	0	GO Cellular Components	20	nuclear membrane	-4.0548	5.007783	5.415725	30242	297	183	9	4.918033	1.598524	147|148|595|596|1910|1956|5024|5743|10280	ADRA1B|ADRA1A|CCND1|BCL2|EDNRB|EGFR|P2RX3|PTGS2|SIGMAR1	-2.62138	0	18	1	1	-4.0548	8.127385112
1	-3.84711	GO:0005635	M1	1	0	GO Cellular Components	20	nuclear envelope	-3.84711	3.884242	4.906506	30242	468	183	11	6.010929	1.757049	147|148|351|595|596|1910|1956|3643|5024|5743|10280	ADRA1B|ADRA1A|APP|CCND1|BCL2|EDNRB|EGFR|INSR|P2RX3|PTGS2|SIGMAR1	-2.4494	0	18	0	0	-4.0548	8.127385112
1	-2.21721	GO:0005637	M1	1	0	GO Cellular Components	20	nuclear inner membrane	-2.21721	8.127385	4.347751	30242	61	183	3	1.639344	0.938686	5024|5743|10280	P2RX3|PTGS2|SIGMAR1	-1.02859	0	18	0	0	-4.0548	8.127385112
1	-3.95712	GO:0061695	M1	1	0	GO Cellular Components	20	transferase complex, transferring phosphorus-containing groups	-3.95712	5.531609	5.48789	30242	239	183	8	4.371585	1.511428	595|890|3551|3643|5290|5291|5293|7046	CCND1|CCNA2|IKBKB|INSR|PIK3CA|PIK3CB|PIK3CD|TGFBR1	-2.54783	0	19	1	1	-3.95712	8.607126594
1	-3.5217	GO:1902911	M1	1	0	GO Cellular Components	20	protein kinase complex	-3.5217	8.607127	5.824756	30242	96	183	5	2.73224	1.205087	595|890|3551|3643|7046	CCND1|CCNA2|IKBKB|INSR|TGFBR1	-2.18765	0	19	0	0	-3.95712	8.607126594
1	-2.82347	GO:1902554	M1	1	0	GO Cellular Components	20	serine/threonine protein kinase complex	-2.82347	8.160831	5.035231	30242	81	183	4	2.185792	1.080886	595|890|3551|7046	CCND1|CCNA2|IKBKB|TGFBR1	-1.56198	0	19	0	0	-3.95712	8.607126594
1	-3.65347	GO:0030139	M1	1	0	GO Cellular Components	20	endocytic vesicle	-3.65347	4.426522	4.92811	30242	336	183	9	4.918033	1.598524	154|1080|1129|1813|1814|1956|1991|2891|4846	ADRB2|CFTR|CHRM2|DRD2|DRD3|EGFR|ELANE|GRIA2|NOS3	-2.2834	0	20	1	1	-3.65347	9.180935033
1	-3.01392	GO:0030669	M1	1	0	GO Cellular Components	20	clathrin-coated endocytic vesicle membrane	-3.01392	9.180935	5.422729	30242	72	183	4	2.185792	1.080886	154|1080|1129|1956	ADRB2|CFTR|CHRM2|EGFR	-1.72246	0	20	0	0	-3.65347	9.180935033
1	-2.92812	GO:0030666	M1	1	0	GO Cellular Components	20	endocytic vesicle membrane	-2.92812	5.137518	4.499248	30242	193	183	6	3.278689	1.316393	154|1080|1129|1956|2891|4846	ADRB2|CFTR|CHRM2|EGFR|GRIA2|NOS3	-1.64523	0	20	0	0	-3.65347	9.180935033
1	-2.63793	GO:0045334	M1	1	0	GO Cellular Components	20	clathrin-coated endocytic vesicle	-2.63793	7.264037	4.669393	30242	91	183	4	2.185792	1.080886	154|1080|1129|1956	ADRB2|CFTR|CHRM2|EGFR	-1.40079	0	20	0	0	-3.65347	9.180935033
1	-2.26055	GO:0030665	M1	1	0	GO Cellular Components	20	clathrin-coated vesicle membrane	-2.26055	5.698511	3.955987	30242	116	183	4	2.185792	1.080886	154|1080|1129|1956	ADRB2|CFTR|CHRM2|EGFR	-1.06481	0	20	0	0	-3.65347	9.180935033
1	-3.63764	GO:0044305	M1	1	0	GO Cellular Components	20	calyx of Held	-3.63764	24.78852	8.303425	30242	20	183	3	1.639344	0.938686	134|3351|5582	ADORA1|HTR1B|PRKCG	-2.2834	0	21	1	1	-3.63764	24.78852459
1	-3.19466	GO:0005942	M1	1	0	GO Cellular Components	20	phosphatidylinositol 3-kinase complex	-3.19466	17.70609	6.900587	30242	28	183	3	1.639344	0.938686	5290|5291|5293	PIK3CA|PIK3CB|PIK3CD	-1.88462	0	22	1	1	-3.19466	17.70608899
1	-3.10726	GO:0044304	M1	1	0	GO Cellular Components	20	main axon	-3.10726	9.72099	5.617462	30242	68	183	4	2.185792	1.080886	134|135|351|6326	ADORA1|ADORA2A|APP|SCN2A	-1.80646	0	23	1	1	-3.10726	9.720990035
1	-2.00253	GO:0048786	M1	1	0	GO Cellular Components	20	presynaptic active zone	-2.00253	6.791377	3.865442	30242	73	183	3	1.639344	0.938686	134|135|351	ADORA1|ADORA2A|APP	-0.82781	0	23	0	0	-3.10726	9.720990035
1	-3.00976	GO:0005667	M1	1	0	GO Cellular Components	20	transcription regulator complex	-3.00976	3.601238	4.153098	30242	413	183	9	4.918033	1.598524	328|595|1991|3066|5468|5591|5970|7157|7421	APEX1|CCND1|ELANE|HDAC2|PPARG|PRKDC|RELA|TP53|VDR	-1.72246	0	24	1	1	-3.00976	3.601238439
1	-2.88421	GO:0098685	M1	1	0	GO Cellular	20	Schaffer collateral -	-2.88421	8.474709	5.157432	30242	78	183	4	2.185792	1.080886	153|1128|2915|5024	ADRB1|CHRM1|GRM	-1.61428	0	25	1	1	-2.88421	8.474709262

						Components		CA1 synapse											5|P2RX3							
1	-2.43567	GO:0005782	M1	1	0	GO Cellular Components	20	peroxisomal matrix	-2.43567	9.72099	4.863495	30242	51	183	3	1.639344	0.938686	472|4843|51179	ATM|NOS2|HAO2	-1.21784	0	26	1	1	-2.43567	9.720990035
1	-2.43567	GO:0031907	M1	1	0	GO Cellular Components	20	microbody lumen	-2.43567	9.72099	4.863495	30242	51	183	3	1.639344	0.938686	472|4843|51179	ATM|NOS2|HAO2	-1.21784	0	26	0	0	-2.43567	9.720990035
1	-2.37022	GO:0030863	M1	1	0	GO Cellular Components	20	cortical cytoskeleton	-2.37022	6.120623	4.159515	30242	108	183	4	2.185792	1.080886	4843|6401|6513|59341	NOS2|SELE|SLC2A1|TRPV4	-1.15988	0	27	1	1	-2.37022	6.120623356
1	-2.29811	GO:0000323	M1	1	0	GO Cellular Components	20	lytic vacuole	-2.29811	2.493587	3.185107	30242	729	183	11	6.010929	1.757049	100|154|427|1080|1636|1991|2904|3643|5159|10203|23621	ADA|ADRB2|ASAH1|CFTR|ACE|ELANE|GRIN2B|INSR|PDGFRB|CALCRL|BACE1	-1.09514	0	28	1	1	-2.29811	2.493587293
1	-2.29811	GO:0005764	M1	1	0	GO Cellular Components	20	lysosome	-2.29811	2.493587	3.185107	30242	729	183	11	6.010929	1.757049	100|154|427|1080|1636|1991|2904|3643|5159|10203|23621	ADA|ADRB2|ASAH1|CFTR|ACE|ELANE|GRIN2B|INSR|PDGFRB|CALCRL|BACE1	-1.09514	0	28	0	0	-2.29811	2.493587293
1	-2.27832	GO:0031526	M1	1	0	GO Cellular Components	20	brush border membrane	-2.27832	8.547767	4.489319	30242	58	183	3	1.639344	0.938686	762|6523|9429	CA4|SLC5A1|ABCG2	-1.07898	0	29	1	1	-2.27832	8.5477671
1	-2.21721	GO:0016328	M1	1	0	GO Cellular Components	20	lateral plasma membrane	-2.21721	8.127385	4.347751	30242	61	183	3	1.639344	0.938686	1813|4363|6331	DRD2|ABCC1|SCN5A	-1.02859	0	30	1	1	-2.21721	8.127385112
1	-37.4086	GO:0008227	M1	1	0	GO Molecular Functions	21	G protein-coupled amine receptor activity	-37.4086	74.52759	41.00464	30242	51	183	23	12.56831	2.450458	146|147|148|150|151|152|153|154|1128|1129|1131|1132|1133|1815|3350|3351|3356|3357|3358|3362|3363|11255|59340	ADRA1D|ADRA1B|ADRA1A|ADRA2A|ADRA2B|ADRA2C|ADRB1|ADRB2|CHRM1|CHRM2|CHRM3|CHRM4|CHRM5|DRD4|HTR1A|HTR1B|HTR2A|HTR2B|HTR2C|HTR6|HTR7|HRH3|HRH4	-33.7247	0	1	1	1	-37.4086	165.2568306
1	-37.0619	GO:0030594	M1	1	0	GO Molecular Functions	21	neurotransmitter receptor activity	-37.0619	41.68641	33.50738	30242	111	183	28	15.30055	2.661143	134|153|1128|1129|1131|1132|1133|1812|1813|1814|1815|2554|2558|2566|2891|2902|2903|2904|2915|3350|3351|3356|3357|3358|3362|3363|11255|59340	ADORA1|ADRB1|CHRM1|CHRM2|CHRM3|CHRM4|CHRM5|DRD1|DRD2|DRD3|DRD4|GABRA1|GABRA5|GABRG2|GRIA2|GRIN1|GRIN2A|GRIN2B|GRM5|HTR1A|HTR1B|HTR2A|HTR2B|HTR2C|HTR6|HTR7|HRH3|HRH4	-33.6791	0	1	0	0	-37.4086	165.2568306
1	-24.3015	GO:0004993	M1	1	0	GO Molecular Functions	21	G protein-coupled serotonin receptor activity	-24.3015	72.90743	32.73321	30242	34	183	15	8.196721	2.027793	1128|1129|1131|1132|1133|1815|3350|3351|3356|3357|3358|3362|3363|11255|59340	CHRM1|CHRM2|CHRM3|CHRM4|CHRM5|DRD4|HTR1A|HTR1B|HTR2A|HTR2B|HTR2C|HTR6|HTR7|HRH3|HRH4	-21.2197	0	1	0	0	-37.4086	165.2568306
1	-24.3015	GO:0099589	M1	1	0	GO Molecular Functions	21	serotonin receptor activity	-24.3015	72.90743	32.73321	30242	34	183	15	8.196721	2.027793	1128|1129|1131|1132|1133|1815|3350|3351|3356|3357|3358|3362|3363|11255|59340	CHRM1|CHRM2|CHRM3|CHRM4|CHRM5|DRD4|HTR1A|HTR1B|HTR2A|HTR2B|HTR2C|HTR6|HTR7|HRH3|HRH4	-21.2197	0	1	0	0	-37.4086	165.2568306

1	-21.4495	GO:0098960	M1	1	0	GO Molecular Functions	21	postsynaptic neurotransmitter receptor activity	-21.4495	42.64692	25.61271	30242	62	183	16	8.743169	2.088053	153|1128|1129|1131|1132|1133|1812|1813|1814|1815|2554|2558|2566|2915|11255|59340	ADRB1|CHRM1|CHRM2|CHRM3|CHRM4|CHRM5|DRD1|DRD2|DRD3|DRD4|GABRA1|GABRA5|GABRG2|GRM5|HRH3|HRH4	-18.4646	0	1	0	0	-37.4086	165.2568306
1	-16.1635	GO:0099528	M1	1	0	GO Molecular Functions	21	G protein-coupled neurotransmitter receptor activity	-16.1635	132.2055	32.37836	30242	10	183	8	4.371585	1.511428	153|1128|1129|1131|1132|1133|11255|59340	ADRB1|CHRM1|CHRM2|CHRM3|CHRM4|CHRM5|HRH3|HRH4	-13.3828	0	1	0	0	-37.4086	165.2568306
1	-15.5773	GO:0016907	M1	1	0	GO Molecular Functions	21	G protein-coupled acetylcholine receptor activity	-15.5773	165.2568	33.91203	30242	7	183	7	3.825137	1.417845	1128|1129|1131|1132|1133|11255|59340	CHRM1|CHRM2|CHRM3|CHRM4|CHRM5|HRH3|HRH4	-12.8476	0	1	0	0	-37.4086	165.2568306
1	-10.3787	GO:0015464	M1	1	0	GO Molecular Functions	21	acetylcholine receptor activity	-10.3787	52.58172	18.8841	30242	22	183	7	3.825137	1.417845	1128|1129|1131|1132|1133|11255|59340	CHRM1|CHRM2|CHRM3|CHRM4|CHRM5|HRH3|HRH4	-8.21333	0	1	0	0	-37.4086	165.2568306
1	-16.8602	GO:0004935	M1	1	0	GO Molecular Functions	21	adrenergic receptor activity	-16.8602	146.895	34.15524	30242	9	183	8	4.371585	1.511428	146|147|148|150|151|152|153|154	ADRA1D|ADRA1B|ADRA1A|ADRA2A|ADRA2B|ADRA2C|ADRB1|ADRB2	-13.9677	0	2	1	1	-16.8602	165.2568306
1	-13.3447	GO:0004936	M1	1	0	GO Molecular Functions	21	alpha-adrenergic receptor activity	-13.3447	165.2568	31.39592	30242	6	183	6	3.278689	1.316393	146|147|148|150|151|152	ADRA1D|ADRA1B|ADRA1A|ADRA2A|ADRA2B|ADRA2C	-10.8913	0	2	0	0	-16.8602	165.2568306
1	-7.71491	GO:0051379	M1	1	0	GO Molecular Functions	21	epinephrine binding	-7.71491	110.1712	20.8669	30242	6	183	4	2.185792	1.080886	150|151|152|1815	ADRA2A|ADRA2B|ADRA2C|DRD4	-5.77983	0	2	0	0	-16.8602	165.2568306
1	-6.66159	GO:0004937	M1	1	0	GO Molecular Functions	21	alpha1-adrenergic receptor activity	-6.66159	165.2568	22.19917	30242	3	183	3	1.639344	0.938686	146|147|148	ADRA1D|ADRA1B|ADRA1A	-4.84696	0	2	0	0	-16.8602	165.2568306
1	-6.66159	GO:0004938	M1	1	0	GO Molecular Functions	21	alpha2-adrenergic receptor activity	-6.66159	165.2568	22.19917	30242	3	183	3	1.639344	0.938686	150|151|152	ADRA2A|ADRA2B|ADRA2C	-4.84696	0	2	0	0	-16.8602	165.2568306
1	-3.63764	GO:0031690	M1	1	0	GO Molecular Functions	21	adrenergic receptor binding	-3.63764	24.78852	8.303425	30242	20	183	3	1.639344	0.938686	150|152|153	ADRA2A|ADRA2C|ADRB1	-2.15244	0	2	0	0	-16.8602	165.2568306
1	-16.8064	GO:0022836	M1	1	0	GO Molecular Functions	21	gated channel activity	-16.8064	11.11376	14.67664	30242	342	183	23	12.56831	2.450458	775|1080|1268|2554|2558|2566|2891|2902|2903|2904|3351|3757|3778|4988|5024|6326|6331|6335|6336|7442|8989|51305|59341	CACNA1C|CFTR|CNR1|GABRA1|GABRA5|GABRG2|GRIA2|GRIN1|GRIN2A|GRIN2B|HTR1B|KCNH2|KCNMA1|OPRM1|P2RX3|SCN2A|SCN5A|SCN9A|SCN10A|TRPV1|TRPA1|KCNK9|TRPV4	-13.9677	0	3	1	1	-16.8064	99.15409836
1	-14.5421	GO:0015267	M1	1	0	GO Molecular Functions	21	channel activity	-14.5421	8.194554	12.44976	30242	484	183	24	13.11475	2.495327	596|775|1080|1268|2554|2558|2566|2891|2902|2903|2904|3351|3757|3778|4988|5024|6326|6331|6335|6336|7442|8989|51305|59341	BCL2|CACNA1C|CFTR|CNR1|GABRA1|GABRA5|GABRG2|GRIA2|GRIN1|GRIN2A|GRIN2B|HTR1B|KCNH2|KCNMA1|OPRM1|P2RX3|SCN2A|SCN5A|SCN9A|SCN10A|TRPV1|TRPA1|KCNK9|TRPV4	-12.0145	0	3	0	0	-16.8064	99.15409836
1	-14.5394	GO:0005216	M1	1	0	GO Molecular Functions	21	ion channel activity	-14.5394	8.737717	12.68337	30242	435	183	23	12.56831	2.450458	775|1080|1268|2554|2558|2566|2891|2902|290	CACNA1C|CFTR|CNR1|GABRA1|GABRA5	-12.0145	0	3	0	0	-16.8064	99.15409836

																		3|2904|3351|3757|3778|4988|5024|6326|6331|6335|6336|7442|8989|51305|59341	|GABRG2|GRIA2|GRIN1|GRIN2A|GRIN2B|HTR1B|KCNH2|KCNMA1|OPRM1|P2RX3|SCN2A|SCN5A|SCN9A|SCN10A|TRPV1|TRPA1|KCNK9|TRPV4							
1	-14.5223	GO:0022803	M1	1	0	GO Molecular Functions	21	passive transmembrane transporter activity	-14.5223	8.177658	12.43356	30242	485	183	24	13.11475	2.495327	596|775|1080|1268|2554|2558|2566|2891|2902|2903|2904|3351|3757|3778|4988|5024|6326|6331|6335|6336|7442|8989|51305|59341	BCL2|CACNA1C|CFTR|CNR1|GABRA1|GABRA5|GABRG2|GRIA2|GRIN1|GRIN2A|GRIN2B|HTR1B|KCNH2|KCNMA1|OPRM1|P2RX3|SCN2A|SCN5A|SCN9A|SCN10A|TRPV1|TRPA1|KCNK9|TRPV4	-12.0145	0	3	0	0	-16.8064	99.15409836
1	-13.9686	GO:0015318	M1	1	0	GO Molecular Functions	21	inorganic molecular entity transmembrane transporter activity	-13.9686	6.252961	11.28836	30242	740	183	28	15.30055	2.661143	775|1080|1268|2554|2558|2566|2891|2902|2903|2904|3351|3757|3778|4363|4988|5024|6326|6331|6335|6336|6523|6530|6531|6532|7442|8989|51305|59341	CACNA1C|CFTR|CNR1|GABRA1|GABRA5|GABRG2|GRIA2|GRIN1|GRIN2A|GRIN2B|HTR1B|KCNH2|KCNMA1|ABCC1|OPRM1|P2RX3|SCN2A|SCN5A|SCN9A|SCN10A|SLC5A1|SLC6A2|SLC6A3|SLC6A4|TRPV1|TRPA1|KCNK9|TRPV4	-11.4888	0	3	0	0	-16.8064	99.15409836
1	-12.6131	GO:0046873	M1	1	0	GO Molecular Functions	21	metal ion transmembrane transporter activity	-12.6131	8.051957	11.50526	30242	431	183	21	11.47541	2.356083	775|1268|2902|2903|2904|3351|3757|3778|4988|6326|6331|6335|6336|6523|6530|6531|6532|7442|8989|51305|59341	CACNA1C|CNR1|GRIN1|GRIN2A|GRIN2B|HTR1B|KCNH2|KCNMA1|OPRM1|SCN2A|SCN5A|SCN9A|SCN10A|SLC5A1|SLC6A2|SLC6A3|SLC6A4|TRPV1|TRPA1|KCNK9|TRPV4	-10.2302	0	3	0	0	-16.8064	99.15409836
1	-12.5862	GO:0005261	M1	1	0	GO Molecular Functions	21	cation channel activity	-12.5862	9.34488	12.00182	30242	336	183	19	10.38251	2.254873	775|1268|2891|2902|2903|2904|3351|3757|3778|4988|5024|6326|6331|6335|6336|7442|8989|51305|59341	CACNA1C|CNR1|GRIA2|GRIN1|GRIN2A|GRIN2B|HTR1B|KCNH2|KCNMA1|OPRM1|P2RX3|SCN2A|SCN5A|SCN9A|SCN10A|TRPV1|TRPA1|KCNK9|TRPV4	-10.2245	0	3	0	0	-16.8064	99.15409836
1	-11.7192	GO:0005244	M1	1	0	GO Molecular Functions	21	voltage-gated ion channel activity	-11.7192	12.27155	12.54139	30242	202	183	15	8.196721	2.027793	775|1268|2902|2903|2904|3351|3757|3778|4988|6326|6331|6335|6336|7442|51305	CACNA1C|CNR1|GRIN1|GRIN2A|GRIN2B|HTR1B|KCNH2|KCNMA1|OPRM1|SCN2A|SCN5A|SCN9A|SCN10A|TRPV1|KCNK9	-9.39703	0	3	0	0	-16.8064	99.15409836

1	-11.6881	GO:0022832	M1	1	0	GO Molecular Functions	21	voltage-gated channel activity	-11.6881	12.2111	12.50517	30242	203	183	15	8.196721	2.027793	775|1268|2902|2903|2904|3351|3757|3778|4988|6326|6331|6335|6336|7442|51305	CACNA1C|CNR1|GRIN1|GRIN2A|GRIN2B|HTR1B|KCNH2|KCNMA1|OPRM1|SCN2A|SCN5A|SCN9A|SCN10A|TRPV1|KCNK9	-9.38445	0	3	0	0	-16.8064	99.15409836
1	-11.6241	GO:0022890	M1	1	0	GO Molecular Functions	21	inorganic cation transmembrane transporter activity	-11.6241	6.334845	10.29864	30242	600	183	23	12.56831	2.450458	775|1268|2891|2902|2903|2904|3351|3757|3778|4988|5024|6326|6331|6335|6336|6523|6530|6531|6532|7442|8989|51305|59341	CACNA1C|CNR1|GRIA2|GRIN1|GRIN2A|GRIN2B|HTR1B|KCNH2|KCNMA1|OPRM1|P2RX3|SCN2A|SCN5A|SCN9A|SCN10A|SLC5A1|SLC6A2|SLC6A3|SLC6A4|TRPV1|TRPA1|KCNK9|TRPV4	-9.35521	0	3	0	0	-16.8064	99.15409836
1	-10.882	GO:0008324	M1	1	0	GO Molecular Functions	21	cation transmembrane transporter activity	-10.882	5.820685	9.717097	30242	653	183	23	12.56831	2.450458	775|1268|2891|2902|2903|2904|3351|3757|3778|4988|5024|6326|6331|6335|6336|6523|6530|6531|6532|7442|8989|51305|59341	CACNA1C|CNR1|GRIA2|GRIN1|GRIN2A|GRIN2B|HTR1B|KCNH2|KCNMA1|OPRM1|P2RX3|SCN2A|SCN5A|SCN9A|SCN10A|SLC5A1|SLC6A2|SLC6A3|SLC6A4|TRPV1|TRPA1|KCNK9|TRPV4	-8.64527	0	3	0	0	-16.8064	99.15409836
1	-10.0971	GO:0015276	M1	1	0	GO Molecular Functions	21	ligand-gated ion channel activity	-10.0971	13.86771	12.03454	30242	143	183	12	6.557377	1.829835	1080|2554|2558|2566|2891|2902|2903|2904|3757|5024|7442|8989	CFTR|GABRA1|GABRA5|GABRG2|GRIA2|GRIN1|GRIN2A|GRIN2B|KCNH2|P2RX3|TRPV1|TRPA1	-7.95736	0	3	0	0	-16.8064	99.15409836
1	-10.0971	GO:0022834	M1	1	0	GO Molecular Functions	21	ligand-gated channel activity	-10.0971	13.86771	12.03454	30242	143	183	12	6.557377	1.829835	1080|2554|2558|2566|2891|2902|2903|2904|3757|5024|7442|8989	CFTR|GABRA1|GABRA5|GABRG2|GRIA2|GRIN1|GRIN2A|GRIN2B|KCNH2|P2RX3|TRPV1|TRPA1	-7.95736	0	3	0	0	-16.8064	99.15409836
1	-9.20681	GO:0005230	M1	1	0	GO Molecular Functions	21	extracellular ligand-gated ion channel activity	-9.20681	20.37413	12.93122	30242	73	183	9	4.918033	1.598524	2554|2558|2566|2891|2902|2903|2904|5024|7442	GABRA1|GABRA5|GABRG2|GRIA2|GRIN1|GRIN2A|GRIN2B|P2RX3|TRPV1	-7.10273	0	3	0	0	-16.8064	99.15409836
1	-8.89542	GO:0022843	M1	1	0	GO Molecular Functions	21	voltage-gated cation channel activity	-8.89542	12.71206	10.95372	30242	143	183	11	6.010929	1.757049	775|1268|2902|2903|2904|3351|3757|3778|4988|7442|51305	CACNA1C|CNR1|GRIN1|GRIN2A|GRIN2B|HTR1B|KCNH2|KCNMA1|OPRM1|TRPV1|KCNK9	-6.84503	0	3	0	0	-16.8064	99.15409836
1	-8.50942	GO:0005262	M1	1	0	GO Molecular Functions	21	calcium channel activity	-8.50942	13.88713	10.99049	30242	119	183	10	5.464481	1.680144	775|1268|2902|2903|2904|3351|4988|7442|8989|59341	CACNA1C|CNR1|GRIN1|GRIN2A|GRIN2B|HTR1B|OPRM1|TRPV1|TRPA1|TRPV4	-6.49766	0	3	0	0	-16.8064	99.15409836
1	-7.97724	GO:0015085	M1	1	0	GO Molecular Functions	21	calcium ion transmembrane transporter activity	-7.97724	12.24125	10.21372	30242	135	183	10	5.464481	1.680144	775|1268|2902|2903|2904|3351|4988|7442|8989|59341	CACNA1C|CNR1|GRIN1|GRIN2A|GRIN2B|HTR1B|OPRM1|TRPV1|TRPA1|TRPV4	-6.02577	0	3	0	0	-16.8064	99.15409836

1	-7.10781	GO:0022824	M1	1	0	GO Molecular Functions	21	transmitter-gated ion channel activity	-7.10781	19.27996	11.05893	30242	60	183	7	3.825137	1.417845	2554|2558|2566|2891|2902|2903|2904	GABRA1|GABRA5|GABRG2|GRIA2|GRIN1|GRIN2A|GRIN2B	-5.25002	0	3	0	0	-16.8064	99.15409836
1	-7.10781	GO:0022835	M1	1	0	GO Molecular Functions	21	transmitter-gated channel activity	-7.10781	19.27996	11.05893	30242	60	183	7	3.825137	1.417845	2554|2558|2566|2891|2902|2903|2904	GABRA1|GABRA5|GABRG2|GRIA2|GRIN1|GRIN2A|GRIN2B	-5.25002	0	3	0	0	-16.8064	99.15409836
1	-6.34129	GO:0099604	M1	1	0	GO Molecular Functions	21	ligand-gated calcium channel activity	-6.34129	31.78016	12.25111	30242	26	183	5	2.73224	1.205087	2902|2903|2904|7442|8989	GRIN1|GRIN2A|GRIN2B|TRPV1|TRPA1	-4.56262	0	3	0	0	-16.8064	99.15409836
1	-6.33799	GO:0099094	M1	1	0	GO Molecular Functions	21	ligand-gated cation channel activity	-6.33799	11.6996	8.891002	30242	113	183	8	4.371585	1.511428	2891|2902|2903|2904|3757|5024|7442|8989	GRIA2|GRIN1|GRIN2A|GRIN2B|KCNH2|P2RX3|TRPV1|TRPA1	-4.56262	0	3	0	0	-16.8064	99.15409836
1	-6.25448	GO:0008066	M1	1	0	GO Molecular Functions	21	glutamate receptor activity	-6.25448	30.60312	12.00727	30242	27	183	5	2.73224	1.205087	2891|2902|2903|2904|2915	GRIA2|GRIN1|GRIN2A|GRIN2B|GRM5	-4.48474	0	3	0	0	-16.8064	99.15409836
1	-5.66547	GO:0022849	M1	1	0	GO Molecular Functions	21	glutamate-gated calcium ion channel activity	-5.66547	99.1541	17.12618	30242	5	183	3	1.639344	0.938686	2902|2903|2904	GRIN1|GRIN2A|GRIN2B	-3.95934	0	3	0	0	-16.8064	99.15409836
1	-5.32939	GO:0004970	M1	1	0	GO Molecular Functions	21	ionotropic glutamate receptor activity	-5.32939	34.79091	11.49593	30242	19	183	4	2.185792	1.080886	2891|2902|2903|2904	GRIA2|GRIN1|GRIN2A|GRIN2B	-3.68295	0	3	0	0	-16.8064	99.15409836
1	-5.11284	GO:0005245	M1	1	0	GO Molecular Functions	21	voltage-gated calcium channel activity	-5.11284	18.36187	9.094047	30242	45	183	5	2.73224	1.205087	775|1268|3351|4988|7442	CACNA1C|CNR1|HTR1B|OPRM1|TRPV1	-3.48588	0	3	0	0	-16.8064	99.15409836
1	-4.92311	GO:0004972	M1	1	0	GO Molecular Functions	21	NMDA glutamate receptor activity	-4.92311	61.97131	13.45734	30242	8	183	3	1.639344	0.938686	2902|2903|2904	GRIN1|GRIN2A|GRIN2B	-3.31113	0	3	0	0	-16.8064	99.15409836
1	-15.0901	GO:0008528	M1	1	0	GO Molecular Functions	21	G protein-coupled peptide receptor activity	-15.0901	17.98714	16.10898	30242	147	183	16	8.743169	2.088053	185|624|1230|1909|1910|2149|3061|3062|3577|4985|4986|4988|10203|10280|10800|729230	AGTR1|BDKRB2|CCR1|EDNRA|EDNRB|F2R|HCRTR1|HCRTR2|CXCR1|OPRD1|OPRK1|OPRM1|CALCRL|SIGMAR1|CYSLTR1|CCR2	-12.4062	0	4	1	1	-15.0901	94.43247463
1	-14.8108	GO:0001653	M1	1	0	GO Molecular Functions	21	peptide receptor activity	-14.8108	17.28176	15.75359	30242	153	183	16	8.743169	2.088053	185|624|1230|1909|1910|2149|3061|3062|3577|4985|4986|4988|10203|10280|10800|729230	AGTR1|BDKRB2|CCR1|EDNRA|EDNRB|F2R|HCRTR1|HCRTR2|CXCR1|OPRD1|OPRK1|OPRM1|CALCRL|SIGMAR1|CYSLTR1|CCR2	-12.1683	0	4	0	0	-15.0901	94.43247463
1	-7.349	GO:0004985	M1	1	0	GO Molecular Functions	21	G protein-coupled opioid receptor activity	-7.349	94.43247	19.28982	30242	7	183	4	2.185792	1.080886	4985|4986|4988|10280	OPRD1|OPRK1|OPRM1|SIGMAR1	-5.46448	0	4	0	0	-15.0901	94.43247463
1	-3.29117	GO:0042923	M1	1	0	GO Molecular Functions	21	neuropeptide binding	-3.29117	19.0681	7.191458	30242	26	183	3	1.639344	0.938686	4985|4986|4988	OPRD1|OPRK1|OPRM1	-1.85283	0	4	0	0	-15.0901	94.43247463
1	-14.7139	GO:1901338	M1	1	0	GO Molecular Functions	21	catecholamine binding	-14.7139	101.6965	28.33417	30242	13	183	8	4.371585	1.511428	150|151|152|154|1812|1813|1815|6531	ADRA2A|ADRA2B|ADRA2C|ADRB2|DRD1|DRD2|DRD4|SLC6A3	-12.1092	0	5	1	1	-14.7139	165.2568306
1	-8.18997	GO:0004952	M1	1	0	GO Molecular Functions	21	dopamine neurotransmitter receptor activity	-8.18997	132.2055	22.89306	30242	5	183	4	2.185792	1.080886	1812|1813|1814|1815	DRD1|DRD2|DRD3|DRD4	-6.22212	0	5	0	0	-14.7139	165.2568306
1	-7.349	GO:0035240	M1	1	0	GO Molecular Functions	21	dopamine binding	-7.349	94.43247	19.28982	30242	7	183	4	2.185792	1.080886	1812|1813|1815|6531	DRD1|DRD2|DRD4|SLC6A3	-5.46448	0	5	0	0	-14.7139	165.2568306
1	-6.66159	GO:0001591	M1	1	0	GO Molecular Functions	21	dopamine neurotransmitter receptor activity,	-6.66159	165.2568	22.19917	30242	3	183	3	1.639344	0.938686	1813|1814|1815	DRD2|DRD3|DRD4	-4.84696	0	5	0	0	-14.7139	165.2568306

								coupled via Gi/Go																		
1	-12.9963	GO:0016773	M1	1	0	GO Molecular Functions	21	phosphotransferase activity, alcohol group as acceptor	-12.9963	6.263378	10.88028	30242	686	183	26	14.20765	2.580833	472|595|673|1453|1956|2050|2263|3551|3643|4914|4915|5159|5290|5291|5293|5582|5591|5979|6197|6347|6793|7046|8986|9475|29110|225689	ATM|CCND1|BRAF|CSNK1D|EGFR|EPHB4|FGFR2|IKBKB|INSR|NTRK1|NTRK2|PDGFRB|PIK3CA|PIK3CB|PIK3CD|PRKCG|PRKDC|RET|RPS6KA3|CCL2|STK10|TGFBR1|RPS6KA4|ROCK2|TBK1|MAPK15	-10.5677	0	6	1	1	-12.9963	6.861875319
1	-12.8598	GO:0004672	M1	1	0	GO Molecular Functions	21	protein kinase activity	-12.8598	6.861875	11.10253	30242	578	183	24	13.11475	2.495327	472|595|673|1453|1956|2050|2263|3551|3643|4914|4915|5159|5290|5582|5591|5979|6197|6347|6793|7046|8986|9475|29110|225689	ATM|CCND1|BRAF|CSNK1D|EGFR|EPHB4|FGFR2|IKBKB|INSR|NTRK1|NTRK2|PDGFRB|PIK3CA|PRKCG|PRKDC|RET|RPS6KA3|CCL2|STK10|TGFBR1|RPS6KA4|ROCK2|TBK1|MAPK15	-10.4547	0	6	0	0	-12.9963	6.861875319
1	-12.1655	GO:0016301	M1	1	0	GO Molecular Functions	21	kinase activity	-12.1655	5.759621	10.27062	30242	746	183	26	14.20765	2.580833	472|595|673|1453|1956|2050|2263|3551|3643|4914|4915|5159|5290|5291|5293|5582|5591|5979|6197|6347|6793|7046|8986|9475|29110|225689	ATM|CCND1|BRAF|CSNK1D|EGFR|EPHB4|FGFR2|IKBKB|INSR|NTRK1|NTRK2|PDGFRB|PIK3CA|PIK3CB|PIK3CD|PRKCG|PRKDC|RET|RPS6KA3|CCL2|STK10|TGFBR1|RPS6KA4|ROCK2|TBK1|MAPK15	-9.82403	0	6	0	0	-12.9963	6.861875319
1	-6.92487	GO:0004674	M1	1	0	GO Molecular Functions	21	protein serine/threonine kinase activity	-6.92487	5.962875	7.676872	30242	388	183	14	7.650273	1.964856	472|673|1453|1956|3551|5582|5591|6197|6793|7046|8986|9475|29110|225689	ATM|BRAF|CSNK1D|EGFR|IKBKB|PRKCG|PRKDC|RPS6KA3|STK10|TGFBR1|RPS6KA4|ROCK2|TBK1|MAPK15	-5.07352	0	6	0	0	-12.9963	6.861875319
1	-6.47122	GO:0106310	M1	1	0	GO Molecular Functions	21	protein serine kinase activity	-6.47122	5.967608	7.398268	30242	360	183	13	7.103825	1.898976	472|673|1453|3551|5290|5582|5591|6197|6793|8986|9475|29110|225689	ATM|BRAF|CSNK1D|IKBKB|PIK3CA|PRKCG|PRKDC|RPS6KA3|STK10|RPS6KA4|ROCK2|TBK1|MAPK15	-4.67945	0	6	0	0	-12.9963	6.861875319
1	-11.6604	GO:0042277	M1	1	0	GO Molecular Functions	21	peptide binding	-11.6604	8.986776	11.40004	30242	331	183	18	9.836066	2.201413	154|1910|2891|2902|2903|2904|3061|3062|3643|4792|4985|4986|4988|5468|5970|7099|10203|23621	ADRB2|EDNRB|GRIA2|GRIN1|GRIN2A|GRIN2B|HCRTR1|HCRTR2|INSR|NFKBIA|OPRD1|OPRK1|OPRM1|PPARG|RELA|TLR4|CALCRL|BACE1	-9.37448	0	7	1	1	-11.6604	15.02334824
1	-11.033	GO:0033218	M1	1	0	GO Molecular Functions	21	amide binding	-11.033	7.621067	10.55811	30242	412	183	19	10.38251	2.254873	154|1910|2891|2902|2903|2904|3061|3062|3643|4792|4985|4986|4988|	ADRB2|EDNRB|GRIA2|GRIN1|GRIN2A|GRIN2B|HCRTR1|HCRTR	-8.78055	0	7	0	0	-11.6604	15.02334824

																		5468|5970|7099|7298|10203|23621	2|INSR|NFKBIA|OPRD1|OPRK1|OPRM1|PPARG|RELA|TLR4|TYMS|CALCRL|BACE1							
1	-7.18315	GO:0001540	M1	1	0	GO Molecular Functions	21	amyloid-beta binding	-7.18315	15.02335	10.27914	30242	88	183	8	4.371585	1.511428	154|2891|2902|2903|2904|3643|7099|23621	ADRB2|GRIA2|GRIN1|GRIN2A|GRIN2B|INSR|TLR4|BACE1	-5.30548	0	7	0	0	-11.6604	15.02334824
1	-10.5809	GO:0004879	M1	1	0	GO Molecular Functions	21	nuclear receptor activity	-10.5809	28.60214	15.54352	30242	52	183	9	4.918033	1.598524	367|2099|2100|2908|5241|5465|5467|5468|7421	AR|ESR1|ESR2|NR3C1|PGR|PPARA|PPARD|PPARG|VDR	-8.37417	0	8	1	1	-10.5809	41.31420765
1	-10.5809	GO:0098531	M1	1	0	GO Molecular Functions	21	ligand-activated transcription factor activity	-10.5809	28.60214	15.54352	30242	52	183	9	4.918033	1.598524	367|2099|2100|2908|5241|5465|5467|5468|7421	AR|ESR1|ESR2|NR3C1|PGR|PPARA|PPARD|PPARG|VDR	-8.37417	0	8	0	0	-10.5809	41.31420765
1	-8.25497	GO:0003707	M1	1	0	GO Molecular Functions	21	steroid hormone receptor activity	-8.25497	41.31421	15.41584	30242	24	183	6	3.278689	1.316393	2099|2100|2908|5241|5465|5467	ESR1|ESR2|NR3C1|PGR|PPARA|PPARD	-6.27008	0	8	0	0	-10.5809	41.31420765
1	-7.44423	GO:0001223	M1	1	0	GO Molecular Functions	21	transcription coactivator binding	-7.44423	30.98566	13.24188	30242	32	183	6	3.278689	1.316393	367|2099|5241|5465|5467|5970	AR|ESR1|PGR|PPARA|PPARD|RELA	-5.53852	0	8	0	0	-10.5809	41.31420765
1	-6.24952	GO:0008134	M1	1	0	GO Molecular Functions	21	transcription factor binding	-6.24952	4.550963	6.74338	30242	581	183	16	8.743169	2.088053	328|367|595|596|2099|3066|4792|5241|5465|5467|5468|5591|5970|7157|7421|114548	APEX1|AR|CCND1|BCL2|ESR1|HDAC2|NFKBIA|PGR|PPARA|PPARD|PPARG|PRKDC|RELA|TP53|VDR|NLRP3	-4.48474	0	8	0	0	-10.5809	41.31420765
1	-6.09112	GO:0051059	M1	1	0	GO Molecular Functions	21	NF-kappaB binding	-6.09112	28.49256	11.55722	30242	29	183	5	2.73224	1.205087	328|3066|4792|5467|5970	APEX1|HDAC2|NFKBIA|PPARD|RELA	-4.33668	0	8	0	0	-10.5809	41.31420765
1	-6.03071	GO:0140297	M1	1	0	GO Molecular Functions	21	DNA-binding transcription factor binding	-6.03071	5.452637	6.941352	30242	394	183	13	7.103825	1.898976	328|367|596|2099|3066|4792|5465|5467|5468|5970|7157|7421|114548	APEX1|AR|BCL2|ESR1|HDAC2|NFKBIA|PPARA|PPARD|PPARG|RELA|TP53|VDR|NLRP3	-4.30943	0	8	0	0	-10.5809	41.31420765
1	-5.63484	GO:0061629	M1	1	0	GO Molecular Functions	21	RNA polymerase II-specific DNA-binding transcription factor binding	-5.63484	6.079683	6.887488	30242	299	183	11	6.010929	1.757049	328|367|2099|3066|4792|5465|5467|5468|5970|7157|7421	APEX1|AR|ESR1|HDAC2|NFKBIA|PPARA|PPARD|PPARG|RELA|TP53|VDR	-3.93326	0	8	0	0	-10.5809	41.31420765
1	-5.57866	GO:0005496	M1	1	0	GO Molecular Functions	21	steroid binding	-5.57866	11.56798	8.259281	30242	100	183	7	3.825137	1.417845	367|2099|2100|2908|5241|6462|7421	AR|ESR1|ESR2|NR3C1|PGR|SHBG|VDR	-3.89043	0	8	0	0	-10.5809	41.31420765
1	-5.09187	GO:0001221	M1	1	0	GO Molecular Functions	21	transcription coregulator binding	-5.09187	12.71206	8.08114	30242	78	183	6	3.278689	1.316393	367|2099|5241|5465|5467|5970	AR|ESR1|PGR|PPARA|PPARD|RELA	-3.46871	0	8	0	0	-10.5809	41.31420765
1	-3.76048	GO:0140296	M1	1	0	GO Molecular Functions	21	general transcription initiation factor binding	-3.76048	14.37016	7.080715	30242	46	183	4	2.185792	1.080886	367|2099|5970|7157	AR|ESR1|RELA|TP53	-2.25847	0	8	0	0	-10.5809	41.31420765
1	-3.63764	GO:0001091	M1	1	0	GO Molecular Functions	21	RNA polymerase II general transcription initiation factor binding	-3.63764	24.78852	8.303425	30242	20	183	3	1.639344	0.938686	367|2099|7157	AR|ESR1|TP53	-2.15244	0	8	0	0	-10.5809	41.31420765
1	-2.67313	GO:0051117	M1	1	0	GO Molecular Functions	21	ATPase binding	-2.67313	7.427273	4.737976	30242	89	183	4	2.185792	1.080886	367|1956|2099|5241	AR|EGFR|ESR1|PGR	-1.27931	0	8	0	0	-10.5809	41.31420765
1	-2.5752	GO:0003682	M1	1	0	GO Molecular Functions	21	chromatin binding	-2.5752	2.889105	3.559031	30242	572	183	10	5.464481	1.680144	328|367|1956|2099|3066|4221|5468|5970|7157|225689	APEX1|AR|EGFR|ESR1|HDAC2|MEN1|PPARG|RELA|TP53|MAPK15	-1.1902	0	8	0	0	-10.5809	41.31420765
1	-2.05227	GO:0001098	M1	1	0	GO Molecular Functions	21	basal transcription machinery binding	-2.05227	7.082436	3.975217	30242	70	183	3	1.639344	0.938686	367|2099|7157	AR|ESR1|TP53	-0.70287	0	8	0	0	-10.5809	41.31420765

1	-2.05227	GO:0001099	M1	1	0	GO Molecular Functions	21	basal RNA polymerase II transcription machinery binding	-2.05227	7.082436	3.975217	30242	70	183	3	1.639344	0.938686	367|2099|7157	AR|ESR1|TP53	-0.70287	0	8	0	0	-10.5809	41.31420765
1	-10.3921	GO:0043176	M1	1	0	GO Molecular Functions	21	amine binding	-10.3921	82.62842	22.0673	30242	12	183	6	3.278689	1.316393	3350|3351|3356|3357|3358|6532	HTR1A|HTR1B|HTR2A|HTR2B|HTR2C|SLC6A4	-8.21343	0	9	1	1	-10.3921	165.2568306
1	-10.3921	GO:0051378	M1	1	0	GO Molecular Functions	21	serotonin binding	-10.3921	82.62842	22.0673	30242	12	183	6	3.278689	1.316393	3350|3351|3356|3357|3358|6532	HTR1A|HTR1B|HTR2A|HTR2B|HTR2C|SLC6A4	-8.21343	0	9	0	0	-10.3921	165.2568306
1	-6.66159	GO:0001587	M1	1	0	GO Molecular Functions	21	Gq/11-coupled serotonin receptor activity	-6.66159	165.2568	22.19917	30242	3	183	3	1.639344	0.938686	3356|3357|3358	HTR2A|HTR2B|HTR2C	-4.84696	0	9	0	0	-10.3921	165.2568306
1	-9.66501	GO:0008081	M1	1	0	GO Molecular Functions	21	phosphoric diester hydrolase activity	-9.66501	18.16009	12.79163	30242	91	183	10	5.464481	1.680144	328|624|1128|1131|1133|1230|1909|5138|5142|8654	APEX1|BDKRB2|CHRM1|CHRM3|CHRM5|CCR1|EDNRA|PDE2A|PDE4B|PDE5A	-7.53746	0	10	1	1	-9.66501	38.13619168
1	-8.0262	GO:0004435	M1	1	0	GO Molecular Functions	21	phosphatidylinositol phospholipase C activity	-8.0262	38.13619	14.78094	30242	26	183	6	3.278689	1.316393	624|1128|1131|1133|1230|1909	BDKRB2|CHRM1|CHRM3|CHRM5|CCR1|EDNRA	-6.06662	0	10	0	0	-9.66501	38.13619168
1	-7.81668	GO:0004629	M1	1	0	GO Molecular Functions	21	phospholipase C activity	-7.81668	35.41218	14.21423	30242	28	183	6	3.278689	1.316393	624|1128|1131|1133|1230|1909	BDKRB2|CHRM1|CHRM3|CHRM5|CCR1|EDNRA	-5.87319	0	10	0	0	-9.66501	38.13619168
1	-7.13465	GO:0042578	M1	1	0	GO Molecular Functions	21	phosphoric ester hydrolase activity	-7.13465	6.202669	7.888722	30242	373	183	14	7.650273	1.964856	52|249|328|624|761|1128|1131|1133|1230|1909|5138|5142|5444|8654	ACP1|ALPL|APEX1|BDKRB2|CA3|CHRM1|CHRM3|CHRM5|CCR1|EDNRA|PDE2A|PDE4B|PON1|PDE5A	-5.26371	0	10	0	0	-9.66501	38.13619168
1	-5.26949	GO:0016788	M1	1	0	GO Molecular Functions	21	hydrolase activity, acting on ester bonds	-5.26949	3.624989	5.776259	30242	775	183	17	9.289617	2.145862	52|249|328|624|759|760|761|1128|1131|1133|1230|1909|2166|5138|5142|5444|8654	ACP1|ALPL|APEX1|BDKRB2|CA1|CA2|CA3|CHRM1|CHRM3|CHRM5|CCR1|EDNRA|FAAH|PDE2A|PDE4B|PON1|PDE5A	-3.62702	0	10	0	0	-9.66501	38.13619168
1	-4.7398	GO:0016298	M1	1	0	GO Molecular Functions	21	lipase activity	-4.7398	8.63282	6.90928	30242	134	183	7	3.825137	1.417845	624|1128|1131|1133|1230|1909|2166	BDKRB2|CHRM1|CHRM3|CHRM5|CCR1|EDNRA|FAAH	-3.16315	0	10	0	0	-9.66501	38.13619168
1	-4.28479	GO:0004620	M1	1	0	GO Molecular Functions	21	phospholipase activity	-4.28479	9.180935	6.645425	30242	108	183	6	3.278689	1.316393	624|1128|1131|1133|1230|1909	BDKRB2|CHRM1|CHRM3|CHRM5|CCR1|EDNRA	-2.74081	0	10	0	0	-9.66501	38.13619168
1	-9.451	GO:0019904	M1	1	0	GO Molecular Functions	21	protein domain specific binding	-9.451	5.171622	8.732148	30242	703	183	22	12.02186	2.404073	153|351|367|596|834|890|1080|1814|1815|2335|3643|3757|4988|5138|5465|5468|5591|5970|6331|7157|59341|225689	ADRB1|APP|AR|BCL2|CASP1|CCNA2|CFTR|DRD3|DRD4|FN1|INSR|KCNH2|OPRM1|PDE2A|PPARA|PPARG|PRKDC|RELA|SCN5A|TP53|TRPV4|MAPK15	-7.33535	0	11	1	1	-9.451	5.17162201
1	-9.1652	GO:0016705	M1	1	0	GO Molecular Functions	21	oxidoreductase activity, acting on paired donors, with incorporation or reduction of molecular oxygen	-9.1652	11.52955	10.80547	30242	172	183	12	6.557377	1.829835	1543|1544|1545|1557|1588|1645|4842|4843|4846|5742|5743|6916	CYP1A1|CYP1A2|CYP1B1|CYP2C19|CYP19A1|AKR1C1|NOS1|NOS2|NOS3|PTGS1|PTGS2|TBXAS1	-7.0724	0	12	1	1	-9.1652	94.43247463

1	-9.12741	GO:0004497	M1	1	0	GO Molecular Functions	21	monooxygenase activity	-9.12741	16.04435	11.9334	30242	103	183	10	5.464481	1.680144	1543|1544|1545|1557|1588|1645|4842|4843|4846|6916	CYP1A1|CYP1A2|CYP1B1|CYP2C19|CYP19A1|AKR1C1|NOS1|NOS2|NOS3|TBXAS1	-7.04561	0	12	0	0	-9.1652	94.43247463
1	-9.02694	GO:0020037	M1	1	0	GO Molecular Functions	21	heme binding	-9.02694	13.07788	11.13601	30242	139	183	11	6.010929	1.757049	1543|1544|1545|1557|1588|4842|4843|4846|5742|5743|6916	CYP1A1|CYP1A2|CYP1B1|CYP2C19|CYP19A1|NOS1|NOS2|NOS3|PTGS1|PTGS2|TBXAS1	-6.95586	0	12	0	0	-9.1652	94.43247463
1	-9.00445	GO:0016491	M1	1	0	GO Molecular Functions	21	oxidoreductase activity	-9.00445	4.893204	8.383671	30242	743	183	22	12.02186	2.404073	217|328|1543|1544|1545|1557|1588|1645|1723|1728|2936|3156|4128|4129|4842|4843|4846|5498|5742|5743|6916|51179	ALDH2|APEX1|CYP1A1|CYP1A2|CYP1B1|CYP2C19|CYP19A1|AKR1C1|DHODH|NQO1|GSR|HMGCR|MAOA|MAOB|NOS1|NOS2|NOS3|PPOX|PTGS1|PTGS2|TBXAS1|HAO2	-6.94385	0	12	0	0	-9.1652	94.43247463
1	-8.70561	GO:0046906	M1	1	0	GO Molecular Functions	21	tetrapyrrole binding	-8.70561	12.20017	10.69354	30242	149	183	11	6.010929	1.757049	1543|1544|1545|1557|1588|4842|4843|4846|5742|5743|6916	CYP1A1|CYP1A2|CYP1B1|CYP2C19|CYP19A1|NOS1|NOS2|NOS3|PTGS1|PTGS2|TBXAS1	-6.67496	0	12	0	0	-9.1652	94.43247463
1	-7.349	GO:0106256	M1	1	0	GO Molecular Functions	21	hydroperoxy icosatetraenoate dehydratase activity	-7.349	94.43247	19.28982	30242	7	183	4	2.185792	1.080886	1543|1544|1545|6916	CYP1A1|CYP1A2|CYP1B1|TBXAS1	-5.46448	0	12	0	0	-9.1652	94.43247463
1	-6.43191	GO:0070330	M1	1	0	GO Molecular Functions	21	aromatase activity	-6.43191	33.05137	12.50913	30242	25	183	5	2.73224	1.205087	1543|1544|1545|1557|1588	CYP1A1|CYP1A2|CYP1B1|CYP2C19|CYP19A1	-4.64568	0	12	0	0	-9.1652	94.43247463
1	-5.48428	GO:0008395	M1	1	0	GO Molecular Functions	21	steroid hydroxylase activity	-5.48428	21.74432	9.983783	30242	38	183	5	2.73224	1.205087	1543|1544|1545|1557|1588	CYP1A1|CYP1A2|CYP1B1|CYP2C19|CYP19A1	-3.80042	0	12	0	0	-9.1652	94.43247463
1	-5.37096	GO:0016712	M1	1	0	GO Molecular Functions	21	oxidoreductase activity, acting on paired donors, with incorporation or reduction of molecular oxygen, reduced flavin or flavoprotein as one donor, and incorporation of one atom of oxygen	-5.37096	20.6571	9.70662	30242	40	183	5	2.73224	1.205087	1543|1544|1545|1557|1588	CYP1A1|CYP1A2|CYP1B1|CYP2C19|CYP19A1	-3.70754	0	12	0	0	-9.1652	94.43247463
1	-4.92311	GO:0101020	M1	1	0	GO Molecular Functions	21	estrogen 16-alpha-hydroxylase activity	-4.92311	61.97131	13.45734	30242	8	183	3	1.639344	0.938686	1543|1544|1545	CYP1A1|CYP1A2|CYP1B1	-3.31113	0	12	0	0	-9.1652	94.43247463
1	-3.48675	GO:0005506	M1	1	0	GO Molecular Functions	21	iron ion binding	-3.48675	6.566497	5.350427	30242	151	183	6	3.278689	1.316393	1543|1544|1545|1557|1588|6916	CYP1A1|CYP1A2|CYP1B1|CYP2C19|CYP19A1|TBXAS1	-2.02038	0	12	0	0	-9.1652	94.43247463
1	-2.77031	GO:0019825	M1	1	0	GO Molecular Functions	21	oxygen binding	-2.77031	12.71206	5.710538	30242	39	183	3	1.639344	0.938686	1543|1557|1588	CYP1A1|CYP2C19|CYP19A1	-1.36748	0	12	0	0	-9.1652	94.43247463
1	-8.77609	GO:0031406	M1	1	0	GO Molecular Functions	21	carboxylic acid binding	-8.77609	10.66173	10.31293	30242	186	183	12	6.557377	1.829835	1645|2902|2904|4842|4843|4846|5467|5468|6401|7298|7364|7421	AKR1C1|GRIN1|GRIN2B|NOS1|NOS2|NOS3|PPARD|PPARG|SELE|TYMS|UGT2B7|VDR	-6.73568	0	13	1	1	-8.77609	11.47616879

1	-7.70781	GO:0043177	M1	1	0	GO Molecular Functions	21	organic acid binding	-7.70781	11.47617	9.832213	30242	144	183	10	5.464481	1.680144	2902|2904|4842|4843|4846|5467|5468|7298|7364|7421	GRIN1|GRIN2B|NOS1|NOS2|NOS3|PPARD|PPARG|TYMS|UGT2B7|VDR	-5.77983	0	13	0	0	-8.77609	11.47616879
1	-3.86758	GO:0033293	M1	1	0	GO Molecular Functions	21	monocarboxylic acid binding	-3.86758	10.20104	6.46983	30242	81	183	5	2.73224	1.205087	1645|5467|5468|7364|7421	AKR1C1|PPARD|PPARG|UGT2B7|VDR	-2.35691	0	13	0	0	-8.77609	11.47616879
1	-8.56059	GO:0042562	M1	1	0	GO Molecular Functions	21	hormone binding	-8.56059	17.29432	11.80687	30242	86	183	9	4.918033	1.598524	367|1910|1956|2908|3061|3062|3643|6462|10203	AR|EDNRB|EGFR|NR3C1|HCRTR1|HCRTR2|INSR|SHBG|CALCRL	-6.53949	0	14	1	1	-8.56059	17.29431948
1	-4.7999	GO:0017046	M1	1	0	GO Molecular Functions	21	peptide hormone binding	-4.7999	15.89008	8.385008	30242	52	183	5	2.73224	1.205087	1910|3061|3062|3643|10203	EDNRB|HCRTR1|HCRTR2|INSR|CALCRL	-3.19883	0	14	0	0	-8.56059	17.29431948
1	-8.41237	GO:0001664	M1	1	0	GO Molecular Functions	21	G protein-coupled receptor binding	-8.41237	7.842697	9.215057	30242	295	183	14	7.650273	1.964856	134|135|150|152|153|185|624|1636|1814|1910|3558|3576|6347|729230	ADORA1|ADORA2A|ADRA2A|ADRA2C|ADRB1|AGTR1|BDKRB2|ACE|DRD3|EDNRB|IL2|CXCL8|CCL2|CCR2	-6.40976	0	15	1	1	-8.41237	7.842697045
1	-6.07336	GO:0046982	M1	1	0	GO Molecular Functions	21	protein heterodimerization activity	-6.07336	6.045982	7.169382	30242	328	183	12	6.557377	1.829835	134|147|148|150|152|153|185|596|624|3551|7099|7157	ADORA1|ADRA1B|ADRA1A|ADRA2A|ADRA2C|ADRB1|AGTR1|BCL2|BDKRB2|IKBKB|TLR4|TP53	-4.327	0	15	0	0	-8.41237	7.842697045
1	-8.29155	GO:0016836	M1	1	0	GO Molecular Functions	21	hydro-lyase activity	-8.29155	20.6571	12.28289	30242	64	183	8	4.371585	1.511428	759|760|761|762|1543|1544|1545|6916	CA1|CA2|CA3|CA4|CYP1A1|CYP1A2|CYP1B1|TBXAS1	-6.29789	0	16	1	1	-8.29155	82.6284153
1	-7.51113	GO:0016835	M1	1	0	GO Molecular Functions	21	carbon-oxygen lyase activity	-7.51113	16.52568	10.84931	30242	80	183	8	4.371585	1.511428	759|760|761|762|1543|1544|1545|6916	CA1|CA2|CA3|CA4|CYP1A1|CYP1A2|CYP1B1|TBXAS1	-5.5907	0	16	0	0	-8.29155	82.6284153
1	-5.77441	GO:0004089	M1	1	0	GO Molecular Functions	21	carbonate dehydratase activity	-5.77441	44.06849	13.01799	30242	15	183	4	2.185792	1.080886	759|760|761|762	CA1|CA2|CA3|CA4	-4.06368	0	16	0	0	-8.29155	82.6284153
1	-5.36639	GO:0004064	M1	1	0	GO Molecular Functions	21	arylesterase activity	-5.36639	82.62842	15.60239	30242	6	183	3	1.639344	0.938686	759|760|5444	CA1|CA2|PON1	-3.70754	0	16	0	0	-8.29155	82.6284153
1	-4.49962	GO:0016829	M1	1	0	GO Molecular Functions	21	lyase activity	-4.49962	6.610273	6.211135	30242	200	183	8	4.371585	1.511428	759|760|761|762|1543|1544|1545|6916	CA1|CA2|CA3|CA4|CYP1A1|CYP1A2|CYP1B1|TBXAS1	-2.93962	0	16	0	0	-8.29155	82.6284153
1	-8.18997	GO:0001609	M1	1	0	GO Molecular Functions	21	G protein-coupled adenosine receptor activity	-8.18997	132.2055	22.89306	30242	5	183	4	2.185792	1.080886	134|135|136|140	ADORA1|ADORA2A|ADORA2B|ADORA3	-6.22212	0	17	1	1	-8.18997	132.2054645
1	-7.49778	GO:0010181	M1	1	0	GO Molecular Functions	21	FMN binding	-7.49778	51.64276	15.80969	30242	16	183	5	2.73224	1.205087	1723|4842|4843|4846|51179	DHODH|NOS1|NOS2|NOS3|HAO2	-5.58477	0	18	1	1	-7.49778	165.2568306
1	-6.66159	GO:0004517	M1	1	0	GO Molecular Functions	21	nitric-oxide synthase activity	-6.66159	165.2568	22.19917	30242	3	183	3	1.639344	0.938686	4842|4843|4846	NOS1|NOS2|NOS3	-4.84696	0	18	0	0	-7.49778	165.2568306
1	-6.64034	GO:0016709	M1	1	0	GO Molecular Functions	21	oxidoreductase activity, acting on paired donors, with incorporation or reduction of molecular oxygen, NAD(P)H as one donor, and incorporation of one atom of oxygen	-6.64034	23.05909	11.29437	30242	43	183	6	3.278689	1.316393	1543|1557|1645|4842|4843|4846	CYP1A1|CYP2C19|AKR1C1|NOS1|NOS2|NOS3	-4.83155	0	18	0	0	-7.49778	165.2568306

1	-6.06147	GO:0034617	M1	1	0	GO Molecular Functions	21	tetrahydrobiopterin binding	-6.06147	123.9426	19.18635	30242	4	183	3	1.639344	0.938686	4842|4843|4846	NOS1|NOS2|NOS3	-4.327	0	18	0	0	-7.49778	165.2568306
1	-6.05651	GO:0050660	M1	1	0	GO Molecular Functions	21	flavin adenine dinucleotide binding	-6.05651	13.60939	9.083343	30242	85	183	7	3.825137	1.417845	2936|4128|4129|4842|4843|4846|5498	GSR|MAOA|MAOB|NOS1|NOS2|NOS3|PPOX	-4.3269	0	18	0	0	-7.49778	165.2568306
1	-5.39158	GO:0005516	M1	1	0	GO Molecular Functions	21	calmodulin binding	-5.39158	7.39956	7.102762	30242	201	183	9	4.918033	1.598524	775|1956|2902|4842|4843|4846|6331|7442|59341	CACNA1C|EGFR|GRIN1|NOS1|NOS2|NOS3|SCN5A|TRPV1|TRPV4	-3.71632	0	18	0	0	-7.49778	165.2568306
1	-4.75899	GO:0016597	M1	1	0	GO Molecular Functions	21	amino acid binding	-4.75899	15.59027	8.294938	30242	53	183	5	2.73224	1.205087	2902|2904|4842|4843|4846	GRIN1|GRIN2B|NOS1|NOS2|NOS3	-3.16891	0	18	0	0	-7.49778	165.2568306
1	-4.75899	GO:0050661	M1	1	0	GO Molecular Functions	21	NADP binding	-4.75899	15.59027	8.294938	30242	53	183	5	2.73224	1.205087	2936|3156|4842|4843|4846	GSR|HMGCR|NOS1|NOS2|NOS3	-3.16891	0	18	0	0	-7.49778	165.2568306
1	-4.74896	GO:0034618	M1	1	0	GO Molecular Functions	21	arginine binding	-4.74896	55.08561	12.6619	30242	9	183	3	1.639344	0.938686	4842|4843|4846	NOS1|NOS2|NOS3	-3.16891	0	18	0	0	-7.49778	165.2568306
1	-6.83853	GO:0002020	M1	1	0	GO Molecular Functions	21	protease binding	-6.83853	11.01712	9.101489	30242	135	183	9	4.918033	1.598524	596|624|836|1991|2335|4915|6531|7124|7157	BCL2|BDKRB2|CASP3|ELANE|FN1|NTRK2|SLC6A3|TNF|TP53	-4.99352	0	19	1	1	-6.83853	20.65710383
1	-4.38865	GO:0051721	M1	1	0	GO Molecular Functions	21	protein phosphatase 2A binding	-4.38865	20.6571	8.680715	30242	32	183	4	2.185792	1.080886	596|3156|6531|7157	BCL2|HMGCR|SLC6A3|TP53	-2.84152	0	19	0	0	-6.83853	20.65710383
1	-3.69783	GO:0019902	M1	1	0	GO Molecular Functions	21	phosphatase binding	-3.69783	5.90203	5.372136	30242	196	183	7	3.825137	1.417845	596|1956|3156|5465|6531|7157|29110	BCL2|EGFR|HMGCR|PPARA|SLC6A3|TP53|TBK1	-2.2043	0	19	0	0	-6.83853	20.65710383
1	-3.5022	GO:0019903	M1	1	0	GO Molecular Functions	21	protein phosphatase binding	-3.5022	6.610273	5.37453	30242	150	183	6	3.278689	1.316393	596|1956|3156|6531|7157|29110	BCL2|EGFR|HMGCR|SLC6A3|TP53|TBK1	-2.03319	0	19	0	0	-6.83853	20.65710383
1	-3.32058	GO:0031625	M1	1	0	GO Molecular Functions	21	ubiquitin protein ligase binding	-3.32058	4.451362	4.663833	30242	297	183	8	4.371585	1.511428	596|1956|3757|4792|5243|5970|6331|7157	BCL2|EGFR|KCNH2|NFKBIA|ABCB1|RELA|SCN5A|TP53	-1.86972	0	19	0	0	-6.83853	20.65710383
1	-3.14462	GO:0044389	M1	1	0	GO Molecular Functions	21	ubiquitin-like protein ligase binding	-3.14462	4.183717	4.43905	30242	316	183	8	4.371585	1.511428	596|1956|3757|4792|5243|5970|6331|7157	BCL2|EGFR|KCNH2|NFKBIA|ABCB1|RELA|SCN5A|TP53	-1.71844	0	19	0	0	-6.83853	20.65710383
1	-6.57703	GO:0016303	M1	1	0	GO Molecular Functions	21	1-phosphatidylinositol-3-kinase activity	-6.57703	66.10273	16.06579	30242	10	183	4	2.185792	1.080886	472|5290|5291|5293	ATM|PIK3CA|PIK3CB|PIK3CD	-4.77398	0	20	1	1	-6.57703	70.82435597
1	-6.20876	GO:0035004	M1	1	0	GO Molecular Functions	21	phosphatidylinositol 3-kinase activity	-6.20876	55.08561	14.62142	30242	12	183	4	2.185792	1.080886	472|5290|5291|5293	ATM|PIK3CA|PIK3CB|PIK3CD	-4.44918	0	20	0	0	-6.57703	70.82435597
1	-5.32939	GO:0052742	M1	1	0	GO Molecular Functions	21	phosphatidylinositol kinase activity	-5.32939	34.79091	11.49593	30242	19	183	4	2.185792	1.080886	472|5290|5291|5293	ATM|PIK3CA|PIK3CB|PIK3CD	-3.68295	0	20	0	0	-6.57703	70.82435597
1	-5.12529	GO:0035005	M1	1	0	GO Molecular Functions	21	1-phosphatidylinositol-4-phosphate 3-kinase activity	-5.12529	70.82436	14.41575	30242	7	183	3	1.639344	0.938686	5290|5291|5293	PIK3CA|PIK3CB|PIK3CD	-3.49451	0	20	0	0	-6.57703	70.82435597
1	-5.12529	GO:0046934	M1	1	0	GO Molecular Functions	21	phosphatidylinositol-4,5-bisphosphate 3-kinase activity	-5.12529	70.82436	14.41575	30242	7	183	3	1.639344	0.938686	5290|5291|5293	PIK3CA|PIK3CB|PIK3CD	-3.49451	0	20	0	0	-6.57703	70.82435597
1	-4.74896	GO:0052812	M1	1	0	GO Molecular Functions	21	phosphatidylinositol-3,4-bisphosphate 5-kinase activity	-4.74896	55.08561	12.6619	30242	9	183	3	1.639344	0.938686	5290|5291|5293	PIK3CA|PIK3CB|PIK3CD	-3.16891	0	20	0	0	-6.57703	70.82435597
1	-4.596	GO:0052813	M1	1	0	GO Molecular Functions	21	phosphatidylinositol bisphosphate kinase activity	-4.596	49.57705	11.98765	30242	10	183	3	1.639344	0.938686	5290|5291|5293	PIK3CA|PIK3CB|PIK3CD	-3.02746	0	20	0	0	-6.57703	70.82435597
1	-3.93862	GO:0016307	M1	1	0	GO Molecular	21	phosphatidylinositol	-3.93862	30.98566	9.360942	30242	16	183	3	1.639344	0.938686	5290|5291|5293	PIK3CA|PIK3CB|PIK3	-2.42502	0	20	0	0	-6.57703	70.82435597

						Functions		phosphate kinase activity											CD							
1	-6.54799	GO:0019199	M1	1	0	GO Molecular Functions	21	transmembrane receptor protein kinase activity	-6.54799	10.18706	8.682308	30242	146	183	9	4.918033	1.598524	1956|2050|2263|3643|4914|4915|5159|5979|7046	EGFR|EPHB4|FGFR2|INSR|NTRK1|NTRK2|PDGFRB|RET|TGFBR1	-4.75062	0	21	1	1	-6.54799	10.40987909
1	-5.95103	GO:0004714	M1	1	0	GO Molecular Functions	21	transmembrane receptor protein tyrosine kinase activity	-5.95103	10.40988	8.291456	30242	127	183	8	4.371585	1.511428	1956|2050|2263|3643|4914|4915|5159|5979	EGFR|EPHB4|FGFR2|INSR|NTRK1|NTRK2|PDGFRB|RET	-4.23566	0	21	0	0	-6.54799	10.40987909
1	-5.60904	GO:0004713	M1	1	0	GO Molecular Functions	21	protein tyrosine kinase activity	-5.60904	9.376274	7.778688	30242	141	183	8	4.371585	1.511428	1956|2050|2263|3643|4914|4915|5159|5979	EGFR|EPHB4|FGFR2|INSR|NTRK1|NTRK2|PDGFRB|RET	-3.91195	0	21	0	0	-6.54799	10.40987909
1	-5.58608	GO:0019838	M1	1	0	GO Molecular Functions	21	growth factor binding	-5.58608	9.310244	7.744815	30242	142	183	8	4.371585	1.511428	1956|2263|3643|4914|4915|5159|6331|7046	EGFR|FGFR2|INSR|NTRK1|NTRK2|PDGFRB|SCN5A|TGFBR1	-3.89345	0	21	0	0	-6.54799	10.40987909
1	-6.0689	GO:0019900	M1	1	0	GO Molecular Functions	21	kinase binding	-6.0689	3.945123	6.390029	30242	754	183	18	9.836066	2.201413	150|595|834|890|1636|1956|2099|2908|2915|3551|4914|5159|5970|6197|6331|6513|7157|59341	ADRA2A|CCND1|CASP1|CCNA2|ACE|EGFR|ESR1|NR3C1|GRM5|IKBKB|NTRK1|PDGFRB|RELA|RPS6KA3|SCN5A|SLC2A1|TP53|TRPV4	-4.327	0	22	1	1	-6.0689	3.945123277
1	-4.80296	GO:0019901	M1	1	0	GO Molecular Functions	21	protein kinase binding	-4.80296	3.694266	5.506873	30242	671	183	15	8.196721	2.027793	150|595|890|1636|1956|2099|2908|2915|3551|5159|5970|6197|6331|7157|59341	ADRA2A|CCND1|CCNA2|ACE|EGFR|ESR1|NR3C1|GRM5|IKBKB|PDGFRB|RELA|RPS6KA3|SCN5A|TP53|TRPV4	-3.19883	0	22	0	0	-6.0689	3.945123277
1	-6.06147	GO:0051380	M1	1	0	GO Molecular Functions	21	norepinephrine binding	-6.06147	123.9426	19.18635	30242	4	183	3	1.639344	0.938686	150|154|1815	ADRA2A|ADRB2|DRD4	-4.327	0	23	1	1	-6.06147	123.942623
1	-3.56522	GO:0016247	M1	1	0	GO Molecular Functions	21	channel regulator activity	-3.56522	6.791377	5.473186	30242	146	183	6	3.278689	1.316393	154|596|1080|1815|4842|7442	ADRB2|BCL2|CFTR|DRD4|NOS1|TRPV1	-2.08548	0	23	0	0	-6.06147	123.942623
1	-3.10534	GO:0005217	M1	1	0	GO Molecular Functions	21	intracellular ligand-gated ion channel activity	-3.10534	16.52568	6.638317	30242	30	183	3	1.639344	0.938686	1080|7442|8989	CFTR|TRPV1|TRPA1	-1.68155	0	23	0	0	-6.06147	123.942623
1	-2.78049	GO:0099106	M1	1	0	GO Molecular Functions	21	ion channel regulator activity	-2.78049	5.90203	4.536058	30242	140	183	5	2.73224	1.205087	154|1080|1815|4842|7442	ADRB2|CFTR|DRD4|NOS1|TRPV1	-1.37539	0	23	0	0	-6.06147	123.942623
1	-6.0295	GO:0042803	M1	1	0	GO Molecular Functions	21	protein homodimerization activity	-6.0295	4.131421	6.444145	30242	680	183	17	9.289617	2.145862	150|152|154|596|2263|3551|3757|4843|4914|4915|5138|5444|5743|5970|6793|7298|9429	ADRA2A|ADRA2C|ADRB2|BCL2|FGFR2|IKBKB|KCNH2|NOS2|NTRK1|NTRK2|PDE2A|PON1|PTGS2|RELA|STK10|TYMS|ABCG2	-4.30943	0	24	1	1	-6.0295	4.131420765
1	-5.42999	GO:0099095	M1	1	0	GO Molecular Functions	21	ligand-gated anion channel activity	-5.42999	36.72374	11.82915	30242	18	183	4	2.185792	1.080886	1080|2554|2558|2566	CFTR|GABRA1|GABRA5|GABRG2	-3.75046	0	25	1	1	-5.42999	45.07004471
1	-5.01826	GO:0099529	M1	1	0	GO Molecular Functions	21	neurotransmitter receptor activity involved in regulation of postsynaptic membrane potential	-5.01826	17.58051	8.875969	30242	47	183	5	2.73224	1.205087	153|1128|2554|2558|2566	ADRB1|CHRM1|GABRA1|GABRA5|GABRG2	-3.39887	0	25	0	0	-5.42999	45.07004471
1	-4.45963	GO:0008503	M1	1	0	GO Molecular Functions	21	benzodiazepine receptor activity	-4.45963	45.07004	11.40644	30242	11	183	3	1.639344	0.938686	2554|2558|2566	GABRA1|GABRA5|GABRG2	-2.90611	0	25	0	0	-5.42999	45.07004471

1	-4.22463	GO:0022851	M1	1	0	GO Molecular Functions	21	GABA-gated chloride ion channel activity	-4.22463	38.13619	10.44945	30242	13	183	3	1.639344	0.938686	2554|2558|2566	GABRA1|GABRA5|GABRG2	-2.6869	0	25	0	0	-5.42999	45.07004471
1	-4.02686	GO:0005237	M1	1	0	GO Molecular Functions	21	inhibitory extracellular ligand-gated ion channel activity	-4.02686	33.05137	9.68793	30242	15	183	3	1.639344	0.938686	2554|2558|2566	GABRA1|GABRA5|GABRG2	-2.50736	0	25	0	0	-5.42999	45.07004471
1	-3.70629	GO:0004890	M1	1	0	GO Molecular Functions	21	GABA-A receptor activity	-3.70629	26.09318	8.536899	30242	19	183	3	1.639344	0.938686	2554|2558|2566	GABRA1|GABRA5|GABRG2	-2.20995	0	25	0	0	-5.42999	45.07004471
1	-3.5109	GO:0016917	M1	1	0	GO Molecular Functions	21	GABA receptor activity	-3.5109	22.53502	7.883985	30242	22	183	3	1.639344	0.938686	2554|2558|2566	GABRA1|GABRA5|GABRG2	-2.03923	0	25	0	0	-5.42999	45.07004471
1	-2.96938	GO:0005254	M1	1	0	GO Molecular Functions	21	chloride channel activity	-2.96938	8.932802	5.330962	30242	74	183	4	2.185792	1.080886	1080|2554|2558|2566	CFTR|GABRA1|GABRA5|GABRG2	-1.55737	0	25	0	0	-5.42999	45.07004471
1	-2.70925	GO:0005253	M1	1	0	GO Molecular Functions	21	anion channel activity	-2.70925	7.598015	4.808722	30242	87	183	4	2.185792	1.080886	1080|2554|2558|2566	CFTR|GABRA1|GABRA5|GABRG2	-1.31319	0	25	0	0	-5.42999	45.07004471
1	-2.6188	GO:1904315	M1	1	0	GO Molecular Functions	21	transmitter-gated ion channel activity involved in regulation of postsynaptic membrane potential	-2.6188	11.26751	5.317883	30242	44	183	3	1.639344	0.938686	2554|2558|2566	GABRA1|GABRA5|GABRG2	-1.23161	0	25	0	0	-5.42999	45.07004471
1	-5.36639	GO:0004969	M1	1	0	GO Molecular Functions	21	histamine receptor activity	-5.36639	82.62842	15.60239	30242	6	183	3	1.639344	0.938686	3362|11255|59340	HTR6|HRH3|HRH4	-3.70754	0	26	1	1	-5.36639	82.6284153
1	-5.36609	GO:0015081	M1	1	0	GO Molecular Functions	21	sodium ion transmembrane transporter activity	-5.36609	8.697728	7.423515	30242	152	183	8	4.371585	1.511428	6326|6331|6335|6336|6523|6530|6531|6532	SCN2A|SCN5A|SCN9A|SCN10A|SLC5A1|SLC6A2|SLC6A3|SLC6A4	-3.70754	0	27	1	1	-5.36609	27.5428051
1	-4.90168	GO:0005248	M1	1	0	GO Molecular Functions	21	voltage-gated sodium channel activity	-4.90168	27.54281	10.14976	30242	24	183	4	2.185792	1.080886	6326|6331|6335|6336	SCN2A|SCN5A|SCN9A|SCN10A	-3.29337	0	27	0	0	-5.36609	27.5428051
1	-4.26166	GO:0097110	M1	1	0	GO Molecular Functions	21	scaffold protein binding	-4.26166	12.3326	7.245688	30242	67	183	5	2.73224	1.205087	673|3551|3757|4842|6331	BRAF|IKBKB|KCNH2|NOS1|SCN5A	-2.72082	0	27	0	0	-5.36609	27.5428051
1	-3.76048	GO:0005272	M1	1	0	GO Molecular Functions	21	sodium channel activity	-3.76048	14.37016	7.080715	30242	46	183	4	2.185792	1.080886	6326|6331|6335|6336	SCN2A|SCN5A|SCN9A|SCN10A	-2.25847	0	27	0	0	-5.36609	27.5428051
1	-2.05432	GO:0044325	M1	1	0	GO Molecular Functions	21	transmembrane transporter binding	-2.05432	4.97013	3.580295	30242	133	183	4	2.185792	1.080886	4842|5142|6331|6336	NOS1|PDE4B|SCN5A|SCN10A	-0.70287	0	27	0	0	-5.36609	27.5428051
1	-5.34499	GO:0015103	M1	1	0	GO Molecular Functions	21	inorganic anion transmembrane transporter activity	-5.34499	8.64088	7.393015	30242	153	183	8	4.371585	1.511428	1080|2554|2558|2566|4363|6530|6531|6532	CFTR|GABRA1|GABRA5|GABRG2|ABCC1|SLC6A2|SLC6A3|SLC6A4	-3.69052	0	28	1	1	-5.34499	55.0856102
1	-4.74896	GO:0008504	M1	1	0	GO Molecular Functions	21	monoamine transmembrane transporter activity	-4.74896	55.08561	12.6619	30242	9	183	3	1.639344	0.938686	6530|6531|6532	SLC6A2|SLC6A3|SLC6A4	-3.16891	0	28	0	0	-5.34499	55.0856102
1	-4.60631	GO:0008509	M1	1	0	GO Molecular Functions	21	anion transmembrane transporter activity	-4.60631	5.229647	5.897387	30242	316	183	10	5.464481	1.680144	1080|2554|2558|2566|4363|6513|6530|6531|6532|9429	CFTR|GABRA1|GABRA5|GABRG2|ABCC1|SLC2A1|SLC6A2|SLC6A3|SLC6A4|ABCG2	-3.03305	0	28	0	0	-5.34499	55.0856102
1	-4.45963	GO:0015378	M1	1	0	GO Molecular Functions	21	sodium:chloride symporter activity	-4.45963	45.07004	11.40644	30242	11	183	3	1.639344	0.938686	6530|6531|6532	SLC6A2|SLC6A3|SLC6A4	-2.90611	0	28	0	0	-5.34499	55.0856102
1	-4.12183	GO:0015373	M1	1	0	GO Molecular Functions	21	anion:sodium symporter activity	-4.12183	35.41218	10.04865	30242	14	183	3	1.639344	0.938686	6530|6531|6532	SLC6A2|SLC6A3|SLC6A4	-2.59331	0	28	0	0	-5.34499	55.0856102
1	-3.85624	GO:0015377	M1	1	0	GO Molecular Functions	21	cation:chloride symporter activity	-3.85624	29.16297	9.062669	30242	17	183	3	1.639344	0.938686	6530|6531|6532	SLC6A2|SLC6A3|SLC6A4	-2.34847	0	28	0	0	-5.34499	55.0856102
1	-3.70629	GO:0005326	M1	1	0	GO Molecular	21	neurotransmitter	-3.70629	26.09318	8.536899	30242	19	183	3	1.639344	0.938686	6530|6531|6532	SLC6A2|SLC6A3|SLC	-2.20995	0	28	0	0	-5.34499	55.0856102

						Functions		transmembrane transporter activity											6A4							
1	-3.52087	GO:1901618	M1	1	0	GO Molecular Functions	21	organic hydroxy compound transmembrane transporter activity	-3.52087	12.47221	6.522246	30242	53	183	4	2.185792	1.080886	6523|6530|6531|6532	SLC5A1|SLC6A2|SLC6A3|SLC6A4	-2.04384	0	28	0	0	-5.34499	55.0856102
1	-3.5109	GO:0015296	M1	1	0	GO Molecular Functions	21	anion:cation symporter activity	-3.5109	22.53502	7.883985	30242	22	183	3	1.639344	0.938686	6530|6531|6532	SLC6A2|SLC6A3|SLC6A4	-2.03923	0	28	0	0	-5.34499	55.0856102
1	-2.99148	GO:0015370	M1	1	0	GO Molecular Functions	21	solute:sodium symporter activity	-2.99148	9.055169	5.376405	30242	73	183	4	2.185792	1.080886	6523|6530|6531|6532	SLC5A1|SLC6A2|SLC6A3|SLC6A4	-1.57244	0	28	0	0	-5.34499	55.0856102
1	-2.45708	GO:0022804	M1	1	0	GO Molecular Functions	21	active transmembrane transporter activity	-2.45708	3.248292	3.56306	30242	407	183	8	4.371585	1.511428	1080|4363|5243|6523|6530|6531|6532|9429	CFTR|ABCC1|ABCB1|SLC5A1|SLC6A2|SLC6A3|SLC6A4|ABCG2	-1.08498	0	28	0	0	-5.34499	55.0856102
1	-2.42864	GO:0015294	M1	1	0	GO Molecular Functions	21	solute:cation symporter activity	-2.42864	6.356032	4.269126	30242	104	183	4	2.185792	1.080886	6523|6530|6531|6532	SLC5A1|SLC6A2|SLC6A3|SLC6A4	-1.05865	0	28	0	0	-5.34499	55.0856102
1	-2.22359	GO:0022853	M1	1	0	GO Molecular Functions	21	active ion transmembrane transporter activity	-2.22359	3.69978	3.463855	30242	268	183	6	3.278689	1.316393	1080|4363|6523|6530|6531|6532	CFTR|ABCC1|SLC5A1|SLC6A2|SLC6A3|SLC6A4	-0.8578	0	28	0	0	-5.34499	55.0856102
1	-5.18261	GO:0005126	M1	1	0	GO Molecular Functions	21	cytokine receptor binding	-5.18261	6.098038	6.577691	30242	271	183	10	5.464481	1.680144	836|3558|3569|3576|4914|6347|7040|7046|7124|729230	CASP3|IL2|IL6|CXCL8|NTRK1|CCL2|TGFB1|TGFBR1|TNF|CCR2	-3.54407	0	29	1	1	-5.18261	6.885701275
1	-3.22365	GO:0005125	M1	1	0	GO Molecular Functions	21	cytokine activity	-3.22365	4.922544	4.710064	30242	235	183	7	3.825137	1.417845	3558|3569|3576|6347|7040|7124|10135	IL2|IL6|CXCL8|CCL2|TGFB1|TNF|NAMPT	-1.79265	0	29	0	0	-5.18261	6.885701275
1	-2.73824	GO:0030545	M1	1	0	GO Molecular Functions	21	signaling receptor regulator activity	-2.73824	3.043404	3.749036	30242	543	183	10	5.464481	1.680144	351|2100|2147|3558|3569|3576|6347|7040|7124|10135	APP|ESR2|F2|IL2|IL6|CXCL8|CCL2|TGFB1|TNF|NAMPT	-1.33769	0	29	0	0	-5.18261	6.885701275
1	-2.47758	GO:0030546	M1	1	0	GO Molecular Functions	21	signaling receptor activator activity	-2.47758	3.00467	3.508813	30242	495	183	9	4.918033	1.598524	351|2147|3558|3569|3576|6347|7040|7124|10135	APP|F2|IL2|IL6|CXCL8|CCL2|TGFB1|TNF|NAMPT	-1.09907	0	29	0	0	-5.18261	6.885701275
1	-2.01885	GO:0042379	M1	1	0	GO Molecular Functions	21	chemokine receptor binding	-2.01885	6.885701	3.901334	30242	72	183	3	1.639344	0.938686	3576|6347|729230	CXCL8|CCL2|CCR2	-0.67145	0	29	0	0	-5.18261	6.885701275
1	-2.00386	GO:0048018	M1	1	0	GO Molecular Functions	21	receptor ligand activity	-2.00386	2.714691	2.976506	30242	487	183	8	4.371585	1.511428	2147|3558|3569|3576|6347|7040|7124|10135	F2|IL2|IL6|CXCL8|CCL2|TGFB1|TNF|NAMPT	-0.65912	0	29	0	0	-5.18261	6.885701275
1	-4.78357	GO:0004175	M1	1	0	GO Molecular Functions	21	endopeptidase activity	-4.78357	4.517271	5.792212	30242	439	183	12	6.557377	1.829835	834|836|1636|1991|2147|2155|4312|4313|4314|4318|4325|23621	CASP1|CASP3|ACE|ELANE|F2|F7|MMP1|MMP2|MMP3|MMP9|MMP16|BACE1	-3.18607	0	30	1	1	-4.78357	7.248106605
1	-4.59404	GO:0008236	M1	1	0	GO Molecular Functions	21	serine-type peptidase activity	-4.59404	6.814715	6.339541	30242	194	183	8	4.371585	1.511428	1636|1991|2147|2155|4312|4313|4314|4318	ACE|ELANE|F2|F7|MMP1|MMP2|MMP3|MMP9	-3.02746	0	30	0	0	-4.78357	7.248106605
1	-4.53073	GO:0017171	M1	1	0	GO Molecular Functions	21	serine hydrolase activity	-4.53073	6.677044	6.253344	30242	198	183	8	4.371585	1.511428	1636|1991|2147|2155|4312|4313|4314|4318	ACE|ELANE|F2|F7|MMP1|MMP2|MMP3|MMP9	-2.96744	0	30	0	0	-4.78357	7.248106605
1	-3.97124	GO:0004252	M1	1	0	GO Molecular Functions	21	serine-type endopeptidase activity	-3.97124	6.535581	5.763092	30242	177	183	7	3.825137	1.417845	1991|2147|2155|4312|4313|4314|4318	ELANE|F2|F7|MMP1|MMP2|MMP3|MMP9	-2.4547	0	30	0	0	-4.78357	7.248106605
1	-3.29521	GO:0008233	M1	1	0	GO Molecular Functions	21	peptidase activity	-3.29521	3.132831	4.231387	30242	633	183	12	6.557377	1.829835	834|836|1636|1991|2147|2155|4312|4313|4314|4318|4325|23621	CASP1|CASP3|ACE|ELANE|F2|F7|MMP1|MMP2|MMP3|MMP9|MMP16|BACE1	-1.85283	0	30	0	0	-4.78357	7.248106605
1	-3.17915	GO:0004222	M1	1	0	GO Molecular	21	metalloendopeptidase	-3.17915	7.248107	5.214973	30242	114	183	5	2.73224	1.205087	4312|4313|4314|4318|4	MMP1|MMP2|MMP3|	-1.75057	0	30	0	0	-4.78357	7.248106605

						Functions		activity										325	MMP9|MMP16							
1	-2.88256	GO:0008237	M1	1	0	GO Molecular Functions	21	metallopeptidase activity	-2.88256	5.033203	4.431323	30242	197	183	6	3.278689	1.316393	1636|4312|4313|4314|4318|4325	ACE|MMP1|MMP2|MMP3|MMP9|MMP16	-1.47286	0	30	0	0	-4.78357	7.248106605
1	-4.44459	GO:0042910	M1	1	0	GO Molecular Functions	21	xenobiotic transmembrane transporter activity	-4.44459	21.32346	8.83349	30242	31	183	4	2.185792	1.080886	4363|5243|6513|9429	ABCC1|ABCB1|SLC2A1|ABCG2	-2.89427	0	31	1	1	-4.44459	35.41217799
1	-4.12183	GO:0015562	M1	1	0	GO Molecular Functions	21	efflux transmembrane transporter activity	-4.12183	35.41218	10.04865	30242	14	183	3	1.639344	0.938686	4363|5243|9429	ABCC1|ABCB1|ABCG2	-2.59331	0	31	0	0	-4.44459	35.41217799
1	-4.02686	GO:0008559	M1	1	0	GO Molecular Functions	21	ABC-type xenobiotic transporter activity	-4.02686	33.05137	9.68793	30242	15	183	3	1.639344	0.938686	4363|5243|9429	ABCC1|ABCB1|ABCG2	-2.50736	0	31	0	0	-4.44459	35.41217799
1	-3.61905	GO:0140359	M1	1	0	GO Molecular Functions	21	ABC-type transporter activity	-3.61905	13.22055	6.74786	30242	50	183	4	2.185792	1.080886	1080|4363|5243|9429	CFTR|ABCC1|ABCB1|ABCG2	-2.13659	0	31	0	0	-4.44459	35.41217799
1	-3.02224	GO:0090482	M1	1	0	GO Molecular Functions	21	vitamin transmembrane transporter activity	-3.02224	15.49283	6.400135	30242	32	183	3	1.639344	0.938686	4363|6513|9429	ABCC1|SLC2A1|ABCG2	-1.60084	0	31	0	0	-4.44459	35.41217799
1	-2.35601	GO:0042626	M1	1	0	GO Molecular Functions	21	ATPase-coupled transmembrane transporter activity	-2.35601	6.064471	4.132972	30242	109	183	4	2.185792	1.080886	1080|4363|5243|9429	CFTR|ABCC1|ABCB1|ABCG2	-0.98812	0	31	0	0	-4.44459	35.41217799
1	-4.18252	GO:0042805	M1	1	0	GO Molecular Functions	21	actinin binding	-4.18252	18.36187	8.132751	30242	36	183	4	2.185792	1.080886	135|775|5468|5970	ADORA2A|CACNA1C|PPARG|RELA	-2.64789	0	32	1	1	-4.18252	18.36187007
1	-3.24195	GO:0051393	M1	1	0	GO Molecular Functions	21	alpha-actinin binding	-3.24195	18.36187	7.04212	30242	27	183	3	1.639344	0.938686	135|775|5468	ADORA2A|CACNA1C|PPARG	-1.80851	0	32	0	0	-4.18252	18.36187007
1	-4.02686	GO:0004953	M1	1	0	GO Molecular Functions	21	icosanoid receptor activity	-4.02686	33.05137	9.68793	30242	15	183	3	1.639344	0.938686	5468|5734|10800	PPARG|PTGER4|CYSLTR1	-2.50736	0	33	1	1	-4.02686	33.05136612
1	-2.21721	GO:0043621	M1	1	0	GO Molecular Functions	21	protein self-association	-2.21721	8.127385	4.347751	30242	61	183	3	1.639344	0.938686	5468|6513|7157	PPARG|SLC2A1|TP53	-0.8535	0	33	0	0	-4.02686	33.05136612
1	-3.66357	GO:0019955	M1	1	0	GO Molecular Functions	21	cytokine binding	-3.66357	7.082436	5.628339	30242	140	183	6	3.278689	1.316393	1230|1991|3577|7046|7132|729230	CCR1|ELANE|CXCR1|TGFBR1|TNFRSF1A|CCR2	-2.17284	0	34	1	1	-3.66357	21.55523877
1	-3.45213	GO:0016493	M1	1	0	GO Molecular Functions	21	C-C chemokine receptor activity	-3.45213	21.55524	7.694542	30242	23	183	3	1.639344	0.938686	1230|3577|729230	CCR1|CXCR1|CCR2	-1.99099	0	34	0	0	-3.66357	21.55523877
1	-3.39607	GO:0019957	M1	1	0	GO Molecular Functions	21	C-C chemokine binding	-3.39607	20.6571	7.516725	30242	24	183	3	1.639344	0.938686	1230|3577|729230	CCR1|CXCR1|CCR2	-1.9401	0	34	0	0	-3.66357	21.55523877
1	-3.29117	GO:0001637	M1	1	0	GO Molecular Functions	21	G protein-coupled chemoattractant receptor activity	-3.29117	19.0681	7.191458	30242	26	183	3	1.639344	0.938686	1230|3577|729230	CCR1|CXCR1|CCR2	-1.85283	0	34	0	0	-3.66357	21.55523877
1	-3.29117	GO:0004950	M1	1	0	GO Molecular Functions	21	chemokine receptor activity	-3.29117	19.0681	7.191458	30242	26	183	3	1.639344	0.938686	1230|3577|729230	CCR1|CXCR1|CCR2	-1.85283	0	34	0	0	-3.66357	21.55523877
1	-2.98278	GO:0019956	M1	1	0	GO Molecular Functions	21	chemokine binding	-2.98278	15.02335	6.288932	30242	33	183	3	1.639344	0.938686	1230|3577|729230	CCR1|CXCR1|CCR2	-1.56843	0	34	0	0	-3.66357	21.55523877
1	-3.45213	GO:0004114	M1	1	0	GO Molecular Functions	21	3',5'-cyclic-nucleotide phosphodiesterase activity	-3.45213	21.55524	7.694542	30242	23	183	3	1.639344	0.938686	5138|5142|8654	PDE2A|PDE4B|PDE5A	-1.99099	0	35	1	1	-3.45213	21.55523877
1	-3.34249	GO:0004112	M1	1	0	GO Molecular Functions	21	cyclic-nucleotide phosphodiesterase activity	-3.34249	19.83082	7.349367	30242	25	183	3	1.639344	0.938686	5138|5142|8654	PDE2A|PDE4B|PDE5A	-1.88908	0	35	0	0	-3.45213	21.55523877
1	-2.80314	GO:0030551	M1	1	0	GO Molecular Functions	21	cyclic nucleotide binding	-2.80314	13.04659	5.797758	30242	38	183	3	1.639344	0.938686	5138|5142|8654	PDE2A|PDE4B|PDE5A	-1.39575	0	35	0	0	-3.45213	21.55523877
1	-3.39607	GO:0001965	M1	1	0	GO Molecular Functions	21	G-protein alpha-subunit binding	-3.39607	20.6571	7.516725	30242	24	183	3	1.639344	0.938686	2149|3357|4988	F2R|HTR2B|OPRM1	-1.9401	0	36	1	1	-3.39607	20.65710383
1	-3.31368	GO:0016627	M1	1	0	GO Molecular	21	oxidoreductase activity,	-3.31368	11.01712	6.060116	30242	60	183	4	2.185792	1.080886	1645|1723|5498|6916	AKR1C1|DHODH|PP	-1.86535	0	37	1	1	-3.31368	11.01712204

<table>
<tr><td></td><td></td><td></td><td></td><td></td><td></td><td>Functions</td><td></td><td>acting on the CH-CH group of donors</td><td></td><td></td><td></td><td></td><td></td><td></td><td></td><td></td><td></td><td></td><td>OX|TBXAS1</td><td></td><td></td><td></td><td></td><td></td><td></td><td></td></tr>
<tr><td>1</td><td>-3.2497</td><td>GO:0047485</td><td>M1</td><td>1</td><td>0</td><td>GO Molecular Functions</td><td>21</td><td>protein N-terminus binding</td><td>-3.2497</td><td>7.511674</td><td>5.338403</td><td>30242</td><td>110</td><td>183</td><td>5</td><td>2.73224</td><td>1.205087</td><td>472|4221|5970|6531|7157</td><td>ATM|MEN1|RELA|SLC6A3|TP53</td><td>-1.81382</td><td>0</td><td>38</td><td>1</td><td>1</td><td>-3.2497</td><td>11.52954632</td></tr>
<tr><td>1</td><td>-2.64755</td><td>GO:0001046</td><td>M1</td><td>1</td><td>0</td><td>GO Molecular Functions</td><td>21</td><td>core promoter sequence-specific DNA binding</td><td>-2.64755</td><td>11.52955</td><td>5.391182</td><td>30242</td><td>43</td><td>183</td><td>3</td><td>1.639344</td><td>0.938686</td><td>2908|5970|7157</td><td>NR3C1|RELA|TP53</td><td>-1.25815</td><td>0</td><td>38</td><td>0</td><td>0</td><td>-3.2497</td><td>11.52954632</td></tr>
<tr><td>1</td><td>-2.12337</td><td>GO:0042826</td><td>M1</td><td>1</td><td>0</td><td>GO Molecular Functions</td><td>21</td><td>histone deacetylase binding</td><td>-2.12337</td><td>5.20494</td><td>3.705156</td><td>30242</td><td>127</td><td>183</td><td>4</td><td>2.185792</td><td>1.080886</td><td>595|3066|5970|7157</td><td>CCND1|HDAC2|RELA|TP53</td><td>-0.76173</td><td>0</td><td>38</td><td>0</td><td>0</td><td>-3.2497</td><td>11.52954632</td></tr>
<tr><td>1</td><td>-2.98337</td><td>GO:0009055</td><td>M1</td><td>1</td><td>0</td><td>GO Molecular Functions</td><td>21</td><td>electron transfer activity</td><td>-2.98337</td><td>6.557811</td><td>4.877831</td><td>30242</td><td>126</td><td>183</td><td>5</td><td>2.73224</td><td>1.205087</td><td>217|1544|1588|2936|4129</td><td>ALDH2|CYP1A2|CYP19A1|GSR|MAOB</td><td>-1.56843</td><td>0</td><td>39</td><td>1</td><td>1</td><td>-2.98337</td><td>6.557810738</td></tr>
<tr><td>1</td><td>-2.70925</td><td>GO:0016209</td><td>M1</td><td>1</td><td>0</td><td>GO Molecular Functions</td><td>21</td><td>antioxidant activity</td><td>-2.70925</td><td>7.598015</td><td>4.808722</td><td>30242</td><td>87</td><td>183</td><td>4</td><td>2.185792</td><td>1.080886</td><td>1728|2936|5742|5743</td><td>NQO1|GSR|PTGS1|PTGS2</td><td>-1.31319</td><td>0</td><td>40</td><td>1</td><td>1</td><td>-2.70925</td><td>7.5980152</td></tr>
<tr><td>1</td><td>-2.64755</td><td>GO:0016504</td><td>M1</td><td>1</td><td>0</td><td>GO Molecular Functions</td><td>21</td><td>peptidase activator activity</td><td>-2.64755</td><td>11.52955</td><td>5.391182</td><td>30242</td><td>43</td><td>183</td><td>3</td><td>1.639344</td><td>0.938686</td><td>351|834|2335</td><td>APP|CASP1|FN1</td><td>-1.25815</td><td>0</td><td>41</td><td>1</td><td>1</td><td>-2.64755</td><td>11.52954632</td></tr>
<tr><td>1</td><td>-2.5264</td><td>GO:0005539</td><td>M1</td><td>1</td><td>0</td><td>GO Molecular Functions</td><td>21</td><td>glycosaminoglycan binding</td><td>-2.5264</td><td>4.273884</td><td>3.905805</td><td>30242</td><td>232</td><td>183</td><td>6</td><td>3.278689</td><td>1.316393</td><td>351|1991|2147|2263|2335|114548</td><td>APP|ELANE|F2|FGFR2|FN1|NLRP3</td><td>-1.14357</td><td>0</td><td>41</td><td>0</td><td>0</td><td>-2.64755</td><td>11.52954632</td></tr>
<tr><td>1</td><td>-2.46014</td><td>GO:0008201</td><td>M1</td><td>1</td><td>0</td><td>GO Molecular Functions</td><td>21</td><td>heparin binding</td><td>-2.46014</td><td>4.977615</td><td>4.009619</td><td>30242</td><td>166</td><td>183</td><td>5</td><td>2.73224</td><td>1.205087</td><td>351|1991|2147|2263|2335</td><td>APP|ELANE|F2|FGFR2|FN1</td><td>-1.08585</td><td>0</td><td>41</td><td>0</td><td>0</td><td>-2.64755</td><td>11.52954632</td></tr>
<tr><td>1</td><td>-2.51721</td><td>GO:0019207</td><td>M1</td><td>1</td><td>0</td><td>GO Molecular Functions</td><td>21</td><td>kinase regulator activity</td><td>-2.51721</td><td>4.255541</td><td>3.892348</td><td>30242</td><td>233</td><td>183</td><td>6</td><td>3.278689</td><td>1.316393</td><td>595|836|890|2915|3558|5290</td><td>CCND1|CASP3|CCNA2|GRM5|IL2|PIK3CA</td><td>-1.13655</td><td>0</td><td>42</td><td>1</td><td>1</td><td>-2.51721</td><td>9.915409836</td></tr>
<tr><td>1</td><td>-2.46008</td><td>GO:0016538</td><td>M1</td><td>1</td><td>0</td><td>GO Molecular Functions</td><td>21</td><td>cyclin-dependent protein serine/threonine kinase regulator activity</td><td>-2.46008</td><td>9.91541</td><td>4.922851</td><td>30242</td><td>50</td><td>183</td><td>3</td><td>1.639344</td><td>0.938686</td><td>595|836|890</td><td>CCND1|CASP3|CCNA2</td><td>-1.08585</td><td>0</td><td>42</td><td>0</td><td>0</td><td>-2.51721</td><td>9.915409836</td></tr>
<tr><td>1</td><td>-2.08687</td><td>GO:0019887</td><td>M1</td><td>1</td><td>0</td><td>GO Molecular Functions</td><td>21</td><td>protein kinase regulator activity</td><td>-2.08687</td><td>4.050413</td><td>3.410943</td><td>30242</td><td>204</td><td>183</td><td>5</td><td>2.73224</td><td>1.205087</td><td>595|836|890|2915|5290</td><td>CCND1|CASP3|CCNA2|GRM5|PIK3CA</td><td>-0.72935</td><td>0</td><td>42</td><td>0</td><td>0</td><td>-2.51721</td><td>9.915409836</td></tr>
<tr><td>1</td><td>-2.09993</td><td>GO:0031072</td><td>M1</td><td>1</td><td>0</td><td>GO Molecular Functions</td><td>21</td><td>heat shock protein binding</td><td>-2.09993</td><td>5.124243</td><td>3.662675</td><td>30242</td><td>129</td><td>183</td><td>4</td><td>2.185792</td><td>1.080886</td><td>134|1543|2908|3066</td><td>ADORA1|CYP1A1|NR3C1|HDAC2</td><td>-0.74036</td><td>0</td><td>43</td><td>1</td><td>1</td><td>-2.09993</td><td>5.124242809</td></tr>
<tr><td>1</td><td>-2.06557</td><td>GO:0016810</td><td>M1</td><td>1</td><td>0</td><td>GO Molecular Functions</td><td>21</td><td>hydrolase activity, acting on carbon-nitrogen (but not peptide) bonds</td><td>-2.06557</td><td>5.007783</td><td>3.600577</td><td>30242</td><td>132</td><td>183</td><td>4</td><td>2.185792</td><td>1.080886</td><td>100|427|2166|3066</td><td>ADA|ASAH1|FAAH|HDAC2</td><td>-0.71009</td><td>0</td><td>44</td><td>1</td><td>1</td><td>-2.06557</td><td>6.7913766</td></tr>
<tr><td>1</td><td>-2.00253</td><td>GO:0016811</td><td>M1</td><td>1</td><td>0</td><td>GO Molecular Functions</td><td>21</td><td>hydrolase activity, acting on carbon-nitrogen (but not peptide) bonds, in linear amides</td><td>-2.00253</td><td>6.791377</td><td>3.865442</td><td>30242</td><td>73</td><td>183</td><td>3</td><td>1.639344</td><td>0.938686</td><td>427|2166|3066</td><td>ASAH1|FAAH|HDAC2</td><td>-0.65912</td><td>0</td><td>44</td><td>0</td><td>0</td><td>-2.06557</td><td>6.7913766</td></tr>
</table>
