# Supplementary material for: Molecular Mechanism of the Saposhnikovia divaricata–Angelica dahurica Herb Pair in Migraine Therapy Based on Network Pharmacology and Molecular Docking
Source: Evid Based Complement Alternat Med. 2022 Nov 26;2022:1994575. doi: 10.1155/2022/1994575 (PMC9722292; doi:10.1155/2022/1994575)
Supplement: Supplementary Materials — Table S1: 704 targets of SAHP. Table S2: 1086 targets of migraine. Table S3: 183 common targets of SAHP and migraine. Table S4: the result of GO functional enrichment analysis. Table S5: the result of KEGG pathway enrichment analysis. [file 1994575.f1.zip › Table S5.the result of KEGG pathway enrichment analysis.pdf]

<table>
<tr><th>_MEMBER_MyList</th><th>_LogP_MyList</th><th>GO</th><th>_PATTERN_</th><th>_RANK_</th><th>GiniIndex</th><th>Category</th><th>CategoryID</th><th>Description</th><th>LogP</th><th>Enrichment</th><th>Z-score</th><th>#TotalGeneInLibrary</th><th>#GeneInGO</th><th>#GeneInHitList</th><th>#GeneInGOAndHitList</th><th>%InGO</th><th>STDV %InGO</th><th>GeneID</th><th>Hits</th><th>Log(q-value)</th><th>EvidenceCutoff</th><th>GROUP_ID</th><th>FirstInGroupByEnrichment</th><th>FirstInGroupByLogP</th><th>BestLogPInGroup</th><th>BestEnrichmentInGroup</th></tr>
<tr><td>1</td><td>-69.3539</td><td>ko04080</td><td>M1</td><td>1</td><td>0</td><td>KEGG Pathway</td><td>24</td><td>Neuroactive ligand-receptor interaction</td><td>-69.3539</td><td>33.40932</td><td>42.2804</td><td>30242</td><td>277</td><td>183</td><td>56</td><td>30.60109</td><td>3.406585</td><td>134|135|136|140|146|147|148|150|151|152|153|154|185|624|1128|1129|1131|1132|1133|1268|1812|1813|1814|1815|1909|1910|2147|2149|2554|2558|2566|2891|2902|2903|2904|2908|2915|3061|3062|3350|3351|3356|3357|3358|3362|3363|4985|4986|4988|5024|5734|7442|10203|10800|11255|59340</td><td>ADORA1|ADORA2A|ADORA2B|ADORA3|ADRA1D|ADRA1B|ADRA1A|ADRA2A|ADRA2B|ADRA2C|ADRB1|ADRB2|AGTR1|BDKRB2|CHRM1|CHRM2|CHRM3|CHRM4|CHRM5|CNR1|DRD1|DRD2|DRD3|DRD4|EDNRA|EDNRB|F2|F2R|GABRA1|GABRA5|GABRG2|GRIA2|GRIN1|GRIN2A|GRIN2B|NR3C1|GRM5|HCRTR1|HCRTR2|HTR1A|HTR1B|HTR2A|HTR2B|HTR2C|HTR6|HTR7|OPRD1|OPRK1|OPRM1|P2RX3|PTGER4|TRPV1|CALCRL|CYSLTR1|HRH3|HRH4</td><td>-66.4546</td><td>0</td><td>1</td><td>1</td><td>1</td><td>-69.3539</td><td>33.40932</td></tr>
<tr><td>1</td><td>-63.2813</td><td>hsa04080</td><td>M1</td><td>1</td><td>0</td><td>KEGG Pathway</td><td>24</td><td>Neuroactive ligand-receptor interaction</td><td>-63.2813</td><td>25.32161</td><td>36.82837</td><td>30242</td><td>372</td><td>183</td><td>57</td><td>31.14754</td><td>3.423309</td><td>134|135|136|140|146|147|148|150|151|152|153|154|185|624|1128|1129|1131|1132|1133|1268|1812|1813|1814|1815|1909|1910|2147|2149|2554|2558|2566|2891|2902|2903|2904|2908|2915|3061|3062|3350|3351|3356|3357|3358|3362|3363|4985|4986|4988|5024|5734|7442|10203|10800|11255|59340|114548</td><td>ADORA1|ADORA2A|ADORA2B|ADORA3|ADRA1D|ADRA1B|ADRA1A|ADRA2A|ADRA2B|ADRA2C|ADRB1|ADRB2|AGTR1|BDKRB2|CHRM1|CHRM2|CHRM3|CHRM4|CHRM5|CNR1|DRD1|DRD2|DRD3|DRD4|EDNRA|EDNRB|F2|F2R|GABRA1|GABRA5|GABRG2|GRIA2|GRIN1|GRIN2A|GRIN2B|NR3C1|GRM5|HCRTR1|HCRTR2|HTR1A|HTR1B|HTR2A|HTR2B|HTR2C|HTR6|HTR7|OPRD1|OPRK1|OPRM1|P2RX3|PTGER4|TRPV1|CALCRL|CYSLTR1|HRH3|HRH4|NLRP3</td><td>-60.683</td><td>0</td><td>1</td><td>0</td><td>0</td><td>-69.3539</td><td>33.40932</td></tr>
<tr><td>1</td><td>-40.1967</td><td>ko04020</td><td>M1</td><td>1</td><td>0</td><td>KEGG Pathway</td><td>24</td><td>Calcium signaling pathway</td><td>-40.1967</td><td>30.87216</td><td>31.53872</td><td>30242</td><td>182</td><td>183</td><td>34</td><td>18.57923</td><td>2.87512</td><td>135|136|146|147|148|153|154|185|624|775|11</td><td>ADORA2A|ADORA2B|ADRA1D|ADRA1B</td><td>-37.7746</td><td>0</td><td>1</td><td>0</td><td>0</td><td>-69.3539</td><td>33.40932</td></tr>
</table>

																		28|1129|1131|1133|1812|1909|1910|1956|2149|2902|2903|2915|3356|3357|3358|3362|3363|4842|4843|4846|5024|5159|5582|10800	|ADRA1A|ADRB1|ADRB2|AGTR1|BDKRB2|CACNA1C|CHRM1|CHRM2|CHRM3|CHRM5|DRD1|EDNRA|EDNRB|EGFR|F2R|GRIN1|GRIN2A|GRM5|HTR2A|HTR2B|HTR2C|HTR6|HTR7|NOS1|NOS2|NOS3|P2RX3|PDGFRB|PRKCG|CYSLTR1							
1	-38.3175	hsa04020	M1	1	0	KEGG Pathway	24	Calcium signaling pathway	-38.3175	27.40845	29.60247	30242	205	183	34	18.57923	2.87512	135|136|146|147|148|153|154|185|624|775|1128|1129|1131|1133|1812|1909|1910|1956|2149|2902|2903|2915|3356|3357|3358|3362|3363|4842|4843|4846|5024|5159|5582|10800	ADORA2A|ADORA2B|ADRA1D|ADRA1B|ADRA1A|ADRB1|ADRB2|AGTR1|BDKRB2|CACNA1C|CHRM1|CHRM2|CHRM3|CHRM5|DRD1|EDNRA|EDNRB|EGFR|F2R|GRIN1|GRIN2A|GRM5|HTR2A|HTR2B|HTR2C|HTR6|HTR7|NOS1|NOS2|NOS3|P2RX3|PDGFRB|PRKCG|CYSLTR1	-36.0203	0	1	0	0	-69.3539	33.40932
1	-34.7817	hsa05200	M1	1	0	KEGG Pathway	24	Pathways in cancer	-34.7817	12.62379	21.97686	30242	576	183	44	24.04372	3.159054	185|367|595|596|624|673|836|1728|1909|1910|1956|2099|2100|2147|2149|2263|2335|3066|3551|3558|3569|3576|4312|4313|4318|4792|4843|4914|5159|5290|5291|5293|5467|5468|5582|5734|5743|5970|5979|6513|7040|7046|7157|9475	AGTR1|AR|CCND1|BCL2|BDKRB2|BRAF|CASP3|NQO1|EDNRA|EDNRB|EGFR|ESR1|ESR2|F2|F2R|FGFR2|FN1|HDAC2|IKBKB|IL2|IL6|CXCL8|MMP1|MMP2|MMP9|NFKBIA|NOS2|NTRK1|PDGFRB|PIK3CA|PIK3CB|PIK3CD|PPARD|PPARG|PRKCG|PTGER4|PTGS2|RELA|RET|SLC2A1|TGFB1|TGFBR1|TP53|ROCK2	-32.5813	0	2	1	1	-34.7817	12.62379
1	-29.4641	ko04024	M1	1	0	KEGG Pathway	24	cAMP signaling pathway	-29.4641	23.36965	24.64049	30242	198	183	28	15.30055	2.661143	134|135|153|154|673|775|1080|1128|1129|1812|1813|1909|2149|2891|2902|2903|2904|3350|3351|3362|4792|5142|5290|5291|5293|5465|5970|9475	ADORA1|ADORA2A|ADRB1|ADRB2|BRAF|CACNA1C|CFTR|CHRM1|CHRM2|DRD1|DRD2|EDNRA|F2R|GRIA2|GRIN1|GRIN2A|GRIN2B|HTR1A|HTR1B|HTR6|NFKBIA|PDE4B|PIK3CA|	-27.343	0	3	1	1	-29.4641	23.36965

																			PIK3CB|PIK3CD|PPARA|RELA|ROCK2							
1	-27.8037	hsa04024	M1	1	0	KEGG Pathway	24	cAMP signaling pathway	-27.8037	20.4743	22.92852	30242	226	183	28	15.30055	2.661143	134|135|153|154|673|775|1080|1128|1129|1812|1813|1909|2149|2891|2902|2903|2904|3350|3351|3362|4792|5142|5290|5291|5293|5465|5970|9475	ADORA1|ADORA2A|ADRB1|ADRB2|BRAF|CACNA1C|CFTR|CHRM1|CHRM2|DRD1|DRD2|EDNRA|F2R|GRIA2|GRIN1|GRIN2A|GRIN2B|HTR1A|HTR1B|HTR6|NFKBIA|PDE4B|PIK3CA|PIK3CB|PIK3CD|PPARA|RELA|ROCK2	-25.7495	0	3	0	0	-29.4641	23.36965
1	-22.7123	ko04933	M1	1	0	KEGG Pathway	24	AGE-RAGE signaling pathway in diabetic complications	-22.7123	31.71596	23.88497	30242	99	183	19	10.38251	2.254873	185|595|596|836|2335|3383|3569|3576|4313|4846|5290|5291|5293|5970|6347|6401|7040|7046|7124	AGTR1|CCND1|BCL2|CASP3|FN1|ICAM1|IL6|CXCL8|MMP2|NOS3|PIK3CA|PIK3CB|PIK3CD|RELA|CCL2|SELE|TGFB1|TGFBR1|TNF	-20.7626	0	4	1	1	-22.7123	31.71596
1	-22.0252	hsa04933	M1	1	0	KEGG Pathway	24	AGE-RAGE signaling pathway in diabetic complications	-22.0252	29.34467	22.91733	30242	107	183	19	10.38251	2.254873	185|595|596|836|2335|3383|3569|3576|4313|4846|5290|5291|5293|5970|6347|6401|7040|7046|7124	AGTR1|CCND1|BCL2|CASP3|FN1|ICAM1|IL6|CXCL8|MMP2|NOS3|PIK3CA|PIK3CB|PIK3CD|RELA|CCL2|SELE|TGFB1|TGFBR1|TNF	-20.1673	0	4	0	0	-22.7123	31.71596
1	-20.833	ko05142	M1	1	0	KEGG Pathway	24	Chagas disease (American trypanosomiasis)	-20.833	29.16297	22.23019	30242	102	183	18	9.836066	2.201413	624|1636|3551|3558|3569|3576|4792|4843|5290|5291|5293|5970|6347|7040|7046|7099|7124|7132	BDKRB2|ACE|IKBKB|IL2|IL6|CXCL8|NFKBIA|NOS2|PIK3CA|PIK3CB|PIK3CD|RELA|CCL2|TGFB1|TGFBR1|TLR4|TNF|TNFRSF1A	-19.0129	0	4	0	0	-22.7123	31.71596
1	-20.4818	hsa05161	M1	1	0	KEGG Pathway	24	Hepatitis B	-20.4818	19.60674	19.37148	30242	177	183	21	11.47541	2.356083	595|596|673|836|890|3551|3569|3576|4318|4792|5290|5291|5293|5582|5970|7040|7046|7099|7124|7157|29110	CCND1|BCL2|BRAF|CASP3|CCNA2|IKBKB|IL6|CXCL8|MMP9|NFKBIA|PIK3CA|PIK3CB|PIK3CD|PRKCG|RELA|TGFB1|TGFBR1|TLR4|TNF|TP53|TBK1	-18.6965	0	4	0	0	-22.7123	31.71596
1	-20.4359	hsa05142	M1	1	0	KEGG Pathway	24	Chagas disease (American trypanosomiasis)	-20.4359	27.80021	21.6686	30242	107	183	18	9.836066	2.201413	624|1636|3551|3558|3569|3576|4792|4843|5290|5291|5293|5970|6347|7040|7046|7099|7124|7132	BDKRB2|ACE|IKBKB|IL2|IL6|CXCL8|NFKBIA|NOS2|PIK3CA|PIK3CB|PIK3CD|RELA|CCL2|TGFB1|TGFBR1|TLR4|TNF|TNFRSF1A	-18.6827	0	4	0	0	-22.7123	31.71596
1	-18.8053	ko04668	M1	1	0	KEGG Pathway	24	TNF signaling pathway	-18.8053	26.01265	20.31793	30242	108	183	17	9.289617	2.145862	836|3383|3551|3569|4314|4318|4792|5290|5	CASP3|ICAM1|IKBKB|IL6|MMP3|MMP9|	-17.1101	0	4	0	0	-22.7123	31.71596

																		291|5293|5743|5970|6347|6401|7124|7132|8986	NFKBIA|PIK3CA|PIK3CB|PIK3CD|PTGS2|RELA|CCL2|SELE|TNF|TNFRSF1A|RPS6KA4								
1	-18.3205	hsa04668	M1	1	0	KEGG Pathway	24	TNF signaling pathway	-18.3205	24.42927	19.64111	30242	115	183	17	9.289617	2.145862	836|3383|3551|3569|4314|4318|4792|5290|5291|5293|5743|5970|6347|6401|7124|7132|8986	CASP3|ICAM1|IKBKB|IL6|MMP3|MMP9|NFKBIA|PIK3CA|PIK3CB|PIK3CD|PTGS2|RELA|CCL2|SELE|TNF|TNFRSF1A|RPS6KA4	-16.6516	0	4	0	0	-22.7123	31.71596
1	-18.1227	ko04071	M1	1	0	KEGG Pathway	24	Sphingolipid signaling pathway	-18.1227	23.80819	19.36921	30242	118	183	17	9.289617	2.145862	134|140|427|596|624|4363|4846|4985|5290|5291|5293|5582|5970|7124|7132|7157|9475	ADORA1|ADORA3|ASAH1|BCL2|BDKRB2|ABCC1|NOS3|OPRD1|PIK3CA|PIK3CB|PIK3CD|PRKCG|RELA|TNF|TNFRSF1A|TP53|ROCK2	-16.4787	0	4	0	0	-22.7123	31.71596
1	-17.6988	hsa05215	M1	1	0	KEGG Pathway	24	Prostate cancer	-17.6988	25.92264	19.67247	30242	102	183	16	8.743169	2.088053	367|595|596|673|1956|2263|3551|4314|4318|4792|5159|5290|5291|5293|5970|7157	AR|CCND1|BCL2|BRAF|EGFR|FGFR2|IKBKB|MMP3|MMP9|NFKBIA|PDGFRB|PIK3CA|PIK3CB|PIK3CD|RELA|TP53	-16.0783	0	4	0	0	-22.7123	31.71596
1	-17.5614	hsa04071	M1	1	0	KEGG Pathway	24	Sphingolipid signaling pathway	-17.5614	22.12099	18.61063	30242	127	183	17	9.289617	2.145862	134|140|427|596|624|4363|4846|4985|5290|5291|5293|5582|5970|7124|7132|7157|9475	ADORA1|ADORA3|ASAH1|BCL2|BDKRB2|ABCC1|NOS3|OPRD1|PIK3CA|PIK3CB|PIK3CD|PRKCG|RELA|TNF|TNFRSF1A|TP53|ROCK2	-15.9631	0	4	0	0	-22.7123	31.71596
1	-16.7175	ko05418	M1	1	0	KEGG Pathway	24	Fluid shear stress and atherosclerosis	-16.7175	19.78427	17.50619	30242	142	183	17	9.289617	2.145862	596|1728|3383|3551|4313|4318|4846|5290|5291|5293|5970|6347|6401|7124|7132|7157|59341	BCL2|NQO1|ICAM1|IKBKB|MMP2|MMP9|NOS3|PIK3CA|PIK3CB|PIK3CD|RELA|CCL2|SELE|TNF|TNFRSF1A|TP53|TRPV4	-15.1404	0	4	0	0	-22.7123	31.71596
1	-16.4072	hsa05418	M1	1	0	KEGG Pathway	24	Fluid shear stress and atherosclerosis	-16.4072	18.9822	17.11079	30242	148	183	17	9.289617	2.145862	596|1728|3383|3551|4313|4318|4846|5290|5291|5293|5970|6347|6401|7124|7132|7157|59341	BCL2|NQO1|ICAM1|IKBKB|MMP2|MMP9|NOS3|PIK3CA|PIK3CB|PIK3CD|RELA|CCL2|SELE|TNF|TNFRSF1A|TP53|TRPV4	-14.8504	0	4	0	0	-22.7123	31.71596
1	-15.7072	ko05215	M1	1	0	KEGG Pathway	24	Prostate cancer	-15.7072	26.59305	18.65256	30242	87	183	14	7.650273	1.964856	367|595|596|673|1956|2263|3551|4792|5159|5290|5291|5293|5970|7157	AR|CCND1|BCL2|BRAF|EGFR|FGFR2|IKBKB|NFKBIA|PDGFRB|PIK3CA|PIK3CB|PIK3CD|RELA|TP53	-14.1697	0	4	0	0	-22.7123	31.71596

1	-14.7785	hsa05205	M1	1	0	KEGG Pathway	24	Proteoglycans in cancer	-14.7785	13.64506	14.62013	30242	218	183	18	9.836066	2.201413	595|673|836|1956|2099|2335|3569|4313|4318|5290|5291|5293|5582|7040|7099|7124|7157|9475	CCND1|BRAF|CASP3|EGFR|ESR1|FN1|IL6|MMP2|MMP9|PIK3CA|PIK3CB|PIK3CD|PRKCG|TGFB1|TLR4|TNF|TP53|ROCK2	-13.3416	0	4	0	0	-22.7123	31.71596
1	-14.7218	hsa05166	M1	1	0	KEGG Pathway	24	HTLV-I infection	-14.7218	12.26516	14.12324	30242	256	183	19	10.38251	2.254873	472|595|3383|3551|3558|3569|3683|4792|5159|5290|5291|5293|5970|6513|7040|7046|7124|7132|7157	ATM|CCND1|ICAM1|IKBKB|IL2|IL6|ITGAL|NFKBIA|PDGFRB|PIK3CA|PIK3CB|PIK3CD|RELA|SLC2A1|TGFB1|TGFBR1|TNF|TNFRSF1A|TP53	-13.3277	0	4	0	0	-22.7123	31.71596
1	-14.7218	ko05166	M1	1	0	KEGG Pathway	24	HTLV-I infection	-14.7218	12.26516	14.12324	30242	256	183	19	10.38251	2.254873	472|595|3383|3551|3558|3569|3683|4792|5159|5290|5291|5293|5970|6513|7040|7046|7124|7132|7157	ATM|CCND1|ICAM1|IKBKB|IL2|IL6|ITGAL|NFKBIA|PDGFRB|PIK3CA|PIK3CB|PIK3CD|RELA|SLC2A1|TGFB1|TGFBR1|TNF|TNFRSF1A|TP53	-13.3277	0	4	0	0	-22.7123	31.71596
1	-14.4008	hsa05146	M1	1	0	KEGG Pathway	24	Amoebiasis	-14.4008	21.62239	16.67369	30242	107	183	14	7.650273	1.964856	836|1645|2335|3569|3576|4843|5290|5291|5293|5582|5970|7040|7099|7124	CASP3|AKR1C1|FN1|IL6|CXCL8|NOS2|PIK3CA|PIK3CB|PIK3CD|PRKCG|RELA|TGFB1|TLR4|TNF	-13.02	0	4	0	0	-22.7123	31.71596
1	-14.1215	hsa05160	M1	1	0	KEGG Pathway	24	Hepatitis C	-14.1215	15.64562	14.89701	30242	169	183	16	8.743169	2.088053	595|673|836|1956|3551|3576|4792|5290|5291|5293|5465|5970|7124|7132|7157|29110	CCND1|BRAF|CASP3|EGFR|IKBKB|CXCL8|NFKBIA|PIK3CA|PIK3CB|PIK3CD|PPARA|RELA|TNF|TNFRSF1A|TP53|TBK1	-12.7663	0	4	0	0	-22.7123	31.71596
1	-14.0831	ko05205	M1	1	0	KEGG Pathway	24	Proteoglycans in cancer	-14.0831	13.83924	14.32125	30242	203	183	17	9.289617	2.145862	595|673|836|1956|2099|2335|4313|4318|5290|5291|5293|5582|7040|7099|7124|7157|9475	CCND1|BRAF|CASP3|EGFR|ESR1|FN1|MMP2|MMP9|PIK3CA|PIK3CB|PIK3CD|PRKCG|TGFB1|TLR4|TNF|TP53|ROCK2	-12.7402	0	4	0	0	-22.7123	31.71596
1	-14.0613	hsa04931	M1	1	0	KEGG Pathway	24	insulin resistance	-14.0613	20.4743	16.18248	30242	113	183	14	7.650273	1.964856	3551|3569|3643|4792|4846|5290|5291|5293|5465|5970|6197|6513|7124|7132	IKBKB|IL6|INSR|NFKBIA|NOS3|PIK3CA|PIK3CB|PIK3CD|PPARA|RELA|RPS6KA3|SLC2A1|TNF|TNFRSF1A	-12.7302	0	4	0	0	-22.7123	31.71596
1	-13.8038	ko05220	M1	1	0	KEGG Pathway	24	Chronic myeloid leukemia	-13.8038	27.93073	17.72633	30242	71	183	12	6.557377	1.829835	595|673|3066|3551|4792|5290|5291|5293|5970|7040|7046|7157	CCND1|BRAF|HDAC2|IKBKB|NFKBIA|PIK3CA|PIK3CB|PIK3CD|RELA|TGFB1|TGFBR1|TP53	-12.5173	0	4	0	0	-22.7123	31.71596
1	-13.6072	ko05146	M1	1	0	KEGG Pathway	24	Amoebiasis	-13.6072	22.37853	16.36948	30242	96	183	13	7.103825	1.898976	836|2335|3569|3576|4843|5290|5291|5293|5582|5970|7040|7099|7	CASP3|FN1|IL6|CXCL8|NOS2|PIK3CA|PIK3CB|PIK3CD|PRKC	-12.3414	0	4	0	0	-22.7123	31.71596

																		124	G|RELA|TGFB1|TLR4|TNF							
1	-13.4286	hsa05222	M1	1	0	KEGG Pathway	24	Small cell lung cancer	-13.4286	21.70039	16.09679	30242	99	183	13	7.103825	1.898976	595|596|836|2335|3551|4792|4843|5290|5291|5293|5743|5970|7157	CCND1|BCL2|CASP3|FN1|IKBKB|NFKBIA|NOS2|PIK3CA|PIK3CB|PIK3CD|PTGS2|RELA|TP53	-12.2014	0	4	0	0	-22.7123	31.71596
1	-13.1516	ko05160	M1	1	0	KEGG Pathway	24	Hepatitis C	-13.1516	17.66104	14.91116	30242	131	183	14	7.650273	1.964856	673|1956|3551|3576|4792|5290|5291|5293|5465|5970|7124|7132|7157|29110	BRAF|EGFR|IKBKB|CXCL8|NFKBIA|PIK3CA|PIK3CB|PIK3CD|PPARA|RELA|TNF|TNFRSF1A|TP53|TBK1	-11.9435	0	4	0	0	-22.7123	31.71596
1	-13.0178	hsa05220	M1	1	0	KEGG Pathway	24	Chronic myeloid leukemia	-13.0178	24.18393	16.40267	30242	82	183	12	6.557377	1.829835	595|673|3066|3551|4792|5290|5291|5293|5970|7040|7046|7157	CCND1|BRAF|HDAC2|IKBKB|NFKBIA|PIK3CA|PIK3CB|PIK3CD|RELA|TGFB1|TGFBR1|TP53	-11.8261	0	4	0	0	-22.7123	31.71596
1	-12.8876	ko05222	M1	1	0	KEGG Pathway	24	Small cell lung cancer	-12.8876	23.60812	16.18971	30242	84	183	12	6.557377	1.829835	595|596|2335|3551|4792|4843|5290|5291|5293|5743|5970|7157	CCND1|BCL2|FN1|IKBKB|NFKBIA|NOS2|PIK3CA|PIK3CB|PIK3CD|PTGS2|RELA|TP53	-11.7044	0	4	0	0	-22.7123	31.71596
1	-12.8527	hsa04932	M1	1	0	KEGG Pathway	24	Non-alcoholic fatty liver disease (NAFLD)	-12.8527	14.66777	13.90237	30242	169	183	15	8.196721	2.027793	836|3551|3569|3576|3643|5290|5291|5293|5465|5742|5743|5970|7040|7124|7132	CASP3|IKBKB|IL6|CXCL8|INSR|PIK3CA|PIK3CB|PIK3CD|PPARA|PTGS1|PTGS2|RELA|TGFB1|TNF|TNFRSF1A	-11.6858	0	4	0	0	-22.7123	31.71596
1	-12.7811	ko05212	M1	1	0	KEGG Pathway	24	Pancreatic cancer	-12.7811	28.40352	17.1233	30242	64	183	11	6.010929	1.757049	595|673|1956|3551|5290|5291|5293|5970|7040|7046|7157	CCND1|BRAF|EGFR|IKBKB|PIK3CA|PIK3CB|PIK3CD|RELA|TGFB1|TGFBR1|TP53	-11.6222	0	4	0	0	-22.7123	31.71596
1	-12.1723	hsa01522	M1	1	0	KEGG Pathway	24	Endocrine resistance	-12.1723	20.6571	15.05139	30242	96	183	12	6.557377	1.829835	595|596|673|1956|2099|2100|4313|4318|5290|5291|5293|7157	CCND1|BCL2|BRAF|EGFR|ESR1|ESR2|MMP2|MMP9|PIK3CA|PIK3CB|PIK3CD|TP53	-11.0512	0	4	0	0	-22.7123	31.71596
1	-12.1723	ko01522	M1	1	0	KEGG Pathway	24	Endocrine resistance	-12.1723	20.6571	15.05139	30242	96	183	12	6.557377	1.829835	595|596|673|1956|2099|2100|4313|4318|5290|5291|5293|7157	CCND1|BCL2|BRAF|EGFR|ESR1|ESR2|MMP2|MMP9|PIK3CA|PIK3CB|PIK3CD|TP53	-11.0512	0	4	0	0	-22.7123	31.71596
1	-12.0185	hsa05162	M1	1	0	KEGG Pathway	24	Measles	-12.0185	14.64301	13.41551	30242	158	183	14	7.650273	1.964856	595|596|836|3551|3558|3569|4792|5290|5291|5293|5970|7099|7157|29110	CCND1|BCL2|CASP3|IKBKB|IL2|IL6|NFKBIA|PIK3CA|PIK3CB|PIK3CD|RELA|TLR4|TP53|TBK1	-10.9046	0	4	0	0	-22.7123	31.71596
1	-11.5471	hsa05212	M1	1	0	KEGG Pathway	24	Pancreatic cancer	-11.5471	22.1686	14.97682	30242	82	183	11	6.010929	1.757049	595|673|1956|3551|5290|5291|5293|5970|7040|7046|7157	CCND1|BRAF|EGFR|IKBKB|PIK3CA|PIK3CB|PIK3CD|RELA|T	-10.4471	0	4	0	0	-22.7123	31.71596

																			GFB1|TGFBR1|TP53							
1	-11.5378	ko04210	M1	1	0	KEGG Pathway	24	Apoptosis	-11.5378	15.56767	13.38301	30242	138	183	13	7.103825	1.898976	472|596|836|3551|4792|4914|5290|5291|5293|5970|7124|7132|7157	ATM|BCL2|CASP3|IKBKB|NFKBIA|NTRK1|PIK3CA|PIK3CB|PIK3CD|RELA|TNF|TNFRSF1A|TP53	-10.4447	0	4	0	0	-22.7123	31.71596
1	-11.5255	ko05210	M1	1	0	KEGG Pathway	24	Colorectal cancer	-11.5255	27.54281	16.05775	30242	60	183	10	5.464481	1.680144	595|596|673|836|5290|5291|5293|7040|7046|7157	CCND1|BCL2|BRAF|CASP3|PIK3CA|PIK3CB|PIK3CD|TGFB1|TGFBR1|TP53	-10.4392	0	4	0	0	-22.7123	31.71596
1	-11.3438	hsa05169	M1	1	0	KEGG Pathway	24	Epstein-Barr virus infection	-11.3438	9.395873	11.38388	30242	299	183	17	9.289617	2.145862	595|596|836|890|3066|3383|3551|3569|3683|4792|5290|5291|5293|5970|7124|7157|29110	CCND1|BCL2|CASP3|CCNA2|HDAC2|ICAM1|IKBKB|IL6|ITGAL|NFKBIA|PIK3CA|PIK3CB|PIK3CD|RELA|TNF|TP53|TBK1	-10.2641	0	4	0	0	-22.7123	31.71596
1	-11.1613	ko05230	M1	1	0	KEGG Pathway	24	Central carbon metabolism in cancer	-11.1613	25.42413	15.38063	30242	65	183	10	5.464481	1.680144	1956|2263|4914|5159|5290|5291|5293|5979|6513|7157	EGFR|FGFR2|NTRK1|PDGFRB|PIK3CA|PIK3CB|PIK3CD|RET|SLC2A1|TP53	-10.0945	0	4	0	0	-22.7123	31.71596
1	-11.1106	ko04932	M1	1	0	KEGG Pathway	24	Non-alcoholic fatty liver disease (NAFLD)	-11.1106	14.41838	12.81141	30242	149	183	13	7.103825	1.898976	836|3551|3569|3576|3643|5290|5291|5293|5465|5970|7040|7124|7132	CASP3|IKBKB|IL6|CXCL8|INSR|PIK3CA|PIK3CB|PIK3CD|PPARA|RELA|TGFB1|TNF|TNFRSF1A	-10.0502	0	4	0	0	-22.7123	31.71596
1	-11.043	ko04722	M1	1	0	KEGG Pathway	24	Neurotrophin signaling pathway	-11.043	16.66455	13.35915	30242	119	183	12	6.557377	1.829835	596|673|3551|4792|4914|4915|5290|5291|5293|5970|6197|7157	BCL2|BRAF|IKBKB|NFKBIA|NTRK1|NTRK2|PIK3CA|PIK3CB|PIK3CD|RELA|RPS6KA3|TP53	-9.99503	0	4	0	0	-22.7123	31.71596
1	-11.0367	hsa04210	M1	1	0	KEGG Pathway	24	Apoptosis	-11.0367	14.22741	12.71397	30242	151	183	13	7.103825	1.898976	472|596|836|3551|4792|4914|5290|5291|5293|5970|7124|7132|7157	ATM|BCL2|CASP3|IKBKB|NFKBIA|NTRK1|PIK3CA|PIK3CB|PIK3CD|RELA|TNF|TNFRSF1A|TP53	-9.99472	0	4	0	0	-22.7123	31.71596
1	-10.8814	hsa05210	M1	1	0	KEGG Pathway	24	Colorectal cancer	-10.8814	19.33857	13.89429	30242	94	183	11	6.010929	1.757049	595|596|673|836|1956|5290|5291|5293|7040|7046|7157	CCND1|BCL2|BRAF|CASP3|EGFR|PIK3CA|PIK3CB|PIK3CD|TGFB1|TGFBR1|TP53	-9.85134	0	4	0	0	-22.7123	31.71596
1	-10.8295	hsa04722	M1	1	0	KEGG Pathway	24	Neurotrophin signaling pathway	-10.8295	15.9926	13.05302	30242	124	183	12	6.557377	1.829835	596|673|3551|4792|4914|4915|5290|5291|5293|5970|6197|7157	BCL2|BRAF|IKBKB|NFKBIA|NTRK1|NTRK2|PIK3CA|PIK3CB|PIK3CD|RELA|RPS6KA3|TP53	-9.8144	0	4	0	0	-22.7123	31.71596
1	-10.3438	hsa05230	M1	1	0	KEGG Pathway	24	Central carbon metabolism in cancer	-10.3438	21.18677	13.92856	30242	78	183	10	5.464481	1.680144	1956|2263|4914|5159|5290|5291|5293|5979|6513|7157	EGFR|FGFR2|NTRK1|PDGFRB|PIK3CA|PIK3CB|PIK3CD|RET|SLC2A1|TP53	-9.37395	0	4	0	0	-22.7123	31.71596
1	-10.3162	hsa04068	M1	1	0	KEGG Pathway	24	foxo signaling pathway	-10.3162	14.47505	12.33411	30242	137	183	12	6.557377	1.829835	472|595|673|1956|3551|3569|3643|5290|5291|5293|7040|7046	ATM|CCND1|BRAF|EGFR|IKBKB|IL6|INSR|PIK3CA|PIK3CB|	-9.35141	0	4	0	0	-22.7123	31.71596

																			PIK3CD|TGFB1|TGFBR1							
1	-10.2873	hsa01521	M1	1	0	KEGG Pathway	24	EGFR tyrosine kinase inhibitor resistance	-10.2873	20.91859	13.83156	30242	79	183	10	5.464481	1.680144	596|673|1956|2263|3569|5159|5290|5291|5293|5582	BCL2|BRAF|EGFR|FGFR2|IL6|PDGFRB|PIK3CA|PIK3CB|PIK3CD|PRKCG	-9.33741	0	4	0	0	-22.7123	31.71596
1	-10.2873	ko01521	M1	1	0	KEGG Pathway	24	EGFR tyrosine kinase inhibitor resistance	-10.2873	20.91859	13.83156	30242	79	183	10	5.464481	1.680144	596|673|1956|2263|3569|5159|5290|5291|5293|5582	BCL2|BRAF|EGFR|FGFR2|IL6|PDGFRB|PIK3CA|PIK3CB|PIK3CD|PRKCG	-9.33741	0	4	0	0	-22.7123	31.71596
1	-10.1329	hsa04923	M1	1	0	KEGG Pathway	24	Regulation of lipolysis in adipocytes	-10.1329	25.6433	14.65753	30242	58	183	9	4.918033	1.598524	134|153|154|3643|5290|5291|5293|5742|5743	ADORA1|ADRB1|ADRB2|INSR|PIK3CA|PIK3CB|PIK3CD|PTGS1|PTGS2	-9.19737	0	4	0	0	-22.7123	31.71596
1	-10.0264	hsa04915	M1	1	0	KEGG Pathway	24	Estrogen signaling pathway	-10.0264	13.67643	11.93866	30242	145	183	12	6.557377	1.829835	596|1956|2099|2100|4313|4318|4846|4988|5241|5290|5291|5293	BCL2|EGFR|ESR1|ESR2|MMP2|MMP9|NOS3|OPRM1|PGR|PIK3CA|PIK3CB|PIK3CD	-9.10484	0	4	0	0	-22.7123	31.71596
1	-10.0127	ko04062	M1	1	0	KEGG Pathway	24	Chemokine signaling pathway	-10.0127	11.80406	11.40682	30242	182	183	13	7.103825	1.898976	673|1230|3551|3576|3577|4792|5290|5291|5293|5970|6347|9475|729230	BRAF|CCR1|IKBKB|CXCL8|CXCR1|NFKBIA|PIK3CA|PIK3CB|PIK3CD|RELA|CCL2|ROCK2|CCR2	-9.09569	0	4	0	0	-22.7123	31.71596
1	-9.7337	ko05214	M1	1	0	KEGG Pathway	24	Glioma	-9.7337	23.23924	13.89636	30242	64	183	9	4.918033	1.598524	595|673|1956|5159|5290|5291|5293|5582|7157	CCND1|BRAF|EGFR|PDGFRB|PIK3CA|PIK3CB|PIK3CD|PRKCG|TP53	-8.83875	0	4	0	0	-22.7123	31.71596
1	-9.47689	ko05169	M1	1	0	KEGG Pathway	24	Epstein-Barr virus infection	-9.47689	10.68825	10.75283	30242	201	183	13	7.103825	1.898976	596|890|3066|3383|3551|3683|4792|5290|5291|5293|5970|7157|29110	BCL2|CCNA2|HDAC2|ICAM1|IKBKB|ITGAL|NFKBIA|PIK3CA|PIK3CB|PIK3CD|RELA|TP53|TBK1	-8.60292	0	4	0	0	-22.7123	31.71596
1	-9.45027	hsa04062	M1	1	0	KEGG Pathway	24	Chemokine signaling pathway	-9.45027	10.63534	10.72085	30242	202	183	13	7.103825	1.898976	673|1230|3551|3576|3577|4792|5290|5291|5293|5970|6347|9475|729230	BRAF|CCR1|IKBKB|CXCL8|CXCR1|NFKBIA|PIK3CA|PIK3CB|PIK3CD|RELA|CCL2|ROCK2|CCR2	-8.58038	0	4	0	0	-22.7123	31.71596
1	-9.37499	hsa04152	M1	1	0	KEGG Pathway	24	AMPK signaling pathway	-9.37499	14.09167	11.6265	30242	129	183	11	6.010929	1.757049	146|148|595|890|1080|3156|3643|5290|5291|5293|5468	ADRA1D|ADRA1A|CCND1|CCNA2|CFTR|HMGCR|INSR|PIK3CA|PIK3CB|PIK3CD|PPARG	-8.51314	0	4	0	0	-22.7123	31.71596
1	-9.34255	ko04915	M1	1	0	KEGG Pathway	24	Estrogen signaling pathway	-9.34255	16.86294	12.27249	30242	98	183	10	5.464481	1.680144	1956|2099|2100|4313|4318|4846|4988|5290|5291|5293	EGFR|ESR1|ESR2|MMP2|MMP9|NOS3|OPRM1|PIK3CA|PIK3CB|PIK3CD	-8.48491	0	4	0	0	-22.7123	31.71596
1	-9.33886	ko04380	M1	1	0	KEGG Pathway	24	Osteoclast differentiation	-9.33886	13.98327	11.57504	30242	130	183	11	6.010929	1.757049	3551|4792|5290|5291|5293|5468|5970|7040|7046|7124|7132	IKBKB|NFKBIA|PIK3CA|PIK3CB|PIK3CD|PPARG|RELA|TGFB1|TGFBR1|TNF|TNFRSF1A	-8.48491	0	4	0	0	-22.7123	31.71596

<table>
<tr><th></th><th></th><th></th><th></th><th></th><th></th><th></th><th></th><th></th><th></th><th></th><th></th><th></th><th></th><th></th><th></th><th></th><th></th><th></th><th></th><th></th><th></th><th></th><th></th><th></th><th></th><th></th></tr>
<tr><td>1</td><td>-9.19737</td><td>ko05162</td><td>M1</td><td>1</td><td>0</td><td>KEGG Pathway</td><td>24</td><td>Measles</td><td>-9.19737</td><td>13.56586</td><td>11.3747</td><td>30242</td><td>134</td><td>183</td><td>11</td><td>6.010929</td><td>1.757049</td><td>595|3558|3569|4792|5290|5291|5293|5970|7099|7157|29110</td><td>CCND1|IL2|IL6|NFKBIA|PIK3CA|PIK3CB|PIK3CD|RELA|TLR4|TP53|TBK1</td><td>-8.36628</td><td>0</td><td>4</td><td>0</td><td>0</td><td>-22.7123</td><td>31.71596</td></tr>
<tr><td>1</td><td>-9.06047</td><td>hsa04380</td><td>M1</td><td>1</td><td>0</td><td>KEGG Pathway</td><td>24</td><td>Osteoclast differentiation</td><td>-9.06047</td><td>13.17265</td><td>11.18275</td><td>30242</td><td>138</td><td>183</td><td>11</td><td>6.010929</td><td>1.757049</td><td>3551|4792|5290|5291|5293|5468|5970|7040|7046|7124|7132</td><td>IKBKB|NFKBIA|PIK3CA|PIK3CB|PIK3CD|PPARG|RELA|TGFB1|TGFBR1|TNF|TNFRSF1A</td><td>-8.23308</td><td>0</td><td>4</td><td>0</td><td>0</td><td>-22.7123</td><td>31.71596</td></tr>
<tr><td>1</td><td>-8.94426</td><td>hsa05214</td><td>M1</td><td>1</td><td>0</td><td>KEGG Pathway</td><td>24</td><td>Glioma</td><td>-8.94426</td><td>19.0681</td><td>12.4667</td><td>30242</td><td>78</td><td>183</td><td>9</td><td>4.918033</td><td>1.598524</td><td>595|673|1956|5159|5290|5291|5293|5582|7157</td><td>CCND1|BRAF|EGFR|PDGFRB|PIK3CA|PIK3CB|PIK3CD|PRKCG|TP53</td><td>-8.12417</td><td>0</td><td>4</td><td>0</td><td>0</td><td>-22.7123</td><td>31.71596</td></tr>
<tr><td>1</td><td>-8.83196</td><td>ko05221</td><td>M1</td><td>1</td><td>0</td><td>KEGG Pathway</td><td>24</td><td>Acute myeloid leukemia</td><td>-8.83196</td><td>24.03736</td><td>13.3426</td><td>30242</td><td>55</td><td>183</td><td>8</td><td>4.371585</td><td>1.511428</td><td>595|673|3551|5290|5291|5293|5467|5970</td><td>CCND1|BRAF|IKBKB|PIK3CA|PIK3CB|PIK3CD|PPARD|RELA</td><td>-8.02259</td><td>0</td><td>4</td><td>0</td><td>0</td><td>-22.7123</td><td>31.71596</td></tr>
<tr><td>1</td><td>-8.69967</td><td>hsa05218</td><td>M1</td><td>1</td><td>0</td><td>KEGG Pathway</td><td>24</td><td>Melanoma</td><td>-8.69967</td><td>17.91942</td><td>12.04349</td><td>30242</td><td>83</td><td>183</td><td>9</td><td>4.918033</td><td>1.598524</td><td>595|673|1956|3569|5159|5290|5291|5293|7157</td><td>CCND1|BRAF|EGFR|IL6|PDGFRB|PIK3CA|PIK3CB|PIK3CD|TP53</td><td>-7.90761</td><td>0</td><td>4</td><td>0</td><td>0</td><td>-22.7123</td><td>31.71596</td></tr>
<tr><td>1</td><td>-8.64159</td><td>ko05223</td><td>M1</td><td>1</td><td>0</td><td>KEGG Pathway</td><td>24</td><td>Non-small cell lung cancer</td><td>-8.64159</td><td>22.79405</td><td>12.96283</td><td>30242</td><td>58</td><td>183</td><td>8</td><td>4.371585</td><td>1.511428</td><td>595|673|1956|5290|5291|5293|5582|7157</td><td>CCND1|BRAF|EGFR|PIK3CA|PIK3CB|PIK3CD|PRKCG|TP53</td><td>-7.8529</td><td>0</td><td>4</td><td>0</td><td>0</td><td>-22.7123</td><td>31.71596</td></tr>
<tr><td>1</td><td>-8.4739</td><td>ko04152</td><td>M1</td><td>1</td><td>0</td><td>KEGG Pathway</td><td>24</td><td>AMPK signaling pathway</td><td>-8.4739</td><td>13.7714</td><td>10.93764</td><td>30242</td><td>120</td><td>183</td><td>10</td><td>5.464481</td><td>1.680144</td><td>148|595|890|1080|3156|3643|5290|5291|5293|5468</td><td>ADRA1A|CCND1|CCNA2|CFTR|HMGCR|INSR|PIK3CA|PIK3CB|PIK3CD|PPARG</td><td>-7.69847</td><td>0</td><td>4</td><td>0</td><td>0</td><td>-22.7123</td><td>31.71596</td></tr>
<tr><td>1</td><td>-8.06526</td><td>hsa04510</td><td>M1</td><td>1</td><td>0</td><td>KEGG Pathway</td><td>24</td><td>Focal adhesion</td><td>-8.06526</td><td>9.223637</td><td>9.44199</td><td>30242</td><td>215</td><td>183</td><td>12</td><td>6.557377</td><td>1.829835</td><td>595|596|673|1956|2335|3569|5159|5290|5291|5293|5582|9475</td><td>CCND1|BCL2|BRAF|EGFR|FN1|IL6|PDGFRB|PIK3CA|PIK3CB|PIK3CD|PRKCG|ROCK2</td><td>-7.31521</td><td>0</td><td>4</td><td>0</td><td>0</td><td>-22.7123</td><td>31.71596</td></tr>
<tr><td>1</td><td>-8.02647</td><td>ko05218</td><td>M1</td><td>1</td><td>0</td><td>KEGG Pathway</td><td>24</td><td>Melanoma</td><td>-8.02647</td><td>19.16021</td><td>11.78345</td><td>30242</td><td>69</td><td>183</td><td>8</td><td>4.371585</td><td>1.511428</td><td>595|673|1956|5159|5290|5291|5293|7157</td><td>CCND1|BRAF|EGFR|PDGFRB|PIK3CA|PIK3CB|PIK3CD|TP53</td><td>-7.27949</td><td>0</td><td>4</td><td>0</td><td>0</td><td>-22.7123</td><td>31.71596</td></tr>
<tr><td>1</td><td>-7.976</td><td>hsa05221</td><td>M1</td><td>1</td><td>0</td><td>KEGG Pathway</td><td>24</td><td>Acute myeloid leukemia</td><td>-7.976</td><td>18.88649</td><td>11.68984</td><td>30242</td><td>70</td><td>183</td><td>8</td><td>4.371585</td><td>1.511428</td><td>595|673|3551|5290|5291|5293|5467|5970</td><td>CCND1|BRAF|IKBKB|PIK3CA|PIK3CB|PIK3CD|PPARD|RELA</td><td>-7.23509</td><td>0</td><td>4</td><td>0</td><td>0</td><td>-22.7123</td><td>31.71596</td></tr>
<tr><td>1</td><td>-7.976</td><td>hsa05223</td><td>M1</td><td>1</td><td>0</td><td>KEGG Pathway</td><td>24</td><td>Non-small cell lung cancer</td><td>-7.976</td><td>18.88649</td><td>11.68984</td><td>30242</td><td>70</td><td>183</td><td>8</td><td>4.371585</td><td>1.511428</td><td>595|673|1956|5290|5291|5293|5582|7157</td><td>CCND1|BRAF|EGFR|PIK3CA|PIK3CB|PIK3CD|PRKCG|TP53</td><td>-7.23509</td><td>0</td><td>4</td><td>0</td><td>0</td><td>-22.7123</td><td>31.71596</td></tr>
<tr><td>1</td><td>-7.9351</td><td>ko04930</td><td>M1</td><td>1</td><td>0</td><td>KEGG Pathway</td><td>24</td><td>Type II diabetes mellitus</td><td>-7.9351</td><td>25.14778</td><td>12.78845</td><td>30242</td><td>46</td><td>183</td><td>7</td><td>3.825137</td><td>1.417845</td><td>775|3551|3643|5290|5291|5293|7124</td><td>CACNA1C|IKBKB|INSR|PIK3CA|PIK3CB|PIK3CD|TNF</td><td>-7.1972</td><td>0</td><td>4</td><td>0</td><td>0</td><td>-22.7123</td><td>31.71596</td></tr>
<tr><td>1</td><td>-7.70781</td><td>ko05224</td><td>M1</td><td>1</td><td>0</td><td>KEGG Pathway</td><td>24</td><td>Breast cancer</td><td>-7.70781</td><td>11.47617</td><td>9.832213</td><td>30242</td><td>144</td><td>183</td><td>10</td><td>5.464481</td><td>1.680144</td><td>595|673|1956|2099|2100|5241|5290|5291|5293|7157</td><td>CCND1|BRAF|EGFR|ESR1|ESR2|PGR|PIK3CA|PIK3CB|PIK3CD|TP53</td><td>-6.9788</td><td>0</td><td>4</td><td>0</td><td>0</td><td>-22.7123</td><td>31.71596</td></tr>
<tr><td>1</td><td>-7.67301</td><td>ko05213</td><td>M1</td><td>1</td><td>0</td><td>KEGG Pathway</td><td>24</td><td>Endometrial cancer</td><td>-7.67301</td><td>23.13596</td><td>12.22288</td><td>30242</td><td>50</td><td>183</td><td>7</td><td>3.825137</td><td>1.417845</td><td>595|673|1956|5290|5291|5293|7157</td><td>CCND1|BRAF|EGFR|PIK3CA|PIK3CB|PIK3CD|TP53</td><td>-6.94692</td><td>0</td><td>4</td><td>0</td><td>0</td><td>-22.7123</td><td>31.71596</td></tr>
</table>

1	-7.51091	ko04150	M1	1	0	KEGG Pathway	24	mTOR signaling pathway	-7.51091	10.94416	9.558168	30242	151	183	10	5.464481	1.680144	673|3551|3643|5290|5291|5293|5582|6197|7124|7132	BRAF|IKBKB|INSR|PIK3CA|PIK3CB|PIK3CD|PRKCG|RPS6KA3|TNF|TNFRSF1A	-6.78773	0	4	0	0	-22.7123	31.71596
1	-7.47453	ko04670	M1	1	0	KEGG Pathway	24	Leukocyte transendothelial migration	-7.47453	13.04659	10.05467	30242	114	183	9	4.918033	1.598524	3383|3683|4313|4318|5290|5291|5293|5582|9475	ICAM1|ITGAL|MMP2|MMP9|PIK3CA|PIK3CB|PIK3CD|PRKCG|ROCK2	-6.75423	0	4	0	0	-22.7123	31.71596
1	-7.39465	ko04510	M1	1	0	KEGG Pathway	24	Focal adhesion	-7.39465	9.1348	8.983346	30242	199	183	11	6.010929	1.757049	595|596|673|1956|2335|5159|5290|5291|5293|5582|9475	CCND1|BCL2|BRAF|EGFR|FN1|PDGFRB|PIK3CA|PIK3CB|PIK3CD|PRKCG|ROCK2	-6.67722	0	4	0	0	-22.7123	31.71596
1	-7.32395	hsa04150	M1	1	0	KEGG Pathway	24	mTOR signaling pathway	-7.32395	10.45929	9.30156	30242	158	183	10	5.464481	1.680144	673|3551|3643|5290|5291|5293|5582|6197|7124|7132	BRAF|IKBKB|INSR|PIK3CA|PIK3CB|PIK3CD|PRKCG|RPS6KA3|TNF|TNFRSF1A	-6.60937	0	4	0	0	-22.7123	31.71596
1	-7.24901	hsa04670	M1	1	0	KEGG Pathway	24	Leukocyte transendothelial migration	-7.24901	12.29183	9.710882	30242	121	183	9	4.918033	1.598524	3383|3683|4313|4318|5290|5291|5293|5582|9475	ICAM1|ITGAL|MMP2|MMP9|PIK3CA|PIK3CB|PIK3CD|PRKCG|ROCK2	-6.53726	0	4	0	0	-22.7123	31.71596
1	-7.22123	hsa05224	M1	1	0	KEGG Pathway	24	Breast cancer	-7.22123	10.20104	9.162032	30242	162	183	10	5.464481	1.680144	595|673|1956|2099|2100|5241|5290|5291|5293|7157	CCND1|BRAF|EGFR|ESR1|ESR2|PGR|PIK3CA|PIK3CB|PIK3CD|TP53	-6.51229	0	4	0	0	-22.7123	31.71596
1	-7.21211	hsa04930	M1	1	0	KEGG Pathway	24	Type II diabetes mellitus	-7.21211	19.94479	11.26813	30242	58	183	7	3.825137	1.417845	775|3551|3643|5290|5291|5293|7124	CACNA1C|IKBKB|INSR|PIK3CA|PIK3CB|PIK3CD|TNF	-6.50596	0	4	0	0	-22.7123	31.71596
1	-7.18723	hsa04919	M1	1	0	KEGG Pathway	24	thyroid hormone signaling pathway	-7.18723	12.09196	9.617829	30242	123	183	9	4.918033	1.598524	595|2099|3066|5290|5291|5293|5582|6513|7157	CCND1|ESR1|HDAC2|PIK3CA|PIK3CB|PIK3CD|PRKCG|SLC2A1|TP53	-6.48385	0	4	0	0	-22.7123	31.71596
1	-6.97946	hsa04611	M1	1	0	KEGG Pathway	24	Platelet activation	-6.97946	11.44086	9.308386	30242	130	183	9	4.918033	1.598524	2147|2149|4846|5290|5291|5293|5742|6916|9475	F2|F2R|NOS3|PIK3CA|PIK3CB|PIK3CD|PTGS1|TBXAS1|ROCK2	-6.28701	0	4	0	0	-22.7123	31.71596
1	-6.89419	ko04650	M1	1	0	KEGG Pathway	24	Natural killer cell mediated cytotoxicity	-6.89419	11.18279	9.182923	30242	133	183	9	4.918033	1.598524	673|836|3383|3683|5290|5291|5293|5582|7124	BRAF|CASP3|ICAM1|ITGAL|PIK3CA|PIK3CB|PIK3CD|PRKCG|TNF	-6.20976	0	4	0	0	-22.7123	31.71596
1	-6.77076	hsa05213	M1	1	0	KEGG Pathway	24	Endometrial cancer	-6.77076	17.26564	10.39971	30242	67	183	7	3.825137	1.417845	595|673|1956|5290|5291|5293|7157	CCND1|BRAF|EGFR|PIK3CA|PIK3CB|PIK3CD|TP53	-6.0942	0	4	0	0	-22.7123	31.71596
1	-6.64856	hsa04914	M1	1	0	KEGG Pathway	24	Progesterone-mediated oocyte maturation	-6.64856	12.83548	9.388074	30242	103	183	8	4.371585	1.511428	673|890|5241|5290|5291|5293|6197|6793	BRAF|CCNA2|PGR|PIK3CA|PIK3CB|PIK3CD|RPS6KA3|STK10	-5.98733	0	4	0	0	-22.7123	31.71596
1	-6.64856	ko04660	M1	1	0	KEGG Pathway	24	T cell receptor signaling pathway	-6.64856	12.83548	9.388074	30242	103	183	8	4.371585	1.511428	3551|3558|4792|5290|5291|5293|5970|7124	IKBKB|IL2|NFKBIA|PIK3CA|PIK3CB|PIK3CD|RELA|TNF	-5.98733	0	4	0	0	-22.7123	31.71596
1	-6.638	ko04917	M1	1	0	KEGG Pathway	24	Prolactin signaling pathway	-6.638	16.52568	10.14692	30242	70	183	7	3.825137	1.417845	595|2099|2100|5290|5291|5293|5970	CCND1|ESR1|ESR2|PIK3CA|PIK3CB|PIK3CD|RELA	-5.97927	0	4	0	0	-22.7123	31.71596

1	-6.4978	hsa04650	M1	1	0	KEGG Pathway	24	Natural killer cell mediated cytotoxicity	-6.4978	10.0494	8.610872	30242	148	183	9	4.918033	1.598524	673|836|3383|3683|5290|5291|5293|5582|7124	BRAF|CASP3|ICAM1|ITGAL|PIK3CA|PIK3CB|PIK3CD|PRKCG|TNF	-5.8657	0	4	0	0	-22.7123	31.71596
1	-6.45841	hsa04211	M1	1	0	KEGG Pathway	24	Longevity regulating pathway	-6.45841	12.12894	9.082016	30242	109	183	8	4.371585	1.511428	3066|3643|5290|5291|5293|5468|5970|7157	HDAC2|INSR|PIK3CA|PIK3CB|PIK3CD|PPARG|RELA|TP53	-5.82865	0	4	0	0	-22.7123	31.71596
1	-6.39031	hsa04917	M1	1	0	KEGG Pathway	24	Prolactin signaling pathway	-6.39031	15.22102	9.685346	30242	76	183	7	3.825137	1.417845	595|2099|2100|5290|5291|5293|5970	CCND1|ESR1|ESR2|PIK3CA|PIK3CB|PIK3CD|RELA	-5.76519	0	4	0	0	-22.7123	31.71596
1	-6.34824	ko05203	M1	1	0	KEGG Pathway	24	Viral carcinogenesis	-6.34824	8.221733	8.015278	30242	201	183	10	5.464481	1.680144	595|836|890|3066|4792|5290|5291|5293|5970|7157	CCND1|CASP3|CCNA2|HDAC2|NFKBIA|PIK3CA|PIK3CB|PIK3CD|RELA|TP53	-5.72543	0	4	0	0	-22.7123	31.71596
1	-6.33799	hsa04660	M1	1	0	KEGG Pathway	24	T cell receptor signaling pathway	-6.33799	11.6996	8.891002	30242	113	183	8	4.371585	1.511428	3551|3558|4792|5290|5291|5293|5970|7124	IKBKB|IL2|NFKBIA|PIK3CA|PIK3CB|PIK3CD|RELA|TNF	-5.71747	0	4	0	0	-22.7123	31.71596
1	-6.16326	hsa05211	M1	1	0	KEGG Pathway	24	Renal cell carcinoma	-6.16326	14.10729	9.273433	30242	82	183	7	3.825137	1.417845	673|3569|5290|5291|5293|6513|7040	BRAF|IL6|PIK3CA|PIK3CB|PIK3CD|SLC2A1|TGFB1	-5.54955	0	4	0	0	-22.7123	31.71596
1	-6.1552	hsa05203	M1	1	0	KEGG Pathway	24	Viral carcinogenesis	-6.1552	7.832077	7.770437	30242	211	183	10	5.464481	1.680144	595|836|890|3066|4792|5290|5291|5293|5970|7157	CCND1|CASP3|CCNA2|HDAC2|NFKBIA|PIK3CA|PIK3CB|PIK3CD|RELA|TP53	-5.54373	0	4	0	0	-22.7123	31.71596
1	-5.97012	hsa04630	M1	1	0	KEGG Pathway	24	Jak-STAT signaling pathway	-5.97012	8.697728	7.876314	30242	171	183	9	4.918033	1.598524	595|596|1956|3558|3569|5159|5290|5291|5293	CCND1|BCL2|EGFR|IL2|IL6|PDGFRB|PIK3CA|PIK3CB|PIK3CD	-5.3631	0	4	0	0	-22.7123	31.71596
1	-5.80648	ko04370	M1	1	0	KEGG Pathway	24	VEGF signaling pathway	-5.80648	16.80578	9.481937	30242	59	183	6	3.278689	1.316393	4846|5290|5291|5293|5582|5743	NOS3|PIK3CA|PIK3CB|PIK3CD|PRKCG|PTGS2	-5.20606	0	4	0	0	-22.7123	31.71596
1	-5.69791	ko04914	M1	1	0	KEGG Pathway	24	Progesterone-mediated oocyte maturation	-5.69791	12.04998	8.460938	30242	96	183	7	3.825137	1.417845	673|890|5241|5290|5291|5293|6197	BRAF|CCNA2|PGR|PIK3CA|PIK3CB|PIK3CD|RPS6KA3	-5.10399	0	4	0	0	-22.7123	31.71596
1	-5.55633	hsa04370	M1	1	0	KEGG Pathway	24	VEGF signaling pathway	-5.55633	15.25448	8.976484	30242	65	183	6	3.278689	1.316393	4846|5290|5291|5293|5582|5743	NOS3|PIK3CA|PIK3CB|PIK3CD|PRKCG|PTGS2	-4.97927	0	4	0	0	-22.7123	31.71596
1	-5.55633	ko05211	M1	1	0	KEGG Pathway	24	Renal cell carcinoma	-5.55633	15.25448	8.976484	30242	65	183	6	3.278689	1.316393	673|5290|5291|5293|6513|7040	BRAF|PIK3CA|PIK3CB|PIK3CD|SLC2A1|TGFB1	-4.97927	0	4	0	0	-22.7123	31.71596
1	-5.54342	ko04960	M1	1	0	KEGG Pathway	24	Aldosterone-regulated sodium reabsorption	-5.54342	22.332	10.13047	30242	37	183	5	2.73224	1.205087	3643|5290|5291|5293|5582	INSR|PIK3CA|PIK3CB|PIK3CD|PRKCG	-4.96843	0	4	0	0	-22.7123	31.71596
1	-5.42682	hsa04960	M1	1	0	KEGG Pathway	24	Aldosterone-regulated sodium reabsorption	-5.42682	21.18677	9.842615	30242	39	183	5	2.73224	1.205087	3643|5290|5291|5293|5582	INSR|PIK3CA|PIK3CB|PIK3CD|PRKCG	-4.85999	0	4	0	0	-22.7123	31.71596
1	-5.33034	ko04662	M1	1	0	KEGG Pathway	24	B cell receptor signaling pathway	-5.33034	13.96537	8.534055	30242	71	183	6	3.278689	1.316393	3551|4792|5290|5291|5293|5970	IKBKB|NFKBIA|PIK3CA|PIK3CB|PIK3CD|RELA	-4.77151	0	4	0	0	-22.7123	31.71596
1	-4.9968	hsa04662	M1	1	0	KEGG Pathway	24	B cell receptor signaling pathway	-4.9968	12.24125	7.904428	30242	81	183	6	3.278689	1.316393	3551|4792|5290|5291|5293|5970	IKBKB|NFKBIA|PIK3CA|PIK3CB|PIK3CD|RELA	-4.45164	0	4	0	0	-22.7123	31.71596
1	-4.84668	ko04012	M1	1	0	KEGG Pathway	24	ErbB signaling	-4.84668	11.52955	7.629716	30242	86	183	6	3.278689	1.316393	673|1956|5290|5291|5	BRAF|EGFR|PIK3CA	-4.30914	0	4	0	0	-22.7123	31.71596

								pathway										293|5582	|PIK3CB|PIK3CD|PRKCG							
1	-4.62554	hsa04012	M1	1	0	KEGG Pathway	24	ErbB signaling pathway	-4.62554	10.54831	7.234316	30242	94	183	6	3.278689	1.316393	673|1956|5290|5291|5293|5582	BRAF|EGFR|PIK3CA|PIK3CB|PIK3CD|PRKCG	-4.09918	0	4	0	0	-22.7123	31.71596
1	-4.4977	ko05231	M1	1	0	KEGG Pathway	24	Choline metabolism in cancer	-4.4977	10.01557	7.010576	30242	99	183	6	3.278689	1.316393	1956|5159|5290|5291|5293|5582	EGFR|PDGFRB|PIK3CA|PIK3CB|PIK3CD|PRKCG	-3.97318	0	4	0	0	-22.7123	31.71596
1	-4.42495	hsa04213	M1	1	0	KEGG Pathway	24	Longevity regulating pathway - multiple species	-4.42495	13.32716	7.581161	30242	62	183	5	2.73224	1.205087	3066|3643|5290|5291|5293	HDAC2|INSR|PIK3CA|PIK3CB|PIK3CD	-3.90408	0	4	0	0	-22.7123	31.71596
1	-4.42495	ko04213	M1	1	0	KEGG Pathway	24	Longevity regulating pathway - multiple species	-4.42495	13.32716	7.581161	30242	62	183	5	2.73224	1.205087	3066|3643|5290|5291|5293	HDAC2|INSR|PIK3CA|PIK3CB|PIK3CD	-3.90408	0	4	0	0	-22.7123	31.71596
1	-4.31617	ko04630	M1	1	0	KEGG Pathway	24	Jak-STAT signaling pathway	-4.31617	7.415371	6.268133	30242	156	183	7	3.825137	1.417845	595|596|3558|3569|5290|5291|5293	CCND1|BCL2|IL2|IL6|PIK3CA|PIK3CB|PIK3CD	-3.79891	0	4	0	0	-22.7123	31.71596
1	-4.30745	hsa05231	M1	1	0	KEGG Pathway	24	Choline metabolism in cancer	-4.30745	9.266738	6.683852	30242	107	183	6	3.278689	1.316393	1956|5159|5290|5291|5293|5582	EGFR|PDGFRB|PIK3CA|PIK3CB|PIK3CD|PRKCG	-3.79199	0	4	0	0	-22.7123	31.71596
1	-3.83632	ko04973	M1	1	0	KEGG Pathway	24	Carbohydrate digestion and absorption	-3.83632	15.02335	7.263155	30242	44	183	4	2.185792	1.080886	5290|5291|5293|6523	PIK3CA|PIK3CB|PIK3CD|SLC5A1	-3.34017	0	4	0	0	-22.7123	31.71596
1	-3.69744	ko04910	M1	1	0	KEGG Pathway	24	Insulin signaling pathway	-3.69744	7.18508	5.682103	30242	138	183	6	3.278689	1.316393	673|3551|3643|5290|5291|5293	BRAF|IKBKB|INSR|PIK3CA|PIK3CB|PIK3CD	-3.21481	0	4	0	0	-22.7123	31.71596
1	-3.52087	hsa04973	M1	1	0	KEGG Pathway	24	Carbohydrate digestion and absorption	-3.52087	12.47221	6.522246	30242	53	183	4	2.185792	1.080886	5290|5291|5293|6523	PIK3CA|PIK3CB|PIK3CD|SLC5A1	-3.04973	0	4	0	0	-22.7123	31.71596
1	-3.42611	hsa04910	M1	1	0	KEGG Pathway	24	Insulin signaling pathway	-3.42611	6.397039	5.256156	30242	155	183	6	3.278689	1.316393	673|3551|3643|5290|5291|5293	BRAF|IKBKB|INSR|PIK3CA|PIK3CB|PIK3CD	-2.95659	0	4	0	0	-22.7123	31.71596
1	-3.10726	ko04664	M1	1	0	KEGG Pathway	24	Fc epsilon RI signaling pathway	-3.10726	9.72099	5.617462	30242	68	183	4	2.185792	1.080886	5290|5291|5293|7124	PIK3CA|PIK3CB|PIK3CD|TNF	-2.65204	0	4	0	0	-22.7123	31.71596
1	-2.92617	ko05100	M1	1	0	KEGG Pathway	24	Bacterial invasion of epithelial cells	-2.92617	8.697728	5.242601	30242	76	183	4	2.185792	1.080886	2335|5290|5291|5293	FN1|PIK3CA|PIK3CB|PIK3CD	-2.48629	0	4	0	0	-22.7123	31.71596
1	-2.86368	hsa04664	M1	1	0	KEGG Pathway	24	Fc epsilon RI signaling pathway	-2.86368	8.367434	5.115981	30242	79	183	4	2.185792	1.080886	5290|5291|5293|7124	PIK3CA|PIK3CB|PIK3CD|TNF	-2.4268	0	4	0	0	-22.7123	31.71596
1	-2.74631	hsa05100	M1	1	0	KEGG Pathway	24	Bacterial invasion of epithelial cells	-2.74631	7.776792	4.881754	30242	85	183	4	2.185792	1.080886	2335|5290|5291|5293	FN1|PIK3CA|PIK3CB|PIK3CD	-2.3139	0	4	0	0	-22.7123	31.71596
1	-2.63793	ko04666	M1	1	0	KEGG Pathway	24	Fc gamma R-mediated phagocytosis	-2.63793	7.264037	4.669393	30242	91	183	4	2.185792	1.080886	5290|5291|5293|5582	PIK3CA|PIK3CB|PIK3CD|PRKCG	-2.21432	0	4	0	0	-22.7123	31.71596
1	-2.53736	ko04070	M1	1	0	KEGG Pathway	24	Phosphatidylinositol signaling system	-2.53736	6.814715	4.475515	30242	97	183	4	2.185792	1.080886	5290|5291|5293|5582	PIK3CA|PIK3CB|PIK3CD|PRKCG	-2.11883	0	4	0	0	-22.7123	31.71596
1	-2.44365	hsa04070	M1	1	0	KEGG Pathway	24	Phosphatidylinositol signaling system	-2.44365	6.417741	4.29743	30242	103	183	4	2.185792	1.080886	5290|5291|5293|5582	PIK3CA|PIK3CB|PIK3CD|PRKCG	-2.03292	0	4	0	0	-22.7123	31.71596
1	-2.39911	hsa04666	M1	1	0	KEGG Pathway	24	Fc gamma R-mediated phagocytosis	-2.39911	6.236107	4.213616	30242	106	183	4	2.185792	1.080886	5290|5291|5293|5582	PIK3CA|PIK3CB|PIK3CD|PRKCG	-1.9912	0	4	0	0	-22.7123	31.71596
1	-2.36294	ko04360	M1	1	0	KEGG Pathway	24	Axon guidance	-2.36294	4.721624	3.852501	30242	175	183	5	2.73224	1.205087	2050|5290|5291|5293|9475	EPHB4|PIK3CA|PIK3CB|PIK3CD|ROCK2	-1.95643	0	4	0	0	-22.7123	31.71596

1	-2.15797	hsa04360	M1	1	0	KEGG Pathway	24	Axon guidance	-2.15797	4.215735	3.524126	30242	196	183	5	2.73224	1.205087	2050|5290|5291|5293|9475	EPHB4|PIK3CA|PIK3CB|PIK3CD|ROCK2	-1.75424	0	4	0	0	-22.7123	31.71596
1	-2.1116	ko04140	M1	1	0	KEGG Pathway	24	Autophagy - animal	-2.1116	5.164276	3.683805	30242	128	183	4	2.185792	1.080886	596|5290|5291|5293	BCL2|PIK3CA|PIK3CB|PIK3CD	-1.71201	0	4	0	0	-22.7123	31.71596
1	-2.06557	hsa04140	M1	1	0	KEGG Pathway	24	Regulation of autophagy	-2.06557	5.007783	3.600577	30242	132	183	4	2.185792	1.080886	596|5290|5291|5293	BCL2|PIK3CA|PIK3CB|PIK3CD	-1.67009	0	4	0	0	-22.7123	31.71596
1	-2.03543	ko00562	M1	1	0	KEGG Pathway	24	Inositol phosphate metabolism	-2.03543	6.982683	3.937918	30242	71	183	3	1.639344	0.938686	5290|5291|5293	PIK3CA|PIK3CB|PIK3CD	-1.64266	0	4	0	0	-22.7123	31.71596
1	-22.7077	ko04022	M1	1	0	KEGG Pathway	24	cGMP-PKG signaling pathway	-22.7077	22.3046	21.28001	30242	163	183	22	12.02186	2.404073	134|140|146|147|148|150|151|152|153|154|185|624|775|1909|1910|3643|3778|4846|4985|5138|8654|9475	ADORA1|ADORA3|ADRA1D|ADRA1B|ADRA1A|ADRA2A|ADRA2B|ADRA2C|ADRB1|ADRB2|AGTR1|BDKRB2|CACNA1C|EDNRA|EDNRB|INSR|KCNMA1|NOS3|OPRD1|PDE2A|PDE5A|ROCK2	-20.7626	0	5	1	1	-22.7077	22.88172
1	-22.1221	hsa04022	M1	1	0	KEGG Pathway	24	cGMP-PKG signaling pathway	-22.1221	21.01532	20.59977	30242	173	183	22	12.02186	2.404073	134|140|146|147|148|150|151|152|153|154|185|624|775|1909|1910|3643|3778|4846|4985|5138|8654|9475	ADORA1|ADORA3|ADRA1D|ADRA1B|ADRA1A|ADRA2A|ADRA2B|ADRA2C|ADRB1|ADRB2|AGTR1|BDKRB2|CACNA1C|EDNRA|EDNRB|INSR|KCNMA1|NOS3|OPRD1|PDE2A|PDE5A|ROCK2	-20.2228	0	5	0	0	-22.7077	22.88172
1	-12.8788	hsa04270	M1	1	0	KEGG Pathway	24	Vascular smooth muscle contraction	-12.8788	16.88756	14.54235	30242	137	183	14	7.650273	1.964856	135|136|146|147|148|185|673|775|1909|3778|5582|9475|10203|114548	ADORA2A|ADORA2B|ADRA1D|ADRA1B|ADRA1A|AGTR1|BRAF|CACNA1C|EDNRA|KCNMA1|PRKCG|ROCK2|CALCRL|NLRP3	-11.7038	0	5	0	0	-22.7077	22.88172
1	-12.2782	ko04270	M1	1	0	KEGG Pathway	24	Vascular smooth muscle contraction	-12.2782	17.75487	14.40905	30242	121	183	13	7.103825	1.898976	135|136|146|147|148|185|673|775|1909|3778|5582|9475|10203	ADORA2A|ADORA2B|ADRA1D|ADRA1B|ADRA1A|AGTR1|BRAF|CACNA1C|EDNRA|KCNMA1|PRKCG|ROCK2|CALCRL	-11.1424	0	5	0	0	-22.7077	22.88172
1	-9.67123	ko04924	M1	1	0	KEGG Pathway	24	Renin secretion	-9.67123	22.88172	13.77959	30242	65	183	9	4.918033	1.598524	134|153|154|185|775|1636|1909|3778|5734	ADORA1|ADRB1|ADRB2|AGTR1|CACNA1C|ACE|EDNRA|KCNMA1|PTGER4	-8.78479	0	5	0	0	-22.7077	22.88172
1	-9.20681	hsa04924	M1	1	0	KEGG Pathway	24	Renin secretion	-9.20681	20.37413	12.93122	30242	73	183	9	4.918033	1.598524	134|153|154|185|775|1636|1909|3778|5734	ADORA1|ADRB1|ADRB2|AGTR1|CACNA1C|ACE|EDNRA|KCNMA1|PTGER4	-8.37199	0	5	0	0	-22.7077	22.88172
1	-8.38326	ko04970	M1	1	0	KEGG Pathway	24	Salivary secretion	-8.38326	16.52568	11.50934	30242	90	183	9	4.918033	1.598524	146|147|148|153|154|1131|3778|4842|5582	ADRA1D|ADRA1B|ADRA1A|ADRB1|A	-7.61109	0	5	0	0	-22.7077	22.88172

																			DRB2|CHRM3|KCNMA1|NOS1|PRKCG							
1	-8.21441	hsa04970	M1	1	0	KEGG Pathway	24	Salivary secretion	-8.21441	15.82246	11.2303	30242	94	183	9	4.918033	1.598524	146|147|148|153|154|1131|3778|4842|5582	ADRA1D|ADRA1B|ADRA1A|ADRB1|ADRB2|CHRM3|KCNMA1|NOS1|PRKCG	-7.46127	0	5	0	0	-22.7077	22.88172
1	-6.59895	ko04261	M1	1	0	KEGG Pathway	24	Adrenergic signaling in cardiomyocytes	-6.59895	10.32855	8.755138	30242	144	183	9	4.918033	1.598524	146|147|148|153|154|185|596|775|6331	ADRA1D|ADRA1B|ADRA1A|ADRB1|ADRB2|AGTR1|BCL2|CACNA1C|SCN5A	-5.94383	0	5	0	0	-22.7077	22.88172
1	-6.5228	hsa04261	M1	1	0	KEGG Pathway	24	Adrenergic signaling in cardiomyocytes	-6.5228	10.11777	8.646418	30242	147	183	9	4.918033	1.598524	146|147|148|153|154|185|596|775|6331	ADRA1D|ADRA1B|ADRA1A|ADRB1|ADRB2|AGTR1|BCL2|CACNA1C|SCN5A	-5.8788	0	5	0	0	-22.7077	22.88172
1	-19.562	hsa04726	M1	1	0	KEGG Pathway	24	Serotonergic synapse	-19.562	24.99683	20.46514	30242	119	183	18	9.836066	2.201413	351|673|775|836|1557|3350|3351|3356|3357|3358|3362|3363|4128|4129|5582|5742|5743|6532	APP|BRAF|CACNA1C|CASP3|CYP2C19|HTR1A|HTR1B|HTR2A|HTR2B|HTR2C|HTR6|HTR7|MAOA|MAOB|PRKCG|PTGS1|PTGS2|SLC6A4	-17.8388	0	6	1	1	-19.562	24.99683
1	-15.652	hsa04015	M1	1	0	KEGG Pathway	24	Rap1 signaling pathway	-15.652	13.7714	15.1036	30242	228	183	19	10.38251	2.254873	135|136|673|1268|1813|1956|2149|2263|2902|2903|2904|3569|3643|3683|5159|5290|5291|5293|5582	ADORA2A|ADORA2B|BRAF|CNR1|DRD2|EGFR|F2R|FGFR2|GRIN1|GRIN2A|GRIN2B|IL6|INSR|ITGAL|PDGFRB|PIK3CA|PIK3CB|PIK3CD|PRKCG	-14.133	0	7	1	1	-15.652	18.05327
1	-15.065	ko04015	M1	1	0	KEGG Pathway	24	Rap1 signaling pathway	-15.065	14.16487	14.93724	30242	210	183	18	9.836066	2.201413	135|136|673|1268|1813|1956|2149|2263|2902|2903|2904|3643|3683|5159|5290|5291|5293|5582	ADORA2A|ADORA2B|BRAF|CNR1|DRD2|EGFR|F2R|FGFR2|GRIN1|GRIN2A|GRIN2B|INSR|ITGAL|PDGFRB|PIK3CA|PIK3CB|PIK3CD|PRKCG	-13.5971	0	7	0	0	-15.652	18.05327
1	-14.9922	ko04810	M1	1	0	KEGG Pathway	24	Regulation of actin cytoskeleton	-14.9922	14.03124	14.85636	30242	212	183	18	9.836066	2.201413	624|673|1128|1129|1131|1132|1133|1956|2147|2149|2263|2335|3683|5159|5290|5291|5293|9475	BDKRB2|BRAF|CHRM1|CHRM2|CHRM3|CHRM4|CHRM5|EGFR|F2|F2R|FGFR2|FN1|ITGAL|PDGFRB|PIK3CA|PIK3CB|PIK3CD|ROCK2	-13.5401	0	7	0	0	-15.652	18.05327
1	-14.7293	hsa04151	M1	1	0	KEGG Pathway	24	PI3K-Akt signaling pathway	-14.7293	9.567501	13.11363	30242	380	183	22	12.02186	2.404073	595|596|1128|1129|1956|2149|2263|2335|3551|3558|3569|3643|4846|4914|4915|5159|5290|5291|5293|5970|7099|7157	CCND1|BCL2|CHRM1|CHRM2|EGFR|F2R|FGFR2|FN1|IKBKB|IL2|IL6|INSR|NOS3|NTRK1|NTRK2|PDGFRB|PIK3CA|PIK3CB|PIK3CD|RELA|TLR4|TP53	-13.3277	0	7	0	0	-15.652	18.05327

1	-14.1744	hsa04810	M1	1	0	KEGG Pathway	24	Regulation of actin cytoskeleton	-14.1744	12.60433	13.96396	30242	236	183	18	9.836066	2.201413	624|673|1128|1129|1131|1132|1133|1956|2147|2149|2263|2335|3683|5159|5290|5291|5293|9475	BDKRB2|BRAF|CHRM1|CHRM2|CHRM3|CHRM4|CHRM5|EGFR|F2|F2R|FGFR2|FN1|ITGAL|PDGFRB|PIK3CA|PIK3CB|PIK3CD|ROCK2	-12.8067	0	7	0	0	-15.652	18.05327
1	-13.5023	ko04151	M1	1	0	KEGG Pathway	24	PI3K-Akt signaling pathway	-13.5023	9.664142	12.57301	30242	342	183	20	10.92896	2.306387	595|596|1128|1129|1956|2149|2263|2335|3551|3558|3569|3643|4846|5159|5290|5291|5293|5970|7099|7157	CCND1|BCL2|CHRM1|CHRM2|EGFR|F2R|FGFR2|FN1|IKBKB|IL2|IL6|INSR|NOS3|PDGFRB|PIK3CA|PIK3CB|PIK3CD|RELA|TLR4|TP53	-12.2562	0	7	0	0	-15.652	18.05327
1	-13.4732	hsa04014	M1	1	0	KEGG Pathway	24	Ras signaling pathway	-13.4732	11.48503	13.22265	30242	259	183	18	9.836066	2.201413	1956|2263|2902|2903|2904|3363|3551|3569|3643|4914|4915|5159|5290|5291|5293|5582|5970|29110	EGFR|FGFR2|GRIN1|GRIN2A|GRIN2B|HTR7|IKBKB|IL6|INSR|NTRK1|NTRK2|PDGFRB|PIK3CA|PIK3CB|PIK3CD|PRKCG|RELA|TBK1	-12.2367	0	7	0	0	-15.652	18.05327
1	-12.3729	hsa04066	M1	1	0	KEGG Pathway	24	HIF-1 signaling pathway	-12.3729	18.05327	14.54348	30242	119	183	13	7.103825	1.898976	596|1956|3569|3643|4843|4846|5290|5291|5293|5582|5970|6513|7099	BCL2|EGFR|IL6|INSR|NOS2|NOS3|PIK3CA|PIK3CB|PIK3CD|PRKCG|RELA|SLC2A1|TLR4	-11.2295	0	7	0	0	-15.652	18.05327
1	-10.6148	hsa04072	M1	1	0	KEGG Pathway	24	Phospholipase D signaling pathway	-10.6148	13.17999	12.16593	30242	163	183	13	7.103825	1.898976	185|1956|2147|2149|2915|3576|3577|3643|5159|5290|5291|5293|114548	AGTR1|EGFR|F2|F2R|GRM5|CXCL8|CXCR1|INSR|PDGFRB|PIK3CA|PIK3CB|PIK3CD|NLRP3	-9.61312	0	7	0	0	-15.652	18.05327
1	-9.91596	hsa04725	M1	1	0	KEGG Pathway	24	Cholinergic synapse	-9.91596	15.80718	12.41308	30242	115	183	11	6.010929	1.757049	596|775|1128|1129|1131|1132|1133|5290|5291|5293|5582	BCL2|CACNA1C|CHRM1|CHRM2|CHRM3|CHRM4|CHRM5|PIK3CA|PIK3CB|PIK3CD|PRKCG	-9.00792	0	7	0	0	-15.652	18.05327
1	-8.79943	ko04072	M1	1	0	KEGG Pathway	24	Phospholipase D signaling pathway	-8.79943	12.45086	10.82172	30242	146	183	11	6.010929	1.757049	185|1956|2149|2915|3576|3577|3643|5159|5290|5291|5293	AGTR1|EGFR|F2R|GRM5|CXCL8|CXCR1|INSR|PDGFRB|PIK3CA|PIK3CB|PIK3CD	-7.99358	0	7	0	0	-15.652	18.05327
1	-15.2827	ko04657	M1	1	0	KEGG Pathway	24	IL-17 signaling pathway	-15.2827	24.87737	17.99401	30242	93	183	14	7.650273	1.964856	836|3551|3569|3576|4312|4314|4318|4792|5743|5970|6347|7124|29110|225689	CASP3|IKBKB|IL6|CXCL8|MMP1|MMP3|MMP9|NFKBIA|PTGS2|RELA|CCL2|TNF|TBK1|MAPK15	-13.7814	0	8	1	1	-15.2827	37.31606
1	-15.148	hsa04657	M1	1	0	KEGG Pathway	24	IL-17 signaling pathway	-15.148	24.35364	17.78814	30242	95	183	14	7.650273	1.964856	836|3551|3569|3576|4312|4314|4318|4792|5743|5970|6347|7124|29110|225689	CASP3|IKBKB|IL6|CXCL8|MMP1|MMP3|MMP9|NFKBIA|PTGS2|RELA|CCL2|TNF|TBK1|MAPK15	-13.6637	0	8	0	0	-15.2827	37.31606
1	-13.9604	ko05164	M1	1	0	KEGG Pathway	24	Influenza A	-13.9604	15.28387	14.70096	30242	173	183	16	8.743169	2.088053	834|3383|3551|3569|3	CASP1|ICAM1|IKBK	-12.6522	0	8	0	0	-15.2827	37.31606

																		576|4792|5290|5291|5293|5970|6347|7099|7124|7132|29110|114548	B|IL6|CXCL8|NFKBIA|PIK3CA|PIK3CB|PIK3CD|RELA|CCL2|TLR4|TNF|TNFRSF1A|TBK1|NLRP3							
1	-13.6126	hsa05164	M1	1	0	KEGG Pathway	24	Influenza A	-13.6126	14.52807	14.2828	30242	182	183	16	8.743169	2.088053	834|3383|3551|3569|3576|4792|5290|5291|5293|5970|6347|7099|7124|7132|29110|114548	CASP1|ICAM1|IKBKB|IL6|CXCL8|NFKBIA|PIK3CA|PIK3CB|PIK3CD|RELA|CCL2|TLR4|TNF|TNFRSF1A|TBK1|NLRP3	-12.3414	0	8	0	0	-15.2827	37.31606
1	-10.8302	ko04064	M1	1	0	KEGG Pathway	24	NF-kappa B signaling pathway	-10.8302	19.135	13.81318	30242	95	183	11	6.010929	1.757049	472|596|3383|3551|3576|4792|5743|5970|7099|7124|7132	ATM|BCL2|ICAM1|IKBKB|CXCL8|NFKBIA|PTGS2|RELA|TLR4|TNF|TNFRSF1A	-9.8144	0	8	0	0	-15.2827	37.31606
1	-10.3948	ko04620	M1	1	0	KEGG Pathway	24	Toll-like receptor signaling pathway	-10.3948	17.47909	13.13497	30242	104	183	11	6.010929	1.757049	3551|3569|3576|4792|5290|5291|5293|5970|7099|7124|29110	IKBKB|IL6|CXCL8|NFKBIA|PIK3CA|PIK3CB|PIK3CD|RELA|TLR4|TNF|TBK1	-9.40934	0	8	0	0	-15.2827	37.31606
1	-10.3843	ko04621	M1	1	0	KEGG Pathway	24	NOD-like receptor signaling pathway	-10.3843	12.63729	11.87221	30242	170	183	13	7.103825	1.898976	596|834|3551|3569|3576|4792|5970|6347|7099|7124|10135|29110|114548	BCL2|CASP1|IKBKB|IL6|CXCL8|NFKBIA|RELA|CCL2|TLR4|TNF|NAMPT|TBK1|NLRP3	-9.40409	0	8	0	0	-15.2827	37.31606
1	-10.2588	hsa04064	M1	1	0	KEGG Pathway	24	NF-kappa B signaling pathway	-10.2588	16.98902	12.9275	30242	107	183	11	6.010929	1.757049	472|596|3383|3551|3576|4792|5743|5970|7099|7124|7132	ATM|BCL2|ICAM1|IKBKB|CXCL8|NFKBIA|PTGS2|RELA|TLR4|TNF|TNFRSF1A	-9.31382	0	8	0	0	-15.2827	37.31606
1	-10.084	hsa04620	M1	1	0	KEGG Pathway	24	Toll-like receptor signaling pathway	-10.084	16.3768	12.6636	30242	111	183	11	6.010929	1.757049	3551|3569|3576|4792|5290|5291|5293|5970|7099|7124|29110	IKBKB|IL6|CXCL8|NFKBIA|PIK3CA|PIK3CB|PIK3CD|RELA|TLR4|TNF|TBK1	-9.1532	0	8	0	0	-15.2827	37.31606
1	-9.89485	hsa04621	M1	1	0	KEGG Pathway	24	NOD-like receptor signaling pathway	-9.89485	11.55021	11.26129	30242	186	183	13	7.103825	1.898976	596|834|3551|3569|3576|4792|5970|6347|7099|7124|10135|29110|114548	BCL2|CASP1|IKBKB|IL6|CXCL8|NFKBIA|RELA|CCL2|TLR4|TNF|NAMPT|TBK1|NLRP3	-8.99558	0	8	0	0	-15.2827	37.31606
1	-9.21056	hsa01523	M1	1	0	KEGG Pathway	24	Antifolate resistance	-9.21056	37.31606	15.78459	30242	31	183	7	3.825137	1.417845	3551|3569|4363|5970|7124|7298|9429	IKBKB|IL6|ABCC1|RELA|TNF|TYMS|ABCG2	-8.37199	0	8	0	0	-15.2827	37.31606
1	-9.21056	ko01523	M1	1	0	KEGG Pathway	24	Antifolate resistance	-9.21056	37.31606	15.78459	30242	31	183	7	3.825137	1.417845	3551|3569|4363|5970|7124|7298|9429	IKBKB|IL6|ABCC1|RELA|TNF|TYMS|ABCG2	-8.37199	0	8	0	0	-15.2827	37.31606
1	-9.04699	ko05133	M1	1	0	KEGG Pathway	24	Pertussis	-9.04699	19.56989	12.64718	30242	76	183	9	4.918033	1.598524	834|836|3569|3576|4843|5970|7099|7124|114548	CASP1|CASP3|IL6|CXCL8|NOS2|RELA|TLR4|TNF|NLRP3	-8.22326	0	8	0	0	-15.2827	37.31606
1	-8.83196	ko05134	M1	1	0	KEGG Pathway	24	Legionellosis	-8.83196	24.03736	13.3426	30242	55	183	8	4.371585	1.511428	834|836|3569|3576|4792|5970|7099|7124	CASP1|CASP3|IL6|CXCL8|NFKBIA|RELA|TLR4|TNF	-8.02259	0	8	0	0	-15.2827	37.31606
1	-8.7955	hsa05133	M1	1	0	KEGG Pathway	24	Pertussis	-8.7955	18.36187	12.20822	30242	81	183	9	4.918033	1.598524	834|836|3569|3576|4843|5970|7099|7124|11	CASP1|CASP3|IL6|CXCL8|NOS2|RELA|T	-7.99314	0	8	0	0	-15.2827	37.31606

																		4548	LR4|TNF|NLRP3							
1	-8.72977	ko05145	M1	1	0	KEGG Pathway	24	Toxoplasmosis	-8.72977	14.6245	11.32149	30242	113	183	10	5.464481	1.680144	596|836|3551|4792|4843|5970|7040|7099|7124|7132	BCL2|CASP3|IKBKB|NFKBIA|NOS2|RELA|TGFB1|TLR4|TNF|TNFRSF1A	-7.93086	0	8	0	0	-15.2827	37.31606
1	-8.61802	hsa05145	M1	1	0	KEGG Pathway	24	Toxoplasmosis	-8.61802	14.24628	11.15292	30242	116	183	10	5.464481	1.680144	596|836|3551|4792|4843|5970|7040|7099|7124|7132	BCL2|CASP3|IKBKB|NFKBIA|NOS2|RELA|TGFB1|TLR4|TNF|TNFRSF1A	-7.83269	0	8	0	0	-15.2827	37.31606
1	-8.58054	hsa05134	M1	1	0	KEGG Pathway	24	Legionellosis	-8.58054	22.40771	12.84255	30242	59	183	8	4.371585	1.511428	834|836|3569|3576|4792|5970|7099|7124	CASP1|CASP3|IL6|CXCL8|NFKBIA|RELA|TLR4|TNF	-7.80184	0	8	0	0	-15.2827	37.31606
1	-6.81435	ko05152	M1	1	0	KEGG Pathway	24	Tuberculosis	-6.81435	9.232225	8.61914	30242	179	183	10	5.464481	1.680144	596|836|3569|4843|5970|7040|7099|7124|7132|7421	BCL2|CASP3|IL6|NOS2|RELA|TGFB1|TLR4|TNF|TNFRSF1A|VDR	-6.13519	0	8	0	0	-15.2827	37.31606
1	-6.72579	ko05120	M1	1	0	KEGG Pathway	24	Epithelial cell signaling in Helicobacter pylori infection	-6.72579	17.01173	10.31366	30242	68	183	7	3.825137	1.417845	836|1956|3551|3576|3577|4792|5970	CASP3|EGFR|IKBKB|CXCL8|CXCR1|NFKBIA|RELA	-6.05183	0	8	0	0	-15.2827	37.31606
1	-6.68154	ko04920	M1	1	0	KEGG Pathway	24	Adipocytokine signaling pathway	-6.68154	16.76519	10.22941	30242	69	183	7	3.825137	1.417845	3551|4792|5465|5970|6513|7124|7132	IKBKB|NFKBIA|PPARA|RELA|SLC2A1|TNF|TNFRSF1A	-6.01477	0	8	0	0	-15.2827	37.31606
1	-6.68105	hsa05168	M1	1	0	KEGG Pathway	24	Herpes simplex infection	-6.68105	8.932802	8.44454	30242	185	183	10	5.464481	1.680144	836|3551|3569|4792|5970|6347|7124|7132|7157|29110	CASP3|IKBKB|IL6|NFKBIA|RELA|CCL2|TNF|TNFRSF1A|TP53|TBK1	-6.01477	0	8	0	0	-15.2827	37.31606
1	-6.68105	ko05168	M1	1	0	KEGG Pathway	24	Herpes simplex infection	-6.68105	8.932802	8.44454	30242	185	183	10	5.464481	1.680144	836|3551|3569|4792|5970|6347|7124|7132|7157|29110	CASP3|IKBKB|IL6|NFKBIA|RELA|CCL2|TNF|TNFRSF1A|TP53|TBK1	-6.01477	0	8	0	0	-15.2827	37.31606
1	-6.59513	hsa05120	M1	1	0	KEGG Pathway	24	Epithelial cell signaling in Helicobacter pylori infection	-6.59513	16.29293	10.0661	30242	71	183	7	3.825137	1.417845	836|1956|3551|3576|3577|4792|5970	CASP3|EGFR|IKBKB|CXCL8|CXCR1|NFKBIA|RELA	-5.94383	0	8	0	0	-15.2827	37.31606
1	-6.55292	hsa04920	M1	1	0	KEGG Pathway	24	Adipocytokine signaling pathway	-6.55292	16.06664	9.986913	30242	72	183	7	3.825137	1.417845	3551|4792|5465|5970|6513|7124|7132	IKBKB|NFKBIA|PPARA|RELA|SLC2A1|TNF|TNFRSF1A	-5.90407	0	8	0	0	-15.2827	37.31606
1	-6.53158	hsa05152	M1	1	0	KEGG Pathway	24	Tuberculosis	-6.53158	8.607127	8.250597	30242	192	183	10	5.464481	1.680144	596|836|3569|4843|5970|7040|7099|7124|7132|7421	BCL2|CASP3|IL6|NOS2|RELA|TGFB1|TLR4|TNF|TNFRSF1A|VDR	-5.88516	0	8	0	0	-15.2827	37.31606
1	-6.51135	hsa05140	M1	1	0	KEGG Pathway	24	Leishmania infection	-6.51135	15.84655	9.909295	30242	73	183	7	3.825137	1.417845	4792|4843|5743|5970|7040|7099|7124	NFKBIA|NOS2|PTGS2|RELA|TGFB1|TLR4|TNF	-5.8769	0	8	0	0	-15.2827	37.31606
1	-6.51135	ko05140	M1	1	0	KEGG Pathway	24	Leishmaniasis	-6.51135	15.84655	9.909295	30242	73	183	7	3.825137	1.417845	4792|4843|5743|5970|7040|7099|7124	NFKBIA|NOS2|PTGS2|RELA|TGFB1|TLR4|TNF	-5.8769	0	8	0	0	-15.2827	37.31606
1	-6.02185	ko05132	M1	1	0	KEGG Pathway	24	Salmonella infection	-6.02185	13.45114	9.022102	30242	86	183	7	3.825137	1.417845	834|3569|3576|4843|5970|7099|9475	CASP1|IL6|CXCL8|NOS2|RELA|TLR4|ROCK2	-5.41261	0	8	0	0	-15.2827	37.31606

1	-5.72858	hsa05132	M1	1	0	KEGG Pathway	24	Salmonella infection	-5.72858	12.17682	8.513229	30242	95	183	7	3.825137	1.417845	834|3569|3576|4843|5970|7099|9475	CASP1|IL6|CXCL8|NOS2|RELA|TLR4|ROCK2	-5.13034	0	8	0	0	-15.2827	37.31606
1	-5.63679	ko04623	M1	1	0	KEGG Pathway	24	Cytosolic DNA-sensing pathway	-5.63679	15.73875	9.137234	30242	63	183	6	3.278689	1.316393	834|3551|3569|4792|5970|29110	CASP1|IKBKB|IL6|NFKBIA|RELA|TBK1	-5.04715	0	8	0	0	-15.2827	37.31606
1	-5.59621	hsa04623	M1	1	0	KEGG Pathway	24	Cytosolic DNA-sensing pathway	-5.59621	15.49283	9.055955	30242	64	183	6	3.278689	1.316393	834|3551|3569|4792|5970|29110	CASP1|IKBKB|IL6|NFKBIA|RELA|TBK1	-5.01081	0	8	0	0	-15.2827	37.31606
1	-5.38218	ko04659	M1	1	0	KEGG Pathway	24	Th17 cell differentiation	-5.38218	10.81119	7.932581	30242	107	183	7	3.825137	1.417845	3551|3558|3569|4792|5970|7040|7046	IKBKB|IL2|IL6|NFKBIA|RELA|TGFB1|TGFBR1	-4.81736	0	8	0	0	-15.2827	37.31606
1	-5.36652	ko04622	M1	1	0	KEGG Pathway	24	RIG-I-like receptor signaling pathway	-5.36652	14.16487	8.603991	30242	70	183	6	3.278689	1.316393	3551|3576|4792|5970|7124|29110	IKBKB|CXCL8|NFKBIA|RELA|TNF|TBK1	-4.8037	0	8	0	0	-15.2827	37.31606
1	-5.29473	hsa04622	M1	1	0	KEGG Pathway	24	RIG-I-like receptor signaling pathway	-5.29473	13.7714	8.465518	30242	72	183	6	3.278689	1.316393	3551|3576|4792|5970|7124|29110	IKBKB|CXCL8|NFKBIA|RELA|TNF|TBK1	-4.73788	0	8	0	0	-15.2827	37.31606
1	-5.2504	hsa04659	M1	1	0	KEGG Pathway	24	Th17 cell differentiation	-5.2504	10.32855	7.717205	30242	112	183	7	3.825137	1.417845	3551|3558|3569|4792|5970|7040|7046	IKBKB|IL2|IL6|NFKBIA|RELA|TGFB1|TGFBR1	-4.69552	0	8	0	0	-15.2827	37.31606
1	-4.23061	hsa05131	M1	1	0	KEGG Pathway	24	Shigellosis	-4.23061	12.15124	7.182861	30242	68	183	5	2.73224	1.205087	3551|3576|4792|5970|9475	IKBKB|CXCL8|NFKBIA|RELA|ROCK2	-3.7175	0	8	0	0	-15.2827	37.31606
1	-2.62065	ko04658	M1	1	0	KEGG Pathway	24	Th1 and Th2 cell differentiation	-2.62065	7.18508	4.635877	30242	92	183	4	2.185792	1.080886	3551|3558|4792|5970	IKBKB|IL2|NFKBIA|RELA	-2.1985	0	8	0	0	-15.2827	37.31606
1	-2.52128	hsa04658	M1	1	0	KEGG Pathway	24	Th1 and Th2 cell differentiation	-2.52128	6.745177	4.444801	30242	98	183	4	2.185792	1.080886	3551|3558|4792|5970	IKBKB|IL2|NFKBIA|RELA	-2.10489	0	8	0	0	-15.2827	37.31606
1	-14.0476	hsa04010	M1	1	0	KEGG Pathway	24	MAPK signaling pathway	-14.0476	10.32855	13.08972	30242	320	183	20	10.92896	2.306387	673|775|836|1956|2263|3551|3569|3643|4914|4915|5159|5582|5970|6197|7040|7046|7124|7132|7157|8986	BRAF|CACNA1C|CASP3|EGFR|FGFR2|IKBKB|IL6|INSR|NTRK1|NTRK2|PDGFRB|PRKCG|RELA|RPS6KA3|TGFB1|TGFBR1|TNF|TNFRSF1A|TP53|RPS6KA4	-12.7281	0	9	1	1	-14.0476	11.66519
1	-13.59	ko04010	M1	1	0	KEGG Pathway	24	MAPK signaling pathway	-13.59	11.66519	13.34469	30242	255	183	18	9.836066	2.201413	673|775|836|1956|2263|3551|4914|4915|5159|5582|5970|6197|7040|7046|7124|7132|7157|8986	BRAF|CACNA1C|CASP3|EGFR|FGFR2|IKBKB|NTRK1|NTRK2|PDGFRB|PRKCG|RELA|RPS6KA3|TGFB1|TGFBR1|TNF|TNFRSF1A|TP53|RPS6KA4	-12.3342	0	9	0	0	-14.0476	11.66519
1	-7.92297	ko04060	M1	1	0	KEGG Pathway	24	Cytokine-cytokine receptor interaction	-7.92297	7.95681	8.959227	30242	270	183	13	7.103825	1.898976	1230|1956|3558|3569|3576|3577|5159|6347|7040|7046|7124|7132|729230	CCR1|EGFR|IL2|IL6|CXCL8|CXCR1|PDGFRB|CCL2|TGFB1|TGFBR1|TNF|TNFRSF1A|CCR2	-7.18805	0	9	0	0	-14.0476	11.66519
1	-6.93342	hsa04060	M1	1	0	KEGG Pathway	24	Cytokine-cytokine receptor interaction	-6.93342	6.549813	7.885231	30242	328	183	13	7.103825	1.898976	1230|1956|3558|3569|3576|3577|5159|6347|7040|7046|7124|7132|729230	CCR1|EGFR|IL2|IL6|CXCL8|CXCR1|PDGFRB|CCL2|TGFB1|TGFBR1|TNF|TNFRSF1A|CCR2	-6.24367	0	9	0	0	-14.0476	11.66519
1	-13.945	ko05014	M1	1	0	KEGG Pathway	24	Amyotrophic lateral	-13.945	35.64363	19.31995	30242	51	183	11	6.010929	1.757049	596|834|836|2891|290	BCL2|CASP1|CASP3|	-12.6478	0	10	1	1	-13.945	35.64363

								sclerosis (ALS)										2|2903|2904|4842|7124|7132|7157	GRIA2|GRIN1|GRIN2A|GRIN2B|NOS1|TNF|TNFRSF1A|TP53							
1	-13.3705	hsa05014	M1	1	0	KEGG Pathway	24	Amyotrophic lateral sclerosis (ALS)	-13.3705	31.89167	18.2146	30242	57	183	11	6.010929	1.757049	596|834|836|2891|2902|2903|2904|4842|7124|7132|7157	BCL2|CASP1|CASP3|GRIA2|GRIN1|GRIN2A|GRIN2B|NOS1|TNF|TNFRSF1A|TP53	-12.1525	0	10	0	0	-13.945	35.64363
1	-12.4632	ko05030	M1	1	0	KEGG Pathway	24	Cocaine addiction	-12.4632	33.72588	17.88845	30242	49	183	10	5.464481	1.680144	1812|1813|2891|2902|2903|2904|4128|4129|5970|6531	DRD1|DRD2|GRIA2|GRIN1|GRIN2A|GRIN2B|MAOA|MAOB|RELA|SLC6A3	-11.3121	0	10	0	0	-13.945	35.64363
1	-11.9254	hsa05030	M1	1	0	KEGG Pathway	24	Cocaine addiction	-11.9254	30.0467	16.82304	30242	55	183	10	5.464481	1.680144	1812|1813|2891|2902|2903|2904|4128|4129|5970|6531	DRD1|DRD2|GRIA2|GRIN1|GRIN2A|GRIN2B|MAOA|MAOB|RELA|SLC6A3	-10.8186	0	10	0	0	-13.945	35.64363
1	-10.9574	ko05031	M1	1	0	KEGG Pathway	24	Amphetamine addiction	-10.9574	24.30248	15.00985	30242	68	183	10	5.464481	1.680144	775|1812|2891|2902|2903|2904|4128|4129|5582|6531	CACNA1C|DRD1|GRIA2|GRIN1|GRIN2A|GRIN2B|MAOA|MAOB|PRKCG|SLC6A3	-9.92149	0	10	0	0	-13.945	35.64363
1	-10.7634	hsa05031	M1	1	0	KEGG Pathway	24	Amphetamine addiction	-10.7634	23.27561	14.66224	30242	71	183	10	5.464481	1.680144	775|1812|2891|2902|2903|2904|4128|4129|5582|6531	CACNA1C|DRD1|GRIA2|GRIN1|GRIN2A|GRIN2B|MAOA|MAOB|PRKCG|SLC6A3	-9.75618	0	10	0	0	-13.945	35.64363
1	-10.5856	ko04728	M1	1	0	KEGG Pathway	24	Dopaminergic synapse	-10.5856	15.25448	12.70836	30242	130	183	12	6.557377	1.829835	775|1812|1813|1814|1815|2891|2903|2904|4128|4129|5582|6531	CACNA1C|DRD1|DRD2|DRD3|DRD4|GRIA2|GRIN2A|GRIN2B|MAOA|MAOB|PRKCG|SLC6A3	-9.58937	0	10	0	0	-13.945	35.64363
1	-10.507	hsa04728	M1	1	0	KEGG Pathway	24	Dopaminergic synapse	-10.507	15.02335	12.59852	30242	132	183	12	6.557377	1.829835	775|1812|1813|1814|1815|2891|2903|2904|4128|4129|5582|6531	CACNA1C|DRD1|DRD2|DRD3|DRD4|GRIA2|GRIN2A|GRIN2B|MAOA|MAOB|PRKCG|SLC6A3	-9.5162	0	10	0	0	-13.945	35.64363
1	-10.0727	ko05034	M1	1	0	KEGG Pathway	24	Alcoholism	-10.0727	11.93522	11.4813	30242	180	183	13	7.103825	1.898976	135|136|673|1812|1813|2902|2903|2904|3066|4128|4129|4915|6531	ADORA2A|ADORA2B|BRAF|DRD1|DRD2|GRIN1|GRIN2A|GRIN2B|HDAC2|MAOA|MAOB|NTRK2|SLC6A3	-9.14655	0	10	0	0	-13.945	35.64363
1	-9.92405	hsa05034	M1	1	0	KEGG Pathway	24	Alcoholism	-9.92405	11.61264	11.29725	30242	185	183	13	7.103825	1.898976	135|136|673|1812|1813|2902|2903|2904|3066|4128|4129|4915|6531	ADORA2A|ADORA2B|BRAF|DRD1|DRD2|GRIN1|GRIN2A|GRIN2B|HDAC2|MAOA|MAOB|NTRK2|SLC6A3	-9.01155	0	10	0	0	-13.945	35.64363
1	-9.54942	ko04720	M1	1	0	KEGG Pathway	24	Long-term potentiation	-9.54942	22.19868	13.55373	30242	67	183	9	4.918033	1.598524	673|775|2891|2902|2903|2904|2915|5582|6197	BRAF|CACNA1C|GRIA2|GRIN1|GRIN2A|GRIN2B|GRM5|PRKCG|RPS6KA3	-8.67133	0	10	0	0	-13.945	35.64363
1	-9.43157	hsa04720	M1	1	0	KEGG Pathway	24	Long-term potentiation	-9.43157	21.55524	13.33749	30242	69	183	9	4.918033	1.598524	673|775|2891|2902|2903|2904|2915|5582|61	BRAF|CACNA1C|GRIA2|GRIN1|GRIN2A|	-8.56572	0	10	0	0	-13.945	35.64363

																		97	GRIN2B|GRM5|PRKCG|RPS6KA3							
1	-8.37985	ko05033	M1	1	0	KEGG Pathway	24	Nicotine addiction	-8.37985	28.91995	13.78679	30242	40	183	7	3.825137	1.417845	2554|2558|2566|2891|2902|2903|2904	GABRA1|GABRA5|GABRG2|GRIA2|GRIN1|GRIN2A|GRIN2B	-7.61091	0	10	0	0	-13.945	35.64363
1	-8.22379	hsa05033	M1	1	0	KEGG Pathway	24	Nicotine addiction	-8.22379	27.54281	13.43088	30242	42	183	7	3.825137	1.417845	2554|2558|2566|2891|2902|2903|2904	GABRA1|GABRA5|GABRG2|GRIA2|GRIN1|GRIN2A|GRIN2B	-7.46753	0	10	0	0	-13.945	35.64363
1	-7.00015	hsa05010	M1	1	0	KEGG Pathway	24	Alzheimer's disease	-7.00015	9.664142	8.865149	30242	171	183	10	5.464481	1.680144	351|775|836|2902|2903|2904|4842|7124|7132|23621	APP|CACNA1C|CASP3|GRIN1|GRIN2A|GRIN2B|NOS1|TNF|TNFRSF1A|BACE1	-6.305	0	10	0	0	-13.945	35.64363
1	-7.00015	ko05010	M1	1	0	KEGG Pathway	24	Alzheimer's disease	-7.00015	9.664142	8.865149	30242	171	183	10	5.464481	1.680144	351|775|836|2902|2903|2904|4842|7124|7132|23621	APP|CACNA1C|CASP3|GRIN1|GRIN2A|GRIN2B|NOS1|TNF|TNFRSF1A|BACE1	-6.305	0	10	0	0	-13.945	35.64363
1	-5.57866	hsa04713	M1	1	0	KEGG Pathway	24	circadian entrainment	-5.57866	11.56798	8.259281	30242	100	183	7	3.825137	1.417845	775|2891|2902|2903|2904|4842|5582	CACNA1C|GRIA2|GRIN1|GRIN2A|GRIN2B|NOS1|PRKCG	-4.99535	0	10	0	0	-13.945	35.64363
1	-5.19952	ko04724	M1	1	0	KEGG Pathway	24	Glutamatergic synapse	-5.19952	10.14735	7.634822	30242	114	183	7	3.825137	1.417845	775|2891|2902|2903|2904|2915|5582	CACNA1C|GRIA2|GRIN1|GRIN2A|GRIN2B|GRM5|PRKCG	-4.6466	0	10	0	0	-13.945	35.64363
1	-5.12503	hsa04724	M1	1	0	KEGG Pathway	24	Glutamatergic synapse	-5.12503	9.887161	7.514997	30242	117	183	7	3.825137	1.417845	775|2891|2902|2903|2904|2915|5582	CACNA1C|GRIA2|GRIN1|GRIN2A|GRIN2B|GRM5|PRKCG	-4.57794	0	10	0	0	-13.945	35.64363
1	-2.70066	hsa05322	M1	1	0	KEGG Pathway	24	Systemic lupus erythematosus	-2.70066	5.659481	4.403479	30242	146	183	5	2.73224	1.205087	1645|1991|2903|2904|7124	AKR1C1|ELANE|GRIN2A|GRIN2B|TNF	-2.27414	0	10	0	0	-13.945	35.64363
1	-2.05432	ko05322	M1	1	0	KEGG Pathway	24	Systemic lupus erythematosus	-2.05432	4.97013	3.580295	30242	133	183	4	2.185792	1.080886	1991|2903|2904|7124	ELANE|GRIN2A|GRIN2B|TNF	-1.6602	0	10	0	0	-13.945	35.64363
1	-13.1438	hsa04750	M1	1	0	KEGG Pathway	24	inflammatory mediator regulation of trp channels	-13.1438	20.6571	15.66806	30242	104	183	13	7.103825	1.898976	624|3356|3357|3358|4914|5290|5291|5293|5582|5734|7442|8989|59341	BDKRB2|HTR2A|HTR2B|HTR2C|NTRK1|PIK3CA|PIK3CB|PIK3CD|PRKCG|PTGER4|TRPV1|TRPA1|TRPV4	-11.9435	0	11	1	1	-13.1438	20.6571
1	-11.2016	ko04540	M1	1	0	KEGG Pathway	24	Gap junction	-11.2016	20.6571	14.4087	30242	88	183	11	6.010929	1.757049	153|1453|1812|1813|1956|2915|3356|3357|3358|5159|5582	ADRB1|CSNK1D|DRD1|DRD2|EGFR|GRM5|HTR2A|HTR2B|HTR2C|PDGFRB|PRKCG	-10.1284	0	12	1	1	-11.2016	20.6571
1	-11.0923	hsa04540	M1	1	0	KEGG Pathway	24	Gap junction	-11.0923	20.19806	14.2317	30242	90	183	11	6.010929	1.757049	153|1453|1812|1813|1956|2915|3356|3357|3358|5159|5582	ADRB1|CSNK1D|DRD1|DRD2|EGFR|GRM5|HTR2A|HTR2B|HTR2C|PDGFRB|PRKCG	-10.0381	0	12	0	0	-11.2016	20.6571
1	-10.8272	ko05144	M1	1	0	KEGG Pathway	24	Malaria	-10.8272	30.3533	16.04494	30242	49	183	9	4.918033	1.598524	3383|3569|3576|3683|6347|6401|7040|7099|7124	ICAM1|IL6|CXCL8|ITGAL|CCL2|SELE|TGFB1|TLR4|TNF	-9.8144	0	13	1	1	-10.8272	30.3533
1	-10.3501	hsa05144	M1	1	0	KEGG Pathway	24	Malaria	-10.3501	27.04203	15.08282	30242	55	183	9	4.918033	1.598524	3383|3569|3576|3683|6347|6401|7040|7099|7124	ICAM1|IL6|CXCL8|ITGAL|CCL2|SELE|TGFB1|TLR4|TNF	-9.37506	0	13	0	0	-10.8272	30.3533

<table>
<tr><th></th><th></th><th></th><th></th><th></th><th></th><th></th><th></th><th></th><th></th><th></th><th></th><th></th><th></th><th></th><th></th><th></th><th></th><th></th><th></th><th></th><th></th><th></th><th></th><th></th><th></th><th></th></tr>
<tr><td>1</td><td>-9.71331</td><td>ko05323</td><td>M1</td><td>1</td><td>0</td><td>KEGG Pathway</td><td>24</td><td>Rheumatoid arthritis</td><td>-9.71331</td><td>18.36187</td><td>12.87052</td><td>30242</td><td>90</td><td>183</td><td>10</td><td>5.464481</td><td>1.680144</td><td>3383|3569|3576|3683|4312|4314|6347|7040|7099|7124</td><td>ICAM1|IL6|CXCL8|ITGAL|MMP1|MMP3|CCL2|TGFB1|TLR4|TNF</td><td>-8.82263</td><td>0</td><td>13</td><td>0</td><td>0</td><td>-10.8272</td><td>30.3533</td></tr>
<tr><td>1</td><td>-9.29856</td><td>hsa05323</td><td>M1</td><td>1</td><td>0</td><td>KEGG Pathway</td><td>24</td><td>Rheumatoid arthritis</td><td>-9.29856</td><td>16.69261</td><td>12.2027</td><td>30242</td><td>99</td><td>183</td><td>10</td><td>5.464481</td><td>1.680144</td><td>3383|3569|3576|3683|4312|4314|6347|7040|7099|7124</td><td>ICAM1|IL6|CXCL8|ITGAL|MMP1|MMP3|CCL2|TGFB1|TLR4|TNF</td><td>-8.44851</td><td>0</td><td>13</td><td>0</td><td>0</td><td>-10.8272</td><td>30.3533</td></tr>
<tr><td>1</td><td>-5.66716</td><td>ko05143</td><td>M1</td><td>1</td><td>0</td><td>KEGG Pathway</td><td>24</td><td>African trypanosomiasis</td><td>-5.66716</td><td>23.60812</td><td>10.44194</td><td>30242</td><td>35</td><td>183</td><td>5</td><td>2.73224</td><td>1.205087</td><td>3383|3569|5582|6401|7124</td><td>ICAM1|IL6|PRKCG|SELE|TNF</td><td>-5.07538</td><td>0</td><td>13</td><td>0</td><td>0</td><td>-10.8272</td><td>30.3533</td></tr>
<tr><td>1</td><td>-5.55633</td><td>ko05321</td><td>M1</td><td>1</td><td>0</td><td>KEGG Pathway</td><td>24</td><td>Inflammatory bowel disease (IBD)</td><td>-5.55633</td><td>15.25448</td><td>8.976484</td><td>30242</td><td>65</td><td>183</td><td>6</td><td>3.278689</td><td>1.316393</td><td>3558|3569|5970|7040|7099|7124</td><td>IL2|IL6|RELA|TGFB1|TLR4|TNF</td><td>-4.97927</td><td>0</td><td>13</td><td>0</td><td>0</td><td>-10.8272</td><td>30.3533</td></tr>
<tr><td>1</td><td>-5.47854</td><td>hsa05321</td><td>M1</td><td>1</td><td>0</td><td>KEGG Pathway</td><td>24</td><td>Inflammatory bowel disease (IBD)</td><td>-5.47854</td><td>14.79912</td><td>8.822699</td><td>30242</td><td>67</td><td>183</td><td>6</td><td>3.278689</td><td>1.316393</td><td>3558|3569|5970|7040|7099|7124</td><td>IL2|IL6|RELA|TGFB1|TLR4|TNF</td><td>-4.90764</td><td>0</td><td>13</td><td>0</td><td>0</td><td>-10.8272</td><td>30.3533</td></tr>
<tr><td>1</td><td>-5.16186</td><td>hsa05143</td><td>M1</td><td>1</td><td>0</td><td>KEGG Pathway</td><td>24</td><td>African trypanosomiasis</td><td>-5.16186</td><td>18.77919</td><td>9.208427</td><td>30242</td><td>44</td><td>183</td><td>5</td><td>2.73224</td><td>1.205087</td><td>3383|3569|5582|6401|7124</td><td>ICAM1|IL6|PRKCG|SELE|TNF</td><td>-4.61284</td><td>0</td><td>13</td><td>0</td><td>0</td><td>-10.8272</td><td>30.3533</td></tr>
<tr><td>1</td><td>-3.81751</td><td>ko05410</td><td>M1</td><td>1</td><td>0</td><td>KEGG Pathway</td><td>24</td><td>Hypertrophic cardiomyopathy (HCM)</td><td>-3.81751</td><td>9.955231</td><td>6.374465</td><td>30242</td><td>83</td><td>183</td><td>5</td><td>2.73224</td><td>1.205087</td><td>775|1636|3569|7040|7124</td><td>CACNA1C|ACE|IL6|TGFB1|TNF</td><td>-3.32307</td><td>0</td><td>13</td><td>0</td><td>0</td><td>-10.8272</td><td>30.3533</td></tr>
<tr><td>1</td><td>-3.69798</td><td>hsa05410</td><td>M1</td><td>1</td><td>0</td><td>KEGG Pathway</td><td>24</td><td>Hypertrophic cardiomyopathy (HCM)</td><td>-3.69798</td><td>9.389593</td><td>6.149589</td><td>30242</td><td>88</td><td>183</td><td>5</td><td>2.73224</td><td>1.205087</td><td>775|1636|3569|7040|7124</td><td>CACNA1C|ACE|IL6|TGFB1|TNF</td><td>-3.21481</td><td>0</td><td>13</td><td>0</td><td>0</td><td>-10.8272</td><td>30.3533</td></tr>
<tr><td>1</td><td>-2.70729</td><td>ko05332</td><td>M1</td><td>1</td><td>0</td><td>KEGG Pathway</td><td>24</td><td>Graft-versus-host disease</td><td>-2.70729</td><td>12.09196</td><td>5.545313</td><td>30242</td><td>41</td><td>183</td><td>3</td><td>1.639344</td><td>0.938686</td><td>3558|3569|7124</td><td>IL2|IL6|TNF</td><td>-2.27931</td><td>0</td><td>13</td><td>0</td><td>0</td><td>-10.8272</td><td>30.3533</td></tr>
<tr><td>1</td><td>-2.65542</td><td>ko05414</td><td>M1</td><td>1</td><td>0</td><td>KEGG Pathway</td><td>24</td><td>Dilated cardiomyopathy</td><td>-2.65542</td><td>7.344748</td><td>4.703422</td><td>30242</td><td>90</td><td>183</td><td>4</td><td>2.185792</td><td>1.080886</td><td>153|775|7040|7124</td><td>ADRB1|CACNA1C|TGFB1|TNF</td><td>-2.23036</td><td>0</td><td>13</td><td>0</td><td>0</td><td>-10.8272</td><td>30.3533</td></tr>
<tr><td>1</td><td>-2.56339</td><td>hsa05332</td><td>M1</td><td>1</td><td>0</td><td>KEGG Pathway</td><td>24</td><td>Graft-versus-host disease</td><td>-2.56339</td><td>10.77762</td><td>5.178139</td><td>30242</td><td>46</td><td>183</td><td>3</td><td>1.639344</td><td>0.938686</td><td>3558|3569|7124</td><td>IL2|IL6|TNF</td><td>-2.14268</td><td>0</td><td>13</td><td>0</td><td>0</td><td>-10.8272</td><td>30.3533</td></tr>
<tr><td>1</td><td>-2.4897</td><td>hsa05414</td><td>M1</td><td>1</td><td>0</td><td>KEGG Pathway</td><td>24</td><td>Dilated cardiomyopathy</td><td>-2.4897</td><td>6.610273</td><td>4.384644</td><td>30242</td><td>100</td><td>183</td><td>4</td><td>2.185792</td><td>1.080886</td><td>153|775|7040|7124</td><td>ADRB1|CACNA1C|TGFB1|TNF</td><td>-2.07472</td><td>0</td><td>13</td><td>0</td><td>0</td><td>-10.8272</td><td>30.3533</td></tr>
<tr><td>1</td><td>-2.48503</td><td>ko04672</td><td>M1</td><td>1</td><td>0</td><td>KEGG Pathway</td><td>24</td><td>Intestinal immune network for IgA production</td><td>-2.48503</td><td>10.11777</td><td>4.983904</td><td>30242</td><td>49</td><td>183</td><td>3</td><td>1.639344</td><td>0.938686</td><td>3558|3569|7040</td><td>IL2|IL6|TGFB1</td><td>-2.0729</td><td>0</td><td>13</td><td>0</td><td>0</td><td>-10.8272</td><td>30.3533</td></tr>
<tr><td>1</td><td>-2.25757</td><td>hsa04672</td><td>M1</td><td>1</td><td>0</td><td>KEGG Pathway</td><td>24</td><td>Intestinal immune network for IgA production</td><td>-2.25757</td><td>8.40289</td><td>4.441017</td><td>30242</td><td>59</td><td>183</td><td>3</td><td>1.639344</td><td>0.938686</td><td>3558|3569|7040</td><td>IL2|IL6|TGFB1</td><td>-1.85245</td><td>0</td><td>13</td><td>0</td><td>0</td><td>-10.8272</td><td>30.3533</td></tr>
<tr><td>1</td><td>-10.3112</td><td>ko05206</td><td>M1</td><td>1</td><td>0</td><td>KEGG Pathway</td><td>24</td><td>MicroRNAs in cancer</td><td>-10.3112</td><td>8.843175</td><td>10.63448</td><td>30242</td><td>299</td><td>183</td><td>16</td><td>8.743169</td><td>2.088053</td><td>472|595|596|836|1545|1956|3551|4318|4325|4363|5159|5243|5290|5582|5743|7157</td><td>ATM|CCND1|BCL2|CASP3|CYP1B1|EGFR|IKBKB|MMP9|MMP16|ABCC1|PDGFRB|ABCB1|PIK3CA|PRKCG|PTGS2|TP53</td><td>-9.3514</td><td>0</td><td>14</td><td>1</td><td>1</td><td>-10.3112</td><td>15.84655</td></tr>
<tr><td>1</td><td>-10.1411</td><td>hsa05206</td><td>M1</td><td>1</td><td>0</td><td>KEGG Pathway</td><td>24</td><td>MicroRNAs in cancer</td><td>-10.1411</td><td>8.612734</td><td>10.4606</td><td>30242</td><td>307</td><td>183</td><td>16</td><td>8.743169</td><td>2.088053</td><td>472|595|596|836|1545|1956|3551|4318|4325|4363|5159|5243|5290|5582|5743|7157</td><td>ATM|CCND1|BCL2|CASP3|CYP1B1|EGFR|IKBKB|MMP9|MMP16|ABCC1|PDGFRB|ABCB1|PIK3CA|PRKCG|PTGS2|TP53</td><td>-9.20091</td><td>0</td><td>14</td><td>0</td><td>0</td><td>-10.3112</td><td>15.84655</td></tr>
<tr><td>1</td><td>-6.51135</td><td>hsa01524</td><td>M1</td><td>1</td><td>0</td><td>KEGG Pathway</td><td>24</td><td>Platinum drug resistance</td><td>-6.51135</td><td>15.84655</td><td>9.909295</td><td>30242</td><td>73</td><td>183</td><td>7</td><td>3.825137</td><td>1.417845</td><td>472|596|836|5290|5291|5293|7157</td><td>ATM|BCL2|CASP3|PIK3CA|PIK3CB|PIK3CD|TP53</td><td>-5.8769</td><td>0</td><td>14</td><td>0</td><td>0</td><td>-10.3112</td><td>15.84655</td></tr>
</table>

1	-6.51135	ko01524	M1	1	0	KEGG Pathway	24	Platinum drug resistance	-6.51135	15.84655	9.909295	30242	73	183	7	3.825137	1.417845	472|596|836|5290|5291|5293|7157	ATM|BCL2|CASP3|PIK3CA|PIK3CB|PIK3CD|TP53	-5.8769	0	14	0	0	-10.3112	15.84655
1	-4.95924	ko04110	M1	1	0	KEGG Pathway	24	Cell cycle	-4.95924	9.329015	7.251498	30242	124	183	7	3.825137	1.417845	472|595|890|3066|5591|7040|7157	ATM|CCND1|CCNA2|HDAC2|PRKDC|TGFB1|TP53	-4.4179	0	14	0	0	-10.3112	15.84655
1	-4.78218	hsa04110	M1	1	0	KEGG Pathway	24	Cell cycle	-4.78218	8.76362	6.974807	30242	132	183	7	3.825137	1.417845	472|595|890|3066|5591|7040|7157	ATM|CCND1|CCNA2|HDAC2|PRKDC|TGFB1|TP53	-4.24652	0	14	0	0	-10.3112	15.84655
1	-3.91905	hsa04115	M1	1	0	KEGG Pathway	24	p53 signaling pathway	-3.91905	10.45929	6.568577	30242	79	183	5	2.73224	1.205087	472|595|596|836|7157	ATM|CCND1|BCL2|CASP3|TP53	-3.41772	0	14	0	0	-10.3112	15.84655
1	-3.08337	ko04115	M1	1	0	KEGG Pathway	24	p53 signaling pathway	-3.08337	9.580106	5.567296	30242	69	183	4	2.185792	1.080886	472|595|836|7157	ATM|CCND1|CASP3|TP53	-2.63125	0	14	0	0	-10.3112	15.84655
1	-9.90591	ko05219	M1	1	0	KEGG Pathway	24	Bladder cancer	-9.90591	32.24524	15.62073	30242	41	183	8	4.371585	1.511428	595|673|1956|3576|4312|4313|4318|7157	CCND1|BRAF|EGFR|CXCL8|MMP1|MMP2|MMP9|TP53	-9.00227	0	15	1	1	-9.90591	34.19107
1	-9.6443	hsa05219	M1	1	0	KEGG Pathway	24	Bladder cancer	-9.6443	30.0467	15.04424	30242	44	183	8	4.371585	1.511428	595|673|1956|3576|4312|4313|4318|7157	CCND1|BRAF|EGFR|CXCL8|MMP1|MMP2|MMP9|TP53	-8.76206	0	15	0	0	-9.90591	34.19107
1	-7.71819	ko05216	M1	1	0	KEGG Pathway	24	Thyroid cancer	-7.71819	34.19107	13.95275	30242	29	183	6	3.278689	1.316393	595|673|4914|5468|5979|7157	CCND1|BRAF|NTRK1|PPARG|RET|TP53	-6.98624	0	15	0	0	-9.90591	34.19107
1	-6.90314	hsa05216	M1	1	0	KEGG Pathway	24	Thyroid cancer	-6.90314	25.42413	11.90865	30242	39	183	6	3.278689	1.316393	595|673|4914|5468|5979|7157	CCND1|BRAF|NTRK1|PPARG|RET|TP53	-6.21605	0	15	0	0	-9.90591	34.19107
1	-9.21205	ko04723	M1	1	0	KEGG Pathway	24	Retrograde endocannabinoid signaling	-9.21205	16.36206	12.06612	30242	101	183	10	5.464481	1.680144	775|1268|2166|2554|2558|2566|2891|2915|5582|5743	CACNA1C|CNR1|FAAH|GABRA1|GABRA5|GABRG2|GRIA2|GRM5|PRKCG|PTGS2	-8.37199	0	16	1	1	-9.21205	16.36206
1	-8.34028	ko05032	M1	1	0	KEGG Pathway	24	Morphine addiction	-8.34028	16.34408	11.43792	30242	91	183	9	4.918033	1.598524	134|1812|2554|2558|2566|4988|5138|5142|5582	ADORA1|DRD1|GABRA1|GABRA5|GABRG2|OPRM1|PDE2A|PDE4B|PRKCG	-7.57455	0	16	0	0	-9.21205	16.36206
1	-8.29782	hsa05032	M1	1	0	KEGG Pathway	24	Morphine addiction	-8.29782	16.16643	11.36763	30242	92	183	9	4.918033	1.598524	134|1812|2554|2558|2566|4988|5138|5142|5582	ADORA1|DRD1|GABRA1|GABRA5|GABRG2|OPRM1|PDE2A|PDE4B|PRKCG	-7.53843	0	16	0	0	-9.21205	16.36206
1	-7.09676	hsa04723	M1	1	0	KEGG Pathway	24	Retrograde endocannabinoid signaling	-7.09676	9.895619	8.994312	30242	167	183	10	5.464481	1.680144	775|1268|2166|2554|2558|2566|2891|2915|5582|5743	CACNA1C|CNR1|FAAH|GABRA1|GABRA5|GABRG2|GRIA2|GRM5|PRKCG|PTGS2	-6.39614	0	16	0	0	-9.21205	16.36206
1	-4.70593	hsa04727	M1	1	0	KEGG Pathway	24	GABAergic synapse	-4.70593	10.89605	7.376805	30242	91	183	6	3.278689	1.316393	328|775|2554|2558|2566|5582	APEX1|CACNA1C|GABRA1|GABRA5|GABRG2|PRKCG	-4.17588	0	16	0	0	-9.21205	16.36206
1	-3.69798	ko04727	M1	1	0	KEGG Pathway	24	GABAergic synapse	-3.69798	9.389593	6.149589	30242	88	183	5	2.73224	1.205087	775|2554|2558|2566|5582	CACNA1C|GABRA1|GABRA5|GABRG2|PRKCG	-3.21481	0	16	0	0	-9.21205	16.36206
1	-8.9387	ko05202	M1	1	0	KEGG Pathway	24	Transcriptional misregulation in cancer	-8.9387	11.01712	10.51736	30242	180	183	12	6.557377	1.829835	472|1991|3066|3569|3576|4221|4314|4318|4914|5468|5970|7157	ATM|ELANE|HDAC2|IL6|CXCL8|MEN1|MMP3|MMP9|NTRK1|PPARG|RELA|TP53	-8.12221	0	17	1	1	-8.9387	11.01712

1	-8.32143	hsa05202	M1	1	0	KEGG Pathway	24	Transcriptional misregulation in cancer	-8.32143	9.72099	9.751731	30242	204	183	12	6.557377	1.829835	472|1991|3066|3569|3576|4221|4314|4318|4914|5468|5970|7157	ATM|ELANE|HDAC2|IL6|CXCL8|MEN1|MMP3|MMP9|NTRK1|PPARG|RELA|TP53	-7.55888	0	17	0	0	-8.9387	11.01712
1	-8.69967	ko04742	M1	1	0	KEGG Pathway	24	Taste transduction	-8.69967	17.91942	12.04349	30242	83	183	9	4.918033	1.598524	775|1131|2554|2558|3350|3351|5024|6326|6335	CACNA1C|CHRM3|GABRA1|GABRA5|HTR1A|HTR1B|P2RX3|SCN2A|SCN9A	-7.90761	0	18	1	1	-8.69967	17.91942
1	-8.60635	hsa04742	M1	1	0	KEGG Pathway	24	Taste transduction	-8.60635	17.49778	11.8844	30242	85	183	9	4.918033	1.598524	775|1131|2554|2558|3350|3351|5024|6326|6335	CACNA1C|CHRM3|GABRA1|GABRA5|HTR1A|HTR1B|P2RX3|SCN2A|SCN9A	-7.82435	0	18	0	0	-8.69967	17.91942
1	-6.83474	ko00380	M1	1	0	KEGG Pathway	24	Tryptophan metabolism	-6.83474	24.78852	11.7467	30242	40	183	6	3.278689	1.316393	217|1543|1544|1545|4128|4129	ALDH2|CYP1A1|CYP1A2|CYP1B1|MAOA|MAOB	-6.15295	0	19	1	1	-6.83474	55.08561
1	-6.23952	hsa00380	M1	1	0	KEGG Pathway	24	Tryptophan metabolism	-6.23952	19.83082	10.39788	30242	50	183	6	3.278689	1.316393	217|1543|1544|1545|4128|4129	ALDH2|CYP1A1|CYP1A2|CYP1B1|MAOA|MAOB	-5.62355	0	19	0	0	-6.83474	55.08561
1	-4.74896	M00135	M1	1	0	KEGG Pathway	24	GABA biosynthesis, eukaryotes, putrescine => GABA	-4.74896	55.08561	12.6619	30242	9	183	3	1.639344	0.938686	217|4128|4129	ALDH2|MAOA|MAOB	-4.21704	0	19	0	0	-6.83474	55.08561
1	-4.74896	hsa_M00135	M1	1	0	KEGG Pathway	24	GABA biosynthesis, eukaryotes, putrescine => GABA	-4.74896	55.08561	12.6619	30242	9	183	3	1.639344	0.938686	217|4128|4129	ALDH2|MAOA|MAOB	-4.21704	0	19	0	0	-6.83474	55.08561
1	-4.17	hsa00982	M1	1	0	KEGG Pathway	24	Drug metabolism - cytochrome P450	-4.17	11.80406	7.061066	30242	70	183	5	2.73224	1.205087	1544|1557|4128|4129|7364	CYP1A2|CYP2C19|MAOA|MAOB|UGT2B7	-3.66166	0	19	0	0	-6.83474	55.08561
1	-4.17	ko00982	M1	1	0	KEGG Pathway	24	Drug metabolism - cytochrome P450	-4.17	11.80406	7.061066	30242	70	183	5	2.73224	1.205087	1544|1557|4128|4129|7364	CYP1A2|CYP2C19|MAOA|MAOB|UGT2B7	-3.66166	0	19	0	0	-6.83474	55.08561
1	-4.09091	hsanan01	M1	1	0	KEGG Pathway	24	drug metabolism	-4.09091	8.474709	6.320626	30242	117	183	6	3.278689	1.316393	1544|1557|4128|4129|6530|7364	CYP1A2|CYP2C19|MAOA|MAOB|SLC6A2|UGT2B7	-3.58433	0	19	0	0	-6.83474	55.08561
1	-3.39607	ko00340	M1	1	0	KEGG Pathway	24	Histidine metabolism	-3.39607	20.6571	7.516725	30242	24	183	3	1.639344	0.938686	217|4128|4129	ALDH2|MAOA|MAOB	-2.92816	0	19	0	0	-6.83474	55.08561
1	-3.34249	hsa00340	M1	1	0	KEGG Pathway	24	Histidine metabolism	-3.34249	19.83082	7.349367	30242	25	183	3	1.639344	0.938686	217|4128|4129	ALDH2|MAOA|MAOB	-2.87938	0	19	0	0	-6.83474	55.08561
1	-6.59513	ko04976	M1	1	0	KEGG Pathway	24	Bile secretion	-6.59513	16.29293	10.0661	30242	71	183	7	3.825137	1.417845	760|1080|3156|5243|6513|6523|9429	CA2|CFTR|HMGCR|ABCB1|SLC2A1|SLC5A1|ABCG2	-5.94383	0	20	1	1	-6.59513	16.29293
1	-6.43006	hsa04976	M1	1	0	KEGG Pathway	24	Bile secretion	-6.43006	15.42397	9.758561	30242	75	183	7	3.825137	1.417845	760|1080|3156|5243|6513|6523|9429	CA2|CFTR|HMGCR|ABCB1|SLC2A1|SLC5A1|ABCG2	-5.80263	0	20	0	0	-6.59513	16.29293
1	-3.83632	ko02010	M1	1	0	KEGG Pathway	24	ABC transporters	-3.83632	15.02335	7.263155	30242	44	183	4	2.185792	1.080886	1080|4363|5243|9429	CFTR|ABCC1|ABCB1|ABCG2	-3.34017	0	20	0	0	-6.59513	16.29293
1	-3.7239	hsa02010	M1	1	0	KEGG Pathway	24	ABC transporters	-3.7239	14.06441	6.99371	30242	47	183	4	2.185792	1.080886	1080|4363|5243|9429	CFTR|ABCC1|ABCB1|ABCG2	-3.23624	0	20	0	0	-6.59513	16.29293
1	-6.23952	ko00330	M1	1	0	KEGG Pathway	24	Arginine and proline metabolism	-6.23952	19.83082	10.39788	30242	50	183	6	3.278689	1.316393	217|4128|4129|4842|4843|4846	ALDH2|MAOA|MAOB|NOS1|NOS2|NOS3	-5.62355	0	21	1	1	-6.23952	23.60812
1	-5.59621	hsa00330	M1	1	0	KEGG Pathway	24	Arginine and proline metabolism	-5.59621	15.49283	9.055955	30242	64	183	6	3.278689	1.316393	217|4128|4129|4842|4843|4846	ALDH2|MAOA|MAOB|NOS1|NOS2|NOS3	-5.01081	0	21	0	0	-6.23952	23.60812

1	-3.57263	ko00220	M1	1	0	KEGG Pathway	24	Arginine biosynthesis	-3.57263	23.60812	8.086415	30242	21	183	3	1.639344	0.938686	4842|4843|4846	NOS1|NOS2|NOS3	-3.0966	0	21	0	0	-6.23952	23.60812
1	-3.56522	hsa04371	M1	1	0	KEGG Pathway	24	Apelin signaling pathway	-3.56522	6.791377	5.473186	30242	146	183	6	3.278689	1.316393	185|595|4842|4843|4846|7046	AGTR1|CCND1|NOS1|NOS2|NOS3|TGFBR1	-3.09083	0	21	0	0	-6.23952	23.60812
1	-3.10534	hsa00220	M1	1	0	KEGG Pathway	24	Arginine biosynthesis	-3.10534	16.52568	6.638317	30242	30	183	3	1.639344	0.938686	4842|4843|4846	NOS1|NOS2|NOS3	-2.65167	0	21	0	0	-6.23952	23.60812
1	-5.92047	hsa05204	M1	1	0	KEGG Pathway	24	Chemical carcinogenesis	-5.92047	12.99773	8.844334	30242	89	183	7	3.825137	1.417845	1543|1544|1545|1557|5743|6530|7364	CYP1A1|CYP1A2|CYP1B1|CYP2C19|PTGS2|SLC6A2|UGT2B7	-5.31566	0	22	1	1	-5.92047	17.09553
1	-5.85088	ko00140	M1	1	0	KEGG Pathway	24	Steroid hormone biosynthesis	-5.85088	17.09553	9.573425	30242	58	183	6	3.278689	1.316393	1543|1544|1545|1588|1645|7364	CYP1A1|CYP1A2|CYP1B1|CYP19A1|AKR1C1|UGT2B7	-5.24828	0	22	0	0	-5.92047	17.09553
1	-5.72011	hsa00140	M1	1	0	KEGG Pathway	24	Steroid hormone biosynthesis	-5.72011	16.25477	9.305509	30242	61	183	6	3.278689	1.316393	1543|1544|1545|1588|1645|7364	CYP1A1|CYP1A2|CYP1B1|CYP19A1|AKR1C1|UGT2B7	-5.12403	0	22	0	0	-5.92047	17.09553
1	-4.96597	ko05204	M1	1	0	KEGG Pathway	24	Chemical carcinogenesis	-4.96597	12.09196	7.847585	30242	82	183	6	3.278689	1.316393	1543|1544|1545|1557|5743|7364	CYP1A1|CYP1A2|CYP1B1|CYP2C19|PTGS2|UGT2B7	-4.42272	0	22	0	0	-5.92047	17.09553
1	-4.88437	ko04913	M1	1	0	KEGG Pathway	24	Ovarian steroidogenesis	-4.88437	16.52568	8.572868	30242	50	183	5	2.73224	1.205087	1543|1545|1588|3643|5743	CYP1A1|CYP1B1|CYP19A1|INSR|PTGS2	-4.34493	0	22	0	0	-5.92047	17.09553
1	-4.64115	hsa04913	M1	1	0	KEGG Pathway	24	Ovarian steroidogenesis	-4.64115	14.75507	8.038787	30242	56	183	5	2.73224	1.205087	1543|1545|1588|3643|5743	CYP1A1|CYP1B1|CYP19A1|INSR|PTGS2	-4.11294	0	22	0	0	-5.92047	17.09553
1	-4.0543	ko00980	M1	1	0	KEGG Pathway	24	Metabolism of xenobiotics by cytochrome P450	-4.0543	11.166	6.831706	30242	74	183	5	2.73224	1.205087	1543|1544|1545|1645|7364	CYP1A1|CYP1A2|CYP1B1|AKR1C1|UGT2B7	-3.54948	0	22	0	0	-5.92047	17.09553
1	-3.99902	hsa00980	M1	1	0	KEGG Pathway	24	Metabolism of xenobiotics by cytochrome P450	-3.99902	10.87216	6.723516	30242	76	183	5	2.73224	1.205087	1543|1544|1545|1645|7364	CYP1A1|CYP1A2|CYP1B1|AKR1C1|UGT2B7	-3.49595	0	22	0	0	-5.92047	17.09553
1	-2.14076	ko00830	M1	1	0	KEGG Pathway	24	Retinol metabolism	-2.14076	7.627238	4.173377	30242	65	183	3	1.639344	0.938686	1543|1544|7364	CYP1A1|CYP1A2|UGT2B7	-1.73979	0	22	0	0	-5.92047	17.09553
1	-2.10448	hsa00830	M1	1	0	KEGG Pathway	24	Retinol metabolism	-2.10448	7.39956	4.091666	30242	67	183	3	1.639344	0.938686	1543|1544|7364	CYP1A1|CYP1A2|UGT2B7	-1.70763	0	22	0	0	-5.92047	17.09553
1	-5.53708	ko00910	M1	1	0	KEGG Pathway	24	Nitrogen metabolism	-5.53708	38.88396	12.19082	30242	17	183	4	2.185792	1.080886	759|760|761|762	CA1|CA2|CA3|CA4	-4.96414	0	23	1	1	-5.53708	38.88396
1	-5.42999	hsa00910	M1	1	0	KEGG Pathway	24	Nitrogen metabolism	-5.42999	36.72374	11.82915	30242	18	183	4	2.185792	1.080886	759|760|761|762	CA1|CA2|CA3|CA4	-4.86113	0	23	0	0	-5.53708	38.88396
1	-5.34089	ko04144	M1	1	0	KEGG Pathway	24	Endocytosis	-5.34089	6.356032	6.767619	30242	260	183	10	5.464481	1.680144	153|154|1956|2149|2263|3577|4914|5979|7040|7046	ADRB1|ADRB2|EGFR|F2R|FGFR2|CXCR1|NTRK1|RET|TGFB1|TGFBR1	-4.78008	0	24	1	1	-5.34089	6.356032
1	-5.18261	hsa04144	M1	1	0	KEGG Pathway	24	Endocytosis	-5.18261	6.098038	6.577691	30242	271	183	10	5.464481	1.680144	153|154|1956|2149|2263|3577|4914|5979|7040|7046	ADRB1|ADRB2|EGFR|F2R|FGFR2|CXCR1|NTRK1|RET|TGFB1|TGFBR1	-4.63164	0	24	0	0	-5.34089	6.356032
1	-4.38792	ko04921	M1	1	0	KEGG Pathway	24	Oxytocin signaling pathway	-4.38792	7.610512	6.375029	30242	152	183	7	3.825137	1.417845	595|775|1956|4846|5582|5743|9475	CCND1|CACNA1C|EGFR|NOS3|PRKCG|PTGS2|ROCK2	-3.86886	0	25	1	1	-4.38792	7.610512
1	-4.22938	hsa04921	M1	1	0	KEGG Pathway	24	Oxytocin signaling pathway	-4.22938	7.18508	6.139718	30242	161	183	7	3.825137	1.417845	595|775|1956|4846|5582|5743|9475	CCND1|CACNA1C|EGFR|NOS3|PRKCG|PTGS2|ROCK2	-3.7175	0	25	0	0	-4.38792	7.610512
1	-2.74008	ko04310	M1	1	0	KEGG Pathway	24	Wnt signaling pathway	-2.74008	5.778211	4.468828	30242	143	183	5	2.73224	1.205087	595|5467|5582|7157|9475	CCND1|PPARD|PRKCG|TP53|ROCK2	-2.30916	0	25	0	0	-4.38792	7.610512
1	-2.42705	hsa04310	M1	1	0	KEGG Pathway	24	Wnt signaling	-2.42705	4.889255	3.956014	30242	169	183	5	2.73224	1.205087	595|5467|5582|7157|9	CCND1|PPARD|PRK	-2.01773	0	25	0	0	-4.38792	7.610512

								pathway										475	CG|TP53|ROCK2							
1	-3.84238	ko04925	M1	1	0	KEGG Pathway	24	Aldosterone synthesis and secretion	-3.84238	10.07664	6.421737	30242	82	183	5	2.73224	1.205087	185|775|5138|5582|51305	AGTR1|CACNA1C|PDE2A|PRKCG|KCNK9	-3.34278	0	26	1	1	-3.84238	10.07664
1	-3.38048	hsa04925	M1	1	0	KEGG Pathway	24	aldosterone synthesis and secretion	-3.38048	8.022176	5.570091	30242	103	183	5	2.73224	1.205087	185|775|5138|5582|51305	AGTR1|CACNA1C|PDE2A|PRKCG|KCNK9	-2.91577	0	26	0	0	-3.84238	10.07664
1	-3.73888	hsa05016	M1	1	0	KEGG Pathway	24	Huntington's disease	-3.73888	5.993771	5.43036	30242	193	183	7	3.825137	1.417845	836|2902|2904|2915|3066|5468|7157	CASP3|GRIN1|GRIN2B|GRM5|HDAC2|PPARG|TP53	-3.24785	0	27	1	1	-3.73888	5.993771
1	-3.73888	ko05016	M1	1	0	KEGG Pathway	24	Huntington's disease	-3.73888	5.993771	5.43036	30242	193	183	7	3.825137	1.417845	836|2902|2904|2915|3066|5468|7157	CASP3|GRIN1|GRIN2B|GRM5|HDAC2|PPARG|TP53	-3.24785	0	27	0	0	-3.73888	5.993771
1	-3.7239	ko04961	M1	1	0	KEGG Pathway	24	Endocrine and other factor-regulated calcium reabsorption	-3.7239	14.06441	6.99371	30242	47	183	4	2.185792	1.080886	624|2099|5582|7421	BDKRB2|ESR1|PRKCG|VDR	-3.23624	0	28	1	1	-3.7239	14.06441
1	-3.61905	hsa04961	M1	1	0	KEGG Pathway	24	Endocrine and other factor-regulated calcium reabsorption	-3.61905	13.22055	6.74786	30242	50	183	4	2.185792	1.080886	624|2099|5582|7421	BDKRB2|ESR1|PRKCG|VDR	-3.13973	0	28	0	0	-3.7239	14.06441
1	-3.62985	hsa04610	M1	1	0	KEGG Pathway	24	Complement and coagulation cascades	-3.62985	9.080046	6.023099	30242	91	183	5	2.73224	1.205087	624|1645|2147|2149|2155	BDKRB2|AKR1C1|F2|F2R|F7	-3.14888	0	29	1	1	-3.62985	9.080046
1	-2.86368	ko04610	M1	1	0	KEGG Pathway	24	Complement and coagulation cascades	-2.86368	8.367434	5.115981	30242	79	183	4	2.185792	1.080886	624|2147|2149|2155	BDKRB2|F2|F2R|F7	-2.4268	0	29	0	0	-3.62985	9.080046
1	-3.60769	hsa04911	M1	1	0	KEGG Pathway	24	Insulin secretion	-3.60769	8.981349	5.982228	30242	92	183	5	2.73224	1.205087	775|1131|3778|5582|6513	CACNA1C|CHRM3|KCNMA1|PRKCG|SLC2A1	-3.13002	0	30	1	1	-3.60769	8.981349
1	-3.5217	ko04972	M1	1	0	KEGG Pathway	24	Pancreatic secretion	-3.5217	8.607127	5.824756	30242	96	183	5	2.73224	1.205087	760|1080|1131|3778|5582	CA2|CFTR|CHRM3|KCNMA1|PRKCG	-3.04973	0	30	0	0	-3.60769	8.981349
1	-3.38048	hsa04972	M1	1	0	KEGG Pathway	24	Pancreatic secretion	-3.38048	8.022176	5.570091	30242	103	183	5	2.73224	1.205087	760|1080|1131|3778|5582	CA2|CFTR|CHRM3|KCNMA1|PRKCG	-2.91577	0	30	0	0	-3.60769	8.981349
1	-2.94761	ko04971	M1	1	0	KEGG Pathway	24	Gastric acid secretion	-2.94761	8.813698	5.28637	30242	75	183	4	2.185792	1.080886	760|1080|1131|5582	CA2|CFTR|CHRM3|PRKCG	-2.50471	0	30	0	0	-3.60769	8.981349
1	-2.92617	hsa04971	M1	1	0	KEGG Pathway	24	Gastric acid secretion	-2.92617	8.697728	5.242601	30242	76	183	4	2.185792	1.080886	760|1080|1131|5582	CA2|CFTR|CHRM3|PRKCG	-2.48629	0	30	0	0	-3.60769	8.981349
1	-3.31368	ko04730	M1	1	0	KEGG Pathway	24	Long-term depression	-3.31368	11.01712	6.060116	30242	60	183	4	2.185792	1.080886	673|2891|4842|5582	BRAF|GRIA2|NOS1|PRKCG	-2.85216	0	31	1	1	-3.31368	11.01712
1	-3.25934	hsa04730	M1	1	0	KEGG Pathway	24	Long-term depression	-3.25934	10.66173	5.94193	30242	62	183	4	2.185792	1.080886	673|2891|4842|5582	BRAF|GRIA2|NOS1|PRKCG	-2.80098	0	31	0	0	-3.31368	11.01712
1	-3.25934	ko00590	M1	1	0	KEGG Pathway	24	Arachidonic acid metabolism	-3.25934	10.66173	5.94193	30242	62	183	4	2.185792	1.080886	1557|5742|5743|6916	CYP2C19|PTGS1|PTGS2|TBXAS1	-2.80098	0	32	1	1	-3.25934	10.66173
1	-3.2329	hsa00590	M1	1	0	KEGG Pathway	24	Arachidonic acid metabolism	-3.2329	10.4925	5.88484	30242	63	183	4	2.185792	1.080886	1557|5742|5743|6916	CYP2C19|PTGS1|PTGS2|TBXAS1	-2.7761	0	32	0	0	-3.25934	10.66173
1	-3.05985	hsa05416	M1	1	0	KEGG Pathway	24	Viral myocarditis	-3.05985	9.443247	5.518142	30242	70	183	4	2.185792	1.080886	595|836|3383|3683	CCND1|CASP3|ICAM1|ITGAL	-2.60928	0	33	1	1	-3.05985	9.443247
1	-3.01392	ko03320	M1	1	0	KEGG Pathway	24	PPAR signaling pathway	-3.01392	9.180935	5.422729	30242	72	183	4	2.185792	1.080886	4312|5465|5467|5468	MMP1|PPARA|PPARD|PPARG	-2.56643	0	34	1	1	-3.01392	9.180935
1	-2.72766	hsa03320	M1	1	0	KEGG Pathway	24	PPAR signaling pathway	-2.72766	7.686364	4.844944	30242	86	183	4	2.185792	1.080886	4312|5465|5467|5468	MMP1|PPARA|PPARD|PPARG	-2.2982	0	34	0	0	-3.01392	9.180935
1	-3.01392	ko04520	M1	1	0	KEGG Pathway	24	Adherens junction	-3.01392	9.180935	5.422729	30242	72	183	4	2.185792	1.080886	52|1956|3643|7046	ACP1|EGFR|INSR|TGFBR1	-2.56643	0	35	1	1	-3.01392	9.180935

1	-2.82347	hsa04520	M1	1	0	KEGG Pathway	24	Adherens junction	-2.82347	8.160831	5.035231	30242	81	183	4	2.185792	1.080886	52|1956|3643|7046	ACP1|EGFR|INSR|TGFBR1	-2.38809	0	35	0	0	-3.01392	9.180935
1	-2.98278	hsa04215	M1	1	0	KEGG Pathway	24	Apoptosis - multiple species	-2.98278	15.02335	6.288932	30242	33	183	3	1.639344	0.938686	596|836|7132	BCL2|CASP3|TNFRSF1A	-2.53835	0	36	1	1	-2.98278	15.02335
1	-2.98278	ko04215	M1	1	0	KEGG Pathway	24	Apoptosis - multiple species	-2.98278	15.02335	6.288932	30242	33	183	3	1.639344	0.938686	596|836|7132	BCL2|CASP3|TNFRSF1A	-2.53835	0	36	0	0	-2.98278	15.02335
1	-2.53666	ko04340	M1	1	0	KEGG Pathway	24	Hedgehog signaling pathway	-2.53666	10.54831	5.111451	30242	47	183	3	1.639344	0.938686	595|596|1453	CCND1|BCL2|CSNK1D	-2.11883	0	36	0	0	-2.98278	15.02335
1	-2.48503	hsa04340	M1	1	0	KEGG Pathway	24	Hedgehog signaling pathway	-2.48503	10.11777	4.983904	30242	49	183	3	1.639344	0.938686	595|596|1453	CCND1|BCL2|CSNK1D	-2.0729	0	36	0	0	-2.98278	15.02335
1	-2.75344	hsa05012	M1	1	0	KEGG Pathway	24	Parkinson's disease	-2.75344	5.818902	4.491024	30242	142	183	5	2.73224	1.205087	135|836|1812|1813|6531	ADORA2A|CASP3|DRD1|DRD2|SLC6A3	-2.31955	0	37	1	1	-2.75344	5.818902
1	-2.14076	ko04137	M1	1	0	KEGG Pathway	24	Mitophagy - animal	-2.14076	7.627238	4.173377	30242	65	183	3	1.639344	0.938686	5970|7157|29110	RELA|TP53|TBK1	-1.73979	0	38	1	1	-2.14076	7.627238
1	-2.10448	hsa04137	M1	1	0	KEGG Pathway	24	Mitophagy - animal	-2.10448	7.39956	4.091666	30242	67	183	3	1.639344	0.938686	5970|7157|29110	RELA|TP53|TBK1	-1.70763	0	38	0	0	-2.14076	7.627238
